# Population Genomics in *Rhamdia quelen* (Heptapteridae, Siluriformes) Reveals Deep Divergence and Adaptation in the Neotropical Region

**DOI:** 10.3390/genes11010109

**Published:** 2020-01-17

**Authors:** Néstor Ríos, Adrián Casanova, Miguel Hermida, Belén G. Pardo, Paulino Martínez, Carmen Bouza, Graciela García

**Affiliations:** 1 Sección Genética Evolutiva, Facultad de Ciencias, UdelaR, Iguá 4225, Montevideo 11400, Uruguay; ggarcia@fcien.edu.uy; 2 Departamento de Zoología, Genética y Antropología Física, Facultad de Veterinaria, Campus de Lugo, Universidade de Santiago de Compostela, Avenida Carballo Calero s/n, E-27002 Lugo, Spain; adrian.casanova.chiclana@gmail.com (A.C.); miguel.hermida@usc.es (M.H.); belen.gomez@usc.es (B.G.P.); paulino.martinez@usc.es (P.M.); mcarmen.bouza@usc.es (C.B.); 3 Instituto de Acuicultura, Universidade de Santiago de Compostela, Campus Vida s/n, E-15782 Santiago de Compostela, Spain

**Keywords:** genomic divergence, environmental adaptation, *Rhamdia quelen*, RAD-seq

## Abstract

*Rhamdia quelen*, a Neotropical fish with hybridization between highly divergent mitochondrial DNA (mtDNA) lineages, represents an interesting evolutionary model. Previous studies suggested that there might be demographic differences between coastal lagoons and riverine environments, as well as divergent populations that could be reproductively isolated. Here, we investigated the genetic diversity pattern of this taxon in the Southern Neotropical Basin system that includes the La Plata Basin, Patos-Merin lagoon basin and the coastal lagoons draining to the SW Atlantic Ocean, through a population genomics approach using 2b-RAD-sequencing-derived single nucleotide polymorphisms (SNPs). The genomic scan identified selection footprints associated with divergence and suggested local adaptation environmental drivers. Two major genomic clusters latitudinally distributed in the Northern and Southern basins were identified, along with consistent signatures of divergent selection between them. Population structure based on the whole set of loci and on the presumptive neutral vs. adaptive loci showed deep genomic divergence between the two major clusters. Annotation of the most consistent SNPs under divergent selection revealed some interesting candidate genes for further functional studies. Moreover, signals of adaptation to a coastal lagoon environment mediated by purifying selection were found. These new insights provide a better understanding of the complex evolutionary history of *R. quelen* in the southernmost basin of the Neotropical region.

## 1. Introduction

The high Neotropical diversity of freshwater fishes is mostly explained by the hydrogeological history, paleontological evolution and climatic change of that region [1]. Over the last 120 million years, South America has experienced wide marine transgressions and regressions as a result of Pleistocene glaciations. Their influence on the hydrological pattern of the region has been extensive, given that a large portion of South America is characterized by low elevation and low topographic relief [2,3]. Marine transgressions have directly affected effective population sizes and have increased population subdivision and genetic isolation [3], while the extension of plains has allowed historical connections between basins through headwater capture [3,4]. In addition, it has been proposed that coastal refugia for salinity-tolerant fish lineages might have been available during glacial periods [5,6]. Some of these lineages have dispersed widely during the post-glacial reclamation of habitat [6,7]. Among Neotropical silver catfish, *Rhamdia quelen* (Quoy and Gaimard, 1824) (Heptapteridae, Siluriformes) is distributed from the northeast end of the Andes mountain range to the central part of Argentina [8] and constitutes a valuable species exploited in artisanal fisheries and regional aquaculture [9,10,11]. Moreover, *R. quelen* has been proposed as a priority species for conservation in its southernmost distribution because of its cultural and economic value [12]. Due to its complex evolutionary history, this species of catfish poses a systematic challenge and represents an appealing evolutionary model for the fish fauna of the Neotropical region. In the first systematic revision of the *Rhamdia* genus, more than 100 species were synonymized, 46 of which were synonymized with *R. quelen* [13]. Previous studies on this species based on mitochondrial DNA (mtDNA) data suggested different mtDNA lineages [11,14,15], while other authors provided morphological evidence supporting sister species previously included in *R. quelen* [10,16]. Those studies were based on two different mitochondrial markers, cytochrome b (*cytb*) and cytochrome c oxidase subunit I (*COI*); however, analyses at different geographical scales based on both markers have not been performed to date. Five *R. quelen cytb* mtDNA lineages (*Rq2*, *Rq4*, *Rq5a*, *Rq5b* and *Rq6*) have been identified in the basin system encompassing the La Plata Basin, Patos-Merin lagoon Basin, as well as rivers and lagoons draining to the SW Atlantic Ocean (altogether LP-PM-AO) [14]. Microsatellite data provided evidence of hybridization and introgression between two mtDNA lineages (*Rq4* and *Rq6*) in several basins of this region (Uruguay River, Negro River and Merin Lagoon basins) [17]. In addition, genetic structure unraveled differentiated population clusters at LP-PM-AO and even within a single basin [17]. Homing behavior and divergent selection have been proposed as possible explanations for these observations. Unlike the riverine populations, *R. quelen* from southwestern Atlantic coastal lagoon environments appeared geographically isolated and genetically differentiated and showed small effective population sizes associated with putative bottleneck events. In such an evolutionary context, it was hypothesized that genomic footprints of selection might be associated with local adaptation to different environments and could therefore be retrieved using genomic screening across populations [17].

Restriction site-associated DNA sequencing (RAD-seq) is a high-throughput technique that enables the simultaneous identification and genotyping of thousands of SNPs (Single Nucleotide Polymorphisms), indels and microsatellite markers in non-model organisms [18,19,20]. Genome-wide SNP population screening allows assessing genetic diversity, demographic and population structure, adaptive variation and the genetic architecture associated with traits of interest [21]. Neutral and adaptive genetic variation accounts for the demographic history and adaptive evolution of populations [22,23]. Adaptive genetic markers allow to identification of populations that experience different environmental conditions, such as temperature or salinity [24]. The use of both types of markers may improve conservation and management strategies for species [25,26]. Moreover, wild populations adapted to different environmental conditions could be useful as founders for aquaculture broodstock [23]. Adaptive genes under divergent selection display higher differentiation than the neutral background of genomes [25]. Additionally, genetic markers physically linked to genes under divergent selection could also show the same differentiation pattern due to linkage disequilibrium, a phenomenon known as “divergence hitchhiking” in the context of the genomic speciation models [26,27,28]. Population genome scans, such as RAD-seq analysis, have proved to be useful in providing a better understanding of the genome-wide pattern of genetic differentiation [29,30,31].

The purpose of this study was to characterize the genetic structure patterns of *R. quelen* in the LP-PM-AO basin system using 17,559 RAD-seq derived SNPs, at an average density of 60 kb assuming a genome size of 1 Gb [32]. The study also intended to find and functionally characterize potential adaptive loci in *R. quelen* using a consistent statistical genomic scanning approach. A further purpose was to integrate previous phylogenetic and population data of the species, particularly those focused on the mitochondrial *cytb* lineages using the *COI* gene to extend and redefine mtDNA lineage distribution in the LP-PM-AO basin system of the Neotropical region.

## 2. Materials and Methods

### 2.1. Sample Collection and DNA Extraction

All sampling protocols were approved by the CNEA (Comisión Nacional de Experimentación Animal) from Uruguay.

**Figure 1:**
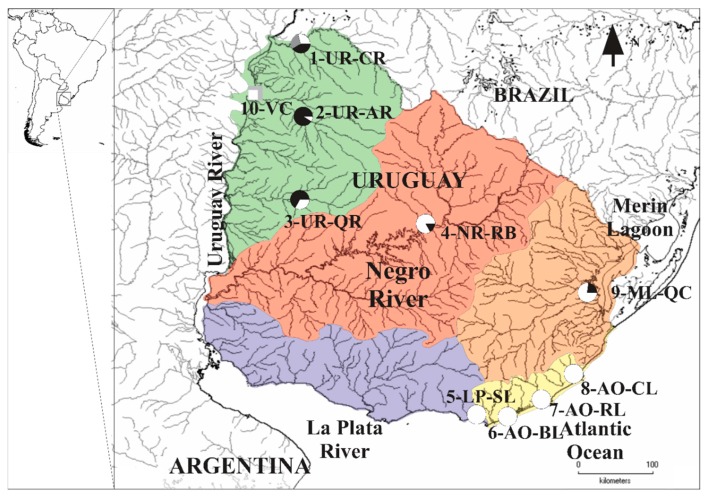
*Rhamdia quelen* samples analyzed from the La Plata Basin (LP), the Patos-Merin Basin system (PM) and the coastal lagoons draining to SW Atlantic Ocean (AO) (LP-PM-AO) in South America. Localities are included in different geographical basins, namely, the Uruguay River basin (UR: green): Cuareim River (1-UR-CR), Arapey River (2-UR-AR) and Queguay River (3-UR-QR); the Negro River basin (NR: red): Rincón del Bonete dam (4-NR-RB), Tacuarembó; the La Plata River basin (LP: purple): Sauce Lagoon (5-LP-SL); the SW Atlantic Ocean coastal basin (AO: yellow): Blanca Lagoon (6-AO-BL), Rocha Lagoon (7-AO-RL) and Castillos Lagoon (8-AO-CL); the Merin Lagoon basin (ML: orange): Quebrada de los Cuervos (9-ML-QC). Additionally, individuals from Villa Constitución hatchery (10-VC) were analyzed. Pie charts for each population display the mitochondrial lineage frequency of each population (Rq2: grey; Rq4: black and Rq6: white) based on Ríos et al. [17].

A total of 224 and 138 *R. quelen* individuals were analyzed, respectively, for *cytb* and *COI* mtDNA markers (Supplementary Files SI, SII and SIII), whereas genomic analyses were performed on 71 wild specimens belonging to nine localities from five major riverine and coastal lagoon basins. Populations analyzed were selected according to previous microsatellite and mtDNA genetic diversity patterns [14,17] (Figure 1; Table 1), namely, the Uruguay River basin (UR): Cuareim River (1-UR-CR), Arapey River (2-UR-AR) and Queguay River (3-UR-QR); the Negro River basin (NR): Rincón del Bonete dam (4-NR-RB); the La Plata River basin (LP): Sauce Lagoon (5-LP-SL); the SW Atlantic Ocean coastal basin (AO): Blanca Lagoon (6-AO-BL), Rocha Lagoon (7-AO-RL) and Castillos Lagoon (8-AO-CL); and the Merin Lagoon basin (ML): Quebrada de los Cuervos (9-ML-QC). Additionally, considering the value of *R. quelen* for aquaculture, we also analyzed three specimens from a hatchery (10-VC). The voucher specimens were stored in ethanol 95% either at Sección Genética Evolutiva, Colección de Zoología de Vertebrados de Facultad de Ciencias, or at the Museo de Historia Natural of Uruguay. Total genomic DNA was extracted from liver or muscle tissue using proteinase K digestion, followed by sodium chloride extraction and ethanol precipitation (modified from Medrano et al. [33]). The DNA quality was visualized by electrophoresis on 1% agarose gels and only non-degraded DNA samples were used.

### 2.2. Identification of Rhamdia quelen mtDNA Lineages Based on Partial Sequences of Cytb and COI

Partial sequences of *cytb* were amplified using fish universal DNA primers Gludg-L and CB3-H [34] following the polymerase chain reaction (PCR) protocol described by Ríos et al. [14]. A total of 246 *R. quelen* individuals and other *Rhamdia* species were analyzed for the *cytb* dataset, comprising 22 new samples collected for this study (Table 1, Supplementary File SI and SII) in addition to the 224 sequences retrieved from the GenBank [8,14,17,35,36] (Supplementary File SII). 

In order to integrate the data from both mtDNA markers reported in previous studies [10,11,14,15,37], partial sequences of *COI* gene were analyzed in at least three specimens from each *cytb* mtDNA lineage (*Rq2*, *Rq4* and *Rq6*, Ríos et al. [14]) along with 126 other *COI* sequences retrieved from the GenBank (Supplementary File SIII). The *COI* gene was partially amplified using the universal DNA primers LCO1490 and HC02198 [38]. The PCR cycling profile was as follows: an initial denaturation of 3 min at 94 °C, followed by four cycles of 94 °C for 1 min, 42 °C for 1 min and 72 °C for 1 min, then 29 cycles of 94 °C for 1 min, 50 °C for 1 min, 72 °C for 1 min and finally an extension of 7 min at 72 °C. The total reaction volume was 20 µl including 1× Buffer, 1.5 mM of MgCl_2_, 0.2 mM of each primer, 1 unit of Taq DNA polymerase (Invitrogen) and 45 ng of template DNA.

The nucleotide substitution models that best fit the *cytb* and *COI* datasets were selected in jModelTest v.2.1.17 [39], based on the Bayesian Information Criterion (BIC [40]), the corrected Akaike Information Criterion (AIC [41]) and the Decision Theory criterion (DT [42]). A heuristic Maximum Likelihood (ML) search was performed using NNI (a fast nearest-neighbor interchange search) and robustness of the nodes was assessed using 1000 bootstrap pseudoreplicates. ML phylogenetic analyses were performed on PhyML 3.1 [43]. Sequences of *Imparfinis mirini* Haseman, 1911 and *Pimelodella chagresi* (Steindachner, 1876) (Heptapteridae, Siluriformes) were used as outgroups in the *COI* phylogenetic tree. 

### 2.3. Library Construction and Sequencing

Library preparation followed the 2b-RAD protocol [44] with slight modifications [45]. Individual DNA samples were adjusted to 50 ng/μL. DNA was digested using *AlfI* (Thermo Fisher), an IIb type restriction enzyme (RE) that cleaves DNA on both sides of the restriction site. As a result of this digestion, the genome is fragmented in thousands of fragments of 36 bp in length to obtain a fraction of the genome. The 74 individual 2bRAD-seq libraries were quantified using Qubit 2.0 fluorometer (Life Technologies, Carlsbad, CA, USA) and equimolarly pooled. The pool of libraries was sequenced on a NextSeq 500 Illumina sequencer using a 50 bp single-end chemistry at the FISABIO Bioinformatic Service (Valencia, Spain). 

### 2.4. Data Processing and SNP Genotyping

Raw demultiplexed data were subjected to three filtering steps: (1) reads were trimmed to 36 nucleotides length and shorter reads were removed; (2) reads without *AlfI* recognition site in the correct position (i.e., (N)_12_GCA(N)_6_TGC(N)_12_) were removed; (3) reads with uncalled nucleotides or more than eight nucleotides with average Phred quality score below 30 (base call accuracy of 99.9%) were excluded using the process_radtags module in STACKS v.1.46 [46,47]. In the first two steps, our own Perl scripts were used. Raw reads and filtered reads quality were checked with FastQC 0.11.5 [48]. Similar STACKS input reads were oriented in the same direction to avoid loci over-splitting using cd-hit-est (CD-HIT [49]) and Bowtie 1.1.2 [50], which were included in an own Perl script. Filtered sequencing reads were processed using the loci-building pipelines STACKS v.1.46 and 2b-RAD v2.1 from Meyer’s Lab (the original loci-building pipeline for 2b-RAD-seq data [51]). SNP genotyping was performed using STACKS and 2b-RAD v2.1 loci-building pipelines without a reference genome. In the first one, the ustacks module was used to merge RAD-tags into putative loci for each individual. For this module, we allowed up to two mismatches among RAD-tags (-M 2) and a minimum depth of three identical reads to build a locus (-m 3). Ustacks was run using the bounded model type. Next, a catalog of loci was created by means of the cstacks module using all samples (n = 74) and allowing two mismatches (-n 2) among them. The rxstacks module was used to correct the genotype call and to filter confounded loci. The STACKS catalog rebuilt after applying the rxstacks module was checked with cd-hit-est to identify putative spurious loci (i.e., loci that should be in a single locus but still had a different orientation) or loci potentially due to indels. Loci that shared cd-hit-est clusters were included in a blacklist for the STACKS populations module. The populations module was finally used to obtain the initial SNP dataset, which included only loci with a minimum depth of 10 reads (-m 10), genotyped in at least 70% of total individuals and genotyped in no less than 60% of the individuals in at least seven populations (-r 0.60, -p 7). Finally, SNPs were filtered by MAF (Minimum Allele Frequency of the less frequent allele, > 0.05) and by intrapopulation fixation index F_IS_ [52] (loci with F_IS_ ≥ 0.50 and ≤ −0.50 were discarded to minimize the presence of null alleles in the former and of paralog sequences in the latter [23]). Additionally, RAD-tags with more than three SNPs were removed. 

For the 2b-RAD v2.1 pipeline, we applied the same parameter criteria as in STACKS for analogous parameters. Thus, a minimum coverage of 10 reads was required (-c 10), two mismatches were allowed among global references (-c 0.944, cd-hit-est parameter) and two mismatches were allowed to align reads with reference (-v 2, --best --strata, Bowtie parameters). Reference was built by selecting 300,000 reads of each sample (-n 300,000) to produce a combined dataset with more than 10 million reads. To determine genotypes from alignments, an interval for minor allele frequency (0.1–0.2) was established (consistent with the mean coverage observed in loci built by STACKS) to define if a locus is homozygous (minor allele frequency < 10% of locus coverage), unknown (10–20%) or heterozygous (> 20%). SNPs were filtered using an MAF threshold of 0.05 and based on the total percentage of individuals genotyped (70%) and on the percentage of individuals genotyped per populations (60%) (in at least seven populations). For both loci-building pipelines, SNPs at extreme RAD-tag positions (positions 1 and 36) were not used, following criteria proposed by Wang et al. [44], and only biallelic SNPs were retained. 

Finally, a consistently integrated catalog was constructed with the SNPs shared by both STACKS and 2b-RAD v2.1 loci-building pipelines. For this purpose, RAD-tag sequences with selected SNPs from both pipelines were merged and clustered with cd-hit-est (-c 0.944, the same value of mismatches used to build both loci-building pipeline references) to identify exclusive and shared RAD-tags from both catalogs. Coincident SNP positions within each RAD-tag pair were confirmed with both catalogs using ad hoc spreadsheets. The final catalog of SNPs was obtained considering a unique SNP per RAD-tag, after eliminating those loci that showed low or high F_IS_ values (F_IS_ ≤ −0.50 or F_IS_ ≥ 0. 50) in at least three locality samples. 

All RAD-tags bearing outlier SNPs of 36 bp were annotated using the GenBank database and Blastn search of BLAST (Basic Local Alignment Search Tool [53]). SNPs included in RAD-tags annotated with an *E*-value ˂ e^−4^, but not matching against Teleostei were filtered out. 

### 2.5. Detection of Loci under Selection

The Total dataset comprised 17,559 SNPs. Among these, the Outlier dataset comprised those SNPs identified by both BAYESCAN v.2.1 [54] and Arlequin v.3.5 [55] as outlier loci or candidate SNPs subjected to selection. Loci that were not detected as outliers using either method were considered neutral and composed the Neutral dataset. BayeScan and Arlequin follow different strategies to detect outliers; the former is considered a conservative program (type II error), while the latter is a more relaxed one (type I error) [56]. Both softwares were run to identify a confident set of outlier loci because the use of different F_ST_-based methods is recommended [56] and this strategy could be effective under certain genetic structures [57]. The BayeScan analysis was carried out for 20 pilot runs, 5000 iterations, 5000 burn-in steps and a sample size of 5000. BayeScan outliers were obtained applying a False Discovery Rate (FDR) of 0.01 and plotted using the R function provided by the program. Arlequin analyses were performed using a hierarchical finite island model testing 100 simulations with 100 simulated demes and 10 groups, and also using a non-hierarchical finite island model, testing 100 simulations with 100 demes. Loci with a *p*-value ˂ 0.01 in Arlequin analysis were selected as putative outliers. Different population groupings, according to previous data [7,10] and the present study (see Results) were explored with BayeScan and Arlequin to find the most consistent set of outlier loci (Supplementary File SIV). We analyzed outliers (i) for all locality samples (with and without the 10-VC hatchery sample); (ii) among mtDNA lineages (*Rq2*, *Rq4* and *Rq6*; Supplementary File SI); (iii) among latitudinal groups of samples from North, Centre and South; (iv) between coastal lagoons and riverine groups of samples. We also performed separate analyses for different sample subsets: (i) among samples within coastal lagoons or riverine environments (Supplementary File SI); (ii) all samples within each latitudinal group (North, Centre and South); (iii) pairwise comparisons of wild locality samples. Finally, based on the genetic structure observed using the Total, Neutral and Outlier marker datasets (See Structure results), we searched for outlier loci between the most divergent groups of samples from the Northern and Southern areas of the LP-PM-AO region studied. Thus, BayeScan and Arlequin (non-hierarchical model) analyses were performed considering the following seven samples as populations: 1-UR-CR; 2-UR-AR; 3-UR-QR; 5-LP-SL; 6-AO-BL; 7-AO-RL; 8-AO-CL. 

All SNP-bearing RAD-tags were annotated using the GenBank database and BLASTn search. Additionally, these RAD-tags were annotated using the *Ictalurus punctatus* (Rafinesque, 1818) genome [58] by means of BLASTn implemented in Ensembl [59]. Availability of chromosomal-assembled genome of the channel catfish *I. punctatus* in Ensembl opened opportunities for further comparative mapping purposes in this study (see below), although limitations for annotations may not be ruled out since *R. quelen* and *I. punctatus* belong to different Siluriformes families. An *E*-value ˂ e^−4^ was considered as the significance threshold for homologies in both searches. Hypothetical chromosomes that would include the annotated genes were analyzed through a BLASTn search against the *I. punctatus* genome. Gene mining around outlier loci was performed using Ensembl within the *I. punctatus* genome. Gene mining was performed only when two or more loci were anchored in the same chromosome region within a range of < 500 Kb. The biological process of the gene lists identified by BLAST and gene mining was performed with BLAST2GO [60]. 

### 2.6. Genetic Diversity and Population Structure of R. quelen in LP-PM-AO

For each locality, the mean number of alleles per locus (Na) and the expected heterozygosity were calculated. The magnitude and sign of departure from Hardy-Weinberg equilibrium (HWE) were analyzed using F_IS_. Na, F_IS,_ and H_E_ were calculated in Arlequin [55]. 

Population Structure analyses were performed using the Total, Neutral and Outlier SNP datasets. Analysis of clusters and graphical representation of genetic structure were performed by Discriminant Analysis of Principal Components (DAPC). Scatter plots were performed using the R package ADEGENET [61]. For Total, Neutral and Outlier datasets, the number of principal components retained was 50, 50 and 20, containing 90%, 90% and 99% of the cumulative variation of the data, respectively. Five, six and four discriminant functions were retained for Total, Neutral, Outlier datasets, respectively. Bayesian analysis of population structure was performed in STRUCTURE v. 2.3 [62] to cluster individuals into populations based on the Total, Neutral and Outlier dataset. Cluster numbers (K) were evaluated from 1 to the number of localities (10) plus 3 (K = 1 to K = 13). Ten independent runs for each K were implemented with a burn-in period length of 50,000 iterations, followed by 100,000 Monte Carlo Markov Chains (MCMC) replicates. An admixture model and correlated allele frequencies were assumed. The consensus result for each K was obtained from the independent runs by means of CLUMPAK [63]. The most probable K value was determined using both likelihood and Delta k criteria (ΔK) [64], and calculated using STRUCTURE HARVESTER [65]. In addition, the secondary structure was analyzed based on the same criteria. We also investigated the population structure using the fineRADstructure package v.0.2 [66], which allows estimating the most recent coalescence between individuals using a co-ancestry matrix. This software is more suitable for very large population numbers. FineRADstructure analyses were performed using 100,000 iterations as burn-in, followed by 1,000,000 MCMC replicates, and the resulting trees were built using 100,000 iterations. Furthermore, inter-sample genetic differentiation was calculated through pairwise F_ST_ estimates with Arlequin v.3.5, for Total, Neutral and Outlier loci. Finally, Isolation by distance (IBD) was analyzed using Mantel test based on F_ST_ and geographical distance matrixes. IBD was performed in Arlequin and based on Neutral loci because they might explain the geographic isolation in absence of gene flow [67], without the influence of selection, which could change the magnitude of population divergence.

### 2.7. Genetic Differentiation by Environmental Factors

A total of 19 bioclimatic variables (annual mean temperature; mean diurnal range; isothermality; temperature seasonality; maximum temperature of warmest month; minimum temperature of coldest month; annual temperature range; mean temperature of wettest quarter; mean temperature of driest quarter; mean temperature of warmest quarter; mean temperature of coldest quarter; annual precipitation; precipitation of wettest month; precipitation of driest month; precipitation seasonality; precipitation of wettest quarter; precipitation of driest quarter; precipitation of warmest quarter; precipitation of coldest quarter) were retrieved from WORLDCLIM database [68] for the nine natural localities. Conductivity (as a salinity indicator) data were obtained from various sources (1-UR-CR, 4-NR-RB, 5-LP-SL, 9-ML-QC [69]; 3-UR-QR, Colina, Kruk and Albo, unpublished data; 6-AO-BL [70]; 7-AO-RL and 8-AO-CL [71]). Conductivity from 2-UR-AR was measured in the field using the Extech EC500 conductivity/pH meter in February 2017. Values of environmental variables are shown in Supplementary File V. All variables were standardized for analyses, i.e., the mean was subtracted from each value and then divided by the standard deviation. Genetic differentiation associated with bioclimatic, conductivity or spatial (i.e., longitude and latitude) variables was studied through canonical redundancy analysis (RDA) using the R platform VEGAN software [72]. For this analysis, allelic frequencies were estimated using the ADEGENET R package. Due to missing values, and as required by VEGAN, 2739 loci were removed from the Total dataset, 2371 loci from the Neutral dataset and a single locus was discarded from the Outlier dataset. 

### 2.8. Evolutionary History of Populations

Population history was inferred using TREEMIX software [73]. This software uses the covariance matrix of allele frequency between pairs of populations to estimate historical relationships among them and to test different events of migration. First, the Maximum likelihood sample tree in the absence of migration was built; secondly, different numbers of migration events (1–10) were tested. TREEMIX analyses were based on the putative Neutral loci subset. Due to the fact that 4-NR-RB and 9-ML-QC locality samples showed an important genetic differentiation (see Structure results), they were subdivided, respectively, in 4-NR-RB-N, 4-NR-RB-S and 9-ML-QC-N, 9-ML-QC-S (Supplementary File I). 1-UR-CR locality sample was discarded from these analyses due to a too low sample size.

## 3. Results

### 3.1. MtDNA Lineages Identification Based on cytb and COI

In order to identify the different *R. quelen* mtDNA lineages, a total of 22 new partial sequences of *cytb* were obtained in this study (GenBank Accession numbers: MK511194–MK511214 and MK511219; Supplementary Files SI and SII). They were analyzed together with 224 *Rhamdia cytb* sequences retrieved from the GenBank (Supplementary File SII). In the *cytb* dataset of 745 bp, we detected 225 variable sites and 171 parsimony informative sites. The 246 *cytb* sequences were grouped into 20 haplotypes. Among the 88 models tested, the HKY + G (gamma distribution) [74] was the nucleotide substitution model that best fitted the *cytb* dataset. The *cytb* topology obtained by PhyML was similar to that by Ríos et al. [14], and the specimens sequenced in this study were identified as *Rq2*, *Rq4* and *Rq6* (Supplementary File SI). At the population level, these *cytb* lineages showed differential geographic distribution across North to South basins (Figure 1), in agreement with previous data from the region [17].

Partial sequences of *COI* were generated for each *cytb* mtDNA lineage (*Rq2*, *Rq4* and *Rq6*) in this study to anchor both types of markers (GenBank Accession numbers: MK511191–MK511193 and MN233634–MN233642; Supplementary File SIII). These *COI* sequences were analyzed together with 126 *Rhamdia COI* sequences retrieved from the GenBank (Supplementary File SIII). *COI* alignment of 551 bp showed 112 variable sites and 89 parsimony informative sites. The 138 *COI* sequences of *Rhamdia* were grouped into 41 haplotypes (Supplementary File SIII). According to the AIK, BIC and DT selection criteria, TIM2 + G [75] was the nucleotide substitution model that best fitted the *COI* dataset. *COI* topology showed six mtDNA lineages distributed in the cis-Andean region (*Rq2*, *Rq4,* and *Rq6*, according to Ríos et al. [14], together with other *COI1, COI2* and *COI3*, found in this article), as well as several sequences, which collapsed in a basal polytomy. Sequences belonging to each mtDNA lineage are detailed in Supplementary Files SIII and SVI.

### 3.2. Genome Data Processing and SNP Genotyping

A total of 341,495,357 reads were retrieved from the sequencing platform, representing, on average, 4,614,802 reads per specimen (range: 2,228,958–8,981,759). After applying sequence quality and RE site position filters, 223,148,950 reads were retained. 

After rxstacks correction, a cstacks catalog of 160,898 SNP loci was built. Population module results retrieved 60,332 loci. After the MAF and number of SNPs per RAD-tag filters, 33,376 loci were retained in the STACKS dataset. On the other hand, the 2b-RAD v2.1 pipeline catalog included a final set of 43,882 SNPs after applying quality and population filters. A total of 24,659 loci were shared between the STACKS and 2b-RAD v2.1 pipelines. Loci that showed high or low F_IS_ values in at least three locality samples were discarded, and the catalog was reduced to 17,575 loci. Finally, a total of 16 loci were discarded because RAD-tags were not homologous to Teleostei sequences, thus the Total dataset was composed of 17,559 loci (Supplementary File SVII).

### 3.3. Detection of Putative Loci under Selection

A total of 1975 outlier loci were detected by Arlequin and BayeScan in the different group comparisons tested (See Supplementary File SIV). All candidate loci under selection were removed from the Total dataset to build the Neutral dataset (15,584 loci). 

According to genetic structure results (see below), a consistent Outlier dataset was identified considering the two highly differentiated clusters and by comparing the Northern and Southern basins of the sampled area. In this analysis, a total of 174 and 524 outlier loci were found, respectively, by BayeScan and Arlequin. A final set of 73 loci detected by both methods was retained as the most confident Outlier dataset between the two major genomic clusters of *R. quelen*. 

Out of these 73 outlier loci, 21 were functionally annotated using their specific RAD-tag sequences and also anchored to 13 different *I. punctatus* chromosomes (Supplementary File SVIII). A list of genes related to different functional annotation terms was obtained through gene mining in those regions where at least two significant hits for the outlier SNPs in the *I. punctatus* genome were found within a range of < 500 Kb (Table 2). 

### 3.4. Genetic Diversity and Structure of R. quelen in LP-PM-AO

Genetic diversity showed an average of 1.35 (Na) and 0.286 (H_E_) across all populations. Na ranged from 1.20 (6-AO-BL) to 1.60 (4-NR-RB) (Table 3), while H_E_ ranged from 0.249 (5-LP-SL) to 0.328 (6-AO-BL). No significant signals of HWE departure were detected, and F_IS_ values were close to zero across all populations except for 4-NR-RB and 9-ML-QC, which showed high and significant positive F_IS_ values (Table 3). The amount of genetic diversity was similar across all localities. High and significant F_IS_ values found in the Negro River basin (4-NR-RB) and Merin Lagoon basin (9-ML-QC) might be explained by a Wahlund effect. When we split these samples by their genomic cluster assignment (4-NR-RB-N, 4-NR-RB-S, 9-ML-QC-N and 9-ML-QC-S; Table 1), F_IS_ was not significant in each sub-sample, although caution is required given the small sampling size.

**Figure 2:**
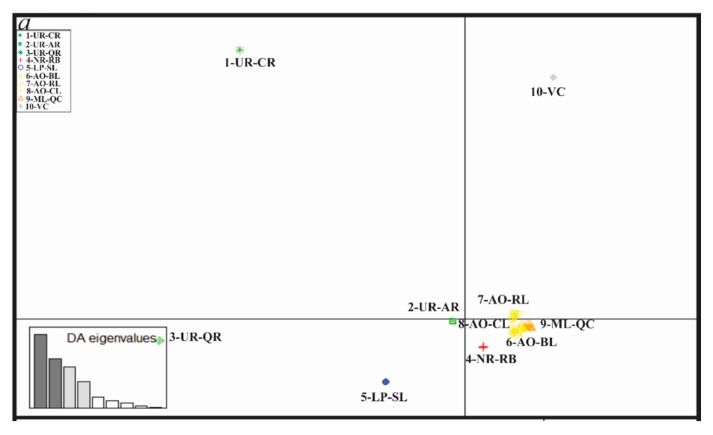

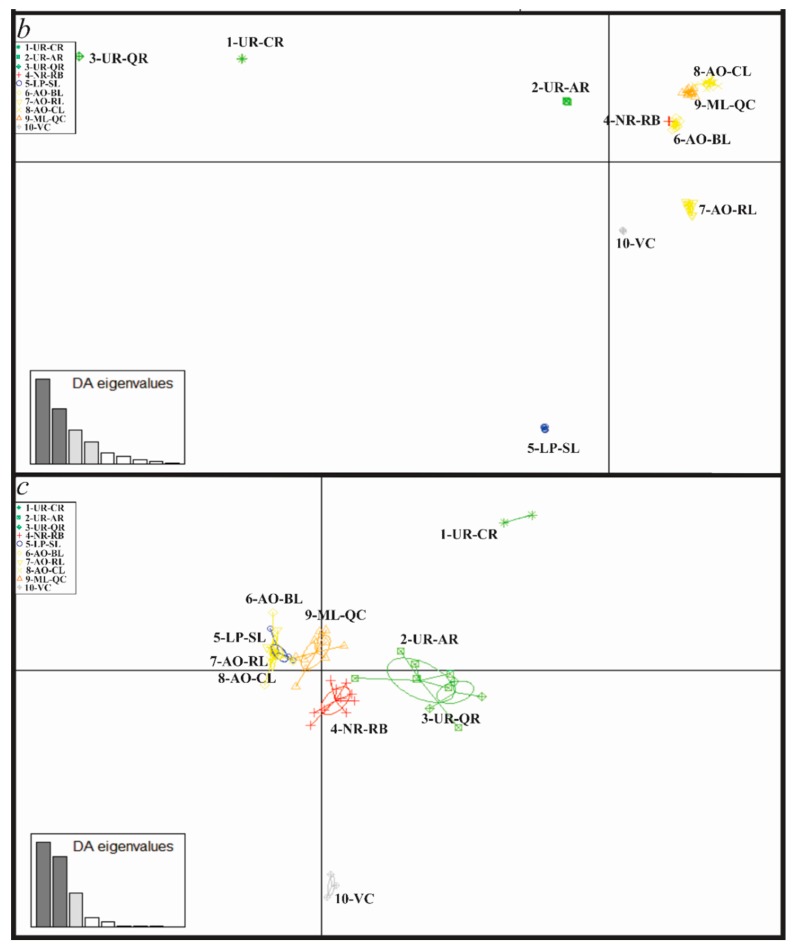
DAPC (Discriminant Analysis of Principal Components) scatterplot of *Rhamdia quelen* from the La Plata Basin, the Patos-Merin Basin system and the coastal lagoons draining to the SW Atlantic Ocean based on Total 17,559 (**a**), Neutral 15,584 (**b**) and Outlier 73 (**c**) loci. Individuals are grouped per locality (Figure 1). Eigenvalues containing, respectively, 90%, 90 % and 99 % of the cumulative variation of the data are displayed on the right or left bottom boxes. Colors refer to different basins as follows: Uruguay (green), Negro (red), La Plata (blue), Atlantic Ocean (yellow) and Merin Lagoon (orange). The hatchery sample is in gray.

DAPC results based on the Total, Neutral and Outlier loci showed rather similar patterns (Figure 2a–c). The samples per locality were arranged following a North to South geographic pattern, excluding the 2-UR-QR sites and the hatchery (10-VC). The three DAPC scatterplots revealed higher differentiation among Northern localities than among Southern ones.

**Figure 3:**
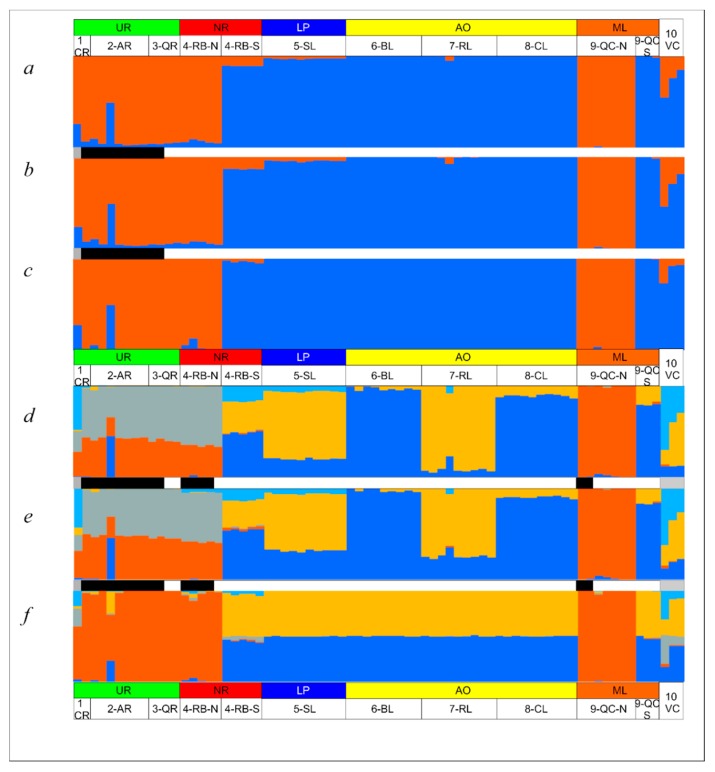
Estimated population structure (K = 2, ***a***–***c***; K = 5, ***d***–***f***) in STRUCTURE of *Rhamdia quelen* from the La Plata River basin, the Merin Lagoon and coastal lagoons from SW Atlantic Ocean based on Total 17,559 (***a***,***d***), Neutral 15,584 (***b***,***e***) and Outliers 73 (***c***,***f***) SNP loci. Each bin or colored vertical bar represents the estimated membership fraction of an individual in the major population clusters. Basin abbreviation and color together with sampling sites codes are indicated in Figure 1. Bar among histograms represent the mtDNA lineage of each individual as follows: *Rq2*: grey; *Rq4*: black; *Rq6*: white.

Structure analyses using the Total, Neutral and Outlier SNP datasets showed the best clustering outcomes for K = 2 (Figure 3a–c), well supported by Delta K (ΔK) and Ln (PX|K) criteria (Supplementary File SIX). Moreover, we show K = 5 for the three datasets (Total, Neutral and Outlier Figure 3d–f), which was the second-best ΔK score for Neutral loci. Although the second-best ΔK score for the Total dataset was K = 6, we show K = 5 for comparison purposes between patterns obtained from the three datasets. The Outlier dataset led to a clear estimation of two clusters (Figure 3c; ΔK = 3,536.15, Ln (PX|K) = −973.43), the remaining ΔK values being low and extremely close to each other (ΔK = 0.09–7.19) for all K tested. This included K = 5 which resulted congruent with the graphical pattern for K =2 (Figure 3f; ΔK = 0.09; Ln (PX|K) = −985.25) in opposition to the substructure pattern observed for K = 5 using the Total (Figure 3d) and Neutral (Figure 3e) datasets. 

Structure histograms for K = 2 based on the Total, Neutral and Outlier data (Figure 3a–c) were concordant, and localities were grouped into two major clusters geographically associated, respectively, with the Northern and Southern basins (Figure 1): Cluster 1): 1-UR-CR, 2-UR-AR, 3-UR-QR, some fish from 4-NR-RB and 9-ML-QC; and Cluster 2): 5-LP-SL, 6-AO-BL, 7-AO-RL, 8-AO-CL, and the remaining fish from 4-NR-RB and 9-ML-QC. Hatchery individuals (10-VC) were not completely assigned to any cluster, showing signs of admixture between them.

**Figure 4:**
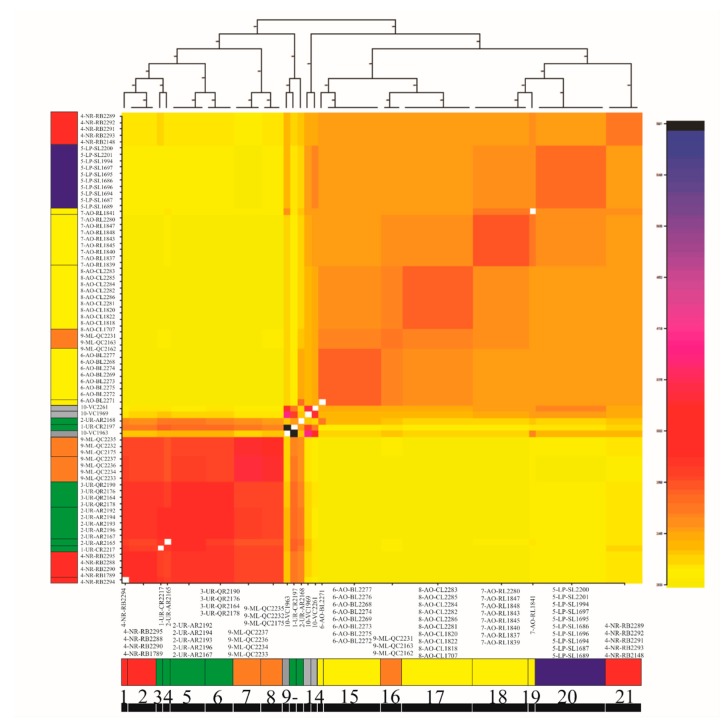
*Rhamdia quelen* fineRADstructure plot based on Total *loci*. The horizontal axis shows clusters of individuals and the vertical axis shows each individual. Pairwise coefficients of co-ancestry are color-coded from low (yellow) to high (black). The dendrogram shows a clustering of individual samples based on the pairwise matrix of co-ancestry coefficients. Legends on the left and below the co-ancestry matrix show the name of each individual, and their corresponding basins are colored according to the code used in Figure 1 (Uruguay River basin: green; Negro River basin: red; La Plata River basin: blue; coastal SW Atlantic Ocean basin: yellow; Merin lagoon: orange). Numbers along the bottom line indicate the different minor clusters detected.

Data analyses of the Total and Neutral datasets with fineRADstructure (Figure 4 and Supplementary File SX: Figure S1) showed a large number of populations mostly grouped in two major clusters as mentioned before. FineRADstructure analysis based on Outlier data (Supplementary File SX: Figure S2) resulted in two major clusters that were concordant with Structure K = 2 (Figure 3b–c).

Pairwise genetic differentiation results were significant for most comparisons tested (Table 4, Table 5 and Table 6). Pairwise F_ST_ values were similar between Total and Neutral datasets (Table 4 and Table 5). Total, Neutral and Outlier analyses showed high differentiation between locality samples from the Northern (1-UR-CR, 2-UR-AR, 3-UR-QR) and Southern (5-LP-SL, 6-AO-BL, 7-AO-RL, 8-AO-CL) basins in the studied region. However, by contrast to the Total and Neutral analyses (mean F_ST_: 0.215 and 0.180, respectively), the Outlier dataset did not evidence genetic differentiation among coastal lagoons (F_ST_: 0.000; Table 6). In addition, much higher F_ST_ values between localities from the North and South Clusters were obtained using Outlier SNPs (mean F_ST_: 0.939) than when using the Total and Neutral datasets (mean F_ST_: 0.527 and 0.336, respectively). Mantel test was not significant; hence, it did not support the IBD hypothesis (*p* = 0.016).

### 3.5. Genetic Differentiation by Environmental Factors

**Figure 5:**
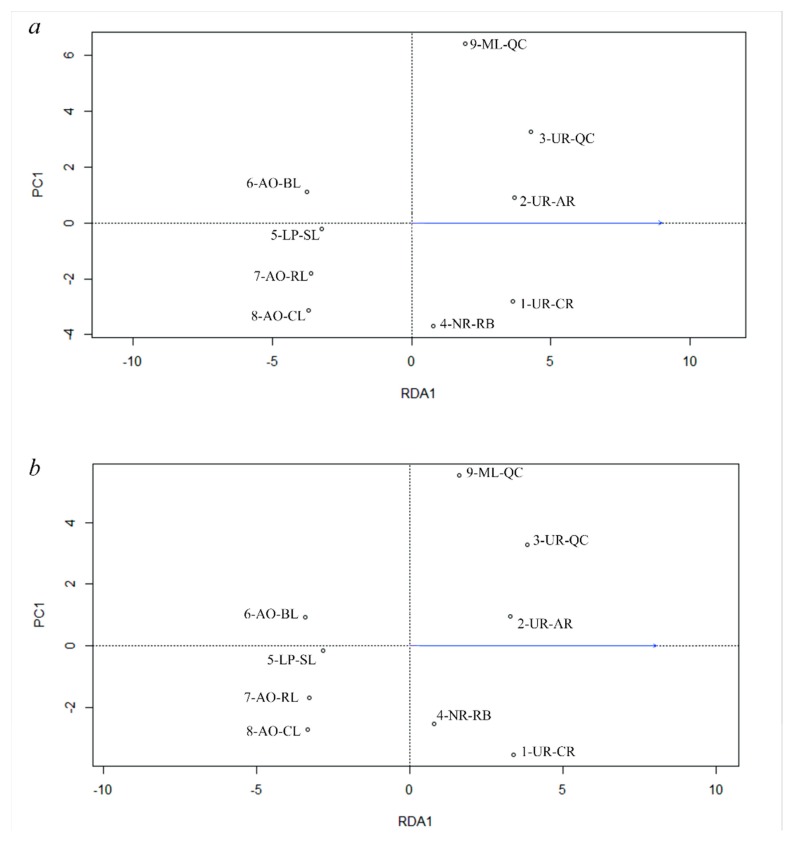

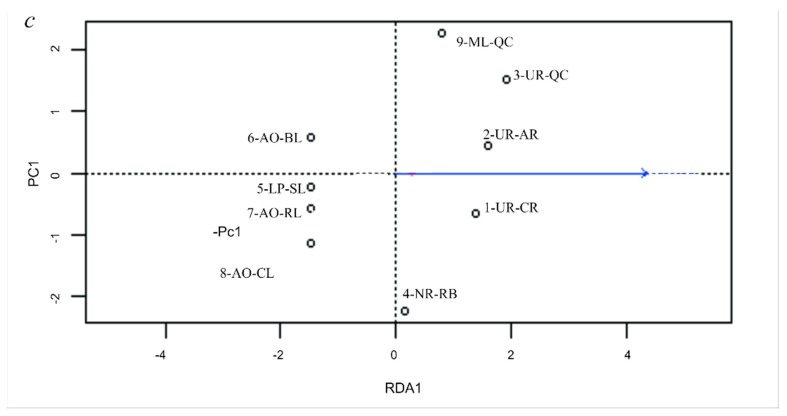
Redundancy analyses (RDA) of 9 locality samples in *R. quelen* based on Total dataset (14,820 loci) (**a**), Neutral dataset (13,213 loci) (**b**), or Outlier dataset (72 loci) (**c**) using the “maximum temperature of warmest month,” which was the most significant variable in all cases.

Selection analyses of Total, Neutral and Outlier datasets resulted concordant, and the “maximum temperature of the warmest month” was the most significant variable explaining genetic differences (*p* ˂ 0.01; Table 7). Redundancy analyses and Analysis of variance (ANOVA) results of the three datasets are shown in Table 6. The maximum temperature of the coldest month was the variable that better explained the genetic differences from the Outlier dataset (Table 7). Plot of the three RDA (Figure 5) showed that “maximum temperature of the warmest month” was associated with genetic differentiation following a South-North geographical pattern. 

### 3.6. Evolutionary History of Population

**Figure 6:**
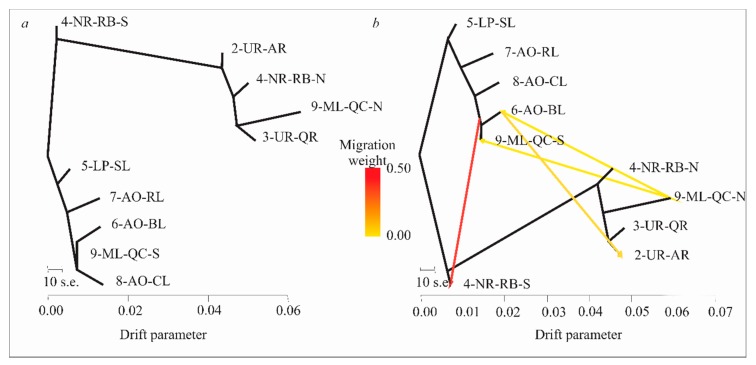
Population trees inferred by TREEMIX of eight locality samples in *Rhamdia quelen* (**a**) without or (**b**) with four significant migration events (*p*-value < 0.005). Color of admixture arrows according to migration weight.

The evolutionary history of localities without migration (Figure 6a) showed a close relationship among the four coastal lagoons (5-LP-SL, 6-AO-BL, 7-AO-RL and 8-AO-CL), while the Merin lagoon belonged to the South Cluster (9-ML-QC-S). Additionally, two groups from the Negro River basin (4-NR-RB-S and 4-NR-RB-N) showed a basal position in relation to all remaining localities from the South and North Clusters, suggesting an ancestral genomic background. The Uruguay River localities (2-UR-AR and 3-UR-QR) were related to 4-NR-RB-N and 9-ML-QC-N. Among the number of migration events tested, four were the most significant (*p* ˂ 0.005). The topology including these four migration events (Figure 6b) was similar to that with no migration events. The four migration events would have occurred as follows: from 6-AO-BL to 2-UR-AR; from 9-ML-QC-N to 9-ML-QC-S; from 9-ML-QC-N to 6-AO-BL; and from a common ancestor of 6-AO-BL and 9-ML-QC-S to 4-NR-RB-S. 

## 4. Discussion

This is the first study where the genetic diversity and structure pattern of *R. quelen* is addressed with thousands of genome-wide distributed SNP loci. The loci were genotyped using 2bRAD-seq methodology and data were integrated with mtDNA lineages information. In a scenario of hybridization among highly divergent mitochondrial lineages (*Rq2*, *Rq4* and *Rq6*) that coexist in the Uruguay River (*Rq2*, *Rq4* and *Rq6*), Negro River and Merin Lagoon basins (*Rq4* and *Rq6*), the study of genomic differentiation becomes crucial to understand *R. quelen* population genetics history. Here, we provide evidence of a population structure characterized by two major clusters differentiated by latitude as well as by its demographic history. We identified and annotated several loci putatively under selection. Finally, we unraveled the underlying substructure of populations.

### 4.1. Wide Distribution of Interbred mtDNA Lineages

Previous studies in *R. quelen* using *cytb* and *COI* mitochondrial markers arrived at important evolutionary conclusions in the Neotropical region, but a comparative analysis between mtDNA lineages reported separately for both markers was lacking in the literature [5,6,9,10]. The studied area is inhabited by fish with three *cytb* mtDNA lineages that coexist in some basins and that would have diverged in allopatry 5.94–4.55 million years ago (MYA) [9]. According to Ríos et al. [14], *Rq2* and *Rq4*+*Rq6* would belong to different clades, A and C, respectively. Clade A (*Rq2*) would have diverged in the Amazon Basin and would have spread to LP-PM-AO. Clade C (*Rq4* and *Rq6*) would have diverged within LP-PM-AO, which is consistent with marine transgressions in this region [14]. *Rq*2 individuals here analyzed showed the same *COI* mtDNA lineage reported for specimens from Miranda River [15] (Supplementary Files SIII and SVI), located far north from the La Plata Basin, close to the Amazon Basin. This is in accordance with Ríos et al. [14], who proposed the origin of *Rq2* in the Amazon Basin. The interchange of biota between the Amazon and La Plata rivers (specifically through the Paraguay basin) was previously proposed by Carvalho and Albert [76]. Furthermore, phylogeographic analyses of *Serrasalmus* and *Pygocentrus* genera (Neotropical piranhas) [77] as well as *Jubiaba acanthogaster* (Eigenmann, 1911) (Characidae family) [78], supported the occurrence of biota exchange by dispersal between the Amazon and La Plata basins. Our *COI* results showed that the *Rq4* mtDNA lineage not only inhabits the lower Uruguay River, Merin Lagoon and Negro River basins, but it is also present in the upper Uruguay and Parana River basins. Rq6, the most abundant *cytb* mtDNA lineage in the Southern region of LP-PM-AO [14], was clustered within the same *COI* mtDNA lineage as the specimens from the La Pampa Plain [37], which is at the far southwest of LP-PM-AO. However, we also found individuals from the Parana River [37] as well as hatchery individuals from Santa Catarina [4] that belong to the same *Rq6 COI* mtDNA lineage. The wide distribution of Rq6 could be explained by the exchange among basins as a consequence of low topographic relief, as proposed by Albert and Reis [3]. Alternatively, Rq6 distribution could be explained by dispersion associated with reclamation of habitat following glacial periods [6]. Surprisingly, *Rhamdia branneri* (Haseman, 1911) from the Iguaçu Basin [10,11] also belongs to the same *Rq6 COI* lineage. *R. branneri* is considered endemic of the Iguaçu River in the far northeast of LP-PM-AO [10,79]. These results would expand the distribution area of *Rq6* and warn about the need to review the taxonomic status of *R. branneri*. Moreover, *R. branneri* could hybridize with *R. quelen*, not only in hatcheries, as proposed by Scaranto et al. [11], but also in the wild, in accordance to Ríos et al. [17].

### 4.2. Genomic Pattern of Differentiation

Genomic differentiation was higher among the Northern samples than among the Southern ones, while central samples (4-NR-RB and 9-ML-QC) were genetically closer to the South samples (Figure 2a–c, Table 4, Table 5 and Table 6). These results agree with Ríos et al. [17], who suggested a microsatellite-based genetic structure pattern according to a North-South and East-West distribution. The low pairwise F_ST_ values among 1-UR-CR, 2-UR-AR and 3-UR-QR could be due to their common origin in the Uruguay River basin. Total and Neutral pairwise F_ST_ analysis recovered significant differentiation among coastal lagoons as previously reported based on microsatellite loci (Ríos et al. [17]: 0.125–0.216; Neutral SNPs dataset: 0.144–0.202, Table 5), indicative of basin isolation and genetic drift-driven differentiation. However, pairwise F_ST_ of Outlier loci putatively under selection revealed much higher structuring among samples from the Northern and Southern basins (F_ST_ mean: 0.952) and lack of structuring among coastal lagoons (F_ST_ = 0.000). Bayesian Structure analyses of the three datasets were similar and revealed the presence of two clusters. Like DAPC, the distribution of these clusters follows a North-South pattern. A North Cluster was detected in the Northern basins (Uruguay River basin (UR): 1-UR-CR), 2-UR-AR and 3-UR-QR river basins); the second one, the South Cluster, inhabits the Southern basins (La Plata Basin (LP): 5-LP-SL coastal lagoon; and coastal lagoons draining to Atlantic Ocean (AO): 6-AO-BL; 7-AO-RL and 8-AO-CL). The two highly divergent clusters were detected in the Negro River (4-NR-RB) and in the Merin Lagoon (9-ML-QC) basins. Latitudinal differentiation associated with water temperature has also been reported in the Chinese Sea Bass (*Lateolabrax maculatus* (Cuvier, 1828)) by RAD-seq analysis on populations of the continental marginal seas along the Chinese coast [21]. Furthermore, a parallel pattern of genomic divergence along a latitudinal gradient was found in sunflower (Helianthus genus) studies [80]. Genomic analyses of the Atlantic salmon (*Salmo salar* Linnaeus, 1758) also showed clinal SNP variation associated with latitude in North Atlantic Ocean populations [81]. 

According to the analyses of environmental variables, the higher latitudinal differentiation associated with outliers could be explained by temperature, specifically, the maximum temperature of the warmest month. Both the North and South clusters coexist in central basins, i.e., 4-NR-RB and 9-ML-QC, which is in accordance with absence of isolation by distance and could be the result of secondary contact. The pairwise F_ST_ analysis based on a strict set of 73 candidate loci under selection recovered much higher differentiation values among the Northern and Southern locality samples (on average, 0.939) than those derived from the Neutral (0.336) and Total (0.527) datasets. This could indicate that a strong divergent selection is acting over these genomic regions where outlier loci are located, and consequently, differentiation significantly increases along the genome background. According to Fowler et al. [82], temperature has various effects on the life-history stages of fish. Thus, Bradbury et al. [83] identified in the Atlantic cod (*Gadus morhua* Linnaeus, 1758) parallel adaptive evolution associated with temperature clines. Conversely, the Outlier dataset unraveled genetic similarity among the four coastal lagoons studied with common SNP variants fixed at each locus. Because these coastal lagoons would have originated recently (˂ 7000 years ago [84]), selection could be acting over an ancestral population in this area that could have taken refuge and survived the marine transgressions. The hypothesis of refuge areas in the Neotropical region was previously proposed [1,85]. Strong selection and genetic drift for small isolated populations as suggested by outlier and neutral loci, respectively, could explain the low genetic diversity evidenced in coastal lagoons samples and the detection of recent bottleneck events by Ríos et al. [12] in these localities. In this sense, locus 67564_7 is closely linked to the *scn8a* gene (sodium channel protein type 8 subunit alpha-like), which is involved in osmoregulation and in a possible adaptation through ion homeostasis in plasma in a changing external salinity environment [86,87]. The fact that *scn8a* is fixed for the same allele in all coastal lagoons might be associated with adaptation in lacustrine populations (5-LP-SL, 6-AO-BL, 7-AO-RL and 8-AO-CL), a hypothesis that should be further investigated at a functional level. Evidence of adaptation to different salinities has been documented for several fish species through genes involved mainly in osmoregulation [23,88,89]. 

The pattern of genetic structure observed revealed strongly differentiated North and South clusters that would represent major lineages of the species. The past migration events evidenced by TREEMIX between locality samples from the North and South clusters would point to a scenario of divergence with gene flow/introgression. These migration events between genomic clusters might be associated with ancestral mtDNA lineage hybridization (*Rq4* and *Rq6*) evidenced by Ríos et al. [17]. Deep genomic divergence with gene flow has been also reported by Feder et al. [27], and accumulation of genomic divergence between major lineages within species [90], either along the whole genome (e.g., *Heliconius* spp. butterflies [91], *Helianthus* spp. sunflowers [92] and *Populus* spp. poplar trees [93]), or limited to specific genome regions (e.g., *Gasterosteus* spp. three-spines stickleback [94,95]; Baltic cod, Berg et al. [88]). Some outlier loci were anchored in close proximity in two small regions of the *I. punctatus* genome (64979_10 and 15866_28, separated by 147 Kb; 49857_34, 9230_26 and 449_34, by 317 Kb; Table 2), suggesting synteny and possible evidence of genomic stretches of differentiation. Genome regions of deep intraspecific divergence can be associated with inversions [96] (e.g., *Littorina saxatilis* (Olivi, 1792) [97], three-spined stickleback [98] and *G. morhua* [99]) or with the vicinity of centromeres [90]. However, the lack of a *R. quelen* reference genome does not allow analyzing the genomic architecture of the divergence process in this species. Another interesting outlier locus 33517_8 in our study was anchored to sperm acrosome membrane-associated protein 6 gene (*spaca6*), which is involved in mediating sperm fusion through binding to an egg membrane receptor [100]. This gene would deserve to be further studied, since closely related species to *R. quelen* lack acrosomal structures [101,102]; thus, it could play a reproductive role in this species, an issue of major interest putatively associated with the divergence between genomic clusters detected in this study. This hypothesis would be in agreement with Bird et al. [103], who suggest that evolutionary studies at the early stages of lineage divergence could reveal details of the mechanisms that generate reproductive isolation. Further mating and functional studies are necessary to investigate reproductive isolation mediated by gamete recognition proteins between individuals of the North and South genomic clusters. Similarly, the outlier loci putatively under selection evidenced in this study should be validated in future studies. 

### 4.3. Genetic Substructure within Genomic Clusters

Based on 10 microsatellite loci, Ríos et al. [17] found that the recent structure in *R*. *quelen* follows a geographical pattern and is composed of different genetically divergent populations. In this study, a population substructure pattern was disclosed based on the Total and Neutral datasets. Specifically, the Total and Neutral datasets evidenced a complex substructure (Figure 4 and Supplementary File SX: Figure S1) with more than 10 minor clusters. This pattern of divergence could be explained by genetic drift and local adaptation. The Neutral substructure clustering (Figure 3e, K = 5) confirmed a close relationship between 5-LP-SL and 7-AO-RL, suggesting that individuals of both basins might have been connected during the last glaciation (14,000–6000 years ago). Local differentiated subgroups were also observed in 4-NR-RB-S and 9-ML-QC-N samples. Moreover, the Uruguay River basin together with specimens of the Negro River basin assigned to the North cluster (4-NR-RB-N) would constitute a clear differentiated subgroup. All these subclades are consistent with the divergent populations proposed by Ríos et al. [17] using microsatellite loci. In the present study, the subgroup comprising 6-AO-BL, 8-AO-CL and 9-ML-QC-S appeared highly differenced but with low intra-subgroup differentiation, despite the fact that a significant differentiation had been reported with microsatellites by Ríos et al. [17]. Hatchery sample analyses show a diversity in origin, with some individuals closely related to the North cluster and others to the South cluster. This fact should alert about seed trade and propagation because they could be artificial hybrids. Irresponsible farming and accidents might lead to escapees and have a negative impact on *R. quelen* natural populations. All these data suggest that differentiation within subgroups is nested into the two major genomic clusters detected in this study (North and South clusters). More recent population differentiation within each genomic cluster may have been the result of geographic isolation and genetic drift. The presence of fish assigned to either the North or South genomic clusters in the Negro River and Merin Lagoon basins, suggests the absence of recent introgression, although caution should be exercised due to the limited sample size. Ríos et al. [17], based on a higher sample microsatellite survey at these localities, also found two divergent population clusters in sympatry in the Negro River, due to either homing behavior or divergent selection. In this sense, new field surveys are recommended to increase the sample size in localities where more than one genomic cluster was detected, followed by nuclear and mitochondrial marker analyses in order to search for genomic signals of linkage disequilibrium as a result of hybridization. Our results allowed identifying genomic footprints of divergent selection between the North and South clusters of *R. quelen* in the LP-MP-AO basin system. Genomic differentiation associated with homing behavior cannot be ruled out, although this behavior has never been described for the *Rhamdia* genus.

## 5. Conclusions

Population genomic analysis has identified high latitudinal differentiation between two clusters of *R. quelen* (North and South clusters) from the LP-PM-AO basin system that may be driven by divergent selection. This latitudinal structure pattern could be explained by temperature gradients, which may affect adaptive variation at specific genomic regions. The two major genomic clusters detected show restricted gene flow and represent valuable adaptive variability for the species. In particular, the coastal lagoons should be considered for conservation and management purposes, given the potential population adaptation to the lacustrine environment. Novel insights into the phylogeographic composition of mtDNA lineages within LP-PM-AO were found, and *Rq2*, *Rq4* and *Rq6* appeared to be more widely distributed within that basin system than previously suggested. 

## Figures and Tables

**Table 1:** Number of specimens analyzed per sampling site using RAD-seq and mitochondrial markers (*cytb* and *COI*).

Sampling Site	N RAD-seq	N *cytb*	N *COI*
1-UR-CR	2	7 (1)	2
2-UR-AR	7	11 (0)	3
3-UR-QR	4	6 (0)	-
4-NR-RB	10	23 (0)	1
5-LP-SL	10	49 (3)	-
6-AO-BL	9	19 (9)	-
7-AO-RL	9	17 (1)	1
8-AO-CL	10	36 (6)	1
9-ML-QC	10	16 (2)	1
10-VC	3	12 (0)	3

N RAD-seq: specimens genotyped by RAD-seq; N *cytb*: total number of specimens analyzed using cytochrome b marker; the number of individuals sequenced in the present study is shown between parentheses; N *COI*: individuals sequenced in this study for cytochrome c oxidase subunit I.

**Table 2:** Annotation and gene mining of the most consistent *Rhamdia quelen* Outlier dataset that results from comparing Northern and Southern basins.

SNP	*Homologous Sequence* (*E*-Value)	Gene	Gene Mining	Biological Processes of Gene Mining
64979_10	*Ictalurus punctatus* genome Chr 5: 13,660,451–13,660,486 (9 e^−10^)	Unannotated sequence	zbtb34 angptl2 ralgps1	RNA polymerase II regulatory region; DNA-binding transcription repressor immune; angiogenesis; hematopoiesis; cell differentiation; positive regulation of Notch signaling pathway; signal transduction (GTPase, Ral protein); cell differentiation; anatomical structure and morphogenesis; embryo development; cellular nitrogen compound metabolic process
15866_28	*I. punctatus* genome Chr 5: 13,807,353–13,807,388 (5 e^−5^)	ralgps1		
49857_34	*I. punctatus* genome Chr 8: 7,712,981–7,713,013 (2 e^−10^)	kdrl	kctd8 lnx2 loxl3 trmt112 atg2a itpkb tc1a seipin-like cdx1; slc25a6 ant3ANT3	Protein binding; scavenger receptor activity; copper ion binding; oxidoreductase activity; protein heterodimerization activity; kinase activity; DNA binding; protein kinase and protein tyrosine kinase activity; ATP binding and transmembrane transporter activity; DNA binding; regulation of transcription, DNA-templated; multicellular organism development
9230_26	XM_027135514.1 *I. punctatus* genome Chr 8: 7,757,023–7,757,984 (7 e^−5^)	chic1		
449_34	XM_027165565.1 *I. punctatus* genome Chr 8: 8,029,587–8,031,148 (4 e^−7^)	fbxw2		

SNP: SNP locus name; Homologous sequence and *E*-value: Significant BLAST hits in the genome of *I. punctatus* (Chr, chromosome and start-end position in pb) and/or GenBank (Accession Number); Gene: annotated gene to which RAD-tag is anchored; Gene mining: genes which were found close to the syntenic SNPs in the *I. punctatus* genome, and summary of associated biological processes.

**Table 3:** Genetic diversity of *Rhamdia quelen* locality samples based on RAD-seq genotypes.

Locality	N	Na	H_E_	F_IS_
2-UR-AR	7	1.47	0.276 (0.163)	−0.018
4-NR-RB	10	1.60	0.283 (0.158)	0.285 *
5-LP-SL	10	1.36	0.249 (0.148)	0.013
6-AO-BL	9	1.20	0.328 (0.139)	−0.015
7-AO-RL	9	1.22	0.268 (0.154)	0.010
8-AO-CL	10	1.24	0.277 (0.151)	0.000
9-ML-QC	10	1.37	0.324 (0.154)	0.486 *

Genetic diversity was analyzed in samples comprising a minimum of 7 individuals. N: sample size; Na: mean number of alleles per locus; H_E_: expected heterozygosity (Standard Deviation); F_IS_: intrapopulation fixation index. *F_IS_: significant values after sequential Bonferroni correction (*p* < 0.007).

**Table 4:** Pairwise F_ST_ values between localities based on the Total dataset of 17,559 SNP loci. Significant FST values are highlighted in bold type (*p* ˂ 0.05).

			1-UR-CR	2-UR-AR	3-UR-UQ	4-NR-RB	9-ML-QC	5-LP-SL	6-AO-BL	7-AO-RL	8-AO-CL	10-VC
Riverine	North	1-UR-CR	0.000									
		2-UR-AR	0.036	0.000								
		3-UR-UQ	**0.075**	**0.040**	0.000							
	Centre	4-NR-RB	0.077	0.107	**0.143**	0.000						
		9-ML-QC	0.121	**0.098**	**0.151**	**0.098**	0.000					
Coastal lagoon	South	5-LP-SL	**0.458**	**0.447**	**0.519**	**0.210**	**0.383**	0.000				
		6-AO-BL	**0.537**	**0.496**	**0.582**	**0.261**	**0.427**	**0.196**	0.000			
		7-AO-RL	**0.537**	**0.497**	**0.581**	**0.265**	**0.432**	**0.174**	**0.246**	0.000		
		8-AO-CL	**0.555**	**0.516**	**0.597**	**0.280**	**0.445**	**0.209**	**0.213**	**0.250**	0.000	
Hatchery		10-VC	0.189	**0.292**	**0.342**	**0.116**	0.269	**0.163**	**0.276**	**0.270**	**0.307**	0.000

**Table 5:** Pairwise F_ST_ values based on 15,584 SNP neutral loci dataset between localities. Significant FST values are highlighted in bold type (*p* ˂ 0.05).

			1-UR-CR	2-UR-AR	3-UR-UQ	4-NR-RB	9-ML-QC	5-LP-SL	6-AO-BL	7-AO-RL	8-AO-CL	10-VC
Riverine	North	1-UR-CR	0.000									
		2-UR-AR	0.020	0.000								
		3-UR-UQ	**0.045**	**0.032**	0.000							
	Centre	4-NR-RB	0.051	**0.058**	**0.076**	0.000						
		9-ML-QC	0.126	**0.081**	**0.129**	**0.081**	0.000					
Coastal lagoon	South	5-LP-SL	**0.270**	**0.258**	**0.307**	**0.138**	**0.252**	0.000				
		6-AO-BL	**0.352**	**0.309**	**0.380**	**0.186**	**0.300**	**0.170**	0.000			
		7-AO-RL	**0.358**	**0.310**	**0.379**	**0.191**	**0.308**	**0.144**	**0.207**	0.000		
		8-AO-CL	**0.378**	**0.334**	**0.399**	**0.203**	**0.316**	**0.173**	**0.185**	**0.202**	0.000	
Hatchery		10-VC	0.065	**0.173**	**0.198**	**0.110**	**0.221**	**0.142**	**0.239**	**0.240**	**0.269**	0.000

**Table 6:** Pairwise F_ST_ values between localities based on the dataset of 73 SNP Outlier loci. Significant FST values are highlighted in bold type (*p* ˂ 0.05).

			1-UR-CR	2-UR-AR	3-UR-UQ	4-NR-RB	9-ML-QC	5-LP-SL	6-AO-BL	7-AO-RL	8-AO-CL	10-VC
Riverine	North	1-UR-CR	0.000									
		2-UR-AR	0.120	0.000								
		3-UR-UQ	0.372	−0.001	0.000							
	Centre	4-NR-RB	0.152	**0.307**	**0.390**	0.000						
		9-ML-QC	0.035	0.149	0.239	0.048	0.000					
Coastal lagoon	South	5-LP-SL	**0.947**	**0.916**	**0.999**	**0.454**	**0.668**	0.000				
		6-AO-BL	**0.942**	**0.910**	**0.999**	**0.437**	**0.655**	0.000	0.000			
		7-AO-RL	**0.942**	**0.911**	**0.999**	**0.439**	**0.655**	0.000	0.000	0.000		
		8-AO-CL	**0.948**	**0.917**	**0.999**	**0.462**	**0.673**	0.000	0.000	0.000	0.000	
Hatchery		10-VC	0.647	**0.750**	**0.883**	0.156	0.404	**0.389**	**0.353**	**0.362**	**0.401**	0.000

**Table 7:** Redundancy analyses (RDA) results for Total, Neutral and Outlier datasets in nine locality samples of *Rhamdia quelen*.

		Total	Neutral	Outliers
RDA	*p*-Value	0.007	0.003	0.006
	r^2^	0.576	0.517	0.827
	Adjusted r^2^	0.515	0.448	0.802

## Supplementary Materials

The following are available online at https://www.mdpi.com/2073-4425/11/1/109/s1, Files SI: Geographic distribution of *Rhamdia quelen* specimens studied, Files SII: Cytochrome b dataset, Files SIII: Cytochrome c oxidase subunit I dataset, Files SIV: Analyses of outlier loci subjected to selection analyzed by Bayescan and Arlequin, Files SV: Values of environmental variables examined for the sampled localities, Files SVI: Phylogenetic analysis of the genus *Rhamdia* in the Neotropical region, Files SVII: Details of the 17,559 SNPs loci and their specific RAD-tag sequences, Files SVIII: Annotation of the *Rhamdia quelen* Outlier dataset, Files SIX: Structure Harvester results, Files SX: *Rhamdia quelen* fineRADstructure plot based on Neutral and Outlier loci.
