# Supplementary material for: Population Genomics in Rhamdia quelen (Heptapteridae, Siluriformes) Reveals Deep Divergence and Adaptation in the Neotropical Region
Source: Genes (Basel). 2020 Jan 17;11(1):109. doi: 10.3390/genes11010109 (PMC7017130; doi:10.3390/genes11010109)
Supplement: Supplementary file 1 [file genes-11-00109-s001.zip › Supplementary File SI.docx]

**Table SI 1.** Geographic distribution of *Rhamdia quelen* specimens studied.

| **Sampling site** | **Environment** | **Basin** | **Sample code ^a,b^** | **Mitochondrial lineage** | **GenBank Accession number *cyt b*^c^** |
| --- | --- | --- | --- | --- | --- |
| 01-UR-CR | Riverine | Uruguay river | UC2197 | *Rq2* | MK511219* |
|  |  |  | UC2217 | *Rq4* | MH669077 |
| 02-UR-AR | Riverine |  | UA2165 | *Rq4* | KX379753 |
|  |  |  | UA2167 | *Rq4* | KX379755 |
|  |  |  | UA2168 | *Rq4* | KX379756 |
|  |  |  | UA2192 | *Rq4* | MH669078 |
|  |  |  | UA2193 | *Rq4* | MH669079 |
|  |  |  | UA2194 | *Rq4* | MH669080 |
|  |  |  | UA2196 | *Rq4* | MH669081 |
| 03-UR-UQ | Riverine |  | UQ2164 | *Rq6* | KX379752 |
|  |  |  | UQ2176 | *Rq6* | KX379761 |
|  |  |  | UQ2178 | *Rq4* | KX379763 |
|  |  |  | UQ2190 | *Rq4* | MH669083 |
| 04-NR-RB | Riverine | Negro river | NSG1789^a^ | *Rq4* | KP798737 |
|  |  |  | NSG2288^a^ | *Rq4* | MH669086 |
|  |  |  | NSG2290^a^ | *Rq4* | MH669099 |
|  |  |  | NSG2294^a^ | *Rq6* | MH669104 |
|  |  |  | NSG2295 ^a^ | *Rq4* | MH669100 |
|  |  |  | NSG2148 ^b^ | *Rq6* | KX379742 |
|  |  |  | NSG2289 ^b^ | *Rq6* | MH669098 |
|  |  |  | NSG2291 ^b^ | *Rq6* | MH669101 |
|  |  |  | NSG2292 ^b^ | *Rq6* | MH669102 |
|  |  |  | NSG2293 ^b^ | *Rq6* | MH669103 |
| 05-LP-SL | Coastal lagoon | La Plata basin | LPS1686 | *Rq6* | KP798786 |
|  |  |  | LPS1687 | *Rq6* | KP798785 |
|  |  |  | LPS1689 | *Rq6* | KP798783 |
|  |  |  | LPS1694 | *Rq6* | KP798778 |
|  |  |  | LPS1695 | *Rq6* | KP798777 |
|  |  |  | LPS1696 | *Rq6* | KP798776 |
|  |  |  | LPS1697 | *Rq6* | MK511196* |
|  |  |  | LPS1994 | *Rq6* | KP798643 |
|  |  |  | LPS2200 | *Rq6* | MK511197* |
|  |  |  | LPS2201 | *Rq6* | MK511198* |
| 06-AO-BL | Coastal lagoon | Atlantic Ocean SW | OLB2268 | *Rq6* | MK511199* |
|  |  |  | OLB2269 | *Rq6* | MK511200* |
|  |  |  | OLB2271 | *Rq6* | MK511201* |
|  |  |  | OLB2272 | *Rq6* | MK511202* |
|  |  |  | OLB2273 | *Rq6* | MK511203* |
|  |  |  | OLB2274 | *Rq6* | MK511204* |
|  |  |  | OLB2275 | *Rq6* | MK511205* |
|  |  |  | OLB2276 | *Rq6* | MK511206* |
|  |  |  | OLB2277 | *Rq6* | MK511207* |
| 07-AO-RL | Coastal lagoon |  | OR1837 | *Rq6* | KP798715 |
|  |  |  | OR1839 | *Rq6* | KP798713 |
|  |  |  | OR1840 | *Rq6* | KP798712 |
|  |  |  | OR1841 | *Rq6* | KP798711 |
|  |  |  | OR1843 | *Rq6* | KP798709 |
|  |  |  | OR1845 | *Rq6* | KP798707 |
|  |  |  | OR1847 | *Rq6* | KP798705 |
|  |  |  | OR1848 | *Rq6* | KP798704 |
|  |  |  | OR2280 | *Rq6* | MK511214* |
| 08-AO-CL | Coastal lagoon |  | OC1707 | *Rq6* | KP798769 |
|  |  |  | OC1818 | *Rq6* | KP798732 |
|  |  |  | OC1820 | *Rq6* | KP798730 |
|  |  |  | OC1822 | *Rq6* | KP798729 |
|  |  |  | OC2281 | *Rq6* | MK511208* |
|  |  |  | OC2282 | *Rq6* | MK511209* |
|  |  |  | OC2283 | *Rq6* | MK511210* |
|  |  |  | OC2284 | *Rq6* | MK511211* |
|  |  |  | OC2285 | *Rq6* | MK511212* |
|  |  |  | OC2286 | *Rq6* | MK511213* |
| 09-ML-QC | Riverine | Merin lagoon | M2175 ^a^ | *Rq6* | KX379760 |
|  |  |  | M2232 ^a^ | *Rq6* | MH669112 |
|  |  |  | M2233 ^a^ | *Rq6* | MH669113 |
|  |  |  | M2234 ^a^ | *Rq4* | MK511194* |
|  |  |  | M2235 ^a^ | *Rq6* | MH669115 |
|  |  |  | M2236 ^a^ | *Rq6* | MH669116 |
|  |  |  | M2237 ^a^ | *Rq4* | MK511195* |
|  |  |  | M2162 ^b^ | *Rq6* | KX379750 |
|  |  |  | M2163 ^b^ | *Rq6* | KX379751 |
|  |  |  | M2231 ^b^ | *Rq6* | MH669111 |
| 10-VC |  | Hatchery | UVC1963 | *Rq2* | KP798676 |
|  |  |  | UVC1969 | *Rq2* | KP798673 |
|  |  |  | UVC2261 | *Rq2* | MH669121 |

^a,b^ Assignment to ^a^ North (N) and ^b^ South (S) clusters in the Structure analysis.

^c^ GenBank accession numbers of novel sequences are marked with an asterisk.
