# Supplementary material for: Population Genomics in Rhamdia quelen (Heptapteridae, Siluriformes) Reveals Deep Divergence and Adaptation in the Neotropical Region
Source: Genes (Basel). 2020 Jan 17;11(1):109. doi: 10.3390/genes11010109 (PMC7017130; doi:10.3390/genes11010109)
Supplement: Supplementary file 1 [file genes-11-00109-s001.zip › Supplementary File SII.docx]

**Table SII 1.** Cytochrome b dataset and mitochondrial lineages according to Ríos et al. [14].

| Genbank accession number cytb | Basin | Species | Cyt b lineages |
| --- | --- | --- | --- |
| KP798762 | Uruguay River | *R. quelen* | Rq2 |
| MH669076 | Uruguay River | *R. quelen* | Rq2 |
| KP798651 | Uruguay River | *R. quelen* | Rq4 |
| KX379745 | Uruguay River | *R. quelen* | Rq4 |
| MH669077 | Uruguay River | *R. quelen* | Rq4 |
| KP798763 | Uruguay River | *R. quelen* | Rq6 |
| KX379744 | Uruguay River | *R. quelen* | Rq6 |
| KX379753 | Uruguay River | *R. quelen* | Rq4 |
| KX379754 | Uruguay River | *R. quelen* | Rq4 |
| KX379755 | Uruguay River | *R. quelen* | Rq4 |
| KX379756 | Uruguay River | *R. quelen* | Rq4 |
| KX379757 | Uruguay River | *R. quelen* | Rq4 |
| KX379758 | Uruguay River | *R. quelen* | Rq4 |
| MH669078 | Uruguay River | *R. quelen* | Rq4 |
| MH669079 | Uruguay River | *R. quelen* | Rq4 |
| MH669080 | Uruguay River | *R. quelen* | Rq4 |
| MH669081 | Uruguay River | *R. quelen* | Rq4 |
| MH669082 | Uruguay River | *R. quelen* | Rq6 |
| KP798663 | Uruguay River | *R. quelen* | Rq4 |
| KX379762 | Uruguay River | *R. quelen* | Rq4 |
| KX379763 | Uruguay River | *R. quelen* | Rq4 |
| MH669083 | Uruguay River | *R. quelen* | Rq4 |
| KX379752 | Uruguay River | *R. quelen* | Rq6 |
| KX379761 | Uruguay River | *R. quelen* | Rq6 |
| KP798646 | Negro River | *R. quelen* | Rq6 |
| KP798647 | Negro River | *R. quelen* | Rq6 |
| MH669084 | Negro River | *R. quelen* | Rq6 |
| MH669085 | Negro River | *R. quelen* | Rq4 |
| MH669122 | Negro River | *R. quelen* | Rq4 |
| KX379748 | Negro River | *R. quelen* | Rq4 |
| MH669086 | Negro River | *R. quelen* | Rq4 |
| MH669087 | Negro River | *R. quelen* | Rq6 |
| KP798743 | Negro River | *R. quelen* | Rq6 |
| MH669088 | Negro River | *R. quelen* | Rq6 |
| KP798742 | Negro River | *R. quelen* | Rq6 |
| MH669089 | Negro River | *R. quelen* | Rq6 |
| MH669090 | Negro River | *R. quelen* | Rq6 |
| MH669091 | Negro River | *R. quelen* | Rq6 |
| KP798652 | Negro River | *R. quelen* | Rq6 |
| KP798649 | Negro River | *R. quelen* | Rq6 |
| KP798650 | Negro River | *R. quelen* | Rq6 |
| MH669092 | Negro River | *R. quelen* | Rq6 |
| MH669093 | Negro River | *R. quelen* | Rq6 |
| MH669094 | Negro River | *R. quelen* | Rq6 |
| KX379747 | Negro River | *R. quelen* | Rq6 |
| MH669095 | Negro River | *R. quelen* | Rq6 |
| MH669096 | Negro River | *R. quelen* | Rq6 |
| MH669097 | Negro River | *R. quelen* | Rq6 |
| KX379749 | Negro River | *R. quelen* | Rq6 |
| MH669098 | Negro River | *R. quelen* | Rq6 |
| KP798737 | Negro River | *R. quelen* | Rq4 |
| MH669099 | Negro River | *R. quelen* | Rq4 |
| KX379742 | Negro River | *R. quelen* | Rq6 |
| MH669100 | Negro River | *R. quelen* | Rq4 |
| KP798739 | Negro River | *R. quelen* | Rq6 |
| KP798738 | Negro River | *R. quelen* | Rq6 |
| MH669101 | Negro River | *R. quelen* | Rq6 |
| MH669102 | Negro River | *R. quelen* | Rq6 |
| MH669103 | Negro River | *R. quelen* | Rq6 |
| MH669104 | Negro River | *R. quelen* | Rq6 |
| KP798759 | La Plata River | *R. quelen* | Rq6 |
| MH669105 | La Plata River | *R. quelen* | Rq6 |
| KP798749 | La Plata River | *R. quelen* | Rq6 |
| KP798748 | La Plata River | *R. quelen* | Rq6 |
| KP798808 | La Plata River | *R. quelen* | Rq6 |
| KP798807 | La Plata River | *R. quelen* | Rq6 |
| KP798806 | La Plata River | *R. quelen* | Rq6 |
| KP798805 | La Plata River | *R. quelen* | Rq6 |
| KP798804 | La Plata River | *R. quelen* | Rq6 |
| KP798803 | La Plata River | *R. quelen* | Rq6 |
| KP798802 | La Plata River | *R. quelen* | Rq6 |
| KP798801 | La Plata River | *R. quelen* | Rq6 |
| KP798800 | La Plata River | *R. quelen* | Rq6 |
| KP798799 | La Plata River | *R. quelen* | Rq6 |
| KP798798 | La Plata River | *R. quelen* | Rq6 |
| KP798797 | La Plata River | *R. quelen* | Rq6 |
| KP798796 | La Plata River | *R. quelen* | Rq6 |
| KP798795 | La Plata River | *R. quelen* | Rq6 |
| KP798794 | La Plata River | *R. quelen* | Rq6 |
| KP798792 | La Plata River | *R. quelen* | Rq6 |
| KP798791 | La Plata River | *R. quelen* | Rq6 |
| KP798790 | La Plata River | *R. quelen* | Rq6 |
| KP798789 | La Plata River | *R. quelen* | Rq6 |
| KP798788 | La Plata River | *R. quelen* | Rq6 |
| KP798787 | La Plata River | *R. quelen* | Rq6 |
| KP798786 | La Plata River | *R. quelen* | Rq6 |
| KP798785 | La Plata River | *R. quelen* | Rq6 |
| KP798784 | La Plata River | *R. quelen* | Rq6 |
| KP798783 | La Plata River | *R. quelen* | Rq6 |
| KP798782 | La Plata River | *R. quelen* | Rq6 |
| KP798781 | La Plata River | *R. quelen* | Rq6 |
| KP798780 | La Plata River | *R. quelen* | Rq6 |
| KP798779 | La Plata River | *R. quelen* | Rq6 |
| KP798778 | La Plata River | *R. quelen* | Rq6 |
| KP798777 | La Plata River | *R. quelen* | Rq6 |
| KP798775 | La Plata River | *R. quelen* | Rq6 |
| KP798774 | La Plata River | *R. quelen* | Rq6 |
| KP798669 | La Plata River | *R. quelen* | Rq6 |
| KP798668 | La Plata River | *R. quelen* | Rq6 |
| KP798667 | La Plata River | *R. quelen* | Rq6 |
| KP798666 | La Plata River | *R. quelen* | Rq6 |
| KP798665 | La Plata River | *R. quelen* | Rq6 |
| KP798664 | La Plata River | *R. quelen* | Rq6 |
| KP798659 | La Plata River | *R. quelen* | Rq6 |
| KP798658 | La Plata River | *R. quelen* | Rq6 |
| KP798657 | La Plata River | *R. quelen* | Rq6 |
| KP798656 | La Plata River | *R. quelen* | Rq6 |
| KP798655 | La Plata River | *R. quelen* | Rq6 |
| KP798643 | La Plata River | *R. quelen* | Rq6 |
| KP798644 | La Plata River | *R. quelen* | Rq6 |
| KP798760 | Atlantic Ocean SW | *R. quelen* | Rq6 |
| KP798761 | Atlantic Ocean SW | *R. quelen* | Rq6 |
| MH669106 | Atlantic Ocean SW | *R. quelen* | Rq6 |
| MH669108 | Atlantic Ocean SW | *R. quelen* | Rq6 |
| MH669109 | Atlantic Ocean SW | *R. quelen* | Rq6 |
| MH669107 | Atlantic Ocean SW | *R. quelen* | Rq6 |
| MH669110 | Atlantic Ocean SW | *R. quelen* | Rq6 |
| KP798746 | Atlantic Ocean SW | *R. quelen* | Rq6 |
| KP798745 | Atlantic Ocean SW | *R. quelen* | Rq6 |
| KP798744 | Atlantic Ocean SW | *R. quelen* | Rq6 |
| KP798715 | Atlantic Ocean SW | *R. quelen* | Rq6 |
| KP798714 | Atlantic Ocean SW | *R. quelen* | Rq6 |
| KP798713 | Atlantic Ocean SW | *R. quelen* | Rq6 |
| KP798712 | Atlantic Ocean SW | *R. quelen* | Rq6 |
| KP798711 | Atlantic Ocean SW | *R. quelen* | Rq6 |
| KP798710 | Atlantic Ocean SW | *R. quelen* | Rq6 |
| KP798709 | Atlantic Ocean SW | *R. quelen* | Rq6 |
| KP798708 | Atlantic Ocean SW | *R. quelen* | Rq6 |
| KP798707 | Atlantic Ocean SW | *R. quelen* | Rq6 |
| KP798706 | Atlantic Ocean SW | *R. quelen* | Rq6 |
| KP798705 | Atlantic Ocean SW | *R. quelen* | Rq6 |
| KP798704 | Atlantic Ocean SW | *R. quelen* | Rq6 |
| KP798688 | Atlantic Ocean SW | *R. quelen* | Rq6 |
| KP798685 | Atlantic Ocean SW | *R. quelen* | Rq6 |
| KP798683 | Atlantic Ocean SW | *R. quelen* | Rq6 |
| KP798681 | Atlantic Ocean SW | *R. quelen* | Rq6 |
| KP798772 | Atlantic Ocean SW | *R. quelen* | Rq6 |
| KP798771 | Atlantic Ocean SW | *R. quelen* | Rq6 |
| KP798770 | Atlantic Ocean SW | *R. quelen* | Rq6 |
| KP798769 | Atlantic Ocean SW | *R. quelen* | Rq6 |
| KP798768 | Atlantic Ocean SW | *R. quelen* | Rq6 |
| KP798767 | Atlantic Ocean SW | *R. quelen* | Rq6 |
| KP798735 | Atlantic Ocean SW | *R. quelen* | Rq6 |
| KP798734 | Atlantic Ocean SW | *R. quelen* | Rq6 |
| KP798733 | Atlantic Ocean SW | *R. quelen* | Rq6 |
| KP798732 | Atlantic Ocean SW | *R. quelen* | Rq6 |
| KP798731 | Atlantic Ocean SW | *R. quelen* | Rq6 |
| KP798730 | Atlantic Ocean SW | *R. quelen* | Rq6 |
| KP798729 | Atlantic Ocean SW | *R. quelen* | Rq6 |
| KP798728 | Atlantic Ocean SW | *R. quelen* | Rq6 |
| KP798727 | Atlantic Ocean SW | *R. quelen* | Rq6 |
| KP798726 | Atlantic Ocean SW | *R. quelen* | Rq6 |
| KP798725 | Atlantic Ocean SW | *R. quelen* | Rq6 |
| KP798724 | Atlantic Ocean SW | *R. quelen* | Rq6 |
| KP798723 | Atlantic Ocean SW | *R. quelen* | Rq6 |
| KP798722 | Atlantic Ocean SW | *R. quelen* | Rq6 |
| KP798719 | Atlantic Ocean SW | *R. quelen* | Rq6 |
| KP798718 | Atlantic Ocean SW | *R. quelen* | Rq6 |
| KP798717 | Atlantic Ocean SW | *R. quelen* | Rq6 |
| KP798703 | Atlantic Ocean SW | *R. quelen* | Rq6 |
| KP798701 | Atlantic Ocean SW | *R. quelen* | Rq6 |
| KP798698 | Atlantic Ocean SW | *R. quelen* | Rq6 |
| KP798697 | Atlantic Ocean SW | *R. quelen* | Rq6 |
| KP798696 | Atlantic Ocean SW | *R. quelen* | Rq6 |
| KP798693 | Atlantic Ocean SW | *R. quelen* | Rq6 |
| KP798691 | Atlantic Ocean SW | *R. quelen* | Rq6 |
| KP798766 | Merin Lagoon | *R. quelen* | Rq6 |
| KP798654 | Merin Lagoon | *R. quelen* | Rq6 |
| KP798653 | Merin Lagoon | *R. quelen* | Rq6 |
| KX379746 | Merin Lagoon | *R. quelen* | Rq4 |
| MH669114 | Merin Lagoon | *R. quelen* | Rq4 |
| MH669117 | Merin Lagoon | *R. quelen* | Rq4 |
| KX379750 | Merin Lagoon | *R. quelen* | Rq6 |
| KX379751 | Merin Lagoon | *R. quelen* | Rq6 |
| KX379759 | Merin Lagoon | *R. quelen* | Rq6 |
| KX379760 | Merin Lagoon | *R. quelen* | Rq6 |
| MH669111 | Merin Lagoon | *R. quelen* | Rq6 |
| MH669112 | Merin Lagoon | *R. quelen* | Rq6 |
| MH669113 | Merin Lagoon | *R. quelen* | Rq6 |
| MH669115 | Merin Lagoon | *R. quelen* | Rq6 |
| MH669116 | Merin Lagoon | *R. quelen* | Rq6 |
| MH669118 | Merin Lagoon | *R. quelen* | Rq6 |
| KP798676 | Hatchery | *R. quelen* | Rq2 |
| KP798673 | Hatchery | *R. quelen* | Rq2 |
| MH669120 | Hatchery | *R. quelen* | Rq2 |
| MH669121 | Hatchery | *R. quelen* | Rq2 |
| KP798679 | Hatchery | *R. quelen* | Rq6 |
| MH669119 | Hatchery | *R. quelen* | Rq6 |
| KP798677 | Hatchery | *R. quelen* | Rq6 |
| KP798675 | Hatchery | *R. quelen* | Rq6 |
| KP798674 | Hatchery | *R. quelen* | Rq6 |
| KX379755 | Hatchery | *R. quelen* | Rq6 |
| KP798671 | Hatchery | *R. quelen* | Rq6 |
| KP798670 | Hatchery | *R. quelen* | Rq6 |
| MK511219* | Uruguay River | *R. quelen* | Rq2 |
| MK511196* | La Plata River | *R. quelen* | Rq6 |
| MK511197* | La Plata River | *R. quelen* | Rq6 |
| MK511198* | La Plata River | *R. quelen* | Rq6 |
| MK511199* | Atlantic Ocean SW | *R. quelen* | Rq6 |
| MK511200* | Atlantic Ocean SW | *R. quelen* | Rq6 |
| MK511201* | Atlantic Ocean SW | *R. quelen* | Rq6 |
| MK511202* | Atlantic Ocean SW | *R. quelen* | Rq6 |
| MK511203* | Atlantic Ocean SW | *R. quelen* | Rq6 |
| MK511204* | Atlantic Ocean SW | *R. quelen* | Rq6 |
| MK511205* | Atlantic Ocean SW | *R. quelen* | Rq6 |
| MK511206* | Atlantic Ocean SW | *R. quelen* | Rq6 |
| MK511207* | Atlantic Ocean SW | *R. quelen* | Rq6 |
| MK511214* | Atlantic Ocean SW | *R. quelen* | Rq6 |
| MK511208* | Atlantic Ocean SW | *R. quelen* | Rq6 |
| MK511209* | Atlantic Ocean SW | *R. quelen* | Rq6 |
| MK511210* | Atlantic Ocean SW | *R. quelen* | Rq6 |
| MK511211* | Atlantic Ocean SW | *R. quelen* | Rq6 |
| MK511212* | Atlantic Ocean SW | *R. quelen* | Rq6 |
| MK511213* | Atlantic Ocean SW | *R. quelen* | Rq6 |
| MK511194* | Merin Lagoon | *R. quelen* | Rq4 |
| MK511195* | Merin Lagoon | *R. quelen* | Rq4 |
| AY036742 | Paraná River | *R. quelen* | Rq2 |
| EF564743 | Paraná River | *R. quelen* | Rq5a |
| AY036743 | Iguaçu River | *R. quelen* | Rq5b |
| AY036744 | Paraná River | *R. quelen* | Rq5b |
| AY036740 | Amazon River | *R. quelen* | Rq3 |
| AY036741 | Amazon River | *R. quelen* | Rq3 |
| AY036739 | Esssequibio River | *R. quelen* | Rq1 |
| DQ119395 | Unknown | *R. quelen* | Rq1 |
| AY036737 | Orinoco River | *R. laukidi* | *R. laukidi* |
| AY036738 | Orinoco River | *R. laukidi* | *R. laukidi* |
| KM489084 | Orinoco River | *R. laukidi* | *R. laukidi* |
| KM489083 | Orinoco River | *R. laukidi* | *R. laukidi* |
| KM489081 | Orinoco River | *R. laukidi* | *R. laukidi* |
| AY036709 |  | *R. laticauda* | *R. laticauda* |
| AY036708 |  | *R. laticauda* | *R. laticauda* |
| AY036672 |  | *R. guatemalensis* | *R. guatemalensis* |
| AY036671 |  | *R. guatemalensis* | *R. guatemalensis* |
| AY036693 |  | *R. wagneri* | *R. wagneri* |
| AY036694 |  | *R. wagneri* | *R. wagneri* |
| AY036725 |  | *R. cabrerae* | *R. cabrerae* |
| AY036726 |  | *R. cabrerae* | *R. cabrerae* |
| AY036735 |  | *R. cinerascens* | *R. cinerascens* |
| AY036736 |  | *R. cinerascens* | *R. cinerascens* |
| AY036734 |  | *R. rogersi* | *R. rogersi* |
| AY036733 |  | *R. rogersi* | *R. rogersi* |
| AY036718 |  | *R. nicaraguensis* | *R. nicaraguensis* |
| AY036719 |  | *R. nicaraguensis* | *R. nicaraguensis* |
| KM489075 |  | *R.saijaensis* | *R.saijaensis* |
| KM489076 |  | *R.saijaensis* | *R.saijaensis* |
| KM489077 |  | *R.saijaensis* | *R.saijaensis* |
