# Supplementary material for: Population Genomics in Rhamdia quelen (Heptapteridae, Siluriformes) Reveals Deep Divergence and Adaptation in the Neotropical Region
Source: Genes (Basel). 2020 Jan 17;11(1):109. doi: 10.3390/genes11010109 (PMC7017130; doi:10.3390/genes11010109)
Supplement: Supplementary file 1 [file genes-11-00109-s001.zip › Supplementary File SIII.docx]

**Table SIII 1.** Cytochrome c oxidase subunit I dataset: sample code of each repository, taxa identification, haplotype, basin, country and mitochondrial lineages.

| Bold Code or GenBank accession number * | Repository | Taxon | Haplotype | Basin, country | Mitochondrial lineages |
| --- | --- | --- | --- | --- | --- |
| BRH002 | BOLD | *Rhamdia branneri* | H01 | Iguaçu River, Brazil | *Rq6* |
| BRH009 | BOLD | *Rhamdia branneri* | H01 | Iguaçu River, Brazil | *Rq6* |
| BRH013 | BOLD | *Rhamdia voulezi* | H02 | Iguaçu River, Brazil | *Basal polytomy* |
| BRH014 | BOLD | *Rhamdia voulezi* | H02 | Iguaçu River, Brazil | *Basal polytomy* |
| BRH015 | BOLD | *Rhamdia voulezi* | H02 | Iguaçu River, Brazil | *Basal polytomy* |
| BSB006 | BOLD | *Rhamdia branneri* | H03 | Grande River, Brazil | *Rq4* |
| BSB025 | BOLD | *Rhamdia quelen* | H04 | Grande River, Brazil | *Rq4* |
| BSB062 | BOLD | *Rhamdia quelen* | H03 | Grande River, Brazil | *Rq4* |
| BSB108 | BOLD | *Rhamdia quelen* | H03 | Grande River, Brazil | *Rq4* |
| BSB209 | BOLD | *Rhamdia quelen* | H03 | Grande River, Brazil | *Rq4* |
| BSB377 | BOLD | *Rhamdia quelen* | H03 | Grande River, Brazil | *Rq4* |
| BSFFA450 | BOLD | *Rhamdia sp.* | H05 | Bayano River, Panama | *COI3* |
| BSFFA451 | BOLD | *Rhamdia sp.* | H06 | Canas River, Costa Rica | *COI3* |
| BSFFA452 | BOLD | *Rhamdia sp.* | H07 | Coto River, Costa Rica | *COI3* |
| BSFFA453 | BOLD | *Rhamdia sp.* | H08 | Sixaola River, Costa Rica | *COI3* |
| BSFFA454 | BOLD | *Rhamdia sp.* | H05 | Yape River, Panama | *COI3* |
| BSFFA455 | BOLD | *Rhamdia sp.* | H09 | Cocle del Norte River, Panama | *COI3* |
| BSFFA456 | BOLD | *Rhamdia sp.* | H10 | Mandinga River, Panama | *Basal polytomy* |
| BSFFA457 | BOLD | *Rhamdia sp.* | H05 | Ipeti River, Panama | *COI3* |
| BSFFA458 | BOLD | *Rhamdia sp.* | H11 | Esti River, Panama | *COI3* |
| BSFFA461 | BOLD | *Rhamdia sp.* | H05 | Cocle del Sur River, Panama | *COI3* |
| BSFFA463 | BOLD | *Rhamdia sp.* | H12 | San Juan River, Costa Rica | *COI3* |
| BSFFA728 | BOLD | *Rhamdia sp.* | H13 | Chagres River, Panama | *COI3* |
| BSFFA729 | BOLD | *Rhamdia sp.* | H05 | Playa Alta River, Panama | *COI3* |
| BSFFA812 | BOLD | *Rhamdia sp.* | H14 | Tranquera River, Nicaragua | *COI3* |
| BSFFA813 | BOLD | *Rhamdia sp.* | H15 | Santa Maria River, Panama | *COI3* |
| BSFFA814 | BOLD | *Rhamdia sp.* | H14 | Hato Grande River, Nicaragua | *COI3* |
| BSFFA873 | BOLD | *Rhamdia sp.* | H15 | Santa Maria River, Panama | *COI3* |
| BSFFA883 | BOLD | *Rhamdia sp.* | H05 | Membrillo River, Panama | *COI3* |
| FARG317 | BOLD | *Rhamdia quelen* | H01 | Mar Chiquita coastal lagoon, Argentina | *Rq6* |
| FARGB235 | BOLD | *Rhamdia quelen* | H16 | El Divisorio stream, Argentina | *Rq6* |
| FARGB236 | BOLD | *Rhamdia quelen* | H16 | El Divisorio stream, Argentina | *Rq6* |
| FARGB250 | BOLD | *Rhamdia quelen* | H01 | El Divisorio stream, Argentina | *Rq6* |
| FARGB297 | BOLD | *Rhamdia quelen* | H01 | Rojas River, Argentina | *Rq6* |
| FARGB365 | BOLD | *Rhamdia quelen* | H01 | Parana River, Argentina | *Rq6* |
| FPSR094 | BOLD | *Rhamdia quelen* | H17 | Paraiba do Sul Basin, Brazil | *Basal polytomy* |
| FPSR095 | BOLD | *Rhamdia quelen* | H17 | Paraiba do Sul Basin, Brazil | *Basal polytomy* |
| FPSR096 | BOLD | *Rhamdia quelen* | H17 | Paraiba do Sul Basin, Brazil | *Basal polytomy* |
| FPSR097 | BOLD | *Rhamdia quelen* | H17 | Paraiba do Sul Basin, Brazil | *Basal polytomy* |
| FPSR098 | BOLD | *Rhamdia quelen* | H17 | Paraiba do Sul Basin, Brazil | *Basal polytomy* |
| FUPR777 | BOLD | *Rhamdia quelen* | H18 | Upper Parana Basin, Brazil | *Rq4* |
| FUPR778 | BOLD | *Rhamdia quelen* | H19 | Upper Parana Basin, Brazil | *Rq4* |
| FUPR779 | BOLD | *Rhamdia quelen* | H03 | Upper Parana Basin, Brazil | *Rq4* |
| GBGC7572 | BOLD | *Rhamdia quelen* | H20 | No data available | *Basal polytomy* |
| GBGCA7590 | BOLD | *Rhamdia quelen* | H21 | Sao Paulo, Brazil | *Rq4* |
| GBMIN119705 | BOLD | *Rhamdia quelen* | H22 | Bolivia | *Basal polytomy* |
| GBMIN132214 | BOLD | *Rhamdia quelen* | H23 | No data available | *Basal polytomy* |
| GBMIN132215 | BOLD | *Rhamdia quelen* | H23 | No data available | *Basal polytomy* |
| ITAPE403 | BOLD | *Rhamdia quelen* | H24 | Itapecuru River, Brazil | *COI2* |
| ITAPE404 | BOLD | *Rhamdia quelen* | H25 | Itapecuru River, Brazil | *COI2* |
| KP225111 | GenBank | *Rhamdia quelen* | H26 | Peri Lagoon, Brazil | *Basal polytomy* |
| KP225112 | GenBank | *Rhamdia quelen* | H26 | Peri Lagoon, Brazil | *Basal polytomy* |
| KP225113 | GenBank | *Rhamdia quelen* | H27 | Peri Lagoon, Brazil | *Rq4* |
| KP225114 | GenBank | *Rhamdia quelen* | H26 | Peri Lagoon, Brazil | *Basal polytomy* |
| KP225115 | GenBank | *Rhamdia quelen* | H26 | Peri Lagoon, Brazil | *Basal polytomy* |
| KP225116 | GenBank | *Rhamdia quelen* | H01 | Commercial origin, fish farm | *Rq6* |
| KP225117 | GenBank | *Rhamdia quelen* | H01 | Commercial origin, fish farm | *Rq6* |
| KP225118 | GenBank | *Rhamdia quelen* | H01 | Commercial origin, fish farm | *Rq6* |
| KP225119 | GenBank | *Rhamdia quelen* | H28 | Commercial origin, fish farm | *Basal polytomy* |
| KP225120 | GenBank | *Rhamdia quelen* | H01 | Commercial origin, fish farm | *Rq6* |
| KP225121 | GenBank | *Rhamdia quelen* | H03 | Commercial origin, fish farm | *Rq4* |
| KP225122 | GenBank | *Rhamdia quelen* | H01 | Commercial origin, fish farm | *Rq6* |
| KP225123 | GenBank | *Rhamdia quelen* | H01 | Commercial origin, fish farm | *Rq6* |
| KP225124 | GenBank | *Rhamdia quelen* | H01 | Commercial origin, fish farm | *Rq6* |
| KP225125 | GenBank | *Rhamdia quelen* | H29 | Commercial origin, fish farm | *Rq4* |
| KP225126 | GenBank | *Rhamdia quelen* | H29 | Commercial origin, fish farm | *Rq4* |
| KP225127 | GenBank | *Rhamdia quelen* | H01 | Commercial origin, fish farm | *Rq6* |
| KP225128 | GenBank | *Rhamdia quelen* | H01 | Commercial origin, fish farm | *Rq6* |
| KP225129 | GenBank | *Rhamdia quelen* | H01 | Commercial origin, fish farm | *Rq6* |
| KP225130 | GenBank | *Rhamdia quelen* | H01 | Commercial origin, fish farm | *Rq6* |
| KP225131 | GenBank | *Rhamdia quelen* | H01 | Commercial origin, fish farm | *Rq6* |
| KP225132 | GenBank | *Rhamdia quelen* | H29 | Commercial origin, fish farm | *Rq4* |
| KP225133 | GenBank | *Rhamdia quelen* | H01 | Commercial origin, fish farm | *Rq6* |
| KP225134 | GenBank | *Rhamdia quelen* | H29 | Commercial origin, fish farm | *Rq4* |
| KP225135 | GenBank | *Rhamdia quelen* | H29 | Commercial origin, fish farm | *Rq4* |
| KP225136 | GenBank | *Rhamdia quelen* | H29 | Upper Uruguay River, Brazil | *Rq4* |
| KP225137 | GenBank | *Rhamdia quelen* | H30 | Upper Uruguay River, Brazil | *Rq4* |
| KP225138 | GenBank | *Rhamdia quelen* | H29 | Upper Uruguay River, Brazil | *Rq4* |
| KP225139 | GenBank | *Rhamdia quelen* | H29 | Upper Uruguay River, Brazil | *Rq4* |
| KP225140 | GenBank | *Rhamdia quelen* | H03 | Upper Uruguay River, Brazil | *Rq4* |
| KP225141 | GenBank | *Rhamdia quelen* | H03 | Upper Uruguay River, Brazil | *Rq4* |
| KP225142 | GenBank | *Rhamdia quelen* | H30 | Upper Uruguay River, Brazil | *Rq4* |
| KP225143 | GenBank | *Rhamdia quelen* | H29 | Upper Uruguay River, Brazil | *Rq4* |
| KP225144 | GenBank | *Rhamdia quelen* | H29 | Upper Uruguay River, Brazil | *Rq4* |
| KP225145 | GenBank | *Rhamdia quelen* | H03 | Upper Uruguay River, Brazil | *Rq4* |
| KP225146 | GenBank | *Rhamdia quelen* | H30 | Upper Uruguay River, Brazil | *Rq4* |
| KP225147 | GenBank | *Rhamdia quelen* | H29 | Upper Uruguay River, Brazil | *Rq4* |
| KP225148 | GenBank | *Rhamdia quelen* | H29 | Upper Uruguay River, Brazil | *Rq4* |
| KP225149 | GenBank | *Rhamdia quelen* | H30 | Upper Uruguay River, Brazil | *Rq4* |
| KP225150 | GenBank | *Rhamdia quelen* | H29 | Upper Uruguay River, Brazil | *Basal polytomy* |
| KP225151 | GenBank | *Rhamdia quelen* | H29 | Upper Uruguay River, Brazil | *Rq4* |
| KP225152 | GenBank | *Rhamdia quelen* | H29 | Upper Uruguay River, Brazil | *Rq4* |
| KP225153 | GenBank | *Rhamdia quelen* | H29 | Upper Uruguay River, Brazil | *Rq4* |
| KP225154 | GenBank | *Rhamdia quelen* | H29 | Upper Uruguay River, Brazil | *Rq4* |
| KP225155 | GenBank | *Rhamdia quelen* | H29 | Upper Uruguay River, Brazil | *Rq4* |
| KP225156 | GenBank | *Rhamdia quelen* | H29 | Upper Uruguay River, Brazil | *Rq4* |
| KP225157 | GenBank | *Rhamdia quelen* | H29 | Upper Uruguay River, Brazil | *Rq4* |
| KP225158 | GenBank | *Rhamdia quelen* | H29 | Upper Uruguay River, Brazil | *Rq4* |
| KU845687 | GenBank | *Rhamdia quelen* | H31 | Quexada River, Brazil | *Rq4* |
| KU845688 | GenBank | *Rhamdia quelen* | H29 | Quexada River, Brazil | *Rq4* |
| KU845689 | GenBank | *Rhamdia quelen* | H03 | Quexada River, Brazil | *Rq4* |
| KU845690 | GenBank | *Rhamdia quelen* | H32 | Miranda River | *Rq2* |
| KU845691 | GenBank | *Rhamdia quelen* | H32 | Miranda River | *Rq2* |
| KU845692 | GenBank | *Rhamdia quelen* | H32 | Miranda River | *Rq2* |
| KU845693 | GenBank | *Rhamdia quelen* | H32 | Miranda River | *Rq2* |
| KU845694 | GenBank | *Rhamdia quelen* | H32 | Miranda River | *Rq2* |
| LARGI368 | BOLD | *Rhamdia quelen* | H01 | Parana River, Argentina |  |
| MNCE190 | BOLD | *Rhamdia quelen* | H23 | San Francisco River, Brazil | *Basal polytomy* |
| MNCE191 | BOLD | *Rhamdia quelen* | H23 | San Francisco River, Brazil | *Basal polytomy* |
| MNCE192 | BOLD | *Rhamdia quelen* | H23 | San Francisco River, Brazil | *Basal polytomy* |
| MUCU033 | BOLD | *Rhamdia quelen* | H33 | Atlantic Ocean. Brazil | *Basal polytomy* |
| MUCU083 | BOLD | *Rhamdia quelen* | H33 | Atlantic Ocean. Brazil | *Basal polytomy* |
| MK511191* | GenBank | *Rhamdia quelen* | H01 | Rocha lagoon, Uruguay | *Rq6* |
| MK511192* | GenBank | *Rhamdia quelen* | H34 | Hatchery, Uruguay | *Rq2* |
| MN233639* | GenBank | *Rhamdia quelen* | H34 | Uruguay River | *Rq2* |
| MN233636* | GenBank | *Rhamdia quelen* | H34 | Hatchery, Uruguay | *Rq2* |
| MN233635* | GenBank | *Rhamdia quelen* | H35 | Hatchery, Uruguay | *Rq2* |
| MN233641* | GenBank | *Rhamdia quelen* | H01 | Merin Lagoon | *Rq6* |
| MN233642* | GenBank | *Rhamdia quelen* | H01 | Negro River, Uruguay | *Rq6* |
| MN233634* | GenBank | *Rhamdia quelen* | H01 | Castillos lagoon | *Rq6* |
| MN233638* | GenBank | *Rhamdia quelen* | H36 | Uruguay River, Uruguay | *Rq4* |
| MN233640* | GenBank | *Rhamdia quelen* | H36 | Uruguay River, Uruguay | *Rq4* |
| MN233637* | GenBank | *Rhamdia quelen* | H29 | Uruguay River, Uruguay | *Rq4* |
| MK511193* | GenBank | *Rhamdia quelen* | H37 | Uruguay River | *Rq4* |
| TZGAA005 | BOLD | *Rhamdia quelen* | H38 | Essequibo River, Guyana | *COI1* |
| TZGAA016 | BOLD | *Rhamdia quelen* | H38 | Essequibo River, Guyana | *COI1* |
| TZGAA027 | BOLD | *Rhamdia quelen* | H38 | Essequibo River, Guyana | *COI1* |
| TZGA039 | BOLD | *Rhamdia quelen* | H39 | Essequibo River, Guyana | *COI1* |
| TGZAA051 | BOLD | *Rhamdia quelen* | H38 | Essequibo River, Guyana | *COI1* |
| TZGAA063 | BOLD | *Rhamdia quelen* | H38 | Essequibo River, Guyana | *COI1* |
| TZGA075 | BOLD | *Rhamdia quelen* | H38 | Essequibo River, Guyana | *COI1* |
| TZGAA087 | BOLD | *Rhamdia quelen* | H38 | Essequibo River, Guyana | *COI1* |
| TZGAA088 | BOLD | *Rhamdia quelen* | H38 | Essequibo River, Guyana | *COI1* |
| UDEA184 | BOLD | *Rhamdia quelen* | H40 | Campoalegre River, Colombia | *COI3* |
| UDEA185 | BOLD | *Rhamdia quelen* | H41 | Campoalegre River, Colombia | *COI3* |
| UDEA186 | BOLD | *Rhamdia quelen* | H40 | Campoalegre River, Colombia | *COI3* |
| UDEA187 | BOLD | *Rhamdia quelen* | H10 | Cienega River, Colombia | *COI3* |
| UDEA188 | BOLD | *Rhamdia quelen* | H10 | Cienega River, Colombia | *COI3* |

(*) Specimens that belong to *Rq2*, *Rq4* or *Rq6* mitochondrial lineage based on phylogenetics analysis of cytochrome b.
