# Supplementary material for: Population Genomics in Rhamdia quelen (Heptapteridae, Siluriformes) Reveals Deep Divergence and Adaptation in the Neotropical Region
Source: Genes (Basel). 2020 Jan 17;11(1):109. doi: 10.3390/genes11010109 (PMC7017130; doi:10.3390/genes11010109)
Supplement: Supplementary file 1 [file genes-11-00109-s001.zip › Supplementary File SIV.docx]

**Supplemental File IV.** Analyses of outlier loci subjected to selection analyzed by Bayescan and Arlequin.

1. Analysis of all samples

BayeScan

Pop1 = 1-UR-CR; Pop2= 2-UR-AR; Pop3= 3-UR-QR; Pop4= 4-NR-RB; Pop5= 5-LP-SL; Pop6= 6-AO-BL; Pop7= 7-AO-RL; Pop8= 8-AO-CL; Pop9= 9-ML-QC; Pop10= 10-VC

Result: 185 outlier loci identified.

Arlequin

Pop1 = 1-UR-CR; Pop2= 2-UR-AR; Pop3= 3-UR-QR; Pop4= 4-NR-RB; Pop5= 5-LP-SL; Pop6= 6-AO-BL; Pop7= 7-AO-RL; Pop8= 8-AO-CL; Pop9= 9-ML-QC; Pop10= 10-VC (non- hierarchical finite island model)

Result: 690 outlier loci identified.

Confident set of outlier loci considering both BayeScan and Arlequin results: 72.

2) Analysis of all samples

BayeScan

Pop1 = 1-UR-CR; Pop2= 2-UR-AR; Pop3= 3-UR-QR; Pop4= 4-NR-RB; Pop5= 5-LP-SL; Pop6= 6-AO-BL; Pop7= 7-AO-RL; Pop8= 8-AO-CL; Pop9= 9-ML-QC; Pop10= 10-VC

Result: 185 outlier loci identified.

Arlequin hierarchical analysis

Group 1 (North): Pop1 = 1-UR-CR, Pop2= 2-UR-AR, Pop3= 3-UR-QR; Group 2 (Centre): Pop4= 4-NR-RB, Pop9= 9-ML-QC; Group 3 (South): Pop5= 5-LP-S, Pop6= 6-AO-BL, Pop7= 7-AO-RL, Pop8= 8-AO-CL; Group 4 (hatchery): Pop10= 10-VC

Result: 172 outlier loci identified.

Confident set of outlier loci considering both BayeScan and Arlequin results: 63.

3) Analysis of natural locality samples grouped for basins

BayeScan

Pop1 = 1-UR-CR; Pop2= 2-UR-AR; Pop3= 3-UR-QR; Pop4= 4-NR-RB; Pop5= 5-LP-SL; Pop6= 6-AO-BL; Pop7= 7-AO-RL; Pop8= 8-AO-CL; Pop9= 9-ML-QC

Result: 63 outlier loci identified.

Arlequin hierarchical analysis

Group 1 (Uruguay river basin): Pop1 = 1-UR-CR, Pop2= 2-UR-AR, Pop3= 3-UR-QR; Group 2 (Negro river basin): Pop4= 4-NR-RB; Group 3 (La Plata river basin): Pop5= 5-LP-SL; Group 4 (Atlantic Ocean basin): Pop6= 6-AO-BL, Pop7= 7-AO-RL, Pop8= 8-AO-CL; Group 5 (Merin lagoon basin) Pop9= 9-ML-QC

Result: outlier loci 545 identified.

Confident set of outlier loci considering both BayeScan and Arlequin results: 13.

4) Analysis of natural locality samples grouped for environment

BayeScan

Pop1 = 1-UR-CR; Pop2= 2-UR-AR; Pop3= 3-UR-QR; Pop4= 4-NR-RB; Pop5= 5-LP-SL; Pop6= 6-AO-BL; Pop7= 7-AO-RL; Pop8= 8-AO-CL; Pop9= 9-ML-QC

Result: 63 outlier loci identified.

Arlequin hierarchical analysis

Group 1 (rivers environment): Pop1 = 1-UR-CR, Pop2= 2-UR-AR, Pop3= 3-UR-QR, Pop4= 4-NR-RB, Pop9= 9-ML-QC; Group 2 (coastal lagoon): Pop5= 5-LP-S, Pop6= 6-AO-BL, Pop7= 7-AO-RL, Pop8= 8-AO-CL

Result: 325 outlier loci identified.

Confident set of outlier loci considering both BayeScan and Arlequin results: 4.

5) Analysis of natural locality samples grouped for region

BayeScan

Pop1 = 1-UR-CR; Pop2= 2-UR-AR; Pop3= 3-UR-QR; Pop4= 4-NR-RB; Pop5= 5-LP-SL; Pop6= 6-AO-BL; Pop7= 7-AO-RL; Pop8= 8-AO-CL; Pop9= 9-ML-QC

Result: 63 outlier loci identified.

Arlequin hierarchical analysis

Group 1 (North): Pop1 = 1-UR-CR, Pop2= 2-UR-AR, Pop3= 3-UR-QR; Group 2 (Centre): Pop4= 4-NR-RB, Pop9= 9-ML-QC; Group 3 (South): Pop5= 5-LP-S, Pop6= 6-AO-BL, Pop7= 7-AO-RL, Pop8= 8-AO-CL

Result: 394 outlier loci identified.

Confident set of outlier loci considering both BayeScan and Arlequin results: 1.

6) Analysis of locality samples from North and South grouped for region

BayeScan

Pop1 = 1-UR-CR; Pop2= 2-UR-AR; Pop3= 3-UR-QR; Pop4= 5-LP-SL; Pop5= 6-AO-BL; Pop6= 7-AO-RL; Pop7= 8-AO-CL

Result: 174 outlier loci identified.

Arlequin hierarchical analysis

Group 1 (North): Pop1 = 1-UR-CR, Pop2= 2-UR-AR, Pop3= 3-UR-QR; Group 2 (South): Pop5= 5-LP-S, Pop6= 6-AO-BL, Pop7= 7-AO-RL, Pop8= 8-AO-CL

Result: 95 outlier loci identified.

Confident set of outlier loci considering both BayeScan and Arlequin results: 3.

7) Analysis of locality samples from North and South

BayeScan

Pop1 = 1-UR-CR; Pop2= 2-UR-AR; Pop3= 3-UR-QR; Pop4= 5-LP-SL; Pop5= 6-AO-BL; Pop6= 7-AO-RL; Pop7= 8-AO-CL

Result: 174 outlier loci identified.

Arlequin

Pop1 = 1-UR-CR, Pop2= 2-UR-AR, Pop3= 3-UR-QR, Pop5= 5-LP-S, Pop6= 6-AO-BL, Pop7= 7-AO-RL, Pop8= 8-AO-CL (non- hierarchical finite island model)

Result: 524 outlier loci identified.

Confident set of outlier loci considering both BayeScan and Arlequin results: 73.

8) Analysis of locality samples from North and Centre grouped for region

BayeScan

Pop1 = 1-UR-CR; Pop2= 2-UR-AR; Pop3= 3-UR-QR; Pop4= 4-NR-RB; Pop9= 9-ML-QC

Result: 0 outlier locus.

Arlequin hierarchical analysis

Group 1 (North): Pop1 = 1-UR-CR, Pop2= 2-UR-AR, Pop3= 3-UR-QR; Group 2 (Centre): Pop4= 4-NR-RB, Pop9= 9-ML-QC

Result: 165 outlier loci identified.

Confident set of outlier loci considering both BayeScan and Arlequin results: 0.

9) Analysis of locality samples from North and Centre

BayeScan

Pop1 = 1-UR-CR; Pop2= 2-UR-AR; Pop3= 3-UR-QR; Pop4= 4-NR-RB; Pop9= 9-ML-QC

Result: 0 outlier locus.

Arlequin hierarchical analysis

Pop1 = 1-UR-CR, Pop2= 2-UR-AR, Pop3= 3-UR-QR, Pop4= 4-NR-RB, Pop9= 9-ML-QC (non- hierarchical finite island model)

Result: 185 outlier loci.

Confident set of outlier loci considering both BayeScan and Arlequin results: 0.

10) Analysis of locality samples from Centre and South grouped for region

BayeScan

Pop1= 4-NR-RB; Pop2= 5-LP-SL; Pop3= 6-AO-BL; Pop4= 7-AO-RL; Pop5= 8-AO-CL; Pop6= 9-ML-QC

Result: 0 outlier locus.

Arlequin hierarchical analysis

Group 1 (Centre): Pop4= 4-NR-RB, Pop9= 9-ML-QC; Group 2 (South): Pop5= 5-LP-S, Pop6= 6-AO-BL, Pop7= 7-AO-RL, Pop8= 8-AO-CL

Result: 401 outlier loci.

Confident set of outlier loci considering both BayeScan and Arlequin results: 0.

11) Analysis of locality samples from Centre and South

BayeScan

Pop1= 4-NR-RB; Pop2= 5-LP-SL; Pop3= 6-AO-BL; Pop4= 7-AO-RL; Pop5= 8-AO-CL; Pop6= 9-ML-QC

Result: 0 outlier locus.

Arlequin

Pop4= 4-NR-RB, Pop9= 9-ML-QC, Pop5= 5-LP-S, Pop6= 6-AO-BL, Pop7= 7-AO-RL, Pop8= 8-AO-CL (non- hierarchical finite island model)

Result: 265 outlier loci identified.

Confident set of outlier loci considering both BayeScan and Arlequin results: 0.

12) Analysis of coastal lagoon samples

BayeScan

Pop1= 5-LP-SL; Pop2= 6-AO-BL; Pop3= 7-AO-RL; Pop4= 8-AO-CL

Result: 0 outlier locus.

Arlequin

Pop1= 5-LP-S, Pop2= 6-AO-BL, Pop3= 7-AO-RL, Pop4= 8-AO-CL (non- hierarchical finite island model)

Result: 144 outlier loci identified.

Confident set of outlier loci considering both BayeScan and Arlequin results: 0.

13) Analysis of samples from riverine environment

BayeScan

Pop1 = 1-UR-CR; Pop2= 2-UR-AR; Pop3= 3-UR-QR; Pop4= 4-NR-RB; Pop5= 9-ML-QC

Result: 0 outlier locus identified.

Arlequin

Pop1 = 1-UR-CR, Pop2= 2-UR-AR, Pop3= 3-UR-QR, Pop4= 4-NR-RB, Pop5= 9-ML-QC (non- hierarchical finite island model)

Result: 179 outlier loci identified.

Confident set of outlier loci considering both BayeScan and Arlequin results: 0.

14) Analysis of locality samples from North

BayeScan

Pop1 = 1-UR-CR; Pop2= 2-UR-AR; Pop3= 3-UR-QR

Result: 0 outlier locus identified.

Arlequin

Pop1 = 1-UR-CR, Pop2= 2-UR-AR, Pop3= 3-UR-QR (non- hierarchical finite island model)

Result: 72 outlier loci identified.

Confident set of outlier loci considering both BayeScan and Arlequin results: 0.

15) Analysis of locality samples from South

BayeScan

Pop1= 5-LP-SL; Pop2= 6-AO-BL; Pop3= 7-AO-RL; Pop4= 8-AO-CL

Result: 0 outlier locus identified.

Arlequin

Pop1= 5-LP-S, Pop2= 6-AO-BL, Pop3= 7-AO-RL, Pop4= 8-AO-CL (non- hierarchical finite island model)

Result: 144 outlier loci identified.

Confident set of outlier loci considering both BayeScan and Arlequin results: 0.

16) Analysis of locality samples from Centre

BayeScan

Pop1 = 4-NR-RB; Pop2= 9-ML-QC

Result: 0 outlier locus identified.

Arlequin

Pop1= 4-NR-RB, Pop2= 9-ML-QC (non- hierarchical finite island model)

Result: 58 outlier loci identified.

Confident set of outlier loci considering both BayeScan and Arlequin results: 0 outlier locus

17) Analysis of locality samples from Uruguay river and Negro river basins

BayeScan

Pop1 = 1-UR-CR; Pop2= 2-UR-AR; Pop3= 3-UR-QR; Pop4= 4-NR-RB

Result: 0 outlier locus identified.

Arlequin

Group 1 (Uruguay river): Pop1 = 1-UR-CR, Pop2= 2-UR-AR, Pop3= 3-UR-QR; Group 2 (Negro river): Pop4= 4-NR-RB

Result: 140 outlier loci identified.

Confident set of outlier loci considering both BayeScan and Arlequin results: 0.

18) Analysis of locality samples from Uruguay river and Merin lagoon basin

Pop1 = 1-UR-CR; Pop2= 2-UR-AR; Pop3= 3-UR-QR; Pop4= 9-ML-QC

Result: 0 outlier locus identified.

Arlequin

Group 1 (Uruguay river): Pop1 = 1-UR-CR, Pop2= 2-UR-AR, Pop3= 3-UR-QR; Group 2 (Merin lagoon): Pop4= 9-ML-QC

Result: 92 outlier loci identified.

Confident set of outlier loci considering both BayeScan and Arlequin results: 0.

19) Analysis of locality samples from Uruguay river and La Plata river basin

BayeScan

Pop1 = 1-UR-CR; Pop2= 2-UR-AR; Pop3= 3-UR-QR; Pop4= 5-LP-SL

Result: 0 outlier locus identified.

Arlequin hierarchical analysis

Group 1 (Uruguay river): Pop1 = 1-UR-CR, Pop2= 2-UR-AR, Pop3= 3-UR-QR; Group 2 (La Plata basin): Pop4= 5-LP-SL

Result: 106 outlier loci identified.

Confident set of outlier loci considering both BayeScan and Arlequin results: 0.

20) Analysis of locality samples from North and South Uruguay river and Atlantic Ocean basins

BayeScan

Pop1 = 1-UR-CR; Pop2= 2-UR-AR; Pop3= 3-UR-QR; Pop4= 6-AO-BL; Pop5= 7-AO-RL; Pop6= 8-AO-CL

Result: 0 outlier locus identified.

Arlequin hierarchical analysis

Group 1 (Uruguay river): Pop1 = 1-UR-CR, Pop2= 2-UR-AR, Pop3= 3-UR-QR; Group 2 (Atlantic Ocean basin): Pop4= 6-AO-BL, Pop5= 7-AO-RL, Pop6= 8-AO-CL

Result: 92 outlier loci

Confident set of outlier loci considering both BayeScan and Arlequin results: 0

21) Analysis of locality samples from Negro river and Merin lagoon

BayeScan

Pop1 = 4-NR-RB; Pop2= 2-ML-QC

Result: 0 outlier locus identified.

Arlequin hierarchical analysis

Group 1 (Negro river): Pop1 = 4-NR-RB; Group 2 (Merin lagoon): Pop2= 9-ML-QC

Result: 58 outlier loci identified.

Confident set of outlier loci considering both BayeScan and Arlequin results: 0.

22) Analysis of locality samples from Negro river and La Plata basins

BayeScan

Pop1 = 4-NR-RB; Pop2= 5-LP-SL

Result: 0 outlier locus identified.

Arlequin hierarchical analysis

Group 1 (Negro river): Pop1 = 4-NR-RB; Group 2 (La Plata basin): Pop2= 5-LP-SL

Result: 11 outlier loci identified.

Confident set of outlier loci considering both BayeScan and Arlequin results: 0

23) Analysis of locality samples from Negro river and Atlantic Ocean basins

BayeScan

Pop1 = 4-NR-RB; Pop2= 6-AO-BL; Pop3= 7-AO-RL; Pop4= 8-AO-CL

Result: 0 outlier locus identified.

Arlequin hierarchical analysis

Group 1 (Uruguay river): Pop1 = 4-NR-RB; Group 2 (Atlantic Ocean basin): Pop2= 6-AO-BL, Pop3= 7-AO-RL, Pop4= 8-AO-CL

Result: 109 outlier loci identified.

Confident set of outlier loci considering both BayeScan and Arlequin results: 0.

24) Analysis of locality samples from Atlantic Ocean basins and Merin lagoon

BayeScan

Pop1= 6-AO-BL; Pop2= 7-AO-RL; Pop3= 8-AO-CL; Pop4= 9-ML-QC

Result: 0 outlier locus identified.

Arlequin hierarchical analysis

Group 1 (Atlantic Ocean basin): Pop1= 6-AO-BL, Pop2= 7-AO-RL, Pop3= 8-AO-CL; Group 2 (Merin lagoon): Pop4= 9-ML-QC

Result: 54 outlier loci identified.

Confident set of outlier loci considering both BayeScan and Arlequin results: 0.

25) Analysis of locality samples from La Plata basin and Merin lagoon

BayeScan

Pop1= 5-LP-SL; Pop2= 9-ML-QC

Result: 0 outlier locus identified.

Arlequin hierarchical analysis

Group 1 (Atlantic Ocean basin): Pop1= 5-LP-SL; Group 2 (Merin lagoon): Pop4= 9-ML-QC

Result: 37 outlier loci identified.

Confident set of outlier loci considering both BayeScan and Arlequin results: 0.

26) Analysis of locality samples from La Plata basin and Atlantic Ocean basins

BayeScan

Pop1= 6-AO-BL; Pop2= 7-AO-RL; Pop3= 8-AO-CL; Pop4= 5-LP-SL

Result: 0 outlier locus identified.

Arlequin hierarchical analysis

Group 1 (Atlantic Ocean basin): Pop1= 6-AO-BL, Pop2= 7-AO-RL, Pop3= 8-AO-CL; Group 2 (La Plata basin): Pop4= 5-LP-SL

Result: 130 outlier loci identified.

Confident set of outlier loci considering both BayeScan and Arlequin results: 0.

27) Non-hierarchical analysis of all pair of locality samples did not show any confident outlier locus.
