# Supplementary material for: Population Genomics in Rhamdia quelen (Heptapteridae, Siluriformes) Reveals Deep Divergence and Adaptation in the Neotropical Region
Source: Genes (Basel). 2020 Jan 17;11(1):109. doi: 10.3390/genes11010109 (PMC7017130; doi:10.3390/genes11010109)
Supplement: Supplementary file 1 [file genes-11-00109-s001.zip › Supplementary File SIX.docx]

Table SIX 1. Structure Harvester results for the Total dataset.

| K | Mean LnP(K) | Stdev LnP(K) | Ln'(K) | \|Ln''(K)\| | Delta K |
| --- | --- | --- | --- | --- | --- |
| 1 | -679477.94 | 13.88 | NA | NA | NA |
| 2 | -501434.94 | 74.24 | 178043.00 | 172031.14 | 2317.29593 |
| 3 | -495423.08 | 934.95 | 6011.86 | 10335.80 | 11.05 |
| 4 | -479075.42 | 1503.40 | 16347.66 | 1662.82 | 1.11 |
| 5 | -464390.58 | 424.47 | 14684.84 | 10205.66 | 24.04 |
| 6 | -459911.40 | 3365.99 | 4479.18 | 1875744.88 | 557.26 |
| 7 | -2331177.10 | 405918.89 | -1871265.70 | 352248.60 | 0.87 |
| 8 | -3850194.20 | 3499435.56 | -1519017.10 | 1024450.91 | 0.29 |
| 9 | -6393662.21 | 3834868.46 | -2543468.01 | 1609055.77 | 0.42 |
| 10 | -10546185.99 | 11498865.84 | -4152523.78 | 2163746.74 | 0.19 |
| 11 | -12534963.03 | 20514825.16 | -1988777.04 | 7803653.97 | 0.38 |
| 12 | -6720086.10 | 10582391.47 | 5814876.93 | 19688622.16 | 1.86 |
| 13 | -20593831.33 | 19452803.91 | -13873745.23 | NA | NA |

Mean LnP(K): mean of the estimate of log of the probability of the data; Stdev LnP(K): standard deviation of the log likelihood values; Ln'(K): rate of change of the likelihood distribution; |Ln''(K)|: Absolut value of the second order rate of change of the likelihood distribution (mean); Delta K: mean(|Ln''(K)|) / Stdev LnP(K).


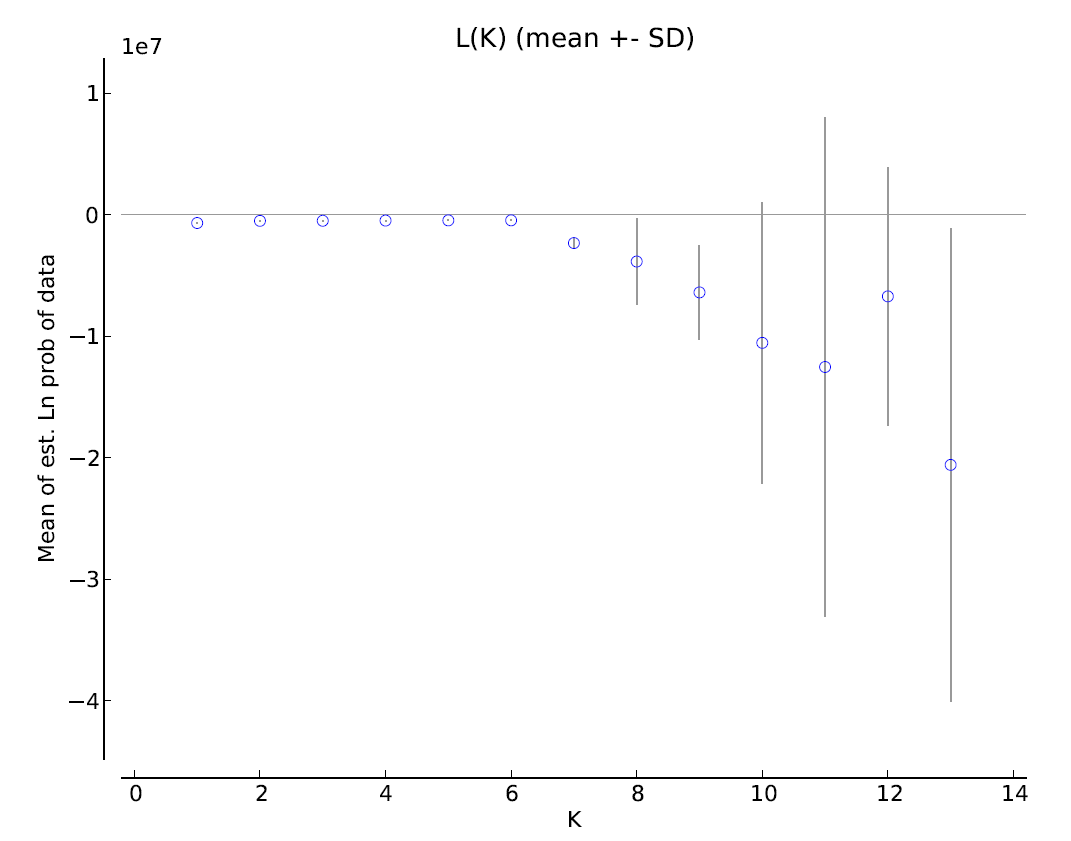


Figure. SIX 1. Plot of mean likelihood L(K) and variance for 10 independent runs per K value from STRUCTURE based on the Total dataset.


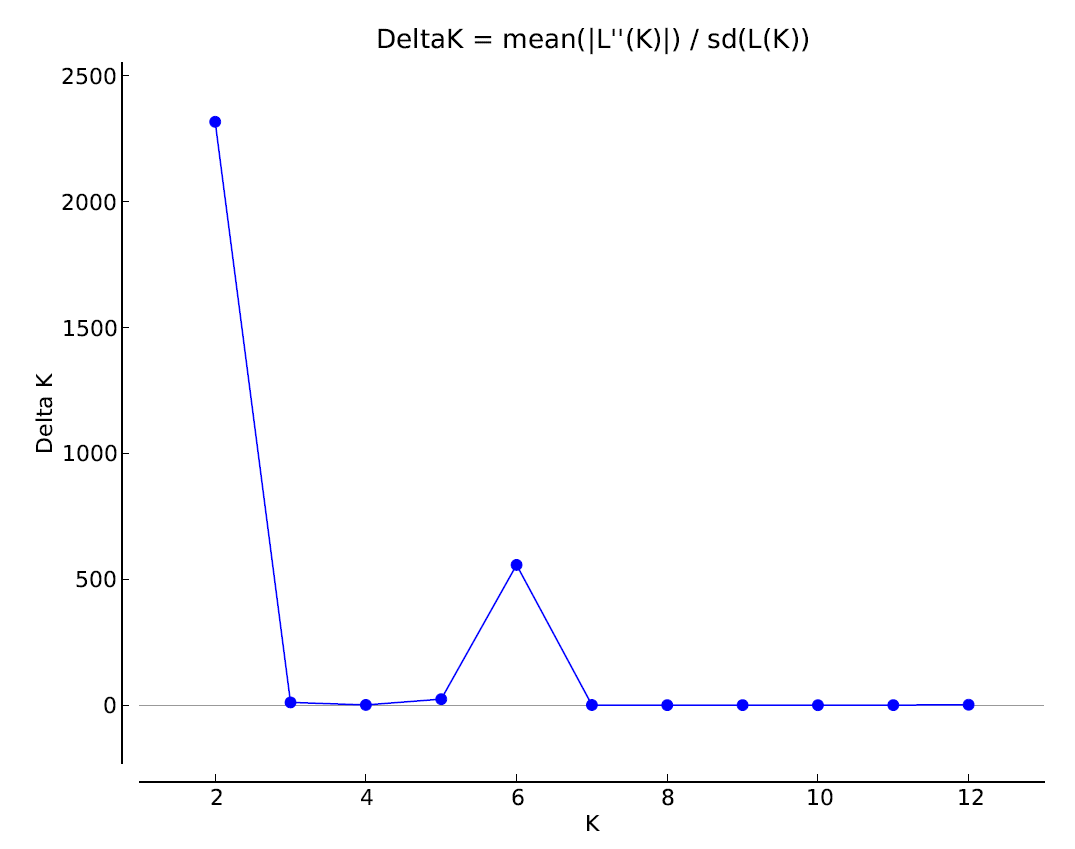


Figure. SIX 2. Plot of Delta K for 10 independent runs per K value from STRUCTURE based on the Total dataset.

Table SIX 2. Structure Harvester results for the Neutral dataset.

| K | Mean LnP(K) | Stdev LnP(K) | Ln'(K) | \|Ln''(K)\| | Delta K |
| --- | --- | --- | --- | --- | --- |
| 1 | -549705.53 | 35.10 | NA | NA | NA |
| 2 | -434402.11 | 58.86 | 115303.42 | 109437.73 | 1859.18 |
| 3 | -428536.42 | 430.56 | 5865.69 | 6447.77 | 14.98 |
| 4 | -416222.96 | 743.67 | 12313.46 | 1750.72 | 2.35 |
| 5 | -405660.22 | 5851.23 | 10562.74 | 1096419.44 | 187.38 |
| 6 | -1491516.92 | 1523157.70 | -1085856.70 | 1179668.18 | 0.77 |
| 7 | -3757041.80 | 3239739.83 | -2265524.88 | 3523165.50 | 1.09 |
| 8 | -2499401.18 | 3381015.12 | 1257640.62 | 3237016.19 | 0.96 |
| 9 | -4478776.75 | 5651729.68 | -1979375.57 | 4620490.55 | 0.82 |
| 10 | -1837661.77 | 567039.13 | 2641114.98 | 7092726.72 | 12.51 |
| 11 | -6289273.50 | 5002294.23 | -4451611.73 | 7120761.33 | 1.42 |
| 12 | -3620123.90 | 3946254.90 | 2669149.60 | 981578.00 | 0.25 |
| 13 | -3873013.96 | 3360990.70 | -252890.07 | NA | NA |

Mean LnP(K): mean of the estimate of log of the probability of the data; Stdev LnP(K): standard deviation of the log likelihood values; Ln'(K): rate of change of the likelihood distribution; |Ln''(K)|: Absolut value of the second order rate of change of the likelihood distribution (mean); Delta K: mean(|Ln''(K)|) / Stdev LnP(K).


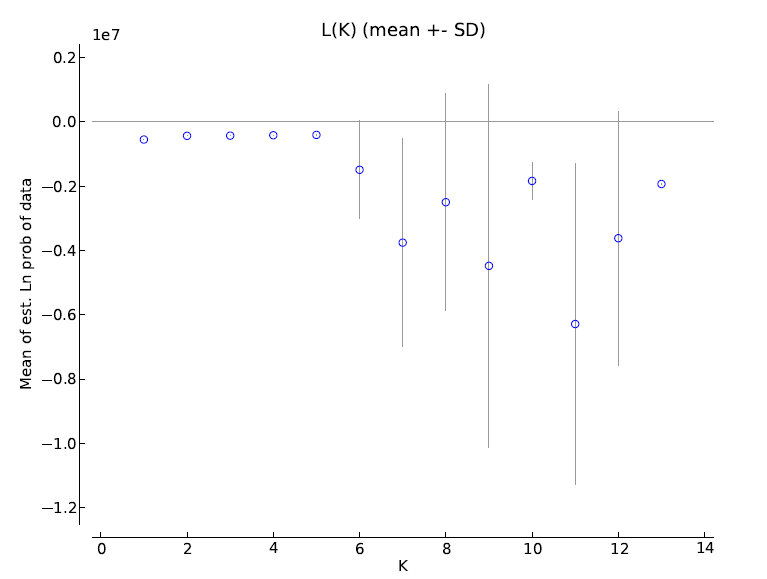


Figure. SIX 3. Plot of mean likelihood L(K) and variance for 10 independent runs per K value from STRUCTURE based on the Neutral dataset.


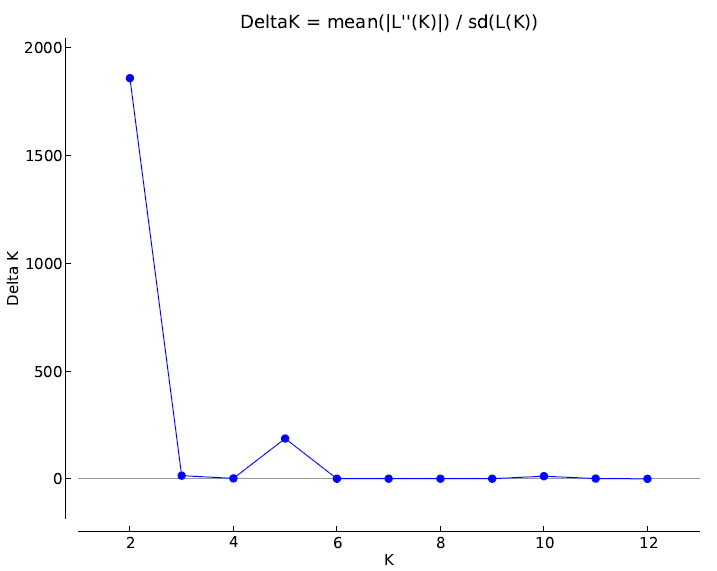


Figure. SIX 4. Plot of Delta K for 10 independent runs per K value from STRUCTURE based on the Neutral dataset.

Table SIX 3. Structure Harvester results for the Outlier dataset.

| K | Mean LnP(K) | Stdev LnP(K) | Ln'(K) | \|Ln''(K)\| | Delta K |
| --- | --- | --- | --- | --- | --- |
| 1 | -6668.82 | 0.26 | NA | NA | NA |
| 2 | -973.43 | 1.61 | 5695.39 | 5706.87 | 3536.15 |
| 3 | -984.91 | 7.08 | -11.48 | 13.78 | 1.95 |
| 4 | -982.61 | 14.64 | 2.30 | 4.94 | 0.34 |
| 5 | -985.25 | 4.57 | -2.64 | 0.39 | 0.09 |
| 6 | -987.50 | 16.82 | -2.25 | 6.18 | 0.37 |
| 7 | -995.93 | 8.55 | -8.43 | 15.75 | 1.84 |
| 8 | -988.61 | 16.19 | 7.32 | 62.66 | 3.87 |
| 9 | -1043.95 | 104.23 | -55.34 | 87.86 | 0.84 |
| 10 | -1011.43 | 46.97 | 32.52 | 17.37 | 0.37 |
| 11 | -996.28 | 16.24 | 15.15 | 15.82 | 0.97 |
| 12 | -996.95 | 12.15 | -0.67 | 87.38 | 7.19 |
| 13 | -1085.00 | 98.72 | -88.05 | NA | NA |

Mean LnP(K): mean of the estimate of log of the probability of the data; Stdev LnP(K): standard deviation of the log likelihood values; Ln'(K): rate of change of the likelihood distribution; |Ln''(K)|: Absolut value of the second order rate of change of the likelihood distribution (mean); Delta K: mean(|Ln''(K)|) / Stdev LnP(K).


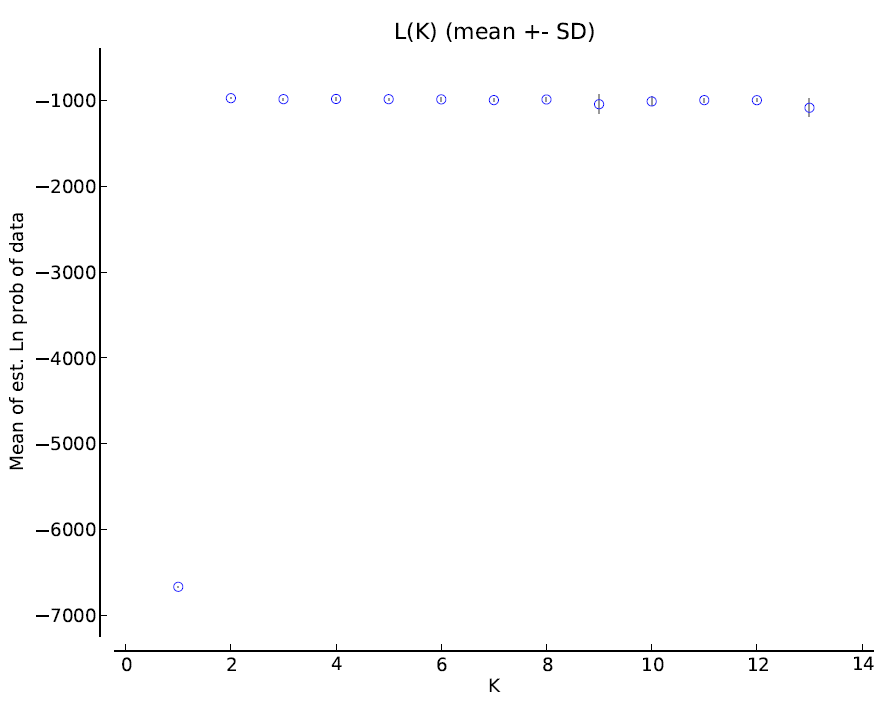


Figure. SIX 5. Plot of mean likelihood L(K) and variance for 10 independent runs per K value from STRUCTURE based on the Outlier dataset.


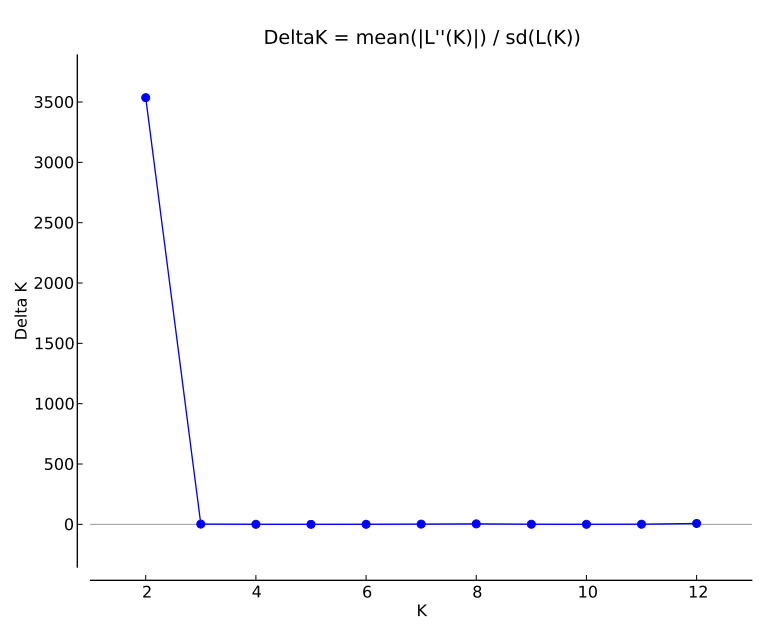


Figure. SVIII 6. Plot of Delta K for 10 independent runs per K value from STRUCTURE based on the Outlier dataset.
