# Supplementary material for: Population Genomics in Rhamdia quelen (Heptapteridae, Siluriformes) Reveals Deep Divergence and Adaptation in the Neotropical Region
Source: Genes (Basel). 2020 Jan 17;11(1):109. doi: 10.3390/genes11010109 (PMC7017130; doi:10.3390/genes11010109)
Supplement: Supplementary file 1 [file genes-11-00109-s001.zip › Supplementary File SV.docx]

**Table SV 1**. Values of environmental variables examined for the sampled localities.

|  | 1-UR-CR | 2-UR-AR | 3-UR-QR | 4-NR-SG | 5-LP-SL | 6-AO-BL | 7-AO-RL | 8-AO-CL | 9-ML-QC |
| --- | --- | --- | --- | --- | --- | --- | --- | --- | --- |
| bio-1 | 197 | 188 | 183 | 178 | 168 | 165 | 163 | 165 | 173 |
| bio-2 | 117 | 120 | 121 | 120 | 78 | 74 | 95 | 98 | 106 |
| bio-3 | 47 | 47 | 47 | 46 | 42 | 41 | 47 | 47 | 49 |
| bio-4 | 4475 | 4621 | 4664 | 4596 | 3789 | 3718 | 3757 | 3639 | 3710 |
| bio-5 | 328 | 325 | 322 | 318 | 267 | 260 | 270 | 275 | 293 |
| bio-6 | 84 | 74 | 66 | 62 | 82 | 81 | 70 | 73 | 79 |
| bio-7 | 244 | 250 | 256 | 256 | 182 | 177 | 199 | 204 | 214 |
| bio-8 | 229 | 220 | 239 | 177 | 134 | 121 | 120 | 119 | 127 |
| bio-9 | 142 | 132 | 125 | 239 | 202 | 175 | 197 | 180 | 212 |
| bio-10 | 256 | 249 | 244 | 238 | 216 | 212 | 211 | 211 | 219 |
| bio-11 | 142 | 132 | 126 | 121 | 116 | 118 | 116 | 119 | 127 |
| bio-12 | 1294 | 1306 | 1222 | 1164 | 1071 | 1041 | 1083 | 1122 | 1193 |
| bio-13 | 146 | 146 | 137 | 122 | 101 | 101 | 100 | 106 | 112 |
| bio-14 | 66 | 75 | 72 | 78 | 68 | 63 | 60 | 69 | 90 |
| bio-15 | 23 | 19 | 19 | 12 | 10 | 12 | 11 | 9 | 7 |
| bio-16 | 410 | 388 | 368 | 326 | 287 | 288 | 293 | 300 | 316 |
| bio-17 | 226 | 253 | 234 | 262 | 230 | 225 | 236 | 253 | 281 |
| bio-18 | 360 | 339 | 326 | 262 | 263 | 260 | 274 | 282 | 295 |
| bio-19 | 227 | 252 | 234 | 292 | 285 | 283 | 290 | 300 | 318 |
| Cond | 72 | 17.45 | 238 | 43 | 170 | 500 | 17700 | 5350 | 116.67 |
| Lat | 30.28 | 31.19 | 32.18 | 32.6 | 34.83 | 34.9 | 34.61 | 34.31 | 33.16 |
| Long | 57.25 | 57.14 | 57.62 | 55.82 | 55.06 | 54.8 | 54.29 | 53.93 | 53.79 |

bio-1: annual mean temperature; bio-2: mean diurnal range; bio-3: isothermality; bio-4: temperature seasonality; bio-5: maximum temperature of warmest month; bio-6: minimum temperature of coldest month; bio-7: annual temperature range; bio-8: mean temperature of wettest quarter; bio-9: mean temperature of driest quarter; bio-10: mean temperature of warmest quarter; bio-11: mean temperature of coldest quarter; bio-12: annual precipitation; bio-13: precipitation of wettest month; bio-14: precipitation of driest month; bio-15: precipitation seasonality; bio-16: precipitation of wettest quarter; bio-17: precipitation of driest quarter; bio-18: precipitation of warmest quarter; bio-19: precipitation of coldest quarter; Cond: conductivity (µS/cm); Lat: latitude (decimal degrees) ; Long: longitude (decimal degrees).
