# Supplementary material for: Population Genomics in Rhamdia quelen (Heptapteridae, Siluriformes) Reveals Deep Divergence and Adaptation in the Neotropical Region
Source: Genes (Basel). 2020 Jan 17;11(1):109. doi: 10.3390/genes11010109 (PMC7017130; doi:10.3390/genes11010109)
Supplement: Supplementary file 1 [file genes-11-00109-s001.zip › Supplementary File SVI.docx]

**
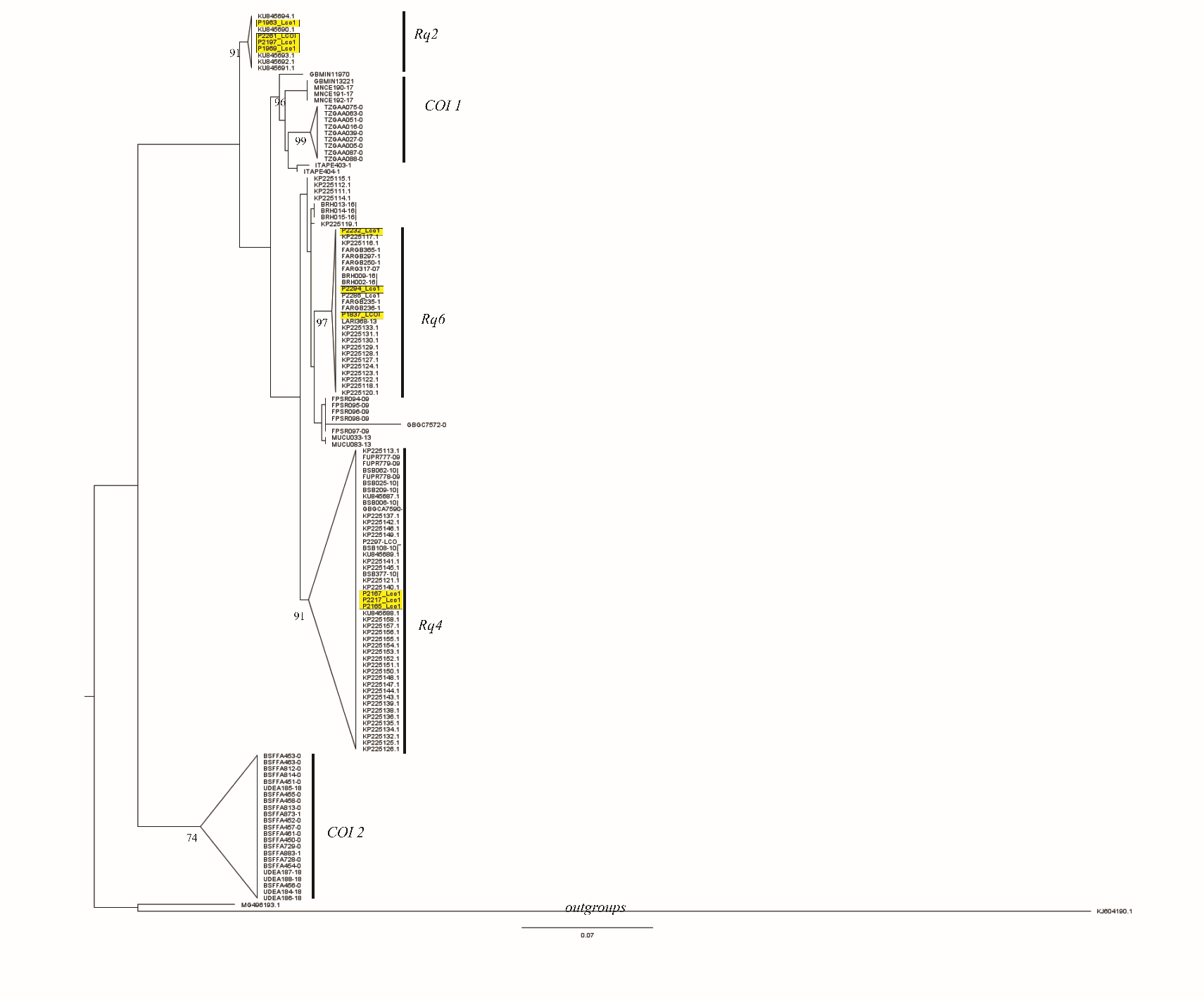
**

**Figure SVI 1.** Phylogenetic analysis of the genus *Rhamdia* in the Neotropical region. Tree topology generated based on 140 sequences of cytochrome c oxidase subunit I (*COI*) gene on PhyML 3.1 [37]. Numbers above nodes refer to the bootstrap support values from Maximum Likelihood. The bottom bar reflects a genetic differentiation of 0.07. *Rhamdia quelen* mitochondrial lineages are indicated and numbered (*Rq2*, *Rq4* and *Rq6* in accordance with Ríos et al. [14]; and *COI1* and *COI2* mtDNA lineages identified in this study). Sequences of individuals identified as *Rq2*, *Rq4* and *Rq6* mitochondrial lineage individuals in *cytb* phylogenetic analysis are highlighted in yellow. *Imparfinis mirini* (KJ604190) and *Pimelodella chagresi* (MG496193) (Heptapteridae, Siluriformes) sequences were used as outgroup.
