# Supplementary material for: Population Genomics in Rhamdia quelen (Heptapteridae, Siluriformes) Reveals Deep Divergence and Adaptation in the Neotropical Region
Source: Genes (Basel). 2020 Jan 17;11(1):109. doi: 10.3390/genes11010109 (PMC7017130; doi:10.3390/genes11010109)
Supplement: Supplementary file 1 [file genes-11-00109-s001.zip › Supplementary File SVII.docx]

**Table SVII 1**. Details of the 17,559 SNPs loci and their specific RAD-tag sequences.

| Locus | SNP position | Alleles | RAD-tag |
| --- | --- | --- | --- |
| 1_31 | 32 | [A/C] | CAATTCTTCGCTGCAACTTGATGCTTCCTGTAAGAC |
| 10004_19 | 20 | [G/A] | CGTTCACCGTCTGCAAGTTGTTGCGGTTAAAACGAT |
| 10007_31 | 32 | [G/T] | TATCATGGTCTGGCATTGTTTTGCTGCATCTGGGCC |
| 10008_33 | 34 | [C/T] | GCACACACACATGCACAGTTTTGCCCTGCTGCTCTA |
| 10009_11 | 12 | [G/A] | GAAATTGAAGCGGCACAGATATGCAGCAGCATGCTG |
| 10014_20 | 21 | [A/G] | GGAGGCACATTTGCAATGGTATGCCAGAGCACACAC |
| 10015_24 | 25 | [T/C] | GAATGGAAACGAGCAATAAATTGCCCGACCGTCCCG |
| 10016_18 | 19 | [G/A] | CAGACAGGTGAGGCATCTGAATGCAGAGTTGCATTT |
| 10018_9 | 10 | [G/A] | CTGCGTCACGTCGCATTTCTCTGCAGGAAGTCTGAC |
| 10021_26 | 27 | [C/T] | AAGCAAAAACGTGCAGAGAGGTGCCTCCAGGACCAG |
| 10022_32 | 33 | [C/T] | GTGAGTAACACTGCAAAGTTTTGCAGCTGAGACACA |
| 10023_34 | 35 | [A/G] | AAGTGTACAGCAGCAACACTGTGCTTCAAGCACGAA |
| 10028_28 | 29 | [G/A] | ATCCGGACCAGCGCATCAGGATGCCGGTACGACGCG |
| 10032_17 | 18 | [C/T] | AACAACCCCGCTGCACTCGATTGCATTTGATGCACA |
| 10034_19 | 20 | [A/G] | GCTGCTTGTGTGGCAGCCGAATGCACACACATAGGG |
| 10035_16 | 17 | [T/C] | AAGAGGAATAGAGCAGTGCGCTGCAGTATGTTTACT |
| 10038_3 | 4 | [G/A] | AGGGATCTAATTGCACAGGTCTGCCCTTCGCCTCCG |
| 10041_2 | 3 | [T/C] | CTTTATCCTGGTGCAGTTGTCTGCGCTGTGTCTGCA |
| 100418_7 | 8 | [C/T] | GACACGACGCATGCAGACAAATGCAAAGTGTATTCT |
| 10044_20 | 21 | [G/A] | CAGAGGAAATTTGCACAGTCGTGCAAAAAACTCTTA |
| 10047_8 | 9 | [G/A] | AGAAAAGTGACAGCACAGCAGTGCATCATGTCCAAC |
| 10051_25 | 26 | [C/T] | CAAGACAAACAAGCACATCGGTGCCCGCCTTAACGT |
| 10053_24 | 25 | [T/C] | TTAGGGCAGATTGCAGTTAGGTGCTGCTAATGGTGT |
| 10055_25 | 26 | [G/A] | TGAGGTCAGAATGCATTAGCATGCAGCGACCAGGAG |
| 10060_3 | 4 | [G/A] | CTGGAGCAGCTGGCAGCCCAGTGCTTCCGAGGAATG |
| 10064_30 | 31 | [C/T] | AAGGACATATGTGCACAAGCATGCGGACACCCAACA |
| 10065_5 | 6 | [G/A] | GGTTCGCAACACGCATGCGACTGCGCTCGTAATCCG |
| 10067_25 | 26 | [T/A] | CTGCCTCGCTGTGCATCACTCTGCATAACACTGAAT |
| 10069_19 | 20 | [G/A] | CAACAATATTTTGCAACAAGCTGCCTTGTCAGCATC |
| 10074_4 | 5 | [C/T] | GCATCCTTAATGGCACCGTGCTGCTCGACATGAAGG |
| 10075_20 | 21 | [T/C] | GAGACCCGACGAGCAAAGCACTGCCTAAAGGAAGCA |
| 10080_24 | 25 | [C/T] | CCACAGACAAACGCACCCAGATGCCTGGAGCTGTGC |
| 1009_3 | 4 | [G/A] | TGAGAGCTCTGAGCAGCCCTGTGCCATTGTATGAGG |
| 10091_26 | 27 | [G/A] | AGGATAGGCAAGGCAGGCAATTGCTTGAGGCCCCAA |
| 10094_4 | 5 | [A/G] | ACCGACAGGCGTGCAACACCATGCACTAATATGTGG |
| 10097_4 | 5 | [A/T] | GGCAAGCTTTCAGCACTCGTTTGCTCAATGACTGAC |
| 10099_8 | 9 | [C/G] | AACATGCCCTTGGCACACAGCTGCGACGCTCTATCA |
| 101_5 | 6 | [T/C] | ATCTGTCATTCTGCAGTCAACTGCTGCCCTGCTGTT |
| 10100_3 | 4 | [C/T] | CGTCGCTGATCTGCATCAATGTGCAGTCTTATCCCG |
| 10101_26 | 27 | [G/A] | CACCCCAAGTATGCAGGTGCATGCTGGAAGATAACC |
| 10104_10 | 11 | [G/A] | TTTGATTTCGGAGCATTACACTGCCAGGATCATCGC |
| 10107_8 | 9 | [T/G] | CGCTCACCTACTGCAGAGAAGTGCGAGGTGAGGTGA |
| 10109_25 | 26 | [T/G] | TGCATGCCTTTTGCATTGCACTGCTGCCCCATAAAT |
| 10115_11 | 12 | [G/T] | TTGCCACCTATGGCAACACTATGCCACCTATTTTAT |
| 10147_16 | 17 | [C/T] | ACCACTGGTGAAGCAGCCACATGCAAACGGTCATAA |
| 10154_27 | 28 | [T/A] | GCTCAATCAGAAGCAAGTGGCTGCCGGTGAACAGAC |
| 10157_27 | 28 | [A/G] | GTGCAGAGCAGAGCAAAGTACTGCCTTAACTGGATA |
| 10168_30 | 31 | [A/T] | ACTGATCGTCTGGCATGTAATTGCACTCAAAGAGTG |
| 10171_17 | 18 | [T/C] | TTGGCTAATAATGCAGGTGCGTGCGCGCGTGCTTTA |
| 10172_31 | 32 | [T/C] | GCCGCCCTAATAGCACTGCTTTGCTCTGTTGTCTTG |
| 10183_26 | 27 | [G/C] | TACCGCCTCAGTGCAATAGACTGCAACCGTACGTGC |
| 10187_15 | 16 | [C/T] | TGTTTTTAACCTGCACGACCTTGCCATGTTTGGTTT |
| 101886_6 | 7 | [C/T] | TGACCCCGAAGGGCATTTGGGTGCGTGTTATGTCAG |
| 10189_34 | 35 | [A/G] | AAAAGCGTGTTTGCACGTGTTTGCGTGTGTGTGTAT |
| 10190_7 | 8 | [G/A] | TGCAAATAGACCGCAACCGATTGCAGCTTATACTAG |
| 101949_19 | 20 | [A/T] | TTTCTGTGCGAGGCAGGTTACTGCTCTGGTGCTCCA |
| 10201_17 | 18 | [C/T] | TTCACTGTCAAGGCACCCGGCTGCCAGGAAAGACTC |
| 10204_30 | 31 | [T/C] | TCTTCACCCTAAGCAGGTTGGTGCCTCGATTACACA |
| 10206_9 | 10 | [C/T] | CTCGTTTTCCGAGCAAACAAGTGCCAGTAAAGAGGC |
| 1021_33 | 34 | [A/C] | CCATCAGAGACAGCAGGTAAGTGCACTGGATATCCT |
| 10211_19 | 20 | [A/T] | CTTCTGAACTCAGCACATCACTGCTGCTGGACATCG |
| 10212_11 | 12 | [C/A] | AAGCTTCAGAACGCAAAAATCTGCCCGGATTACACT |
| 10216_34 | 35 | [C/T] | ATTCACATCCCAGCACCTCTATGCTGGACTGAACTG |
| 10220_6 | 7 | [T/C] | GAGGAATGGACAGCAAGCAAATGCATCTCTGTGTCT |
| 102231_16 | 17 | [C/T] | CCTGTCATTGCTGCAGCTTTGTGCTGTCTGTTAGGA |
| 10224_17 | 18 | [A/G] | CATTATCAGCAGGCAGCAAGATGCTTGAGCTCAGTT |
| 102267_2 | 3 | [C/T] | ACTGACCTAATGGCAGATCACTGCAGCTCTACTGCT |
| 102273_11 | 12 | [C/T] | TTGTGCCTTGACGCAAGGAAATGCCAACTGTTATCA |
| 10238_30 | 31 | [T/G] | GCGTTTGTGACTGCAGCGTGATGCATTCACGATATG |
| 10241_25 | 26 | [C/T] | GGGTGTACATCCGCAAAGGACTGCCCCCTTCTCACG |
| 10247_3 | 4 | [C/T] | TATTATGGCTGTGCAGCTGTTTGCAAGCTTTACATC |
| 10249_2 | 3 | [C/T] | TTCACTGATCCAGCACTCTGCTGCTCAGGCAGGAGT |
| 10255_26 | 27 | [A/G] | CACACACAACACGCATTTTCATGCAGACAACAGCCC |
| 10258_5 | 6 | [C/T] | GACCTCGTACATGCACCGCCGTGCCTCAGTAGCTTC |
| 10259_1 | 2 | [C/T] | TCTCTGCCTTTAGCACATTCGTGCAGTGAAACACCG |
| 10272_19 | 20 | [G/A] | GAGCTGTGGTTTGCAGCTCGGTGCTGACGACCTGAG |
| 10275_8 | 9 | [A/G] | CGAGTACAAAAAGCACTCATCTGCAGAACTGCTCAT |
| 1028_2 | 3 | [C/G] | TGCGGGCATGTGGCATGAATGTGCATGTCTGGCTTT |
| 10284_34 | 35 | [C/T] | GGGTCTTCTGCTGCATGCTGCTGCTCTGGTTCCTCT |
| 10286_4 | 5 | [A/G] | TAATAAAGGGAGGCACTGTGCTGCAGGTAATGCATG |
| 1029_24 | 25 | [A/T] | GATTTATGCATGGCAGGATGATGCATGTGGGTTGAT |
| 102963_3 | 4 | [C/T] | GTGCGTGCGTACGCAATCAGGTGCGGACCTGTCCGA |
| 10300_30 | 31 | [G/A] | AATTTGGGCACTGCACAAAAATGCAGACACGCAGCA |
| 10303_19 | 20 | [G/A] | TATCACAAAGCTGCAATGCGGTGCTGCTGTTAATCT |
| 10304_9 | 10 | [T/C] | TGACGAGGATGAGCACAGAGCTGCAGCTGGGCAGAA |
| 103051_32 | 33 | [C/T] | TGATCATCTAAAGCAACGTTTTGCTTAACGCGTGTG |
| 103056_3 | 4 | [A/G] | GACGGCACACTGGCACACTAGTGCGTCACATTCTCT |
| 10307_8 | 9 | [A/G] | GTGTCTGCATGTGCAACCTAATGCCCAAATAAAGCT |
| 10313_34 | 35 | [A/G] | TCATATTTCACTGCAGGTTCATGCAGCCTGCCTTAG |
| 10315_33 | 34 | [T/G] | CCCCACCCACTAGCATTTCACTGCCGCTCTTAGTCT |
| 10322_26 | 27 | [G/A] | GTGCACAATCTCGCAGAGGCCTGCTCGAACCAAGGC |
| 10330_9 | 10 | [A/G] | GCCCTGTCCAGGGCATATTCCTGCCTTATGCCCAAT |
| 10331_15 | 16 | [A/G] | TGTAAGATCTGAGCAAGGAAGTGCTAACCGCTACAG |
| 10337_7 | 8 | [G/A] | CACTAACGAACTGCACACAGATGCAGCACATGCAGA |
| 1034_19 | 20 | [G/A] | GTGACGTATGCGGCATATTGATGCCATCTGCACGTT |
| 10343_28 | 29 | [G/A] | CAGCCTGACTGTGCACGCTATTGCCACGGGCATCTA |
| 10344_29 | 30 | [C/T] | GTTTGGCTTGCTGCAAATTGCTGCATCTTCACTGAA |
| 10345_5 | 6 | [G/T] | TAGAAGGTGAGAGCACTTACTTGCTTGTGCATTTCG |
| 10362_18 | 19 | [A/C] | ATGTTTGCCCTTGCAGCAAATTGCCTCAGTAGATTT |
| 10363_19 | 20 | [A/T] | CTGAGATAAAATGCAACTCACTGCTGCCACTTGACG |
| 10366_33 | 34 | [T/G] | ACATGGTGACATGCAGTGTGTTGCATGTTAGTCTGT |
| 10369_24 | 25 | [T/G] | AATTTGGAACAAGCAGGGAGTTGCGGAAGCAGCCAT |
| 10378_18 | 19 | [G/A] | ATCAAGTAACCTGCACTCGGTTGCAACTGTACGAAG |
| 10386_15 | 16 | [C/T] | GGTCACTCATCAGCACGTGGATGCGGTTACTAAGCA |
| 10389_15 | 16 | [C/A] | TTGTGTGTGCTGGCACATTTCTGCATAAAACAACCT |
| 10393_28 | 29 | [C/T] | GAACCATCGCCTGCAGAGAGGTGCGTAACGCCCATA |
| 10394_32 | 33 | [T/G] | AGCATCCCCACAGCATGACACTGCCACCACCATGAC |
| 10395_30 | 31 | [G/A] | ACACACCTGATTGCACACACATGCAGTAGTGAACAC |
| 10398_29 | 30 | [T/C] | CACAAGCCAACTGCAATTTACTGCATCCTCGTGTGT |
| 10399_2 | 3 | [G/T] | AAGACAGAAAATGCAACCCAGTGCCGTTATCTTGTA |
| 104_2 | 3 | [G/A] | TGGAGCGATTTTGCATTGGTTTGCAGTTGTATGTCA |
| 1040_5 | 6 | [A/C] | GGGCAATTTAGGGCATCCAATTGCACCCTCATAGAC |
| 10408_5 | 6 | [C/T] | AAACCCCGGTGTGCATTGCATTGCACTCACTCACAC |
| 10411_6 | 7 | [G/A] | GGCAGCGGCAAGGCAATTTCCTGCACAGGAAAGTGC |
| 10412_17 | 18 | [A/T] | TCCTGACACCTTGCAGCAGCATGCACCTTAACGGTA |
| 10413_29 | 30 | [G/C] | TTGTGCCACCCGGCATCTTCTTGCACAAGGGGTGCC |
| 10416_33 | 34 | [C/A] | TACTGTTTTTCTGCATCGGGTTGCCATGTGTACCAA |
| 10419_8 | 9 | [G/A] | AACAAGGCGAATGCACAGGTTTGCGATGATTTAGCT |
| 10426_30 | 31 | [T/G] | CTGAACAATGAGGCAGCAACATGCATAGGGGTTTTT |
| 10427_27 | 28 | [G/A] | AAAGGTGATCCTGCATAACAATGCCTGGGCTCTCCT |
| 10431_34 | 35 | [G/C] | CTACATTTAGTCGCAGTGTCTTGCAAACTTCTCGGT |
| 10432_11 | 12 | [C/T] | GGTACCACATACGCACCCGTTTGCTTACAGCAGCTG |
| 10433_16 | 17 | [A/G] | TTTTTTTTTGGCGCAGATAATTGCAGTCGCTCTTAC |
| 10435_2 | 3 | [G/A] | TAGACACGTGGAGCAGAGTGCTGCATTTTAATAGAA |
| 104507_19 | 20 | [C/T] | CTGTATTTAATCGCAGGCATGTGCTAGGCATCCCGT |
| 10454_31 | 32 | [C/T] | GTTTTACTGGCGGCACCAAAGTGCTCGATTCCCGGT |
| 10464_17 | 18 | [A/G] | CTTTCTCTCTGTGCACAGTGCTGCAACAGTGGTAAG |
| 10469_30 | 31 | [C/T] | CATCATCCTAACGCAGGGTAGTGCCGGCAGCGTACA |
| 10477_6 | 7 | [C/T] | TCTCACCGAGATGCACTTAAGTGCTTTCCGTTGTGT |
| 10478_6 | 7 | [A/C] | GTGAGGAAAGGGGCAAGCTGGTGCTCTTGGAGCAGG |
| 10480_34 | 35 | [A/G] | GCCAATCCGAACGCACTCTACTGCTCTCTTGGCCAG |
| 10491_26 | 27 | [C/T] | TCTACTTTGGGTGCACTGACATGCATCCCTCACATA |
| 10492_34 | 35 | [G/T] | TGTGTAACATTGGCAGACTGATGCGGATCTGCGCGC |
| 10494_10 | 11 | [G/A] | TGCAACAGCTATGCAGGCACCTGCTAACAAATGCTT |
| 10495_8 | 9 | [C/T] | TGTGTGTACGGAGCAGCTTTTTGCTCCCACAGGCCA |
| 10498_31 | 32 | [C/A] | TTCATCCTCGCAGCACTCAAATGCTCCATGCCCTTT |
| 10502_28 | 29 | [G/A] | GTTTGTTTGTGTGCATGTGTGTGCGCACGTGCTATT |
| 10508_4 | 5 | [C/T] | TGTTCTCCAAGCGCACTGTGATGCTGTTAGTCAGCA |
| 10509_8 | 9 | [G/A] | CTGACTTTGACTGCATCCCAATGCACCAAATGTGCT |
| 10512_17 | 18 | [C/T] | CTGGGGTTCACTGCACTCCATTGCTGGAGAGCAGGT |
| 10517_25 | 26 | [G/C] | TATCTGACTCCAGCATCGTTCTGCTGTTACTATTGA |
| 10524_20 | 21 | [C/T] | GACTCCTGACTTGCAAATTCCTGCTTTCAACCCATC |
| 10525_17 | 18 | [A/G] | ATCACTGACAAAGCAGCACACTGCTCTCACAACTCT |
| 10526_18 | 19 | [C/T] | GTGTAACTGCCAGCACCATCATGCTGCTCTCTGTAT |
| 10527_5 | 6 | [T/C] | GTCTTTGGGTGTGCAAACTTTTGCACCTCATGCAGT |
| 10535_5 | 6 | [G/A] | TGGGAGGTCCCTGCAGAAATCTGCTGAATGGCCACT |
| 10536_1 | 2 | [C/T] | ACTGGCTGGAGCGCACCGGGCTGCCGCAGTACGAGA |
| 10539_19 | 20 | [G/C] | CCACGTGATGACGCACGGAGATGCTGCCACTGTCTG |
| 10542_15 | 16 | [C/T] | GCGGAGCTCTTTGCACTTTGCTGCCTCGTGTGGTCA |
| 10546_24 | 25 | [T/C] | GAACACACGCACGCACCAATCTGCTGTGCCGTTGTT |
| 10547_34 | 35 | [C/T] | CATGGAGTGACTGCAATATTGTGCTGCTGGGTTTCA |
| 10548_34 | 35 | [A/G] | AAAGTTGCTAAAGCACGCCGTTGCTAAGATACCGAC |
| 10550_8 | 9 | [G/A] | TCGGCTCAGGCAGCAGATACATGCCACGGACATTCT |
| 10551_17 | 18 | [T/C] | GTATGCACGTACGCACATACGTGCAGAGAGATGAAC |
| 10552_10 | 11 | [C/T] | CACACTGGTACGGCATGTCCATGCATCCATCATCTT |
| 10553_10 | 11 | [C/T] | TCTGTAATCCCGGCACCGTTGTGCAGTTTTCACTGT |
| 105534_30 | 31 | [A/T] | CAAATCTGTCCTGCATTGCTCTGCACCCAATGCTCA |
| 10556_33 | 34 | [G/A] | AGAGACAAAACAGCACCAGCTTGCCAAGAAATGGAG |
| 10558_7 | 8 | [C/T] | TTCTGTTCCATGGCAGTGTAATGCATTCCAAGGTTA |
| 10559_26 | 27 | [G/A] | TGGTGCCAGAGAGCAACAGTTTGCTGAAATATGTAT |
| 10561_32 | 33 | [T/C] | CACTACCCTGTGGCACCCCCATGCCACCCGCATGAT |
| 10565_2 | 3 | [A/G] | CGACGAGCGACAGCATCTTCATGCCTCACCGTAGCA |
| 10566_29 | 30 | [T/C] | CTCCAAAGAGCTGCAGCAAGCTGCAAGCATGCCACT |
| 10569_32 | 33 | [G/A] | TGAATCGTGAAAGCAGCAGTCTGCCGTTCCTGGAGC |
| 10570_1 | 2 | [C/T] | ACTTTGTCTTCTGCATGCTGCTGCTACAGTGAGAGG |
| 10571_17 | 18 | [A/G] | TTGGCAGGCTGAGCAAAAACCTGCAGAGGAACACGG |
| 10576_28 | 29 | [C/T] | GGCTGGGGCGGTGCATCACTCTGCGTTTCTCTCCCG |
| 1058_3 | 4 | [G/T] | TCGGCTCCTGCTGCACAGATGTGCGCGTAAACGTGC |
| 10583_19 | 20 | [G/A] | TGATAGTGACATGCACCTCGCTGCTCCAAGTCTTAG |
| 10586_29 | 30 | [G/A] | AGTGTTCCTGCTGCATCGTGATGCCCTTTGAAAAAT |
| 10588_18 | 19 | [C/T] | CAATAATCCGTCGCAGGACGTTGCATCGCGCATCAT |
| 10590_19 | 20 | [C/T] | ATGCTTCCTGCAGCACCGCCGTGCCGCCGTCCCTTT |
| 10594_31 | 32 | [A/G] | CCAAGAGCTCCAGCAGACTCGTGCTGACGCAAATGC |
| 10595_26 | 27 | [C/A] | GACGTGTTGGCAGCATGGACGTGCGGCAGATTCCCA |
| 10597_10 | 11 | [G/A] | AAAAGGCCCAGAGCATCACAGTGCCACCACCATCAT |
| 10600_33 | 34 | [T/C] | TGCCAGAGTTGAGCAAGGTGGTGCAAACATGAACGC |
| 10606_3 | 4 | [T/C] | GACTGATCACATGCAGAAATGTGCAGGTCATGCGAG |
| 10607_31 | 32 | [C/T] | CAATGACAGTCTGCATGATGATGCGATGATGCGCCG |
| 10610_11 | 12 | [C/T] | AAGAGCTGGTACGCATGAGACTGCGACAGGAGCTTC |
| 10614_4 | 5 | [C/A] | ATATCAAGTCATGCATGCTGGTGCCATTTGTACACA |
| 10621_2 | 3 | [G/A] | CTGAGACAGGGAGCATTATCTTGCTGAAAGAGGCTA |
| 10623_15 | 16 | [T/C] | CAGGCCCCATGAGCATGGCCTTGCCTTTAACCCTCA |
| 10624_24 | 25 | [T/G] | CATCAAAAGTGTGCAGGCTTTTGCTACTGTTTTGCA |
| 10626_10 | 11 | [C/T] | AGGTCAAGCCCGGCAGAGTGATGCGGATCTGTCACA |
| 10633_18 | 19 | [A/T] | GGACCGTAAAGTGCAGAAAGATGCTGCCGTGTCACT |
| 10634_7 | 8 | [G/T] | ATACTCAGTGATGCAGCACAATGCCCAAGAGCTTCA |
| 10637_20 | 21 | [T/G] | TGTCGCTGAATGGCACTGGTTTGCGACTGAGCGTGT |
| 10642_30 | 31 | [C/G] | TGTACAGACTCAGCAGAGCGTTGCTGAACACTGACA |
| 10643_24 | 25 | [A/G] | TTCCCCCCTGTAGCAGGGCATTGCAGTGGTTAAAAA |
| 10650_17 | 18 | [C/A] | CATGATCGGGTGGCATCCAAATGCTACGAGGCTCCG |
| 10653_25 | 26 | [T/A] | GCCTCAGCCTGCGCACATCAATGCATTTCATTTGTG |
| 10656_17 | 18 | [T/A] | TTTTCTTAGATAGCAAGTCACTGCCACTGAGCGCTA |
| 10658_25 | 26 | [C/A] | GTGTGTGTGTGGGCACGTACATGCAAGAACACAGGT |
| 10659_1 | 2 | [A/T] | AAGTCTAGAAAGGCATGTCTGTGCACCACAGAAGAG |
| 10669_25 | 26 | [C/T] | ATTAGTCAGTAAGCAAGTGAGTGCACGGCAAAATAA |
| 10674_2 | 3 | [G/A] | CTGGGGGCACGTGCATATGTGTGCATGTGGGTGTGT |
| 10677_31 | 32 | [G/T] | CAATTATGTTGTGCATGGACGTGCAAAACAGGTAGT |
| 10678_8 | 9 | [T/G] | GCCTGACCTGCTGCATGCTGCTGCGGATAGAGCTAG |
| 10680_31 | 32 | [C/T] | GAATGACCTGCAGCAGCTGTTTGCATTTTCTCATGA |
| 10681_28 | 29 | [G/A] | ACTGGCTGTCTTGCACACACCTGCATTCGTTTGGTG |
| 10685_25 | 26 | [T/C] | CCAAACATTACGGCATCAAGGTGCTTGTCCCATTCT |
| 10689_28 | 29 | [T/C] | ATGTTGCACACTGCAGTATATTGCTCAATGTTCCCC |
| 10691_9 | 10 | [C/A] | CTGCCTTAACTAGCATCCTACTGCCCTACACTAAGC |
| 10694_19 | 20 | [C/T] | ATAGGGTCATGTGCACTTACGTGCTTAAGGGTGTAA |
| 10695_7 | 8 | [A/C] | CTTTAGCATAAGGCACCAGAGTGCCACAAGTGATGT |
| 10697_17 | 18 | [G/A] | AGCATGGTGGTGGCAGCGTTATGCTGTGGGTACGCT |
| 1070_18 | 19 | [A/T] | TCCGCTGAACGAGCAGCGATCTGCTTAAGCTACGCC |
| 10701_16 | 17 | [G/A] | ACTGCCATCTTAGCAGGCTGGTGCTGACTGAAAGAC |
| 10703_4 | 5 | [C/G] | GGCACACGCCCAGCACATTTGTGCACATCTTTTCAT |
| 10708_24 | 25 | [A/G] | CTGGGTAGATAAGCAGAAGCCTGCACTGGTCAGTGG |
| 10709_10 | 11 | [A/G] | ACATGTACAGACGCACTGCACTGCTGCGAGCTCAAC |
| 10710_25 | 26 | [G/A] | GATAGTTTGCCTGCATGTGCATGCTGATCTGGCATG |
| 10713_20 | 21 | [A/T] | ACACACCCTGATGCAGTGCTATGCGCACTACATGCA |
| 10714_30 | 31 | [C/T] | ATTAATAGATGAGCAAAGCCGTGCCAGTCACGCTGG |
| 10715_24 | 25 | [A/G] | ATGGACGAACATGCAAATTTGTGCATACAGACGTTA |
| 1072_31 | 32 | [C/T] | ACAAAGATGTTTGCAAGTATCTGCAGCTCCGCAACT |
| 10723_6 | 7 | [C/A] | ATCATCCTTGGTGCAAAGGCATGCTAAGGGTAACTG |
| 10729_1 | 2 | [C/T] | CCGTAACGGAGAGCATGACACTGCGAAGGCACACCG |
| 10733_4 | 5 | [G/A] | CCGCGTCCGCACGCACAAGCATGCCGCTTTACTGTG |
| 10736_10 | 11 | [T/C] | AGCGCGCATCTGGCACGCGTGTGCGCGCCTGCTCTA |
| 10739_18 | 19 | [A/G] | TCCACAGAACTTGCACTGAGTTGCCTCTGTGCCTTG |
| 10743_11 | 12 | [G/T] | TCTCCCTTCATGGCAATAAGCTGCGTCTCAAACAAC |
| 10746_32 | 33 | [G/A] | AATGGTTTCAGAGCATTAAATTGCCAGTGCATGGAC |
| 10754_2 | 3 | [T/C] | CATTCGACCTGAGCAGACAGCTGCACTCCATGTGCA |
| 10757_11 | 12 | [C/T] | CGCAGTTGACTCGCAGAGGCCTGCAAGGTCAGCAGG |
| 10758_27 | 28 | [T/A] | AGCTAACTGGGTGCATTTTCCTGCTGCAAGTTCACC |
| 10759_32 | 33 | [G/A] | AAACTGTCATCAGCAATCAACTGCTCATCTGTGGGG |
| 1076_34 | 35 | [T/A] | ACACTACCTGCAGCATCATCGTGCCACAACACAGTT |
| 10766_33 | 34 | [T/A] | CATGGCTGGCCTGCATTTCTCTGCTCCACTGGATAT |
| 10768_16 | 17 | [A/G] | TGAGTTTGGGTAGCACAGTGTTGCCGCAGGTAGTGT |
| 10772_8 | 9 | [C/T] | TGCAAAGCCCTGGCAGATTTCTGCTTTCAGATGAGA |
| 1078_26 | 27 | [T/A] | AGGCACGGATCTGCATTCCAATGCGTTTTTAATTCT |
| 10780_4 | 5 | [C/G] | CAGTCAATAATGGCAACTCCCTGCCTGTGCCCACAG |
| 10783_32 | 33 | [A/G] | GTAAAAACAGCAGCAGGACTTTGCCAAGTTCAAGGT |
| 10784_2 | 3 | [A/G] | GCACAGCAGTCAGCAAAAAAATGCATATCTGGCAGC |
| 10786_7 | 8 | [A/T] | TTGCAAAAGGTTGCAACGGTTTGCTATACGGATGTC |
| 10787_19 | 20 | [G/T] | TCATAGCATGCTGCAGCTCGGTGCTCAGCTCGCTAT |
| 10791_27 | 28 | [C/T] | ACAGCCTATCGGGCACTTCATTGCATTCTCTTACAT |
| 10792_3 | 4 | [G/A] | GTTGTGTGGGCAGCACGGTTATGCCTGTATCATATG |
| 10798_33 | 34 | [C/T] | TACGAGTCATTAGCACTGATCTGCAGGAGATCTCCT |
| 10803_15 | 16 | [G/A] | CTGCCTGCTTTGGCAGAAACCTGCACTCGGTTCCCT |
| 10818_1 | 2 | [A/G] | CAGGTTCATTAGGCAGGAAGCTGCTGGCCCAAACCT |
| 10822_5 | 6 | [G/T] | TCTTTGTTGGTTGCACACCATTGCAGGAGTGCTTAC |
| 10824_29 | 30 | [A/G] | GGAGCTTTACTTGCAGCCTCTTGCCCGCGAAAAAAA |
| 10826_3 | 4 | [T/C] | AGATTTAAGACAGCAGGGAACTGCAGGGCTATTTAC |
| 10828_2 | 3 | [A/G] | GGAGCCCACTTTGCATCTTTGTGCGCAGGTCATGGA |
| 10830_19 | 20 | [G/A] | GCAGAAAGTCTAGCAGTCGGCTGCAAGGAAAAAATA |
| 10832_27 | 28 | [C/A] | CGTGATTGGCTAGCATCACTATGCTAGCCAGGAAAT |
| 1084_33 | 34 | [T/A] | ACAAATGCACAGGCAGCATGCTGCAGCTTCTCTTCA |
| 10843_16 | 17 | [G/C] | TGGAAAGGGAATGCACGATGCTGCCTGACTCTGCCC |
| 10845_8 | 9 | [T/C] | TCTGTCTCTTTCGCATTCTGTTGCATGCTCTATTTC |
| 10848_25 | 26 | [T/A] | GGTTCTCCTGTGGCATGAAGCTGCGTGGGAACGTGC |
| 10849_10 | 11 | [C/A] | TCATACTGCTCGGCAGCCGGATGCCATGGAAACAGA |
| 10851_30 | 31 | [G/A] | GCACTAGCTATGGCACCACTGTGCCACCCTGCTTTT |
| 10854_34 | 35 | [G/A] | ACTTCCCATTGTGCAACTTTGTGCTTGCGCTGCAAA |
| 10857_7 | 8 | [T/A] | TGAGGGATTTCTGCACCGGGCTGCGTGTCCCGATTG |
| 10860_2 | 3 | [G/A] | TGGGATAAAACGGCATCCTATTGCGTCGCATCTGCC |
| 10861_34 | 35 | [G/A] | TGTAGAGAGTCAGCAAGCTGCTGCTGTTAATGTTGT |
| 10862_5 | 6 | [T/C] | TTTTCTGGATGGGCATCAACATGCACAATGCCTGCG |
| 10866_15 | 16 | [T/A] | GATGGCAATTATGCATAGTACTGCCACCACAGACTA |
| 10867_6 | 7 | [T/C] | AACACACGCGGCGCACACGGCTGCTGTGCCTAATGG |
| 1087_26 | 27 | [C/T] | GTCCCCCCAGAGGCACTGATCTGCCTCAGACATGTT |
| 10874_4 | 5 | [T/A] | CGGTTAACTTCAGCACCACACTGCTCACCTTAAAAA |
| 10877_30 | 31 | [G/A] | ACGGATATCTGGGCAGGTCTGTGCGTTCATGCATGC |
| 1088_15 | 16 | [T/A] | CAATCCTGTAGTGCATGGGCGTGCTGTATGTGCTTT |
| 10880_19 | 20 | [C/G] | TCCTGCAGTAGTGCAGCGCCGTGCAGCCCCAGACGC |
| 10883_5 | 6 | [G/A] | ACATTGAACTTAGCATCACACTGCCTGGTGCCATGC |
| 10884_32 | 33 | [G/A] | TCTCTTTCTCAAGCACAGATGTGCACCGACTCGCTC |
| 10885_1 | 2 | [G/T] | TGTGTTGGTGTTGCATTTTTATGCCCGAGGAGGTCC |
| 10886_15 | 16 | [C/T] | GAGTCTATTTCAGCACATTCTTGCCTCCAGCCAACA |
| 10890_33 | 34 | [G/A] | GTAGGCGACAAGGCAGGGTGGTGCAGTGTGTACGTG |
| 10892_11 | 12 | [T/C] | CAAGCAATCCCTGCAGCTACCTGCCATCGTTTCTTT |
| 10902_32 | 33 | [C/T] | GATTGGCTAGGAGCAGTCATGTGCAGAAAGAGCCAA |
| 10904_1 | 2 | [G/A] | TGATCTAATCTGGCACACACATGCACACACACACAC |
| 10908_18 | 19 | [C/T] | TTGTGAAATGGTGCATTACCCTGCTAGAAGTAGCCA |
| 10909_18 | 19 | [G/C] | GGACACACGTGAGCAGGCGGCTGCACTGGTGACGTG |
| 10912_5 | 6 | [T/C] | TTAACTGCCCAAGCATGAAATTGCGGGCCTGCAAAA |
| 10916_33 | 34 | [G/A] | GATATTGCTACAGCATCACAGTGCACACACAACGCA |
| 10920_11 | 12 | [C/T] | TATAAATCCAACGCATGACCGTGCATCGTAGCCCAG |
| 10925_32 | 33 | [G/C] | TGTGGCAGGCTGGCACTCTGTTGCCCGATCGTGAGT |
| 1094_7 | 8 | [T/C] | CTTTTTGTCACAGCATTTCTTTGCATGGCCCACAGT |
| 10946_34 | 35 | [C/T] | CACTGACGTTCCGCATCGCAGTGCGCGTCCTGCTCG |
| 10947_9 | 10 | [G/A] | AAAACAAATGAGGCACTCGGATGCAAGCTTGAGAGA |
| 10948_5 | 6 | [G/A] | CGCTCGTATTGTGCAAGTAGGTGCACGTAAGCCTAG |
| 10950_19 | 20 | [G/A] | ACTGTGCGGGTGGCACGGCGCTGCTGCTGCAGCGAG |
| 10953_9 | 10 | [G/A] | TAGTTTTCTGCAGCAAGCACATGCGACCTGTGCTAG |
| 10955_24 | 25 | [C/T] | GCATGCCATCCAGCACCCGACTGCCAACAGCATCAT |
| 10959_15 | 16 | [G/T] | ACTACAGACGGAGCAGCCCACTGCTCAGTGCATGTG |
| 10961_26 | 27 | [C/G] | ATCTCCCCATTAGCACTGACCTGCCGCACACGGTTG |
| 10966_33 | 34 | [A/T] | ATCATGGTGTGGGCAGCATCATGCTGTGAGGGTTTT |
| 10968_19 | 20 | [A/G] | AGTGCCCAGAGAGCAATTAAGTGCCTTGCCCAAGGA |
| 10969_32 | 33 | [G/A] | TCATACACCTGAGCAGACCGCTGCCGTGGAGCGTGT |
| 10970_32 | 33 | [C/T] | AGCAGACAAACGGCATCGACCTGCCTCCAGCTCAGG |
| 10976_18 | 19 | [C/T] | CCAGAACACTTGGCAATTCAATGCTACAGCTCTGCA |
| 10981_10 | 11 | [C/G] | CCCAGCTGCGCCGCACAAAATTGCTTCAGAGTGGAA |
| 10984_32 | 33 | [T/A] | CCAGTATGGCTTGCATTTTTCTGCCTCCTCCCTGCT |
| 10985_6 | 7 | [G/C] | GAACCAGACACTGCATCACCATGCCACCTGCAAAGT |
| 10989_31 | 32 | [T/C] | GGAATAGAAAACGCAGAAAAATGCACACTCGTGCTT |
| 10990_2 | 3 | [A/T] | AGATCAAATGCAGCAGGGTCGTGCAGTTCCTGAACT |
| 10994_28 | 29 | [C/T] | CATTAGTGCCGAGCAGTGGGCTGCATTCCTGGGCAC |
| 10997_33 | 34 | [G/T] | GGTGATGTTTTGGCACTGATCTGCTTGTCCTTCGGA |
| 10999_4 | 5 | [T/C] | GTTCTTTAGAAAGCAGTGTGGTGCCGCTTTTTCTTT |
| 1100_4 | 5 | [C/T] | CACACCAGTCTAGCACAAGACTGCAGAAAAACATGA |
| 11003_8 | 9 | [T/C] | CTTTCTGCTCCAGCATCAATCTGCCAGCATGATATT |
| 11004_33 | 34 | [A/T] | TGAACCAAGTAAGCAGCTGAATGCAGAAAGACAAGG |
| 11005_3 | 4 | [C/A] | TCTAATGGCGGCGCAGAAGGGTGCATTTAGTTTTAT |
| 11009_15 | 16 | [C/T] | ATGTCCAGGAGAGCACGACCGTGCCGTGTCAGACCT |
| 11010_32 | 33 | [G/A] | TGTAATGCTACAGCAGCGAAATGCGACATGCTGAAT |
| 11015_26 | 27 | [G/A] | GTGAGTGTCGGAGCAGCGACGTGCCAGCAGAGTGGC |
| 1102_25 | 26 | [G/A] | GACTCACCAGGTGCATTTACATGCTGATCCACAGTT |
| 11020_8 | 9 | [C/T] | AAAAAGCTCGCGGCACCCTGCTGCATTTACACGCTA |
| 11025_10 | 11 | [T/C] | AAGATAGAGATGGCACGAAGATGCCAGAGGCTACAT |
| 11026_30 | 31 | [G/A] | CGTATGGTGGTGGCAGCATCGTGCTGTGGGGAGGTT |
| 11028_33 | 34 | [G/C] | AAACATGTTCTAGCATGACAATGCCCCTGTGCAGAA |
| 11033_3 | 4 | [G/C] | GTGGGAGGACATGCAGATCTTTGCTGTGTGTTTGTG |
| 11035_8 | 9 | [T/G] | TGTGTGTGTGGGGCACTTTACTGCAGTGCTGGTCTC |
| 11040_1 | 2 | [C/A] | ACTGGAGGCAGAGCAGTGATTTGCAATTCCATAGCT |
| 11041_4 | 5 | [A/G] | TTAGAGAGTCTGGCAGATGTCTGCTGAAGTATGAAA |
| 11043_11 | 12 | [C/T] | TCATGCAGCAGCGCACAAATCTGCTAAGGAAAGATA |
| 11049_8 | 9 | [A/T] | TGAGTGCGATTTGCATTTCGCTGCATGCCTTTTGGC |
| 11055_34 | 35 | [A/T] | CCGACTTCAGAGGCATGGGAATGCTTGGGCTGGTAA |
| 11059_1 | 2 | [C/T] | CTACCATCAGGAGCACCAGAGTGCCCGGTGTTCATC |
| 11071_27 | 28 | [G/A] | CCTGTTATTGATGCAGGCCTCTGCCTGGGCGTAGAG |
| 11075_4 | 5 | [T/G] | CAAGGTTAACTAGCAGTCTGGTGCCAGACAGTCTGC |
| 11082_27 | 28 | [C/T] | TGTATGTACTCAGCATTTCACTGCTGTCGACTACAC |
| 11085_18 | 19 | [T/G] | TAGACCGAAATGGCACATTCATGCATACAACCTTAT |
| 11086_2 | 3 | [C/G] | TTCAATCCTGTAGCACTTCTCTGCTGTCTCTCTCTA |
| 11094_27 | 28 | [C/G] | GCAGCGTCGGTTGCAGTTAATTGCTCGCTTTAATGG |
| 11096_9 | 10 | [G/A] | GTGACCACAGTGGCACAGTGGTGCTCTGGGTCTTGC |
| 11097_26 | 27 | [A/G] | TTCGGCAGGGCTGCAGGCTGTTGCGCACGCCAGTAG |
| 1110_32 | 33 | [T/C] | TGGCGACGTGATGCATAGCACTGCTGCTGCTCTGTG |
| 11100_29 | 30 | [A/G] | GCCAAAGTGCCTGCACCTGTTTGCCAGTGACTGCAC |
| 11111_28 | 29 | [T/C] | GACGGTGTGAGAGCACACATCTGCAACCCGACACAC |
| 11119_2 | 3 | [G/T] | CAGTACATCCCAGCACAGCCATGCCAGGCACCAACG |
| 11120_30 | 31 | [T/C] | GTGTCTACAGCTGCAACCTACTGCTGTTCTTTACAG |
| 11122_15 | 16 | [G/A] | TCTCAAAGTCTTGCAGAACGCTGCACATGCCAGCCT |
| 11123_10 | 11 | [G/A] | AAGAACAGACGTGCAGCTGTGTGCTACAGCTATCTC |
| 11132_2 | 3 | [C/G] | GTCCTACATGCAGCACCACTATGCAGCCAAGATTAT |
| 11137_24 | 25 | [G/A] | ACAGATATCACCGCAAAGGAATGCGCCATTGTGTAC |
| 1114_3 | 4 | [A/G] | ACAAAGTGACATGCAGGGGAATGCACATAACGAGCA |
| 11141_8 | 9 | [G/A] | GCGGGTCCGAGCGCATGGTGCTGCTCAGCTCAGCTC |
| 11145_7 | 8 | [G/A] | ATCCTTGGATTTGCATCCAGTTGCTTAGCCAATCAG |
| 11148_28 | 29 | [C/T] | AGAGCACACTGAGCAGCAGATTGCTCCTCTTCCACT |
| 11151_33 | 34 | [G/T] | TTTTCATAACAGGCAAATCGCTGCAAGATTTCCGCT |
| 11155_34 | 35 | [A/C] | AGAATGAGTGCTGCATGAACCTGCAGATGTGCCGAC |
| 11157_28 | 29 | [G/A] | TACCAGTTTTTGGCACAGCTGTGCAGGTGAATGTTG |
| 11165_4 | 5 | [G/A] | GTTCGGCTGTTTGCAGAATGCTGCAGTAATGATCTA |
| 11166_2 | 3 | [G/A] | CAGACCTCTGTGGCACAGTGATGCCTCACCAGAGGC |
| 11167_5 | 6 | [G/A] | AATGCGCGCACCGCAAACCACTGCAGTCCCAGTGGC |
| 11168_28 | 29 | [G/A] | ACACTACCTGCAGCAGCACTGTGCCACCGCTACTTG |
| 11175_3 | 4 | [A/T] | GGCAGGGATGACGCATAGTGTTGCTGTACAGATCCG |
| 11176_15 | 16 | [A/G] | TGGTGGAGAGCCGCAAAGCGATGCTGAGGAAGAGCA |
| 11182_7 | 8 | [G/A] | TGGGTCTGAGCAGCATTTGTATGCTTGCTGTAGACT |
| 11189_34 | 35 | [T/A] | TCTAAACCGAAGGCATCCGTGTGCTATGATCACGTA |
| 1120_20 | 21 | [A/T] | CGAGGCATTGTAGCACGGCTATGCAATTTACATCAG |
| 11203_4 | 5 | [G/A] | GGCCGGAGCATGGCAAGAGCATGCCAGGCTACAGTT |
| 11204_2 | 3 | [G/A] | ACGGACAGACAGGCAGAAACATGCCTCAGCTCAGCT |
| 11214_19 | 20 | [C/T] | GCAAGTCAGCCAGCAGGCTCCTGCTTTAAACGGCCC |
| 1122_33 | 34 | [A/T] | ACACACGAGCGCGCACAGAATTGCCTGCCAACTAAG |
| 11220_7 | 8 | [G/A] | GCACCCTGAAAAGCACTGTGCTGCTGGAGATACTGT |
| 11228_34 | 35 | [T/A] | CACTGCATGTAGGCATGCAAGTGCAATACAGACTTA |
| 11230_17 | 18 | [A/G] | AAAGCTGAACCTGCATTAGCATGCGTCTCCTGCGAC |
| 11231_31 | 32 | [A/G] | TATGCTGGCCTGGCAGCATTATGCTCTGGGGATGCT |
| 11233_19 | 20 | [A/T] | TACGGTAGGAGGGCAGTGTAATGCCCTCAGAGGAAA |
| 11234_11 | 12 | [G/T] | GCCTTAAGGTGGGCAAGACCCTGCTGTAAGTCTTAG |
| 11238_11 | 12 | [C/T] | ATGATAATGCACGCATGCAAATGCAGCTCAAATGCT |
| 11240_31 | 32 | [T/C] | TTTAGTATTAGCGCAGGTAGCTGCGATTAGATGCGG |
| 11242_19 | 20 | [G/T] | CTAACCCTAACCGCAGAGTGTTGCTTGGTGATGGAC |
| 11243_27 | 28 | [C/T] | AGCTGCAGGCCAGCAAATTTCTGCTTTCTGCAGAGT |
| 11248_8 | 9 | [C/A] | AAAGGAAACGTAGCATGGCTGTGCAACTGTGCAATT |
| 1125_11 | 12 | [A/G] | AGAGTCTGGAGAGCAGCTCCATGCCCATGGGACTCA |
| 11252_3 | 4 | [T/A] | GAATTGCACATGGCATGGGACTGCAGAGATGTGACG |
| 11255_16 | 17 | [T/G] | GTCACGAGATCAGCAAGGTCATGCGAAGGCTTCGTT |
| 11260_19 | 20 | [A/G] | ATCATGCATCCAGCATCACACTGCTACTGACAACGC |
| 11261_18 | 19 | [C/T] | AGCACCCCACTAGCACAGCTCTGCTCTTTTCATGGA |
| 11264_1 | 2 | [G/A] | TGAAGCTCTGCTGCAATCTGCTGCTGATAAAACATT |
| 11266_11 | 12 | [C/T] | ATGCATGTTGACGCATGCAGGTGCATTTAAGTTGCT |
| 11275_32 | 33 | [T/G] | CTACATTATGGTGCATTTTGCTGCTGTGAAGGTTTT |
| 11278_10 | 11 | [C/T] | ATCGATTCCCCAGCACACGTCTGCTCAGGCCAAGTC |
| 1128_1 | 2 | [C/T] | TCAAGATGAAGGGCAGTGGTTTGCTGTCAGCAGGGT |
| 11281_19 | 20 | [C/T] | CGGTGCTATTTTGCAAACACGTGCTCCGTGCAGGCG |
| 11282_2 | 3 | [T/C] | TGTGGAGTGTGTGCAGCGTGTTGCATAAACCAGATT |
| 11284_6 | 7 | [G/A] | ATGATGGAGAGTGCATTTTGCTGCTCGGGTGTCGCT |
| 11299_33 | 34 | [T/A] | AGTCATCCCTGAGCACGAGTTTGCCGCCGGTCCTGT |
| 11303_26 | 27 | [G/A] | ACGGCCTGCAGAGCACGCAGCTGCTCGAGCTTTTAA |
| 11307_19 | 20 | [C/G] | AAACAGGAAAAAGCACTTTCCTGCTTCATCCGGACT |
| 1131_29 | 30 | [C/T] | CCAGAAGGCTAAGCAGGTGACTGCTGCTGCGGCTGA |
| 11318_11 | 12 | [T/G] | GAACAGCTGGAGGCAGCTGGATGCAACGTTTCTCTG |
| 11319_17 | 18 | [C/T] | ATGGGTGTGTAAGCAAGCGTGTGCATGTGTCTGTAA |
| 1132_11 | 12 | [A/G] | AAATGTTGTTCAGCAGCCCGTTGCTGGCCCTGTCAT |
| 11321_4 | 5 | [C/T] | CTCACGGTTTAGGCAGGTGTGTGCCTTTCCAAATCA |
| 1133_17 | 18 | [T/A] | CGGCTAACGCTGGCAAATCGCTGCTCTTTTTTTGAG |
| 11333_15 | 16 | [G/A] | CACAAAATTTGTGCAGAAACCTGCAGAGCCTGTTCT |
| 11340_34 | 35 | [G/A] | GGAAACGCAGCAGCATTAACATGCGACCCAGCGAGT |
| 11344_24 | 25 | [G/A] | AAAAAGGCACCAGCAAACACCTGCGTAGCAAACCAG |
| 11346_2 | 3 | [G/A] | GTGATGCTCTAGGCAATGTTCTGCTCAGAAACCTTG |
| 11347_1 | 2 | [C/T] | TCAGCAATTATAGCACTCCATTGCTCTGTCACACTG |
| 11351_30 | 31 | [A/T] | GGCAGACTGACAGCACAAAACTGCTGTGATAGACTC |
| 11353_17 | 18 | [C/T] | GCATAGTACCCAGCATGCGCCTGCTCTTGGTCACAT |
| 11354_19 | 20 | [G/A] | CTGTAGCACGTGGCAAAGTGTTGCTAAAGGTCACCA |
| 11362_26 | 27 | [G/A] | GCAAAATGTACCGCATGTGAATGCCTGACCATCACA |
| 11363_2 | 3 | [C/T] | ATCATGTCCTGTGCATGATGATGCCCGTTGGGCTCC |
| 11365_25 | 26 | [C/T] | AGTTCCTTATGCGCAGTGGAATGCTCACTCCAGACC |
| 11367_25 | 26 | [G/A] | ATTTTCACAGTGGCAGGGTGGTGCTGCAGGTAGTGC |
| 11370_1 | 2 | [C/T] | TCGTTTATTAAAGCACTGAGCTGCTGGAATGTCTCG |
| 11383_25 | 26 | [G/A] | CCTGGAGGAGACGCAACACTCTGCAGCTGCACCGTT |
| 11388_5 | 6 | [T/G] | GAGTTTTGGCAGGCAGCCAGATGCACATAAGCTGAT |
| 11401_30 | 31 | [A/T] | TAAAGTTCACAAGCAAAGCTGTGCTTCCACACAGAC |
| 11407_1 | 2 | [T/G] | GTCGCATCGTGAGCATTGTAATGCAGTGGCGTCTCC |
| 1141_4 | 5 | [G/T] | GAACGTCACGAGGCACCGCTGTGCTGCTGTACGTGT |
| 11412_1 | 2 | [A/C] | CAAAAGAAAGACGCACCTCCATGCCAAGGCGTATAT |
| 11413_11 | 12 | [C/G] | GATCAGCGCCGCGCATGGCACTGCAGTGAGTCTCTA |
| 11415_3 | 4 | [C/T] | CCCCGCGGCAGTGCATCAACATGCCCTAACGAGTTA |
| 11419_10 | 11 | [G/A] | CCGTTTCTACGGGCAATGTTTTGCATTCTTTCGTTA |
| 11422_7 | 8 | [A/G] | TGGGCAAGACCAGCAGGATGTTGCAGAGCACCAGCG |
| 11423_26 | 27 | [C/G] | TGACCTTCCGATGCACCAATTTGCTTCATGCATCTT |
| 11429_29 | 30 | [T/C] | ATGTAAGGATTGGCAGGCCAATGCCTTGCTGGAAAA |
| 1143_1 | 2 | [T/A] | GTGCCACTCAGAGCAAGGTTTTGCATGTCTTCTTTG |
| 11434_8 | 9 | [G/C] | TTAGAAACGAGGGCAGAAATCTGCCTGTTGTACAGT |
| 11439_4 | 5 | [A/G] | AAGTAATAGGTGGCATGGTCATGCTGCAGGTACTGT |
| 1144_18 | 19 | [G/A] | CAGACTAAGTGCGCATTCAAATGCGCCAGGATTCAA |
| 11440_4 | 5 | [G/A] | ACTTGTGACCTGGCAACACTTTGCTGGCTTTACTTG |
| 11442_25 | 26 | [G/A] | TGATGGGTTACTGCATCTGGCTGCAGCTTCACAAGC |
| 11444_27 | 28 | [T/G] | AAACACACATAGGCACTGCCATGCAGGTTTTTTTTC |
| 11447_5 | 6 | [T/A] | TGTGTTAAAGCAGCATGTCTGTGCAGGTCTCAGATG |
| 11450_7 | 8 | [C/T] | TTTGCTCCGAGTGCAGGTAGTTGCTCAGGTAGAAGA |
| 11451_32 | 33 | [T/G] | CAACAATACTCAGCAATGAGTTGCTATGAGCTTAGC |
| 11454_15 | 16 | [G/A] | ATACGCATGTACGCAGAAATATGCATATGCACACAC |
| 11458_24 | 25 | [G/A] | GTCAGCTTACTGGCAGGAGACTGCGGAGGGGGAACG |
| 1146_31 | 32 | [C/T] | ATCTAGAGAGCAGCATGACTTTGCGTCTCTACGGTC |
| 11463_33 | 34 | [C/T] | TGGAGTGAAGATGCAGTTAAGTGCTTGTGCTCGCTG |
| 11466_4 | 5 | [C/T] | TGTACGCAATGAGCAAAGTGCTGCAGCAGCACATTT |
| 11468_5 | 6 | [G/A] | TGCATGAACCAAGCATTGTGCTGCTTCTGTCGGCTG |
| 11472_17 | 18 | [G/A] | TGTTACTGTGGAGCACCGAGTTGCCGGGGGTTCCTG |
| 11473_27 | 28 | [G/A] | GTGACTAAAGCTGCATGTGATTGCTTCGGGGGGAAA |
| 11477_19 | 20 | [T/C] | CCACAGTATTATGCATCGATCTGCCGTTCCTAATAA |
| 11479_16 | 17 | [T/C] | CAACCCTGCACTGCAGTGAGCTGCTGGTGCCATCCC |
| 11487_32 | 33 | [G/A] | CTATAGCTAATCGCACACATCTGCCGAGTGCCGAGT |
| 11489_6 | 7 | [C/A] | AAGATCCATATCGCAACTCCTTGCAACTGGATCAAT |
| 11490_26 | 27 | [T/A] | TCGTTTCCGTCAGCACCCCATTGCAATAATGTGTGA |
| 11492_32 | 33 | [C/T] | ACACAAGGGGAAGCAGTCGAATGCCAGCTTCTCACT |
| 11494_6 | 7 | [T/C] | CCTTTCTTGACTGCAGTGAGTTGCCTGACCTGGGGC |
| 11495_11 | 12 | [C/T] | TTGCTTCACTCCGCACGTCTATGCTCTCCACTCCGT |
| 11496_32 | 33 | [C/T] | ATACAAAGAGTCGCAAACAGATGCAAAATGTGCGTG |
| 11499_2 | 3 | [A/C] | ACATGGTGCAGTGCATGTAGCTGCCAGGTGCAGGTG |
| 115_1 | 2 | [A/G] | GAGACACTGTAGGCACACAAATGCACTCAAACATTC |
| 11502_17 | 18 | [G/A] | CAGTCCACAACCGCAGTGAAATGCAGAGACACCATA |
| 11504_16 | 17 | [G/A] | AGACATGTTCCAGCATGAGTGTGCTGCTAACAGAGG |
| 11505_19 | 20 | [C/T] | GTCCTCTAGGTGGCACCGTCGTGCTACCAACCAGCC |
| 11506_16 | 17 | [T/C] | GCTGTGGCTTTGGCACTGAAATGCACAGATGTTTCC |
| 11509_7 | 8 | [C/G] | AAGCACTCTCCTGCAAAATGCTGCAGGATCTATAAG |
| 11510_11 | 12 | [A/T] | CGCTGACCTCCAGCAGTAATCTGCAACAATGTCTGG |
| 11513_31 | 32 | [C/T] | CACACCATCGTGGCAACCGCCTGCTCTGTCACGGCT |
| 11515_32 | 33 | [C/A] | GCTGAGATGGTGGCACGCTGCTGCTGCTGCTGCTGC |
| 11516_24 | 25 | [C/T] | GGAGAGGGTCTCGCAGCTGTGTGCCGAACTCGAACG |
| 11520_17 | 18 | [A/T] | GTTAATCGTGTTGCAGAAAGATGCTATCGTACCTGC |
| 11522_11 | 12 | [A/G] | CCAACATCGGTAGCAAAAAAATGCTTCACGAAAGAG |
| 11523_24 | 25 | [G/C] | TCACCAGCTCCTGCAAAACAGTGCGATTTGTGTCTT |
| 11524_11 | 12 | [A/G] | GTCAGTCTTCCAGCACGTTCATGCTGCCGACCGGCT |
| 11528_29 | 30 | [G/C] | GAAACTGCCCTAGCAACAAGATGCTGAGTGATGTAA |
| 11533_28 | 29 | [A/C] | GCAGAGCAACAGGCAGACCCATGCCACCAAATTCCT |
| 11546_1 | 2 | [T/A] | CTGCTTCCACTGGCATACAAATGCACACTGATGCAA |
| 11548_32 | 33 | [C/A] | AAAAACACATGCGCACTGGACTGCTACTACATCCCT |
| 11552_28 | 29 | [C/T] | GAATATTTGCTAGCAGCCAATTGCGTGACGCCGGTG |
| 11554_15 | 16 | [C/G] | TTACTAGAAGGTGCACTCAGGTGCACTTTTCTCTCA |
| 11559_1 | 2 | [C/A] | GCAGAGCTCCCTGCAGGAAAATGCAGAAATGAGAAA |
| 11562_19 | 20 | [C/T] | GACAAACGAGAAGCAACAGCCTGCATCCCTTTTCTG |
| 11565_31 | 32 | [C/T] | GAGCAGGGATGAGCACACACCTGCGTCTCCTCAAAT |
| 11569_5 | 6 | [C/A] | ATATGCAATGTAGCATACAACTGCAGGGCATCAGAC |
| 11571_26 | 27 | [A/T] | TTGGTCTAGAATGCACCAGACTGCAAATGCTAGACG |
| 11572_8 | 9 | [G/T] | ATCCTACAGGGTGCAGGTATCTGCTGCGTATCTCTC |
| 11576_4 | 5 | [A/G] | CCCAATGGCAAAGCAAGTTCATGCAATCTTTGCTTT |
| 11580_24 | 25 | [G/A] | GCAACACTCATCGCATGGCTTTGCGATGGTCGGCTG |
| 11581_1 | 2 | [T/A] | CTGCTTCAGGCTGCAGTATCATGCACTCTGACCCCA |
| 11582_11 | 12 | [G/A] | TGGCATGTCGTAGCACAGCGCTGCAGTGGGTTGCAC |
| 11584_26 | 27 | [T/C] | TTTTTCTTCGACGCATCAAGCTGCCCTCAGCTAGAG |
| 11593_6 | 7 | [C/T] | TTACTTCTCAGTGCAAGGCCTTGCTTGAGTTTGCAC |
| 11597_31 | 32 | [G/A] | TTGCAGAACACAGCAGTGAGCTGCAGAAGGAGGACT |
| 116_2 | 3 | [T/C] | CTTCAGGACGCTGCACATAGCTGCTGTTCTCCAGAG |
| 1160_20 | 21 | [C/T] | TGTCAGATATAGGCAGGTAGCTGCAGCTGGATTTAC |
| 11601_11 | 12 | [T/C] | CTCTTGAGTAATGCATCTGTCTGCTCGCTGGGAACT |
| 11604_32 | 33 | [T/A] | CACTTCTGGAGTGCATTACGATGCGTCTGTTCTGCT |
| 11605_28 | 29 | [G/A] | CCTAGCAGACAGGCATCACTGTGCCAACGCGCACTC |
| 11609_11 | 12 | [A/G] | GGTCACATTTAAGCACCCCAATGCCCCCATGGTCAC |
| 11612_8 | 9 | [C/A] | GGCATGTCCAGTGCATTTTACTGCAGTTTTGCATAT |
| 11614_33 | 34 | [C/A] | TGGAAGGTTAGTGCAAATTCATGCAAGTCGGGTCAG |
| 11617_27 | 28 | [T/C] | AGTCGAGCCACTGCAAATCGCTGCAAGTCGCTTAAA |
| 11618_32 | 33 | [A/C] | CACAAACCAGTTGCACAGACATGCAGGTGGAAAACT |
| 11622_18 | 19 | [C/T] | TCTCCCCCTGTAGCAGCGCGTTGCATAAAGGCCCGA |
| 11623_11 | 12 | [T/A] | TAGTAGCATGATGCAGTACTGTGCACAAGTCTTAGG |
| 11633_29 | 30 | [G/C] | AATAGTGGAAAAGCAGGGCATTGCTGCATGGTAAGA |
| 11634_2 | 3 | [C/T] | AACCCGCGTCCGGCACCGACGTGCGTTCTCCAGACG |
| 11636_11 | 12 | [C/T] | CACGCGAGGCTCGCAAACGACTGCGCGCTCCGGAAG |
| 11643_31 | 32 | [A/C] | CAGTGTAGCAGGGCACAGGGCTGCTCAGTGCAGCCT |
| 11647_28 | 29 | [T/G] | CAGTCTTACACTGCAGTACTGTGCGGTTGGGGCTTT |
| 11648_33 | 34 | [G/A] | CAAACCAAATATGCATTTCTCTGCTTTGGCCCTGAG |
| 11654_33 | 34 | [C/T] | GACACGGCAGCTGCAGGTCTCTGCATGCTCATACGG |
| 11655_2 | 3 | [A/G] | TTAAGAATCCCTGCAAACAGATGCTGTCCGGAGTGT |
| 11659_6 | 7 | [G/A] | TGCCTGGGATTTGCAAAACTTTGCTCGTTCTTCTCC |
| 11661_11 | 12 | [G/T] | CTCCGTCGACAGGCAATGCTCTGCGGCAACCCGTCA |
| 11664_1 | 2 | [A/T] | TACTCTTTAGTAGCAGAGTGCTGCTCTGTCAGACAG |
| 11672_28 | 29 | [G/T] | TGTGACATGTTAGCATGCTGTTGCTGCTGGACAGAC |
| 11673_16 | 17 | [C/A] | CTGATTACAGCTGCATCTGTCTGCGTTACGTCACTG |
| 11682_2 | 3 | [C/T] | TTCAGTAAAGCTGCAGTTACTTGCTGCTGAAGTGAT |
| 11683_30 | 31 | [C/T] | CTACTTTGAGCTGCAGAAGAATGCAGACCCCGAGCG |
| 11684_26 | 27 | [A/G] | TTTTAAGCCTCGGCAGATTGATGCACAGGCTGTCTG |
| 11702_2 | 3 | [G/A] | GGGAAAACCTTGGCAGAGGGCTGCATATCTGTAGAC |
| 11709_4 | 5 | [G/A] | GTGTGTACGAACGCATTCAGATGCGAGGATGTGCTT |
| 11713_25 | 26 | [T/C] | CTGGATGTATGTGCAGAGTTCTGCATGAATACGAGA |
| 11714_34 | 35 | [G/A] | AACTGTCAGTCTGCACTGAGCTGCAGATTCATGAGA |
| 11715_8 | 9 | [C/T] | CTTCGGATCTGGGCAGCAGGATGCCGTTGTAGATAT |
| 11718_15 | 16 | [A/G] | GATCTACTGTTTGCAAATGGCTGCAACATTCGAACG |
| 1172_4 | 5 | [T/A] | AAACTAGATTGTGCATGGCAATGCCACTGTACACAA |
| 11720_20 | 21 | [C/G] | CTTTCTGAACCAGCAGCACACTGCAGAAGGGGTTTA |
| 11725_9 | 10 | [G/A] | AACAGCTGGGTGGCACCGCTGTGCTCCCATCTTAGT |
| 11727_31 | 32 | [A/C] | CTTGCACACACAGCATCCTGATGCATGTAAAAACCT |
| 11728_9 | 10 | [G/A] | TTCCTCAGCGTAGCAAATGAATGCAAAGGACATGCC |
| 11736_4 | 5 | [G/A] | CACTGCATGGTGGCATCTGTGTGCTGACATTCAAAG |
| 11738_9 | 10 | [C/T] | TAAGGCTCTCCTGCATCTTGGTGCCAGTTAGCATAG |
| 1174_2 | 3 | [G/A] | TTGATACTAAGCGCAAATATTTGCCAGGCCTGTGAG |
| 11749_25 | 26 | [T/G] | AGCATAATGACTGCAGATTACTGCCTCCAGATGGTG |
| 11757_26 | 27 | [G/A] | TGTTCTCGTCCAGCAGCTGCATGCCTGAGGACAGGA |
| 11758_17 | 18 | [C/T] | CTCAGTGTCACAGCAAACCAGTGCACTGTTCATCTT |
| 11759_27 | 28 | [G/A] | CTTACTATATTCGCACCGCGCTGCCGCGCATAGGGA |
| 1176_26 | 27 | [A/G] | TCATCTGACGCTGCAGCAATTTGCCCGAATAAACGA |
| 11761_19 | 20 | [C/T] | CCATGCACAACTGCATGTGCGTGCAACAGGAAGAAG |
| 11764_27 | 28 | [C/T] | AATGCAATCTGCGCATGGATTTGCGCGTACGAACGC |
| 11766_7 | 8 | [C/T] | GCTCTTTCCTGTGCAATGTGTTGCTAATCTGTCCAT |
| 1177_27 | 28 | [A/G] | ACGTCTGTTCCGGCATGGTTGTGCTCCAGCGCACAA |
| 11770_33 | 34 | [C/T] | ACTATGAGACATGCAATGACATGCCTACACCCACAC |
| 11773_6 | 7 | [G/A] | GATGTCGCAGGGGCACAGTCCTGCTTGCTTTTGTGT |
| 11777_9 | 10 | [C/T] | CTGCCTTCACCTGCATGCTCATGCCACCATAGTAGG |
| 11783_31 | 32 | [G/T] | CACATGGACAGTGCAGATTACTGCCAGACAAGCAGC |
| 1179_31 | 32 | [A/G] | CAAAGCGTCTCTGCAGGAAGCTGCTCCAAATAGAAC |
| 11796_3 | 4 | [T/C] | TGACGCTCTCAGGCAGCTCAATGCCTCAAAATGCAT |
| 11797_19 | 20 | [G/A] | AAAGGGTACACAGCAAGTGGGTGCATGCCGTTATAT |
| 11800_18 | 19 | [C/T] | CACCAACCCTTAGCATGATGCTGCCACCAGCCTGCT |
| 11805_20 | 21 | [C/A] | CACATCCTTATTGCATCTGGCTGCTTGGTCAACACC |
| 11807_10 | 11 | [G/A] | CACTACCACTGAGCATCAGAGTGCTACATGCACACC |
| 11808_25 | 26 | [C/T] | AACCAGGTCCGGGCAGTGTCGTGCTCTTCAGCGAAC |
| 11809_32 | 33 | [C/T] | GCCGAAGTGACTGCAGTGACTTGCCTGCAGTACGTT |
| 1181_3 | 4 | [A/T] | AATAAAAAAACAGCAGCAGAGTGCGAACAGCAGAGC |
| 11815_34 | 35 | [C/T] | CGCTGCTATTGTGCAGGAGACTGCTGATGTCTGGCT |
| 11816_25 | 26 | [G/A] | TGGGGACATACCGCAGTGAAATGCAGATATCACCAA |
| 11820_1 | 2 | [G/T] | GGTGGAAAAAGTGCAGAAGACTGCATGCCTTGCCCT |
| 11825_32 | 33 | [C/T] | ACACACAAGCATGCATGGGAATGCATGAACACCTCA |
| 11827_24 | 25 | [T/C] | GAAGAACGTGAAGCACAGTGATGCTACAGCGGTACT |
| 11828_2 | 3 | [G/A] | CCGTCTCCAGAGGCATGTCTCTGCTTTGACCTTCGT |
| 1183_33 | 34 | [G/A] | CAGCAGCCCAAAGCACAGCAGTGCAAAGAAAACGCT |
| 11830_11 | 12 | [T/A] | GAGGAAGCTTGTGCAAAGATCTGCAGAAGGTCAGAG |
| 11833_27 | 28 | [C/T] | TTTGCATATGTTGCAGAACATTGCCCCCGTCTGCAG |
| 11836_3 | 4 | [C/A] | ACCCATGCATCAGCAAAGTCCTGCTCTCTCGCTCTC |
| 11837_27 | 28 | [C/T] | TCACTACCTGCAGCACCATGGTGCCACCCGATATAG |
| 11840_17 | 18 | [A/T] | TGCTGCCAGGCTGCAGAAACATGCAGACCACACTGG |
| 11844_5 | 6 | [C/A] | TCTGGCTGTGAGGCAACACCGTGCCACCTGACGAAA |
| 11845_5 | 6 | [C/A] | GATGTCTAAAGTGCAGCACAGTGCAGGAAAACAAAG |
| 11853_8 | 9 | [A/G] | TCCCCACCATGTGCAGCAGAGTGCCCATGAACTTGT |
| 11854_34 | 35 | [G/A] | GATTTGAGTCTGGCATTGTGGTGCTGTGGGACTTGA |
| 11855_18 | 19 | [T/C] | ATGTCTTTTTAAGCACGTTGGTGCGAACATCCATGA |
| 11856_24 | 25 | [C/T] | CAGCAGGAAGTAGCAGGAGTATGCCTGCCAGATGAA |
| 11864_34 | 35 | [G/A] | TATCCAGAGACAGCAGGCTCATGCCCTCCAGGCTGA |
| 11865_25 | 26 | [C/A] | GAATCGTCTTGTGCAAACTGCTGCCCGAGTGACTGT |
| 11867_10 | 11 | [T/G] | GCTCAAAGCCTGGCATGCAGGTGCAGGATCCCACAG |
| 11877_24 | 25 | [G/A] | TCCATTAAGGACGCAGCGGAGTGCGGTGTGGCCCTT |
| 11882_8 | 9 | [G/A] | ATTTCTACGAAGGCACGATGATGCCACGATTATACA |
| 11883_10 | 11 | [T/C] | GGTCCAACAGTGGCAGTTTGGTGCTGAGCAAATCAT |
| 11886_3 | 4 | [G/A] | CCGGGCATGACCGCAAATTCGTGCGAGTGGATTTGG |
| 11887_27 | 28 | [G/A] | ATTAAAAAGTCAGCAGCACCTTGCCCAAAGGACACA |
| 11889_17 | 18 | [G/C] | GTCTCTCTCTCAGCACCGCTATGCCTTAATTGGAGG |
| 11890_3 | 4 | [T/A] | ATGTAACTGCTAGCACATGGGTGCACATGGCTTATT |
| 11891_15 | 16 | [C/A] | TTGAATGGACAAGCACTGTAATGCCTACATCGTCTG |
| 11897_10 | 11 | [G/C] | TTATAGTTTGGTGCAATTTCGTGCCCACGGTCCGAC |
| 11902_15 | 16 | [C/T] | AAGGATCAGCCTGCATGGCCCTGCCACCCTCACCGA |
| 11905_9 | 10 | [C/T] | AAAAACAAGCGTGCATGCACTTGCACAAAACACACA |
| 11906_27 | 28 | [T/C] | GCGTCTCATCATGCAAAAATTTGCATGCCGTACATT |
| 11909_16 | 17 | [T/G] | CTTTTCAGAGCAGCATTGTGGTGCATTGTTGTTGCC |
| 1191_32 | 33 | [G/A] | AAATGATTGAGTGCATAATCGTGCGCCTCTGCGAAG |
| 11910_20 | 21 | [G/A] | TGGCAGCCTGCAGCAAACTGGTGCCCTACCCTGGGG |
| 11913_5 | 6 | [A/C] | GAACAAACACTGGCAGCATCATGCTCTGGGGTGCTT |
| 11914_30 | 31 | [G/A] | GTGAGCTACATGGCATTCTTGTGCGGGCGGAAGCCT |
| 11916_18 | 19 | [G/A] | ACATGGTTGTTGGCAGGCGCTTGCTGTTTTCCTTTG |
| 11920_19 | 20 | [G/T] | ATGACACTGCTGGCACAATGCTGCTGGGTTCTTCCC |
| 11922_18 | 19 | [C/A] | TACGAAGAGAAAGCACCACCTTGCTGGTATCGTAAA |
| 11925_5 | 6 | [T/C] | GTAAATGTGCACGCATCCTGCTGCCATCCAGGGTAT |
| 11937_1 | 2 | [A/C] | CAAAGGTTTCCAGCATTGCACTGCCACTGTGCAAAA |
| 1194_32 | 33 | [C/T] | AAATGAGTCATGGCACTAACATGCGTCTGGAACTTT |
| 11945_29 | 30 | [C/T] | TGCCACCTCCCTGCAGAACATTGCCAGATCTGAACA |
| 11952_25 | 26 | [G/T] | TTGTGCAGTTGTGCAGCTCGCTGCGGTTTGGCCTAG |
| 11965_8 | 9 | [C/T] | ATCCTTCTCGAGGCAGGATCTTGCTTAAATGCTCAG |
| 11966_5 | 6 | [G/A] | CTTTGGTGGATTGCAGTCTGGTGCACTAGATGTTGT |
| 11971_31 | 32 | [A/G] | TGTACTGTTTACGCAAGCTCATGCCCTCGTCAGAGT |
| 11973_34 | 35 | [A/T] | ACAGAACGGTCCGCATGGTTCTGCCAGCAGGACAAA |
| 11976_16 | 17 | [C/T] | CAGCTCCCTCTGGCATCGCACTGCCTATGGGGACAT |
| 1198_30 | 31 | [C/T] | CAGGAGAAATGCGCAGCACAATGCCTTTTTCATCAA |
| 11980_28 | 29 | [G/A] | CTCTCAGTGGACGCAGGTTAGTGCAAGAGCAGTTTG |
| 11998_30 | 31 | [C/T] | GAGCTTTGTGCAGCAGGCACGTGCAGAACCCATCAG |
| 12000_24 | 25 | [C/T] | GGCGAAGGCGGAGCAGCTCGGTGCCGAGGGAAACGT |
| 12001_10 | 11 | [G/A] | TTGCGCAACAGAGCATTGTCATGCTGGAACTGGTTT |
| 12003_10 | 11 | [T/A] | GCAGGTAGTTTAGCAGCTCGGTGCACTGCGGCATGC |
| 12006_31 | 32 | [G/A] | GACGCTTCAGTGGCACGAAGCTGCTCGTCACGCAGG |
| 12007_8 | 9 | [C/T] | GTACAGGCCCGAGCAGCTGGATGCGAGCAAAAAGAA |
| 12009_27 | 28 | [C/T] | GCACGAGCCACAGCAGATCACTGCGGGCAACCCACT |
| 12013_28 | 29 | [A/T] | ACCAGTTACATTGCATCAGTCTGCATTCACACTGGG |
| 12014_24 | 25 | [G/A] | GTATTTACAGTTGCAGCGCATTGCGTTGTTGGTGAA |
| 12015_4 | 5 | [G/A] | AGCTGCCGAAATGCAGATTACTGCTCCTCGTGTTCA |
| 12021_17 | 18 | [G/A] | CTCATCTTGACTGCATTGAGCTGCTCTTTGCGATCT |
| 12030_28 | 29 | [T/C] | CAGATCCCTAAAGCAACACTGTGCTGCTTCGCTAGT |
| 12032_34 | 35 | [C/A] | GCAGCCTGCTTTGCAGCGTTCTGCAGGAAATGTTCA |
| 12039_7 | 8 | [A/G] | GAAATCTAGGCTGCAGCTTTGTGCTAATGAGGAGAG |
| 12040_11 | 12 | [C/T] | GTAACCCCTGACGCAGGGACTTGCGGTACGTCTCTA |
| 12042_28 | 29 | [G/A] | CATGCCATCACTGCAGGCAAATGCACACGTTTACAA |
| 12043_30 | 31 | [T/C] | AAAAGGAAAAGAGCAAACGACTGCTCGAATCCGCCA |
| 12044_11 | 12 | [C/T] | TCATGACATTTCGCAAAAACCTGCCCTCGACCCCCA |
| 12049_31 | 32 | [C/T] | TGGCTGCAGCCCGCAGTCGAGTGCCAGGCTTCCTCG |
| 12055_15 | 16 | [C/T] | CTACACTCCCCTGCACGCCGCTGCCGCCAGCGGACA |
| 12058_31 | 32 | [C/T] | TGTGATGTAAACGCACCATGATGCCAGTCAGCAGAC |
| 1206_28 | 29 | [G/A] | GAAGGAGGAAGAGCATTAGTATGCAGGTGAACTCGT |
| 12066_17 | 18 | [G/A] | GAGTAGAAGGTGGCACTGTTGTGCAGTGGTAGTGTT |
| 12069_3 | 4 | [A/G] | CGTACGGCGGACGCAAACGCATGCCTCTGGTTAATC |
| 12070_15 | 16 | [T/C] | CCACTAAAGTGAGCATGCTCGTGCTCGGTCAATCCG |
| 12072_1 | 2 | [C/T] | TCGCGTTCTCTTGCAGAAACTTGCGGAATTTGTTGA |
| 12074_6 | 7 | [C/T] | CAGATTTTTACAGCACAGACATGCCAACTGGCTGAA |
| 12075_8 | 9 | [G/T] | GCAAATCAGTCAGCAGAACCGTGCCACAGTTTCACT |
| 12078_11 | 12 | [G/A] | GTGACCTGGCTGGCACAGGGCTGCTCTGGTGACGCA |
| 12083_6 | 7 | [C/T] | AACCTCCTACATGCAGAATGCTGCTCTGTGGGTACT |
| 12086_6 | 7 | [G/C] | ACAAAAGTTTGTGCATTTTAATGCAGGATGCCGGGT |
| 12089_26 | 27 | [C/T] | CAAGCACTACCAGCATCCCCATGCCTCCTGGATGAA |
| 1209_29 | 30 | [A/G] | AGACCCCAGGAAGCATCAAAGTGCCTTTCATTTATC |
| 12090_2 | 3 | [T/A] | TCTGCTCAGCTGGCAGTGAAGTGCTCAGAAACAGTG |
| 12098_34 | 35 | [T/C] | GGTGCAGTTTTGGCAGAGTGGTGCTAGAATGTGGTT |
| 1210_25 | 26 | [C/T] | ATGAACCTCAATGCAAAACACTGCTCATGTGGGTGC |
| 12100_16 | 17 | [G/A] | CTTTCCCTGGACGCAGGAAATTGCTCAAATATCTCC |
| 12102_1 | 2 | [T/A] | TTCTACCCTTGTGCAAGAGATTGCCCACATGTATTG |
| 12108_32 | 33 | [C/T] | CTTGCCAACGTAGCATGTAAGTGCCACGGCGTCTCC |
| 12120_10 | 11 | [C/T] | GCAGCACCAGCGGCAATACGCTGCTCAACATCACAG |
| 12124_20 | 21 | [G/A] | GTCTCACAGGCAGCATGTGTGTGCTGCTGAATGTCT |
| 12128_6 | 7 | [G/C] | ACACAGGACTGGGCAGGAAACTGCATAATCCAGAAA |
| 12129_31 | 32 | [A/G] | GGTGTGGGTGATGCAGGTGCCTGCTTACTCTAGTGG |
| 1213_31 | 32 | [A/G] | TACTTACTGTGAGCAGATGCCTGCCCTGTTTAAAGT |
| 12139_15 | 16 | [C/A] | CTCAGGCAATTAGCACACCACTGCTGCTTGTTGAAC |
| 12140_26 | 27 | [G/T] | CCAATGCTTCAGGCACGTGTGTGCACGGTTCACTAG |
| 12146_4 | 5 | [G/A] | TGATGGAGGTGTGCATCATAGTGCTGCTTGCTACAC |
| 12149_16 | 17 | [C/A] | GTCTTCAGGTCAGCAACATGATGCTCTGAGTCTGTA |
| 12151_17 | 18 | [G/A] | CTTTAAAACTAAGCACAAAGCTGCACAATGAGGCTT |
| 12156_17 | 18 | [G/A] | TAGTGTCGAACAGCAATGAGCTGCTAACATGTAATC |
| 1217_34 | 35 | [G/T] | ATAAAGGTTCCAGCATCAATCTGCTCTTTACTCGGT |
| 12171_25 | 26 | [C/T] | TCGGCTGAAAGAGCACATCGGTGCTCAGGGTGTGCT |
| 12174_1 | 2 | [G/A] | CATAACTTACTTGCAGCATGTTGCCGCGGCTCTGAT |
| 12178_31 | 32 | [C/T] | GTCATGACCCTGGCACCGGTTTGCTGGTTGTCCTTC |
| 12180_28 | 29 | [G/A] | GGATCATGGTTTGCAGGTCGATGCTGGCGTCTGACA |
| 12181_1 | 2 | [T/A] | ATGGCTGAGAGAGCATCTTCTTGCAGGTGATTAAAC |
| 12183_27 | 28 | [C/T] | AACGTCCCTCCAGCACTATCCTGCTCTTCGGGTCAT |
| 12185_8 | 9 | [A/T] | TTTTTGGCAGTAGCAGTTACCTGCTGGTTGGGAAAA |
| 12194_34 | 35 | [T/A] | CTAAATTGCACAGCACTTATCTGCGCCCCTTGTATA |
| 12197_30 | 31 | [T/G] | CTGCCTGGTTGTGCACAGCCATGCGAATCCTAGTTC |
| 12200_20 | 21 | [A/G] | CCTCCTCTGAGAGCAAATCAATGCAGGTGGTCATTT |
| 12204_24 | 25 | [T/C] | GGAGAGGCGAGGGCAACGGTGTGCTGGGCATGACCG |
| 12210_26 | 27 | [T/A] | CGTCCAAAATAGGCAAGCGAATGCTATTCGTTGCCA |
| 12216_11 | 12 | [C/G] | TATTTCAAACACGCACATGTGTGCACACAAGCTTTT |
| 12220_31 | 32 | [C/T] | CGCAGTGTCACTGCACAGGTGTGCACAATCTCAAGA |
| 12221_19 | 20 | [C/T] | CAAAGTTACGCCGCAGCTTCTTGCGTGGAAAGTGGA |
| 12225_20 | 21 | [C/T] | GCCTGCTGCAGTGCATGGCCCTGCCACACCTTTCCT |
| 12227_3 | 4 | [A/T] | ATTAAGTGCATTGCAGACCCTTGCACCCAGTATATA |
| 12228_15 | 16 | [A/G] | CGGGTACTAGAAGCAAAAATCTGCCAGACCTAGCAA |
| 1223_17 | 18 | [C/T] | TTCAGGTCACGAGCATCCGTCTGCACGAAGGACCGC |
| 12230_26 | 27 | [G/A] | GCTCAGCCAGATGCAGAGTGCTGCAGGGAAGCAAGA |
| 12232_2 | 3 | [C/T] | CTCTTGCTTTTTGCAGGGTGGTGCTGATGAAATGAG |
| 12234_26 | 27 | [T/C] | TAGTACTTGTGAGCAGTACAATGCTTTGGCCTTGTG |
| 12239_6 | 7 | [G/A] | GTATTCGTTGATGCAGTGCAGTGCTGCAACATGACT |
| 12245_18 | 19 | [G/A] | CATTATGGAGGAGCATGTGAGTGCATGTTTTAGTAA |
| 12246_1 | 2 | [T/A] | ATACAAAAGGGAGCACAATGGTGCAGTCTGAGTGAC |
| 12247_8 | 9 | [C/T] | CACCACTTCAAAGCAGCAGGTTGCACAGCCCTGAGG |
| 12255_30 | 31 | [C/G] | GATTTCAGTTTTGCATTACCGTGCGTACTTCTGGGT |
| 12272_25 | 26 | [G/A] | GTGGGCAGAGAGGCATGGACATGCAAAGCTCCTTTT |
| 1228_1 | 2 | [G/A] | GGAAGCACTGAGGCAGATTTGTGCCTCGTCTATTTT |
| 12281_2 | 3 | [A/G] | CAAGACTTTTGCGCAGTACTGTGCTTACATATGCTA |
| 12283_10 | 11 | [C/T] | GGTATAATTACGGCAGTGATTTGCTACGGTGGCCCG |
| 12284_11 | 12 | [T/C] | AGCTACCTCTTTGCAGGTAATTGCCTTGTCAGCAGA |
| 12288_9 | 10 | [C/G] | ATTTTAAGCCGGGCATCCACATGCATGAAGCTGACA |
| 12291_4 | 5 | [A/G] | TGGTAGAGCCGAGCAGCATTGTGCCAAACCAGGTGA |
| 12294_5 | 6 | [C/T] | CAGTTCACGGCTGCAGAAATTTGCTCCTTTGCCGCT |
| 12307_30 | 31 | [A/C] | GCACATGCATGTGCACATGGGTGCAGAACACAATCT |
| 12309_16 | 17 | [T/C] | TGGATAAGTGCTGCACTGTGCTGCTCATTATAACAC |
| 12317_16 | 17 | [C/T] | AATACACTGATAGCAACGACCTGCATGCGGTTATTG |
| 12319_31 | 32 | [G/A] | CGGCGCTACTGCGCACCTGCCTGCAGGGGGTGATGC |
| 1232_26 | 27 | [G/A] | AGAACGAGAACAGCATTGCATTGCAGGCAGCTGGGA |
| 12321_6 | 7 | [G/A] | CTCGGCGAAGATGCAGCCGGCTGCCCACATGTCAAT |
| 12323_24 | 25 | [G/A] | TTAGGTAGAAAGGCAGTGCGTTGCGACAACACTAGT |
| 12330_11 | 12 | [G/C] | AGCGTACGCTCGGCAAATGGCTGCTTTAAATCTTCT |
| 12331_25 | 26 | [C/T] | GGATGGCGCCGCGCAGGCGTGTGCCCGTGCGGTAGT |
| 12333_29 | 30 | [A/G] | CTGAAGCCCCTTGCACGCACATGCGAAACACGCATT |
| 12337_8 | 9 | [G/C] | TGGCTGTCGCCTGCAGACACCTGCACTACCTTCTCC |
| 12339_27 | 28 | [C/A] | TACTGACCCCCTGCACATGGATGCTACCTACAGATC |
| 1234_11 | 12 | [G/T] | CACAATAGGGCGGCACAGTGGTGCCACAGGTAGAAC |
| 12353_18 | 19 | [C/T] | CCACTACCTGCAGCACCACCGTGCCACCTGAGATGG |
| 12357_11 | 12 | [G/A] | TGGAACATTGCGGCAGCAATCTGCCAGTTTCATTAC |
| 12358_19 | 20 | [A/G] | GCTATAGCGGTCGCATGTCAATGCACCAGTGCTACT |
| 1236_18 | 19 | [C/T] | AAATATCTCGACGCACAGCGTTGCCAGAGTCGATAC |
| 12361_25 | 26 | [G/A] | CCTGAAGTTCAAGCAGTCCTTTGCTGAAGGATGCAT |
| 12364_16 | 17 | [G/A] | TTCATGTCACTGGCAGGCTTCTGCCTAATTCTCATT |
| 12367_9 | 10 | [C/G] | TAGCTATATCACGCACCGTAGTGCCTTGTTCTGTCT |
| 12368_31 | 32 | [A/G] | TTTGCTGAGCTAGCACGCTTTTGCGCCTGCAACCAC |
| 12374_15 | 16 | [C/T] | ATAGCCACAACCGCACACCACTGCTACACAATCCCG |
| 12377_30 | 31 | [G/A] | TACAGACCAAACGCAAGCTTCTGCAAGGCTGAAGGG |
| 12379_34 | 35 | [C/T] | CCACCAACGAAGGCAGCAGGATGCTCTCTCTCTCCG |
| 12386_27 | 28 | [A/G] | AACCTTCTGCTGGCACCAACATGCTAAAATGGCTGT |
| 12388_16 | 17 | [C/T] | GCCAGTCCCGATGCAACGTGCTGCCGACTTGCTAAT |
| 12398_9 | 10 | [G/A] | GAATACAAAGGCGCATTTGGCTGCTGCTGCCGCTGT |
| 1240_4 | 5 | [T/C] | GTTTTGCAGTTGGCACAGTGGTGCGCTTTACCTGTA |
| 12403_27 | 28 | [C/T] | AGTGCTGTGTGGGCAAGAGTGTGCCATCTTTTTATA |
| 12405_24 | 25 | [C/T] | ACACTACCTGCGGCACCACCATGCCGCTCGAACCAG |
| 12407_9 | 10 | [G/A] | TAGGCCCACGAGGCACAGTGGTGCTGAGCTAGTGTG |
| 12408_9 | 10 | [T/C] | GCAAAGTGGTGTGCAAGAAGATGCGACTGAAAAGTT |
| 12410_16 | 17 | [C/G] | CTTTTAGGCAGAGCAACAGGCTGCTCATAGTGGTTA |
| 12412_4 | 5 | [A/G] | CTTAAGTGCGCCGCACACTCCTGCCTTTCATACACA |
| 12413_24 | 25 | [T/C] | TTGGCCTTGAAGGCATCCGCTTGCTCACAGGCATCC |
| 12416_2 | 3 | [G/T] | TCGTGATCCATGGCAGCTCCATGCTGACATCTGGTG |
| 12417_25 | 26 | [C/T] | CCAGTTTCCTGAGCACTAGTCTGCTCCCACAAGGAA |
| 1242_32 | 33 | [A/G] | ATTCAACGTGATGCATTCTTCTGCCACACAGTAAAA |
| 12420_26 | 27 | [T/C] | ACCCCGTACGACGCAGCATTGTGCAGCGGGATCAGA |
| 12425_27 | 28 | [C/T] | GTAAAGGCACAGGCAACTGCTTGCTTTCTGGCAAAT |
| 12426_30 | 31 | [C/T] | CCAAAATCTTACGCAAACACATGCACTCCCCCTGCT |
| 12427_16 | 17 | [A/G] | AGTCCACAGTATGCACATCCTTGCTGTTCAGCTGGA |
| 12428_16 | 17 | [G/C] | AAGAGAAACTGGGCAGGAGACTGCAGATGGTGACCG |
| 12430_6 | 7 | [T/C] | CGGGGGTTGGCGGCAGGGAGATGCCGGAGAAAACAG |
| 12434_20 | 21 | [T/C] | AAAATGCTCCTGGCAAAATGTTGCCTGGTGAAGTAT |
| 12440_2 | 3 | [G/A] | TTGAGAACTGCGGCACTCTTTTGCCTTCCAGGCACG |
| 12441_28 | 29 | [T/C] | ACACACTGACCTGCAACACTCTGCATCCTAACTTAT |
| 12449_9 | 10 | [T/C] | AAATGCTCTCATGCAAAGCAATGCAAAGCTGCCACT |
| 12452_29 | 30 | [A/T] | CAAGAAAAGGAGGCAGCAGCCTGCTTTGTAGAAAGA |
| 12458_34 | 35 | [A/G] | TTAGCAGCAGCAGCAATTTTCTGCAGGTAATTGTAC |
| 12463_18 | 19 | [T/C] | TCCTGCACGGCTGCATGCTGCTGCTGGCACGGCATG |
| 12465_28 | 29 | [G/A] | AATTCACCGAATGCAACAAGATGCTGTCAGAAACAT |
| 12486_33 | 34 | [C/T] | GAAAAGCCTGTTGCAGAAAGGTGCCGAGCCCAACAC |
| 12487_3 | 4 | [C/T] | ACGCGTTTTAAAGCACACTCATGCTCCAGTCCTCGT |
| 12491_30 | 31 | [G/A] | GAATTTTCCCGAGCAAGTGAATGCGTAAACGTGGCT |
| 12492_27 | 28 | [T/A] | TTAAGTTTTGATGCATGTGGGTGCACTTCCAGTGAC |
| 12493_19 | 20 | [C/T] | CTGTTAGGCGCCGCAGGCCCTTGCCAGCTGTTTTCC |
| 12509_31 | 32 | [G/C] | TGTGTAGGACCTGCACAAACATGCACTTGTCGCCGG |
| 12518_1 | 2 | [T/C] | TTGCACAGATCAGCAGAACTTTGCGCTCTGTTTTGC |
| 12529_3 | 4 | [G/A] | TGTGTGCAGCCAGCATGGCCATGCTATTGGCAGACC |
| 12536_34 | 35 | [C/A] | TGTCCACCAGCCGCAATTCGCTGCCTCTTTCACCCC |
| 12538_10 | 11 | [C/T] | TAAAGCTGTACGGCATTCCTGTGCGCAGGAAGAGGG |
| 12540_32 | 33 | [C/T] | CAGAACCTGATAGCAGTCGGCTGCTACTGCCTCATC |
| 12553_17 | 18 | [A/G] | TGTGGAAGCCATGCAAGAAAATGCTGCAAAATGAAG |
| 12556_17 | 18 | [G/A] | ACATGTGTGGTCGCATTGAACTGCAGCTACATTCTC |
| 12558_16 | 17 | [T/C] | CAGTACAGCCAGGCAGTATAATGCCACGTTGCACAT |
| 1256_31 | 32 | [G/T] | ATACAGGACTGTGCAGTCTCATGCAAAGAAAGGACA |
| 12562_5 | 6 | [C/A] | GGGAACAGCAGGGCAAACAGGTGCTTCCTCTGAGAC |
| 12566_30 | 31 | [T/C] | GCCGGCCACCCAGCATGAATATGCCAAACATCATTC |
| 1257_26 | 27 | [C/T] | CTCTGACCCAGAGCAAATTCTTGCTTCCAACACATT |
| 12573_33 | 34 | [G/A] | GGTTAGTTCGGAGCAGCAACATGCTCTCTTTAAGAC |
| 12581_34 | 35 | [T/A] | TAAAGTGCCTGGGCACATTTCTGCCCTGTCTTTATA |
| 12584_30 | 31 | [A/G] | CCCAAAATTTCAGCAGGAAGCTGCCGTCGAAAAAAA |
| 12596_7 | 8 | [C/T] | GCGATCCCTTCTGCAGCGCGATGCCGTATCCCGTAG |
| 12599_34 | 35 | [A/T] | GCCCTGTAGGCTGCAGGTTCTTGCCTTTGGTCTTAT |
| 12600_30 | 31 | [A/G] | AAGGCTACATTGGCATCCCAGTGCACTGGCAGCACA |
| 12604_25 | 26 | [G/A] | TCACGTGCATTTGCAAAGCCATGCCGCAGAATTTGG |
| 12616_16 | 17 | [C/T] | GCGTGCTGTTCTGCATTGTTGTGCTCAGGCAGACAC |
| 12617_16 | 17 | [G/A] | ACACACGCTTGCGCACGCATTTGCCGTCTATGCACT |
| 1262_25 | 26 | [C/G] | ACACCTACTGTAGCACTCAACTGCTCCCCAGACACT |
| 12627_28 | 29 | [C/T] | GACTAGCTATGTGCAGCCAGGTGCCCCTCGTTGCTC |
| 12628_1 | 2 | [G/A] | TGGGGGAAACCTGCAACCTGCTGCACCACTGCACCA |
| 12631_17 | 18 | [G/A] | TAACCTTGGTTGGCAAGGTGGTGCTGCAGGAAACTT |
| 12633_17 | 18 | [C/T] | TTGAGTTGATTCGCATTCCCATGCTGATCTACTGAG |
| 12636_19 | 20 | [G/A] | CCGTTGCAACCGGCATTCAGTTGCACTTTGAAGTTA |
| 12644_6 | 7 | [C/T] | CTGTCCCATGCAGCAGACTGTTGCAGCAATATGAAC |
| 12645_5 | 6 | [C/T] | TCTACCGTGTTCGCAAGTTGCTGCATTTTTAGAAAC |
| 12649_18 | 19 | [A/G] | ACGCTGGACAGTGCAGGAACTTGCTCACTAACAGAC |
| 1265_15 | 16 | [T/C] | GTTTTCACTGCTGCATGGCTCTGCGTGTGATATTAC |
| 12651_32 | 33 | [C/T] | GCAATGCTGACCGCACTCCTGTGCCTGCCTTTCAAA |
| 12652_32 | 33 | [A/G] | CGGGTGGGGAAAGCAGGAGACTGCTGAGATGAAGTT |
| 12656_30 | 31 | [T/G] | GAAAGCCTGCCTGCACACCAATGCCTGTAGTGCTGG |
| 12658_3 | 4 | [T/A] | GAGTCTCATTGCGCAGTGCAGTGCATGAGAGATCTG |
| 12661_26 | 27 | [T/C] | TGTGATGGATAGGCACCCTAATGCCTTGTGGGTATT |
| 12663_1 | 2 | [A/C] | GCTGTATCACCTGCAGGTCCATGCTGGTAGACAATA |
| 12668_1 | 2 | [A/G] | TAGAATTCAAATGCAGTGGAATGCTTTTCGACCAAG |
| 12678_29 | 30 | [C/T] | TTGCCCTTCTGAGCAACAGCATGCAACGGCTGAAAT |
| 1268_4 | 5 | [G/T] | GCACGATATGCAGCACCACCATGCTGCCAAACTCAT |
| 12680_16 | 17 | [G/A] | TTTGGATGAATGGCATGAGCTTGCAGATGTACACAC |
| 12687_15 | 16 | [G/A] | TTTCTGTACAGTGCAGGTTCATGCCCGGGCTGTCCT |
| 12690_34 | 35 | [C/T] | TACTATAAGTGTGCATGCGGCTGCTATTCCCACACG |
| 12691_10 | 11 | [C/T] | ATAGTGTGTTCGGCAAACTCCTGCACCGTAGTGAGT |
| 12692_20 | 21 | [T/A] | TGCACTGACACTGCAGCTCATTGCTTTCCGCTGGGC |
| 12698_18 | 19 | [G/T] | GGTTTAGGTTCAGCAACCGTGTGCTCAAAGAATGAG |
| 12700_28 | 29 | [C/T] | GAGAGAAAAGCAGCACTGTCCTGCCGGACGTCTTAC |
| 12703_18 | 19 | [T/A] | CGCTTTATTGATGCAAATTGCTGCCAGGCTCTGTGA |
| 12709_29 | 30 | [G/A] | TACGTGTAAGAGGCATGGCTTTGCAGAAAGCTGCGT |
| 1271_29 | 30 | [G/A] | TCGCTTTTTGGAGCAGAAACGTGCAAAACGCTTCCA |
| 12712_32 | 33 | [G/T] | GTGTGTGTGTGTGCAATAGGATGCATGTTTGTGTAG |
| 12714_30 | 31 | [T/C] | CTATCAACTCAAGCACGTCTGTGCTGCTTGTGTGAT |
| 12719_1 | 2 | [T/C] | GTCCCATCTCTAGCATAAACCTGCTCACCTCCACAG |
| 1272_19 | 20 | [C/T] | CGGGCTGTAAACGCAGCCTCGTGCATTCTTCTCCGT |
| 12720_5 | 6 | [C/T] | AGGTCCGATACTGCATAGAAGTGCGAATCATCCTCG |
| 12723_5 | 6 | [G/A] | TGATCGTGAGTGGCAGAAGCTTGCTGGACTCATAAC |
| 12725_7 | 8 | [C/T] | ACACAACCTCTCGCATTTTATTGCCTGAAGCATTCA |
| 12726_9 | 10 | [A/T] | GGGAAAGCTAAGGCACTTTGTTGCTTTGGATGACCT |
| 12728_28 | 29 | [G/A] | CTGCAGTGCCCAGCACCGCTTTGCCAGAGAAAGGCA |
| 12729_28 | 29 | [C/T] | TCAGCACACAATGCAGAGTAATGCACCTCACCTGGG |
| 12731_10 | 11 | [G/A] | CTCTCTGTCTAAGCAGGACTATGCGAGACAACACTG |
| 12736_19 | 20 | [G/A] | AGAACAGACAAAGCATTGCGATGCCACGGCTCTTGG |
| 12752_24 | 25 | [T/A] | CCAAATTTCATGGCAGTTTGATGCTGAAGCTGATGT |
| 12753_18 | 19 | [G/A] | TGCGACTTGCTCGCATGCGTCTGCCAAAACAGGAGC |
| 12756_34 | 35 | [A/G] | ATGGGTGATGAAGCACATGGATGCGTAACGTTACAC |
| 12759_3 | 4 | [A/G] | TTCAGTGTTCATGCAGGCCGCTGCGTTTTCATGGCT |
| 12769_20 | 21 | [G/C] | TGGTATTCAATAGCAATGGTGTGCGAAGGCCTTAAG |
| 12770_32 | 33 | [G/A] | AAACACATTTCAGCATGGCAGTGCTTCTGTACGCAG |
| 12773_8 | 9 | [C/T] | AGGTGTCACCAGGCACACTGCTGCTAAAGGGTGACC |
| 12777_19 | 20 | [G/A] | CAGCTGGGTTACGCACAATGATGCTGCTACCACAAT |
| 12779_10 | 11 | [G/A] | TAAGACGCGCAAGCAACAACTTGCCAGAACATCCCT |
| 12785_32 | 33 | [G/A] | GTCTTACTTTCTGCAGTGTCTTGCTGGTCTTGGGCT |
| 12789_11 | 12 | [A/C] | GTTATTAAAGCAGCAATGGCTTGCCCATCCAAGAGC |
| 12791_34 | 35 | [A/G] | GGTGATGTGATGGCAGGTTGTTGCGTAATCATTTAG |
| 12793_2 | 3 | [G/A] | AGGATTCTCACTGCACTTTTCTGCAGTTCTGCAGCA |
| 1280_26 | 27 | [A/T] | GACAGCAAGTGAGCACAATCATGCTCACACACACAC |
| 12802_16 | 17 | [C/A] | ATTAGAGGAGGTGCAGCAGAGTGCTCAGCAGGAGAA |
| 12805_15 | 16 | [G/A] | CCCAGTGTCTGTGCAGCTTTCTGCCAAGGGTTGTAA |
| 12809_18 | 19 | [C/T] | ATCTGTGTATAGGCACTTCCATGCACTCGTTTTCAG |
| 12810_3 | 4 | [C/A] | CTGAACGCGCTAGCAGTCTCGTGCACCACCAGTTAT |
| 12811_11 | 12 | [C/T] | CACTTTCCTGACGCATCAGCCTGCACACACACACAC |
| 12812_2 | 3 | [A/G] | CTAGTGGGCTGGGCAACCTAATGCCAATGAGCTTGG |
| 12820_11 | 12 | [T/G] | GTGTGTCATTCTGCATCAAAGTGCATCAGCATTTAA |
| 12826_4 | 5 | [G/A] | AAGCGTGTACCAGCACCATGCTGCTTTTCCCACAGG |
| 12827_5 | 6 | [C/T] | TTCGACCCCGCAGCAGAACGGTGCCGACAGATTTTT |
| 1283_20 | 21 | [A/G] | GAACTACCGCCTGCACCGACATGCTGCCCAGATTGA |
| 12839_30 | 31 | [G/C] | TTTAATAAGCCTGCAGGACCCTGCGCTTTTGATTGA |
| 12840_32 | 33 | [C/T] | ATTGTGTACGTTGCACACTGTTGCTATGGAGACGAT |
| 12841_10 | 11 | [C/T] | ATCCACTTTTCTGCACGCGCATGCCGGAGGCCGCCG |
| 12842_32 | 33 | [A/C] | TGGCACGCTGTAGCACAGGCCTGCCGAAACCCACAC |
| 12847_15 | 16 | [T/A] | TAATCTAATTGAGCATCCCACTGCGAAACCCCTTGC |
| 12853_16 | 17 | [G/A] | CAAAGGCAGGAGGCACGAATATGCAACTAAGAGGAC |
| 12859_18 | 19 | [C/T] | AAATGCCAGCCTGCAGGTCAGTGCTTACTCATACAC |
| 1286_34 | 35 | [C/T] | TACTATGGCACTGCACTAACCTGCTGGTTTCTTTCT |
| 12861_30 | 31 | [C/T] | ATTTATTTTCCTGCACGATGATGCTTGACTCCTGCC |
| 12864_33 | 34 | [C/T] | AAGTGCCCATGGGCAAGTGATTGCTACCCAGGACGC |
| 12870_2 | 3 | [A/T] | AAAGTCCCAGGTGCATTTTGATGCTGATGCTGCCTG |
| 12874_1 | 2 | [G/T] | TGGTGCATTAGGGCAGTTGTGTGCTCACCGTTCAGC |
| 12881_5 | 6 | [G/T] | GTCTGGCCCTCTGCAGCACAGTGCCTTGTTCCTCAG |
| 12883_32 | 33 | [A/G] | CAGTACAACCCTGCAGATCCCTGCAGAACCCAATAC |
| 12886_31 | 32 | [A/T] | GTTTTGATGTCAGCAACCGTCTGCGTTCCGCTGCAG |
| 12899_11 | 12 | [G/T] | GGCTGTTATCAGGCACGCACCTGCAGAAAGTCTTGT |
| 12904_32 | 33 | [C/G] | TTGTCGCCCACTGCACCACCATGCTGTCCTGTCAAC |
| 12908_27 | 28 | [C/T] | AGCGGAAACTTCGCATCATCCTGCTAGCTGGCCTCA |
| 12910_16 | 17 | [G/A] | TACTGCCGTTCAGCAGGTTCTTGCTCATATCCACCA |
| 12911_25 | 26 | [G/A] | AGCAGAGCTATAGCAGTGGAATGCAGAGGTGCATAC |
| 12913_7 | 8 | [G/A] | CAGAAAGGGATTGCAGAGTAATGCCTTTGTTCGCTT |
| 12914_26 | 27 | [C/T] | TCCTTTCTTGCCGCACTTCCCTGCCTCCTCTGTTCT |
| 12919_16 | 17 | [G/T] | CATCCTGTCCAGGCATGTTCCTGCCCAGTGCCTGAA |
| 12920_31 | 32 | [G/A] | CTGTGGACAATGGCAGAAACATGCAGCCAGGGCAAT |
| 12922_33 | 34 | [G/A] | CTTTCCGGATCTGCAGTGTTCTGCCGCAAATTTGGT |
| 12923_34 | 35 | [G/A] | AGACAAACTACTGCAACTAAGTGCATGCACTGCTAA |
| 12924_19 | 20 | [T/A] | CCAGCATTCCGAGCAGAAGTCTGCCAGCTAGGAAGC |
| 12941_15 | 16 | [A/G] | CCTCCATTAAGGGCAGAGCAATGCCCTTGTGCTTTC |
| 12946_1 | 2 | [T/C] | CTGGTTCCTTTGGCATACCCATGCAAAAACACTTGA |
| 12951_5 | 6 | [G/A] | TGTATGAGCTCGGCAGGCGTCTGCGTGCACGGTGAA |
| 12957_25 | 26 | [A/C] | GAGGCGTATATTGCACGAGCGTGCAATTCCAGACCT |
| 12959_9 | 10 | [A/G] | GGGACTGAGACAGCAGCACTTTGCCTTTCTTTATCT |
| 12960_32 | 33 | [A/G] | CCCTCAGGGGCAGCAGACTGGTGCCCACAGAAATGT |
| 12962_2 | 3 | [A/G] | GTGAGATTTGGAGCACCTGCTTGCTGAACCTTCACA |
| 12963_8 | 9 | [G/A] | TCTGGCACGTTGGCAGAGGGCTGCCACTCACCCCCT |
| 12964_3 | 4 | [A/C] | GGAAGGAGGTGTGCAATGGAGTGCGCTTGAGTTCAA |
| 12974_30 | 31 | [T/A] | CGTGTGCTTTGGGCACATTGCTGCACAGAATTGTGA |
| 12976_5 | 6 | [G/A] | CTTCAGTCTACTGCAGGCATCTGCCTTTGATTCACA |
| 12977_30 | 31 | [A/T] | AGACTTGAACCTGCAACACACTGCTGGTTCACTGAG |
| 12980_16 | 17 | [A/G] | GGGAGGAAGGACGCACACGGGTGCCGTAAATGGATT |
| 12994_29 | 30 | [C/T] | GTGTTTTACAGAGCAGAAGGTTGCGGGTTTGATTCC |
| 12995_26 | 27 | [C/G] | TCTCAACTAAGAGCACTAAAATGCAGCAAGGTGCAT |
| 13000_2 | 3 | [G/A] | ACGCACGCACACGCACATCTGTGCCGGGTTCTTCTC |
| 13002_33 | 34 | [C/T] | GACAGCTGAATCGCATCACCGTGCGAGTCCCTTCAT |
| 13006_30 | 31 | [C/T] | GGAGGGAGGGGAGCAAATCGGTGCTCTGTTCAGGAT |
| 13008_31 | 32 | [C/T] | AAGAAAGCATAAGCAGACGCATGCGCTGTCATGCTG |
| 13010_29 | 30 | [G/A] | CCTAGACAGCTAGCATTTACCTGCTGCGAGAAAGGG |
| 13011_19 | 20 | [C/T] | ATAGTTGGTTTTGCACTGGCCTGCTGTTTCCTTAAA |
| 13013_4 | 5 | [C/T] | ATTACCAATCTGGCAGTTCTCTGCGCTTTTGTTGTG |
| 13014_9 | 10 | [G/T] | ACTTCATGAGGTGCAAGCATCTGCCAAGTTTTGCCA |
| 13015_4 | 5 | [G/A] | CCTTGAGGTGCTGCAGCTGTCTGCGATCGTCCTCCA |
| 13020_19 | 20 | [G/A] | TGTTAGATGATGGCAGGCTGATGCATTTCCGTTTAA |
| 13022_3 | 4 | [C/T] | GTACGGCAAAATGCACTCATCTGCATAGCTGTCACA |
| 13027_8 | 9 | [G/A] | GGACCATGGAGAGCAAGACCTTGCCGCTCGAAAAGA |
| 13030_4 | 5 | [C/T] | CTGGCAGATTCGGCAATGCAATGCAGTCAATGGTAT |
| 13036_4 | 5 | [G/A] | ATCTGATACTTTGCATACGGCTGCAAATGTTGCCAT |
| 13039_7 | 8 | [C/A] | TTCTTAGCAACAGCACGAACGTGCCGAGTAGAAAAA |
| 13040_28 | 29 | [C/A] | TGGTAAAAGCCAGCAAAACAGTGCCATACCAACCAG |
| 13041_24 | 25 | [G/C] | TCGGGTGCACTGGCATGGTGTTGCGCTTGGGCAACA |
| 13043_20 | 21 | [C/A] | ACCAAGAAGCTTGCATGCCTCTGCTACGAGACGCAT |
| 13051_16 | 17 | [A/C] | TCACACTAAATTGCAAAATATTGCGCACGCTCTCAC |
| 13056_8 | 9 | [G/A] | GCTGTTATGAGTGCAGCTGAGTGCAAAGAGACTGGA |
| 13059_11 | 12 | [T/C] | TTGCCACGCCCCGCAAATCAATGCCATTCATTTGCA |
| 1306_18 | 19 | [C/G] | AGGTCATCCAATGCAGAGCACTGCAAAATTTCTGGG |
| 13067_19 | 20 | [A/G] | CAGTGGACGGACGCAGCCGATTGCCCACTCCAACTA |
| 13068_10 | 11 | [C/T] | GGCCCACTAGTAGCAGGCTGTTGCATGGGCAAAAAA |
| 13071_31 | 32 | [G/T] | TGGCTCCTCTCAGCACTGATGTGCCCATTCTGTCCT |
| 13075_33 | 34 | [T/A] | CATACGACACCAGCATGTCTCTGCTGTTACATTTAA |
| 13078_34 | 35 | [T/C] | ATTTCTGTACTCGCAGACATTTGCATATGACAGCTC |
| 13083_31 | 32 | [C/T] | GGTTCTCCTCCTGCAGACACTTGCACTTGAGCCGGA |
| 13087_20 | 21 | [C/T] | CGGGGCGAGTCGGCACCTGACTGCTCGACAAAAGCC |
| 13091_31 | 32 | [A/G] | CATTCAGCCTGTGCATGTCTTTGCATGCTTTATTGG |
| 13105_6 | 7 | [T/A] | TTTCGCTTTTTCGCACCATTCTGCGTACCCTCTTGA |
| 13106_24 | 25 | [T/C] | GCTGATCGCGTTGCAAAAATGTGCTGCACTGAATGT |
| 13113_8 | 9 | [C/T] | CGTCCACCCCGAGCACCTTACTGCGTGTAAGCAGAT |
| 13119_6 | 7 | [C/T] | GAGATACGTGTGGCACAGACGTGCTCCACATCTGCT |
| 13122_31 | 32 | [G/C] | TAGATGATACTAGCAGCTTGTTGCTGTCAGTGCAAT |
| 13125_34 | 35 | [C/T] | ATAGTGCAAGCAGCACCCGCGTGCTGCTCTGATCCA |
| 13131_19 | 20 | [G/A] | TGGCCCACAGGTGCACTCAGTTGCCTTTAACATGAA |
| 13132_34 | 35 | [C/A] | ACTGTGCGGCTCGCAGCCCTGTGCCATTTGACCGCC |
| 13134_32 | 33 | [T/G] | TACACATTAAGCGCACGTTTTTGCGTTTAACCTCAA |
| 13138_25 | 26 | [G/A] | TTGTTTTGAATTGCAGATGGATGCGGATCCTGCATG |
| 13140_31 | 32 | [G/A] | CTTCAGGAGACTGCACAAACATGCATGAGGAGAAGT |
| 13146_29 | 30 | [C/T] | TTCGTCTGTAGTGCATTCTATTGCGCTTTCTTGTTG |
| 13147_28 | 29 | [C/T] | GTGGAGATCAGAGCAGTTTGGTGCCTTACTTGTGTT |
| 1315_29 | 30 | [A/T] | GTGTGTGCACGTGCATACTCATGCATGTGAGTTGAA |
| 13156_25 | 26 | [C/T] | CATTGCTATGCTGCACAAGTATGCACACACACACAC |
| 13159_20 | 21 | [G/A] | ACATTTGCCATGGCAAGGGCGTGCTGTACTGCTTAC |
| 1316_11 | 12 | [A/G] | ATATGAATTCTAGCAGTGGCGTGCACAGACATTTTG |
| 13169_1 | 2 | [A/G] | GAGAGAGCAGTAGCAGCCGTTTGCCGGCTCTGTGTT |
| 13171_30 | 31 | [C/T] | CAACAGCCGCCCGCACTGCTGTGCAGTCTGCGGTAA |
| 13174_2 | 3 | [T/G] | TATATATGAGCAGCATGGTGGTGCCAAAGGTAGTTT |
| 13184_11 | 12 | [C/T] | AACCTGCCTTACGCACAAGAGTGCAGCTTACTGAAA |
| 13185_19 | 20 | [G/C] | AATCCCACGGAGGCATACTGCTGCAGGCTTGCGATG |
| 13187_17 | 18 | [C/T] | GTAACATCTCCAGCACCCCTGTGCCAACCACTGATC |
| 13188_9 | 10 | [A/G] | TGGCACTGTAGAGCAGATGTCTGCGTCTCAGCGGGG |
| 1319_33 | 34 | [C/G] | CAAACACCACTAGCATCAAAATGCCCCTCATTACCA |
| 13190_28 | 29 | [C/T] | GATTTGATCCTGGCACAAGCTTGCCATTCAGCCTCT |
| 13191_24 | 25 | [G/A] | AAGAAAGCACCAGCAACTATATGCGTGTGTGTACGT |
| 13192_10 | 11 | [G/A] | CACTGTAGCTGTGCACCTCTGTGCACCTTCTAATCT |
| 13193_10 | 11 | [G/A] | GTGACACAGAGAGCATGCATCTGCACAGCGTCTCAA |
| 13194_16 | 17 | [C/T] | GCGCAGCACAGCGCAGCACAATGCCAAAGCCAAATT |
| 13200_34 | 35 | [G/A] | GGTGACATATTTGCACACACCTGCTTACCGTGTCGG |
| 13202_9 | 10 | [C/T] | AAAGGGAAACGAGCAAATCTGTGCACGCCTGAACAT |
| 13205_15 | 16 | [A/G] | CATCCAGTCCTGGCAACATTTTGCTGAAACCTACAT |
| 13206_19 | 20 | [G/A] | GTCTGTGTATCTGCACTTCGATGCACTGGTGACCTG |
| 13207_27 | 28 | [T/C] | AGTAGAAGTGGTGCAGCTGCCTGCCAGTGTGAGATA |
| 13209_32 | 33 | [T/C] | TTTTTTTTCGCTGCAAGAGGGTGCTGCCATATTGGC |
| 13211_32 | 33 | [G/A] | TAGTAGTGGGCAGCACAGCAGTGCCACAAGTAGAGT |
| 13213_18 | 19 | [A/G] | GGAAGAACTCCGGCACAGAGCTGCTGCCTGACACAC |
| 13214_29 | 30 | [C/T] | AATAAAGGACACGCAAGTGGCTGCAGCTCCAGCCAG |
| 13224_33 | 34 | [C/A] | TTTGATTGACAGGCATCAGCATGCAATTCCATACAT |
| 13227_26 | 27 | [G/A] | CTATGCTAATCAGCACTTTACTGCTGGCACAAGGTC |
| 13228_15 | 16 | [C/T] | GAAAGGAAGTTGGCACGCTGCTGCCGGTGTTGATGC |
| 13229_20 | 21 | [G/A] | TAAAAGATCTCTGCAGAAGCGTGCTCCAGTGACACT |
| 13237_32 | 33 | [G/A] | CTCACGGATGCTGCAGCAGCGTGCTGGTACACGTCT |
| 1324_2 | 3 | [C/T] | ATCACCAGTTTTGCATCAAATTGCTCTCCAGCCACT |
| 13243_2 | 3 | [A/T] | TTAAAACTGCTGGCACATACCTGCATGATTTTATGC |
| 13244_5 | 6 | [C/A] | TGTTGCTTTAGGGCAACACCATGCCATAGAGGGTTA |
| 13247_20 | 21 | [G/A] | AAACGCTACCATGCAGTGGCGTGCCTCGACCAGAAG |
| 13252_6 | 7 | [C/T] | GCGGTTCATATGGCAATGCGTTGCACAAGTGCAATC |
| 13253_30 | 31 | [A/G] | TATATCTCACAGGCAGACTGGTGCACGTGTATTGAG |
| 13254_16 | 17 | [G/T] | CGTGGTGTATGTGCAGGAGGCTGCAGTTCCTCCTGT |
| 13264_7 | 8 | [T/A] | GAGGAAATTTCAGCAAAGGTGTGCACAAGTCAGTCC |
| 13265_27 | 28 | [C/A] | TCAGGCTGTCTTGCAATTCTTTGCCAACAGTGAATG |
| 13266_18 | 19 | [G/A] | CCTGCTACAGAGGCATAGGTTTGCTTGCCGACGAAT |
| 13267_32 | 33 | [G/T] | CATGGCGTGGGTGCAGATGTGTGCAGAGGCTCGGCA |
| 13268_4 | 5 | [C/T] | ACTACACCATCAGCACTGACATGCCCGGGCCAGTAC |
| 1327_17 | 18 | [G/A] | AACCTGAGACATGCAGAGAGATGCACAGAGACACAT |
| 13272_33 | 34 | [T/C] | CGTGGTCCCTGTGCACAGTTTTGCCCTTTTTACTCT |
| 13273_16 | 17 | [G/C] | CATGTCGAGACAGCAGGGTCCTGCTGAGAACTGCAC |
| 13274_31 | 32 | [C/T] | CTTTTCTTTACAGCATTTCGTTGCATGAGGGCGCTA |
| 13277_7 | 8 | [A/T] | TAGAGTCATACCGCAGTGCACTGCTGGGTTTTTCCA |
| 1328_11 | 12 | [T/C] | TAGAAACATTGTGCAACCTCATGCCCAAACCACAAT |
| 13287_10 | 11 | [C/A] | ACAGAAATAACGGCAGGAGTCTGCTGGCACAGGCAG |
| 13296_26 | 27 | [T/C] | TTATCGTGCCATGCAGTAACCTGCCGTGCATCACTC |
| 13298_33 | 34 | [T/A] | CACACGCAGATAGCACAGGTTTGCAAACCCTCCTCC |
| 13304_24 | 25 | [A/G] | CAGTAAACTCCTGCATTTTTATGCACACCCCGTCTC |
| 13305_26 | 27 | [T/A] | ATCTATGGCACAGCAGGCGTTTGCTCTCGCTCCGTC |
| 13309_10 | 11 | [C/A] | GGTGGTAAGGCAGCACAGTGGTGCAGCAATTGGTGC |
| 13310_25 | 26 | [G/C] | AGCTAGACTCCCGCAGGTCACTGCAGCACTTGATGG |
| 13311_29 | 30 | [G/A] | CAGTGTCGCCATGCAAATCCCTGCGTATCGCGGTTA |
| 13319_34 | 35 | [G/C] | ATGTGTGGAATGGCACGGTGGTGCCACAGGCACTGA |
| 13320_16 | 17 | [C/T] | GGGCCTTCGAGCGCACCGGCCTGCTGCGCTTTTTTG |
| 13322_25 | 26 | [A/T] | GTGTAGGAATGAGCATGACGATGCCTGGGACCTGCA |
| 13324_26 | 27 | [C/T] | GGCAAAGAACTGGCACCTGGATGCTCCCACGGGTAC |
| 13325_24 | 25 | [A/T] | TATGGACTTAACGCAGGAATGTGCAATGGGCAGGGC |
| 13331_8 | 9 | [C/T] | AAAGCCACCCGAGCACCTGACTGCCCTTTGGACCCA |
| 13333_3 | 4 | [G/T] | ACTTAGACCAGTGCACAGGGCTGCTCACTGCTCTGG |
| 13342_33 | 34 | [G/T] | ATAGAACCAGTCGCATGGAGTTGCGTAACACCTGAA |
| 13349_16 | 17 | [T/C] | GGGCAGAAACGTGCAATAACGTGCTACACGTCTCAC |
| 13356_33 | 34 | [C/A] | GGTAATGTTGGGGCAAATTCCTGCACTGACAGACCG |
| 13362_24 | 25 | [T/A] | ACCATCACCATGGCAACATGATGCTCTTCGGTATGA |
| 13366_28 | 29 | [A/G] | TAAAGGGAAAACGCAAGCTATTGCATGCATGCAACG |
| 13369_4 | 5 | [G/A] | ATGCGTCCATTAGCACCATGCTGCTAACGATAACCA |
| 13372_28 | 29 | [T/C] | CACCCTCCTCTCGCACACGGATGCTCTGTAGGAGCT |
| 13377_16 | 17 | [C/T] | GGCGGGGTCGAGGCATCGCCATGCCGCCTGGATGAT |
| 1338_15 | 16 | [G/A] | TTTCGTATATACGCAGATTTCTGCACCGAGTCAATC |
| 13384_5 | 6 | [C/A] | TGGCACTAGGGAGCAGCTAGGTGCCTTGCTCAAGGG |
| 13385_33 | 34 | [C/G] | CGAAGCAGCAAAGCAGCATCGTGCATGCCCTGCCTA |
| 13386_18 | 19 | [T/C] | GTCATTTCTGCAGCACAATAGTGCCGTCCTTTGACT |
| 13388_33 | 34 | [C/T] | ATGTGTAACACTGCACTATCCTGCCATAGAACCCCA |
| 13389_26 | 27 | [A/G] | GACCCAGGTTGGGCAAAGTGCTGCGCATTTTAGCTT |
| 13395_31 | 32 | [T/C] | GAGTCATTCCAAGCAGGGTAATGCGATCCTGTACGT |
| 13398_25 | 26 | [G/A] | GCAGCCACTGGAGCATGATGCTGCCGGCACTTCAGT |
| 13402_2 | 3 | [G/T] | TCGCTCCGGAGCGCATCTGGTTGCCATGTCATCTGT |
| 13406_34 | 35 | [G/A] | GCAGTCCCTGAAGCACACTGCTGCGAGAGTGTGAGA |
| 13408_16 | 17 | [T/C] | GCACTTCCTGTGGCACTACTGTGCCACCCTATTATC |
| 13415_31 | 32 | [G/A] | CCTTGGCATTAAGCAGGGGTCTGCTCTTACCGAAAT |
| 13416_24 | 25 | [C/T] | ACACTACATGCAGCACCACTGTGCCACCCCAGCTAA |
| 13420_7 | 8 | [C/T] | GGCAGAGCCGCAGCAGGAATCTGCGAAGCATCACAG |
| 13421_30 | 31 | [T/C] | GCATGGTGCCCTGCAATGGAGTGCATCCTATTCAGA |
| 13424_30 | 31 | [A/C] | ACCAGACCCAAAGCAAAATGCTGCCACCACAGTGCT |
| 13426_31 | 32 | [G/T] | ATGAAAGCTTAAGCAGAGAGCTGCTCCAAAAGCAAC |
| 13430_31 | 32 | [C/T] | AGTTGAACATCGGCAATGTGTTGCCTGTTGGCGTCA |
| 13431_29 | 30 | [A/G] | TGGGTAGTAGAGGCAATGCCTTGCAACAGATCATTG |
| 13437_26 | 27 | [T/G] | ACCACATCCTATGCACCAGGCTGCACTGCTTAAATG |
| 13438_5 | 6 | [T/C] | TATATTGGGGAGGCACAGTGGTGCTGCAGGTATCAC |
| 13440_18 | 19 | [T/C] | ACCAGTATTAGTGCAGACCGATGCAGTCTCGTGGCA |
| 13448_6 | 7 | [A/C] | CACCAAAATAAAGCAAGCCCATGCACCAGACTACTG |
| 13450_15 | 16 | [T/C] | GGCACTAATCGTGCATCCTGGTGCCAGATCTTCCCC |
| 13452_28 | 29 | [T/C] | TCAGAAACGCAGGCAGAGCAATGCAGCCTAATGATG |
| 13456_33 | 34 | [G/A] | GAACCGAACGGCGCAGCTGCCTGCTCCTAATCAGCA |
| 13460_28 | 29 | [A/G] | TGTTTATCCCCGGCAGATTGATGCTTTCAGTCTAGA |
| 13461_9 | 10 | [T/C] | TGGTCTAAATCGGCACACTGCTGCAAAACTTCACGT |
| 13462_33 | 34 | [C/T] | ACTTGCCAGTGGGCATCATAGTGCCTGGGGATGCGT |
| 13471_16 | 17 | [G/A] | GCATGCGCGGTCGCAGGAGTTTGCTGGTCGGATGTT |
| 13482_30 | 31 | [A/C] | TGGTGACAGGGTGCAGTTGAATGCTGCAGGATGTTT |
| 13483_18 | 19 | [C/A] | AGCCCTTAAAGTGCACAGCACTGCCCCTACTCTCTT |
| 13485_18 | 19 | [G/A] | CTCTACGGTTTGGCACACGGCTGCAGGATGCATTTT |
| 13488_4 | 5 | [A/G] | ACACAACCTGTGGCACCGCAGTGCCATCTTATGTTT |
| 13491_11 | 12 | [A/G] | AGAACGGCCAAAGCAATAACATGCCATACGTGGTCA |
| 13498_33 | 34 | [C/T] | GTGTGGCACCCAGCAACAAATTGCCACCATGTCCCG |
| 135_30 | 31 | [G/T] | TAAACCTCAAAGGCACATTCCTGCTTTCAGGTTTTT |
| 13502_33 | 34 | [T/C] | AAGGGTGAGGAAGCAGCTCATTGCTTTGGAGGATGT |
| 13511_8 | 9 | [G/T] | AGGAACAAGACTGCAAACATCTGCTTCTGGGCTCTG |
| 13524_32 | 33 | [T/C] | CCAGAGGATCTGGCAAGCTAATGCTGGCTTCCTGTC |
| 13525_4 | 5 | [C/T] | ATTGCTGTCTGTGCAAAGTCTTGCATGTGCATCTAA |
| 13532_34 | 35 | [G/C] | GCTGATGGATAAGCAGAGACCTGCAATGTGCATGGT |
| 13536_33 | 34 | [A/C] | GATCATGCCCTGGCATTCGCATGCAGAGAACTCAGT |
| 13538_1 | 2 | [G/A] | GGCATCAGCACAGCATGTCATTGCTGAATGAAACGT |
| 13548_10 | 11 | [C/G] | ATTTGGCAGACGGCATTGATGTGCCCACATGCTGGA |
| 13552_18 | 19 | [G/A] | GTTTTGTGAAATGCATCCGAATGCAAATTCAGTCGC |
| 13562_17 | 18 | [A/T] | CAGGGCAAGAGTGCAGAAATGTGCATGCATTAGCAA |
| 13564_5 | 6 | [C/A] | TCCGCCTGTCTTGCACGACTCTGCTGCTGTCTGCTC |
| 13570_8 | 9 | [T/G] | AGTAGAGATGAAGCAGGGTTGTGCACTTTGACGAAA |
| 13571_9 | 10 | [G/T] | GCGCAGGCCGGAGCACCGAGGTGCTCAGCTCGGAGT |
| 13573_28 | 29 | [C/T] | TGGCACAGCGTGGCACGTGCCTGCATAGCAACAGCG |
| 13576_24 | 25 | [C/A] | TGACTAGGAGTTGCATGACGCTGCCTGAAGTTCAAA |
| 13581_25 | 26 | [T/C] | TGATTGAAAGAGGCAGAGAAGTGCGTGTGTGTGTGT |
| 13586_34 | 35 | [G/A] | CTTGGAAAAATCGCAGCACTCTGCAAACCCACTTGA |
| 13587_26 | 27 | [A/G] | GAGGCTGATCTGGCATGCTGTTGCGTAGTTCTGTCA |
| 1359_10 | 11 | [G/A] | TCTGATGACGGAGCATTACAATGCTGACAAAGACCA |
| 13591_7 | 8 | [A/G] | TGTCAGGACGCAGCAGGAGGATGCGCTGAACACCGG |
| 13594_25 | 26 | [T/C] | AGAAACCATTATGCAAAATGCTGCATGGCTGAAACA |
| 13596_9 | 10 | [G/A] | CCAACTTTGGATGCACTACAATGCGCTTGTTCTTCA |
| 13599_34 | 35 | [C/T] | ATGTTCAAGAGTGCAACAATGTGCCTCAGACAAACA |
| 136_33 | 34 | [C/G] | AGCACGAAAAAAGCACCTTTATGCACACAAGCACAG |
| 1360_17 | 18 | [C/T] | TAATGACGTAATGCATGCAAATGCACGGGTTCAGGA |
| 13600_32 | 33 | [T/A] | TCTGCAGCAGGTGCATGGTGTTGCTCCTTCAATCAC |
| 13604_34 | 35 | [C/T] | AGCGCAGGTCCGGCAGCGGCATGCCGAGCATCTTCT |
| 13607_31 | 32 | [C/T] | ACCCACAGGCATGCAACGCCATGCTGGACTGCGCCG |
| 13608_15 | 16 | [T/C] | GCCATTTGACTGGCATAGTCGTGCACCACAGACCCC |
| 1361_18 | 19 | [G/C] | GCAACGGCTGGGGCACGCGATTGCTCGAATTCATTC |
| 13610_11 | 12 | [A/T] | GACTGAGAGGCAGCAGGTCTTTGCTGACTAAACAGA |
| 13617_3 | 4 | [T/C] | ATCTGCCAAGCAGCAAACTTTTGCAACAGAGTGGTG |
| 13618_19 | 20 | [G/A] | TAGCACTGGGTGGCACGGTGGTGCCGCAGGTAGTGA |
| 1362_18 | 19 | [C/T] | GTGTACATCTCTGCACTGCCATGCTCATAGCCATGC |
| 13620_11 | 12 | [G/T] | AAGTCGAACGAGGCACTGTAGTGCTAGTGATAAACC |
| 13621_32 | 33 | [C/T] | CCAGTACATTTGGCACGGCGCTGCTGAAACGACTAG |
| 13623_1 | 2 | [T/C] | ATACACCACTCTGCATACACATGCCTGTAGCACTGC |
| 13624_1 | 2 | [G/A] | CGATTCACGCCAGCAACGGGATGCCTAAAACTAGAG |
| 13626_11 | 12 | [G/A] | CTATTGAGACCGGCATGAGGATGCAAGAGAATGGAG |
| 13628_19 | 20 | [G/A] | CTAGCCAGCTCTGCACAAGGCTGCGAAATATCATTC |
| 1363_24 | 25 | [C/T] | CTGTGTAGCGAGGCATTCGTATGCCGACAATTAAAC |
| 13632_27 | 28 | [C/T] | CCCTTACAGGGAGCAGGGAGGTGCAGTCAGTAACAT |
| 13635_26 | 27 | [G/T] | ATACAGAGACATGCAAGATCCTGCGTTGTCGTTTAG |
| 13636_8 | 9 | [C/A] | GGGCGGCTCGGAGCAAACACCTGCTGGAAAACAAGA |
| 13638_25 | 26 | [A/G] | AAGCTGAGATCTGCACTAAAATGCAACTCACCCACA |
| 13642_27 | 28 | [C/T] | CCTACCCACCCTGCAGGCTCGTGCCTTCGGCGGATC |
| 13645_18 | 19 | [A/G] | CCTGGGCACTGTGCACAGACATGCCCACCACTCTGG |
| 13648_28 | 29 | [G/A] | CCACTATGAGCTGCAGCCCGGTGCCCAGATTGTGCC |
| 13651_20 | 21 | [C/G] | AGGCGGGTAAGTGCACGGTGCTGCTGCCGTGGACGG |
| 13654_7 | 8 | [C/A] | CTCCACACACACGCACCGAATTGCTGGATGGCTCGG |
| 13659_33 | 34 | [G/A] | GGAGCAGGAGGAGCACTGGGCTGCTGAGGGTCCGGA |
| 13662_20 | 21 | [G/A] | AAAACCTCAAACGCAGCAGCGTGCCCTGACTTCAGG |
| 13663_1 | 2 | [C/T] | TCTTGGGCATTTGCATATTGCTGCACAGGTGTGTAC |
| 13664_16 | 17 | [G/A] | ACTGGATCGCAGGCAGGATTATGCTATTGACTAGAC |
| 13668_8 | 9 | [C/T] | TCATCATTCAAGGCATGTCTGTGCCAAATGCCACAA |
| 13672_31 | 32 | [C/T] | AAAATGATCGCAGCATCTTAGTGCACTTCGCCTAAG |
| 13673_29 | 30 | [C/T] | ATGTGTTGTGCTGCAGGGATTTGCCTCCTCACTGAC |
| 13675_1 | 2 | [C/T] | TCTTTACAGCCAGCACTCCTGTGCGCACTTCTCCTG |
| 13678_6 | 7 | [C/T] | ACGGGACAGCTGGCAACCCTATGCACGGCAGATCTC |
| 13679_19 | 20 | [A/G] | ATGCAGTCAATTGCAGTCAACTGCAGCAGTTTCCAC |
| 13689_4 | 5 | [G/C] | AACTGTGGGGTGGCATGGAAATGCAGGTGTTCATTA |
| 13692_17 | 18 | [A/G] | AGGTTTTAGCGTGCATCATGTTGCTGTGCTGCGTGT |
| 13694_7 | 8 | [T/C] | GGCAGCATGTTGGCACTGCACTGCTGAGGGACATGA |
| 13695_11 | 12 | [T/A] | CAAACAATGCAAGCAAAACAATGCGTCTGTACACAC |
| 137_33 | 34 | [C/T] | AAATACACACTTGCACATACATGCATGCGCTCACAC |
| 13702_32 | 33 | [T/G] | TGCACATACAGGGCACACGTCTGCCCTAAAAAGGAA |
| 13705_18 | 19 | [A/C] | TAAACCCAGGAAGCATTGAAGTGCTAAACTGTCACA |
| 1371_25 | 26 | [T/C] | CGGAGTTGATGAGCAGACGAGTGCGTCAGTATCTCG |
| 13712_4 | 5 | [C/T] | TATTCCAGGAGAGCAACAAGATGCATGGTAGCCACT |
| 13713_27 | 28 | [G/A] | GCTTAAGCAGTGGCAGCTTGATGCTGGGATTCGAGC |
| 13714_24 | 25 | [A/G] | GTGGAGTGAAACGCACATATCTGCACACACAAGTGA |
| 13716_24 | 25 | [G/A] | CTACAGGACACTGCACTGACCTGCGGGATTTATATG |
| 13719_34 | 35 | [G/T] | CAGCTGCGATCAGCAAGTATATGCACAAGCTTTCGG |
| 13720_24 | 25 | [C/T] | TCCTGCGTCTCTGCAAATTCCTGCCGCTCTTCGCAT |
| 13724_28 | 29 | [G/C] | GGCACCCTGACAGCAAATCACTGCATCTGTGTGTGT |
| 13732_26 | 27 | [C/A] | GTTCCTGGGCTGGCAAACAGATGCACCAGGCCATTG |
| 13735_28 | 29 | [A/T] | GGTGCAAAAGCTGCATCTTTGTGCTACAAAACGCAC |
| 13736_16 | 17 | [A/G] | GCACACCGTCCTGCACATTTCTGCTTATAACCATGT |
| 13742_16 | 17 | [T/A] | CTGAACTGTCCAGCACTCCGTTGCCGTCTCGGTCGT |
| 13748_33 | 34 | [G/T] | CAATGATTCATAGCACAATCCTGCCCTGTACGAGCC |
| 13755_24 | 25 | [G/A] | GCCCCTGTTGCGGCACTGTTCTGCAGGAGGGGTGAG |
| 1376_29 | 30 | [T/C] | TTGGCTACTTTAGCACAGCGCTGCTTGCCTTTCCAA |
| 13764_27 | 28 | [A/T] | TTTTCAAGTGCTGCATTACCTTGCCTTAGCACATGT |
| 13768_25 | 26 | [A/G] | ATCGAAAGCGATGCAACATCATGCGAAATACAAATC |
| 1377_32 | 33 | [T/C] | GTGTATACAAAAGCACTCAAGTGCAGCTGGGATGAA |
| 13772_27 | 28 | [C/A] | TCTGACATACCAGCACATGCATGCGCTCACACACAC |
| 13780_32 | 33 | [A/G] | ATGTCAGCCCAAGCAGATCTTTGCTGCTTTATATCA |
| 13790_18 | 19 | [C/T] | CAGACCCACAGCGCAAACCGTTGCGTAAACGGATGA |
| 13791_29 | 30 | [T/G] | ATTTGGGTCCCAGCAATAGACTGCCACTGTTGGACC |
| 13793_26 | 27 | [G/A] | TCACGACCCAGTGCAGCATAGTGCTGGAGCCCATTA |
| 138_8 | 9 | [G/A] | GTCAGGGTGAAAGCAAGGGAATGCTGGGATGGTTGT |
| 13800_1 | 2 | [C/T] | ACGGATTAAACTGCAATTAGCTGCGCGGTGGGTGAT |
| 13803_32 | 33 | [G/A] | GATTGCGACTTAGCAACATCATGCTGTTCGGTGTGG |
| 13809_7 | 8 | [T/G] | GTGAGTGGGAAAGCAAACATGTGCTTTTGCCAAAAA |
| 13815_29 | 30 | [C/T] | TCGCCTCCATCTGCACTCACCTGCCTCTGCCTTACG |
| 13820_18 | 19 | [G/A] | ATGTATATCACAGCACCAGCATGCAGTCAAGACACA |
| 13821_24 | 25 | [C/A] | TAGCTATGAGTGGCAAAAGTATGCCAACCTTTCCAT |
| 13827_10 | 11 | [C/T] | GTGGGGAACTCAGCAGGAACGTGCTGTACTTTAACA |
| 13828_8 | 9 | [G/T] | GCCCGTTTGCCAGCACTTATTTGCCGTTTTCTCTTG |
| 1383_17 | 18 | [T/C] | TCATAATGCACCGCATGTCGTTGCCAGTCCACATGG |
| 13830_28 | 29 | [A/G] | TCCCTATGATGCGCACAACATTGCGTTGAATCTTCT |
| 13832_17 | 18 | [G/A] | CCCTCGCACGCTGCAGCGTTCTGCACAACCGGCGGC |
| 13835_7 | 8 | [G/A] | GTACATCGCTGGGCAAGTGCATGCTGGGAAAAGGGG |
| 13841_17 | 18 | [G/C] | GGGCTTATTATGGCAGGGACTTGCAGCATATGGCAA |
| 13847_25 | 26 | [T/G] | TGGCATTTCAGCGCATTGTGCTGCTTTAATCAACAC |
| 13849_16 | 17 | [C/T] | GGAGGCCCGTGAGCAACGCACTGCAGACTCAGGCTT |
| 13851_32 | 33 | [A/G] | ACTGTTTTGGTGGCATGGTGATGCCAGAGATCACGG |
| 13852_5 | 6 | [T/C] | GCGTGCGCTCGGGCACCCACGTGCTGTGAGCGGATG |
| 13853_10 | 11 | [G/A] | GCGGTCACGGGAGCAGGGGACTGCGGAGGAGGTGCG |
| 13859_8 | 9 | [C/T] | TTTTGCACCGAAGCATAGTCATGCTGGAACAGACGG |
| 13865_33 | 34 | [G/A] | CAGCAATTAGCAGCAAACTGGTGCAAACAAACAGAG |
| 13868_24 | 25 | [A/G] | GATGGTGACTGGGCAACAATCTGCAATGACAAACAA |
| 13874_7 | 8 | [T/C] | GTGAGCGTGCGAGCACGCTAATGCGCAGCAGCGACG |
| 13876_33 | 34 | [G/A] | AGCACCCAGGGGGCAGTTATTTGCCTTGCCCAAGGG |
| 1388_18 | 19 | [C/T] | ATAACGTGAGCAGCATACCAGTGCCAATATCTCACA |
| 13883_32 | 33 | [C/A] | TAATGACAGAAAGCAGCAGTGTGCAGCAGTATCTCT |
| 13886_16 | 17 | [T/C] | AGACTTCCTGTGGCACTTTTGTGCTGGTGCTCAGGG |
| 13888_27 | 28 | [C/T] | GAGGAAACCGGTGCACTCAAATGCACTTTTGTATAT |
| 1389_27 | 28 | [G/A] | AGGCCTCGTGCTGCAGCTGCCTGCTCAGGCTTTCTT |
| 13892_4 | 5 | [C/T] | TCTCCACATAAGGCAACATCCTGCGTTTAGATGGCC |
| 13893_3 | 4 | [T/G] | ATTTTGCTTCATGCACTGAGGTGCAGTCATGCAGGA |
| 13900_17 | 18 | [C/T] | ATGATGAACTCAGCAAACGTTTGCTACTCTCGTGCA |
| 13901_16 | 17 | [A/C] | ATTTACTGTTCAGCACAAAGCTGCCAGTTTCTCCAT |
| 13902_27 | 28 | [A/T] | ATACACATGGCCGCAACTCAATGCATTATGTTGAAC |
| 13908_19 | 20 | [C/T] | GCAAGAGTCTGGGCAAGATCCTGCAACAAGGTAAAT |
| 13912_19 | 20 | [G/A] | TTGGCAAATGCAGCAAAATGATGCTTGTTTGTTGGA |
| 13916_4 | 5 | [C/T] | CCTGCGAGGCAAGCAGGCGCATGCCGTGCTGCAGCT |
| 13917_33 | 34 | [G/A] | ATTTCTGAGACAGCAGCTTGCTGCTATTGCCACGCA |
| 13922_28 | 29 | [G/A] | ACGGGGGTTCTGGCACGCTGCTGCTCATGAAGGATT |
| 13927_18 | 19 | [T/C] | TGTCCAGTTTGAGCAACGTTGTGCCCTAAATAGCTG |
| 13929_10 | 11 | [G/T] | GCCGTCGTCCGAGCACCTGCCTGCGAGCGGAGACAG |
| 13935_8 | 9 | [G/A] | GATTGAGGAGCGGCATGGTGGTGCTGCAGGTAGCGT |
| 13937_33 | 34 | [A/G] | TCTCTGCCGATGGCACACAGGTGCTCGGGGGGGAAA |
| 13940_28 | 29 | [G/A] | TGAACCTCGTTAGCACACATTTGCATGCGCGCTGGA |
| 13942_9 | 10 | [G/A] | CTTCACCTTGAAGCACGCAAGTGCTTTGGCACAGAA |
| 13945_1 | 2 | [T/G] | TTATGACAGGTCGCAAAGTCATGCACTCGATGACCC |
| 13960_1 | 2 | [C/A] | GCACTACCTGCGGCACCACCGTGCCTCAAAGTAAAA |
| 13963_7 | 8 | [A/G] | GAAGCAGACGCTGCATATCAATGCGTTTGTTGCGTG |
| 13965_29 | 30 | [A/T] | AAATTCCACACAGCATTTCCCTGCAGCACAGAAACA |
| 13966_3 | 4 | [G/A] | AATGAACAGTAAGCAGAATGTTGCTGCCTCAGAACT |
| 13970_28 | 29 | [A/T] | CTTTACACGATTGCATTCGAGTGCACATAAACGGGT |
| 13972_16 | 17 | [G/A] | TCAGCTCTCGGTGCAGGAGGCTGCTGTGAACTGCCA |
| 13979_19 | 20 | [G/A] | ACACACATGCATGCATTTGGGTGCATTTGTCTGTAT |
| 13982_29 | 30 | [A/G] | TGCAGGGCCTATGCATACAGGTGCTGCCAGTCAGCT |
| 13991_18 | 19 | [C/T] | GGATCTCATGATGCACATCCATGCCGACGTGTTCAC |
| 13992_33 | 34 | [G/A] | CAAACCTGGCATGCAGACCTCTGCTAGCAGCTTGAA |
| 13998_26 | 27 | [G/A] | GGGCATCTCTCAGCACACAGATGCACGCAACTAGCA |
| 140_15 | 16 | [C/T] | GTTGTCAGCGATGCACGAGGCTGCTTATGAAACTCA |
| 14006_18 | 19 | [G/T] | TTTAGTCGAGTTGCACTTGATTGCATGTTTGCATGT |
| 14010_1 | 2 | [G/C] | CGGATCTGTCCAGCAAAGATGTGCAGAGCGAATCAG |
| 14013_17 | 18 | [G/A] | TCAGTTTCGCACGCACGGGGTTGCAACCTGGAGTTT |
| 14015_16 | 17 | [C/A] | AGGGTTTGATCAGCAACCATCTGCAGTCAGCCAGAA |
| 1402_20 | 21 | [T/C] | TAACCTTTTTGGGCACCATTTTGCTGAACTGTAACC |
| 14022_30 | 31 | [C/T] | TGGACTAAAAACGCATCCAAATGCAGCAAACTGTAT |
| 14031_33 | 34 | [G/A] | GTGTTTAGAGAGGCATCTGTCTGCGCTTGAAGCGAA |
| 14032_24 | 25 | [G/T] | GGAGGTGGATGTGCAGTTGGGTGCGGCTGAGTCCGG |
| 14033_33 | 34 | [A/T] | TCTGAGCTGTAGGCATCTGGATGCCAGCTGAAATAG |
| 14034_25 | 26 | [C/A] | ATATATGCACTGGCAGATTCGTGCCCAATACTGCAC |
| 14047_4 | 5 | [G/A] | CTTGGAATGAACGCAGCATGATGCGTTTCAAAGAGC |
| 14050_7 | 8 | [G/A] | ACACGGAGCCCAGCAGCCCCATGCGCCTTCTGCGAA |
| 14051_8 | 9 | [C/A] | TCTTTGGACCTTGCAGTATCCTGCACATTGTCACGT |
| 14055_2 | 3 | [C/G] | TACAACTGCAGGGCAACGAAATGCAGTAAGCGTCTA |
| 14058_29 | 30 | [G/A] | CAGTGTGGACCTGCAGAGACCTGCTCACAGCCCCTA |
| 14062_29 | 30 | [A/G] | AGTATGGTGGAGGCAGTGTTATGCTGTGGAGTGTTA |
| 14065_6 | 7 | [G/A] | CTCCATGGAGCAGCAGATCACTGCGTCTTCCTTCAC |
| 14066_16 | 17 | [C/T] | ACTTTCCACAGAGCATCGAGTTGCCCTTGCCCTTCT |
| 14067_28 | 29 | [A/G] | ACTCTTCTGTATGCATCAGCCTGCACGCAAACGAGC |
| 14070_8 | 9 | [C/A] | GCACACTGCATGGCATCAAACTGCATGGCATCAAAT |
| 14072_34 | 35 | [C/T] | TGCAGCACTGCAGCAGCTGCATGCGTGTATTGTTCA |
| 14073_2 | 3 | [A/C] | GACACTGGCCCGGCACTACACTGCTGCAATGGAATC |
| 14076_5 | 6 | [A/G] | ACTGAGCATTTAGCAACCTCCTGCCCTGGGACTCTT |
| 14077_1 | 2 | [A/G] | CAACATTGAGGGGCAGACTGATGCCAAGACCTCAAG |
| 14081_4 | 5 | [C/G] | CCGGGTTTGCCTGCAGTGTTTTGCATGATACAGCGC |
| 14084_25 | 26 | [C/T] | ACATGCGTGCACGCACAGAGATGCACGTGCAAACAC |
| 14086_15 | 16 | [C/T] | GCACAACCTGCTGCACCACCGTGCCAGCCAGTAGTT |
| 14090_26 | 27 | [A/G] | GGGTCTTGGCATGCACTTGAGTGCGCACACACACAC |
| 141_1 | 2 | [C/T] | GCCAGTGTGCAAGCATGTATGTGCTTGTGTGTGTTT |
| 14103_1 | 2 | [A/G] | CACATAGGTGTGGCAAGGTGCTGCTGCAGGTAGTGT |
| 14105_25 | 26 | [C/T] | ATTGCTGAGCTTGCAAACTTATGCACATCAATTAGC |
| 14108_25 | 26 | [C/T] | AGCCAGCTGTAGGCACCAACCTGCTCGTCACTGCTA |
| 14109_6 | 7 | [C/T] | AATGTCCATGTGGCAGGAGGTTGCATGCAGCATCAC |
| 14115_29 | 30 | [T/A] | ATGGATTGGCATGCAACATTTTGCAATAGTCACAAA |
| 14116_25 | 26 | [G/A] | CTAAATACGTGAGCAGAGGTCTGCAGAGGCAGAACA |
| 14126_7 | 8 | [C/G] | CACTTCCCTGCTGCACTGGACTGCAAGTCTTTACTG |
| 14127_31 | 32 | [G/T] | TAGACTTCCTTCGCATACAATTGCTCACTTCGTCCC |
| 14128_18 | 19 | [C/A] | GCACTGTTGTTTGCACTGCAATGCACACTTGCATCA |
| 14130_17 | 18 | [A/C] | AAAGTGCACAGTGCATAAATTTGCACACAGCAAAAT |
| 1414_19 | 20 | [T/C] | ATCGATCACTGAGCAATTCTGTGCCTTACTCTCATA |
| 14143_33 | 34 | [T/C] | CAGTGCCAGGCTGCAGGCTGCTGCTTCCTCCCGTTT |
| 14144_11 | 12 | [T/C] | ACTGGAGCAGCTGCACTCGCCTGCATGCCTGCAGCG |
| 14147_29 | 30 | [G/A] | TAGCCTTTGCTTGCATTGTCCTGCCCATCGAACGCT |
| 14153_18 | 19 | [C/T] | ATGTTGACGGCTGCATTTCAATGCACTTTTTATCCT |
| 14157_34 | 35 | [C/A] | CAGTAAGGTAATGCATGTGCATGCACAAACACACAC |
| 14160_29 | 30 | [G/A] | CTCTGACCACATGCACAGCATTGCCTAATGTGCAAT |
| 14161_32 | 33 | [C/T] | AAAGGTTTATCTGCAGATTCCTGCACAGGCTCCTGG |
| 14163_19 | 20 | [C/T] | CTGTGTCCGATCGCAAGTCCCTGCAGCGGATAGTAA |
| 14169_7 | 8 | [C/A] | GTATGTGCAGTTGCATTGTATTGCCTTCGATTGCAG |
| 14171_31 | 32 | [G/T] | CTGAAAGCAGAAGCAGAGCTGTGCTGTAAAAGCACT |
| 14181_17 | 18 | [C/T] | ACAGTGGGACTGGCAGCCCTGTGCCAGTGACGGCCA |
| 14184_32 | 33 | [T/C] | GTGCACTGAGCAGCAGATATCTGCTCCAGGGTTTAT |
| 14188_5 | 6 | [G/T] | GAGTTGTTCTTGGCAGCTTAATGCAAGACCTTCATA |
| 14189_2 | 3 | [G/A] | AGGATAAGTTGAGCAGCAGTCTGCTTCGGAAAACCG |
| 14192_30 | 31 | [G/A] | ATGAAGTAGATTGCATTGTGGTGCAGTCTGGCCCAC |
| 14195_25 | 26 | [C/T] | AAGACTGAAGATGCAGCCATCTGCTCCTGCTTATGT |
| 14200_6 | 7 | [T/A] | TCGGCAATTTCAGCAGAGGACTGCAATAGGCATTTT |
| 14203_8 | 9 | [C/G] | AAAGGAGGCTGTGCACACTTATGCAGCAACTGGATT |
| 14204_10 | 11 | [A/G] | AAAGTTTTACACGCACCGCGGTGCTACGCCGCCACA |
| 14205_1 | 2 | [G/A] | AGACATAAACTGGCACTGACCTGCAGAAGGACCTAG |
| 14206_32 | 33 | [C/T] | GAGTGCAGCCGAGCATCCAGCTGCAGTCTCGCCGTG |
| 14209_4 | 5 | [A/C] | CATGACTGATTTGCATACTCATGCCCCGCCCTCTAG |
| 1421_16 | 17 | [G/A] | TCAATCAGGGTGGCACGGAGGTGCCGCAGGATGTGT |
| 14210_4 | 5 | [G/C] | TACTGCACAAGAGCAAATGCATGCTGCCATGCACAG |
| 14215_7 | 8 | [C/T] | GCTATCCCGATGGCAAGGACGTGCTGCGCGAAACCA |
| 14218_8 | 9 | [C/T] | CCATGTAGCAGGGCAATGCAATGCCAAAGCAAGTAG |
| 14222_25 | 26 | [C/G] | AAGCACAAGCCAGCATCAGCATGCACACTATTCACT |
| 14226_3 | 4 | [C/A] | TGCCACAGCAATGCAGCGTAATGCTTAGGATGCAAA |
| 14233_11 | 12 | [G/T] | AGGATGACGTCGGCATTCTTCTGCTCGCTCTCAGCG |
| 14235_17 | 18 | [A/G] | CTCCTCTCTGAAGCAGCAGAATGCACAGATAACCCG |
| 14237_7 | 8 | [T/C] | TTTCAGCTCGGAGCACTGACCTGCCTGTGAGTAACC |
| 1424_16 | 17 | [G/A] | CCTCCGAGATTTGCACGGCAGTGCTGGTGGAAATAC |
| 14242_32 | 33 | [G/A] | GTTTCCATGCATGCATGCGCGTGCTAGTGCTCGGTA |
| 14244_4 | 5 | [T/A] | GGTGTTGGGGAAGCAGCCAGCTGCTTTATACACCAG |
| 14252_30 | 31 | [G/A] | TCTACCTGGTGTGCAGACTTTTGCCAGGAAGCTTCA |
| 14260_15 | 16 | [T/A] | TTATGGCTGATCGCATACCGATGCCGTATATCATGT |
| 14262_9 | 10 | [C/G] | CTGCAAACTCGAGCATCTGGATGCCATCAGCGGTGA |
| 14267_17 | 18 | [T/A] | CCAGCCCGACGGGCAAGTCGGTGCCGTGCGTGGTGG |
| 14269_8 | 9 | [G/A] | TCGTGATGGATGGCATCATCATGCCAACCAAACAGG |
| 14274_10 | 11 | [G/A] | TGGACCAGTTGAGCATGAGGATGCTTTCCTCCTGCT |
| 14276_5 | 6 | [G/A] | TTGGAGAAAGAAGCATCACTCTGCCAGATCATGATA |
| 14283_17 | 18 | [G/T] | TCCAGCCTGCCTGCAAAGCTCTGCCACCGAGCACCT |
| 14284_1 | 2 | [C/T] | ACGGGCGCAGGCGCAGGTCGGTGCCCGAGCATCAGA |
| 14285_33 | 34 | [C/T] | TGTGTACAGACAGCACCTCTCTGCAGGAGAACACAA |
| 14293_28 | 29 | [G/A] | GTCGCCCGCTCAGCATCTCGGTGCACTGGAAGCCAG |
| 14294_20 | 21 | [A/G] | CAGGCAGCAGCAGCAGAAAAATGCCATCCGTCAGGA |
| 14297_29 | 30 | [C/T] | CGAACAGAGGGGGCATCATGATGCCCGGCCTGACAC |
| 14303_26 | 27 | [A/G] | GAAGATAAAATGGCACTGCTTTGCCCAGCTCTGTGG |
| 14306_17 | 18 | [G/A] | AGACCGCCGCGTGCATCGCACTGCAGCCTTTGTGAT |
| 1431_2 | 3 | [T/C] | CTTCCTGTGAGCGCAGATGTTTGCATTACACACAGC |
| 14310_11 | 12 | [C/T] | CCCGGGGCCAGCGCAGCTACATGCTACACAGCTAGC |
| 14312_18 | 19 | [C/A] | TGTGGTTTCTCAGCATGACGTTGCTCTTTGGCTCTT |
| 14316_33 | 34 | [C/T] | GGAAGAAGTACAGCACAGATCTGCACGATGCATCAG |
| 14317_3 | 4 | [C/T] | ACACGCACACACGCATGCACATGCTCACACACACTG |
| 14319_2 | 3 | [A/G] | AGAGGAACCAAAGCACCACAATGCCCCACTATACAA |
| 14321_25 | 26 | [C/T] | CACAGGGCAGGCGCAGGGTTGTGCGCTACACTAAAC |
| 14329_31 | 32 | [T/G] | GCGACTGCACAAGCACCTGAATGCCCTTCATTGTTT |
| 14332_4 | 5 | [G/C] | AGCTGTATGCTAGCACTTAGCTGCTATCCAGTCCAC |
| 14333_28 | 29 | [G/A] | TGCAACCAGGCTGCAGCGTGTTGCATATGAACATCA |
| 14342_29 | 30 | [C/T] | CCACCACCGATGGCAATCACCTGCGCATTCTGGAGC |
| 14343_8 | 9 | [C/T] | TTGGCCTCCAGAGCACTGTACTGCATGTTAAAAAGG |
| 14344_8 | 9 | [C/T] | GAAGATAACGGTGCAATGTGATGCAGCATGGTTAAA |
| 14345_30 | 31 | [T/C] | ACTCTGCGTGACGCATTCATGTGCTCTGTGTGACAA |
| 14351_19 | 20 | [G/T] | GATGCCCCCACAGCATGGCGCTGCCACCACCGTGCT |
| 14357_25 | 26 | [T/C] | CTGCAAGAACAGGCAGTTCCCTGCTTGCGTTTTGAT |
| 14358_11 | 12 | [T/G] | CTGGGTGCTGTTGCATTGGGCTGCTCTCAGGAAGTA |
| 14360_1 | 2 | [T/A] | CTTGTGCCCTGGGCAGCGGCATGCCTCTAGGAAAAG |
| 14363_5 | 6 | [G/A] | CCTTTGTACTTAGCAGGAGGCTGCCATTACTGTCCA |
| 14367_30 | 31 | [A/T] | AGACCTACATTAGCACTCGGTTGCTATGCTAACACG |
| 14375_8 | 9 | [C/T] | TACACTTTCAGAGCACACGTGTGCCACCGGAGGCAA |
| 14382_34 | 35 | [C/G] | CGAACACAATGGGCACTGTTGTGCACTGTCCCAGCG |
| 14386_3 | 4 | [T/G] | CCATCTTGGGTGGCATTGTGGTGCCAGAGGTGGTGT |
| 14391_4 | 5 | [G/A] | TTACGCATCCCTGCAGCTGGTTGCTGAATAGGCAGC |
| 14401_28 | 29 | [G/A] | TGTGATTGTGTTGCACTTGGGTGCATGTGTGCATGC |
| 14404_32 | 33 | [G/A] | ACACAGCCTAGGGCATGTTGGTGCCACCGGGAGAAT |
| 14408_9 | 10 | [C/T] | CAACGTCCCCGCGCAAACAGATGCGTTTTTCCAACA |
| 1441_30 | 31 | [A/T] | TCTATTAAAGCAGCAGTCTGATGCAGAAAGAGGCAT |
| 14415_20 | 21 | [T/G] | CTAGAGCCGCCAGCATGGTGTTGCGCACAAATATCT |
| 14422_28 | 29 | [G/A] | CAAAGTTTGTTTGCATAGTGGTGCAGTAGGCAGTGC |
| 14424_2 | 3 | [C/T] | TTCCCCCACACTGCAGTCTCTTGCTAAACTTGTGAA |
| 14425_31 | 32 | [G/A] | TGCTCACCTCAGGCAGGCCAGTGCCACTGACGCACT |
| 14426_16 | 17 | [G/A] | GAGAGAGAGACAGCAAGAAGATGCAATCAACAGGGA |
| 14428_18 | 19 | [C/T] | CTCCATTCTTATGCAGATCGCTGCGCCGTTAAAAGA |
| 14431_32 | 33 | [C/A] | TGAATTATACATGCATCCGTCTGCAGATCCGTCTCA |
| 14434_10 | 11 | [T/C] | ATTCTCTCTTTAGCATTGATCTGCCTCCGACACACA |
| 14435_5 | 6 | [A/C] | TTTTCAAGCGCAGCAAATTTTTGCACGTGTAGCTTT |
| 14439_24 | 25 | [G/A] | CAGGGGAAAATGGCACACGGTTGCGTAACTTTAAAA |
| 14448_31 | 32 | [C/T] | AGACCTGGACTAGCACGCTCTTGCCCTCTACCGGGC |
| 1445_29 | 30 | [G/A] | AATCAATCAGTGGCAAAAAGTTGCAGTAGGCATGCC |
| 14454_16 | 17 | [C/T] | CTTCTCATTTGAGCACCGTGGTGCGTAAAACGACAC |
| 14465_32 | 33 | [T/A] | TTGGGCATCCTGGCAGTCAGTTGCTTCACTCCTCAT |
| 14466_31 | 32 | [G/A] | TGTTTTCTGTTTGCACCATGCTGCAGCAGCAGATAA |
| 1447_27 | 28 | [C/T] | GTAAAATGCTACGCACTGGCTTGCTCATCTGGTCAG |
| 14472_2 | 3 | [G/A] | GCGCACACATGCGCAGAAACATGCATACACACTTCC |
| 1448_34 | 35 | [G/A] | ACATTACATGCAGCACCACCTTGCTGTCTGCATCGG |
| 14480_32 | 33 | [G/A] | ATGCTGTTGTCAGCAGGTTGCTGCTAACAACAGTGC |
| 14482_11 | 12 | [C/T] | GCCCTTCCTGTCGCAAAACTCTGCGCTCATACACAC |
| 14487_33 | 34 | [C/T] | CTGCGTGAAATCGCACACTAATGCTCATGAGTGCGC |
| 14489_6 | 7 | [G/A] | GACTACGGTGTGGCATTTTGGTGCCACTTGCCAACA |
| 1449_7 | 8 | [A/G] | TGATGTGAACTGGCAAAGTGTTGCTCATCTAGTCAA |
| 14494_11 | 12 | [G/A] | GTTCAATGCTGGGCACAGAGCTGCATATGTAAGCAG |
| 14495_1 | 2 | [A/G] | GACACAATACGGGCAATTTTCTGCAGACACGTTCAA |
| 14497_8 | 9 | [G/A] | TGTAATTGGGTGGCATCCTGGTGCAGTGGGTAGATT |
| 14501_4 | 5 | [G/A] | GAGCGCACATACGCACCCGGCTGCTTTGTGTTATTA |
| 14502_34 | 35 | [G/A] | TCCACCGTTTTCGCAGCGCTGTGCATGCACAGAGGA |
| 14506_10 | 11 | [G/A] | ATTGGGCAGAGAGCACTGCAATGCAGCAGCAACACT |
| 14510_5 | 6 | [G/C] | AAGTCCTAGAGTGCACTTGGGTGCCCAATTTTTCCT |
| 14515_28 | 29 | [A/G] | AGTACGAGCTGTGCAGGCTGCTGCAGCTACTGGCTG |
| 14520_26 | 27 | [C/T] | TAGGGCTTTGAAGCACAAAGGTGCCACCTACAGCTG |
| 14524_1 | 2 | [C/T] | TCAGTTTTCCCAGCAGAACATTGCCCAAAGCATTAC |
| 14526_33 | 34 | [A/T] | TAGTGTCCCAGAGCATCGTCCTGCTCTACAGGTATT |
| 14529_4 | 5 | [G/A] | TGATGAACTATGGCACGACTGTGCCGCCGAAAAACA |
| 14530_29 | 30 | [T/G] | GCATGCATGCCTGCACAGAACTGCAGGATTGCTGCA |
| 14532_33 | 34 | [T/C] | GCTGGTGCATGTGCAGGTGTGTGCGGTAAATCTTCC |
| 14534_18 | 19 | [C/T] | GAAGAAGATCCAGCACTCCAATGCCGTCACTCTCCG |
| 14540_31 | 32 | [T/C] | AGTACTTCAGCAGCAAAAGCCTGCGCTCCAGTGAGA |
| 14544_24 | 25 | [G/A] | GAAACTCAATTTGCAGGAGGCTGCGCCATAGACCTT |
| 14546_1 | 2 | [C/A] | CCCCGTTGACCTGCATCTATATGCTTCACGGACCCG |
| 14548_16 | 17 | [T/A] | CACATGGCCTGAGCATTTTGTTGCAATTGATGCAAT |
| 14552_8 | 9 | [G/A] | CCAGATGGGATGGCATGCCATTGCAGAACACTGTGG |
| 14558_16 | 17 | [G/A] | ACGCAAACAGCAGCAGGTAACTGCGCAGCATCCTCT |
| 14560_34 | 35 | [G/A] | TATTTTGGGCATGCATAATGATGCTGGTTGTCACGA |
| 14568_31 | 32 | [G/T] | GAAAGTTGATGAGCAAGCTTATGCTGTGTGTGTGTG |
| 14569_30 | 31 | [C/T] | GAGAGTGATGGAGCACCAGCATGCCGTCATCCAACA |
| 14571_6 | 7 | [G/T] | CTATGAGCAGTAGCAGAAAGTTGCTCACTCAGTTTG |
| 14578_20 | 21 | [G/A] | TTTCTTGGCCAGGCAGCCCAATGCTGGATAGGCCAT |
| 14589_31 | 32 | [T/C] | TTTATGAATGCTGCAATCTCCTGCTGAGCTCTTGAG |
| 14592_1 | 2 | [A/G] | GAAGTTATCGCTGCACATCGCTGCCTCCAAACACAT |
| 14596_3 | 4 | [G/A] | ACAGTCTCTTCTGCAAACTACTGCTGTCGTGCAGTT |
| 14602_25 | 26 | [C/T] | GTGAATCCAAGGGCAGTCATTTGCTCCCTCAAATAT |
| 14605_19 | 20 | [C/T] | CAGGTCTTCTTAGCAGAGGCATGCGACTGTTACAAC |
| 14607_27 | 28 | [C/T] | TTTCTTTCCCATGCAGACAAGTGCACTCGGTCACGT |
| 14608_10 | 11 | [C/T] | ACACAAAGCCCGGCAGTAGTGTGCAGTAGCTTCCTC |
| 14609_30 | 31 | [T/C] | GGTCCCTGAGCTGCAGGAGTATGCACCCAATGTTCC |
| 14611_31 | 32 | [A/T] | ACATGCCACCATGCACACACATGCAAACTCAATCAA |
| 14613_9 | 10 | [C/T] | GTCACAGTCCGTGCACATCTATGCTGTACGATTGGT |
| 14624_33 | 34 | [C/T] | TGGCAAGGCGATGCATATTGCTGCTATGGAAATCCA |
| 14632_3 | 4 | [G/A] | CAGGAAGAATCGGCACGCATATGCACAGGTAGTGCA |
| 14635_6 | 7 | [G/A] | CAGATAGCCCAGGCATCAGGCTGCAAATCAGGTCTT |
| 14636_7 | 8 | [G/T] | CAATTTGGGGTGGCATGGTGGTGCTGCAGGTAGTGT |
| 14643_19 | 20 | [C/G] | GAGGGACAGGTAGCATGCCCCTGCAACTGACCCAGA |
| 14651_28 | 29 | [C/T] | ACTGGCTGGCTGGCACCACGATGCGCAGCGTGACAG |
| 14652_5 | 6 | [C/A] | CTGAACATTTCGGCATTTTCTTGCCACAGCAGCTAG |
| 14653_6 | 7 | [A/G] | TGCGGCACTGTGGCAGCGTCCTGCTGAAATCTGCTG |
| 14656_16 | 17 | [C/T] | TCCTGGTGCTGTGCATCCTGGTGCTGATCTACGCCA |
| 14658_3 | 4 | [C/T] | CTGCTTAGCTAAGCAGTCATGTGCCAAATACCACAC |
| 14661_25 | 26 | [G/C] | GTAAAGAGATGCGCAGGCAGATGCTGGCTGGCGCAC |
| 14665_33 | 34 | [A/T] | CAGCGTCACTGAGCACTATAGTGCAATGTACACAGA |
| 14666_33 | 34 | [C/T] | TAAATGACGCTGGCATTTCCATGCTCCACTTTACTC |
| 14671_9 | 10 | [A/T] | CAACCACCCAGCGCAACAGGCTGCCCCACACCGTAC |
| 14673_2 | 3 | [G/A] | ACGCATTAGCAGGCATTAACATGCGCCGTGGGTGAT |
| 14674_29 | 30 | [T/C] | AAAACGTCAAGTGCACCGGAGTGCATCAATGTATCC |
| 14676_20 | 21 | [T/C] | GCTTAACCACAAGCACATGGTTGCCTGGCATACCTA |
| 14680_16 | 17 | [C/T] | AAGAGCTCTTACGCAGCACCATGCGCATTGAGGAGG |
| 14682_32 | 33 | [A/C] | GCTGGGACCAGGGCACAGGGGTGCTTGCCTTAAATT |
| 14684_6 | 7 | [C/G] | ATTTAGCCGAATGCACGACAATGCAATACGATCCAA |
| 14693_25 | 26 | [C/T] | AGCCACAGCTCTGCATCCTGCTGCTCCCTGATCCAT |
| 14695_16 | 17 | [G/A] | GCAGTAGATGGAGCAGAAGTGTGCTGGAGGGAGACC |
| 14696_4 | 5 | [T/A] | TATCTGTGAGCGGCATAGATGTGCCGCAGGTAGTGT |
| 14700_34 | 35 | [G/A] | AAAAACTACAGAGCATTGAACTGCAGCAGCAGCAGC |
| 14704_8 | 9 | [G/A] | GCGTTTATGAGGGCAGCCACATGCTTGTGGTTTAAA |
| 14705_7 | 8 | [G/T] | AAAAAGCTACTTGCAGCTGGCTGCTTCAGCGACGGT |
| 14708_18 | 19 | [G/T] | GTCGTTTCATCAGCAGCCTTATGCTTCCCGGTCTGC |
| 14710_2 | 3 | [T/C] | TGTGGGCATGATGCATCAGTCTGCTGATATCAGCCA |
| 14711_5 | 6 | [C/T] | AAAAACGACACCGCAGAATAGTGCTAGCCGGTGCAG |
| 14719_29 | 30 | [T/G] | ACTGCCAAGTCAGCATTCTGTTGCCCATCTTTAGCA |
| 1472_18 | 19 | [T/G] | ATTAGCCCAATAGCAGGGTGGTGCATATGATGTCAC |
| 14720_18 | 19 | [G/A] | ACAGCTCTCCACGCAGGCGGCTGCGAATCCGCTCGC |
| 14725_15 | 16 | [G/C] | CTGCATTATCTGGCAGCTCTGTGCAGATCCAGGAAA |
| 14726_8 | 9 | [G/A] | AAATCGTCGACAGCAAAACGGTGCGGTGCGAAAAAC |
| 14731_33 | 34 | [C/T] | TTGACCTTTAGGGCAGGGCTCTGCCCTGACCTCCGA |
| 14735_28 | 29 | [C/T] | GAGCCCTCAGTGGCAGCCCGGTGCTGTTCCTGATGC |
| 14738_18 | 19 | [T/C] | ACTCTGAACTGAGCAGCTTTATGCATTGATTCGTTT |
| 14740_6 | 7 | [A/G] | CACTGCATAGTGGCAAACAGATGCAGGTGGTGTTAA |
| 14742_31 | 32 | [A/G] | GAGAACGGAACTGCACCGCAGTGCACGCCTCAATCC |
| 14749_18 | 19 | [T/G] | ACCATGTCCGAGGCATATTCCTGCCCTGTGCCTGAA |
| 14750_3 | 4 | [C/G] | TGTCGTCATCGGGCACCTTTTTGCGGTTAGTTGCCG |
| 14751_10 | 11 | [A/T] | GGTGGATGCCAAGCAGTACAGTGCATAGTAACTGGA |
| 14756_3 | 4 | [A/G] | CAAAGAACCTTGGCACAAAACTGCCAGCATGTGATA |
| 14758_28 | 29 | [C/T] | TTTGGTACTGCTGCACCACCATGCCACCCCATATCA |
| 14763_7 | 8 | [C/T] | AGAGCCTTAGCAGCATGGGTCTGCCTGCCAAGGTGA |
| 14765_4 | 5 | [G/T] | TAGCTCTGAATGGCAAACCGTTGCTTATGCCTCTCA |
| 14766_1 | 2 | [G/T] | TGGTGCAATAAAGCAGAAGGTTGCCTCTGCGCTCTT |
| 14771_32 | 33 | [G/C] | GAATGAAGGGCAGCATGGTGGTGCCACAGGTAGTAT |
| 14773_1 | 2 | [T/C] | TTAACAACAAAAGCATGGCGGTGCTTTTTTCTCCAG |
| 14781_33 | 34 | [C/T] | TCAGACAGAACAGCACAGCCATGCCAGCTGTGATAA |
| 14783_6 | 7 | [G/A] | ATTGTGGCCTGTGCAGCTTAATGCTGTTTAAAGGCT |
| 14785_24 | 25 | [A/G] | TATGACAACATGGCACACTTGTGCGAATTCAGCAAC |
| 14787_7 | 8 | [T/G] | AAAGTGTTTTGTGCATAGGTTTGCCACCTAGGTCTG |
| 14788_20 | 21 | [C/G] | AACAGCCCCAAAGCATAATGCTGCTGCCACCATGCT |
| 14789_16 | 17 | [C/T] | AAGTGTCCAATGGCATCGAGGTGCAGGCTGCTTGAT |
| 14790_27 | 28 | [T/G] | ATGCAAATCTCAGCACATCGATGCTTTGGAATTAGC |
| 1480_8 | 9 | [C/T] | GAGCAGCTCCAGGCAGAGCAGTGCCTGAAGGAGAAC |
| 14801_3 | 4 | [C/T] | CGCCGGGACTCTGCACGTCGATGCCGTGACTCTCCA |
| 14802_8 | 9 | [G/A] | TGGACGGCGCAAGCAAAGATCTGCACTGGTTGGGGG |
| 14805_11 | 12 | [T/C] | GGCAGAGGACGTGCAAATAAGTGCGAACACACTGAA |
| 14806_32 | 33 | [C/A] | GTTAATTACCTGGCAAACTTGTGCTGGCTGAGCCCC |
| 14807_24 | 25 | [G/A] | ACGCACTAAACCGCATTCAAATGCGCCATTTCCAAA |
| 14809_31 | 32 | [T/C] | TCTATAAGCACTGCATGCATGTGCTCTCCGCTCCAT |
| 14810_7 | 8 | [G/A] | GAGGAGAGAAGGGCAGCAAAATGCAATGAGGCATAA |
| 14811_16 | 17 | [A/G] | TACGCTTACTTCGCAGATTTGTGCTCATTCAGCCAC |
| 14812_6 | 7 | [C/T] | AGCACTCTGTGTGCACTACAGTGCCAAATGCTTTGC |
| 14817_34 | 35 | [T/G] | AAAAGGAACTCCGCAGGATCTTGCCAGCTATTCGGG |
| 1482_16 | 17 | [C/T] | ACATGCAAATTAGCAGCGATTTGCATACGCTGCCTG |
| 14820_24 | 25 | [C/T] | CTGTACCTGCGTGCATGCCCATGCCCATGCCCGTAC |
| 14823_18 | 19 | [C/T] | CCATGAAGTAGCGCAACATCCTGCAGTGTGCGTTCG |
| 14833_24 | 25 | [A/G] | TGTTGTGACATTGCAGAAGCTTGCGGAAACAATTCC |
| 14834_20 | 21 | [T/G] | CTCATCGTGAGGGCATAAGCTTGCTCAGGCCCAGAA |
| 1484_31 | 32 | [A/G] | TCAAGTCCTACAGCAGCCGACTGCTGAAAGTACAGG |
| 14842_5 | 6 | [T/C] | ACTTTTCAATGGGCATCACGATGCCCCTTAAAGCCA |
| 14845_33 | 34 | [T/C] | GTTCACTGGGTGGCATGGTGGTGCTGCAGGTAGTGC |
| 14849_33 | 34 | [C/T] | GCAGAATGGCTAGCAGGAAAATGCTGAGTCACTCGC |
| 14850_9 | 10 | [A/C] | GAGGAACAGAGAGCAGGATTCTGCGAATCAAAGTTA |
| 14851_24 | 25 | [C/T] | TGCCACGAACCCGCAAACAGCTGCCGAGCAAAAACT |
| 14854_1 | 2 | [C/A] | CCCCCCACCCACGCATCCTCCTGCCCTCAACAGCCC |
| 1486_25 | 26 | [T/C] | AACAGTGAACCAGCAACAGACTGCATTGCTTGGAGG |
| 14862_6 | 7 | [G/A] | AATTGCGCGTGGGCATTCCGGTGCCTTCGTCGTGCA |
| 14863_15 | 16 | [C/T] | GGATAAACACATGCACAGCCGTGCACACCATGAGGC |
| 14865_11 | 12 | [A/T] | ACACAGCAGCCAGCAGAAATGTGCTCTTATTATCCC |
| 14866_19 | 20 | [C/T] | CGCAAGTGCAGAGCATTCGTGTGCACAAAGCTATGG |
| 14869_1 | 2 | [C/A] | GCACACATACTCGCACAGGTCTGCATGTGCACAAGG |
| 14872_16 | 17 | [C/T] | ACATTGCCTAGAGCATCACTGTGCATCAGCTGGCTT |
| 14876_18 | 19 | [C/T] | CTAGGTGATGAGGCAATGCTTTGCACTTCGACCACC |
| 14881_6 | 7 | [A/G] | AATGGTAATGCTGCAAAATGATGCAATCTGCTCCCC |
| 14883_9 | 10 | [T/C] | CATGTGGTCTTGGCACCCGTGTGCGTGCCTACATTA |
| 14888_15 | 16 | [A/G] | CACACACACACAGCAAAGCTCTGCACTGTATGGAGA |
| 14891_10 | 11 | [C/G] | CGAAATAACGCCGCACGTCACTGCTGATGAGCTCTG |
| 14896_15 | 16 | [T/C] | ACATGACCGCGAGCATTTTCTTGCTTCGTTGAAGTT |
| 14897_8 | 9 | [G/A] | CAATGAATGAATGCAGTGTGATGCTGGTGACAATGT |
| 14898_16 | 17 | [C/T] | TGTCACCACATGGCAGCGGCTTGCCACAGATCTTTC |
| 14900_1 | 2 | [A/C] | TAATTAAACATGGCACACTTCTGCCATTGATGCCAA |
| 14903_9 | 10 | [T/G] | AGGCAGACTTCAGCAGCGGCGTGCTCCAGTGCAGTG |
| 14905_32 | 33 | [A/T] | CCCGACCTCACCGCACATCACTGCAGTGATTTACTT |
| 14906_11 | 12 | [T/C] | GCTCTTTACCGTGCAGGGCTCTGCTGACGGAGAAAG |
| 14907_34 | 35 | [C/G] | TGATCAACGTACGCACGTCGGTGCAGACGGTCACCG |
| 14911_6 | 7 | [A/T] | GTTGCTAAGGTAGCACATTGTTGCGGTTTCACGTCA |
| 14918_34 | 35 | [C/A] | AGGAGACGCGCAGCATTGTGCTGCTCTCACATACCG |
| 14923_32 | 33 | [G/A] | GCACTTCCCAGAGCAATGCTCTGCCTGCCCTTGCAC |
| 14925_1 | 2 | [A/T] | CAGTCGACCACAGCATGTCTGTGCCACAGCTTCTCT |
| 14929_24 | 25 | [G/A] | CAGCATTTTTAGGCAGCATAATGCGTGTTCCCAAGA |
| 14934_10 | 11 | [T/C] | CAGTGTACTGTAGCATCTGTCTGCAGCAGAGTTGTA |
| 14937_29 | 30 | [G/A] | CAATGGAACTATGCAGGCAGCTGCAGAACAGACAAA |
| 14940_33 | 34 | [C/T] | TGTGTGTACGGGGCACCGTAGTGCTGCAGGCAGCGG |
| 14944_24 | 25 | [T/A] | TGCGTTTCCTGGGCAGCTCCATGCTGGATGCCATGC |
| 14945_2 | 3 | [A/T] | TAAACAAGATGTGCACCACCCTGCGTGTTTCCTTAT |
| 1495_8 | 9 | [T/C] | TTTTTATCCAATGCAGGCTGCTGCAGAATGCCAGTG |
| 14955_32 | 33 | [G/A] | TTTAATCCCCGCGCACAATATTGCGCATCAACGTTC |
| 14966_5 | 6 | [T/C] | TTCTTTTAGATAGCACACATGTGCCACCTCACACGC |
| 1497_16 | 17 | [C/A] | ACATCAAACTGTGCATCAGGATGCCAAGTTGTTCTC |
| 14973_31 | 32 | [T/G] | TTAGGATCAACTGCAGTCAACTGCAGCGACTTTAAT |
| 14976_19 | 20 | [G/A] | CGGGGAACCCGGGCACCGCGCTGCCGTAGCCGACAC |
| 14977_17 | 18 | [G/T] | TTACCAGCCCCTGCAGAGTATTGCAGTAAAACAGCT |
| 14985_5 | 6 | [T/G] | GCCCATAGGACAGCACTGTTATGCTGTTGGAAGCCA |
| 14989_26 | 27 | [C/T] | AATCAATGACAGGCATTTAGCTGCACCATGTCCAGC |
| 14992_20 | 21 | [T/A] | AATGGGTTGAGGGCATAATTTTGCTCACTTTCCAAA |
| 14993_19 | 20 | [A/C] | ACAAAGCAAGAAGCAGAGGAGTGCGGAATAAGGAGA |
| 14997_17 | 18 | [A/G] | ATCGTGACTCCGGCATTAGCATGCGTAATGGCAGGG |
| 15_9 | 10 | [T/G] | GAGCTGGAGTTTGCAATCGACTGCACAACACGCTCC |
| 15004_30 | 31 | [G/A] | ATTGCTGGGACAGCAGTCTGCTGCTGAGAGGAGGAA |
| 15007_32 | 33 | [C/T] | GGATTAGCCCATGCAGTTGTCTGCAAGAGCATCTAT |
| 15010_20 | 21 | [G/C] | GCGGCGGGCCTCGCAGCAGAGTGCTGGCCATGGACA |
| 15016_18 | 19 | [A/G] | GACACTACACTAGCATGTAGATGCCAGACAAACCCA |
| 15017_19 | 20 | [G/T] | TCCAGAGGGGAAGCAACAGGCTGCACCATACTGTCC |
| 15018_32 | 33 | [C/T] | CCAGTTTTTCTGGCACATCCGTGCATTCTAAGCCTT |
| 15020_34 | 35 | [T/A] | TGATCAGTACCTGCAGCTCTCTGCCAAACTTCCCTC |
| 15024_34 | 35 | [T/G] | GGTTCCCAGGAGGCATGGCGGTGCCGCAGGTAGTTC |
| 15025_6 | 7 | [G/T] | TATTTGGTTTTTGCACAGCTGTGCATCGGGGCTTGC |
| 15030_19 | 20 | [G/C] | CAGATTCCGGTGGCACGGTGGTGCCACATGTTTTGC |
| 15031_16 | 17 | [C/T] | AATGAATGGTTTGCAGCGTCCTGCTTTTCCAGCAAG |
| 15032_25 | 26 | [C/T] | CAGCCATTTTCAGCAGTCCATTGCACGTTACTCAAA |
| 15035_8 | 9 | [T/C] | ATCTTAAATGACGCAGTACCTTGCTGCCCGTCTGTC |
| 15036_25 | 26 | [C/G] | CTTGGCACCTCTGCAGCTGATTGCTCAACTAGTGAC |
| 1504_25 | 26 | [G/A] | GATTGTGTGGAGGCACAGATCTGCAGAACAAAAACA |
| 15041_5 | 6 | [C/T] | AACAGCTTTAGTGCACTTTAATGCCCCTGCCTTTCT |
| 15042_9 | 10 | [A/G] | GGCCAGCCTAAGGCACACCTGTGCTGTCTAAACAGT |
| 15059_28 | 29 | [G/A] | GCCTCACTCTTGGCAACGTCATGCTGCCGCTCACGC |
| 1506_15 | 16 | [G/A] | TGCCCTAGCTGGGCAGACTTCTGCCTTGTCCAAGTA |
| 15066_1 | 2 | [C/G] | CCTCACTTACACGCACACGACTGCAACAAGAGATGA |
| 15067_26 | 27 | [C/T] | ACTGCTGCACTCGCAGGTTGATGCTTCTTCAGTTGC |
| 15069_11 | 12 | [C/T] | TCCCAGGCTCCCGCAGCGTCCTGCCCACACAAGTGT |
| 15071_28 | 29 | [G/A] | GTCGGTGCATGTGCACGCACGTGCATACGATGTGTG |
| 15073_6 | 7 | [C/T] | TCACATCAGGTTGCATCACACTGCACATTGGTCATT |
| 15076_25 | 26 | [G/A] | ATCCTGAGCTCCGCAGGTTGCTGCCGATCATGCTGT |
| 15079_32 | 33 | [G/A] | TACATGTGAAAGGCATTCATGTGCGAAGTGCTGGAT |
| 15080_4 | 5 | [G/A] | CACCACAAGCTTGCATGCAGCTGCTCGGCCGTGGAA |
| 15082_7 | 8 | [T/C] | AGCGCAGTGCGAGCACAAGGGTGCACTGGCTTCACA |
| 15083_3 | 4 | [G/A] | GCCGTGTCTGCTGCAGTTTTATGCTAATTGCATTCA |
| 15085_29 | 30 | [A/G] | GCATGTGATAGAGCAACACAATGCCGTCCAGGCTCA |
| 15086_2 | 3 | [G/T] | CAGGAGCCTGTTGCACAAACCTGCTGCTTTTGCATT |
| 15087_27 | 28 | [C/T] | GCAGGTGGAGAAGCATGTGCGTGCTCTCAAACAAAC |
| 15090_9 | 10 | [G/A] | AGGTTGAGGGTGGCACAATGGTGCTGCAGGTAATAC |
| 15091_6 | 7 | [C/T] | CAAGCACGGCATGCAGTAAGGTGCGGTGCAGGAGCT |
| 15093_7 | 8 | [G/A] | ACGACACGGAAAGCAAAACCATGCATCAGTTTCTTC |
| 15094_32 | 33 | [C/T] | GATTTGTGGTTAGCAGCGCTATGCAAGCTTTCCATA |
| 15096_15 | 16 | [T/C] | CAAGATTGCTGTGCACGTCCCTGCCTCCCAGATACC |
| 15098_4 | 5 | [T/C] | TTACTACCTGCAGCACCGCTGTGCCACCCAAAGAAA |
| 15102_3 | 4 | [G/A] | ACCGAGACCACTGCAAAGAAATGCTGAGCGGTAAAT |
| 15104_16 | 17 | [A/T] | GAGAGCAGCACAGCACATCCATGCGCTTGCAGAGCA |
| 15110_30 | 31 | [C/A] | TTCACACCCATAGCAGCAGATTGCCTTTAGCGTTAT |
| 15116_4 | 5 | [G/A] | GGGAGAGTGTTAGCAGAGCCGTGCAGTTTACTGTAC |
| 15125_28 | 29 | [C/G] | GTCCTCAGTGGTGCAGGGAGATGCAGGGCCAGGTGA |
| 15134_31 | 32 | [G/A] | GGTATCTGAAAAGCACTTCTTTGCCAGCTGTGCTTT |
| 15135_30 | 31 | [C/T] | CCTTGAATCCGAGCATGTTTTTGCCAGGTGCTCAGG |
| 15148_18 | 19 | [A/G] | CTGGACTATCTCGCACCAAACTGCCCCCATCTCTTT |
| 15150_30 | 31 | [G/A] | CATAGAAGACAAGCAACAAAATGCCAACAGGAGCTA |
| 15153_18 | 19 | [G/A] | GTACTAACAAGCGCAGAAGAGTGCCATCTGTCTCTG |
| 15154_3 | 4 | [T/A] | TGGTGGAGGAAGGCAGTGGTCTGCATACTAAAGTCA |
| 15158_15 | 16 | [G/A] | CTTCACTAGCTTGCAAAAAACTGCTGCAGGCAATGA |
| 15159_24 | 25 | [C/T] | CTTGCTTTCTCTGCACTGCCTTGCCTTACACTGTGA |
| 15162_18 | 19 | [G/A] | AAACAGGAATGTGCATGTGAGTGCGTTTTCCAGAAG |
| 15163_20 | 21 | [G/A] | CTTAATCCTGCTGCACCGCCGTGCTGCCTCATATAC |
| 15164_7 | 8 | [C/T] | AGGTGAACGTGTGCAAAAGTTTGCCAAACTCTACAT |
| 15165_1 | 2 | [C/T] | CTGACCGTTACTGCATTGCCATGCTGCCCAAATCTT |
| 15168_27 | 28 | [G/T] | AACACCTGCCTAGCAGAGCGGTGCTGCGCTGTGTCG |
| 15169_28 | 29 | [C/T] | GTTATGCCAGGGGCATGTTATTGCATGTCGACTTCC |
| 15173_6 | 7 | [A/T] | ATTAAGACTGGTGCACATACCTGCACATAATGGCAC |
| 15175_34 | 35 | [T/A] | ATCAGGCCCAGTGCAGAAATATGCAGGGTATCTCTC |
| 15177_34 | 35 | [T/A] | ACTTGCTTCTGTGCAAATTGCTGCTGCCACATGATT |
| 15178_17 | 18 | [C/T] | TCACTGGAGAAGGCAGACATGTGCATAGAGAACCCG |
| 15183_1 | 2 | [G/A] | CGGGTCTCAGCAGCACAGGCTTGCTTGTACTTTCAG |
| 1519_29 | 30 | [C/T] | GAAAAACAACACGCATCAGCATGCAAAAACTCAAGC |
| 15190_1 | 2 | [G/A] | TGGCCATGATAAGCAGACTCATGCAGCGGGAGCCAA |
| 15193_1 | 2 | [T/C] | TTGTCACTTACCGCAGATGCATGCACAGAAGAAGAC |
| 15195_30 | 31 | [G/A] | GGGGTCGCCATGGCACTTAAATGCAAACGGATTACG |
| 15197_18 | 19 | [G/T] | ATCTTCCCTCTCGCATGGGGATGCCGGCTGGCCATT |
| 15204_7 | 8 | [G/A] | AACCCAAGCTCAGCAATGACCTGCGCAGGGTCAACT |
| 15210_16 | 17 | [T/A] | GAAATGTGCATGGCAGTGTTGTGCTGCCCCCTCGGT |
| 15215_8 | 9 | [G/A] | AAGTGCGTGAAGGCAGGAAGCTGCAGGCAAACACAC |
| 15219_11 | 12 | [A/C] | AGTTCAGAGGTCGCACGCGAGTGCATGCCGCGGTTC |
| 15222_8 | 9 | [C/T] | AGGAAGTCCATTGCAGAGGACTGCAGGCGGAAAGTG |
| 15223_30 | 31 | [C/T] | CCTGGCATGTAGGCACGCACGTGCCTTGTCCTCCCA |
| 15230_34 | 35 | [G/A] | AATAGGCACATGGCAAACTGCTGCTGTTTTCAGTGA |
| 15233_30 | 31 | [T/C] | CCCCCTGATTCAGCAGCAGCCTGCACAGCCTTCCTA |
| 15234_3 | 4 | [A/G] | TCCACCCGAGTGGCACCATTTTGCCTTGAGGATTGT |
| 15236_24 | 25 | [G/A] | TCCCTATTAAAAGCAAAACCATGCGAGCGCCGCTCG |
| 15238_24 | 25 | [G/A] | ATGGTACATGGGGCAAACAAGTGCGCAGCAGATTGT |
| 15241_4 | 5 | [G/A] | CCATGATATCTGGCAGAATTTTGCAGCAGGGACAGT |
| 15243_4 | 5 | [G/T] | CAGAGACAACAGGCAAGGACATGCAAGAGCTTCAGG |
| 15254_33 | 34 | [G/A] | GTATCTTCCTGAGCATTGTGCTGCTGCCACACTGGC |
| 15259_2 | 3 | [G/A] | CCGGTTGTGACAGCAAATCGCTGCAGTGGGCAGTAA |
| 15260_20 | 21 | [G/C] | CAAAGTACAGCTGCAGCTGAGTGCCACCAGTGCTCA |
| 15261_29 | 30 | [G/A] | TCACCGGGAGGCGCAGGCGCGTGCTCGTGGTGCTGA |
| 15262_9 | 10 | [G/T] | AGGGCAGGGGTGGCATGGCAGTGCCACAGGTGTGTT |
| 15269_24 | 25 | [A/T] | TCACTGATAATGGCATTAAAATGCACGCAAAGCCAT |
| 1527_34 | 35 | [G/A] | CTTACCATCTGTGCAAAACCTTGCCTAATGCCTTGC |
| 15275_1 | 2 | [G/A] | TGACAGGACAATGCAACCTGCTGCAACACCATCCTG |
| 15277_4 | 5 | [C/T] | CCCCCTTCCGTCGCACACCCGTGCCACATGAGGCCC |
| 15280_34 | 35 | [G/A] | GTGTGTGTGGAAGCAAGTGTGTGCCCTGGGCCTGGA |
| 15284_32 | 33 | [A/G] | TCGACAACACGCGCAACGGGCTGCGGTGTAGCGGCA |
| 15288_25 | 26 | [G/A] | TGTGCCGTTTAAGCAGTGGCTTGCCGTATCATGTCT |
| 15295_26 | 27 | [A/G] | GTGCTGTGCTAAGCATTGCTCTGCTTAGAAATCACT |
| 15303_17 | 18 | [G/T] | GCCAAGCACATGGCAGTGTAATGCACAGCAAAAATC |
| 15308_15 | 16 | [C/A] | GTTGCATGTTTAGCACTTGCTTGCTTGGCAGACAAC |
| 15309_31 | 32 | [A/G] | GACACTGGAATCGCACTTGGGTGCGGACCAAAGCAA |
| 1531_20 | 21 | [C/T] | GATGTTTGGCTTGCATTAAGCTGCACGGCCATGAAA |
| 15316_17 | 18 | [T/C] | GAGAGTGAGGGAGCACTTGTTTGCACTAAGGCGAGT |
| 15318_15 | 16 | [G/C] | CTACTTGGTGTGGCAGAGCTGTGCTTTGGGTTTAAA |
| 1532_9 | 10 | [C/T] | ATTTGCTTTCAGGCAGTAGTGTGCTGACCACAATTC |
| 15320_17 | 18 | [C/T] | GTGCTGCACTTTGCACTCGTGTGCCATATTCAGACA |
| 15321_18 | 19 | [G/A] | GTCTGATTTACAGCAGCCGTGTGCCATGTTTTCCGT |
| 15322_16 | 17 | [T/A] | CAGACCAGATGGGCAATTGTTTGCTCCATAAGCACC |
| 15327_2 | 3 | [G/A] | CCGGAAGGGCTAGCAATTATGTGCTTGTGTCACACG |
| 1533_3 | 4 | [G/A] | CTGGAGCAATGGGCAGGCCTGTGCACTGCACCTGGG |
| 15335_15 | 16 | [C/T] | TCCACTTATAGGGCACCCGGGTGCAGAATTCTGAAG |
| 15343_3 | 4 | [T/A] | GTTAGTTTTCTAGCACTGGCCTGCAGATCTGAGTAA |
| 15346_2 | 3 | [C/A] | GACGTGTGAAATGCATCATCCTGCGTGTCAGTTCAT |
| 15358_19 | 20 | [C/G] | CCACTAAAGTCAGCAGTTACTTGCCAGTGGGGTATT |
| 15359_17 | 18 | [C/G] | AAGCTTCTCCTTGCAGTCTAATGCAGTCTAAAGCGT |
| 15360_24 | 25 | [A/G] | TGCTCACCTCTAGCAGACGTGTGCAGGTTTTACCCT |
| 15362_4 | 5 | [A/G] | AAGTAATAGCAAGCAATCAAATGCCCCCTGTTCTCC |
| 15365_9 | 10 | [C/T] | ACAGCTTTCCTGGCATAATGTTGCCCATGCTGTGAA |
| 15366_27 | 28 | [T/C] | TGCTTCCCAAGGGCACGATGCTGCCGCTCCCTGTGC |
| 15372_2 | 3 | [G/C] | TGGATTTCTGAAGCAAAAGCATGCTGGACTGAGATA |
| 15381_26 | 27 | [A/G] | GCATGACAGACAGCAACACAATGCTGACAAATCCAC |
| 15383_26 | 27 | [C/T] | CTCTATCGATTGGCAGGAAAATGCTTCAGCTACACA |
| 15388_6 | 7 | [A/G] | GCCGATAGACCAGCAGTCCACTGCTTTACTGTACGG |
| 15396_16 | 17 | [G/T] | ATCACCAAACGTGCATGTTAGTGCCAGGTGTAAACA |
| 15407_11 | 12 | [G/A] | TTCCACCAGACGGCAGGCAGCTGCTCAGACTGTATG |
| 15408_20 | 21 | [A/G] | AACCATGATGCCGCATGTCCGTGCTGTGTGCTAATG |
| 15410_6 | 7 | [G/T] | GGCAAAGGTAGCGCAGATTTTTGCCGCCTGGAAGCT |
| 15414_24 | 25 | [A/G] | TGGGGTCGATCAGCAGGTGAATGCATGGTCACTGTA |
| 15419_20 | 21 | [C/T] | GATTGCTTGTTAGCACTCCTCTGCCACTGCAGGGTG |
| 15421_29 | 30 | [T/G] | TCACGACCTTTAGCACTGCTGTGCTGGATTTGTGTC |
| 15422_15 | 16 | [C/T] | ATGATGAACTGTGCATGGATCTGCCTGAGGGGGAGA |
| 15425_33 | 34 | [G/A] | TCTACATCAGCCGCAGTGTTTTGCTTGAATCTGGAG |
| 15426_7 | 8 | [G/T] | TGCAGCAGGGTTGCAGAGCCATGCACCAGTCTGAGG |
| 15429_7 | 8 | [C/G] | GGTATGTCTGGAGCATGTCAGTGCAGTGAAGCTTTT |
| 15430_24 | 25 | [G/C] | GCTGACCCATGTGCAGGATTCTGCGATGCATTTACA |
| 15431_9 | 10 | [G/A] | GTATGCACCGCCGCATTCACATGCTGTTTAATGGCG |
| 15432_34 | 35 | [C/T] | TTCTGAGATGATGCAGCATGATGCACTGCAGCTTCG |
| 15434_9 | 10 | [A/T] | CATAGAACTACAGCAACACATTGCCCTTGGTGTAAC |
| 15436_18 | 19 | [T/A] | TGTGTTGACTGAGCACAATTGTGCCCAATCCCTATT |
| 15438_11 | 12 | [C/T] | CTATATTCTACCGCACAGTGATGCTAAACTGGCGCT |
| 15439_33 | 34 | [T/C] | GACGCAGCGTCGGCAGATTTGTGCTAACGTTAACGA |
| 1544_16 | 17 | [C/T] | CAAACAAACACTGCACCGTGATGCATTGTGGGATTG |
| 15442_6 | 7 | [T/C] | TTTCCTCGACTTGCAGGGCCGTGCTGAGACCTTTAG |
| 15443_17 | 18 | [G/C] | CAACTTGCACCAGCAGAGCGCTGCAAGAAAGTAGAG |
| 15445_20 | 21 | [C/A] | CAAAATGTCTGTGCACGCCACTGCATCAAGGTATTA |
| 15448_11 | 12 | [C/T] | AGGAGGATGTCCGCACAGAGCTGCCTAGTATTTCAG |
| 15449_30 | 31 | [A/G] | AAGTCCACTGGAGCAGCACAGTGCTGTTGGAGAGCA |
| 15455_16 | 17 | [A/G] | TGTGTGTGTCCTGCATAGTTGTGCGTCTGTGTTTGT |
| 15456_32 | 33 | [T/A] | ACAGTATTGTTGGCAGGCGGTTGCATTTGTATTCTT |
| 15462_29 | 30 | [T/C] | TTCATATGACACGCATGCTTCTGCCTCAGTGCCAGA |
| 15466_2 | 3 | [C/T] | ACCGGAACCCGAGCACGGGAATGCTGCTGAGCAAAC |
| 15479_26 | 27 | [A/C] | ACAGATCGTTCGGCACCTTTTTGCACAGCTTCTTTT |
| 15480_25 | 26 | [A/C] | CAACCTCACTATGCACCACTATGCCACCCTGTGCCC |
| 1549_8 | 9 | [T/C] | ACAACAGATTCAGCAGACAGATGCCTCAAAAATGAT |
| 15491_19 | 20 | [T/C] | GGGCAGCATGTGGCAACCATGTGCACCTTTTGATCC |
| 15496_5 | 6 | [G/A] | TCGCCGTCAAAAGCATCGAGCTGCAGCTAGTACGGG |
| 15497_30 | 31 | [C/A] | GCACCACTTGCAGCACCACCATGCTACCCACTTGTT |
| 15498_4 | 5 | [A/G] | AGATGGCCCCTCGCATCTGGCTGCTTCTAAATCATC |
| 15504_31 | 32 | [C/T] | GGTCAGTTTCGTGCATGTTAGTGCAGAGATGCTATG |
| 15505_2 | 3 | [A/C] | ATATTGAGCTTGGCATCTGGTTGCTTTCAATACCAG |
| 15511_16 | 17 | [G/C] | TGTATTATCATGGCACGTCAATGCTGTGGAAACCTC |
| 15515_20 | 21 | [C/T] | TTCAGAGGTTCAGCAACTTTCTGCCAGAACTGATGA |
| 15516_2 | 3 | [T/G] | ACTTACTGTGCAGCATGATGGTGCAGTTTCTAGCAT |
| 15529_15 | 16 | [A/T] | TATTGCTTAAGGGCAAGTCTTTGCTGTACGATGGAT |
| 15534_30 | 31 | [T/C] | GGCCCCCCCTCTGCACGTGCCTGCTCCAGGCATGTG |
| 15535_19 | 20 | [G/A] | GAGGATGGCATGGCAAACTGCTGCTCCACTGCTATC |
| 1554_16 | 17 | [A/T] | TTTAAGTATTGTGCATTCGAATGCCAGGCAAGGAAA |
| 15541_20 | 21 | [G/C] | GGGAGGTGGGTAGCACCCATGTGCCGATAGAGAGAA |
| 15545_32 | 33 | [C/T] | GTGGGATTGTGAGCACATGCATGCAGGACAATTACA |
| 15549_25 | 26 | [C/T] | AACTGCTGGAATGCATGCAGCTGCTTCAGACTCTTG |
| 1555_28 | 29 | [T/C] | GCCAGCTCCAGGGCACCATTCTGCAGTATGTCAGGA |
| 15551_31 | 32 | [C/G] | TAACACTGCGTCGCATCAGGCTGCATCACATCACCC |
| 15553_1 | 2 | [C/G] | GCCGATTACCCAGCAGGCTACTGCTGCCACTGCCGG |
| 15555_24 | 25 | [G/A] | CTGACAAGAAATGCAATGTGGTGCGTGACCTCTATT |
| 15559_27 | 28 | [C/T] | AAAGTCATAGCAGCATACAGCTGCCAGCGCAAGACT |
| 15568_18 | 19 | [A/G] | AACAGTTCTGGTGCAACCAATTGCCTTCACCAATCA |
| 15571_33 | 34 | [G/A] | AGGAACACACTTGCAGACTTCTGCTCTGGATTAGAG |
| 15574_26 | 27 | [G/A] | CTCTTACCTGCAGCAGCCATGTGCTCGCTTGTCCTT |
| 15576_31 | 32 | [A/G] | GGGTTGAAGTGAGCACATGTGTGCCTGGATCAGTGA |
| 15577_28 | 29 | [C/T] | GTCTCCTTCAATGCAGTGCGCTGCTTTTTATTTTCC |
| 15595_16 | 17 | [A/T] | TCCTTCCAAAATGCAAAGACATGCAGTCAGGCAAAC |
| 15598_9 | 10 | [T/A] | ACATGCTCCTGAGCATCCTGCTGCATCCTGCTCTCA |
| 1560_4 | 5 | [C/G] | AGAGCCAGACAGGCACTGATGTGCAGAGTCAAAGAG |
| 15600_26 | 27 | [C/T] | AAAAAGAAACCTGCATTGATATGCATCGGCCGTTTG |
| 15604_34 | 35 | [A/G] | ACAAAGCCTGCTGCACCAGCCTGCCATCATCAGCAT |
| 15606_15 | 16 | [C/A] | CTGCACGCTTCTGCAAAGCCCTGCAGACACATCGCA |
| 15607_2 | 3 | [G/A] | GGGAACTGTCCAGCACATGCCTGCCTGTATGTATGT |
| 15610_16 | 17 | [A/G] | CCCTCCTGCTGTGCAGATCTGTGCTTATCTCATTAC |
| 15611_10 | 11 | [A/G] | TTCCTTCAGAAAGCATTTGGCTGCCTGACATTGTGC |
| 15616_4 | 5 | [C/A] | CTGCCCCAACAAGCATATAGCTGCCAAGACATGCCC |
| 15619_19 | 20 | [C/A] | ACCATAGTGGTTGCAGAATCATGCTGTGGGGTGTTT |
| 15624_32 | 33 | [C/T] | CTGAACTATCCAGCAGCGACATGCAAAGCCCTCACA |
| 15625_5 | 6 | [C/T] | GTTCTCGTTGCCGCAGCTGACTGCGTTCCTCCGTCA |
| 15632_34 | 35 | [A/C] | TCACACACACCTGCAAGTTCCTGCGTTTTAGATCAT |
| 15633_31 | 32 | [A/G] | ATACGTACATGTGCAACGTATTGCGCAATTGATGTC |
| 15640_25 | 26 | [G/C] | GTGTGTATTACTGCAAACCTCTGCTGGAGTCTGGTA |
| 15642_5 | 6 | [C/T] | CGGCTCAAAGGTGCAGATATATGCACCTGGCACATA |
| 15648_20 | 21 | [G/C] | GTTTTACCGTGTGCATGTTTGTGCTTGTTTTGGGGT |
| 15652_32 | 33 | [G/A] | ATGTAAAGGCTTGCATGCAGCTGCTTAGCCATGAAA |
| 15653_7 | 8 | [A/G] | TCCTCTGATGCTGCATCATGTTGCGTAATCGTAGCG |
| 15654_9 | 10 | [G/A] | GGTTCCATCGATGCAACTTAGTGCTTACGTAGGCTG |
| 15659_33 | 34 | [G/A] | GGGGCACAAATAGCATCACGCTGCAGCAAGGGAGAA |
| 1566_3 | 4 | [A/T] | CAGATGGATGGGGCAAGGTTCTGCTTCTATCTATCT |
| 15664_28 | 29 | [C/T] | TGAAGAACTTTGGCATCTGGCTGCGTTACGACTCCC |
| 15667_20 | 21 | [G/A] | TACATTAGGGTGGCATAGTGGTGCGTGTGGTACATA |
| 15673_30 | 31 | [T/A] | GGGCTCACGTGTGCAGTGTGCTGCCCGAGCTTTTAA |
| 15683_26 | 27 | [A/T] | TCAGCAGGACACGCACGCGGGTGCCAAGGTCAACCG |
| 15686_17 | 18 | [C/T] | GCTTCGACGGCTGCAGTCTTTTGCCTCTTCTGCTCT |
| 15689_5 | 6 | [A/C] | GAGAGAGAGAGAGCAGTCATGTGCATGTGTCCAACG |
| 15692_7 | 8 | [G/A] | ATACTGCGGTTAGCAATGCGATGCCAGCAGGGCTTA |
| 15695_3 | 4 | [T/G] | GTCTGACCGACTGCAGGTACATGCACATCCCGGTCA |
| 15697_9 | 10 | [A/G] | TAGTAGGGCAAAGCACTGGTTTGCTGTTTTGAATTT |
| 15705_6 | 7 | [T/G] | TGTAGGTTTTTGGCAGCCTTCTGCAGCTCCTGTACT |
| 15706_4 | 5 | [C/T] | GTCTCCACTGAGGCATGACTGTGCTGGCTAGATCAG |
| 15707_25 | 26 | [A/G] | TTGTTCACTGGGGCACAGTCATGCTAAAATAGGCAA |
| 15711_19 | 20 | [C/T] | ACGGTTTACTTAGCAGGTGCGTGCGTTGGACACACA |
| 15712_10 | 11 | [A/T] | CTAAATGGCAAAGCAATTAAATGCGTTCTCCCCTCA |
| 15713_15 | 16 | [G/C] | CGTCAGGCAGGAGCAGTGCGATGCCCCACATTAAGA |
| 15715_24 | 25 | [A/C] | CCTTCAGATACAGCAGGATGATGCAGCATCATTCCC |
| 15716_31 | 32 | [C/T] | GGTTAACGCCACGCAGTGCCTTGCCCGTGTTCCCTG |
| 15717_7 | 8 | [G/A] | CATCCTGGAACAGCACCCTTGTGCTTTTACCCTGAA |
| 15721_2 | 3 | [A/G] | ATATTTTGCTCAGCAAGCAGGTGCTGAATGCTCAGA |
| 15725_19 | 20 | [A/T] | TAAATGGCATTTGCACTTCACTGCCAAGTGACCTAG |
| 15729_1 | 2 | [C/G] | ACGAGCAGGAAAGCACCGCCGTGCAGGAGAGACTCC |
| 15733_17 | 18 | [A/C] | TGCATCTCCACAGCATGCTGCTGCCACCCCCATGCT |
| 15740_31 | 32 | [C/T] | TAACCGTAAACCGCAACCTGGTGCTAGGACCCGGAA |
| 15742_24 | 25 | [T/C] | TCAAGCCAGCGTGCACTGTAATGCTTAGTTCTGTCT |
| 15744_27 | 28 | [C/T] | TGTGTAGGTGGTGCACAAGTATGCACTCTCACAAAT |
| 15754_6 | 7 | [G/A] | GTCAGTGATGGAGCAGTCGGGTGCGTGGGTGCAGCT |
| 15758_20 | 21 | [A/T] | TTGAGGAATTTCGCACAGTTATGCACACATTCACAG |
| 15764_32 | 33 | [G/T] | TCACAGTAAACTGCACTTGCTTGCACAACTTTGCTT |
| 15773_27 | 28 | [A/G] | AGATGTGTACCAGCAGGCAGGTGCCAAAGTGCAGCG |
| 15776_27 | 28 | [C/A] | CGGTACATACCAGCAGCATTTTGCCCGCCATCAGGT |
| 15779_11 | 12 | [G/A] | ACACTACCTGCGGCACCACCGTGCCATCCGATGACT |
| 15786_31 | 32 | [G/A] | TGTGCATTAGGTGCAGGGGAGTGCAGTCCAGGTCTA |
| 15791_20 | 21 | [G/A] | GTGAAACATATTGCATGGAAGTGCTGACGCCCTTCA |
| 15795_27 | 28 | [A/G] | CATCAGAGGGAGGCAAAATGCTGCTCAACTCCTCAT |
| 158_28 | 29 | [A/G] | GCTCTAGCCTTTGCAGTCACCTGCTGAAAATGTTAG |
| 15803_5 | 6 | [G/A] | GGAAGGAAGTGAGCACTATGATGCACAGCGGAGAGA |
| 15804_10 | 11 | [C/T] | CAGCCTCTATCAGCAATGGCATGCAAGTCATGACTG |
| 15807_1 | 2 | [G/T] | GTCATGCAAAATGCAGGCGTCTGCAGAAATGTAGTC |
| 1582_33 | 34 | [C/T] | GATTTGTCAGCTGCACACAATTGCTGCAAATCTCCA |
| 15823_19 | 20 | [C/T] | ACTCTAGCTCCGGCATGCTCCTGCGCATGCGCACTA |
| 15834_11 | 12 | [C/T] | ATTTACCCCTGCGCATGATAATGCGGTGCAATAACT |
| 15840_8 | 9 | [C/T] | ACATTCCTTAGTGCACTGATTTGCCACGTTCCTCTT |
| 15849_32 | 33 | [G/A] | CTACTTCTGTATGCATGAGCTTGCAGACACGCGAGA |
| 15850_11 | 12 | [T/C] | TGTGGATGGCTTGCACGGTTTTGCATGTGTTTGGGT |
| 15854_18 | 19 | [A/T] | TAACACACAGTAGCATCTAAATGCGTTCCCAGTGTA |
| 15858_10 | 11 | [C/T] | TTTCAATAGTCAGCAGGGATTTGCTGTCAGGTCTGG |
| 15866_28 | 29 | [A/G] | CTGTTCAGCTCTGCAGATTGCTGCCAAAAAAATGAG |
| 15872_18 | 19 | [A/G] | AGAGTCTGGACAGCATCGACCTGCTGGACTGTAACG |
| 15873_3 | 4 | [G/A] | TGTGAACTCTGTGCAGCCTGATGCAGAAATTTCCTG |
| 15881_1 | 2 | [G/A] | CGATCATCTTCCGCAATCATCTGCTGGTTTGTGTAG |
| 15883_10 | 11 | [C/T] | CTCTGCAGCTCGGCAAGACTCTGCAAATCCGAATGA |
| 15891_27 | 28 | [G/A] | GAGCTTGTCGACGCAGTCTGGTGCCACGCTGTGGAG |
| 15892_27 | 28 | [C/T] | CTGTCACTCACCGCATGAAGCTGCAAACGGATGGAT |
| 15900_32 | 33 | [T/G] | CCACCATCATGTGCACGCCGCTGCGACGCACGGTAA |
| 15901_5 | 6 | [G/A] | CTTTCGAGGCGTGCACACCGCTGCTCCAGCAGCAGC |
| 15902_3 | 4 | [G/C] | ACCGCTCGAGCTGCAGTCGGTTGCGTGGAAGCAGTG |
| 15904_34 | 35 | [A/T] | TAGTTTACATCAGCACACGTGTGCAGAGCAGCGGAG |
| 15905_9 | 10 | [C/T] | ATTCTAAATCAGGCATTAACGTGCACTTGGTCCTTC |
| 15906_17 | 18 | [A/G] | ATACAATGTCCCGCAGTACTGTGCCAGGTTTGTGGT |
| 15908_24 | 25 | [A/G] | ATAATGCTACAAGCATCTTGCTGCACAGTTGGTATG |
| 15912_1 | 2 | [C/T] | GCGACGTAATGAGCAAATGCATGCATATGGGCCACT |
| 15917_11 | 12 | [C/G] | CAGGACTGTGACGCACAGTATTGCATAACAACTGCA |
| 15919_18 | 19 | [T/A] | TATATCTGGATGGCATGGAGATGCCACATGTTGTGT |
| 15920_34 | 35 | [G/A] | CCTCATTCCTAGGCAGCGAGATGCGCCATCAAAGAC |
| 15922_19 | 20 | [A/G] | CATTTGCCAAATGCACCGAAATGCTATGACGTGCAG |
| 15925_3 | 4 | [T/C] | CATCGTGAGATTGCAGAGCCTTGCTTGCAGTTTTGA |
| 15928_34 | 35 | [A/G] | GAGCTATGTGCAGCACCCTAATGCTCATGTAGTTAT |
| 1594_34 | 35 | [T/C] | ACACTACCTGTGGCAACACCATGCCATCAACTCCTT |
| 15947_33 | 34 | [T/G] | TGCCATTCAGAAGCAACTTCCTGCCTCTGGGTCTGC |
| 15948_24 | 25 | [C/T] | TGTGCTTGCTTTGCACAACAATGCCGGCGCTTTTAC |
| 1595_2 | 3 | [G/A] | TTGACCTCAGCTGCAAAAGCATGCAGGAGGAAACAG |
| 15952_16 | 17 | [C/T] | ACGAACTGCCATGCACCTGGCTGCAGCATGCGGTCA |
| 15957_34 | 35 | [T/G] | ACAGCGTTAATGGCAGGCGTTTGCTAAAGAATGGTG |
| 15959_25 | 26 | [G/A] | TTATGCAGGGTGGCATAGTGGTGCCGCAGGTGGTGC |
| 15960_30 | 31 | [G/A] | TGTGTGTGTGTTGCAGATTCCTGCTCTGGGGAAGCA |
| 15966_25 | 26 | [T/C] | GCGCGGCTGAAGGCAGATCAATGCCTATTATTTTTA |
| 15972_7 | 8 | [G/A] | ACTAAAAGACAAGCAGGCAAGTGCGAGTTTAGAGTT |
| 15975_20 | 21 | [T/C] | TTTTTAGTGTTGGCAGCAAGTTGCAACTTAGACTTA |
| 15977_19 | 20 | [C/A] | CATCAGGCAAGGGCAAACTACTGCTCATCCCACAGG |
| 15979_10 | 11 | [C/T] | CCTGTAGTGGCAGCATCCTCTTGCGGTTCACCTCGG |
| 15982_29 | 30 | [G/A] | GCAGACGCAGTCGCATGAATATGCATCGCGCCATAT |
| 1599_24 | 25 | [T/C] | CCATGTTATTCTGCAGAGAGATGCTGATGAGTGCAT |
| 15990_16 | 17 | [C/T] | CTTGTGACTTCAGCAACGTGATGCAACGTTTCATTA |
| 15991_6 | 7 | [G/A] | AAAAAAGAGGAAGCAGGAAGATGCACATCCTGCAGT |
| 15992_4 | 5 | [A/T] | AATGAACAAAGAGCAGCCCACTGCATCTTTCTGACT |
| 15997_32 | 33 | [A/C] | TTGTGTTCGGTGGCATACAGGTGCAGCAAATGAACA |
| 16000_1 | 2 | [G/A] | CGTCTGGCCTCCGCACCACCCTGCCCTTACGCCCAG |
| 16004_8 | 9 | [T/C] | TGGCATGCTAACGCACACAGCTGCTTTCCTGCCTGT |
| 16013_9 | 10 | [G/A] | CCAGGACCAGAGGCAGCAATTTGCCTGTGGTCCATC |
| 16018_10 | 11 | [T/C] | ATTCCAGTAGTTGCACAGCTGTGCGCTAATTCAGGA |
| 16025_15 | 16 | [G/A] | CACTGGGGCATTGCAGAATTATGCCACCTGGGTCAC |
| 16027_24 | 25 | [T/C] | AACCAAACCGAAGCAGCTGTGTGCTAGGTGAGCCCA |
| 1603_16 | 17 | [C/T] | CAAACTCTGCTAGCAGCTAAATGCTGTTACCAACAA |
| 16030_30 | 31 | [T/G] | AACTCTTTTGCTGCAACACTTTGCTGCATCTGCTTC |
| 16031_30 | 31 | [A/G] | ATTTTACCTTCAGCACCACCATGCCACGGTAAAGTA |
| 16036_29 | 30 | [T/C] | GTGATGCTACAAGCACACTGATGCCACCATGCACAC |
| 16038_10 | 11 | [C/G] | GACACTCCCAGAGCATGATGCTGCCACCACTATGCT |
| 16041_15 | 16 | [C/A] | AACAATCACTGGGCACAGTATTGCAACCTCTGTCAT |
| 16046_15 | 16 | [C/T] | CGTGTTGTAATAGCACCGCGCTGCGCGAGCTCGGCG |
| 16055_29 | 30 | [G/A] | TCCAGACAAACAGCACAGGTCTGCTGCAGGGTGAAA |
| 16067_17 | 18 | [A/G] | CCTAATTGGACAGCATGAAAATGCCCACACCTGGAT |
| 16068_16 | 17 | [A/G] | GGCTGTGTGAGTGCACACGCGTGCAAGTGTGTTGTT |
| 16071_4 | 5 | [C/T] | CAGACGGAAAGAGCACAATGCTGCATTATGGTGTGC |
| 16078_11 | 12 | [G/C] | TCTCAACACCGCGCACAACCCTGCCATGGCACCGGG |
| 1608_33 | 34 | [C/T] | AAAGTGTGGTCAGCAGAATTGTGCTGTTTGTGGCAG |
| 16081_28 | 29 | [C/T] | ACCCACAGTGCTGCAGCTACATGCTGAGCCATAGAC |
| 16087_25 | 26 | [G/A] | CACCACCACAAAGCACCGCACTGCTGAATCAGGACC |
| 1609_29 | 30 | [G/A] | ATACTACCCTTGGCACCACTATGCCATCCGATATAC |
| 16091_25 | 26 | [G/A] | ACGATGTGAGCGGCATGTGTCTGCTGAAGTGTAGGA |
| 16097_19 | 20 | [A/G] | TTGAGGACAGTGGCAAGTCAATGCATCTTATTGCTT |
| 16102_17 | 18 | [A/G] | TTGCCATAGTGTGCAGCAATGTGCAGCGGGGTGAAG |
| 16104_4 | 5 | [C/T] | AGGTCAAGCTTGGCATTTTACTGCAAATTTGGAGCG |
| 16107_11 | 12 | [T/C] | CCTCCCGCCATTGCACTAATCTGCGACTCCTTTTCC |
| 16111_5 | 6 | [C/A] | CACATCCTACAAGCATTTCCATGCTTGGGCTGATGG |
| 16113_25 | 26 | [C/T] | TTTCTCCACGTTGCAAAGGATTGCACGATGAAGCTG |
| 16114_8 | 9 | [G/A] | GATGGCAGAATGGCAGGGTACTGCTTTACCGGAAGG |
| 16115_34 | 35 | [A/G] | CTGACTTTGAGGGCATCCCGGTGCATGACATGGGGG |
| 16119_32 | 33 | [T/A] | TAAGTAACGACTGCATGAATGTGCTAACGTGCTAAT |
| 16123_20 | 21 | [A/G] | GAACTGGTTGGGGCACTAAGATGCAGTTAAGTTTGG |
| 16126_16 | 17 | [C/A] | ACGGAGACGCACGCACCTGACTGCCTAGTGGTTCTG |
| 16129_18 | 19 | [C/G] | TTATTGAGTTTGGCAGTGCCTTGCTGATCAAGTGAT |
| 16130_8 | 9 | [G/A] | TGTCCTGTGAAAGCACTTTTGTGCCCTACAGCTCCT |
| 16133_11 | 12 | [G/A] | TCACTCGCACCGGCAGAGAGATGCCAGTTAGACTGT |
| 16136_2 | 3 | [C/T] | TCCATAGCAGCAGCAGCCCTGTGCCTGGGGAGCAGC |
| 16137_31 | 32 | [C/A] | TGCACCAGTCCTGCAGAGGACTGCAGGGACCCAGTG |
| 16138_29 | 30 | [C/T] | GAAAAGGTATTGGCACCCTCGTGCTGCCCCGGTGCA |
| 16145_2 | 3 | [A/C] | TTATTTTTTACTGCACTGTCCTGCAGGTGGCCAGAG |
| 16149_20 | 21 | [C/G] | GATCTATACATTGCAGGGTGCTGCTCTTGCTGGGAG |
| 16154_9 | 10 | [G/A] | CACTTACCTGTGGCATCACATTGCTGCGCATTTTGC |
| 16158_29 | 30 | [C/T] | ATAGCTACACATGCAAGCACATGCCACATCGAGTAC |
| 16161_3 | 4 | [T/A] | TACTCAGACTCAGCAGCACAGTGCTCACCTCACTCT |
| 16167_2 | 3 | [C/T] | GTCTTCGCCATAGCAGACTGCTGCTACTACAACATG |
| 16168_34 | 35 | [G/C] | TCTGTCCAGTAAGCACACTGATGCAGATGTGTCTGT |
| 16170_3 | 4 | [T/C] | AGATGCATAATGGCAGTGTCCTGCTGATAAACTGCA |
| 16174_9 | 10 | [C/T] | CTCTGATCTCTGGCATGTCTATGCATGGACTCCACA |
| 16177_19 | 20 | [C/T] | CAGAACTTTCACGCATGTTCATGCACAGAACTTCTT |
| 16179_3 | 4 | [A/T] | AGAAACTATGCAGCAGCATGTTGCTCTTGTCTCTCA |
| 16180_32 | 33 | [A/C] | TTTGGCTTGAAAGCAGCTGGATGCTGAAAACAAATA |
| 16182_5 | 6 | [C/T] | TTGCACAGTGTGGCACCATTCTGCTCACAGAGCTAA |
| 16188_16 | 17 | [G/T] | GGCTTTAGATGGGCATGACCCTGCCACTGGTTCACT |
| 16192_10 | 11 | [A/G] | TTTAGGCACGAGGCACTGAATTGCACTGCACTTTTA |
| 16199_4 | 5 | [A/G] | GGATAAGACTGGGCATTGACATGCCTCACACATTTA |
| 16205_10 | 11 | [T/C] | TGGAGGAGATTGGCATGCTGCTGCCACAGGTAATGT |
| 16209_4 | 5 | [T/C] | TCATTCCATGTAGCATTGCCATGCTTGCCATTGCCG |
| 16211_9 | 10 | [G/A] | GGCAAGCAGGAGGCATCTGGCTGCAAAGGCCCCACC |
| 16212_16 | 17 | [G/A] | TTACTGGAACTAGCAGGACAATGCTCCAGTGTGCAA |
| 16220_5 | 6 | [G/T] | TTCAATCATGACGCAAGCTACTGCCATACTGAACGA |
| 16223_33 | 34 | [C/T] | CATTGCAGCGTGGCAATTATGTGCAGAATAGTTCAG |
| 16225_29 | 30 | [T/C] | TAGAGAGACACTGCAGCGAATTGCTAATCTGCAGCA |
| 16226_34 | 35 | [G/A] | ATCGGATTTCCAGCACTGTACTGCTTGCTGTGCCGT |
| 16228_4 | 5 | [C/T] | CACTCAAAGGCAGCATCAGTCTGCGGAGTAATTAGT |
| 16229_33 | 34 | [G/T] | AGTTTACCACTGGCACCGACATGCTTCACTGTGGGT |
| 16237_34 | 35 | [T/G] | AAGGCCAACCTGGCATTTATCTGCAGGTTTTATCTA |
| 16240_29 | 30 | [G/A] | TCGAACAAGTGGGCATATGCATGCTTGCCGTGAGGT |
| 16242_31 | 32 | [A/G] | ACTCTGCAGCATGCACCACACTGCCTGGTAGAAACA |
| 16247_26 | 27 | [C/T] | AGACAGAAGTCTGCAATGTCGTGCTTCACCTTTTGT |
| 16248_20 | 21 | [C/T] | TGTCAGGAGAGCGCACTGCGCTGCTTTCTTTTTTCG |
| 16252_33 | 34 | [C/G] | GAGATTCACACTGCAGAGCAGTGCAATGTCTACCGC |
| 16255_24 | 25 | [G/T] | ATTACGGGTAAAGCAGCGTGGTGCGTTTGCCCGGTG |
| 16257_30 | 31 | [C/T] | CTGGTGCTGAGAGCAAGCAGATGCGATAGACAGTTT |
| 16259_30 | 31 | [G/A] | GATAATCGGCAGGCAGGTGGATGCGATGTTATACAA |
| 16262_34 | 35 | [G/A] | CTGGGCTTATTTGCACAAACATGCCCAGTTCCCCGG |
| 16265_33 | 34 | [T/G] | AGAAGGCTGACCGCATATGTGTGCATGTGTGTGTGT |
| 16266_4 | 5 | [C/T] | TGGGCTGTTGGGGCAATAAGGTGCCTTGCTAAAGAG |
| 16268_32 | 33 | [C/T] | CCAGCTAACTCAGCATGCAACTGCTGATGATCCCAG |
| 16273_26 | 27 | [C/T] | TTGCCCTTGGCAGCAGCTTCGTGCAGCGGCGTAAAC |
| 16276_11 | 12 | [T/A] | AAGTTTCCTAATGCAACAGTCTGCCTGTAACCTATC |
| 16277_29 | 30 | [C/T] | GCGGAGACTCTGGCACCCAGGTGCGTGCCCGGGGAC |
| 16278_1 | 2 | [G/A] | CGAACCACTCGAGCAGCGGCATGCTCTCCAAGAGCT |
| 16291_29 | 30 | [G/T] | AATGTGAGCTCAGCACTTACCTGCAGCATGGGGACA |
| 16292_1 | 2 | [A/T] | AATGAAAAGTAAGCACCACACTGCCAGTCTCGGAAA |
| 16297_7 | 8 | [C/T] | ATGTGCTCCACTGCATTCCTGTGCTTACTTTAGATC |
| 16301_25 | 26 | [T/G] | CAATCCAAAGCTGCAGTGCAGTGCCTGAGATTAGAG |
| 16312_8 | 9 | [C/T] | ATATCTCACCCAGCATCACGCTGCAGCAATTAAATG |
| 16316_31 | 32 | [G/C] | TAGCTACCAGAGGCAGGGTGGTGCTCTTCCTGTTTG |
| 16324_4 | 5 | [C/A] | CACAATACCTGGGCATCAGTGTGCTTCCTCATATAC |
| 16325_27 | 28 | [C/T] | CTTGACAGGTCAGCACTGTTTTGCAGGCGTCAGAAG |
| 16329_16 | 17 | [G/A] | TAGCAGAAGTGAGCAGAATGTTGCAAATACACAGCT |
| 16339_10 | 11 | [C/T] | TGGCCTCACTCAGCACTCAACTGCCTACATGACATC |
| 16342_6 | 7 | [C/T] | CTGTTTCGTGCTGCAGAGATCTGCTGGAACTCAGGA |
| 16346_29 | 30 | [C/T] | TCAGCCTCAACAGCAGGTCAGTGCTGATCCCTTTGT |
| 16352_24 | 25 | [A/C] | TTACAAACCCTTGCAGCTTGGTGCATAATAGGGCAA |
| 16355_30 | 31 | [C/T] | GCAGTCCCATCAGCACTCACCTGCAGGTAACACATC |
| 1636_7 | 8 | [C/T] | TTTATGTCTTTAGCACTGGCTTGCCTACAAATCCAG |
| 16362_3 | 4 | [A/G] | GGAAGAGGGACTGCATAAATGTGCACTGTGGGTGTA |
| 16363_27 | 28 | [T/C] | TGGAGTTGTCCAGCAGATACATGCTCCTCTGGCTTG |
| 16364_8 | 9 | [C/T] | TGTCCATATTGAGCAGCACAGTGCAGCGCTGTCTCC |
| 16369_31 | 32 | [A/T] | TGAGCAGGGTGAGCACCACGGTGCTGTGCTGAGAGT |
| 16373_8 | 9 | [C/T] | TTGGGATTCCATGCAACTTGCTGCAGTTTAGATGCA |
| 16374_29 | 30 | [G/A] | CTGGTCTGTGATGCACTGGTATGCTGCAGGAGGAGA |
| 16376_11 | 12 | [A/G] | TGGTCTGCAGTAGCAGCTTTATGCTGGTCAGGGTCA |
| 16379_17 | 18 | [G/A] | TTGCATTTGGAAGCACCGCTGTGCACAGAGTCACAT |
| 16380_34 | 35 | [C/T] | GGGGCTACTGAAGCAGAAGATTGCTCCGTCTGCTCT |
| 16381_11 | 12 | [A/G] | CGACCAGCAGCAGCAGTGGCATGCCATAGGGAACTC |
| 16385_20 | 21 | [G/A] | GAAAATTTAGTGGCACTTCGGTGCGAGTGGTACGAT |
| 16387_1 | 2 | [G/A] | GGAACGGAACCGGCAGCGTGGTGCACGCGAGCTACG |
| 16388_29 | 30 | [A/G] | AGTCACTCACCAGCAGGTGGCTGCTCTCGAACAACA |
| 16389_30 | 31 | [C/T] | CCATCTGCATCAGCACGCTTCTGCAACTTTCCCAAA |
| 16392_25 | 26 | [A/C] | CCCAAGTGATCCGCACTCTTCTGCCATTGTTATTGG |
| 16399_27 | 28 | [T/C] | ACGTTAGCCATAGCATCCCCTTGCTTTTACTCCTGT |
| 16407_29 | 30 | [T/A] | GAAATCACTGTGGCATGAGAGTGCTGGAGTTTATCA |
| 1641_10 | 11 | [G/A] | GGTGTGAGAGGGGCATACAGGTGCTTCTCACCACTG |
| 16417_5 | 6 | [A/G] | ACCAGAGTCCATGCAGAAGGCTGCTAATTACTGGCT |
| 16419_19 | 20 | [A/G] | GAAGAAACACGTGCAGACGAGTGCGTGCGAGTGTGT |
| 16420_10 | 11 | [A/G] | TCACCGAATCAGGCACCAGAATGCACTGATGCCAGC |
| 16424_32 | 33 | [G/A] | GTGAATGCTCAGGCAGAGGGCTGCACTGCTGGGAGA |
| 16425_8 | 9 | [G/T] | AAAAGGGGGGAGGCAGAATCATGCAGTCATAGGTAC |
| 16427_32 | 33 | [G/A] | TTTTCATGTCTTGCATTGCACTGCAGCCACAGGATT |
| 16439_9 | 10 | [T/C] | CTGTGCTTATGTGCAGGCCGGTGCTTTATAACGCCT |
| 16442_30 | 31 | [G/A] | GTCAGCATGCCAGCATTACACTGCACAACTGAGTCG |
| 16444_17 | 18 | [A/G] | ATACACACATACGCACAAATATGCACACAGACACAT |
| 16446_24 | 25 | [C/T] | GTCATTTCTTATGCAACAGCTTGCCGCTCCATCTGG |
| 16449_20 | 21 | [C/T] | TCCACAATGCATGCATTTATCTGCTGTGGAAGAAAG |
| 16456_5 | 6 | [C/T] | ACTCTCCCTAAGGCAGTTTACTGCAGGTCTGCAGTT |
| 16459_31 | 32 | [T/C] | TCATGTCACTCGGCACAGGCTTGCATGCTACTGTAG |
| 16462_30 | 31 | [T/C] | TACTTCCCTTCTGCACTCATCTGCTGCCTCTAAACA |
| 16464_33 | 34 | [T/C] | ATTCCACATACAGCAGTGCTCTGCTCTTCTGTTTCT |
| 16466_7 | 8 | [A/G] | AGGGCACATTCAGCACCGCCGTGCTTTCTGGGTAAG |
| 16468_8 | 9 | [G/A] | AGACCCTCGTGGGCAGTTGGGTGCAGCACAGCTTAT |
| 16469_26 | 27 | [A/G] | TTGGCTATTCAGGCACAACAATGCTCAATTGTTCCA |
| 1647_5 | 6 | [A/C] | TAAAACGCTTCGGCAGATAAGTGCAAAAGTTTGTGC |
| 16473_8 | 9 | [C/T] | TTGGTAGACGAGGCACCCTGGTGCCAGGCGTTGAGT |
| 1648_7 | 8 | [C/T] | GCTGCACCGTATGCAAGTTGCTGCTGCAGGTGCTAA |
| 16480_16 | 17 | [A/C] | GGTGCCATTGTTGCACACTGATGCAAATGTGCTGCA |
| 16482_3 | 4 | [C/A] | TACCCGCCAAAAGCAAAACACTGCGGCTTGTTTATA |
| 16488_28 | 29 | [G/A] | ACATCATGTGACGCACCCTTGTGCACTTGCATTCCT |
| 1649_24 | 25 | [G/A] | ATTGTTACCTGTGCATCAGACTGCGGAATCAAAATG |
| 16490_3 | 4 | [A/T] | ACCAGTTCACCTGCACACCCATGCAGTTATTCAATA |
| 16492_10 | 11 | [G/A] | CCTGGGCAGCGTGCACAGGGCTGCCCAGTGCTTCAG |
| 16496_7 | 8 | [G/A] | ACTGACTGAAGTGCAGTGCAGTGCTGTGGCTCCGGG |
| 16499_17 | 18 | [A/G] | TACAAGTACAAGGCAACGAACTGCAGTAGAGACCTT |
| 16502_8 | 9 | [C/T] | GTTATGCACGATGCACGTACCTGCACGATCTTATAC |
| 16505_7 | 8 | [G/C] | TGAAGGTGGGACGCAGCGGGATGCTCGTCTACGGCC |
| 16507_34 | 35 | [A/T] | CTTACCAAAATGGCAGATTAGTGCTCACCTGCGAAA |
| 16509_16 | 17 | [C/A] | GTCTTGGAGTGGGCACCTGGATGCTTTACCTGCTTA |
| 1651_19 | 20 | [G/A] | TTTCAGCTACTTGCAGGCCGCTGCCAGTTACATTTC |
| 16511_10 | 11 | [C/G] | GTGTGTTAATCTGCAGGGTGGTGCTCAGCCCAACCC |
| 16517_28 | 29 | [C/T] | AAAAGAAAGCAGGCAGCTACATGCCTTTCTGACACA |
| 16518_19 | 20 | [G/C] | ATCCAATTTACTGCAGCTGCATGCTCGAATCTGGAC |
| 16525_32 | 33 | [G/A] | TGGGATAGCTGTGCATTGTGATGCTTAGTCAGGGTT |
| 16528_1 | 2 | [C/A] | TCAAGCAGTTAAGCACCCGTTTGCGTCTGTTGTGGG |
| 16529_28 | 29 | [G/A] | GCTTTAGATCCAGCACATCACTGCAGGCGCTTTAGA |
| 16531_34 | 35 | [T/C] | ACTTTCTCCATCGCATTAGAGTGCGAGTGCTGCTTC |
| 16532_28 | 29 | [A/G] | AGACAGGATGGTGCATTATTCTGCATGAAAGCGTAC |
| 16533_5 | 6 | [A/T] | ATCACATGCCCTGCAAAAACATGCTGGAAATGCACC |
| 16538_9 | 10 | [T/C] | ACATAGCGCTCAGCACATTTCTGCTTTAACACACCC |
| 16539_6 | 7 | [G/A] | CTTGTGGATGCAGCATTTTAGTGCACACACATCCTT |
| 16547_24 | 25 | [G/A] | ATCTGCAGCGATGCATCACACTGCGAGGTATTTTTG |
| 16551_19 | 20 | [A/T] | CCATATCCTGAAGCACTACAGTGCCACCTTCGGGGG |
| 16554_3 | 4 | [G/A] | TATGAAGCCAAAGCAGTGCAATGCAGCCAGTGTGCT |
| 16557_24 | 25 | [A/G] | AAGTACATCAGCGCACGTCTTTGCAAGTGCTGTTTC |
| 16558_26 | 27 | [C/T] | TGAACTCGATTTGCAGCCTTTTGCCTCCAAACTCTG |
| 16559_19 | 20 | [C/T] | GCGAGATGCGTCGCAGGTCCGTGCGCCGTGCCAGGA |
| 16560_27 | 28 | [A/G] | AAGTATTCAGGCGCAGTTGGATGCCGAAGCAGAGAG |
| 16561_19 | 20 | [G/T] | AAACCCACAAGTGCAGATTGCTGCAGCTGTACACAC |
| 16572_17 | 18 | [G/A] | GAGGAAGCAGAAGCATTGAGCTGCCAAGACACTTCC |
| 16575_15 | 16 | [T/C] | CTCTATTGCCTCGCATCATGTTGCCTGTAGTAAGAT |
| 16576_1 | 2 | [C/A] | CCAAACGGATTAGCAGAAGTCTGCGTGAAGAGTATT |
| 16577_32 | 33 | [C/T] | ATTGGTGTCGTAGCAGGTAGATGCAGCCGCTTCACA |
| 16585_2 | 3 | [G/C] | CTGTGATCGCATGCATTGGGGTGCGCTGAGATTCCT |
| 16593_5 | 6 | [G/C] | AGAGAGGCCTGGGCATATGGATGCTCTGCTTTGGTC |
| 16601_19 | 20 | [G/C] | AGATGTGTGGCAGCAATACGCTGCTCCTCAAGGCTG |
| 16607_31 | 32 | [G/A] | TAAGTCGAGACTGCAGTGTCCTGCCATGGGGGGTGC |
| 16612_34 | 35 | [A/T] | GCACGCTTTTGTGCAGTATTTTGCGCAGTGTGTGAG |
| 16613_32 | 33 | [C/A] | TCTCGTTTTCCAGCACAGCACTGCAGGCCTTTCTGA |
| 16617_18 | 19 | [C/T] | TAAAAACTACAGGCAGGCCAATGCGGCACGCAACGA |
| 16618_9 | 10 | [C/T] | CTTCAGCTTCCAGCAGTTTGGTGCAGATGTTCCACA |
| 16631_4 | 5 | [G/A] | TGATGATGGCTGGCAGTGTTGTGCTGAGTCCCTGAT |
| 16632_11 | 12 | [C/T] | AACTACATGTACGCATGGCGTTGCCTCGTGTATTCT |
| 16639_26 | 27 | [C/T] | GCGAACAAATCGGCACCCAGATGCGACTGGTAGATC |
| 16642_17 | 18 | [T/A] | ACGGGTGTGTATGCAGTTTGTTGCGATGGACTGGCG |
| 16647_24 | 25 | [C/A] | GACACTGGCGCTGCAGCAAAGTGCCATGGCCAGGCA |
| 16652_29 | 30 | [T/A] | TAGCAGTTCGGTGCAATGTGATGCCACGCAGTTTAT |
| 16655_4 | 5 | [C/T] | CACTCATTGGCAGCAAGTCTGTGCCATTCGCCTTAC |
| 16656_9 | 10 | [C/T] | TTAGTTTTTCCAGCAAAAGCTTGCCAAAACCAAGTT |
| 16661_2 | 3 | [G/A] | GCGTGCATGTGTGCAGGCATGTGCCAATATAAAGTA |
| 16663_10 | 11 | [G/A] | TGAGCACACTGAGCAGAAATATGCCATATGCACTCA |
| 16664_8 | 9 | [A/G] | GATGTAAGACTTGCATACAGCTGCTTGGCCAAGGAA |
| 16668_11 | 12 | [G/T] | TTTCTGATCTTGGCACGAATATGCACGAAAGGTCTG |
| 1667_33 | 34 | [A/G] | CATCTGTGACACGCAGATGATTGCTTTCCAAATAAG |
| 16673_8 | 9 | [C/T] | AAAATCCTCGGTGCAGGTGACTGCCGATTGCCACCG |
| 16678_10 | 11 | [T/G] | ACTAGGACTGTGGCAGAAACCTGCACAGGGCAACAG |
| 16682_11 | 12 | [G/T] | TGTCAACTCCAGGCAGGACGCTGCCAAGCACAGCAA |
| 16683_4 | 5 | [G/A] | CACGGTGTGTGTGCAATGCAGTGCAGTACGTGGACT |
| 16685_4 | 5 | [C/G] | TCATCCACATCAGCACGCCGATGCTTAAACCTAATT |
| 16686_20 | 21 | [T/C] | GCTGTGGTCCTGGCAGGGAGTTGCGTGAGTGTTTCT |
| 16688_10 | 11 | [G/A] | GCGTGGCAGGGAGCAATATCCTGCTGAAAGAAGCTA |
| 16689_20 | 21 | [G/A] | CCCACCGAACTGGCAGACTCGTGCTTTAAACAGTAA |
| 16693_29 | 30 | [C/T] | CTGGGAGAAACAGCACTCCTCTGCAAGCTCGTAAGA |
| 16697_28 | 29 | [A/G] | GACACACGTCCTGCATCCTGCTGCCAAAAGGGAGTC |
| 16705_29 | 30 | [C/A] | AACAGCTGTGCAGCACACAGCTGCATGTGCACATTT |
| 16707_18 | 19 | [C/T] | GTCACTTTTTGTGCAGGACGATGCATTCCAAATTTT |
| 16716_5 | 6 | [C/A] | ATTTCCAAAGCTGCAAACTTGTGCGCTGCGGAAGCA |
| 16718_3 | 4 | [C/T] | GCTCTTAAAAATGCACCAGCCTGCAATGTGTACGCT |
| 16723_30 | 31 | [A/T] | ATTTACTACAGCGCAAATCCCTGCAGAGGTATCGGC |
| 16729_25 | 26 | [C/T] | CAGTCAGGAGTCGCACCGCGTTGCTCCTGTGCGCGC |
| 16737_15 | 16 | [C/A] | GCTGGTGCACCCGCACAACCCTGCTGAGAAGGAGGT |
| 16741_5 | 6 | [T/A] | TGGCATGAAATAGCAACGTCCTGCAAGGTCAGAGCA |
| 16744_10 | 11 | [G/A] | CAGACTCCACAGGCATTGATGTGCACGAATTCTTGG |
| 16746_8 | 9 | [G/A] | TCCAAGCTAAATGCAACAGTTTGCACCATGATGCAC |
| 16752_6 | 7 | [G/A] | GGCTCAGAGGAGGCAGTAGCTTGCGTGAGAAGCACA |
| 16754_5 | 6 | [A/T] | TCATAAGTCAAGGCACATGATTGCGTCTTTCTGCCA |
| 16756_24 | 25 | [T/A] | ATGTATTCCAGCGCATGGTCATGCTACAAAATGACT |
| 16757_34 | 35 | [G/T] | TGATGGCCCCATGCATTGTACTGCTTCCACACAATT |
| 16762_26 | 27 | [C/T] | AACTGTCCACTAGCAGCTTCATGCTTCATGCTAAGT |
| 1677_8 | 9 | [A/G] | CTGAACACATTGGCAGTCAGCTGCTGCTCCAAGAAA |
| 16771_16 | 17 | [T/C] | GTGATTAAAGATGCACTGTGATGCCGACGCCCATTT |
| 16772_18 | 19 | [G/A] | GGGTGCACATATGCAACCGTATGCGCGTGTGCGAGT |
| 16779_30 | 31 | [C/T] | CCACCAGGGTGGGCAGAACTCTGCGGCCTCCTCGAC |
| 1678_31 | 32 | [C/T] | AGCTCTTGCTGAGCAGGGCTCTGCTTTGAATCGCCA |
| 16780_15 | 16 | [A/C] | AAATTAAGGGTGGCAAGGTGGTGCTGCAGGTAGTGT |
| 16781_19 | 20 | [C/T] | CGGAGCTGAACCGCAAGTTCCTGCTCTTCGCCGACA |
| 16786_33 | 34 | [C/G] | TGTTTGTGCATTGCAGGCAGGTGCTGGCAAACCCCT |
| 16787_30 | 31 | [T/C] | CAGCACAGTGTTGCATGATGCTGCTTGATTTCCATT |
| 16789_30 | 31 | [A/G] | AACTGAGCAGTGGCAACTTGGTGCTAGTGGAATTTG |
| 16799_18 | 19 | [T/C] | CGTGCTGGATGAGCAACGTTTTGCGAAAGAAATCCT |
| 16813_20 | 21 | [G/A] | GATTGGAGAATTGCACGGACGTGCAACGAGTCATGC |
| 16819_10 | 11 | [T/C] | ACATGGTCCATTGCATACAGCTGCAGTACACCGACC |
| 16823_26 | 27 | [G/A] | TTTCCAGATGTTGCACACGGCTGCAGGGTGACATTA |
| 16826_29 | 30 | [C/T] | ATGCACAGGTGTGCATGACGCTGCCATGTTAGCATT |
| 16843_29 | 30 | [T/C] | CTTCAGACTGCAGCATGGTTTTGCGTTTCTCTATGA |
| 16846_17 | 18 | [A/G] | GCTTTCAATTTTGCAACACTGTGCTGCGGGTCTCTG |
| 16848_32 | 33 | [C/T] | AAGAACACATCAGCACTAGCATGCACGGCGCTCGAC |
| 16851_6 | 7 | [T/A] | AAAATATCAGCAGCAAGCATATGCAGCCATACCCTT |
| 16857_31 | 32 | [C/G] | CTGAGGAGAACCGCACCGGGCTGCTCCCGTCCACCT |
| 16861_32 | 33 | [G/A] | TCTACATTTTTGGCATTCAGCTGCCTCCTACAGAGC |
| 16862_10 | 11 | [T/A] | TCCAGCTGCTTAGCACCAGTATGCACATGTTCACAC |
| 16864_29 | 30 | [C/T] | ATACGAACATGGGCAATTACCTGCCCCATCATTGTA |
| 16865_33 | 34 | [T/C] | TCAGCAACAGCAGCACGAACCTGCGCCTGTGCGTGC |
| 16866_2 | 3 | [A/G] | AGAGAGGTCTTGGCAGATGCATGCAAATTCACAGTA |
| 16869_34 | 35 | [C/G] | TACAGTGTTAGTGCATCCCCCTGCGATTTGTCGCCA |
| 16870_26 | 27 | [T/C] | CTCTGATCTAGTGCATTTTTCTGCCTCCAGCGCATG |
| 16877_20 | 21 | [A/G] | CATGTGCCACTAGCACCTTCATGCTAAGAATTGGAT |
| 16886_34 | 35 | [T/A] | ATGGTCACCGTGGCAACTCAGTGCTTTAATTTAATT |
| 16893_5 | 6 | [C/T] | GCTCTCTATCCCGCATACAGCTGCTGCTCCTGGATA |
| 16898_5 | 6 | [T/C] | AAGTGTCATTTAGCAAGGCTCTGCTCCCTGGGCATA |
| 16903_34 | 35 | [T/A] | GGATTGAGCAAGGCATTATATTGCAAGGTGTCATTG |
| 16905_20 | 21 | [A/G] | AACATACATGGAGCACCACTATGCCTACTGAACAGG |
| 16906_4 | 5 | [G/A] | CTCCGGCCTTTAGCAGCTTCCTGCGGAACTCCTCCG |
| 16911_10 | 11 | [C/A] | ACGCTACCTGCGGCACCACCGTGCTGCTCTGTTTTA |
| 16914_17 | 18 | [G/T] | CTGTCAATAGAGGCATAGTGGTGCGGTGGGTATTGC |
| 16919_28 | 29 | [A/G] | CTGACAGAAAAAGCAGCTGCTTGCTGAAACCTGGGA |
| 16922_10 | 11 | [G/A] | GGATACCGCTGAGCAGGCTCATGCTATAAGGGACAG |
| 16927_17 | 18 | [C/T] | CAGCTTTCATGTGCATTCGATTGCATACACCTGTAA |
| 16930_18 | 19 | [G/A] | TTGTTTTGGTCAGCACAGGAGTGCCTTCTCACCTGC |
| 16938_18 | 19 | [T/C] | TCCCACATCACAGCATGCTACTGCCTCTAGCCAGGT |
| 16939_30 | 31 | [T/C] | CTACGCTGGTCAGCATGGAGCTGCTGGCCGTAGTCT |
| 16941_9 | 10 | [T/C] | GCGTCACCATGTGCATGACAATGCTGCTCCACCCGA |
| 1695_33 | 34 | [G/C] | TTGAGTACCTGAGCAGGAATGTGCCAAAAATGTGTT |
| 16953_32 | 33 | [G/A] | ACACTGTCCTCAGCAGGGTTGTGCCATGGTGTGAAG |
| 16959_32 | 33 | [T/C] | CAGAAGCCTATTGCATCATGTTGCTGGTGGCATTTT |
| 16961_8 | 9 | [G/T] | TTTAATGGGTTGGCACAGTTTTGCCTCACTACACCA |
| 16966_15 | 16 | [G/A] | CACTATCCACAGGCAGACTGCTGCAATTACAGGTAA |
| 16967_28 | 29 | [T/C] | AAGGACACATGTGCACCAGACTGCGCAATGTAAACA |
| 16973_10 | 11 | [G/A] | AGAGAGGAAGGAGCAGCACGGTGCAAACCCTGCACT |
| 16974_24 | 25 | [A/G] | TATCTCTGACAGGCAGAGTAATGCAGCATCGAGGTG |
| 16978_1 | 2 | [T/C] | CTCCTTCCTGCTGCAGAGCTTTGCATGGCTGCCGGA |
| 1698_5 | 6 | [G/A] | AACGTGAGACTGGCATACAGCTGCTCAGCCATGAAA |
| 16980_24 | 25 | [T/C] | TATTGACATATGGCAAGCTAATGCTGCGTTCATGAC |
| 16984_20 | 21 | [A/G] | GGCCCAGCAGTGGCAGCTTGATGCTGGTGGCATTCA |
| 16986_19 | 20 | [G/A] | TCTTGCCTAACGGCAGAGCACTGCGACACACGCTCT |
| 16988_4 | 5 | [G/A] | CTGGGAAACATGGCAGGCGGCTGCCATGTAGCCTTT |
| 16989_1 | 2 | [C/A] | TCTATCTTGTGTGCAGTGGTCTGCCTATTCTTGCTC |
| 16990_7 | 8 | [G/A] | GAAAGCGGTTCGGCATCTTACTGCTTGACACCTAGG |
| 16996_9 | 10 | [T/C] | CAATGCCTGTGGGCAAGACTGTGCCATAAAGGGCTA |
| 16999_26 | 27 | [T/A] | GGAGGATGAGATGCAAGAGGATGCGGTACAACAGGA |
| 17_31 | 32 | [T/G] | GTAGCAAATCCTGCAATCGAATGCAGGAGTGTTTTT |
| 17001_8 | 9 | [G/A] | AAATGGCCGCGTGCACTGTGCTGCTGCATGTCAGCT |
| 17002_8 | 9 | [A/T] | TCATGTCCAGAAGCAGGGAGATGCCCCAGGTCCACT |
| 17008_18 | 19 | [T/C] | AATGCCAATCAAGCAGCATGATGCTGGGCTGTGAGT |
| 17010_2 | 3 | [A/C] | AAACAAACATTTGCACACCATTGCACAAAGGCATAT |
| 17013_4 | 5 | [C/T] | TTCACCCCCAGAGCATGACGCTGCCACCGCCATGCT |
| 17015_9 | 10 | [G/A] | TACATGCAGGAAGCACACACATGCGTATGCAAACAG |
| 17017_33 | 34 | [A/C] | AAAGCTCCGGGTGCAAATCACTGCTCACTCACTAGA |
| 17019_8 | 9 | [T/A] | AACTGACTTAATGCAACGGCATGCACCCTGAACACA |
| 17028_4 | 5 | [G/A] | GTGTGTGTGAGTGCAAGTCCTTGCTTGCTTTACCAT |
| 17029_34 | 35 | [A/T] | GCGGAGCATTTCGCAACTCCGTGCGGATCTAAAAAA |
| 17034_18 | 19 | [G/A] | GGGAAAAGCAGTGCAGAAGGCTGCAGAGGCACCCAG |
| 17041_25 | 26 | [A/G] | GCCACAGAGGTGGCATTGCACTGCTATCCCTCTGGA |
| 17043_9 | 10 | [G/A] | TCATCAAATGAGGCACCTTTTTGCTCCACAGTCTTG |
| 17045_9 | 10 | [G/A] | TGAAAAGCAGAGGCAGCAACATGCTGAAACAGGACA |
| 17051_11 | 12 | [G/A] | CTGTGGGTCAAGGCAGGGCCGTGCCAGCATGTCACG |
| 17055_17 | 18 | [C/T] | CCGGTGACTAATGCAGTCTGCTGCTACCATCGTCTA |
| 17056_3 | 4 | [A/T] | TGTAAGCCAATGGCAGCGCGCTGCGCTGGAACATAC |
| 17058_2 | 3 | [C/T] | TACGTTACATTTGCATCATTGTGCTGAATCTGGGCT |
| 17064_8 | 9 | [T/C] | TAAGTTATTTATGCATCAGCTTGCTGTCTGACCAGC |
| 17065_25 | 26 | [A/T] | CCAGGTGACAGGGCAGTGCCGTGCCAGTGAAGTGCT |
| 17066_32 | 33 | [G/T] | AGGTCATCTGGTGCATGTGTTTGCACAAGAATGGTA |
| 17068_31 | 32 | [G/A] | TCAGCGTTGTCCGCACTCAGCTGCTTTCCCAGCACT |
| 17070_11 | 12 | [T/C] | GTTCAGTTACATGCACTGGCCTGCTTGTTTCTCTTT |
| 17073_25 | 26 | [G/C] | GAGAAAGGACCAGCAGTGGAATGCTGTGAGCAGATT |
| 17079_19 | 20 | [T/A] | GGTTAGTCAAAGGCAGGGGTTTGCTTTGTACACCTG |
| 17081_8 | 9 | [C/T] | AGAAAGCACGTTGCACTGACATGCACCTATGATCCT |
| 17084_11 | 12 | [T/G] | TCCGCGTTTCCGGCATACCTCTGCTCGTCCTCACAC |
| 17093_17 | 18 | [G/A] | TTTTACCATGTTGCAACGACATGCCATCTTTTCCCT |
| 17094_8 | 9 | [A/G] | ACACAGCCAGGAGCATCGTATTGCATTTTACATGGA |
| 17099_33 | 34 | [C/T] | TCTGTAGCCAGAGCAAGGAAATGCACCATGAAACGC |
| 17102_19 | 20 | [T/G] | AAGAAACCTGCTGCACGTCTGTGCACGTGTCTGCAC |
| 17106_5 | 6 | [A/T] | GTTTTATAAAGGGCAGTGTGGTGCTGCTGCATTGCA |
| 17108_10 | 11 | [A/T] | TCATGTACACAGGCAATCAGTTGCCATGGCAACTCC |
| 17109_25 | 26 | [G/A] | TATGGAAATGTAGCACAGGCCTGCAAAAACGCTTCA |
| 17112_18 | 19 | [C/T] | ACCCGGCCTCCAGCAGGGCGGTGCTCACCAGTCAGA |
| 17125_20 | 21 | [A/G] | GAGGATCTCTCAGCAAGCTAGTGCGCTAGCAGGCAG |
| 17126_19 | 20 | [T/G] | TAACTCCTACAAGCACAATTTTGCAGCTGTGGTAGA |
| 1713_15 | 16 | [T/C] | GCAGCTCTTGGGGCATGCGGATGCCATTGTGCACAA |
| 17133_5 | 6 | [T/A] | CAGCCTGGTCGTGCAGTGGACTGCTGACTTACAATG |
| 17137_8 | 9 | [G/A] | GGGAAGTGGAGAGCAATTTGTTGCCTCCTGTGAGAA |
| 17139_4 | 5 | [C/T] | CTCTCGTGTCCCGCAGCTTTCTGCACAAGATTGTGT |
| 17140_17 | 18 | [G/C] | CAGGTCCTGCATGCAGTGAACTGCTGACCGAAAGTA |
| 17147_34 | 35 | [G/A] | ATGAAACAGTGAGCAAAGCCATGCAAGTTGCCTGGG |
| 17150_10 | 11 | [A/G] | CATGCAAGCAGAGCAGTCGAATGCTAAAGTTAACGC |
| 17151_28 | 29 | [T/C] | TGAATTCAGCGTGCAGCGTGTTGCACTGTAACGTCG |
| 17158_32 | 33 | [G/A] | ACTCAGACGAGAGCACCAGAGTGCTTCAGTGAGAGA |
| 17165_16 | 17 | [G/A] | CAGCGCAGGAACGCACGGATATGCAACACCAAAAAC |
| 17166_18 | 19 | [T/C] | TTGTAAAAACAAGCAGCTTTGTGCTCATGAATGCTG |
| 17170_33 | 34 | [C/A] | CATGACCATCTTGCAGAGGAATGCACACACACACAC |
| 17174_34 | 35 | [A/T] | GCTCCTAGGATGGCATGGTGATGCCATAGGTAGGAC |
| 17175_9 | 10 | [C/T] | TGGCTCTGTTAGGCAGTCGGTTGCTCTGTTCCTCCA |
| 17176_26 | 27 | [T/C] | TGTGTGTGACACGCAGGGGGATGCAATTGCGGAAAA |
| 17182_34 | 35 | [G/T] | GCCGCAGCACGAGCAAAACCCTGCGCGTGAAGCTGA |
| 17190_18 | 19 | [C/T] | TGTTTGGGTTTTGCACCACACTGCCAGATAGATAGT |
| 17191_9 | 10 | [G/A] | AATGCAGCGGACGCAGAGACATGCAGCACACCAGAA |
| 17192_18 | 19 | [G/A] | CATGCTGGCCTGGCAGGAGGCTGCAGAAGAAAGCTG |
| 17199_30 | 31 | [G/A] | AGCATCTGAACAGCAAGTCGGTGCACGTCTGAATAG |
| 17202_3 | 4 | [C/T] | ACGCGTGTTTCAGCATGATAATGCTTCTGTACACAA |
| 17205_10 | 11 | [G/A] | TTTGTTGGGGGAGCACTGCTGTGCAGACCTCCACAA |
| 17208_34 | 35 | [A/T] | CACTCTATCCAAGCAACAAATTGCGATTTGGTGCAG |
| 17217_32 | 33 | [T/C] | GACTGGTCCTACGCAGGTGAGTGCTACTGGGTTACC |
| 17218_20 | 21 | [A/G] | TAACAAGTAAAAGCAGACACATGCCGACATCTAGTG |
| 17224_28 | 29 | [G/A] | AGCAGAGGAGCAGCAGCAGTCTGCACTCGCTGCACC |
| 17225_24 | 25 | [T/C] | TGTCTGACAGAAGCACGCGCCTGCTGTTGCTGTTAG |
| 17232_5 | 6 | [G/A] | CACATGCAGTTGGCAATTAGGTGCCATGCCCAAGGG |
| 17236_3 | 4 | [C/T] | AGCCCAATGACTGCAGAGCGCTGCTAAGGTTAGCTT |
| 17237_32 | 33 | [T/C] | CCCTGTTTGTTTGCATCATTGTGCCCGTTTGGTGCC |
| 17238_24 | 25 | [G/A] | AAATTTACTGATGCACGTTGATGCGCCAGCATAAGA |
| 17243_26 | 27 | [C/T] | TACAAGAGAAAGGCAGATTCATGCCTCGGTTCTCAG |
| 17244_26 | 27 | [A/G] | TCATGTTTCCCAGCACAATTCTGCCAACGCTTCGTC |
| 17248_8 | 9 | [A/C] | GTGTTAGCACTCGCATGGCAGTGCTGCATGATGGTC |
| 17264_7 | 8 | [C/T] | GACTGAACGTTAGCATCATGCTGCAGTCCCCAGGCT |
| 17265_1 | 2 | [C/A] | ACGGACGGCTCTGCACCACGCTGCCTACTGTGGAGA |
| 17268_25 | 26 | [G/T] | TTGAACTTTTGTGCATTGCGGTGCCGTGTGTGTGTG |
| 17269_34 | 35 | [G/A] | TAGCGGAGGCCGGCAGGAAATTGCACTCTCCTCCGG |
| 17270_1 | 2 | [G/A] | GGACAAGTGGAGGCAAAACCATGCCAGGGAAATAAA |
| 17273_2 | 3 | [A/G] | GAAAGAGAGAGTGCACGACCGTGCGTGCGTGTGTGC |
| 17274_24 | 25 | [G/A] | GCACTGATAACAGCATGCAGCTGCGGTGGATGGTAT |
| 17275_31 | 32 | [C/T] | CGTTTCAGTGCCGCATCTGCATGCGCAGCTTCAGCC |
| 17280_34 | 35 | [A/T] | AATGGAAGCATTGCAGCACCTTGCCTTAGTGTTTAA |
| 17285_11 | 12 | [T/C] | GGGCCAACTAGTGCATCCAGCTGCTCACAAAACTCT |
| 17288_8 | 9 | [T/G] | GTGGTAGCTCTTGCATGTTCTTGCGGTTCGGAAAAG |
| 17291_34 | 35 | [A/T] | TTGCTAAACGACGCAATTTAATGCACTCGCTTTAAT |
| 17297_4 | 5 | [A/G] | GCCCATGTACTAGCACTGGTTTGCTGGTTGTCCTTC |
| 17303_3 | 4 | [G/C] | AAAGTTGAGGGTGCAAAAGTTTGCACAAAACGAAAC |
| 17304_6 | 7 | [T/C] | ACATCCTGTAAGGCAAACTGGTGCTAAACATCCCAA |
| 17306_28 | 29 | [A/G] | ATGCCACGTTTTGCAAAAAGCTGCCCTGAAATAAAT |
| 17307_8 | 9 | [C/T] | ATGTTTTACTGAGCATCTGGCTGCAGTTCATCTGGA |
| 17310_7 | 8 | [C/T] | ACGATAGCGAGAGCATGGGATTGCAAACAGGAGGCG |
| 17311_6 | 7 | [G/A] | TGAAAGGCTACAGCACCAGCATGCAGAGCAGGAGGG |
| 17321_31 | 32 | [G/A] | GGCCTGTTGGCTGCAGTGCAATGCATTTCTCGAATT |
| 17322_11 | 12 | [A/G] | CAATGTTGTGAAGCAGGACTGTGCCACATATGTTTA |
| 17328_20 | 21 | [C/A] | GAGCTGTGCGAGGCAGAGGGCTGCCGGTTGGAAGAC |
| 17332_28 | 29 | [A/C] | AAGGAGGTAGTAGCAACCAGGTGCTGCTATTCAAAT |
| 17333_8 | 9 | [C/T] | AGCGCGATCCGCGCATACAGATGCAGCCGACGGTGG |
| 17339_24 | 25 | [C/T] | AAGTGAGGGAAAGCACAATGCTGCCATATCCTGTGA |
| 17340_10 | 11 | [G/A] | TTACTGCTCTGAGCAGTGTTGTGCCTCCTGGTTTGT |
| 17343_4 | 5 | [G/A] | GCACGCACACACGCACATGTCTGCACAGACATATGG |
| 17350_29 | 30 | [G/A] | TGGTTTGCTCAGGCATCCATGTGCAAATTGATCATG |
| 17351_7 | 8 | [A/G] | GTTTGGTACGCAGCATCCTTGTGCTGACTCTTTTAA |
| 17352_20 | 21 | [G/A] | ATATTACAAGCGGCAGGCGCGTGCCTCTGATCTGCA |
| 17353_5 | 6 | [G/T] | TGTGTTTGTGTGGCAGGGTTATGCTGACGAGATGGA |
| 17355_16 | 17 | [G/A] | AAATGTGTGCTGGCAAGTTACTGCTAGTCACACTGC |
| 17359_7 | 8 | [T/C] | TTTGAGCTATGAGCAGCGGCATGCGATCTCTGTTAC |
| 17365_3 | 4 | [C/T] | AAACGCACTAGTGCAATTAGTTGCATCATCTGACCT |
| 17370_17 | 18 | [T/C] | CAGGCCACTGAGGCATGTTATTGCTAAGTGTGCTCT |
| 17373_16 | 17 | [T/C] | CCCCATTCGAGTGCAATTCCATGCTTTCTTCAAGCA |
| 17375_24 | 25 | [A/G] | GGTGATGGCTGTGCACCACGCTGCAGGCTATTAATG |
| 17376_5 | 6 | [C/T] | GTTTTCACGAACGCACAGAAATGCCAACTACACAAA |
| 17378_30 | 31 | [A/G] | TCTGATGCTATGGCAGTTGTCTGCTCTGACAGCCCT |
| 17383_34 | 35 | [C/T] | TTTCCGGTACAGGCAAGGCGGTGCCTGTGTTCGGCT |
| 17384_31 | 32 | [A/G] | CTTACCTCACATGCAGTGGTGTGCACTGGGCATTGT |
| 17389_3 | 4 | [G/A] | AATGAGGCCAAGGCAAAAGACTGCTGTGAAAACAAA |
| 17390_31 | 32 | [G/A] | CCATAACCTTCAGCATTCCTCTGCAGAAGTGGAGGA |
| 1740_27 | 28 | [T/C] | TCCCACAAAGCTGCAGTCATTTGCACTTAATCGACA |
| 17405_8 | 9 | [T/C] | TTAGTCGGTAAAGCACGCGTGTGCATGCTCAAATTA |
| 17407_2 | 3 | [C/T] | TTCCCAGTTCCTGCAGGGACATGCGTGGGTCTTTAC |
| 17408_6 | 7 | [C/T] | CGCAGCCATTATGCAACCTTATGCAAATCGAGCAGG |
| 17414_18 | 19 | [G/A] | ACAGCCAGCAGTGCAGCCGAGTGCTCCAATAGGATG |
| 17416_4 | 5 | [G/A] | CACTGAGTAGAGGCACTTGTGTGCATCATTGCTGAA |
| 17418_1 | 2 | [A/G] | TAGAACAGATGTGCAAAGGTGTGCAAAACGAAACCA |
| 17420_15 | 16 | [C/G] | AAACCAGTTCCAGCACGACAATGCACATGTGTAACA |
| 17437_25 | 26 | [T/C] | TAGAAGAGTTTCGCAACAATCTGCTTGTGAGTGCAG |
| 17442_20 | 21 | [C/G] | GCCCATCCACTGGCAGCATACTGCTCTCGAACCCCA |
| 17443_15 | 16 | [T/G] | ATGATTTGATGAGCATGATGATGCTGAAGGTGGAGT |
| 17446_34 | 35 | [C/T] | AGAGGCCCAAGAGCACAGAGCTGCAGGTACAGGGCG |
| 17447_33 | 34 | [C/G] | GGCATGAGAATGGCAAGTATCTGCCCAAAGCAGCCG |
| 17448_16 | 17 | [G/A] | GTGTGTGTCTGTGCAGGTGGCTGCTCATGAAATACA |
| 17452_29 | 30 | [T/C] | AAACATCCCACAGCATGATGATGCCACCTTAATGCT |
| 17453_33 | 34 | [A/G] | GCGATAAAGCTTGCATGCAGCTGCTCAGCCATGAAA |
| 17463_4 | 5 | [C/T] | TACTCACAGCTGGCACAATTGTGCCACCACTACCTC |
| 17466_24 | 25 | [A/T] | TGATCAGGTGGAGCACAAAGATGCATTCCGTTCAAT |
| 17469_33 | 34 | [G/A] | TGTTGATGTTTTGCATCCTGCTGCCAAATCCAGGCA |
| 17470_34 | 35 | [G/A] | CCACGCCCGCCAGCAGGATGGTGCGACTCAGCTGGC |
| 17471_15 | 16 | [G/A] | CAGCCCCATCTGGCAGAACTGTGCAAACAAACAGCT |
| 17475_28 | 29 | [C/T] | ACAAATTCCAGGGCATCTGGATGCACGTCGCTCTTA |
| 17480_34 | 35 | [C/T] | TGGTCACAGGTTGCACATAATTGCAGCCATCATTCT |
| 17484_18 | 19 | [A/G] | GCAAACCCTTAAGCACACATCTGCTGGCAAGAACAA |
| 17486_29 | 30 | [G/A] | TAGTCTTAGAAGGCAGTCATGTGCTAAGTGGCAAAA |
| 17492_19 | 20 | [C/T] | GTGTGACCCTCTGCACCACCGTGCCACCCCAGAGGT |
| 17495_2 | 3 | [G/A] | ATGTGTCTTCAGGCAGCACTATGCGGTGTCGGGCAG |
| 17501_15 | 16 | [C/T] | TATATTAGGGTGGCACGGTGGTGCCGCATGTAATGT |
| 17504_33 | 34 | [C/T] | TGGCTGGTTAGTGCAGAGGGTTGCTCAAGAGCCCAA |
| 17508_8 | 9 | [T/C] | ATCAGGTGTAGGGCATCAATATGCCCTGACAGAGCC |
| 17509_32 | 33 | [C/T] | CAGACATCCTGGGCATAGAGGTGCCACAGTGCCCCC |
| 17513_3 | 4 | [G/A] | GCTGAAGGCTGTGCAGATGTTTGCTGAATACATGTC |
| 17515_9 | 10 | [T/C] | CTACCTCATTCAGCACATGCCTGCTGTTCTCTTACT |
| 17521_24 | 25 | [A/G] | TAGGCTTGGTGTGCAGGAGCATGCAAGGGTCACAAT |
| 17528_3 | 4 | [C/A] | GCACGGTAATAAGCAGTAAGCTGCATGTGTGAGGCC |
| 17534_10 | 11 | [C/T] | GGAAGAGGTCCGGCATGTTGATGCGACTGGAAGATG |
| 17537_6 | 7 | [G/A] | TCACAGGAAGGCGCAGAAATTTGCCCAAGGCGGTGC |
| 17539_1 | 2 | [C/A] | ACACTTTAAGGTGCATTCAGCTGCATGTCTCTCACA |
| 1754_31 | 32 | [A/T] | TATATTGTGGCAGCACAGTAATGCTGCAGGTAGTGT |
| 17553_34 | 35 | [T/G] | AAGGGGATGCCTGCATGGGTGTGCACGTGTGTGTTT |
| 17556_9 | 10 | [G/A] | GCAAATATGGCTGCAGCCTCGTGCCTACGATCACAG |
| 17558_28 | 29 | [A/G] | CCTCCAGCCCAGGCAGAGACCTGCCTCCATTTGAAG |
| 17564_9 | 10 | [G/A] | ACGCACAAAGTAGCAGGTGTTTGCTTCAAGTGGTAC |
| 17575_18 | 19 | [T/A] | TAAGGAAGACCTGCATGTTCCTGCATGACTGTACCT |
| 17576_7 | 8 | [A/G] | GCACACCACCCTGCACATTTTTGCTTCAACCCCTGC |
| 17580_26 | 27 | [G/A] | TCCATTACACTTGCATTCTTGTGCGCGCACACACAC |
| 17586_1 | 2 | [G/A] | CGCACCTCTATCGCACCTCTGTGCTGACCTTCAGCT |
| 17596_34 | 35 | [G/A] | GTGCACTGCATGGCAGGCCCATGCCACCAGGACGAC |
| 17601_19 | 20 | [C/A] | GGTTGTCTAGAAGCACAGTCATGCCATGCTGTGAGA |
| 17602_28 | 29 | [C/T] | TGCATCATGCATGCAATCCAATGCATGTCGCATGCT |
| 17606_3 | 4 | [T/A] | CACTGTCAATGAGCATAGCTGTGCTTCAGGATGCTG |
| 17614_34 | 35 | [G/C] | GAGACAGCTGACGCACAGCTGTGCAGACACAAGGGC |
| 17615_11 | 12 | [C/T] | GGGATGCATCCCGCAATGGGGTGCAAAATTCCACAT |
| 17616_2 | 3 | [T/A] | GGTCTTTTTCTGGCAGCTGACTGCCCTCATTCATTC |
| 17620_19 | 20 | [C/T] | CACACACACTGAGCAGGTTCGTGCTGCCATTTACTG |
| 17621_33 | 34 | [A/G] | GACAGCGACTGTGCATGTGTGTGCATGGTATGTAAG |
| 17627_26 | 27 | [A/G] | ATCCATGGAGCTGCACCAACATGCCAACCAACAATA |
| 17628_10 | 11 | [G/A] | GGAACAGTCCGAGCAGCGTTCTGCCGTCTGTCATGC |
| 17632_11 | 12 | [C/T] | TTATCCCTACCCGCATACGTTTGCTGACCACCAGTA |
| 17635_9 | 10 | [G/A] | ACTCCATACGGTGCACTTCTGTGCGCCACCAGGATA |
| 17639_16 | 17 | [G/A] | ACAATCACACACGCACGATCATGCCGTCTCGTGTCT |
| 17643_24 | 25 | [C/T] | ATGTGAACTGAAGCATCTAATTGCCGCTCAGCCCCT |
| 17647_3 | 4 | [A/T] | ATTAAAAGGCCTGCAAGCAGCTGCTCGGCCATGAAA |
| 17651_19 | 20 | [C/T] | CTTTAGACAGCAGCATCAGCGTGCGCTCTATACACA |
| 17657_30 | 31 | [G/A] | GCTTGTGGGGAGGCAGACACTTGCAACACCGTCTAT |
| 17661_26 | 27 | [G/A] | CAGATTTAAAACGCAGCACACTGCTCGGATTTTCCT |
| 17662_7 | 8 | [C/T] | TCTGTTGCTGGGGCACAGAGGTGCTGCAGGCAGTAT |
| 17663_27 | 28 | [T/G] | GTGTCAAATGCAGCAATTGTTTGCTGCTGGTTATTG |
| 17667_5 | 6 | [C/G] | TCCCACAGGACAGCATCGGCTTGCAGAGATTCTGAC |
| 17670_32 | 33 | [A/G] | GATTGTACAGGCGCAGAAACATGCCAGGCTACACAC |
| 17671_10 | 11 | [C/T] | AACATTCCCTCAGCATGATGTTGCCTCCACCTTGCT |
| 17675_8 | 9 | [C/T] | TAAGAGAGCAGGGCAAACGTGTGCGGTGAGATGCAG |
| 17676_28 | 29 | [C/G] | GCATGTGTGACAGCAAAAGCCTGCTTGACAGCATCC |
| 17678_34 | 35 | [T/A] | ATGCGCATATCTGCACAATGATGCCACAAGGTTTTA |
| 17685_25 | 26 | [A/G] | GCAAATACAGGAGCAGACCAGTGCCGAGCATGTAAG |
| 17686_5 | 6 | [G/A] | GGTCTGGCGTCTGCATACATTTGCTCCTGTGTGTTG |
| 17690_16 | 17 | [G/A] | GGACCGATCATAGCAAGTGGCTGCTAGTCCGAAGGT |
| 17692_17 | 18 | [C/T] | ACCAACAAACTAGCAGACCTCTGCATGCTGTCATTC |
| 17694_3 | 4 | [G/T] | ATTGGATGTCAAGCACTTATTTGCCGAAACACTGGG |
| 17695_16 | 17 | [G/A] | CTTCCTCAGCCGGCACGAATTTGCGCCGTGTCCTGA |
| 17702_30 | 31 | [G/A] | GCTGATGCCTGTGCAGCCGGCTGCAGACCTGGGCTG |
| 17707_7 | 8 | [G/A] | CAAGTGTGAGCTGCAGTGCAGTGCAGTGTCCTCTCC |
| 17709_15 | 16 | [C/T] | GCTGCAGCGCGTGCACGGCAGTGCCGAAATCCAGAG |
| 17713_19 | 20 | [T/C] | CAGAACGCGAGTGCACGGCTGTGCTACAGGAGTTTG |
| 17716_24 | 25 | [T/G] | TGATGCGTTTGGGCACATGCCTGCTCAGGACACGTT |
| 17719_16 | 17 | [G/A] | TCACTGCTCCGGGCACGTGTTTGCTGCACAGATGGG |
| 1772_7 | 8 | [A/G] | TTGCTTCAGATGGCATCACTGTGCATCTGCACAAGG |
| 17727_33 | 34 | [G/A] | CACCACCCTGTAGCACCAACGTGCCACCCTGACAAA |
| 1773_18 | 19 | [A/G] | CCCCATTTCAAGGCAGTTAACTGCACACATGTACAC |
| 17732_19 | 20 | [A/G] | GAGGGAGAGGTGGCAGTGCAATGCAGCTCGTCGATG |
| 17733_19 | 20 | [T/C] | AATCAACATTCCGCAGCAATGTGCACAGACCAACAC |
| 17738_20 | 21 | [G/A] | GCACACACGTACGCATGCACGTGCCACACGTGTCTC |
| 17740_11 | 12 | [G/A] | ACACCCACAGTGGCAGCATTATGCTTTAGGGCTGCT |
| 17750_16 | 17 | [C/T] | GCGTGTATGTGTGCACCGACCTGCTCCAGGCCCCTG |
| 17751_30 | 31 | [A/G] | GCCTCTAACAACGCACATGGATGCACAGTGAATTCC |
| 17752_4 | 5 | [G/A] | GCTCGCTCGCTCGCAGCGTTGTGCCTGCGTTGGAGA |
| 17757_16 | 17 | [C/T] | AACGAAAGCGCAGCACCTCCATGCGACTCACTTCAC |
| 17760_24 | 25 | [T/C] | TTGCAAACGTAAGCAAAGCGCTGCTATTTTAGCCTT |
| 17769_18 | 19 | [T/A] | AAGCTTGTTCCAGCATGGTAATGCTCCTGTGCACAA |
| 17771_25 | 26 | [T/C] | TCACATCTCATAGCATGCACGTGCATACACACACAC |
| 17775_32 | 33 | [G/T] | GTGAACAAGTGTGCAACCGTGTGCACGAAAGAGAGT |
| 17779_29 | 30 | [C/G] | GATCTACCTGTGGCACCACCGTGCCACCCCCTTTAA |
| 17780_10 | 11 | [G/A] | CTACAGCTGTGAGCAGACAACTGCAACACCCATTGC |
| 17784_5 | 6 | [A/T] | ACACAAACACATGCACGTGTGTGCAAAGACACCCAC |
| 17786_19 | 20 | [C/T] | AATCAAACTCGTGCACGCTCGTGCACCGTTCTCCTG |
| 17787_9 | 10 | [G/A] | GTAACACCTGAGGCAGCCAGCTGCTTTCTGCATGAG |
| 17793_8 | 9 | [A/C] | AAGAGACCCGGAGCAGAACGCTGCTCTTTGATGTAA |
| 17797_18 | 19 | [C/T] | GTTTTATACTGAGCACTTCGCTGCTAGACACTTCAT |
| 17798_30 | 31 | [C/T] | CTGAGGTCAAGAGCACCAAACTGCACAGCTCATTCT |
| 17799_19 | 20 | [A/T] | ACCTGTCCATTCGCAGAACAATGCAGAGTCACACTT |
| 17801_34 | 35 | [C/A] | TGGAGGAGGCGCGCAGCCACGTGCAACCACACACCA |
| 17804_31 | 32 | [G/T] | GACGTTTCCACTGCAGTTTGATGCTCATTGCGGTGA |
| 17813_5 | 6 | [T/A] | AAGCATATCAGGGCAGTTATGTGCCTTGCCCAAGGG |
| 17818_17 | 18 | [A/T] | GTATGTTAAACAGCAGCAGTCTGCAGACAATGTGGA |
| 17822_7 | 8 | [C/T] | AAGATCTCAGTTGCAGGACCATGCTGTTTGTTAAAG |
| 17823_24 | 25 | [G/A] | CAAGGACCTGCGGCATTGTGGTGCGTTTTGTTTTCT |
| 17831_15 | 16 | [C/T] | AAGAATTTGAACGCACGCAGGTGCTTTAAATTTCAA |
| 17832_30 | 31 | [T/G] | AAGGTTAAAGAGGCATGTGTCTGCTGCTGGTCGATA |
| 17833_7 | 8 | [G/A] | GCTCCGAGTCGAGCACCACGCTGCTGCGGTTACTCA |
| 17835_10 | 11 | [A/G] | AGTGGTTGGGATGCACTTGTGTGCCCACTGTCTAAA |
| 17843_8 | 9 | [C/T] | TTTGAGCATGACGCAGCTGTGTGCTTTCTGTCTGAC |
| 17844_3 | 4 | [C/T] | TCTCCATGTAGCGCACCACACTGCCATGATCACCGC |
| 17847_7 | 8 | [T/C] | TTGTGCTTCCGAGCATGTCATTGCTACGTGATTGAC |
| 17849_30 | 31 | [G/A] | AAATAAAACGATGCAGCTTAGTGCAATCGGAATAAA |
| 17858_34 | 35 | [T/C] | AAGGACGTGTTTGCATGCTGCTGCAGCCAAACACTG |
| 17862_24 | 25 | [G/A] | GATTGTTTTTCAGCACCGGTCTGCGAAGCAATTTCG |
| 17864_10 | 11 | [G/A] | CCTTTAACACGTGCATGTTTCTGCTGCAGCTAGCTG |
| 17869_3 | 4 | [T/C] | CTTTGCACTGTAGCACTTCTTTGCCAGTGGTGCATT |
| 17873_7 | 8 | [G/A] | TCACATTGCGTTGCATTACCTTGCCCATGAAAAGTT |
| 17876_11 | 12 | [C/T] | ACAGACGCACCCGCATAATACTGCAGTGCAAGAAGG |
| 17880_16 | 17 | [C/T] | ACTGTGGTGAGTGCAACACACTGCAAGATTTGTCAC |
| 17885_18 | 19 | [G/C] | CAAGTGGAAACGGCAACTGGCTGCAGAACTCGAGGC |
| 17887_33 | 34 | [T/C] | TGCTCTACCTCAGCAAGAGACTGCATGAGCATTCAG |
| 17892_10 | 11 | [G/A] | AAATGCAGACGGGCAAGTGTGTGCCAAGAGTGCATT |
| 17894_24 | 25 | [C/T] | AAGCAGCGGTGTGCATGCTGATGCCGTTTTGACAAT |
| 17897_1 | 2 | [T/C] | GTGCATGCACATGCATCTGTCTGCGTGTACATGAAT |
| 179_27 | 28 | [C/T] | ATGCTGCAAGATGCACTTGTCTGCCTTCACTTGTCT |
| 17901_6 | 7 | [T/A] | ATGTGTTATTAGGCATTTTGCTGCCTCAGCTCTTTG |
| 17907_18 | 19 | [T/C] | TCCAGGACGATAGCATCCTCCTGCTCAAATTGAGCC |
| 17918_19 | 20 | [C/T] | CGGTCGCCATCAGCACATCCATGCCCATGAACATCA |
| 17919_5 | 6 | [T/G] | AAATCTACTCCAGCATGTGCTTGCAAGCATCATGAT |
| 17923_9 | 10 | [C/T] | GGGCGTCGGTCTGCACTTACCTGCATGCAGAATTAG |
| 17924_4 | 5 | [G/A] | TATCGGTCACGTGCACTTTCCTGCTTGCAACAGCAG |
| 17931_5 | 6 | [A/C] | TCTCCACAGAAAGCACCGCCCTGCTAATTCTTACCT |
| 17935_3 | 4 | [G/A] | AACGGGCAACATGCATCCGGCTGCTTTTATAAACAA |
| 1794_9 | 10 | [C/T] | ATGATGCTCCGGGCAATGTTCTGCTGGAAAACTTTG |
| 17944_24 | 25 | [T/A] | ACTGTGGGAACGGCAAACCCGTGCTAATCATATTTG |
| 17951_19 | 20 | [C/T] | AGATGACGCTATGCATATCCGTGCTGCTGGCGCTTA |
| 17959_30 | 31 | [C/T] | CTGTTGAATTTTGCATCGGGCTGCAGTGTTCTTTCA |
| 17961_25 | 26 | [C/T] | TGGCCCAATCTGGCAACACCGTGCTCCAGAGCGACC |
| 17965_18 | 19 | [G/T] | TCCCAGGAGGAGGCATTGGTCTGCTCGGACAAGCTC |
| 17967_3 | 4 | [A/T] | CTTAAAAAAGCTGCACGAAGCTGCTTGTGTTATTCT |
| 17975_29 | 30 | [G/A] | AGTGTCCAAACTGCACGTCCCTGCAGGACGATCTGC |
| 17976_17 | 18 | [C/T] | CCAGGGACTGTTGCAGCCCGTTGCGTAATTGGACTT |
| 17978_25 | 26 | [C/A] | GACAGAGAGAGAGCAATGCCTTGCACACATTCAAGA |
| 17988_11 | 12 | [C/T] | ACGCAGCTCCTCGCACTTAGCTGCAGTTTTACACGA |
| 17992_24 | 25 | [G/A] | ACTAAATACTGAGCACAAAAATGCGGAGCCAACCAT |
| 17999_33 | 34 | [T/A] | AGTTAAACACTCGCAAAGTTTTGCCTTCTGGCATGG |
| 18002_4 | 5 | [G/A] | TCAAGGTGACCTGCATACTTTTGCTGTAAAGGCAGG |
| 18005_9 | 10 | [C/A] | GAACAAACACACGCAGTCACATGCAGGAGGTCATAC |
| 1801_11 | 12 | [C/T] | GACACTGTTAGCGCATTAGCCTGCAGCATAAACACT |
| 18012_25 | 26 | [C/T] | TTTGGATTGTTTGCATGTGCGTGCTCGCCTAAGCAT |
| 18016_31 | 32 | [A/G] | ATTGATTCCCAGGCACGTGTGTGCTCAAGTCACTAC |
| 18018_6 | 7 | [G/A] | CCTCTGGAGGCTGCAAATGTGTGCTGCAAGTGTGAC |
| 1802_24 | 25 | [C/T] | AAATCTATTGCGGCAAGTATGTGCCGATTTGCACAT |
| 18023_3 | 4 | [A/T] | GTGTGCAGAGGTGCAGGCGCATGCTGCCAGAGCGAT |
| 18027_19 | 20 | [G/A] | CGAGTGGGTTTTGCATGCAGCTGCCAGAACTAAATT |
| 18032_25 | 26 | [G/A] | AAAATGGGCGAAGCAGCCAACTGCAGAGATAAAAGT |
| 18033_20 | 21 | [G/A] | GACAGGAGCGGTGCAACTGTGTGCCACCTTGATAAT |
| 18034_17 | 18 | [A/G] | GTTATCCAGGTGGCATGATGGTGCCACAGGTAGTGC |
| 18040_10 | 11 | [C/T] | CAACTGCCTACGGCAGGATTTTGCTTTGCTGTGGCT |
| 18042_7 | 8 | [C/T] | CTGTGTGCTTATGCAAGTTTGTGCAGACTTGAGCAC |
| 18044_27 | 28 | [C/T] | AATGTTGGTAAAGCAATGCAATGCCACCGGCTGCCA |
| 18050_2 | 3 | [C/T] | CACTTTACTTCTGCAGTGTGCTGCCTCTAATGGAGA |
| 18051_2 | 3 | [A/C] | CAAAAAGTGTTTGCAGCAGTGTGCAGTTTTTCGTTA |
| 18054_29 | 30 | [A/C] | CTTATGAAGAAAGCATCACCCTGCAGAGAACATCTC |
| 18057_27 | 28 | [C/T] | AAAACATGGCTTGCATGGAAATGCTGCCGAGTTAGA |
| 18059_30 | 31 | [C/T] | CGCGTCTTCGGAGCACGGCCCTGCCCGCCTCGAGTA |
| 18060_18 | 19 | [G/A] | AATTTTCCAGGCGCACCAGCGTGCAATGCATCCAAT |
| 18064_25 | 26 | [T/C] | TTTCCACAGCCTGCAGTCTGTTGCATGAGGCATGGA |
| 18072_24 | 25 | [A/C] | TTTAATGTCTCAGCAGCTTCATGCAAGCCTTAAGTC |
| 18074_26 | 27 | [G/A] | AACTACACAACCGCAGGAAAGTGCGCGAGATGGAGG |
| 18081_1 | 2 | [A/G] | CACACTCACTCAGCAAAAATCTGCCTCAAATTCCAA |
| 18084_20 | 21 | [C/A] | GGCCAGAGTCCAGCACTTCCCTGCACTAGCACATCT |
| 18085_4 | 5 | [A/G] | AGCTAAGGCTGTGCACAGACATGCCAAAAGACCAGC |
| 18086_2 | 3 | [G/A] | AGGAAACTCACAGCAAATTGTTGCTGCTTCTTCCAT |
| 18088_2 | 3 | [C/T] | TGCGCGATATTGGCACGGTTCTGCTTGCACGCCATC |
| 18089_24 | 25 | [G/A] | TATAGTAGTGGGGCAGGGATGTGCGGGGGGTGACTA |
| 18098_8 | 9 | [T/C] | ACTCGTCATGCCGCACTTCTGTGCCATCTAAAACGG |
| 1810_32 | 33 | [G/A] | CCATTCACTCATGCAGTGAACTGCACAGGTCTGGTT |
| 18100_16 | 17 | [C/T] | CCTCCTTGCCTGGCACCTCGGTGCTGATCCCTGCAC |
| 18102_10 | 11 | [C/T] | ATCGGTCCTTCAGCATCTTCATGCACTGCCTGGTGG |
| 18103_25 | 26 | [A/T] | ACACCACAGTGTGCACATGACTGCCATTCTCACTGT |
| 18104_30 | 31 | [G/A] | AAGTAGTGAATAGCAGATCCGTGCCACACTGTCCTG |
| 18107_8 | 9 | [G/A] | TCACGCTGGAAAGCACACACATGCCGAAAAACACAT |
| 1811_28 | 29 | [T/C] | AATAAATGCCAAGCACCAGGCTGCCTTGTCAAACAA |
| 18111_19 | 20 | [A/C] | CGCACCTGTACAGCACACAAATGCAACTTGGACTAG |
| 18116_17 | 18 | [G/A] | TGTGTTAGGGTGGCACGGTGGTGCCACTCAATAACT |
| 18117_27 | 28 | [C/T] | CAGGAGGCAGAAGCAATCACATGCTTCCTTTGAGTT |
| 18124_27 | 28 | [G/A] | CCTCCTGAAAGTGCACTTTGCTGCTAAGTTTCCGGC |
| 1813_10 | 11 | [T/A] | TCGGTATGAATAGCATGTAATTGCGTGTAGCCTCCA |
| 18133_3 | 4 | [A/G] | AGCAGCAGCGCGGCAGCGCAGTGCGGAGTGTGTTCT |
| 18134_19 | 20 | [C/T] | AAACCAACATCAGCAGCAACGTGCCAAATCTCACTT |
| 18136_5 | 6 | [T/A] | GCCTTTCCCACAGCACAACGTTGCCAGCTCCATTTC |
| 18137_1 | 2 | [G/A] | TGAGGCATGAGAGCACTACCCTGCAAGCACGTTTAA |
| 18138_5 | 6 | [G/A] | CCACAACATTCTGCAGCGACATGCCATCCCACCCGG |
| 18139_15 | 16 | [A/G] | AAGTGTGTATCGGCAAGCATTTGCGGTTATGGAAAA |
| 18143_28 | 29 | [C/T] | CAAAGCAATGAGGCAGAGTGCTGCAGGTCGTCCTCT |
| 18144_16 | 17 | [A/G] | GACGCACTGCCGGCACAGACCTGCTGATACGGCACA |
| 1815_34 | 35 | [G/A] | GGGCTAGTGTTAGCATGCTAGTGCAGCTGCATAAGA |
| 18152_5 | 6 | [G/A] | GGTTCGAATCCAGCACAGAGCTGCTGCTGCATTCAT |
| 18162_3 | 4 | [C/T] | ATTCCTTGCATGGCAACTCACTGCATGGAAATCCAG |
| 18168_15 | 16 | [A/G] | GGGCATGAAGCAGCAACACATTGCTACACTTCAAAC |
| 18179_7 | 8 | [C/A] | GTGACCTCATGTGCAGCCCCCTGCTATGCCATGCAT |
| 18192_26 | 27 | [C/T] | CTCAGAGCTGCAGCAGCACACTGCCTCCTCATTACA |
| 18194_4 | 5 | [A/T] | CACAACATGCACGCACACACATGCCAGTACGAAGCT |
| 18204_16 | 17 | [G/A] | CTCAGCTGTGTGGCACGTTTCTGCAATGCTAAGCAA |
| 18206_17 | 18 | [G/A] | CAGATCTCTGCAGCATCGACATGCAGCCCTCAGAAT |
| 18214_9 | 10 | [T/C] | TGTGGTCGGTTGGCACGGTGGTGCTACAGGTGAAGT |
| 18215_4 | 5 | [A/G] | GTGCACTCACACGCACACATCTGCAAACACTTTAGA |
| 18220_15 | 16 | [C/T] | AAACGTACTCACGCACGGTTCTGCGATATCGCCTGA |
| 18225_19 | 20 | [G/T] | AGGATATGATTTGCAGCCCGTTGCAGCGTCTCACTT |
| 18232_4 | 5 | [C/A] | TCTTCGCTCCACGCAAAGAACTGCTGTGAAACTGAC |
| 18233_5 | 6 | [G/A] | ATCATGGTGAGCGCATATGACTGCGGCCAAAAGAGG |
| 18238_30 | 31 | [T/A] | GTAAAGCTCACTGCACAAACCTGCTTACTGTACAAT |
| 18239_27 | 28 | [G/T] | TGGGTAATGGGGGCATCCATTTGCAGCGTCGGCTTT |
| 18241_8 | 9 | [C/T] | CGTTTTCTCTGTGCATGGAAGTGCTGCTTTTGCTCT |
| 18243_26 | 27 | [G/A] | CGTCGATTTCAGGCACAGGCATGCCAGAATCCCAGC |
| 18250_20 | 21 | [C/T] | ATTAAACGGGTTGCAATAAACTGCCCTGGGTATGTG |
| 18265_1 | 2 | [T/C] | CTCTGTGTTACAGCACACTGCTGCAACATTGTGTCA |
| 18266_31 | 32 | [C/T] | AGTGCGTCCATAGCACCACGCTGCACAGAACCGCCA |
| 1827_31 | 32 | [G/A] | ACTCGCTTCCTAGCAATGCCATGCAAGAAAAGAGTT |
| 18272_7 | 8 | [C/A] | GTTGGCACAGGGGCATCATTCTGCAGCTAATTAACT |
| 18276_2 | 3 | [A/G] | GAAGATGTTATTGCAAGGCACTGCTATGGCCACAGT |
| 18290_15 | 16 | [G/A] | CAGGTATAATCTGCAGGGTGGTGCGGTGCAGGAGCG |
| 18293_7 | 8 | [G/T] | GAAATTAGGTTGGCAAGGTGTTGCTGCAGGTATTGC |
| 18300_7 | 8 | [T/C] | TTCCTCGTCAGTGCACACACATGCAGGGTGTGAGTA |
| 18308_10 | 11 | [C/T] | GGATGAAGAACGGCATTGTGCTGCAGCCTGAAGGGA |
| 18310_16 | 17 | [C/T] | TGCTGGCGATGGGCACCGGCGTGCTGACGACAGGAC |
| 18316_24 | 25 | [A/T] | CTGGCAACAGGAGCAACGAGATGCAAAATTTTGCTA |
| 18322_11 | 12 | [A/G] | AGAACACACACGGCACCGCCGTGCTCCGTGTTCTCA |
| 18327_20 | 21 | [C/T] | AACCCCAGATCTGCAGAGACCTGCATTACCTGCAGT |
| 18330_7 | 8 | [G/A] | GAAGAATGGGTGGCACGGTGGTGCTGCGGGTAGTGT |
| 18331_27 | 28 | [G/A] | AAAATGATATCCGCAGCCCTTTGCGAGGATGATCCA |
| 18334_18 | 19 | [C/A] | GTAGCACCTTCTGCAGTACAGTGCACCTTCTTGGGT |
| 1834_26 | 27 | [C/T] | TATTCACTTCATGCACAGTGGTGCTGCCAGACCTCT |
| 18351_34 | 35 | [G/A] | ATACTGGCACCAGCATCAACATGCAGGCCAACCCGA |
| 1836_9 | 10 | [A/T] | ATCCACTAGACTGCACAGGGGTGCTTTGATTAGTCT |
| 18360_2 | 3 | [G/A] | ACATCTAAGCCAGCATTTAACTGCCACAATACAAAG |
| 18362_10 | 11 | [C/T] | GCTGTAGTGACGGCAGACGGATGCCGTAGTGCTGCT |
| 18366_17 | 18 | [C/A] | TCATGGCAATTTGCAAACACTTGCATGGCTGTTAAA |
| 18374_31 | 32 | [C/G] | TGTTTACTTCCTGCACTTTGATGCCCACCCACTGGA |
| 18382_2 | 3 | [A/G] | TCAGCTAGCGTTGCAGGTCCGTGCACTGATTCATCT |
| 18386_8 | 9 | [T/C] | TCCTTTAATGGTGCATACGGCTGCACTGGGTGAACC |
| 18387_20 | 21 | [C/T] | TGAGCACTCAGAGCAAAGTGCTGCTCATACAAGGCA |
| 1839_15 | 16 | [C/T] | GTACATGTAAGTGCACAAGAGTGCGTGCGTGTACAT |
| 18392_27 | 28 | [T/A] | GTACAACGGGCAGCACAGTGGTGCAGGTAGGTGTGT |
| 18393_8 | 9 | [A/G] | GCTGCTCCAGTGGCATATTGTTGCACCCTCTGAGAC |
| 18397_18 | 19 | [T/A] | AGGCGAACCATAGCAGCATAATGCCTTCCATAGAGT |
| 18398_26 | 27 | [T/C] | CGGTGACTTTCTGCAACTCGCTGCAGTGCCTGCCCA |
| 18401_26 | 27 | [T/C] | AAGCTCAGCCGTGCAGGCACCTGCCATAACACGCAC |
| 18402_5 | 6 | [T/C] | GAGCTCGAATTAGCAATCAATTGCTCTGCCATCGTG |
| 18406_32 | 33 | [G/A] | GCTCGAGCAATTGCAATCCTCTGCTTTTGTCCGCCA |
| 18408_26 | 27 | [C/A] | GCACAGAAGATCGCACAAAACTGCTGCACAATAAAG |
| 18409_34 | 35 | [G/A] | AGCACGGTGGGAGCAAAGCACTGCAGATTGTAGTGG |
| 18411_27 | 28 | [T/C] | CAGTTTTGGACAGCATGCCCATGCAGCTTATCACCT |
| 18426_3 | 4 | [G/A] | ACCGCTTTCAACGCAGCCCAGTGCTCGAACTTGATT |
| 18429_5 | 6 | [T/G] | AATTGTTGAATGGCAGCAAGATGCTCTTGGTACGAT |
| 18430_24 | 25 | [G/A] | GTTGCAGCCGCTGCATTTCTGTGCGTACAGGCAGCT |
| 18432_18 | 19 | [A/T] | GGTGGCGATGAAGCACCCTTCTGCAGTGACAGCTTG |
| 18434_28 | 29 | [C/T] | GGGAGATGAGGCGCATTGTTGTGCCGCTCCAAAAAT |
| 18440_17 | 18 | [A/G] | ACCAAGTTACAAGCAACAGCGTGCGAGCCAAACCTC |
| 18446_1 | 2 | [G/A] | TGAAGGGGGTCAGCATCTCAGTGCTTGGTTCCCATA |
| 18452_10 | 11 | [G/A] | CACACACCATGAGCATTTTGCTGCACACCCTCTATG |
| 18454_32 | 33 | [G/A] | CCATATCACCTCGCAAAGCTTTGCATAATCAGGCAG |
| 18456_28 | 29 | [G/T] | GAAAAACAGAATGCACTGAACTGCCCTAGGTGTGAG |
| 18463_1 | 2 | [G/A] | TGAGCACTCATTGCACTGAGGTGCTCAGTGAAGCTG |
| 18466_24 | 25 | [T/C] | GAGGAAAGCTGAGCACGCGTTTGCTTACCGCACTTT |
| 18469_2 | 3 | [T/A] | CCTTATCTAGCTGCACAAGTGTGCCTGGGGTTACTG |
| 1847_5 | 6 | [C/T] | TGAATCACCACTGCATGGTTTTGCTCCGGGTCAGTT |
| 18473_4 | 5 | [G/T] | TTGTGTGTGTGTGCATGCACTTGCTCAGTTGCATCC |
| 18478_2 | 3 | [T/A] | GAAGCGCTATGGGCAAAAGTATGCGGACACACAGCC |
| 18481_8 | 9 | [G/C] | GATGTAAGGCTCGCATGCAGCTGCTCGGCTGTAATA |
| 18482_26 | 27 | [C/T] | GATTTGAAGGGGGCAAGCGAGTGCAACGCCAACCTA |
| 18485_5 | 6 | [G/T] | CTCTCGAACGCTGCACTGACGTGCAACCACTGCTTC |
| 1849_32 | 33 | [C/T] | TTTTTTAAACCAGCAAGTGTTTGCGTTGCTGCCCAG |
| 18495_28 | 29 | [T/C] | GGTCTGTGTGCTGCAACCACCTGCTTCATGAAGTCA |
| 18502_1 | 2 | [A/T] | TAGTAAGGCAGAGCAGTGTTCTGCACACAAGAGATC |
| 18506_34 | 35 | [A/G] | CATTGAGGAGGCGCAGTCTCCTGCGCTCTGACTCAT |
| 18507_18 | 19 | [G/A] | GGCCTCGTGCCTGCAGCCGAATGCTGATATTCTTCT |
| 1851_16 | 17 | [T/A] | AATGCGATCGGCGCAGTAAAATGCCAGGTGAGGCGA |
| 18511_19 | 20 | [G/A] | GATGTAAGACCTGCATGCAGCTGCACAACCATTAAG |
| 18515_11 | 12 | [T/C] | GAAATACACAGTGCACAACTGTGCAGACACACCATG |
| 18517_8 | 9 | [C/T] | ACCAGTTACACAGCAGCAGCCTGCCGAGCAGCAACA |
| 18518_7 | 8 | [C/T] | GAACTATCGAAGGCAAATGTCTGCCGCATTTACCAC |
| 18534_30 | 31 | [G/T] | CACATGGCCAGAGCATCAAGCTGCCTCAATGACACC |
| 18535_1 | 2 | [C/T] | CCGTGGTAACCTGCACACGACTGCCCTCGCATGCCA |
| 18552_30 | 31 | [A/G] | AACACATTGCATGCAAAACACTGCCAATGAAACAGA |
| 18557_27 | 28 | [T/C] | GCACCTGTGCGTGCACGTGTGTGCGTATAGATCCTC |
| 1856_26 | 27 | [G/A] | CGCACAGCATTGGCACATCAATGCAGGCAGGCATAA |
| 18562_3 | 4 | [A/G] | GCCAGATGCTGAGCAAGTACGTGCCTGGATTTATTT |
| 18563_1 | 2 | [T/C] | GTTCATCCCTGTGCATGCCTGTGCATTTTTTAACGA |
| 18565_26 | 27 | [T/C] | TCTGCTAGCTGGGCAATTCTCTGCTCTGCTTCCTTT |
| 18567_2 | 3 | [C/T] | ATCTGGAGTCCGGCAAGTGATTGCAGCTCTCAAACA |
| 18568_25 | 26 | [G/C] | CACGATAGACTGGCAGTGATCTGCTGCAGAGGAAAC |
| 18570_4 | 5 | [C/T] | TCCCCACGCGATGCACTGCCATGCTGACATGAGACT |
| 18572_6 | 7 | [C/T] | GAGGGACGTTCAGCAATTTCCTGCCCTTTTCTTTGA |
| 18573_33 | 34 | [G/A] | GCTCTCGGCATGGCAAACACTTGCTCCGCCACCGAC |
| 18575_17 | 18 | [T/A] | TTAAGCTCCGAAGCATTTTTCTGCCAGGGCATGGAG |
| 18577_24 | 25 | [A/G] | AAGAAGTCACAGGCATGATGATGCATTACGGTGAGC |
| 18578_20 | 21 | [C/T] | TAATAGGATAACGCACAGCGCTGCCCGATCAACCCC |
| 18580_18 | 19 | [C/T] | TGCATTTAGTAGGCAGTTCGTTGCAACTGGGAGAAC |
| 18586_2 | 3 | [C/T] | CTCATCAGTCCTGCATGCTTGTGCTAAAGTGTACTT |
| 1859_30 | 31 | [G/A] | CCAGGGAGTAGCGCACGATGTTGCCATGGCGACTGG |
| 18593_27 | 28 | [T/A] | AGAACCCAGTGTGCAGGCATCTGCTTTTCTTCTAAC |
| 18594_10 | 11 | [C/T] | CAACAATACTCAGCAGAGATCTGCGCTCACAGTGGT |
| 18596_2 | 3 | [C/G] | GGCACCATGGATGCACACAAATGCCAGCAGCAACAG |
| 18601_1 | 2 | [A/C] | GAGTCTCTACAGGCATTCCGCTGCATTATGCGATGC |
| 18607_33 | 34 | [C/T] | AATGGCAATAAGGCATCATGCTGCGTCCATTTACTG |
| 18619_26 | 27 | [A/G] | TGGACTGAGCAAGCAGGTCCCTGCCAAATCCGTCAC |
| 18627_4 | 5 | [T/C] | ACCGTACAGTCGGCATGACTGTGCTGCAGCTGAAAG |
| 18628_15 | 16 | [C/G] | AAGGCATGTTGTGCAGCGGATTGCTGGGATACTTTA |
| 1863_8 | 9 | [G/C] | ACATCAGTGTGTGCAATCAGCTGCTAGTTTACCTAA |
| 18631_16 | 17 | [A/C] | ACTGAATAAAGGGCACACCCGTGCACAGCGTGTACC |
| 18643_24 | 25 | [C/T] | AAAGTGGAGAAGGCATTCAGATGCCGCAGGTGGATT |
| 18647_6 | 7 | [C/T] | ACTGAGCGCTCTGCAGATTTCTGCTACAGATTTGAA |
| 18648_31 | 32 | [A/G] | GACACGACGCGTGCATCCATCTGCAGACGCCAGCAG |
| 18652_1 | 2 | [C/G] | TCTATCCACTGTGCAGACATCTGCCCCTGTCCTCGG |
| 18655_26 | 27 | [T/A] | GGGCAGGTGGCAGCAGATATGTGCTTTAACAAGGCC |
| 18658_1 | 2 | [C/T] | CCACACCCCTCTGCACATCGCTGCCATCCACAGACA |
| 1866_31 | 32 | [T/C] | ACACACACTGAAGCATGAACCTGCAGAGAGCTCGGT |
| 18668_16 | 17 | [T/C] | TTAATACCAGATGCACTTTCCTGCCAGTTCTCCATC |
| 18669_29 | 30 | [C/A] | AACCAGTGGTTTGCATTTCTTTGCCGTGGCACAAAA |
| 18670_31 | 32 | [A/G] | AAAGATTCTCCAGCAGCAATTTGCCAAGGCCATCAC |
| 18682_2 | 3 | [T/A] | TGTACATGTGAGGCAGCAATCTGCTGGATTGCCTGC |
| 18684_34 | 35 | [A/G] | TTCAAGGCGAAAGCACAGGATTGCATAATGTGGTAA |
| 18686_30 | 31 | [G/A] | CTGCCCACTGGAGCATTTTACTGCAGTATCGCAATA |
| 18687_33 | 34 | [C/T] | TACCTCCCACGTGCACTATCATGCATCCTGGTTCTT |
| 18689_8 | 9 | [G/C] | GCAGCGGACACAGCAGCAGGCTGCTCACACACAGCA |
| 18692_3 | 4 | [T/C] | CAATAAAGTTAAGCACAACTGTGCAACTGAGCGTGC |
| 18694_5 | 6 | [G/A] | TTAACGAGCACTGCACCAACATGCCTGACCTTTTCC |
| 18696_10 | 11 | [C/A] | TTCCCGCACTAGGCACAGGGATGCGGTTTCTCTCCC |
| 18698_19 | 20 | [C/T] | AAGTCATCCAAGGCATGCTCCTGCGAAAAAGGTTGT |
| 18704_10 | 11 | [C/T] | TGGTCACACTCCGCATCCAGCTGCAAAGGTCAGACT |
| 18705_25 | 26 | [A/G] | TTCAGCAATCTGGCAACACGCTGCTAAAGAGAAAAA |
| 18706_17 | 18 | [C/T] | GATCACTGCAGGGCATTCAAATGCTGCTCACCTAAC |
| 18707_10 | 11 | [C/T] | ATCTAGCCTGCAGCAGCACAGTGCCAAGGGAGCAGC |
| 18709_15 | 16 | [T/A] | GCCCTGCCTGCAGCATTACCATGCTGCCCAGTGATA |
| 18711_8 | 9 | [G/A] | GGCACTGTGGAGGCAGCTCACTGCTCTGCCCAGGGT |
| 18712_16 | 17 | [C/G] | TACGAGTACAAGGCAACGAAATGCTGTCAGCATCAA |
| 18719_5 | 6 | [A/G] | AGCCAAGGCATGGCAGAGCTGTGCATGCGTGAGTGA |
| 18724_29 | 30 | [C/G] | CCGCACGCAGACGCAGGCAGATGCTCCGTCCGCTTC |
| 18725_27 | 28 | [G/A] | GTGGTGCAGGGGGCAGCTCCATGCCCGAGCCTCGGC |
| 18726_17 | 18 | [A/C] | GGTGACCTAATGGCAGCACTTTGCCAGTTGGGATAT |
| 1873_3 | 4 | [A/T] | TTCAGCATTTGTGCATTGTGCTGCTATGTGCTATAT |
| 18731_28 | 29 | [T/C] | CACACAATTGGTGCACCAACCTGCCATTCGTTTGAT |
| 18732_32 | 33 | [G/A] | GGAACTGTTTCTGCAGCTCCTTGCTGGTCGGTGCTG |
| 18735_24 | 25 | [G/A] | ACCTGATTAAGAGCAATGATGTGCGGAGCCTACCAG |
| 18737_25 | 26 | [A/T] | AGCAGTGATGGGGCAGCCATATGCTAGTGGTTAAAA |
| 18742_26 | 27 | [G/A] | ATGTTCCCACAAGCATAGTAGTGCATGTGTGTGTGT |
| 18748_24 | 25 | [T/C] | TTATGAATGAGCGCATTGATCTGCTTAACCACGCCC |
| 1875_15 | 16 | [C/T] | TCCGATCACGTAGCACGGCAGTGCCCGAAATCCAAT |
| 18756_24 | 25 | [A/G] | TGGAAGACTTGTGCAGTAATGTGCACAAGGTGCTGG |
| 18771_29 | 30 | [C/A] | ATACCGACAGCAGCATAGAAGTGCGCACACACACAC |
| 18773_33 | 34 | [T/C] | GCGTAGACGGAAGCACAGGCGTGCATTACGACGTCT |
| 18777_34 | 35 | [T/C] | CTGAAAATCTCTGCATCCTGCTGCTAGATGTGCATG |
| 18779_1 | 2 | [C/A] | GCACCTTTCTCAGCAGCCACGTGCAACGCTGTTGCC |
| 18781_26 | 27 | [G/A] | ATTAGCAAATCAGCACACAGCTGCTAGCTATCAAGC |
| 18783_6 | 7 | [C/T] | GCACATCACTGGGCAGCAAAGTGCTGAGTGACGTTT |
| 18786_32 | 33 | [G/T] | TATATTAAATGAGCAGTTCGCTGCCTGAAGGAGTGG |
| 18794_17 | 18 | [C/T] | CCACAGTACACTGCATTCAGCTGCTAGAGGAGCGAA |
| 18801_3 | 4 | [G/C] | AAAGACGAGGGTGCAGTTACCTGCTAGGGGCTCCAA |
| 18802_17 | 18 | [G/A] | TAAGAGGCGTGGGCAATGAAGTGCAAAAAATACATG |
| 18818_4 | 5 | [C/T] | CTTTCTTTAAAGGCATAGCTCTGCCTGTGCTGTGGT |
| 18821_31 | 32 | [G/T] | TACGTATAAACAGCAGCGGATTGCATTGTTTGTCAC |
| 18828_3 | 4 | [A/C] | TACAGAACACACGCACAACACTGCTACACTAGAGAT |
| 1883_6 | 7 | [G/A] | CAATAAGGTCATGCATTAAAATGCATGCGGCGTCTT |
| 18831_27 | 28 | [G/A] | GAGAGAAAAGGTGCAGCATTTTGCTTTGACTTGAGT |
| 18836_4 | 5 | [A/G] | TCTCAGCTTTTTGCACTGCTTTGCAAGGCAAGGCCT |
| 1884_33 | 34 | [C/T] | ACAGGCAGCAGAGCATGCTAATGCCACTCCGCTCTA |
| 18849_10 | 11 | [G/T] | GCACGTCAAGGGGCATCGCTTTGCGTTTTATCTTAC |
| 18852_31 | 32 | [G/A] | ATCAGAATCGGAGCAAAACGGTGCATCGTGTGTGAA |
| 18855_19 | 20 | [G/C] | GAAGCAGGCAGTGCACTTCGCTGCAATCAGAACCAA |
| 18865_7 | 8 | [C/T] | GCCAGATCTCCAGCACAAAGCTGCATGGACATCTGA |
| 18868_6 | 7 | [C/A] | TGCCCCCTCCAGGCATCCACCTGCAGCTCAAAACAG |
| 18869_3 | 4 | [T/A] | TGGTTGGTGGAGGCAGCTAGTTGCTGGGGCAACAGC |
| 18873_16 | 17 | [C/T] | CCCAGATCTCTGGCACCATTGTGCACCATGCACTAA |
| 18877_1 | 2 | [C/G] | ACGTCTCTGCCAGCAGCTGCATGCACTCATCAAATG |
| 18879_3 | 4 | [C/T] | GGACCTGGGAAAGCAGGGCGCTGCAATTAAGACGCG |
| 18880_30 | 31 | [G/A] | GCACAACCTGTAGCACCACAGTGCTGCCTTGAGATA |
| 18884_16 | 17 | [C/T] | AAGCCCCACGATGCATCAGCATGCCCCTCCTCTTTC |
| 18886_4 | 5 | [A/T] | GTTTATTCGGGGGCATAGTCATGCTTCCAGTCTCCT |
| 18898_5 | 6 | [C/T] | CTGACCAGTGTTGCAGCAATGTGCCCCATCGGGGGG |
| 18902_16 | 17 | [T/C] | GCTGTTCGCATCGCATTCGGTTGCCGAATGAGCACC |
| 18912_2 | 3 | [G/C] | CGGCGCTGTGAGGCAGGGAAGTGCATCAATAACACT |
| 18916_24 | 25 | [C/T] | CATCACGGACAGGCAGAGGCATGCCGAGTATCACGA |
| 18919_19 | 20 | [T/G] | TTCCGTTCTAACGCACGGCTTTGCTCTATAACTCCA |
| 18923_30 | 31 | [A/G] | ACCCTGAATGTAGCAGCTTCATGCTGATGAAACCTG |
| 18924_29 | 30 | [G/A] | TGTGTGAAAGCAGCATGCATCTGCAGCCTGAATCAA |
| 18925_15 | 16 | [A/G] | ACACCATCTGCAGCAAGATGATGCTGCTGCAGCTCT |
| 18928_20 | 21 | [A/G] | TGTCTGTGCATTGCAGCAGCATGCGGTCATTCTCCC |
| 18929_25 | 26 | [C/T] | CATGCAGCTCTGGCACCACATTGCTCAGCTCTTTCC |
| 1894_15 | 16 | [G/A] | ATGGCCTACATGGCAAGGCAGTGCCAAGACAAATAC |
| 18948_31 | 32 | [G/A] | TGGAAGCGGAAGGCAGTTCGGTGCTAATTGGGATAG |
| 18949_2 | 3 | [A/G] | GCAGAATTTCCAGCAATTATCTGCGTGGCAGGAATA |
| 18951_3 | 4 | [T/C] | GCGTCTCGCTTTGCATTCTTATGCGGGCGTGTTTGG |
| 18958_15 | 16 | [C/T] | TTCTGCACTCGAGCACTGTGTTGCTCTGTTAGGATG |
| 18959_7 | 8 | [C/A] | CCTGGTACGATAGCAATGGTCTGCAAAGAGCTTATA |
| 18961_32 | 33 | [A/T] | CACATGAATTCAGCAGACATCTGCAAGAAAGCACCG |
| 18962_18 | 19 | [G/A] | GACTGACCTTCAGCACCTGAATGCTGCAGGAATGGA |
| 18965_3 | 4 | [G/A] | CCTGAGTAAGGTGCATCGAGGTGCCGCAACCACGAT |
| 18966_1 | 2 | [A/G] | CAGCTGCTAACAGCATTATGGTGCCACCACCATGCT |
| 18968_29 | 30 | [C/T] | TACAAGATGATGGCACAGATTTGCTGATGCGGTTTA |
| 18969_4 | 5 | [A/G] | CTCTGTCAGTCTGCACGCGAGTGCCTCGCTCGATTA |
| 18970_25 | 26 | [G/T] | GTCACCGACATAGCAGACCACTGCAGAGACACGTGG |
| 18980_29 | 30 | [T/G] | GCCTCTTTGGCTGCAATGATCTGCTTCTCTGCTTCT |
| 18984_31 | 32 | [C/T] | CACAGGTCCAATGCACATATTTGCCTCTAACCGCAA |
| 18989_16 | 17 | [A/T] | GTTTATAGTCAGGCAAACAGATGCTACCCAAGCACC |
| 18996_24 | 25 | [T/C] | ACATCTACAAGAGCAATAACCTGCTGGCACAGGTGA |
| 19004_6 | 7 | [G/A] | AATTCAGTGAGAGCATCGGGATGCGCTTTGGATGGA |
| 19005_20 | 21 | [T/C] | CAGAGGACCAAAGCAACAGCTTGCCTTTGATATAGC |
| 19012_29 | 30 | [G/A] | TCTCAGGGAAAAGCACAGAAATGCTTCACGGGGTTT |
| 19015_11 | 12 | [C/T] | TCATTTTCCTGCGCATTTGCATGCTGTCGAGGCGAA |
| 19029_7 | 8 | [G/A] | TATCTGTGAACTGCAACAAGATGCAGACAGCACTGG |
| 19030_27 | 28 | [G/A] | GAGGAAGACGATGCATACTTCTGCCATGATCTTGAA |
| 19037_3 | 4 | [A/T] | TCGAAGGAGACTGCAAGGTGATGCCACACTTAGAGA |
| 19043_26 | 27 | [T/C] | AAATTCCTCACAGCATGACATTGCCCTTGCCCCTGT |
| 19047_33 | 34 | [A/G] | CCTCCTGTGTCTGCATGCCACTGCGAGTCCAAGAAC |
| 19048_8 | 9 | [G/A] | AACAGGCAGAAAGCACCTTTGTGCTACCTGTCCTCT |
| 19050_27 | 28 | [G/A] | AAAAGACCTGGCGCAGTACGGTGCCGAGATAAAGGA |
| 19056_10 | 11 | [C/T] | TTTCGATTTACGGCACCCGCGTGCGATTCTGTTTGT |
| 19067_26 | 27 | [G/A] | TCCACCCAGATTGCAAAAAAATGCTAGAACACGTTT |
| 19071_6 | 7 | [G/A] | TAAAAGGCTGCTGCATTGATGTGCTGTCATCATGCA |
| 19075_34 | 35 | [C/T] | TAAAAGTGGAAAGCAAGGACATGCCAGAGGTAGCCA |
| 19078_24 | 25 | [A/G] | CTGATCAGGTCTGCATTGTTCTGCATAGGTGTTGTC |
| 19079_10 | 11 | [A/C] | AAAGGGGCCAAAGCACCAACTTGCAAAGAGCTTGAG |
| 19082_16 | 17 | [G/A] | GGAGAACAAAGAGCACGGGCATGCGGTGTTGAACAG |
| 19085_17 | 18 | [T/A] | TGGAAATGTGTCGCACTTGCCTGCAAAGCCAAGCCT |
| 1909_7 | 8 | [T/C] | ATTTTAGTCACTGCAGTCTTGTGCAGTGATGGAGAA |
| 19093_15 | 16 | [G/A] | GATCAGGGAGCGGCAGGAATGTGCATGTTTTTATAG |
| 19096_20 | 21 | [G/A] | TGATAAAGCGTGGCATTCATGTGCTCTTGACCAAGG |
| 19097_29 | 30 | [T/C] | TGGCTTTAACACGCACACTGCTGCTTCTTCACTTCA |
| 19098_5 | 6 | [G/C] | CTTCAGAAGGTGGCAGTAGCGTGCCACTGAAACAGT |
| 19101_33 | 34 | [T/G] | GTTTACAAGCTGGCAAACTTTTGCGGGTTTGTTTTT |
| 19107_24 | 25 | [C/A] | GATGTAAAGCCTGCATCCAGGTGCCTGGCCAAGGAA |
| 1912_1 | 2 | [T/A] | GTGTGTGTGTGTGCACACGCATGCCCACCCGGAGAA |
| 19120_19 | 20 | [C/T] | GTGTCACCTCGAGCATCCTCGTGCGATGGGAATCGA |
| 19124_15 | 16 | [C/T] | CCTGTTCTGAGTGCACGGCTGTGCCGACATTGTTAC |
| 19125_24 | 25 | [G/A] | TCCTTCTGTCATGCAGCCCTATGCGTACCTGCTCTG |
| 1913_4 | 5 | [C/T] | AACACTGCCACAGCATGATATTGCGACCCTCATGAT |
| 19143_32 | 33 | [T/A] | GACGTCACACCCGCACTGTGCTGCCTGATCATTAAA |
| 19144_25 | 26 | [C/T] | TCCCTGAGCTCTGCACTTTATTGCTCTGCTGCACAG |
| 19148_28 | 29 | [G/A] | TGTATGTTTCAAGCACCAGATTGCTGCTGTGTTTGG |
| 19161_19 | 20 | [C/T] | TAAAGCAGAGCAGCAGCTCCTTGCTGGAGATTTCCA |
| 19169_4 | 5 | [G/T] | ATTAGTGTACTAGCACAAAAATGCCCACATCGTCAG |
| 1917_28 | 29 | [G/A] | TACACAGCCTGGGCAGCCAGCTGCAGCTGAACCAGC |
| 19170_20 | 21 | [A/C] | CATTTTTAACAGGCACTTGTATGCGCCAAATGCAGC |
| 19173_11 | 12 | [C/T] | ACACACATGTGCGCATGGCTTTGCTCCTGTGTGCAT |
| 19180_3 | 4 | [G/A] | AATGGATAACCTGCATTGGACTGCCGCTCTGTGCAG |
| 19181_2 | 3 | [C/T] | CGCTGTCCGGAGGCACCCTGCTGCACCTGATTCCCG |
| 19188_9 | 10 | [G/A] | GGTATTTTTGAAGCATGTGAATGCAGCACCGTCAGC |
| 19189_20 | 21 | [A/G] | GCCAGGCAAGGTGCAGGAGCATGCTCTGAGCAGATA |
| 19194_1 | 2 | [G/A] | TGTTTTCTTGCTGCAGATTCATGCTCAGATCCACAC |
| 19198_34 | 35 | [C/G] | GACGAAGCTAATGCATGTGACTGCAGCGTCAGAGCT |
| 192_19 | 20 | [G/A] | GTGCATAAATGTGCAGTTAGATGCAGTGAGCGGTGC |
| 1920_15 | 16 | [A/G] | ACGTTACACTGCGCAAGAATGTGCTGCTTTCTAGTT |
| 19201_28 | 29 | [T/C] | AACACTGATTTGGCAGCTTTGTGCACGCCGTGGGAA |
| 19202_1 | 2 | [T/C] | GCCACTGCGTAGGCAGACGAGTGCGCAGACGCTCTC |
| 19210_2 | 3 | [C/T] | CTCCTCTCTCAAGCATGCTCATGCGTTCATGGGCCG |
| 19214_20 | 21 | [C/T] | ACGCATGCAAACGCAGCATTCTGCACTGCAGTTAGC |
| 19216_2 | 3 | [A/T] | ATATTATTGGTGGCAGAGTGGTGCTGTGGGTAGTGT |
| 19219_27 | 28 | [T/A] | GAAATCCCAGCAGCACCAAGCTGCCACTGTAGTACC |
| 19220_31 | 32 | [C/T] | GATGTATCCAGTGCATCCACATGCATGTATGCGCTC |
| 19221_4 | 5 | [G/A] | TGTGATTACCTTGCAGTTTCCTGCGGTGGAAGCGCG |
| 19223_34 | 35 | [T/A] | GACAGACGTGCTGCAGCTCTTTGCTCTTCATGTGTA |
| 19224_18 | 19 | [G/A] | GAAGGACCGCTGGCATCAGCGTGCATGGCACATGTT |
| 19225_32 | 33 | [G/A] | CAGGAAGCCCCGGCATCCTCCTGCAGCGCCACGCGT |
| 19226_24 | 25 | [G/A] | GTAGACGTGATTGCATTGGCCTGCGGGCAGAAAGGT |
| 19227_17 | 18 | [G/A] | ACTCACATGCTTGCAGTGATATGCCCCCCTCATCTA |
| 19228_6 | 7 | [C/A] | TGGCTCCCTATGGCAGGAAGATGCTCAATTCACTGT |
| 19230_8 | 9 | [A/C] | TTAGGAACAACAGCAAAACTGTGCACCAGGAGGTTT |
| 19235_28 | 29 | [A/G] | CCAGCTAAAGACGCAGCCTGCTGCGGCCAATGCTAA |
| 19238_33 | 34 | [C/T] | GATTCACTGCCTGCAGGGAATTGCACAATGCCACGT |
| 1924_2 | 3 | [C/G] | AACAAAATACCCGCATTGTGTTGCAACGTTCGTAGT |
| 19240_15 | 16 | [A/C] | CAAATAAGGTAAGCAACAACATGCCGGGGGAGCAGT |
| 19242_18 | 19 | [C/T] | ATTCATATGGCCGCAACTCAATGCATTTAGGCATGA |
| 19244_26 | 27 | [T/C] | GCAGACAGAGGCGCAGTTATTTGCTGTTTCTGGCAG |
| 19245_9 | 10 | [T/C] | TTTTATAATTGGGCACAGTGGTGCCGCAGGTAGTGT |
| 19246_18 | 19 | [G/A] | ATGCAAAGTTTGGCATCAGGTTGCTGGTGACTACAC |
| 19249_28 | 29 | [G/A] | CTCGCATGCCACGCAGGCATGTGCAGGCGGAGGACG |
| 19254_10 | 11 | [G/A] | AGCACGTCTCGAGCATTAAAATGCTGCAGGATGGCT |
| 19256_26 | 27 | [C/G] | GTCTCCTTAAGAGCAAATATGTGCACCGCTCCACAC |
| 19258_2 | 3 | [T/G] | ACTCCGCCTGGAGCAGGAGCCTGCTGCCGGCCTGCA |
| 19261_25 | 26 | [T/C] | ATGACAAACACGGCACCTCTTTGCTTGCCCTTTACG |
| 19263_9 | 10 | [T/C] | TATAGGAGGTATGCAGGCACTTGCTCTGCATCTGAC |
| 19266_25 | 26 | [G/T] | CTTGTGGTCAGTGCAGGAGTCTGCAGCAGTCATATC |
| 19268_7 | 8 | [T/A] | GATGTGGTGATGGCACGTGTTTGCCCACGACTGTAC |
| 19270_2 | 3 | [G/C] | CAGGGTACCAACGCACACACATGCACATCACAATTC |
| 19272_7 | 8 | [C/T] | ACCAGAGTGACTGCAGCAACCTGCACTGCTGCGCCG |
| 19275_33 | 34 | [G/A] | TATGACCAATTAGCAGAAACATGCTGACCAGCCGAG |
| 19282_10 | 11 | [T/G] | GTACACACCTTTGCACACCTGTGCACACCTGTGAGT |
| 19290_29 | 30 | [C/T] | CAGCAAAAGCTTGCAGAGAAATGCGAACGCGCTCGG |
| 19293_15 | 16 | [G/A] | AGTTGTAAAAGAGCAGACCGCTGCAGAACGTGTCCT |
| 19298_17 | 18 | [T/C] | TTGTATTGGTGAGCACCTTACTGCTGCCAAAACTCA |
| 19303_15 | 16 | [C/G] | CATAAGGTAACAGCACGTGGATGCTACACACCTTAG |
| 19305_8 | 9 | [G/A] | TCCACTAAGAAAGCATGAAGCTGCTGCTGTTTCCTT |
| 19309_4 | 5 | [A/C] | AGTGAAGCCGGGGCAAATACATGCTTCCTCTGAGAC |
| 1931_32 | 33 | [C/A] | GGCGTGGTGTTTGCATGCGGCTGCTCGTTCGCCTAT |
| 19310_1 | 2 | [G/A] | GGGTGCGATCACGCAAAAGTCTGCTTTATATGGAGG |
| 19316_25 | 26 | [C/T] | GTAATGGCGAGCGCATGCGTGTGCACGCTTGTGGCT |
| 19317_29 | 30 | [G/C] | ACAGTACACCCTGCAGAGATCTGCAGGAGGAGTTTC |
| 19318_34 | 35 | [G/A] | CTCTTCCGGGTTGCACGGTGGTGCCACAGGTGGTGC |
| 19319_26 | 27 | [C/T] | AATACTCTCCTGGCAGGCGTTTGCATCTGAATTCAA |
| 19322_2 | 3 | [G/A] | GCGGTGGGCACAGCAATAATGTGCAACAATTAGTCC |
| 19325_28 | 29 | [T/G] | TGGGATGCTTCTGCATGTGAGTGCCAATTAATCGAA |
| 19330_5 | 6 | [G/T] | TTGGGGTAACAAGCAAACCACTGCTCTGTAGTGCAA |
| 19335_28 | 29 | [G/A] | TAGGTTGGGGCGGCACAGTGGTGCAGCAGGTAGTGG |
| 1934_28 | 29 | [T/A] | CACAGACTCACAGCAAACTTCTGCTTGATGTTGAAG |
| 19340_24 | 25 | [A/C] | GCCTTGTGTAATGCAGTTGACTGCAAGCTTTAGCAG |
| 19344_28 | 29 | [G/A] | AGTACCCATACAGCAGTTCCCTGCTGGCGTAGAGGG |
| 19345_34 | 35 | [G/A] | CAGAGTTGTTGTGCAGGAGAATGCTGGAGGGAGAGA |
| 19350_16 | 17 | [G/T] | GTAAGCAGATGTGCATGGAGCTGCAAGCAGTCTCTT |
| 19353_9 | 10 | [C/T] | AAACAAGCCCGGGCATGCCAATGCCTACCAATTTAT |
| 19359_20 | 21 | [G/A] | TGTCATCCCTTTGCATATAAGTGCCATGGCGACCCT |
| 19368_25 | 26 | [C/T] | ATGTCATCATGGGCACAGGGATGCTCTGTAGCATTA |
| 19370_11 | 12 | [T/G] | TCCTGAGAAACTGCAATGCAGTGCCCCATGTCCCCA |
| 19374_19 | 20 | [C/T] | TGCGGCGATAAGGCACGAGCGTGCTGGAGATTGGGT |
| 19387_15 | 16 | [T/C] | CGTGGGACTGCCGCATAGTGTTGCTGTTAATGGATT |
| 19391_24 | 25 | [G/A] | AAACATTTGCCAGCAACCAAATGCGTGATGGAAAAA |
| 19392_26 | 27 | [C/T] | ACACAAACACAAGCAAACACTTGCCCCGTTTCTCCT |
| 19394_33 | 34 | [C/T] | CTTTGTTTGCATGCAAAATTTTGCAGCCTCCAGCAG |
| 19400_11 | 12 | [A/G] | ATGCTGTGCTAAGCAACTAAATGCAGAAATGGTCCA |
| 19403_28 | 29 | [G/A] | AAGGTGCTGCAGGCATTCCAGTGCTCTCGACAGCCA |
| 19406_2 | 3 | [G/A] | GGGTTTCTCGCTGCATTCACATGCTCCATGTAATGT |
| 19413_3 | 4 | [G/A] | TCTGAAACCACAGCAGAGGCTTGCATATGCTGGCAG |
| 19419_8 | 9 | [G/A] | GTGCAGCTGAAAGCAGCTACATGCATAATGCATCAA |
| 19420_19 | 20 | [C/T] | ATCGCCACCTCAGCATCTCCATGCCCAGACCTGGCC |
| 19431_7 | 8 | [A/C] | CCAGTTCAGACTGCAGCGGAATGCCATGACACCATC |
| 19433_2 | 3 | [T/C] | GTTAGCAGCAAAGCACCCCCATGCCATTACAGTTCC |
| 19440_33 | 34 | [G/A] | CATGACATGTGTGCATTGTGCTGCTGTCAGAAGGTT |
| 19441_17 | 18 | [C/T] | GAGATCAATCCTGCAACCTTCTGCTTAGCAACCTAA |
| 19444_4 | 5 | [C/T] | TGCCCATCATCAGCAACGCCATGCTGATCGGAGACA |
| 19445_31 | 32 | [G/A] | CACTCTATATGTGCATTTCACTGCACGGATGGGTTA |
| 19448_27 | 28 | [C/T] | CTTTCCCATCAGGCATCTGGGTGCTGGCCGAGCGGC |
| 19449_1 | 2 | [T/A] | TTAAAAAGTCCAGCAGCCAGTTGCACAGGTGTCTGC |
| 19451_24 | 25 | [G/A] | CTTACTGACAGCGCAGCACGCTGCGGTCTTTGTCAC |
| 19452_11 | 12 | [A/C] | TCTCTTGCTCCAGCACCAGTTTGCTGAAATGGTTTG |
| 19457_5 | 6 | [T/C] | TTTGCTGCAGATGCATTGTACTGCACAACACTTGTA |
| 19459_3 | 4 | [C/T] | GCACTACCTGCGGCACCACAGTGCCCTGGTAAGTAA |
| 19461_25 | 26 | [G/T] | GCAGAGAGCAGGGCAGTGAGCTGCTGAGTGGCGGTA |
| 19463_8 | 9 | [C/T] | ATGCTACTCAATGCACTCGGCTGCCCTAAAGCCACG |
| 19465_28 | 29 | [T/C] | GATGTCTTACCTGCATAGATCTGCAGCTTTGGCTGG |
| 19472_16 | 17 | [G/A] | ATGTGCATGTGTGCATGGATTTGCTTGCACGTCTGC |
| 19476_25 | 26 | [C/G] | AAGAGTACAGGAGCATATGGATGCCCCAGCAGGGGT |
| 19477_17 | 18 | [G/A] | TCGAAACTCTTTGCAAGGCACTGCAACATGCTCAAA |
| 19480_29 | 30 | [T/A] | TAGGTAGGACATGCAGTGAGGTGCACCGTTAGCCAT |
| 19481_29 | 30 | [G/C] | GACAGTAATGGCGCAGTGATTTGCTCATGGCCACAT |
| 19484_2 | 3 | [A/G] | GCATCTCTTCTGGCAGTGCCATGCAGACACTGGAGA |
| 19489_3 | 4 | [A/C] | ATAACCCATCTAGCATTGTTGTGCGTCTGTGTGGCT |
| 19490_30 | 31 | [G/A] | TTCAGTATCCATGCAGGAAGTTGCCAACACGAGCTT |
| 19491_25 | 26 | [T/C] | GCGTCTGCGTCTGCATGAATGTGCGTGTCTGTGTGC |
| 19492_9 | 10 | [C/T] | AGAGGCCTTCCTGCACTCCACTGCCTGATATATTCT |
| 19494_34 | 35 | [A/G] | CGTGAATGACTTGCATAGCAATGCCCGCATATCGAC |
| 19496_25 | 26 | [G/A] | AATGTGTAACACGCATTAGTGTGCTGAGACCCACAT |
| 19497_18 | 19 | [A/C] | CTCAACTGCAATGCAAAAAGATGCTACTCCAGCTTT |
| 1950_26 | 27 | [G/A] | GATTATAGCCTGGCAGTGTTCTGCATGCCACTGCAA |
| 19504_15 | 16 | [G/A] | CTCAGCAGGCAGGCAGAGGGCTGCTGGGATGACAAC |
| 19505_11 | 12 | [G/T] | TGGAGTATTGGGGCACCATGTTGCCAGTACAAAGAA |
| 1951_10 | 11 | [G/A] | TGGCCACCTCGTGCACATTAATGCACTCCAGCCAAT |
| 19512_26 | 27 | [C/T] | TCATCCCCTGTAGCAGCATGCTGCAACGTGGCCTTG |
| 19514_19 | 20 | [C/T] | CTTTTAATAGCTGCACTCGCTTGCTCGTGTCCTGGG |
| 19519_25 | 26 | [C/T] | CCACCACCTGTTGCAACATTGTGCTCAATCTGCCCT |
| 19526_2 | 3 | [C/G] | ATCAAGTGCATAGCATCTGATTGCGAACAGAAGCCG |
| 19530_29 | 30 | [G/A] | TAAATGTGGGCAGCACAATGGTGCTGTAGGTAGCAG |
| 19534_7 | 8 | [G/A] | GAACAGCGTTCTGCAACAACATGCTTGACCAAGACG |
| 19540_4 | 5 | [A/T] | CTGTAGAGAACTGCAGAGTGGTGCTCCACGGCCCAC |
| 19551_4 | 5 | [T/C] | GCACTACCTGTGGCACCACCGTGCCACCCCGATCTC |
| 19562_25 | 26 | [A/C] | CAGATACGCCATGCACTGTGATGCAATGGTGATGAA |
| 19569_17 | 18 | [G/A] | CCCCACTGGCCTGCAGGGGAGTGCGCCAAGACAGCT |
| 1957_8 | 9 | [C/G] | ACAATGCACTCAGCACCGACGTGCTCAGCAGCCACT |
| 19571_7 | 8 | [C/A] | ACGTGACCAAGAGCACAGCGGTGCAAACCTCACCTG |
| 19572_11 | 12 | [C/T] | TTCTCAGTGTTCGCAGAAACCTGCCAGAGGAAAGGC |
| 19575_6 | 7 | [T/A] | GATAAATTCCATGCAGTGTGCTGCACAGTTTCAGGG |
| 19576_3 | 4 | [C/T] | TTTCAGCGAGCTGCACTTTGCTGCCTGCTCTCATCT |
| 19577_1 | 2 | [G/C] | AGGGTCTCACCGGCACACTCCTGCAAGCTCTGCAGA |
| 19579_25 | 26 | [C/T] | GGGAAAGTGTAGGCACCACAATGCACTGTGAGAAGA |
| 19580_11 | 12 | [C/T] | ACGCTTACAAGCGCACACACATGCATACACGCAAAC |
| 19581_24 | 25 | [C/T] | TTCGGTTGCAGTGCAGGTGACTGCTGTCTTCGTGCT |
| 19582_15 | 16 | [G/A] | TGGAACGTCTCCGCAGTCACCTGCTGGAGATCGAGG |
| 19584_15 | 16 | [C/G] | ACAGGCTAAGACGCACTGTGCTGCCAGAGTGCTGCT |
| 19593_10 | 11 | [C/T] | CTGCTGGCACCAGCACATTTGTGCTCGCTTATGCTG |
| 19595_31 | 32 | [G/A] | AAGGTCCGTGTGGCAGCACTCTGCCTCCTGCACTGG |
| 19602_9 | 10 | [C/T] | GTTTAAATCCGAGCACCTTCCTGCACAGTGGTGAGA |
| 19604_3 | 4 | [T/A] | TCTTAATCCAGCGCAGGAAACTGCAGATAGCACATT |
| 19608_6 | 7 | [A/G] | ACACACACACATGCATGGTTTTGCTTGAGTCCAACA |
| 19613_24 | 25 | [G/A] | TAAACTTTTGGAGCAGAGCAATGCGCTCCTCACCCC |
| 19617_25 | 26 | [A/T] | CTGCCTTGTGTTGCATGTTTGTGCCAAAGGTCTATA |
| 19619_29 | 30 | [T/A] | AGGCAGCAATGTGCAGGGCAGTGCCATAGTAGAAGT |
| 19620_17 | 18 | [G/A] | TAGTCAGAGTAGGCAAGGAGCTGCTCGGACCAGTGC |
| 19621_28 | 29 | [T/A] | GCGTCCTGGCAGGCAGATCCATGCAACCTAACACAG |
| 19623_33 | 34 | [G/A] | TAGATTGCCGCAGCAAATACATGCACTCGCAACGAC |
| 19626_2 | 3 | [C/T] | TACGTTACAATCGCAAGCCGCTGCCAGTGTTATCCA |
| 1963_34 | 35 | [C/A] | GCGTGGAGATTTGCAGCGATCTGCAGTAAATTTTCC |
| 19635_2 | 3 | [G/A] | GTGAGATTGGTAGCATGCTTTTGCATCACAGTGTGA |
| 19644_9 | 10 | [C/G] | TTGGCAAGTCCAGCAATTTCATGCAGAGATGTAATT |
| 19645_31 | 32 | [C/T] | CTGTCCAGAGACGCACAAATTTGCAAACATGCCACA |
| 19647_25 | 26 | [T/C] | TTTCATCTTGTAGCATCCAAGTGCCTACAGTGCATT |
| 19649_24 | 25 | [C/A] | CTTTTATGGCAAGCAGCCGCCTGCAAAATAAGCCTC |
| 19650_15 | 16 | [G/A] | TGACCGCCTGCTGCAGAAGTCTGCGATTCGCACCGA |
| 19656_15 | 16 | [C/T] | ACAATACCCGTGGCACCACCGTGCTGCGTCCGGGTG |
| 19659_28 | 29 | [C/T] | TCAACATCATGTGCAATCACATGCGCTTCAACGAAG |
| 1966_26 | 27 | [A/G] | TGTGTGATTCAGGCAGGACAGTGCCGAGTCGATCCT |
| 19661_27 | 28 | [C/T] | CACACCACCTTAGCAACCAAATGCACACGACATGTA |
| 19667_9 | 10 | [C/T] | CACGGATTCCAGGCATGTCCGTGCATTAATTACAGC |
| 19669_5 | 6 | [C/T] | TTCCCCTTAAAAGCAGTGGCATGCTTTGAAGACATT |
| 19670_34 | 35 | [T/C] | CTGACAACATTAGCATGTTCATGCTTCGCCATTATT |
| 19675_17 | 18 | [A/T] | GCAGACGTGTGTGCAGGACCTTGCGCAGTTTCTGCC |
| 19679_7 | 8 | [G/A] | ATACACCGCCTTGCACATTTCTGCTTTACCCCAGCT |
| 19680_29 | 30 | [T/A] | TGAGTGCAGACAGCATGCTTGTGCTGTACTGTCATG |
| 19683_18 | 19 | [C/A] | AAACCCACACTAGCAACCCACTGCAAAATGCAGACA |
| 19688_15 | 16 | [T/G] | ACACACGCTTTAGCATGTCCCTGCATGCTAGCTTTG |
| 19690_4 | 5 | [G/A] | GGGAGCGGAATGGCACCTCTTTGCGAAATTAGACCC |
| 19692_10 | 11 | [G/T] | TGGACGGGCAGGGCAGGAGAGTGCGGCTCTGCTGAG |
| 19693_27 | 28 | [G/A] | CGTACATGCAGAGCAGTGGAATGCAACGCAGTCTGG |
| 19694_30 | 31 | [T/G] | ACAGGCATATCAGCAGTGCAATGCCGCTGATTGATT |
| 19698_5 | 6 | [C/T] | GCCTTCCTCCAAGCACGCAACTGCATCGAGGAGAGA |
| 19700_34 | 35 | [G/A] | TTCAAAATGTCAGCACTAAAATGCACCTGTGTTCGC |
| 19701_10 | 11 | [T/A] | AAATTTTATATGGCATGTGGTTGCGGGTTGTGCCCC |
| 19702_11 | 12 | [A/G] | GAGAAGATAATAGCAGCTCTCTGCTCCCCCTGTCTG |
| 19709_6 | 7 | [T/C] | AAGCTTCAGTGAGCATCTGGATGCACTGTAAAGGAA |
| 19710_10 | 11 | [A/G] | CTCGCGTGGTACGCATCTCTTTGCCGTGATCATGTG |
| 19716_4 | 5 | [A/G] | GGAGAAAAGTCAGCACCAGTTTGCTTTGTGAGCTTA |
| 19720_31 | 32 | [T/G] | TCGGTTGGACTTGCAAAAGGATGCTCTTGTTTGTCT |
| 19730_26 | 27 | [C/T] | GTGAATGTACCAGCAGAATACTGCAGCGTGGATCTT |
| 19734_3 | 4 | [C/T] | GGCCGAAGCCTGGCAGACGTGTGCGAGGTGTCTGAG |
| 19735_3 | 4 | [G/A] | GCTGATCAAAGAGCATCCCAGTGCAGCGATGATGAG |
| 19737_15 | 16 | [A/G] | ATTTGTTTTTGGGCAAGGACATGCCTGCCCACCACT |
| 19741_25 | 26 | [G/A] | GCACTACCTGCGGCACCACCATGCTGCACTTTTTTC |
| 19743_26 | 27 | [A/G] | CTCTAGGTGCGTGCACAACGTTGCTAACAGTTCAAC |
| 19744_31 | 32 | [C/T] | GGGTTTGTATAGGCATGCCGTTGCTTACTTGCGTTT |
| 19749_29 | 30 | [A/T] | AGTATCAGGGCAGCATGCTCGTGCCACAGTGATCTT |
| 19750_18 | 19 | [C/T] | ACAACCTCTGGAGCACAACGGTGCCACCTTGCTGCC |
| 19753_31 | 32 | [T/A] | CGAAGGAAGGCTGCAAAAGACTGCAAATGCCTTTGG |
| 19756_32 | 33 | [G/A] | GGCATAATCATGGCACAATCATGCCCACTCTGGAGT |
| 19763_3 | 4 | [A/T] | TGTAACAAGTGGGCAGGTAAGTGCTTTGTGGTCTGT |
| 19773_26 | 27 | [A/G] | ATGATCCAAATGGCATGGGGTTGCCGATAGGAAACT |
| 19776_17 | 18 | [A/T] | TGTGCCCTTGTGGCATCAGACTGCCTCCGAGCCAAT |
| 19778_29 | 30 | [G/T] | CACGGTGGCGCTGCAGGGTAGTGCGTGTGGTACACA |
| 19783_32 | 33 | [G/T] | GGTGCCAGATTGGCACCAGGGTGCTGGTGAAGGAGG |
| 1979_1 | 2 | [T/C] | ATACACAGGCATGCACACAGATGCATACTGGAAATA |
| 19790_16 | 17 | [T/C] | TGTCGTTTGTAAGCATTCAGATGCAACAATGCACTT |
| 19792_7 | 8 | [A/G] | TCGCATCGCATTGCAAGGCCCTGCCACGAAATAACC |
| 19798_19 | 20 | [T/C] | GACACGGCTAAGGCACTCATGTGCACAGCACTTACC |
| 1980_5 | 6 | [G/A] | ACTCTGAGAAGGGCAAGAACATGCTTTTTGGGCTGT |
| 19803_26 | 27 | [C/G] | GCACTAATCATTGCACCATCGTGCTGCCAGAAAAAA |
| 19804_15 | 16 | [G/A] | CAGGTCCTTCCTGCAGAGAGGTGCATCAACCCCGAA |
| 19806_28 | 29 | [T/C] | GTGCTAGGACGAGCAGGTGAGTGCAGCTTGGCTGGG |
| 19809_20 | 21 | [G/T] | GGACCTTTCCGAGCATTGGTGTGCCAAGTTTTATGC |
| 19810_17 | 18 | [C/G] | TGAAAAAGATGTGCACCCTGATGCATTGGATGTCTA |
| 19811_25 | 26 | [G/A] | TTTCCTGCACTGGCAATATGGTGCCGAAGCTTACCT |
| 19813_8 | 9 | [C/G] | TCCACCACCAGCGCATTATCATGCAGCGATGCTCCC |
| 19814_31 | 32 | [C/T] | ACAGGAGGAGACGCATCGATCTGCCGCTGGACGGAA |
| 19815_2 | 3 | [G/A] | GTGAGAAAAATGGCATCCACATGCTCCTTCTCAGCA |
| 19816_1 | 2 | [A/G] | AAGTAACAACTGGCAGTGGTCTGCCAATTATAGTTG |
| 19821_24 | 25 | [A/G] | ATGACCTCAAGCGCAGCATGCTGCAGAGTAAGAGGA |
| 19830_29 | 30 | [G/T] | CCTGGTCTCGCAGCAGCTTCCTGCCAGCAGAGATGC |
| 19834_32 | 33 | [C/G] | GCACTTAGCACCGCACTCACATGCTGTGTATCCGGA |
| 19835_25 | 26 | [A/G] | TGCTCGTTCACAGCAAATTAGTGCGAGTTTAATCAA |
| 19836_33 | 34 | [G/A] | TGTTAACTTCATGCATGTCAGTGCCTGAGGGTGGAG |
| 19845_4 | 5 | [G/C] | CACTGTGCATGAGCAAGAGCTTGCTTCTCATGCATA |
| 19846_24 | 25 | [C/T] | GAGTTCCACTCAGCAGGCTTATGCCTAACTCTTTAA |
| 19848_18 | 19 | [C/T] | ATTTATGGGGCAGCATGACGGTGCTGCAGGTTACAC |
| 19852_24 | 25 | [G/A] | AAATATACATCGGCACAGACGTGCGTAAGGTACATA |
| 19857_15 | 16 | [C/A] | CTGCTAGCTGCAGCACCACCATGCTACCTAGGTTTG |
| 19863_33 | 34 | [C/T] | ACACATTCACTTGCACCTGACTGCAGGTCTTTGCCA |
| 19865_29 | 30 | [T/C] | CAGACTGCACAGGCACGGGCTTGCTATATTCTCTCC |
| 19866_17 | 18 | [C/T] | GCATGCAGGAATGCACACATGTGCATGTTCAAGGTT |
| 19869_10 | 11 | [G/A] | CTAAGATGCTGAGCAGGGGGCTGCGCTGCTAGACCA |
| 19871_6 | 7 | [G/C] | CAGGCGGTGAAGGCATGTGTTTGCAAGTACACTGTA |
| 19872_27 | 28 | [C/A] | GATGTTCTGATGGCAGCAGTTTGCAAACCTCCACAT |
| 19874_4 | 5 | [G/A] | GGATGGCGTAATGCAGCCTGATGCCACAGTGGAGAT |
| 19876_7 | 8 | [T/C] | GGTTACTTTCAGGCATGGAGATGCAGCCGGCGTTTA |
| 19877_7 | 8 | [C/T] | AGAACCACCTGCGCAAACAGCTGCCAAAGCAGTTCG |
| 19878_16 | 17 | [T/C] | CTTTTTCGTGGGGCATTGTGATGCCGGAAACTGAGG |
| 1988_10 | 11 | [C/T] | TCAGTCTTTTCAGCAGCCATGTGCTTCTCTAAACGA |
| 19882_1 | 2 | [G/T] | TGCCGTCTCTTGGCAACTGTGTGCGCCTTCCTCGAC |
| 19884_5 | 6 | [A/T] | AAGAAATCTCAGGCATTAAGTTGCCGTTGGGGAAGG |
| 1989_17 | 18 | [G/A] | GCCACCACATTGGCAGCGTTCTGCTTCATTCAATCC |
| 19893_28 | 29 | [G/A] | CACACTGGGGCTGCACTGTGTTGCTCTGGAATGGAA |
| 19896_5 | 6 | [G/A] | GTGCTGAGCTCTGCACTTAATTGCACAGTTTTCCAA |
| 19897_10 | 11 | [C/T] | AGCATCGCACCGGCATTCCTTTGCACGAAAAGAAGA |
| 19908_1 | 2 | [G/C] | CGATCACTGGCAGCACCGGACTGCCACTAATGGCCC |
| 19909_7 | 8 | [C/T] | TGTCTGGCGCTTGCAGCTGGGTGCGGATGGTCCGAC |
| 19911_34 | 35 | [T/C] | CTGTCCATAATGGCATTCTCATGCCCTCACCATTTA |
| 19912_2 | 3 | [T/C] | CTTACAAAACTAGCATGAGGCTGCCTGTTTGTCCCA |
| 19913_7 | 8 | [A/T] | TATTATTATGTGGCACGACCATGCCAGATGCATAAA |
| 19917_29 | 30 | [A/T] | ATTCAGCAGCAGGCAAGTGATTGCATTGCTGTGTGT |
| 19929_3 | 4 | [A/G] | GGAACAGCTGCTGCATGCAGATGCCTGTGTGAAACT |
| 19930_16 | 17 | [G/A] | GCTTACAGGTATGCACGGGTCTGCGATGTGATCGTG |
| 19937_7 | 8 | [G/A] | CCCAAGCGCACTGCATTGGGTTGCAGATTGGGGCGA |
| 19939_19 | 20 | [C/G] | TGTCTGTTCTCTGCACTGACCTGCATTTCCCAGAAA |
| 19941_1 | 2 | [G/A] | CGTGGGTCAGCGGCAACTTGATGCGTCTGACAAGCT |
| 19944_17 | 18 | [C/T] | GTTCTGGCATCTGCAGTCAGCTGCATTAAGGTTCAT |
| 19945_29 | 30 | [C/A] | GGGCGCTTTTCGGCAACGCAATGCAAAAGCTTTTCT |
| 19946_11 | 12 | [C/T] | ATCTGCACATTCGCACATACCTGCTTGAATCCACCT |
| 19947_28 | 29 | [G/A] | AAAGATGCCTTCGCAGGGTCATGCAAGAGTATTAGC |
| 19956_34 | 35 | [C/T] | TAGGACATTAAAGCACGTGGATGCAGTGTGTCCTCC |
| 19965_5 | 6 | [A/G] | CTGAAAAATTCTGCATGCTCGTGCAGCGTCACATAC |
| 19966_31 | 32 | [T/C] | GTACATGAACGCGCATGCGGGTGCCCGTGTGTGTGT |
| 19969_16 | 17 | [G/A] | TGGACAGGAACGGCACGGGGGTGCAGCTGGATCCGG |
| 19970_3 | 4 | [G/T] | GCTTCGAGGCTTGCATTGGTATGCAAGCGCCGCTCT |
| 19975_17 | 18 | [A/G] | GTGTTCATGATGGCATAACCTTGCATGTCCAGGGAG |
| 19976_15 | 16 | [T/C] | CGCCGCCAACACGCATCGATGTGCCCGGAACTTGAC |
| 19979_10 | 11 | [C/T] | TTCCTGAGGCCGGCATGCTCCTGCTCCAGTCCTGAA |
| 19984_2 | 3 | [C/T] | ATCCTTTCTCATGCAACACAATGCGATGCAACACAG |
| 19987_25 | 26 | [G/A] | CAGGTGGGTGGTGCAAGAGGCTGCTGCATTTACATT |
| 1999_16 | 17 | [C/T] | CAGTGCAGCATGGCAGCTTTCTGCTGTGAGGTTAGC |
| 19995_4 | 5 | [A/G] | TTCCATAACTGAGCAGGTGTGTGCAAGTCTTACATT |
| 19998_28 | 29 | [A/T] | TCGCCTGTCCGAGCAGGTCGATGCATTTAAACGCGA |
| 20000_31 | 32 | [A/G] | TGGGGTCTTTCTGCAGCCTGTTGCAATGACAGTTTA |
| 20008_17 | 18 | [A/T] | GTCTCTTTAGGAGCAGGAAAATGCACCATCAGACGC |
| 20014_18 | 19 | [C/A] | TTTGGCATGGACGCAGGTCACTGCAGGTGTGTAGGC |
| 20015_29 | 30 | [G/C] | GTGCTCAGGGTGGCACAGTGATGCCACAGGTAGCAT |
| 20017_7 | 8 | [C/A] | GCACTACCTGTGGCACCACCATGCCGCCCATCTGGT |
| 20020_8 | 9 | [A/G] | AATCATTTAGCCGCAGCGCAATGCATTAAAACAGGG |
| 20021_1 | 2 | [C/T] | CTCCTCTGTCCTGCATTATCCTGCTGCCGCCACACT |
| 20023_11 | 12 | [A/T] | TAAAGTGCTGCAGCACTGAAGTGCAATTGCGTAATT |
| 20026_24 | 25 | [G/A] | TGTGTGTTTGGAGCAGATCAATGCGGCAGTAGCTTC |
| 20028_25 | 26 | [T/C] | ACATGTGCACATGCACGTTTGTGCGTGTGTGTGTGT |
| 20031_11 | 12 | [T/C] | GCCTCAGAAATTGCAGGTCAATGCCACACACAGTTC |
| 20037_34 | 35 | [A/C] | TAGAAACCTAGTGCAAACAGATGCTCAAGGCACAAA |
| 2004_29 | 30 | [A/G] | CTCCTCTGCACTGCACATGCATGCACAATATCTTCT |
| 20049_15 | 16 | [C/G] | CTCAATAATGAAGCACTGCGATGCGTCAGGCTTTCT |
| 20053_1 | 2 | [T/C] | CTTGTGTGTTCTGCAGTGATTTGCAGGGTTCAACAG |
| 20055_19 | 20 | [G/A] | TGGATTTGCGAAGCACGGCATTGCGTTAAAACGACT |
| 20059_26 | 27 | [G/T] | AGTGTATTTTCTGCAGTCGGTTGCTAGGAAACGGCC |
| 20062_3 | 4 | [A/T] | GTTAACTTGGCTGCATGTCTTTGCACTGTGGTAGGA |
| 20065_11 | 12 | [A/T] | AATCATGTGGCAGCAGTGCAGTGCATAAACTCAGGC |
| 20069_29 | 30 | [G/A] | TAGACGAAAGCTGCAAAAACCTGCAAATGGAAAACA |
| 20070_3 | 4 | [C/T] | TTTCTGATCGTTGCACTGCTCTGCCTCTGTGGCTGA |
| 20071_34 | 35 | [A/G] | GGGTGCAGTAAAGCACACGCATGCACACACAAGTAA |
| 20072_25 | 26 | [C/T] | CCACTGCTCCAGGCATGTGTGTGCTCACTTCAATTG |
| 20073_18 | 19 | [T/A] | TCGTAAATGACTGCAGAGTCCTGCTGAAAGAAATGA |
| 20077_10 | 11 | [C/T] | CTAACACACTCAGCACTTCGGTGCCCAACACCTATT |
| 20079_7 | 8 | [G/A] | ACCCACAGCACAGCAAGCCATTGCATAGACTTCCAG |
| 20080_20 | 21 | [C/T] | CTGGCCAACTCTGCAACTCTCTGCTCTGCAATTTCT |
| 20089_17 | 18 | [C/T] | ATGACTTTCTTTGCAACCGCTTGCAACATTTTCTCA |
| 20092_25 | 26 | [G/A] | AAAAATAGGCCAGCAGGGGTATGCAGAGGGTAACTC |
| 20097_33 | 34 | [C/T] | CCCTGCTGCGGAGCATGATGTTGCACCATGACTCGA |
| 20099_3 | 4 | [A/T] | GCTATTTATACAGCAGGATGGTGCAGGTGTGACCTG |
| 201_10 | 11 | [C/T] | TGACCCAGCCCTGCATGTGCATGCTGTTACACGCCT |
| 2010_16 | 17 | [G/A] | CAAGGAGCTTTAGCACGGAAATGCTGATTGCTGCTG |
| 20105_11 | 12 | [C/T] | ACACACACACACGCAAAACTGTGCTCGAAATCTCGC |
| 20112_31 | 32 | [T/A] | TGTAGCACAGCTGCACAGAAGTGCTGAATACTGTGG |
| 20113_31 | 32 | [G/A] | CTGTCGGATTGTGCATTGTGCTGCTGCTGCCGCAAC |
| 20117_29 | 30 | [T/G] | CATGTGGGGAGAGCAAAGCTGTGCTTCTGTTCAAGA |
| 20123_30 | 31 | [T/G] | CAATCCTGTCATGCAACATGTTGCTTGTTGTCTGTT |
| 20126_10 | 11 | [C/A] | CAGCGGCAGCCCGCAGGCCTGTGCCTGCGACAGTGG |
| 20131_20 | 21 | [C/A] | GGACATTTTATGGCAGCAGGCTGCTGTAAGGCTCCC |
| 20132_7 | 8 | [T/C] | CTATATGTCCTTGCAGGGACGTGCTGAGATGATAGC |
| 20134_20 | 21 | [A/C] | TTCAGCAATTCTGCAGCAGCATGCTGAGGGGAATCA |
| 20135_1 | 2 | [C/A] | GCAACACAAAAGGCAACACTCTGCAGCTCGGAGCGA |
| 20139_24 | 25 | [G/A] | TGAAGATCAACAGCAACTGCTTGCGCTCAGTGCCCG |
| 2014_11 | 12 | [T/C] | ACAGCGTTCACTGCAAAGGCATGCACAGAATTCAAT |
| 20142_28 | 29 | [C/A] | ACTGCTGTGGGTGCATTGTGTTGCATTACATGGATT |
| 20143_26 | 27 | [C/T] | AAATGCTTTACTGCAGTCTTTTGCAGCCACTACAGA |
| 20147_1 | 2 | [C/T] | TCTCTTTCTCCAGCATGGACGTGCTGTCTAAAGTGT |
| 20148_10 | 11 | [G/T] | ACTGCATTACGGGCATCAATATGCAGCAAGCCTCTT |
| 20149_33 | 34 | [C/A] | ATCTCTGGAACTGCATCGAGCTGCGCATCACATCAG |
| 20153_9 | 10 | [C/T] | TTTGGACTTCGAGCATCAGGCTGCATCACACCACCC |
| 20161_4 | 5 | [T/C] | TCAGTGCTACGGGCAGTGTCCTGCGCAGTGAGATTC |
| 20162_24 | 25 | [G/A] | AGTCACTTGGCAGCACCACAATGCGAAGGTCTGCCA |
| 20163_16 | 17 | [C/T] | TCACACACTCTAGCATCATGATGCCCCTCCCCCATT |
| 20171_6 | 7 | [G/T] | CTCCAGGACTAGGCATCACACTGCAGTGGCAGAGAT |
| 20175_10 | 11 | [A/G] | AAAATAGGCAAAGCAGCTGGATGCAGAGCATCTTAG |
| 20177_34 | 35 | [C/G] | TCACCGAAAGTAGCAAGCTGCTGCCAGGACGTCACT |
| 2018_7 | 8 | [G/A] | TGGCAACGCTCTGCAAATCTGTGCATGCACGCACAC |
| 20180_15 | 16 | [G/C] | CCATCACCAGGTGCAGTGAGGTGCTGTAAGAGGACA |
| 20184_28 | 29 | [A/G] | GCGTTCACTGAAGCACAGGGTTGCGTCAGATCGTGC |
| 20185_3 | 4 | [G/A] | CTGGAATTTACAGCAGCTTTTTGCCCCGAAAATCCA |
| 20189_2 | 3 | [G/C] | TTGAGAGACCTGGCAATGGACTGCAGGTAGTAGATC |
| 20190_11 | 12 | [C/T] | CATGCCTAAGCCGCAGTAGCCTGCACAGGTTAATAC |
| 20193_5 | 6 | [T/C] | ATGCCTACAGAGGCATAGGCCTGCTGGTTAAAGCGC |
| 20195_8 | 9 | [G/A] | ATCTCTGCGGTAGCATGAAACTGCTGAAAGCTTATT |
| 20196_29 | 30 | [C/T] | ACCAGGGCCAGTGCAGCACCATGCCACCACTGATAC |
| 20198_17 | 18 | [G/A] | GCGGCTGCTGTGGCACTAGAATGCAACGACCATGAG |
| 20199_28 | 29 | [C/T] | CGAAAGGCATCAGCAGGTTACTGCTGCGCTACAACC |
| 20201_9 | 10 | [G/A] | GTACCTGCAACCGCACAGCGCTGCGTCCCAGCAGAG |
| 20203_1 | 2 | [C/G] | CCGCTGATCCACGCAGCCCAGTGCGCTCTGTAATGT |
| 20209_10 | 11 | [G/A] | GAAATGGCTAGCGCAGGCATCTGCCAGGCCTCTCTC |
| 20211_1 | 2 | [C/T] | CCGTGTGTTTATGCATTCCTCTGCTGTCTGTTGATG |
| 20212_19 | 20 | [T/C] | GAGATAGAGGGTGCACAACTATGCATGCTGGCATTG |
| 20214_31 | 32 | [G/A] | CAGGCTCAAAGAGCAAACACCTGCTCCCAAGGAGTG |
| 20222_3 | 4 | [C/T] | CTCCGTGCATATGCAGTGCACTGCAGAGAAAGAGGA |
| 20223_19 | 20 | [C/T] | GCTACTGATGGAGCACTCTCTTGCCAGACCAGCTGT |
| 20224_2 | 3 | [A/G] | AAGCTCTAATTAGCAGCCTGCTGCTTTGTGACACTC |
| 20227_2 | 3 | [C/T] | ACCAGTTAGCTAGCATTAGTGTGCATGCAACCCTCA |
| 20234_26 | 27 | [A/T] | ACAAGCCCCGAAGCAGCAGCATGCTGTAAATGTTGC |
| 20235_34 | 35 | [T/G] | TATGACAGCTCAGCAGAGGTGTGCTGCTTATGTTTA |
| 20236_34 | 35 | [T/C] | AGGTAAAGAAGAGCACGAGTATGCGGTGTCCCAGTC |
| 20238_19 | 20 | [G/A] | TGAGTTATCCTCGCACAATGATGCATTCGGGACACA |
| 20239_29 | 30 | [G/T] | TTTACCTGTTGTGCAGGTGTCTGCAGTGAGATGACA |
| 2025_25 | 26 | [T/C] | TCAGGTTGTTCGGCAAAGATGTGCTTTCATGTGCTG |
| 20255_28 | 29 | [A/T] | CGTATTCCGTTTGCAGGCTTTTGCGTTTAAGGAGCT |
| 20266_30 | 31 | [G/A] | AAAATGTCAATCGCAGGTGATTGCCTCAGGGAGGTA |
| 20269_11 | 12 | [C/T] | GATGAGAGAGGCGCATCATTCTGCTTTACTGCGCTT |
| 20278_10 | 11 | [C/T] | GGTGTTGCTGCAGCAGGCCGCTGCGTGTTCGTTCCT |
| 20286_9 | 10 | [T/C] | TCATAGAGCTCGGCATTATCATGCTGGAACATGTCT |
| 20295_34 | 35 | [A/G] | GCATACTGATCTGCAGCTGTTTGCTGCCACACACAG |
| 20299_24 | 25 | [A/G] | TCCAAATGTTTCGCAGTGGTTTGCAAACACTGTTAT |
| 203_15 | 16 | [C/T] | AGAAGTGTGCGAGCATGACCTTGCAAACACAAAACA |
| 20301_24 | 25 | [C/T] | ACTAAGAGTGTGGCAAGAATGTGCCATCTCCTCTCT |
| 20310_33 | 34 | [C/T] | AGCAGAGAAAAAGCAGCAGGATGCCCTAAAGGACGC |
| 20313_32 | 33 | [G/A] | GTCCTAACAAAGGCATTCATCTGCTGCTGCTGGGTT |
| 20314_16 | 17 | [C/T] | GCAACATGGGTGGCAACACCTTGCCAACCATGCTCA |
| 20319_31 | 32 | [C/T] | TGCTGAAGCCGTGCAGTGTCCTGCTGCAGCACAGGC |
| 20332_18 | 19 | [G/A] | CCAGCTGGACAGGCACTTGAGTGCGCGCGCCTTCAC |
| 20335_27 | 28 | [T/C] | CTTCCTGCAGCGGCATGGTTTTGCATATGGTTTGCT |
| 20337_7 | 8 | [C/T] | GTCAGCCCTGCAGCATGTGTGTGCGCTCCCAGGCCT |
| 20339_20 | 21 | [C/A] | ATAAGTTCACAAGCAAAAGACTGCCAGGAAGCCGGA |
| 20342_30 | 31 | [T/C] | GAACAATCTGCTGCACGGCCCTGCGTTCATTACACA |
| 20344_1 | 2 | [G/A] | TGTGCTGACTCAGCATGGTCTTGCATACCCTTTTCT |
| 20345_11 | 12 | [C/T] | GAACTCTCAGACGCACCCCTGTGCTGACTACAAAGC |
| 20347_2 | 3 | [A/C] | TGACTGAAAACAGCAGTAAACTGCAGCACAGCTTCT |
| 20348_9 | 10 | [A/C] | GAGTGACAGAGTGCACATCGCTGCTGCTGAGATGTT |
| 20356_18 | 19 | [G/A] | CAATATTCTAGTGCACCGGCCTGCTACAGACAGGCG |
| 20361_27 | 28 | [A/C] | GGGCGTTTACCGGCATGAGCGTGCCCCATGTGAGGT |
| 20362_30 | 31 | [C/T] | CACCTGGGTCCTGCACGTGTGTGCTCACATCACTAG |
| 20363_27 | 28 | [G/A] | ATGTATGTAATCGCATATGTCTGCTGAGAGGCGAGA |
| 20374_15 | 16 | [G/C] | ATATGTTTGTGTGCAGACCTTTGCCTTCCACATGAG |
| 20375_31 | 32 | [C/T] | GACCAGCAGGTGGCAGCTTTGTGCCGTTTCTCCAGC |
| 20381_30 | 31 | [G/C] | TTCCTGCTCTGCGCATGATGATGCCAGGTGCGCTCC |
| 20383_1 | 2 | [C/T] | ACACACACACATGCATGCACCTGCTCTCACTAGGCA |
| 20388_26 | 27 | [G/A] | CAGTGTTGCACAGCATTGCATTGCACGTATAACTGC |
| 2039_8 | 9 | [G/A] | GTAACTAAGAAGGCAGGCTGATGCTGTGGTTCTTTT |
| 20391_9 | 10 | [A/G] | TGGGTTTTGATGGCATTTTGGTGCATTTCTCTCTCT |
| 20396_19 | 20 | [T/C] | GCCCACGGCCTAGCAGCGGTGTGCCGATCGACGGGG |
| 20397_17 | 18 | [G/A] | GCATAGGGCAGGGCACCGTGCTGCCCCAATATATGT |
| 20400_29 | 30 | [G/A] | CCATCATCAGGTGCATATTCTTGCCAGCCGCAGGCA |
| 20404_7 | 8 | [C/T] | AAACCCCCACAGGCAGGATGCTGCTGGTGGGATATT |
| 20408_28 | 29 | [G/A] | TTCATTGAATGAGCAATTTCATGCCGCAGACTTCTG |
| 20410_4 | 5 | [A/G] | AATCAGAGAAACGCATCAGGCTGCCAACCAATCAGC |
| 20411_24 | 25 | [T/C] | TGTAGCAGTGCTGCAGTGTGATGCTGCTGTGCTTAC |
| 20412_9 | 10 | [T/A] | TGATGGTGATGTGCATCAACCTGCCAGATGCCCAGA |
| 20414_34 | 35 | [G/A] | ACGTTCGTAAGGGCAGACCTCTGCCATCTGCTGCGC |
| 20415_34 | 35 | [A/T] | AACTGCCTGCCAGCAAGAACATGCCTTAACCTGTAC |
| 20424_32 | 33 | [A/T] | GCGTCCAAGTTAGCACAGATCTGCAAGGCATTATAA |
| 20426_31 | 32 | [G/A] | AACCGCAACAGTGCACACAAGTGCATATCGAGAAAG |
| 20432_19 | 20 | [C/T] | TGACGATGGGTGGCATGTTCCTGCACGTTCCTTTCC |
| 20436_11 | 12 | [G/A] | TGATCTCTACAGGCAGGCTTATGCAGGTGTGTGCCT |
| 20440_25 | 26 | [C/T] | GTACATTTCCTGGCATTCATGTGCTCGACACCTACC |
| 20441_9 | 10 | [C/T] | ACACAGTTTCACGCAGCTGTTTGCCAAACAAGACAA |
| 20442_16 | 17 | [C/T] | CAGTGCTATTGTGCAGCGCTGTGCTCGGTGTTAAAA |
| 20452_30 | 31 | [C/T] | CAAGACAAGCAGGCAATGTTATGCACAGGTCCTTAC |
| 20454_24 | 25 | [G/A] | ATCAGACCCAGTGCATGTAATTGCGTTCTCCTGTGC |
| 20459_29 | 30 | [C/T] | TTTTCACACCTTGCAATTCAGTGCAGGATCTTGCTC |
| 20464_27 | 28 | [G/T] | GCACCCACGTGGGCACTGACATGCAGCGCCGTGTCC |
| 20472_26 | 27 | [G/T] | AAGCAACCCTGTGCAAGTGCCTGCAAGAATATTTGG |
| 20476_24 | 25 | [C/G] | AGAGATTCGTTGGCATCCCGCTGCGTTTGTAATGGA |
| 20478_10 | 11 | [C/A] | ATAAAAGGGGCAGCAGGCTCGTGCTTGAGCTCTCGC |
| 20479_27 | 28 | [G/A] | GGTCTGATGGTGGCAATGCAGTGCAGCGTTTTGCAT |
| 20481_24 | 25 | [T/A] | GGTGTTTGTGGCGCATTGTGTTGCTGGCAGATGTCA |
| 20485_10 | 11 | [G/A] | ATGTCTTTCTGGGCAGATGATTGCCTTGTAATCCCA |
| 20487_4 | 5 | [C/A] | GCGGCAGCCTGAGCATGTCGCTGCCAGTAGAAACAT |
| 20495_20 | 21 | [A/G] | GCCAAATCCATTGCATTGTAATGCTGTGACTCACAG |
| 20502_33 | 34 | [G/A] | TCTCCCCCCTCAGCAGCCTTCTGCCCTAGCACCACC |
| 20503_16 | 17 | [A/G] | AGCTACACGCACGCACACACATGCACAGCTCGCTAG |
| 20506_29 | 30 | [C/A] | ACACACGCGCAAGCACACACATGCTCTCACACACGT |
| 20510_10 | 11 | [G/A] | TTGTCAGCGCGGGCAGCCATTTGCCGAGCTAGTGTG |
| 20511_9 | 10 | [C/T] | ACGGAGCAGCGAGCAGGTTTTTGCCTTTTATCCGCT |
| 20512_8 | 9 | [T/A] | GTGATTTTTGTTGCACCTCTATGCTGTTCTGGCCTG |
| 20513_10 | 11 | [G/T] | TTCAAAGCAAGGGCAATTATTTGCTCAGTAGGCCTC |
| 20514_6 | 7 | [C/A] | TTGCTCCTGGAGGCACATTTCTGCTTTAACACAGCC |
| 20519_11 | 12 | [G/A] | AGACAGAAAATGGCAGCCTCTTGCCTTGCTAAAGAA |
| 20525_34 | 35 | [T/C] | GGGCCATGTCCTGCAAGTCAGTGCATAGTAGTTTCA |
| 20536_2 | 3 | [T/C] | TCTTGGTGCAAGGCAATGACATGCAACATGGTTTAC |
| 20537_7 | 8 | [C/T] | TAACTCGCGGCGGCAGATTATTGCTGTAAACATACC |
| 20538_18 | 19 | [G/A] | TCTTGGACGCCAGCAGAAGGTTGCAGTGATCCATCA |
| 20540_9 | 10 | [G/A] | TCCACCGATGATGCACCACAGTGCCCGCCATGCATG |
| 20542_11 | 12 | [T/C] | CTTGTTTGACTCGCAACAAACTGCACATACTCCCTG |
| 20548_6 | 7 | [G/T] | CTCCTCGCCATGGCAACAGCTTGCTGAATTTACTTG |
| 20552_16 | 17 | [C/T] | GAGTGTCCTGAAGCATCACAATGCCAGTACCAATTA |
| 20555_34 | 35 | [C/T] | CCAGTTTCTCCAGCATCCGGATGCCTTTGGCATTCA |
| 20556_30 | 31 | [C/T] | GATGACCGAAGTGCACAGACCTGCAGCTTTCAGGGA |
| 20559_24 | 25 | [C/T] | ATTCCCTGTGGAGCACCAATGTGCCTGACCACTCTG |
| 20560_17 | 18 | [C/T] | GAATTGGTCATTGCATGCAGGTGCAGGTGCACATGT |
| 20561_4 | 5 | [C/T] | GGTTCAACACAAGCAGGTGTCTGCATTGCAGCAGCT |
| 20564_3 | 4 | [T/C] | CAGTGGTGTGAAGCAAAGAGCTGCAAACATGCAGGA |
| 20565_20 | 21 | [C/T] | GAATTGCATGCTGCAGTCTGCTGCTTTGAAATAGGT |
| 20567_32 | 33 | [A/C] | GGTCACCACACAGCATATTATTGCAACACCGCAGCT |
| 20573_32 | 33 | [G/T] | GAACATAGATGTGCACAATGCTGCCGATGTTAGTTT |
| 20576_5 | 6 | [G/A] | GCAGGGATTCCAGCACATTACTGCACCATCCCCAAC |
| 20577_17 | 18 | [C/G] | TAAACCCACAGGGCACACTGGTGCAATTTCCATTCT |
| 2058_1 | 2 | [C/T] | CCAGATGGGATAGCATGTCACTGCAGAATGCAGTAC |
| 20580_16 | 17 | [G/A] | AAGGTAGCACAGGCATGAGGGTGCCCTAGGATATAA |
| 20581_6 | 7 | [C/T] | TGCTGGCTGTCAGCACAGAAATGCTGCTCATGTTCA |
| 20583_10 | 11 | [G/A] | TGGAGAAGCCGAGCAAGTGTTTGCAATGGCTCATTC |
| 20586_29 | 30 | [A/G] | ATAAGCAACCATGCACTCGCATGCACCGTAACATTT |
| 2059_16 | 17 | [G/T] | CTGCTGCAGGATGCATGAGGCTGCCGGCTGTGCGTT |
| 20591_24 | 25 | [G/A] | TGATTAGAGTCCGCACAGCTCTGCGCATGCATTATA |
| 20597_4 | 5 | [A/C] | AGAAACAACCGAGCAGCCAAATGCCCAATTAGTTCT |
| 20598_10 | 11 | [G/A] | TAACATGAGCGGGCAGTGTGATGCTGATGACAACCC |
| 20600_18 | 19 | [A/G] | AGTTGGTACCCTGCAATTAATTGCTGCCCTGTCCAG |
| 20601_9 | 10 | [C/T] | CCCCGGGTCCCTGCAGTCTTTTGCACAGTAGTGTAA |
| 20602_18 | 19 | [A/G] | CCTTGGCCCCTAGCAGCAGTCTGCCCTTCTCCTCAT |
| 20603_33 | 34 | [C/A] | TCTACACGCATCGCAACGTCCTGCCGAAGACACCGA |
| 20606_3 | 4 | [T/C] | TTCTATTGTCAAGCAGTTGCATGCAAGCCTTGCATC |
| 20608_27 | 28 | [C/A] | CATGACAACTCTGCAGCACATTGCAATCAACAGCAA |
| 20619_31 | 32 | [C/T] | CATATCGTATCCGCAGGTTTTTGCCAATACACAGCC |
| 20621_9 | 10 | [G/C] | ATTAGTACGGCAGCACATCGGTGCATCAGGTAGTAT |
| 20626_24 | 25 | [G/A] | TAGGGACAGAATGCATCCCTGTGCGATCGATTCTGT |
| 20627_29 | 30 | [T/A] | CATGTGGATCCAGCATCCGAGTGCTCGCATTGATGA |
| 20631_5 | 6 | [G/A] | TCAAGGCGCACAGCAGCGCGCTGCAGGCCAAATTCA |
| 20633_10 | 11 | [A/T] | AGTCCATCACATGCAGACACATGCACACAACAAGTA |
| 20634_32 | 33 | [C/T] | GGTTCTTCAGAAGCAGCACACTGCGGGCGGTTTGCC |
| 20637_28 | 29 | [G/A] | ACACAAAAGAGAGCAGATGGGTGCCCACGAGCTTGT |
| 20638_31 | 32 | [G/A] | CACGAGGTCCATGCAGCAGCGTGCTGATGTTGTAGT |
| 20641_28 | 29 | [G/A] | ATTTTTTGAAAAGCAAGCTGATGCCTGTGAGCACTT |
| 20648_32 | 33 | [G/A] | TACTATCGTGCTGCATCTGACTGCATTTTCCCGGCA |
| 2065_8 | 9 | [C/T] | GGATGTCTCTATGCAGCTGTCTGCAAAAGACAGGAT |
| 20652_25 | 26 | [G/A] | GGTGCATCTAAAGCACCAGACTGCAGCACCGGCACG |
| 20653_8 | 9 | [T/A] | GACTAGCTTATGGCAGGTTTTTGCTTGCTCATGGTG |
| 20658_20 | 21 | [T/C] | TTATTATCCGAAGCATCTTATTGCACGTGTTGCACG |
| 20663_4 | 5 | [C/G] | ATGCCCCGTTGTGCAGAACCCTGCTGTGTTGCTCCT |
| 20664_17 | 18 | [C/G] | TGTAGTACACATGCATGCGGATGCAGTGACATCTAA |
| 20667_27 | 28 | [G/A] | GCACCTCTGACAGCACATTGTTGCACAAAGTCTATA |
| 20668_4 | 5 | [T/A] | GACCTGCCCACTGCACTGCACTGCGTGCAAACATAG |
| 2067_24 | 25 | [C/A] | TCTAGTAACTGTGCAAAACTGTGCCGTAGTGCTGAT |
| 20670_17 | 18 | [T/C] | GAAAGGCCAAATGCATTTGACTGCAGGAGAGATGAG |
| 20672_28 | 29 | [G/A] | GACAAACTTCATGCAAAGAGCTGCAAACGCACGCCT |
| 20673_19 | 20 | [C/T] | CACACTCGTACAGCAGGTGCGTGCGCTTCTTAGGGT |
| 20674_25 | 26 | [C/G] | ACATAAACACATGCAAACGGCTGCACGAACATGCAC |
| 20680_29 | 30 | [G/A] | AGATAAACACATGCAGAGCCATGCATTTGGAGCTAT |
| 20681_7 | 8 | [A/G] | CCATACAATCGGGCACGCTGGTGCCCGCTGCCAGGA |
| 20683_20 | 21 | [T/C] | TGGTCGATATTGGCAACCAATTGCTCCCTTTAGAAG |
| 2069_5 | 6 | [C/T] | AGGTTCAGCTGAGCATCTTGGTGCCTCTTTTGCCAT |
| 20691_18 | 19 | [C/A] | CATAACAGCCAGGCAGACACATGCTGGAAAATATGA |
| 20692_11 | 12 | [C/T] | TGAAATCCAGACGCAAACATCTGCATGAGCGGTCTC |
| 20695_18 | 19 | [C/T] | ACAGAGATCAGAGCATCACAGTGCTGCCCTGCTAGG |
| 20696_28 | 29 | [T/C] | CCAGTGCTGTGGGCACCTGGTTGCCCGCTGGGACCA |
| 207_4 | 5 | [A/T] | ACACAACTTGTTGCACCACCATGCTGCCTATTAGTA |
| 20701_18 | 19 | [C/T] | CGGTGTGAGAGGGCATTGCGCTGCGGTGTACCTAAC |
| 20702_2 | 3 | [C/T] | TCCTGGCAGTGAGCACGTAACTGCTGTACTTCTTCA |
| 20704_26 | 27 | [C/T] | GACGGGCAGGTTGCAAGGGGCTGCTTCGAGAAGGAG |
| 20711_25 | 26 | [G/T] | GACTAGGTTGCTGCAAGAAACTGCTGCTGCAGAATA |
| 20712_29 | 30 | [A/C] | GTGTTTGACAGTGCATGTGTATGCCAGGCACTCGTT |
| 20726_19 | 20 | [G/A] | CACAGCGACTCAGCATTACGCTGCTGCCTTGATTAA |
| 20728_15 | 16 | [G/A] | TAACACGACACTGCAGAAAAGTGCAAAATTCCTGAG |
| 20742_18 | 19 | [G/A] | TCAATTCATCCCGCATGGGAATGCCTCATTAACGAT |
| 20743_27 | 28 | [C/T] | ATATGCAATGATGCATGCTGCTGCCTTCTCGGATGG |
| 20744_9 | 10 | [A/G] | CGAGGATCCAGAGCACGTCCGTGCGATCCAGCTGGC |
| 20745_28 | 29 | [C/T] | TTTCACAGATCTGCAATTCCCTGCTGCTCTGCTAAG |
| 20749_1 | 2 | [C/A] | CCTTTCCTTTTTGCACAAAGTTGCTCTTTGGCCGTA |
| 20751_8 | 9 | [A/C] | CAATCTTCACGTGCAAAATGCTGCTTTCATTTGCAT |
| 20754_3 | 4 | [C/T] | TTCCCCTGGAGAGCAGATGCCTGCTTCTTTCTCAAG |
| 20756_29 | 30 | [A/T] | CTGGATGGGATGGCAGACTATTGCAGTGGACCATGC |
| 20759_18 | 19 | [G/A] | ATATTTGACAGAGCATACGTGTGCATGACACGTGTT |
| 20763_33 | 34 | [G/A] | ATTGTAGCTTCAGCAGCCTCCTGCATCCATCCCGGA |
| 20767_24 | 25 | [G/A] | GTGTGTGTGTGTGCAAGTGTTTGCGTGTGTGCGAGC |
| 20768_27 | 28 | [C/T] | GAATCTCCTCCTGCAACCTGATGCAATCAAACCAAA |
| 20774_25 | 26 | [C/T] | GAGATTACCGACGCAGTAATCTGCTCGTCTATCTAT |
| 20776_6 | 7 | [C/T] | TCCATACACAGTGCAGTGTAATGCTCACAACTGCTG |
| 20786_32 | 33 | [C/T] | ACTTTAAGCGACGCATGTGGCTGCAGGAGTTACTGC |
| 20790_7 | 8 | [C/G] | GGTATCTGTTGTGCAAGACGGTGCTTTTCCTCATCT |
| 20795_6 | 7 | [C/T] | TAAACTTAAGTGGCAACCATCTGCAGCTCATATCTG |
| 20798_24 | 25 | [T/C] | GGATAACGTGGAGCATGCGTATGCTGTACAGCAGCT |
| 2080_11 | 12 | [T/A] | CTTTATTACAATGCACAGCACTGCGCTATGACCTGA |
| 20803_8 | 9 | [T/C] | AATTGTTCTAGTGCATTGAATTGCCAGGGCTCCTCT |
| 20804_10 | 11 | [C/T] | CATTAGGTACCGGCATGCTGGTGCAGTGGGCCCTAG |
| 20808_17 | 18 | [G/A] | TCACAACGACAGGCACTGAAATGCTGCTTGAGGACC |
| 20820_27 | 28 | [T/A] | CCAGGACTGGCAGCAAGTCAGTGCACTTAACGAGTT |
| 20821_15 | 16 | [C/G] | AACTGACACACAGCACCGACTTGCCTTTGCATGTTG |
| 20823_20 | 21 | [C/A] | CACACCACCATTGCAACAGACTGCTTTTGAGTGTCT |
| 20824_27 | 28 | [G/A] | ATCTCACACAACGCAACTTTCTGCCCTGCTACTGTA |
| 20826_15 | 16 | [A/G] | ACAGGAGGTGATGCAGAAGCATGCAAGGAGCATAGA |
| 20827_28 | 29 | [C/T] | AATGCATCCCTGGCAATGTTATGCTGCTCAGTCTGT |
| 20828_28 | 29 | [G/T] | TGGGGTTGGGTTGCATAAGGTTGCGATTGAGTTATG |
| 20829_16 | 17 | [G/A] | TCCTAGGTTGGAGCATGAGCGTGCTGTGGTACAGAC |
| 20834_29 | 30 | [C/A] | GAGGGCTTATTAGCAAGGCGTTGCCTTTACCGTTTT |
| 20837_30 | 31 | [G/A] | GTTCATGCCCTGGCAGATCAGTGCTGTTTTGACAGG |
| 20842_16 | 17 | [G/A] | GATGCAAGGATTGCATGGAGCTGCTCGACCATAAAG |
| 20843_1 | 2 | [G/T] | GGACGAGACGTTGCACTATGTTGCATTCCTGCTAAA |
| 20845_8 | 9 | [C/G] | CTGCAATCCAGAGCAGTAATGTGCAAACACATGGTT |
| 20850_30 | 31 | [T/C] | CTGAGGAAACGTGCAGTGCATTGCTATACTTACCAA |
| 20854_19 | 20 | [T/C] | CTGTGGGTCACAGCAGGCCTATGCACTTATTAATGA |
| 20863_16 | 17 | [G/A] | TAAGGTTTCTTGGCACGACGCTGCCACTAAAATATT |
| 20865_7 | 8 | [C/T] | GGTTCCTCCTAAGCAGCAGATTGCCTGAAGAGAGAC |
| 20871_10 | 11 | [C/T] | GGTGAGACAGCTGCAGGTCTTTGCAGGTCCTGGCCG |
| 20875_10 | 11 | [C/T] | TGAATGACAGCGGCATCGCCCTGCCAACGTACACAA |
| 20878_1 | 2 | [G/A] | CGGCTGATGAAAGCATCACGATGCTGGAATAATCCT |
| 20886_29 | 30 | [T/C] | CTATTTCTTCTCGCACTGTAGTGCCGTGGTAGCTCT |
| 20887_28 | 29 | [A/G] | GCGTGAGCAGCGGCAACGCCATGCTGATATTGCAAC |
| 20889_25 | 26 | [G/A] | TTCCCTGCATGAGCACCAAACTGCCGCACTCTTAGG |
| 20896_15 | 16 | [A/G] | ATATGTCCTTGAGCAAGCTGCTGCCTGGTTTAACCT |
| 20907_19 | 20 | [G/A] | AGATATGAGAAGGCAAGCCGCTGCATTCGAATTACT |
| 20909_5 | 6 | [T/A] | CCCCATGCTTTCGCAAAAATATGCCACACAACAAAC |
| 20910_19 | 20 | [G/A] | CCTTGAAGAGCTGCACTCCGATGCATGCAAACATGA |
| 20912_29 | 30 | [G/T] | GGGTAATAGTGTGCATGAAGCTGCCAGTGGCTCATG |
| 20917_8 | 9 | [T/A] | GTCCTCCCTGGCGCAGCACTTTGCCAAATGAGTTGA |
| 20920_2 | 3 | [G/A] | CAGAAGAGTTAAGCAACAACATGCTAAGGAAGCCCA |
| 20925_10 | 11 | [A/T] | CAGTGGTGCTAAGCAGTAAATTGCTCAACATCCTAA |
| 20929_15 | 16 | [G/C] | GCTCATAAAATGGCAGAGTGCTGCTGTCTGCAGTTA |
| 2093_32 | 33 | [G/C] | ATCAGAATGGTGGCACAATGGTGCAGATAAGTGTGC |
| 20930_27 | 28 | [C/T] | AGGATGTCGAGTGCACTCCTGTGCTCTCCTGTCTAC |
| 20933_7 | 8 | [C/T] | ATCTATCTGCAAGCAGCTAATTGCCAAAAGCTGCTA |
| 20937_17 | 18 | [T/A] | TGGATAGGCTTTGCAGATGTGTGCACACTGAAGAAC |
| 20943_10 | 11 | [T/C] | TACACTGCAATGGCAGGGCCCTGCACCTTTTCAGAT |
| 20944_31 | 32 | [A/G] | TCAAACACACATGCAAACCTATGCACACCCAAACAG |
| 20951_20 | 21 | [C/A] | TGCATATAAGTCGCATTGCCCTGCGAGACCTGCGGC |
| 20952_24 | 25 | [G/T] | AATAATGTGTCAGCAGCTACTTGCGTTCGAGATGGA |
| 20953_10 | 11 | [C/G] | ACCCCAAGACCAGCATTAAAATGCTACACAGGCGGC |
| 2096_28 | 29 | [T/A] | CAAAAGTGAACAGCAGACCATTGCTCTGTCTCAGGA |
| 20960_10 | 11 | [A/G] | GATGAAACACATGCAGAACCCTGCAGCAAAAGACCT |
| 20963_20 | 21 | [G/A] | TGCACATGTTGTGCATAGTCGTGCAGTAGAGTGCAT |
| 20966_33 | 34 | [C/T] | GGTAGAGTACAAGCAAGAACTTGCTGTCCGCTTCAA |
| 20973_10 | 11 | [G/A] | GAGAGAGAGAGAGCAGATCTGTGCAGAGACACCTGA |
| 20980_25 | 26 | [C/T] | CTATGCTCTGATGCACAGTGTTGCACTGCTGCATGT |
| 20981_10 | 11 | [A/G] | TTAAAGCTGTAGGCAGGAGTGTGCGTGTGGTCAGTG |
| 20988_17 | 18 | [T/C] | ATGCGTATGATGGCACGTTAATGCACATGTTCTGGG |
| 2099_30 | 31 | [A/G] | TGGGCAAGGCTAGCACCCATATGCTGCACGGAGACA |
| 20991_24 | 25 | [G/A] | GTTTCGTGCGTTGCATCAGGCTGCGGCTAAATTCCT |
| 2100_24 | 25 | [C/A] | CAATGTTTACCAGCACAACATTGCCCAGAGCATCAC |
| 21001_3 | 4 | [C/T] | TTCCAGGTGAGGGCAGTAGGGTGCAATGTGAGCACT |
| 21007_19 | 20 | [C/T] | TGTGTCAGCAAAGCAAATCCGTGCGTGTGCAGGAGG |
| 21011_26 | 27 | [G/A] | GTGTGTGTGGAAGCACGACATTGCTTGACCTCGGCC |
| 21015_19 | 20 | [C/A] | CATGACTTCTGGGCATGTCCCTGCCTTATGTTGCAC |
| 21016_6 | 7 | [A/C] | TCTAGAAATCCAGCATCATGATGCTTCTGAAGCTCA |
| 21018_31 | 32 | [G/A] | ACGAATCGATGTGCACCGGAGTGCCGACCTCGACCT |
| 21019_31 | 32 | [A/G] | TGATGATGATGAGCATAGGAGTGCCTGAGGGAAAAG |
| 21021_8 | 9 | [T/C] | TATATTGATAGTGCAGACTGGTGCAGGAGGAGGTGA |
| 21025_17 | 18 | [G/A] | TATGATTATCTGGCAGTGAAGTGCTGGTCATGCTTT |
| 21028_10 | 11 | [G/T] | TGGGAATCCTGTGCAGTGCTCTGCAGAAGATGGAGT |
| 21030_32 | 33 | [G/A] | GACCGAGCTTCTGCAGCTGAGTGCGGAGGGCTGAAG |
| 21032_1 | 2 | [C/T] | GCGCCTTCACCTGCAGGTTGCTGCTACATTTGTTAT |
| 21034_6 | 7 | [A/G] | CTCAGCAGTGCTGCATGAGGATGCGAGTTTAACGCT |
| 21035_32 | 33 | [C/T] | ATCGGAGACGCCGCACGGACCTGCCAGCTTACCGGA |
| 21044_28 | 29 | [C/T] | ATCTGTGAACTGGCAGGTCTATGCTTCACTTATGTG |
| 21054_4 | 5 | [G/A] | TCCCGCCTGTCAGCAGCAGCGTGCCGCCTGAATCCG |
| 21057_32 | 33 | [T/A] | TGGACTGCTTCAGCACGTCACTGCAACAAGGATGTG |
| 21063_16 | 17 | [A/G] | AGACCGGAAGACGCACAGCTTTGCCACACGGCTTTG |
| 21074_29 | 30 | [T/G] | AAATGACCGGTAGCAAGGCACTGCAATTCTTTTTTT |
| 21077_3 | 4 | [G/A] | CTAGCATGTGTGGCATGGTGGTGCCTCAGGTAGTGT |
| 21079_8 | 9 | [C/T] | GTCAGGCTCACAGCAGACGGCTGCTGCACACTACCT |
| 21087_25 | 26 | [G/A] | TATAACAATTGGGCAAGACCATGCAGACAGCCTTTT |
| 21089_29 | 30 | [A/G] | CTTAAGAGGTTTGCAGTAGAATGCACCTGGATGCAA |
| 2109_6 | 7 | [C/T] | GTAGCTCACTGAGCACACAAGTGCTGTATAAAGCAA |
| 21091_33 | 34 | [G/C] | TCCTGCTCGTCTGCAGCAGGCTGCTCTTCCTCTGCT |
| 21092_15 | 16 | [A/T] | GACTGCCAGTCGGCAAAGTCGTGCCTGTTGTCTTAT |
| 211_1 | 2 | [A/G] | CATGTCAAATGAGCAAAACGGTGCTGTGAATGAACT |
| 21108_24 | 25 | [C/T] | GGATTCCCACCAGCACTTCCCTGCCTTCCATCTTCT |
| 2111_16 | 17 | [T/G] | ATGTGTGTATTGGCATTCGTGTGCTTCTATTGTCAT |
| 21112_27 | 28 | [A/C] | TTGGTGAATCTGGCAACCCACTGCTAAAGTGTTAGC |
| 21122_20 | 21 | [G/T] | CGTGCTGAGAGTGCATGCGTGTGCTGCAGAGCTACA |
| 21123_8 | 9 | [C/G] | TTACTTAACCATGCACACAAGTGCACACACACACAC |
| 21131_5 | 6 | [C/T] | GGTCTCGACATCGCAGCTTGGTGCTATCAAATTATT |
| 21137_17 | 18 | [C/T] | GTGTTGCCAGCAGCATTCAAATGCAGCTGTTTGAAG |
| 21138_10 | 11 | [G/A] | ACACAGCCTCGAGCAGCTTGGTGCTGGCAGTCGCTG |
| 2114_18 | 19 | [C/T] | GGACATGGCGGTGCACAGCGTTGCTACACACTTTCC |
| 21141_10 | 11 | [A/C] | ACTGATGGGTATGCAGGTGAATGCTAGCCTGTCTGT |
| 21144_18 | 19 | [C/T] | CCGCCTGCACCTGCACCACGCTGCAAACGGCCCACA |
| 21146_3 | 4 | [G/A] | AATGCAGAAAGTGCACAGTGTTGCATGGAATGCACC |
| 21151_28 | 29 | [C/T] | ACTGGCACATAAGCATTTCAGTGCCCACCACTTCAA |
| 21153_17 | 18 | [C/T] | TCAGCGGAACCTGCAGCCGACTGCGAAAGAGATAAA |
| 21155_11 | 12 | [C/T] | GAAGTGTGAGACGCAGACGAGTGCTGGAACGACAGA |
| 21161_15 | 16 | [G/A] | TGTAGTAATGGGGCAGTTTGGTGCCCAGCTGCCTGA |
| 21162_18 | 19 | [C/T] | ACGAGGAACATGGCAGTTCAGTGCACAGAACCAAGA |
| 21163_2 | 3 | [C/T] | CTCTCTCTCTGTGCATGTGTGTGCCTCTCTCTCTCT |
| 21164_31 | 32 | [C/T] | TGCTTATCATCAGCAGGCAGCTGCTGCCGTGCGTTT |
| 21166_4 | 5 | [T/C] | AACATCCCCACAGCATGAAGCTGCCTTCCACTGGGC |
| 21167_26 | 27 | [A/G] | TTTGTAACACGTGCATTATCATGCCAAGGCAAAGGG |
| 2117_18 | 19 | [C/T] | AATGCCCTTAAAGCAACCCGATGCATTAAGCAACCG |
| 21170_15 | 16 | [T/C] | GGGATGTGGGCAGCATGCTTTTGCACAAGCTATGTT |
| 21177_11 | 12 | [T/C] | AATACATTCCCTGCAGAGCCGTGCCAATTTCAGCTT |
| 21178_25 | 26 | [C/G] | AGGCGTGACCACGCAAACTTTTGCTCTCATCTGTAG |
| 21183_19 | 20 | [G/A] | ACTGGTGTCAATGCATGCTGCTGCAACATGTGCAAA |
| 21184_18 | 19 | [T/C] | CAGATGGACCCTGCAGTCTTCTGCAGAAGGCAGGTG |
| 21185_9 | 10 | [G/A] | TGCTGGCTGGGGGCAGGGTCCTGCGTTCACACACGC |
| 2119_2 | 3 | [A/T] | TGAAAAAAAGGGGCAGTCGTATGCTTGCGGTTAAGG |
| 21196_32 | 33 | [G/A] | CGCCTGCAGAGGGCAACTTGCTGCGCCACGCTGCTC |
| 21203_28 | 29 | [A/T] | GGTTAAATAAAGGCAGAGTGGTGCCGCTATGCCAAC |
| 21204_31 | 32 | [C/T] | GATTCACGCTTTGCAGTTTGCTGCGGTTTCTCAGTT |
| 21205_28 | 29 | [C/T] | TGTGCAGACACAGCACAGGAATGCTTCCCTTTTTCC |
| 2121_31 | 32 | [C/T] | CTGAGGGATCCTGCACACATCTGCACCATGTCAGCT |
| 21213_30 | 31 | [G/A] | GCGTTTAGACGGGCACCAGGCTGCGGACACGCTGCA |
| 21214_30 | 31 | [G/A] | GCATGAGTTTATGCATCGCGCTGCTGCCACATGATT |
| 21217_31 | 32 | [G/A] | CATGTCTGCCGGGCAGGTTGGTGCCTGTAATGATGC |
| 21219_30 | 31 | [C/T] | CAGCAGGGTTGGGCAGATTTGTGCGCTCTTCAAAGT |
| 21222_32 | 33 | [A/T] | GGAGGTTTGGCTGCACCGGATTGCTCCAGTTTACTT |
| 21223_27 | 28 | [G/A] | ACCGAACCCGGTGCACACAGCTGCTCTGAGGAATTG |
| 21230_34 | 35 | [A/T] | CCTGTGACTGAGGCAGAGAGCTGCCACTCTGTTCAC |
| 21231_34 | 35 | [A/G] | AGGTTTTGGGGTGCATCTCTCTGCAGCAGAGGCTAA |
| 21233_11 | 12 | [C/T] | AATCACATGGCCGCAACTCGCTGCAATTAGGCATGC |
| 21234_4 | 5 | [G/A] | CAAGGAAGCCCAGCAAAACATTGCTTTCGACTGCTT |
| 21236_6 | 7 | [G/A] | AGTCTGGAGATGGCATTCTGCTGCCTGCAAACATTA |
| 21237_7 | 8 | [A/G] | GGTGAGGAGACGGCATGAGTCTGCGTCCTTCACTGC |
| 21238_29 | 30 | [G/A] | CTGACACACCGAGCATCATCCTGCACCGCGGAGACT |
| 21243_24 | 25 | [C/T] | ACCTGAGCCGCAGCAGTGGTGTGCCGCTAATCGCTA |
| 2125_34 | 35 | [C/T] | GTATAAGTGAGAGCAAGATGCTGCCACCGGCCTGCA |
| 21251_10 | 11 | [T/C] | CAGAGGTGACTTGCATATTTGTGCAGGATGTCAACA |
| 21255_10 | 11 | [G/T] | AACTGCAGAGGGGCAAGGGCATGCCCAGGTAAGTGG |
| 21256_17 | 18 | [T/G] | GCAGCCTCACAAGCAGATTGATGCATGAGGTCATAC |
| 21264_29 | 30 | [G/A] | TCAGTATCGATCGCAGTTAATTGCAGTACGATGCAC |
| 21269_25 | 26 | [A/G] | TTTGCCCCAACGGCAGCAACGTGCTAAGAGAATTAA |
| 21270_7 | 8 | [C/T] | TGCAGAACGCTCGCAACTGTGTGCTGGCCAGTCTGG |
| 21284_3 | 4 | [G/A] | GATGGGAAGCAGGCAGGGTGCTGCAGGCTGTATCTT |
| 21291_18 | 19 | [C/A] | AAAAAGGTAAAAGCACACCGATGCAGCTGGGAAACC |
| 21295_24 | 25 | [A/T] | GTGGAGCAAAATGCAAACACATGCAACACAACACAT |
| 21296_28 | 29 | [G/A] | GAAGCTGTTCCAGCATGGCACTGCACCTGTGCACAA |
| 21297_16 | 17 | [G/A] | TAACCCTATGTGGCACGGTGTTGCCTTCAGGCAACA |
| 21299_1 | 2 | [G/A] | AGCCAGCAACTGGCAATCGGCTGCTTTTTTGCTGGC |
| 21305_31 | 32 | [T/C] | ATCTCCATTACAGCAGGACTTTGCGTGCGTTTCCAG |
| 21306_6 | 7 | [A/G] | AGCAGCACCATAGCATGATACTGCCACCACCATGCT |
| 2131_34 | 35 | [T/C] | CCGTGATAGCTGGCACGCAGGTGCCACACCACACTC |
| 21311_16 | 17 | [G/A] | CGCTTCGATTGTGCACGCAAATGCATCCGTTCGTGT |
| 21318_25 | 26 | [A/T] | CGGTGAGCTGCAGCACCAGCCTGCGAGGAGCGGAAA |
| 21327_30 | 31 | [A/C] | TGGGTTAGCATGGCACACCTATGCACGTCCACACAC |
| 21336_24 | 25 | [G/A] | GATTATTTCTGGGCAAAATTTTGCGGCTGGCGGGCA |
| 21339_6 | 7 | [C/T] | TTATGTCCAGCTGCAATCGGCTGCAAGCATCTGTTA |
| 21341_10 | 11 | [A/T] | ACTGATAGTAAGGCACTTGAATGCTGAAGGAACGAC |
| 21344_4 | 5 | [C/G] | TTTGCCCGTTGCGCACTTTTTTGCCCCCGTGATCAC |
| 2135_20 | 21 | [T/C] | ACCAGAAATGCAGCACAAACTTGCAAGCTTCAATGA |
| 21363_32 | 33 | [C/G] | GCTGATTGTGTGGCATGGTGGTGCAGTAAGCTGCTA |
| 21364_19 | 20 | [C/T] | ACAACAAACACTGCACCACCGTGCCACTGTTATTTA |
| 21365_4 | 5 | [A/C] | CAGCAGATGCATGCACACAAATGCAGAAACATGTTG |
| 21373_17 | 18 | [G/T] | GACTTACTCAGAGCATCGGCCTGCATCGTTATTATT |
| 21385_6 | 7 | [C/T] | GAGACTCCAAAAGCATGCTGCTGCTACAGAAAATTG |
| 21387_27 | 28 | [T/G] | CACCTAGCTAATGCAACAGCCTGCTTTTGTGGAAAC |
| 21390_27 | 28 | [A/T] | CAGCTAAACTTAGCATGCGCTTGCACAATCATCCGC |
| 21391_17 | 18 | [G/T] | TGGGGGTTGGTGGCAATGTTGTGCGTGATGGCATCC |
| 21395_20 | 21 | [G/T] | TGTTAGACAGCTGCAGAGATGTGCGGTTGTCGGTCC |
| 21396_2 | 3 | [G/A] | CTGTGACCGCCGGCACGGTCGTGCCCGGGGTGATGG |
| 21397_32 | 33 | [T/C] | AAATTGATGCGTGCATTCTCATGCACAGACACTAAC |
| 21398_1 | 2 | [G/A] | TGTTCTCATCCTGCATATGCTTGCAACAGCGTTAAT |
| 21402_11 | 12 | [G/T] | AGCATGGAGGTGGCAGCATCATGCTGTGGACAGGAA |
| 21404_15 | 16 | [T/C] | TTATAGTTTGGAGCACTGGGATGCTCTAAGACCTCC |
| 21407_32 | 33 | [G/A] | CAGCGCTGTCAGGCAGATTTCTGCGAGATGGAGATG |
| 21411_7 | 8 | [C/T] | TGCGACTCGCTGGCACATGAATGCGCGTTGGCTGCT |
| 21413_2 | 3 | [A/T] | CCAACCTGAGGCGCATAGACTTGCTCTACAGAAGAA |
| 21414_28 | 29 | [C/T] | AAAGGCGAGGCTGCAGCGTGGTGCAGAACGTGTTTC |
| 21415_7 | 8 | [A/C] | ATTTGCCATTCAGCACACAAATGCATTGTAACCCAA |
| 21416_31 | 32 | [T/A] | AAAATAAGCTAGGCATCTAGGTGCCTGGAAATGGTG |
| 21418_3 | 4 | [T/C] | ACATGGTGGAGAGCAGACTGGTGCTGTGAGTCAGAA |
| 21426_19 | 20 | [T/C] | TAGACCCAGACGGCATGGCTGTGCCCGTGGCTGAGG |
| 21427_10 | 11 | [G/A] | GCCAGGACACGAGCAGTAACGTGCGAGAATCTACCT |
| 21434_15 | 16 | [C/T] | TATGTGAACACGGCACTGGAGTGCATGAAAAGTCTG |
| 21435_10 | 11 | [T/C] | TATACTCGTTTAGCACTCTGCTGCAACTAAGTGGGG |
| 21438_7 | 8 | [C/T] | GAAAATTCTCAGGCAAAAGACTGCGTTGCTTTGCTG |
| 21441_26 | 27 | [T/A] | AAGAAATGGACCGCAGGATGCTGCTGATGCTGCATG |
| 21450_34 | 35 | [C/A] | TGGTGACTTCGTGCACGCTGCTGCTTCTGCTCTCCG |
| 21451_9 | 10 | [C/T] | GGAGTGACGCGAGCACCTGAGTGCTGGGAAATATAA |
| 21453_1 | 2 | [C/T] | TTAGTTGTGCAAGCACGTCAGTGCCATATAAACAAC |
| 21454_18 | 19 | [G/T] | AATAATACAGGGGCAACAGGTTGCCGGGAAACTGCA |
| 21465_17 | 18 | [T/G] | TTGTTTTATCTGGCAAATAGTTGCAAGCTGAGCCTT |
| 21466_7 | 8 | [C/T] | CTAGTCACACTGGCAGCACAGTGCAAAAGTTTGCAC |
| 21468_26 | 27 | [C/G] | ATGCGGGAGAATGCAGAGCTGTGCTCCTAAACCGAA |
| 2147_5 | 6 | [C/T] | CACGGCGGGGCAGCAATCGGCTGCATGTTACAGGCT |
| 21470_30 | 31 | [G/A] | GTTGCGGTTTTTGCATGCATATGCTGGTACGGATTG |
| 21473_5 | 6 | [T/C] | CTTCGTCGGCGTGCACCTTGTTGCTGCTCCCGTGCC |
| 21478_11 | 12 | [T/G] | GAGATCCGGGTTGCAGGTACTTGCCTTGGTTTGCCT |
| 21479_6 | 7 | [G/A] | GTGCTAGCGTTAGCATGCTGCTGCACCTGGCCTCCG |
| 2148_1 | 2 | [C/T] | ACTCACGGTGAAGCATGGTGGTGCCAGCATTCTGCT |
| 21480_33 | 34 | [C/T] | GAGGGTCTGTTGGCACTGTCTTGCACCTTTTTTCTC |
| 21481_20 | 21 | [A/G] | TTGTGACATGGTGCATTATCATGCCTGGAGTAACCA |
| 21483_3 | 4 | [A/T] | TGTAAGGGGTTTGCAGTGACCTGCAATGCCTGTTGT |
| 21486_11 | 12 | [C/T] | AACGTGTTAAACGCAGGTCAGTGCATGTCTTCACAC |
| 21488_32 | 33 | [C/T] | AGTCCAAAAGCAGCAAACTTTTGCCGAAACATCCTT |
| 21489_3 | 4 | [G/A] | TCAGAACCAAATGCAGTTCAATGCACATCAGCTAAA |
| 21492_1 | 2 | [C/A] | CCAGTTTTTCTGGCACCTCAGTGCACATTGCAATAC |
| 21493_20 | 21 | [C/T] | TCAGAGCTCTGTGCAGACCACTGCAGTCTTTGTGGA |
| 21494_32 | 33 | [A/T] | AGGGGGAAATGGGCAAATTTGTGCTCCAACAGAGAA |
| 21498_28 | 29 | [C/T] | GCCATAAACATGGCACTTCACTGCAGCCCAAATGCT |
| 21499_29 | 30 | [T/G] | CTCAGTAGGTTTGCAGCAGACTGCTCCCCTCGCCTC |
| 215_30 | 31 | [T/A] | CTGTGCAAATCTGCAAAACCATGCACCTTAAGCCAT |
| 21500_15 | 16 | [C/T] | ATGTCTGAGAGTGCACTCTCATGCACTGGGCTAACT |
| 21503_18 | 19 | [A/T] | GCTGTCTGACCTGCAGCAACATGCTGTTCCACTATA |
| 21511_6 | 7 | [C/T] | CACATCCCACATGCATTCATGTGCATGAAGCACCCA |
| 21518_3 | 4 | [C/T] | ACTCGACCCACGGCAGCAGTGTGCTCCAGGTCTTAG |
| 21525_11 | 12 | [G/T] | TTACTACATGGGGCACCACCATGCCACTGCCACACT |
| 21527_1 | 2 | [G/A] | CGAGAGATCGGCGCACTGAATTGCTACAAATACTTC |
| 21529_33 | 34 | [C/A] | TTGTGCTTAGGGGCATGAAGATGCCAGATATCACAG |
| 21530_4 | 5 | [A/C] | TGTCAGAAGACAGCAGCTCCTTGCCCAACCTCCACT |
| 21533_25 | 26 | [A/G] | TCACTATGACATGCACTCTTTTGCAATCTCGCTTTG |
| 21536_4 | 5 | [A/G] | ACTCACACAACAGCAGCCATCTGCTCATTTACTTCA |
| 21539_11 | 12 | [G/T] | ACACCACATGTGGCACCACCATGCTGACAAGGCTGG |
| 21543_10 | 11 | [G/A] | CAAAACTCACGGGCAATTTTGTGCATTTCGGACACT |
| 21544_7 | 8 | [T/G] | GATCGGGTTCAGGCATGCTGGTGCATGACGGAGAAT |
| 21545_3 | 4 | [C/T] | TCTCAGGCTAATGCAATTTTGTGCCGGTGTGTATCG |
| 21553_19 | 20 | [C/T] | GCCACCCCTGTAGCAAGGCCATGCCAGCTTAGGCAA |
| 21554_16 | 17 | [T/C] | GTGTAAATGTCAGCACTACACTGCACACTCAGCATT |
| 21563_5 | 6 | [G/A] | ACGCTGATGTGTGCAGGGCTCTGCAGCCCTTTACAG |
| 21564_29 | 30 | [C/T] | CCCACAGGTCAAGCAGATGAGTGCAAATGCGCATTA |
| 21568_15 | 16 | [G/A] | TGCGATGGCACTGCAGTATCTTGCAGTAGCAACTGA |
| 21580_26 | 27 | [A/G] | GCACGTCTGGAAGCATCGGCGTGCCCACTGGTTACA |
| 21583_2 | 3 | [T/C] | TACAGGAGCATTGCAAAATAATGCCAGTAGTGCTGG |
| 21589_2 | 3 | [A/T] | CTAATGCATTGTGCACCACGCTGCACTGCTGCCCCT |
| 21590_31 | 32 | [C/A] | TCTAAGACTTTTGCACAACGCTGCATAATGCCAGTT |
| 21591_6 | 7 | [A/T] | TCCCGCATTTTTGCAGGCATGTGCCAACAACAGTAA |
| 21594_1 | 2 | [G/A] | TAAGGAGCAGTGGCAGACGCCTGCTGGTACAAGTAT |
| 21595_1 | 2 | [C/A] | GCAGGGCCTCAAGCAGGCAAGTGCGATTCTGGTGGT |
| 216_7 | 8 | [C/A] | AAAGGAACTGAGGCAGAGTTTTGCATTTGGCTGATT |
| 21600_28 | 29 | [T/G] | GGTCCCCTGTTCGCATCCTCATGCCTCGTGTCATGA |
| 21601_10 | 11 | [C/T] | GTAGTTTTTTCAGCACAGAGGTGCCGATTAAGCACA |
| 21602_24 | 25 | [C/A] | AATCGAGAAGCTGCAGAGAAATGCAGACAAGGTGGA |
| 21604_33 | 34 | [C/A] | CATAGCAGGGTGGCACAGTGGTGCCCACAAACCCCC |
| 21611_20 | 21 | [G/A] | GTACTGATTGGTGCAGTGGCGTGCAGGCCCTGTACG |
| 21616_33 | 34 | [C/T] | GTGTCCATGCCAGCAGAATGATGCTCAGTCATTCGT |
| 21626_1 | 2 | [G/A] | TGAAAACGGAGAGCAAATAGGTGCTCATGGTGTGTA |
| 21629_16 | 17 | [G/A] | ATAGACTCTGAGGCAGGGACGTGCCTCGCTCTTACC |
| 21633_5 | 6 | [C/T] | GATCTCTCGCCAGCAGCCTGCTGCTTGCAAATAGAC |
| 21636_16 | 17 | [C/T] | TTTCTTCAATCTGCATCGCGGTGCCCTAAAAAGTGA |
| 21641_24 | 25 | [C/T] | CTACCCTGCACAGCATGTATGTGCCCACACACACAG |
| 21650_11 | 12 | [A/G] | GATTGAGGGGTAGCAGCAGAGTGCTCGTTTGCGCAA |
| 21653_26 | 27 | [G/A] | CTCGTGGGGTGTGCAAACTTTTGCACGGATCATATC |
| 21657_2 | 3 | [A/G] | CCACACCGCACCGCACCACAATGCTGAATAAAACCC |
| 21659_29 | 30 | [T/C] | ATCAATATGGTGGCAAAGTGGTGCAGCACTGGAGAA |
| 21667_28 | 29 | [C/T] | ACTACACATGTAGCAACCTTCTGCTCACCTTTCCTG |
| 21670_27 | 28 | [C/T] | GGCACCTGGGGAGCAGTTGGTTGCCTTCAGCCTTAT |
| 21676_10 | 11 | [G/C] | CTCTAAAGCGGCGCATTAACCTGCAATCTAAATGCA |
| 21677_24 | 25 | [C/A] | GAGCTCCAGCAGGCAGCGGACTGCCGCGGTGCAGAG |
| 21679_30 | 31 | [A/T] | TAATACACTGGAGCAGCACTTTGCTTCACTAAACTC |
| 21684_18 | 19 | [G/A] | CATGTGTCCTAAGCATCCGTGTGCTGCTTTGCCTGT |
| 21686_10 | 11 | [C/T] | CCACCGTCTTTAGCAGGTCCTTGCCCTCGACGTCTG |
| 21687_17 | 18 | [G/A] | CCGTGCTAAAAGGCAGCGACGTGCTTTATTTTACAT |
| 21693_17 | 18 | [A/G] | CTGGTATATCCTGCAACAGATTGCCACCCTGTCCAG |
| 21695_4 | 5 | [C/T] | TCTGCCCTCCCTGCACGCCAGTGCCGTTCGTACTGT |
| 21700_27 | 28 | [G/T] | CACATGTTTACCGCAACAAGGTGCTGAGTTTACAGG |
| 21703_15 | 16 | [A/T] | GCATCCCCTGCAGCAACACTCTGCAGAGGGTCTAAT |
| 21705_10 | 11 | [C/T] | GCATGTCTGTCTGCAGTTCAATGCAAGTTTTCTGGT |
| 21706_29 | 30 | [G/A] | GCAGATGCGGAGGCAGGAGAGTGCAGTGCGGTCATG |
| 21707_5 | 6 | [A/G] | TTACGATCCCGGGCATCAGCATGCTCTGCCTCCTCA |
| 21708_6 | 7 | [T/C] | CCGGGCTGATGAGCATGAATGTGCACACAACACACC |
| 21716_29 | 30 | [C/T] | ATGTGAGTTTGTGCATACGAATGCGCGTTCATGCAT |
| 21720_28 | 29 | [C/G] | AGGACAGATGAGGCACTGCTCTGCAGTACTGAAAAT |
| 21723_26 | 27 | [G/A] | ATCTCGAACCCTGCATTCCTCTGCTCGATTCTCAGC |
| 21729_18 | 19 | [C/T] | GGCACAAAGCAGGCAAACCCCTGCACAGGGTGCCTA |
| 21732_29 | 30 | [C/T] | TGCTTTATTCCAGCACCAAACTGCTCTGGCGTTTCA |
| 21735_29 | 30 | [A/G] | TGGTTTGTACTCGCATGTGAATGCTTTGCACTGGAC |
| 21737_15 | 16 | [G/C] | GAGAGGAATGGAGCAGTGTTGTGCTGAAGCCCCAGG |
| 2174_11 | 12 | [A/T] | GGTGGGCAGGAAGCAGATGGCTGCAGATTAACGTGA |
| 21741_19 | 20 | [C/T] | TGCGTATGGCCAGCACCCTCCTGCTGCCTGACCTTG |
| 21744_30 | 31 | [A/T] | CAGGGAGCAAGTGCAGCCCCTTGCATCATTAGCACT |
| 21745_27 | 28 | [T/G] | ACGAGAGTTGACGCAGGACTCTGCTGCTCCTGACAG |
| 21748_32 | 33 | [T/C] | GACAGACACAATGCACATCAGTGCCACTGTTGTTTA |
| 2175_15 | 16 | [G/A] | TAACAAACAGAAGCAAAAACCTGCACCTGGTTGTAA |
| 21751_34 | 35 | [C/T] | AGACTCCGGACTGCAATTTGCTGCGCGAGACTCTCA |
| 21752_4 | 5 | [T/C] | CACCTGGGTGAGGCAAAACTCTGCGTCTCACAGTAA |
| 21756_24 | 25 | [G/A] | GTCTGTCTCTGGGCACTCCAGTGCGCTGTCATTAAA |
| 21759_3 | 4 | [C/T] | ACACTCCCTGCAGCACTACGGTGCCAGTCACCAATA |
| 21761_3 | 4 | [A/G] | GGAAGAAGGGCAGCAGATCCATGCTCCCTGGGAGAC |
| 21767_32 | 33 | [G/A] | TGTGTGTATCTTGCAGCAGTGTGCATAAAGCCGAGG |
| 21768_1 | 2 | [T/C] | GTGTATATCGGTGCAGGAATTTGCAAGTGTGTCCTT |
| 21773_24 | 25 | [G/A] | CTGAAGCAGCTAGCATACAGTTGCGCGTCTGATCCC |
| 2178_15 | 16 | [T/C] | AAGCGACGAGAAGCATGTTGATGCTCCTGAGTCCAG |
| 21781_10 | 11 | [C/T] | AAGATCCGCTCAGCAGCTCCGTGCATCAAGCATGAG |
| 21783_9 | 10 | [G/A] | GTCTGCATCGGCGCATGTACATGCCAGTGATCTAAC |
| 21784_2 | 3 | [T/A] | ACTCAAAACCAGGCACCATGATGCAATTGTGAAGCT |
| 21787_4 | 5 | [A/G] | TTTCAAGTCACTGCACCTCTATGCCGAAGAACATCC |
| 21789_17 | 18 | [C/T] | TTGCATTGTGTGGCAGACGAATGCATTGCTTTGAAT |
| 21792_29 | 30 | [C/T] | ATGAAGAATGTTGCATTAAGTTGCAGAGCCGTTGTT |
| 21793_5 | 6 | [C/A] | TAATCCGACACAGCATCAACCTGCTGTTTTTGAGAA |
| 21794_25 | 26 | [C/T] | GTGTGTGTGTGTGCAGGGTAATGCCCACTTGTTAAT |
| 21797_3 | 4 | [C/T] | AATCGCTTTGGTGCAGCAATGTGCCTTTTCCACACA |
| 21799_7 | 8 | [G/A] | GACCTGAGCTGAGCACCACCATGCCTTTTAACCAGG |
| 2181_3 | 4 | [T/C] | CCATGTCTAACAGCACCATGATGCCCATAGAGTGAG |
| 21811_34 | 35 | [T/C] | CAACCAATCTGTGCAACCACATGCATGTGGCTTTTA |
| 21813_34 | 35 | [A/G] | CCCATTCAGATTGCACTGCAGTGCCTAAAGTTCGGA |
| 21814_31 | 32 | [C/A] | AAATGGCAAAGCGCATCAAGCTGCCTTACACCATGC |
| 21819_6 | 7 | [C/T] | AGAGGTCTTACAGCACATTTGTGCTTTCGTGCGAAC |
| 21822_33 | 34 | [G/T] | CTCCTGCACTCCGCAGCTGTATGCTCATAAAAAGGG |
| 21838_34 | 35 | [G/A] | CAGAGTTGGTAAGCAGGGCAGTGCAGAATTTAGTGA |
| 21839_33 | 34 | [G/C] | TTACTGGCAGTGGCATAAGTATGCACACAATCTCTG |
| 21846_20 | 21 | [C/T] | ATGACAGAGACGGCAGTTAGCTGCACACTCGTGCAT |
| 21847_29 | 30 | [T/C] | ATTGAAAGTGAAGCATTTACCTGCTGGGCTTGCACG |
| 21850_18 | 19 | [G/C] | GGACGATGCGAGGCAGCTGTTTGCCCTGGCAGCAGG |
| 21853_31 | 32 | [G/A] | CACAGCTGTTGTGCAGTTGCATGCTATCACGGCGCA |
| 21858_18 | 19 | [G/A] | GATCTATTTAGCGCAGACGGTTGCTGTCTGGACTAT |
| 21869_1 | 2 | [C/T] | CCGCCCCTCCGTGCAGTCCTTTGCTCTCGACTGAGG |
| 21871_32 | 33 | [G/A] | CTAACGTGGAGGGCAGATCTCTGCCGGAGCTCGGCC |
| 21877_3 | 4 | [T/G] | TACTTACGAGCAGCATAGTGGTGCTGCAGGCACTGT |
| 21880_16 | 17 | [G/A] | CTACACACTGCGGCATAAGCATGCAATTGGTATCTC |
| 21882_32 | 33 | [G/A] | CAGCAGATAAGCGCAATGTGATGCTCTTAGCTGAAC |
| 21884_4 | 5 | [G/C] | TGCTGAGGGAGAGCATTGTTTTGCTGCTAAATGTGC |
| 2189_17 | 18 | [G/A] | GATGAGCGAGATGCAGAGGGATGCATTGTAAAAGCT |
| 2190_20 | 21 | [A/G] | TGTAAATGCTGCGCAGTTAAGTGCAGAAAGTTGGAT |
| 21903_18 | 19 | [T/C] | GCGGCACTGCTCGCATTTTACTGCACAGCACCAGTG |
| 21907_2 | 3 | [C/T] | TTCCCTCCTTTTGCAATGAGTTGCGAAATCGATTTC |
| 21910_28 | 29 | [G/A] | GCTAGAAAATATGCAAATGCGTGCACCAGAAGCTGT |
| 21911_27 | 28 | [C/T] | TCGGTCTATGGGGCAGTTGGCTGCCCTCGGCTGGCC |
| 21922_18 | 19 | [C/T] | TGCAGGTCAAAAGCACAGCAGTGCAGCAAAAACTGA |
| 21927_6 | 7 | [C/T] | TATAAACGTTCTGCATGGTGTTGCACCTTCTCGTAT |
| 2193_2 | 3 | [C/T] | TTCATTATGGTGGCATGATGGTGCAGTGAGACAGCA |
| 21931_3 | 4 | [A/G] | GATAGAAAGCAGGCACATTGCTGCAGACCAAGTACA |
| 21932_4 | 5 | [A/T] | TCAGAGCGAAAAGCATGTGTGTGCAACCTGACCACT |
| 21937_7 | 8 | [T/C] | GAGATGATGGTAGCAGTGTGGTGCTTCTCCCCAGAA |
| 21941_27 | 28 | [T/A] | CTAGGCATGAGTGCATGGGTCTGCACTTACTGTGAC |
| 21944_31 | 32 | [C/A] | GACCGATTAGTTGCATTAGCTTGCTACTGTTCGCAA |
| 21945_6 | 7 | [A/G] | TGCTACACTTGCGCATGTGTGTGCAGGATGCCGATA |
| 21947_19 | 20 | [A/T] | CCACACTGTACTGCACTCTACTGCGAATTTTCCTTT |
| 21951_19 | 20 | [C/T] | ACAGGGCCAGAAGCATGATCCTGCTGGAGAAAAGTC |
| 21953_9 | 10 | [C/T] | GTTCTGGCACGAGCAGTCACGTGCCGATCGTGACGA |
| 21957_24 | 25 | [C/T] | GAAGGCTGTGCTGCAGTAGACTGCCTGCCTGGGCAA |
| 21958_28 | 29 | [A/C] | CCTTCAATTACTGCACGCCCATGCAGTAAACGCAAC |
| 2196_3 | 4 | [A/T] | TGCAAATACAAGGCAACAGAATGCAGAACGGACATC |
| 21962_26 | 27 | [G/A] | GAAGCAGAACCTGCATTTTCGTGCAGGAAATAAGGG |
| 21968_30 | 31 | [C/T] | GAGTGAACAGGGGCAGGTGTTTGCGTGGGGCGCAGG |
| 2197_3 | 4 | [C/T] | CACCGTCCTCCTGCATCTCACTGCACCTGCTCTCTC |
| 21970_28 | 29 | [T/C] | TACAGGCGAAGTGCACAGTGTTGCCTCCTGGTTCAG |
| 21973_30 | 31 | [C/T] | GGGAGTTTTCATGCATGGCCGTGCATTTTTCTGGTG |
| 21974_5 | 6 | [C/T] | ACTGACGAGGCTGCAACATACTGCCAGCATCACATA |
| 21977_6 | 7 | [G/T] | CAGATAGCTGAGGCATTGTCATGCCAATCTCCATGC |
| 21980_34 | 35 | [A/C] | TTACCCACCCATGCAGTAAAATGCACACACACACAC |
| 21981_30 | 31 | [T/G] | TCATTGTGGTCTGCACCCGAGTGCGATTTCTGTGTT |
| 21983_28 | 29 | [A/G] | GGAGCTCCAGGGGCAGTAGAGTGCAAACAGTGGGGG |
| 21986_32 | 33 | [G/A] | GTGTGTTGAGATGCATTGTGATGCAGCTGTGAGGTT |
| 21990_27 | 28 | [A/C] | ATACACAGGGTGGCACAGTGGTGCTGCATACCCGTG |
| 21993_27 | 28 | [T/G] | TTTGTTTTTTCTGCACGTGTTTGCTCATGGACCTAT |
| 21994_19 | 20 | [C/T] | CCACGCCCGTCTGCAGACACGTGCGAAGCTCAAACA |
| 21998_4 | 5 | [C/T] | AAATCTGATTGAGCATCTACATGCTGGGCAAACTAG |
| 22015_9 | 10 | [G/A] | GAACCGCAAGAGGCACTTCAATGCCCCGTCACACAT |
| 22019_11 | 12 | [A/G] | TTTCATGGCCAAGCAGCTGCTTGCAAGTCTTACATG |
| 22020_34 | 35 | [G/A] | GACGTCCAGACTGCACAAAGCTGCACCCTGATCTGA |
| 22026_32 | 33 | [C/T] | CTCCTAAACTTGGCATGACCCTGCAGCCACAGCCTT |
| 2203_19 | 20 | [G/A] | CTGAGACCAAAGGCAAATCGCTGCAGGACATCGACA |
| 22033_34 | 35 | [A/T] | TTCTATGTCCTGGCACCAAGGTGCTGGGATGAACAT |
| 22037_20 | 21 | [G/A] | ATGTCACACCCTGCAGGACAGTGCAGTGTGAGAGAC |
| 22038_33 | 34 | [G/A] | GTTCTCAGTGTAGCAGCACAATGCAGTGCGTCAAAT |
| 22056_16 | 17 | [C/T] | TCTCATCCATCAGCATCCATCTGCTCATGCGCCAGT |
| 22057_7 | 8 | [G/A] | CAGATTAGGATTGCAACACACTGCGAACGGGGAGTT |
| 22058_4 | 5 | [A/G] | AAAAAGAAAGTGGCAAAACCTTGCAAGGGTGTGAAG |
| 22061_31 | 32 | [T/G] | AGACTCTATGAGGCAGCTCTCTGCATAGGGATCTCC |
| 22062_32 | 33 | [T/C] | TGCTGCACCGCTGCACAGGTATGCCGATTTATTAAC |
| 22063_26 | 27 | [G/A] | TCCTTCAGTGGAGCAAAGAGATGCCTGATCACCGGG |
| 22066_34 | 35 | [G/A] | CTGGAATGGTGCGCAGCCCTGTGCCATGGGAGTGGC |
| 22070_17 | 18 | [C/T] | GCATCTATCTCAGCACTCGTATGCACACGTGCACAT |
| 22071_9 | 10 | [C/T] | TAAAAAGCACGAGCAGGTGTCTGCATTCACAACGTG |
| 22074_2 | 3 | [A/T] | TGAGTCTCTGATGCATGCATGTGCCCTGGGCTTGAC |
| 22078_32 | 33 | [A/C] | TCCACCGTTCAGGCATGTCGATGCTCATATTTAATT |
| 2208_2 | 3 | [T/C] | TGTCTATCTCAGGCATGCACATGCAGCAACAATTGT |
| 22084_6 | 7 | [C/G] | ACATTACCTGTGGCACCACTATGCTGCCCCAGAGGG |
| 22097_31 | 32 | [C/A] | TGCGTATGACTTGCATTAAGCTGCCAAACCACTGAC |
| 22099_9 | 10 | [A/G] | TACAACTCTAAAGCAGAGCTTTGCCAAGACACCAGA |
| 22106_24 | 25 | [T/C] | TAAAGGGCCAGCGCATTGTCATGCTTGTAAAAATAC |
| 22108_1 | 2 | [G/A] | CAGTGTCTGCGTGCACATCTGTGCCTGAGTGATATG |
| 22109_28 | 29 | [C/A] | CAGGAAACAGTCGCAGGACAATGCACAGCGAGGAAA |
| 22115_28 | 29 | [A/G] | AGGAGGAGTTCAGCATCTCTCTGCTTCCATCAGCAC |
| 22124_19 | 20 | [G/T] | GACTGGATGCCTGCAGATGGCTGCTTGGATTGAGCA |
| 22127_8 | 9 | [G/A] | AAAGCTACGCATGCACCAAAATGCACATCACAATTG |
| 22130_18 | 19 | [C/T] | GGCTAACCAGAAGCACCTCAGTGCATCCAGGCACAT |
| 22131_1 | 2 | [C/T] | TCATCTGTTGTTGCACAACAATGCCACTTTGCACTA |
| 22133_19 | 20 | [C/T] | ACATGGCGCTTCGCAACCACATGCGCATCCACACGC |
| 22146_3 | 4 | [C/A] | ACACTGTCATATGCAGTGACGTGCCAGTGCATGCTG |
| 22148_16 | 17 | [C/T] | ATGATTCCGTCTGCACCGTCTTGCTTCATCATGAAT |
| 22152_1 | 2 | [T/C] | CTATGTAGCAAGGCACATGATTGCTTTAACTCCCAT |
| 22153_26 | 27 | [C/A] | TAAGAAAAGTCAGCATTGCATTGCTGCATGCCTTTC |
| 22154_3 | 4 | [C/G] | TGTCCAGCTCCAGCACCAGCGTGCAATTGAGCTGGA |
| 22157_24 | 25 | [C/T] | ACCCATGTGTCTGCAGCTAGGTGCCGCTTATTAGTG |
| 22160_24 | 25 | [T/A] | GGTGCCTCTAATGCAGTCCACTGCTCTGAAATCACT |
| 22161_15 | 16 | [A/T] | GCTCGCAGACTTGCAACTTCGTGCGAAAATGACAGG |
| 22163_33 | 34 | [A/G] | CTAACGTGATTAGCACAATGCTGCGTATGTCACATG |
| 22167_32 | 33 | [T/G] | TGCTCACTGATTGCATTCTCCTGCATTTGCTGTTTT |
| 22168_4 | 5 | [C/T] | GTTCCAGAGCAGGCAGACCCCTGCACTTTCTGTGCA |
| 22173_7 | 8 | [G/A] | ACACACTGAGTGGCACGAAAATGCAAAACACGTCTA |
| 22177_16 | 17 | [T/G] | GGGAATTCTTTCGCATTCTCATGCCAACGTAGCACC |
| 22180_27 | 28 | [G/A] | AAGGCGTCCACTGCAGGCCAATGCACAGAGGCAATT |
| 22185_7 | 8 | [T/C] | CGGTACGTCGGTGCATTCCTGTGCACGCGTGTTAGA |
| 2219_19 | 20 | [G/A] | TTATTAAGGGTGGCATATTGGTGCCACAGGCGATAT |
| 22192_24 | 25 | [G/A] | CACGGTCCTGGGGCACATCGCTGCGTCTCTTAATGA |
| 22195_31 | 32 | [G/A] | CGCACGTTTTTGGCATTTACGTGCAGCGCAGGAAAA |
| 22196_24 | 25 | [G/A] | CCGCTAGTCACTGCAACAGATTGCGGCTTCTTTAGA |
| 22199_25 | 26 | [T/C] | CCTTTATGTAAAGCAGATAGATGCCCGCTGGGCTCT |
| 222_17 | 18 | [G/C] | TGAGGAACACCTGCACAGTCATGCAGTTATCTAATC |
| 22209_27 | 28 | [G/A] | TCTGTAAGCGTGGCAGCATTGTGCTTGGCTCTGCTG |
| 2221_28 | 29 | [G/C] | GCTGTTTTGTTAGCAATAGTCTGCATGTGCTGCAAT |
| 22213_7 | 8 | [G/A] | TCACGTCGTTCGGCAAAACCATGCACCAAAATACGA |
| 22217_25 | 26 | [A/G] | CTGTCCTCCTGGGCACGCTTCTGCAAGGAGTTCACG |
| 22220_7 | 8 | [G/A] | ACTGTCCGTATCGCACTCCTGTGCAGTTCTGTTCAT |
| 22224_17 | 18 | [G/A] | ACATTTCTCAATGCACCGAAATGCTCCACTTGACAT |
| 22228_16 | 17 | [A/C] | TCTCAGACGCATGCACAGACGTGCTGTAAAGCGAAG |
| 22230_16 | 17 | [C/T] | CATGGTTCCAGCGCACCTGGCTGCTATGGAGACGGG |
| 22231_18 | 19 | [G/C] | GACTTGGTGCGAGCAGCTGGTTGCTCAATACGTGAT |
| 22234_1 | 2 | [G/T] | CGGCTGCAGGCGGCAGTGTCCTGCCTGAGGGGTTGC |
| 22235_27 | 28 | [C/A] | GTAAAACCCACTGCACCACTGTGCTTCCTTACTACA |
| 22240_20 | 21 | [C/T] | TTCAGCATAACTGCAAGGTTCTGCATGGCTCTAGAT |
| 22244_28 | 29 | [A/G] | CTGCCAATGAGTGCAAACAGATGCCGCAAGTGAAGG |
| 22250_1 | 2 | [C/A] | ACCCTTTCCTTGGCATTCCGCTGCACTAGACGCACT |
| 22255_34 | 35 | [G/A] | GCAGCAGGGGTAGCAGTGCTTTGCTGAATGCTGAGT |
| 22260_34 | 35 | [G/A] | CTGTGAGCGTAGGCAGTAAGATGCAGTTATGTACGG |
| 22261_4 | 5 | [T/G] | TCATTATAGGCAGCACAGTGGTGCTACAGGTAGTGT |
| 22262_33 | 34 | [C/T] | TGTGCGCTATCGGCACCACTATGCCCCCGAGAACGG |
| 22264_2 | 3 | [C/T] | TTCATCTTTCCAGCAGTGACTTGCTTTTCAGAGACC |
| 22268_16 | 17 | [T/G] | CCTCTGGTCTTTGCATTGGGCTGCTGGAGGACAGTG |
| 22272_28 | 29 | [T/G] | AGTGGTGCAGGTGCACAGTTCTGCAGGATGACCCAC |
| 22274_25 | 26 | [A/C] | ACAGCGAGTGATGCAGGAATGTGCGATTCTCTGGCC |
| 22275_7 | 8 | [C/A] | GATTACACACCGGCATGCATGTGCATACAAACTTAG |
| 2228_33 | 34 | [G/T] | TCATTTCTAAACGCAATCTTGTGCATGTCTGGGGCG |
| 22288_31 | 32 | [C/T] | TGGACGTGGCGCGCAACATTTTGCGCGAGTTCGCCC |
| 22289_28 | 29 | [C/T] | AAACTGCACAAGGCAATGACTTGCTACCCGGTTATA |
| 22290_15 | 16 | [T/C] | CTAATCAGGAGTGCATTGAGGTGCATGAATCGGTAA |
| 22291_7 | 8 | [C/T] | CGAAAGCCGTGCGCAACACAATGCCATAGATTTATT |
| 22295_16 | 17 | [C/G] | TATCACCTTTTGGCACCAGACTGCAGAGGCATAAAG |
| 22298_10 | 11 | [G/A] | GGAAACGATGGAGCATCTCTTTGCATTCCTGATCAA |
| 22299_25 | 26 | [G/A] | GACTGGTTTGCGGCAAAGATTTGCAGATAAAGCTCT |
| 2230_25 | 26 | [C/T] | CGCAGAAACAATGCACGGTGTTGCACCACTTCATCT |
| 22302_11 | 12 | [T/A] | CCTGTCATGCCTGCATTGTAGTGCATGGCTGAACCT |
| 22303_2 | 3 | [C/G] | AGCAAACACTCAGCAACCATATGCTCACAGTGTGCA |
| 22307_30 | 31 | [G/A] | TTTCTATTGACGGCACTAACCTGCTATTACGGCCAT |
| 22310_15 | 16 | [A/G] | TGTAAATGTGTTGCAATAGACTGCCACCTTGTCCGA |
| 22321_7 | 8 | [C/A] | ATTCTTACCAACGCAGAAAGCTGCATCAGTGTAGTG |
| 22323_4 | 5 | [G/C] | CCCAGTAAAACTGCAGGCAGCTGCTTTCACGAGAAT |
| 22326_33 | 34 | [G/A] | TTGAAATGTGGGGCATGTGTGTGCCCACAGAGTGAA |
| 22327_34 | 35 | [T/C] | GGATGATCTCGGGCATGTGGCTGCACATCTCATATA |
| 2233_28 | 29 | [T/G] | TGAACGTATTTTGCAGTGTATTGCAGCATGCAGTTT |
| 22331_6 | 7 | [T/C] | GTGTCTTTGTGAGCATGTGTGTGCGTTGTGTGTGTG |
| 22332_5 | 6 | [C/T] | TACATCTACTGTGCAGCTCAATGCCATATGGGGCAG |
| 22333_24 | 25 | [G/A] | GCAATTCTGGGAGCAATTTGCTGCGAGGGTTCTTGA |
| 22334_27 | 28 | [G/A] | CACTGAGGGGCAGCAAGACTGTGCAGTGGTGAGTGA |
| 22336_3 | 4 | [C/G] | TAGCGCTGGCATGCATGAGCGTGCTAAGCTGTCGAA |
| 22340_34 | 35 | [C/T] | CAAGGCGTATGAGCAGATTACTGCAATGCCACTTTC |
| 22342_1 | 2 | [G/A] | GGAACAGTGGTCGCAGTGGCATGCTGGTACAATAGC |
| 22345_5 | 6 | [T/C] | GTGTCTGTGCAGGCAAAAATATGCATATCCCTTCCA |
| 22358_34 | 35 | [A/G] | GTGGATTGTTCTGCAGTGAAGTGCCTCAATAACCAA |
| 22359_15 | 16 | [C/T] | AAGGTAGACACTGCACAAAGGTGCCAGAATACCCAA |
| 2236_4 | 5 | [G/C] | GGTAGAATGGCAGCACCTGTGTGCGGATGTATTTCA |
| 22364_30 | 31 | [A/T] | TTCTGAACTGTTGCACATTGTTGCAACAACAACCAA |
| 22366_25 | 26 | [G/C] | AGTGTTTGCAGGGCATGCGTTTGCGGCAGTACCACG |
| 22384_29 | 30 | [T/C] | ACGGGCCTCTCTGCACTCTACTGCTACCTTGCAATT |
| 22390_33 | 34 | [G/A] | ACCGTGCTACTTGCAGCAGTTTGCTCCTTGTATGAA |
| 22393_31 | 32 | [C/G] | CAGGAGAATGTTGCACTGATTTGCAGTGACACTTCT |
| 22395_24 | 25 | [A/G] | AACAGTGAATCAGCAGCAATGTGCACTGAGTCAGTG |
| 22397_33 | 34 | [C/T] | ACTGAGACTTGCGCAGGAAATTGCGTCAGATTTCAA |
| 22404_16 | 17 | [C/A] | TCTGGCTACTAAGCATCATTTTGCAGCAAGTCATAA |
| 22410_19 | 20 | [A/G] | CAGGCTGGATGCGCACTGAAATGCTGGGTAACACTG |
| 22412_9 | 10 | [C/T] | GTTTGTCGGCCGGCATCTATTTGCATGTCAATGACA |
| 22423_32 | 33 | [G/A] | TACCACAGCCTGGCATTTGCGTGCCCGTCTATGAAC |
| 22427_6 | 7 | [A/G] | GGCTACAACTCTGCACAGCTTTGCCCTCTTTACCAA |
| 22428_17 | 18 | [T/A] | CGAACATCTCCAGCACTTAGTTGCTTACCTGCTGCT |
| 22431_19 | 20 | [T/G] | CCTCCTTCTTTGGCATCCCTTTGCAGTGTATCGCAC |
| 22435_16 | 17 | [A/G] | GAAACCCCACTGGCACAGACCTGCCTCGCCGCACCT |
| 22436_18 | 19 | [G/T] | GCATTACTGCTGGCACCAGCATGCTGCTCTTTATTT |
| 22437_18 | 19 | [G/T] | CACTAAACGGTGGCAGAAGTGTGCACACTCAGGCCC |
| 22438_29 | 30 | [G/A] | GAACGAGAGCATGCAAACGTTTGCAAATCGGCCTGT |
| 22439_9 | 10 | [G/A] | GGCCCTGTCAATGCATCACAGTGCAACGGCAGCTTC |
| 22445_2 | 3 | [C/A] | TTCTGTCCTCCAGCAGGGTCATGCTAAAAGCTCCAG |
| 22449_33 | 34 | [C/A] | AATACAGCAGCTGCAATTTGCTGCAAACCTGGACCA |
| 2245_3 | 4 | [T/C] | TTCTTTTTAAAGGCAGGCAGTTGCAGCAGCTGCCGT |
| 22451_3 | 4 | [A/G] | AGAAAGACTGGGGCAGTAGTATGCTTGCGGTTAAGG |
| 22461_32 | 33 | [C/T] | AGAACCGGTTCTGCATTAAGATGCATCACAGTCGCA |
| 22469_18 | 19 | [G/A] | TATTACAATGCAGCACTCGTCTGCTACACAGGCGAA |
| 22470_17 | 18 | [C/T] | GTTCCTGAAGAAGCAGCCCACTGCAGTCACGCCCAG |
| 22471_18 | 19 | [T/G] | TCCACTTCCCCAGCAGATTGCTGCAAAGAACTTTCC |
| 22476_11 | 12 | [A/G] | TCTCTAGGAGCGGCAGAGTTCTGCTTCCATGCAGCC |
| 22478_3 | 4 | [C/G] | ACTCGATCGATAGCATTTTCATGCTGAGGCTGTTTT |
| 22484_5 | 6 | [C/A] | AAAGACACAAAGGCAGTAGTTTGCAGTGGGCAGGTT |
| 22488_24 | 25 | [C/T] | GGAAGAGAAGAAGCAAGGTGGTGCCGCTATAGGTGA |
| 22489_28 | 29 | [T/C] | CTTGGCGAAAACGCAAAATGCTGCTCTCTCTTTTTC |
| 22493_4 | 5 | [T/G] | AGAATGGGACGTGCATAGAAATGCTGAGTTCGGCTG |
| 22496_15 | 16 | [G/A] | CAGGTGTATCACGCAGATAAATGCCCACCCTCCCCC |
| 22500_28 | 29 | [G/T] | AGTGATGCCTGAGCACCCTGCTGCTGCTGTACAGTC |
| 22507_24 | 25 | [G/A] | CGGCAACACGCAGCATCTGGCTGCGAAATCAAAAAT |
| 22511_9 | 10 | [C/A] | AGGATCCAACGTGCAAACATTTGCAGCGTGTGTGTT |
| 22518_15 | 16 | [G/A] | AAAATGCTGACGGCAGACAACTGCTATCATCATGGT |
| 22519_16 | 17 | [C/G] | CACTACCTTGTGGCACCACTGTGCCACCTGTAAGTA |
| 22520_19 | 20 | [C/T] | CGCCCAGGAGATGCAACACCGTGCAATCTTTCCATC |
| 22534_33 | 34 | [C/T] | CACAGCAAGTTGGCATCGGTGTGCCAAGATTCTCCT |
| 22538_27 | 28 | [A/G] | TCAGAAGGTGGTGCACCTGGCTGCTCAAAGATCCTG |
| 2254_6 | 7 | [G/A] | AGAAACGAGTCTGCAATGGGATGCAGCGGCTGTATT |
| 22542_4 | 5 | [G/T] | TTCGGCTCACTTGCAAAACCCTGCAAAAGAACCTTG |
| 22546_25 | 26 | [C/T] | GTGGCCTATTCAGCACTCGGTTGCCCGTCATCGGCG |
| 22550_9 | 10 | [C/T] | CACGGTTCTCTGGCAGGGTTCTGCTGCTCCTTTGGC |
| 22552_32 | 33 | [C/T] | AGCAGCACAGTAGCATTCGGTTGCTAAGCTACCATG |
| 22553_29 | 30 | [C/T] | GTTTTTTGTCTCGCAGTGTCTTGCAGCTCCGACTAC |
| 22555_29 | 30 | [C/G] | CAAGAGCACAGAGCAGCAATCTGCAGACACGAAAAG |
| 2256_9 | 10 | [C/T] | GAACCTCTCCGAGCAACAAAATGCTGTGAACTGGAG |
| 22566_4 | 5 | [C/A] | AACACACAGACTGCAGGTCCCTGCTTAGGCTAGACG |
| 22567_5 | 6 | [A/T] | TCAAAAGCTGGGGCAATGTGATGCAGGTCGATAGGA |
| 22580_10 | 11 | [T/C] | CTGGAGAATCTGGCAGCTGAGTGCTGTCCTGAGGCG |
| 22587_24 | 25 | [C/T] | TGTAAGATCTCAGCAGAGTCATGCCATCCTGGCACA |
| 22591_10 | 11 | [C/T] | TGGATTGGATCGGCAGATAATTGCTCCCAGCAGTCT |
| 22598_26 | 27 | [A/T] | CTCATGCATAAGGCACAGCACTGCCAATTCACCTAC |
| 226_33 | 34 | [G/A] | TGTTGGTCTGGTGCATTTTTCTGCCGGTTTAATGGA |
| 22600_31 | 32 | [C/G] | CCGTGATTAGTTGCACTTGGGTGCCAGGTTACGTCA |
| 22602_11 | 12 | [C/T] | GTGTGTGTGTGCGCACTGTTATGCTAAATCCAGCTC |
| 22603_34 | 35 | [C/A] | TTCACTCATAGTGCATCGTGGTGCCTTGTCCTCCCA |
| 22606_2 | 3 | [A/G] | CTAGTTACACAGGCAAGCCCTTGCCTGATCCCACAC |
| 22608_1 | 2 | [C/T] | ACGCTGCCGGCTGCAGCTCTGTGCCATCTTTATTCT |
| 22609_32 | 33 | [T/C] | ACGATGTCATTTGCACGGGTCTGCCTAGGAGCTCTG |
| 22611_34 | 35 | [G/C] | CAGATGTGTCTCGCAAAAATGTGCCGCACATCCCGA |
| 22615_1 | 2 | [T/A] | CAACAGCATTCTGCAGTGACATGCCATTTCATCTCT |
| 22616_25 | 26 | [T/C] | GGGAGTGTGTTGGCACGGAACTGCCTGTACTCTGGT |
| 22618_1 | 2 | [A/G] | TAATCTACCAGGGCAGGGTTCTGCAGGATGACAGGA |
| 22619_6 | 7 | [C/A] | CTGGAACGTAGCGCACGAAGATGCCATTGACGGCAA |
| 22620_29 | 30 | [C/G] | ATGCATGCTCGAGCAGGCTGCTGCTGGCCCAGCTAC |
| 22625_25 | 26 | [G/A] | AGAGACACAACAGCACAGTATTGCAGTGGGCACTGG |
| 22629_10 | 11 | [C/T] | GGATCACTTTTAGCACACTGTTGCTTTTGAGCTTTT |
| 22630_28 | 29 | [C/T] | GAAATAGTGAGAGCAGCTCAGTGCTGAACCCATTCC |
| 22631_33 | 34 | [G/A] | GCAGTTAATGCGGCAAAGGACTGCAGTGGAAACGTC |
| 22632_15 | 16 | [T/G] | TCGCATGTTTCTGCATTCGAATGCGGAGAGATTTAC |
| 22633_6 | 7 | [C/T] | AACAGACGGGTGGCATGGTGGTGCATTGGGAATGGT |
| 22634_2 | 3 | [A/G] | TAAAAACATGTAGCAGCACCCTGCATCACCTGAGAG |
| 2265_25 | 26 | [G/A] | GTAAAACAAGAGGCACGTGTATGCCGAGACGGCGGC |
| 22650_10 | 11 | [C/A] | TTACAGCTGACTGCAAACATTTGCACCCCTGTCATC |
| 22662_34 | 35 | [T/C] | CCAACATGCAGCGCACTCAATTGCTTGGGGCCCCTG |
| 22667_31 | 32 | [T/A] | CCTCTTTTCACAGCAAATGCATGCCATGGGATTCCA |
| 22668_4 | 5 | [C/T] | TTTTCAACCATCGCAAATCACTGCTAGGAGACATGT |
| 2267_28 | 29 | [G/T] | TCCCTAACACTGGCAAATTCATGCCACAGATTACGT |
| 22671_26 | 27 | [C/T] | CCAGTGAACGCAGCATGCTAGTGCTTTCAAACACAC |
| 22672_15 | 16 | [C/T] | TTGCTGAAAATAGCACACGGTTGCTGTGGAAACCAT |
| 22675_17 | 18 | [T/C] | ACGGCACAGGCTGCAAATGTATGCACACGCAAATGC |
| 22678_1 | 2 | [T/G] | GTTTTTAACCGAGCATGCATTTGCATAACCGCTATT |
| 22680_24 | 25 | [C/A] | TGGTGTACAAAGGCACAATGTTGCCAATGTCCAAAT |
| 22682_26 | 27 | [C/T] | CAGCAATGCACAGCATCACTGTGCCACCACCATGTT |
| 2269_18 | 19 | [T/G] | AACATGGTGGTGGCAGTAGTATGCTTTGGAGCTGTT |
| 22690_31 | 32 | [T/C] | AACTGTCAGGTGGCACAGTGTTGCCACAGGTTGTGT |
| 22698_30 | 31 | [G/A] | TGAGTAGCATGGGCATCTCACTGCTGCAGGGTTCAG |
| 22701_9 | 10 | [G/T] | AGTAATCCCGCAGCAACCAGATGCAAACGTTTAACT |
| 22703_15 | 16 | [G/A] | AAACCTGTTCCAGCAGAACAATGCACTGTGCACAAA |
| 22705_2 | 3 | [A/C] | TAAAATAAGCTTGCAACCAAGTGCCACGTCTAAATG |
| 22714_34 | 35 | [T/A] | TGAGTCATATCTGCATAAGGTTGCACAAGCAGCTTA |
| 22717_18 | 19 | [C/T] | TAATAACACGGAGCATCTCTCTGCAGACGACCCTCA |
| 22718_4 | 5 | [G/A] | TGGAGCAGGTGTGCAGAGAGCTGCGAAGTCTCTACG |
| 2272_16 | 17 | [A/T] | CAGTGTAAAAACGCATAATTGTGCCGTTGATGCATC |
| 22721_9 | 10 | [C/A] | TGAGACACACACGCATACACATGCATACACACACAC |
| 22726_2 | 3 | [T/C] | TTTTTCCCTCTGGCAGAACACTGCACACAAAATCTA |
| 22728_6 | 7 | [G/A] | CACTGCACTGAAGCATATGGCTGCATCTCCTCCCTC |
| 22730_4 | 5 | [G/A] | GGAGGAAACAAGGCATGCAATTGCAGTCACCATCAC |
| 22731_19 | 20 | [A/G] | GTCAGGACAGACGCATCACACTGCTTTCAAACGCTT |
| 22732_1 | 2 | [G/A] | AGTTCCACCTACGCACGCAACTGCTTCTCATGTGCC |
| 22737_32 | 33 | [C/T] | GCACTCTCCGTGGCAGAGTTCTGCCGCTCGGACGCG |
| 22739_2 | 3 | [G/A] | CTGTCTGGCTCGGCATCTTCCTGCGCTTCTGAAGCT |
| 2274_9 | 10 | [A/G] | AATCATGTGACTGCAGTATAATGCAAGAAACACGCA |
| 22751_30 | 31 | [G/T] | GTGGACCCTCTGGCACTCGTTTGCTCGCTCGGTGTT |
| 22752_25 | 26 | [C/T] | CCCTTGACCAAGGCACCTAAATGCTCCCTGGGAACT |
| 22755_33 | 34 | [G/T] | CCAGACGACTCTGCATGAACCTGCCCATGTCTGGCT |
| 22766_29 | 30 | [C/T] | CCTCCACCGTCAGCAAACACCTGCAGTTCCATTCGA |
| 22773_33 | 34 | [A/T] | AGTGAGAAGAGAGCAAACTGCTGCCAGGTGTACATT |
| 22778_29 | 30 | [T/C] | TACTGGCAGGTAGCAGAGAAATGCGTCAGTGCTCCT |
| 22784_25 | 26 | [A/T] | CCATGATGCCCTGCACACGCGTGCATTACATCATAC |
| 22785_30 | 31 | [G/A] | TTGGCATAGCCAGCAAGAAAATGCCTTTCCGCCGCT |
| 22788_24 | 25 | [C/A] | CTGGGTTGCTGTGCACTAGGTTGCCTACTGCTCCAG |
| 22793_26 | 27 | [G/C] | CCCCAGCCTCTGGCAATGCCATGCAGGAACATGTTT |
| 22797_27 | 28 | [G/A] | CATGGGGTTGTAGCAGGACAGTGCTGCGGAGTCTGC |
| 22799_29 | 30 | [A/C] | ACCAAGTAAACAGCAGTCCTGTGCTCACACACTGAC |
| 228_29 | 30 | [C/T] | TTAAGCTAAAGTGCATTGTGTTGCAGCTCCTCCTGC |
| 22806_8 | 9 | [T/C] | GTACCCCCTGAGGCACTGCTCTGCCTGCGCAAAGCC |
| 22811_3 | 4 | [G/A] | GCCGTCCGCATTGCAAAGTGCTGCCTGCGCCAGACT |
| 22812_9 | 10 | [G/A] | GATCAGCGTGAGGCAGGAGTGTGCTTTATATAGGAC |
| 22817_15 | 16 | [G/A] | TATGATGGCTCAGCAGAACACTGCATCTCTCTTTAT |
| 22818_33 | 34 | [C/A] | GAGAGAGAGAGAGCAATCCGATGCAAAATCCAACAG |
| 22824_5 | 6 | [C/T] | TATAACGATGCCGCACATCCCTGCTGGGTTTTCTAC |
| 22825_7 | 8 | [G/A] | TTGTGTGGAATTGCACTGGGTTGCAGTAAAGGTGAC |
| 22826_5 | 6 | [C/T] | GTTTTCCCTAGTGCAGGACAATGCCTGGTCTCATGT |
| 22837_24 | 25 | [A/C] | GGGCTTAAACTGGCAACCTTCTGCATGCCAAGGTAG |
| 2284_32 | 33 | [A/T] | AAGCCAGTGAAGGCAGTGTGATGCCTTCTTTAAAAC |
| 22842_10 | 11 | [T/A] | GTTGGGTTTCTGGCAGAGGTTTGCGTAGCTGAAGCT |
| 22843_19 | 20 | [G/A] | GTAAAGAGGGTGGCACTGCGGTGCCGCTGGTAGTGA |
| 22844_25 | 26 | [G/A] | ATTGCATTCACTGCATCCTTCTGCAGAGACGACCTG |
| 22846_11 | 12 | [C/A] | GAGAGAGAGAGCGCAAGAGTTTGCCATTCCAAACCT |
| 22847_33 | 34 | [C/T] | GCTGCACACAGTGCATTGATTTGCCCCTCCTTTCCC |
| 22848_1 | 2 | [G/T] | AGCAGTGCCATAGCATTATGCTGCCACCACAAAGCT |
| 22857_27 | 28 | [G/A] | TGGGTACAATTCGCACACCGATGCAAAGATGGTGAG |
| 2286_26 | 27 | [C/T] | GCTGTAGTTCCTGCAGTAGAATGCTTCAATCCCTGA |
| 22862_16 | 17 | [T/G] | CGTGGCCCTGGGGCAGTAGGATGCAGTAGTGTGGCT |
| 22863_11 | 12 | [G/A] | GCCCAGTCCTGGGCATGGGTATGCTGGTGTGTAAAG |
| 22865_15 | 16 | [A/C] | AAACAAAGTATGGCAAACAGGTGCACATGCGTGTGT |
| 22866_24 | 25 | [G/A] | GGGAGTGCGACTGCATGAATGTGCGAGTGTGTGTGA |
| 22868_24 | 25 | [A/C] | CGCAGATGTTTCGCACAATGGTGCAGATTTGTTTTT |
| 22874_19 | 20 | [T/C] | GAGTTGGTCTACGCATACATTTGCCGCTCTTTTCTG |
| 22875_34 | 35 | [G/T] | GGAGAATGCATGGCACCTGGATGCCTTCTGAGATGA |
| 22876_15 | 16 | [C/G] | TGCTGCAGGGAGGCACAGCGGTGCAGCAGGTAACTT |
| 22877_19 | 20 | [C/T] | TGTGCATCAGAAGCATTTACGTGCCGTCTGCTATTA |
| 22880_24 | 25 | [A/T] | AGATGACACATCGCAAATCGTTGCATGCTAGCACGC |
| 22884_17 | 18 | [G/C] | CTGGAGTGATGTGCAATGAAGTGCTGTGTGTGTGTG |
| 2289_31 | 32 | [C/T] | GACAGCGGGTAAGCAACCGGATGCAAAGAAACACCA |
| 22894_20 | 21 | [C/A] | TTACCCCGGTTTGCAAACCGCTGCGGCAGTTTCCAC |
| 22904_33 | 34 | [C/T] | GAAACACTTTGTGCAATTTGTTGCTGTTTGGACCGG |
| 22909_1 | 2 | [G/A] | CGTCATTCTGCTGCAGGACGTTGCTGTAGAAATGCA |
| 22915_3 | 4 | [A/T] | ATCACGAAACTCGCAATAACATGCTCCGCTTCTGAC |
| 22916_19 | 20 | [C/A] | GGCACGGAGGTGGCAGCGTAGTGCTGTGGGGTGTTT |
| 22917_26 | 27 | [C/T] | GGATGATTGTCAGCAGGTTAATGCATCCTGCCACAC |
| 22939_30 | 31 | [C/A] | AACCCGACAGCAGCAATTAATTGCGTCACACACAGC |
| 22942_10 | 11 | [A/C] | CAGAGAACTGATGCATTCTGCTGCATTCCCAAGTGA |
| 22943_1 | 2 | [G/A] | GGGATGGATATGGCAGAGCATTGCTGAGCAGGAATT |
| 22944_32 | 33 | [G/A] | TATGGCCGATAGGCATACAGCTGCAGTACGGTGAAT |
| 22945_6 | 7 | [C/T] | TGTGCTCCTTGTGCAGGCCAGTGCTTATGGACAAGC |
| 22947_29 | 30 | [C/T] | TGTGAGGATTGTGCAGCTTGTTGCACTGTCCCTTTA |
| 22948_27 | 28 | [G/A] | ATGCAGATCAGGGCAAGAAAGTGCAGTGACCTCCAT |
| 22951_6 | 7 | [T/A] | AAGAAGTAGTGAGCAGAGAACTGCTGCTTAAGTTGC |
| 22953_4 | 5 | [T/C] | TTCCTGCGGTGTGCATCAAGCTGCGTGCGCGTGTGT |
| 22954_33 | 34 | [T/C] | CGCAGACACTCTGCATTGCTCTGCATCATGAATTTC |
| 22959_5 | 6 | [C/T] | GCGTTCAGTAGCGCATTGCTCTGCGTCCTGTCTCAT |
| 22960_24 | 25 | [C/A] | GAATGAGTTCAGGCACCTGCGTGCCGTGCAGATGAA |
| 22969_24 | 25 | [G/A] | GCCGAGCCCAGTGCATTATTCTGCGGAGAGACAGAG |
| 22972_20 | 21 | [C/T] | GAGTTATGGCTTGCAGGATTCTGCCTCCCTGCCTGG |
| 22978_30 | 31 | [C/T] | GACATTTGGCCTGCACCCAAATGCAGACATCGCCAG |
| 2298_7 | 8 | [A/G] | GAATGTGAGTTGGCAGGAATGTGCATGTTGTGTAAG |
| 22981_11 | 12 | [A/C] | CACAGGGTCAGAGCACAAAAGTGCCAGCTTGGCAGT |
| 22989_9 | 10 | [C/T] | GTGAACTGTCAAGCAGTACGCTGCAGGAGTGGTCAT |
| 22990_29 | 30 | [T/C] | GGTGCGGTTTGTGCAGCAGCATGCATTCTTAAGAAT |
| 22992_29 | 30 | [G/A] | TTTCTGTGCTGAGCAAAGGAATGCCCGGTGAATTTC |
| 22994_9 | 10 | [G/A] | CACTCACGGAAGGCAACAATGTGCAATCCTCTTCAA |
| 23011_28 | 29 | [G/A] | TGTGCAACCCTGGCAAGAGAATGCTTTTGAAGTATA |
| 23016_4 | 5 | [G/A] | CGAGGAAGCAAAGCACTCTTCTGCTGCCAAGACGAG |
| 23023_1 | 2 | [C/A] | GCCTCGGCTTCAGCAACATGGTGCTGGCCAACATCT |
| 23024_29 | 30 | [A/G] | TCTCTCTCTCTCGCAACAGAGTGCAGAGTAATCCAT |
| 23025_3 | 4 | [C/T] | TAACGAAGGCATGCAAACCAGTGCACACAGGCACCG |
| 23026_9 | 10 | [G/A] | AGACTTGCTGCTGCACTGCACTGCACTTTTGCAGAA |
| 23028_15 | 16 | [G/A] | ACGCTCCTCAGAGCAGCGCACTGCTGCCGGAATCGA |
| 23029_20 | 21 | [A/T] | ATCATGTATCTGGCAGCACCATGCTAAGAGTCACTT |
| 23032_29 | 30 | [T/C] | CCAGGACGATGAGCATCCCGCTGCTCGACTACATAG |
| 2304_27 | 28 | [A/T] | GATTGCATGCTGGCATAGAAATGCCATAAGGAGGAG |
| 23040_16 | 17 | [G/A] | CATGACGGAGGAGCATAAAGCTGCGAGTGTGAATGC |
| 23042_20 | 21 | [T/C] | CCCCAGAGGGCTGCACTTTATTGCTGGCTCGTGAAT |
| 23044_34 | 35 | [C/T] | GACAACATGGCAGCAATGCACTGCAACTAGGCACCG |
| 23047_6 | 7 | [G/A] | AGGACAGAGAGGGCATGAGAATGCGTTTTACCACAG |
| 23051_8 | 9 | [T/G] | ATTTGCATTTTGGCAGCACTCTGCTCCTGGCACTTG |
| 23055_34 | 35 | [A/G] | GTGCTCCAGTGTGCACTGTTTTGCCATATGTGTTAT |
| 23057_29 | 30 | [A/G] | TCTTGAAAGCTTGCACAATCTTGCCTGGCAGGCGTG |
| 23065_2 | 3 | [T/C] | GCTTAATACACAGCATTGACATGCACATCCTGCCAG |
| 23068_28 | 29 | [G/T] | TGAGTTGTTCCAGCAGGGGTCTGCTGAGGGTCCTGT |
| 23081_19 | 20 | [G/A] | GATCACGTAACTGCATCAGGATGCGAGAAACTCCTC |
| 23088_33 | 34 | [A/C] | TTTGACAAGTACGCAGCCAGTTGCGGAAAATTAACA |
| 23089_16 | 17 | [G/T] | TCAAGCAGTCATGCAAGCAAATGCCAACAACACAAC |
| 23090_10 | 11 | [C/T] | CTGTGACACTCTGCATCTTAATGCCAGCTCGGTTGT |
| 23091_3 | 4 | [A/G] | CTTAAATAAAAGGCAAGGTCTTGCTCTGCTGACCAA |
| 23092_20 | 21 | [C/T] | TGCTGAGAAGGAGCAGGACTCTGCATCACCGCCCGG |
| 23095_8 | 9 | [C/T] | ACATCACGCTCAGCAATCCACTGCTCCAGATCTTCC |
| 23097_29 | 30 | [A/G] | CCACTGAAAACAGCATAATGTTGCCACCAACATGTT |
| 23101_30 | 31 | [G/A] | CAGGTGCCCAAAGCACCCAATTGCACTTACGTCACT |
| 23102_27 | 28 | [G/A] | ACAGATGAATTGGCATCTTTCTGCCTTGTGTACCAT |
| 23107_17 | 18 | [C/T] | TGCATGGGCACAGCAGACGGGTGCAGTGTGGGCAGA |
| 23108_24 | 25 | [C/G] | ACACAACCTGTGGCACCATTGTGCCATCATCCGAGG |
| 23109_32 | 33 | [T/C] | GGCCCTGGTTGAGCACCATGATGCGGTCACAATTGA |
| 2311_33 | 34 | [T/G] | GAACCTCATTTGGCATTGCCATGCTGGTATAGGTTT |
| 23110_24 | 25 | [G/A] | ACTTTGGCAATAGCAGCGCAGTGCGTCGGAGAAAGC |
| 23111_24 | 25 | [A/T] | CTGTACTGTCTGGCAGGTTCCTGCAGAGTTCAGTCA |
| 23112_24 | 25 | [C/T] | GCTCAGGGGTGGGCAGTCTAGTGCCTTGTCAATTAA |
| 23113_28 | 29 | [C/T] | ACGCCAGCAAGCGCAGGATCCTGCCCACCGGCGTCT |
| 23115_2 | 3 | [C/T] | AACACCACAGTGGCAGTCATTTGCTCTAAGAGGGAG |
| 23118_4 | 5 | [C/A] | CAGACGTTGCTAGCACGGGTGTGCAAACAAAGTGCT |
| 23119_27 | 28 | [T/C] | TTAGGAAGCCCAGCACGGATGTGCCACTGCTGTGAT |
| 23121_19 | 20 | [G/C] | AGCGGGGGATGGGCAAAACGATGCCAAATTTGCTGA |
| 23123_9 | 10 | [C/T] | AACACTTTTCCAGCACGGCTCTGCCTTCCTCTTCTG |
| 23125_25 | 26 | [A/G] | TGTTTACCCAGGGCAATTTGCTGCGGCAGGTGAACT |
| 23126_28 | 29 | [G/A] | CTGCTTTAGCCAGCAGGGTTTTGCCACAGCCTGGAG |
| 23129_9 | 10 | [G/A] | CAGCTATAAGAGGCAGAAAGATGCCTTTGCACTGTT |
| 2313_1 | 2 | [G/T] | CGCCGCCGTAATGCAGCATGTTGCTAATTTGTTTGG |
| 23132_28 | 29 | [C/T] | TACTTCTGGAAGGCATAATGCTGCACACCGGCCCAT |
| 23137_15 | 16 | [G/C] | CAGCAGATTTACGCAGCCTGGTGCAAAGAAAGAGAC |
| 23139_7 | 8 | [T/C] | ATAGAAATGAGCGCAGCACTGTGCCCGCTGCTATGC |
| 2314_20 | 21 | [C/A] | TTGGTTTGTGTGGCATTGTACTGCTGCATGACCCAC |
| 23142_17 | 18 | [C/T] | GAGTGTGTGTGTGCAAGCGTGTGCGAGTGTGTGTGT |
| 23148_16 | 17 | [G/A] | ACCTGACATAGTGCATGAACCTGCATCAACGGATGA |
| 23150_7 | 8 | [T/G] | GAATTACGCCTTGCAGCGTTATGCAAGTGCTGAAGT |
| 23155_24 | 25 | [A/G] | GACACGGTTTTGGCAAGTTTGTGCATCGTGTGATGA |
| 2316_30 | 31 | [C/T] | GCAGTGATCATTGCAGTTCTTTGCAAGCTTCTGTTG |
| 23161_5 | 6 | [C/G] | GTCTGCGTTCTTGCATGTTTTTGCGGTTGTGTATTT |
| 23162_32 | 33 | [G/A] | ATTCAGGACCAGGCACCATTCTGCAAAATGATGAGG |
| 23165_1 | 2 | [C/T] | GCGCGGCGAAGCGCATGGCCCTGCTGATGGCCGAAA |
| 23167_7 | 8 | [C/T] | TTTTCGCTGCCAGCACATACGTGCTTCTCAACTACA |
| 23171_29 | 30 | [T/G] | GGTAACTGTATAGCAGTTTTTTGCCCCGGTGCATCA |
| 23174_5 | 6 | [T/C] | GCTGATCGGATTGCACACGTGTGCGACTCTGTGATT |
| 23177_3 | 4 | [C/T] | ATTCACCCAACAGCATTGAGGTGCTCATGTGTACAA |
| 23181_9 | 10 | [C/T] | ACACACTGACGTGCATACGTTTGCACACGTGTGACC |
| 23182_34 | 35 | [G/A] | TACAGACGCAACGCACAATGCTGCTGATCAAAGCGA |
| 23188_16 | 17 | [T/G] | CAGCTTCATTTTGCAGTGTAATGCCCAGGGGCCACT |
| 23190_24 | 25 | [A/C] | AGCGTTATGAGAGCATGTCAGTGCATGCTCAGTGCG |
| 23193_24 | 25 | [G/A] | GGAGAGTCATAAGCAAATGATTGCGTGCCCAGACAT |
| 23194_32 | 33 | [C/T] | CCCTCCTGACCTGCAGGCAGCTGCAGCTTTTTCACA |
| 23196_33 | 34 | [T/C] | GGTGTCTAAGCAGCATCGTGGTGCAGGAATGCATCT |
| 23202_31 | 32 | [T/A] | CTAAACCACACGGCACACGATTGCGTTAATTTTTAC |
| 2321_10 | 11 | [C/T] | TCACCTGACACGGCAAAACAATGCTCTCACCCACTG |
| 23212_28 | 29 | [G/A] | GTATGTGTGTGCGCATGTGTGTGCGCGCGTGTGTCT |
| 23214_18 | 19 | [G/A] | CAAAATGAGACAGCACACGCTTGCGCCAGAGCTACA |
| 23215_30 | 31 | [C/T] | TTTCTGTAAGCAGCATCCAGGTGCTGACATCGCTCA |
| 23217_10 | 11 | [G/A] | GTGCTGGAATGGGCAACCTTGTGCCAAAAGCCATGT |
| 23219_9 | 10 | [A/T] | CATTGTATCACAGCAGTTCATTGCAAAGGCAGGTTC |
| 23220_33 | 34 | [A/C] | GAGTGGTGGTAGGCACCGGACTGCGTCGAGTGCACA |
| 23222_17 | 18 | [G/T] | TGTCTGTCTCCTGCAAAGGGCTGCTCATTTCCCCGT |
| 23223_28 | 29 | [G/A] | TAGACAAAGGGAGCATTGTTTTGCTACCGTCATGTG |
| 2323_7 | 8 | [C/T] | ACCCCCTCGATGGCAATTGGCTGCAGAGAGGGCCTT |
| 23241_3 | 4 | [G/A] | AACGTGATGGAGGCATTTTGATGCTCAACTCTGGAT |
| 23243_20 | 21 | [A/T] | TAGTAAAGGCAAGCATGGCTATGCAACACAAACAGG |
| 23246_24 | 25 | [T/A] | GGAAAGTGACTGGCAATACCCTGCTTTTCTTCTTGT |
| 23247_32 | 33 | [C/T] | TTAAGGAGTTTTGCACTTTGATGCCAAACACACGCA |
| 23249_18 | 19 | [T/G] | TCCAAATGCCAAGCAGACTTGTGCGAGGGTTTTACG |
| 23251_19 | 20 | [T/A] | CTAAAATAACGTGCATCAGTGTGCATGTAGCAGCAA |
| 23258_19 | 20 | [G/A] | TGTTAAAAACCAGCAACGTGATGCATCGTGTTGGCT |
| 23269_3 | 4 | [C/G] | TTTCGCTGGCAAGCAGCAGCCTGCAGTACGAGACTG |
| 23270_20 | 21 | [T/C] | CTCCTTAGCATTGCAGATGCTTGCTAACTCATGCTA |
| 23272_33 | 34 | [C/T] | CAAGGGTGCAGTGCACAGGGCTGCCCACCACTTCAG |
| 23275_27 | 28 | [T/C] | ATACACTTTGTGGCACCTGGTTGCATTTAGGCTGAG |
| 23278_10 | 11 | [G/A] | GGAGCCCTAAGAGCAACACACTGCAGCTGGATCGAT |
| 2329_29 | 30 | [C/G] | GGTGTGTCAACTGCAGCTGCATGCGACAGCGGAGGA |
| 23291_33 | 34 | [C/A] | GGGCAGAAAACAGCAAACGCCTGCACAACTACACCG |
| 23293_26 | 27 | [G/A] | GGGGAAATTCGGGCAAATTCCTGCAGGATGGTGTGG |
| 23297_24 | 25 | [C/A] | AAAGCTTCCACAGCAAAACCCTGCCGAGTCATGGCT |
| 2330_24 | 25 | [A/G] | TACCCAGGTGCGGCATATGATTGCGGAGGGAATGAT |
| 23300_28 | 29 | [A/T] | ACCTCTCCAGAGGCACCGTGTTGCTCTCAGACTCTG |
| 23302_20 | 21 | [C/T] | GAGGCTTCTTCAGCACTCATCTGCTGCTCTGAGTGT |
| 23321_28 | 29 | [G/A] | CCGTGTGCGTGCGCAGGTGGTTGCGCAGGTGGATCT |
| 23324_5 | 6 | [A/T] | GTAACAGCAGCTGCAAAGCTTTGCAGTCAGACACTG |
| 23326_4 | 5 | [G/T] | CAGCGCTGTGGTGCAGGACATTGCCAGCGCTTTACA |
| 23328_1 | 2 | [G/C] | GGAGTTACAGTAGCACCTGCCTGCGTCTGACCCCGC |
| 23332_31 | 32 | [G/A] | GCAGGACAAAGAGCAGGACCATGCCCACTAGGATGC |
| 23335_1 | 2 | [C/A] | CCTGACCATGCTGCATGTGGCTGCAAAACACGCCTA |
| 23336_7 | 8 | [A/G] | AACTTTAAGTTTGCATTTGAGTGCGTGAGTGGGACT |
| 23339_18 | 19 | [G/A] | GCCGACGTGAAGGCAAACGTGTGCTGAGACATGACA |
| 23340_33 | 34 | [C/T] | TGACACCACTGCGCAACAGTGTGCATCTTCTCTCCA |
| 23341_18 | 19 | [C/T] | ACACACACACGTGCACTGTGCTGCACACACTCACAC |
| 23342_8 | 9 | [C/A] | TTCTATCACACAGCAGTATAGTGCTGACACCCACCT |
| 23344_26 | 27 | [T/G] | AACAGCCCCAAAGCATAACACTGCCATCACCATGCT |
| 23348_19 | 20 | [C/T] | ACGGCCAAACTGGCAATGACGTGCTGGAATCAGTTA |
| 23349_15 | 16 | [G/A] | ATGGCATCTTCAGCAGAGTTTTGCTGCATGCTTGAG |
| 23354_2 | 3 | [G/A] | GAGTTTGGAATGGCACAGTGTTGCCAACAGTAGTGC |
| 23362_26 | 27 | [A/T] | GCAGAAGGCGATGCAGGGAAGTGCAGAGGGGTTGAG |
| 23364_7 | 8 | [A/T] | GAGAGACAGAGAGCACACAGCTGCTAAAATGTGCTA |
| 23366_24 | 25 | [C/G] | TGCTTTTAATGTGCATTGTATTGCCGCGATGTGTTT |
| 23370_24 | 25 | [A/C] | CTGGAGAGTAGTGCAGCAGGATGCAGGCTTGTACAG |
| 23371_7 | 8 | [T/G] | TGTGAAGTGTGGGCAGTCTCCTGCAGGTCTTATGGG |
| 23373_30 | 31 | [C/T] | TAAAGCACATAAGCATCTCCATGCCCCAGGCTCTCA |
| 23375_15 | 16 | [C/T] | CAATAAGACTTTGCACGCTCTTGCACAAGTGTCATG |
| 23377_20 | 21 | [C/T] | TAAAAACAGTAAGCAAAACACTGCAAAGCTGTCCTC |
| 23385_27 | 28 | [T/C] | TTACAGGTAAAAGCAGACAACTGCGTTTGTGCAGAT |
| 23388_30 | 31 | [G/A] | TAGTTGCTTTTGGCACATGATTGCACACACGCCCTT |
| 23393_25 | 26 | [G/A] | AAAGTAGCTGAAGCAAATCAATGCGGAAGAGCACGA |
| 23397_25 | 26 | [T/C] | CCAGCTACCAATGCAAATGAATGCTTAGCTCCTGAG |
| 23400_24 | 25 | [C/T] | TGAATGGTTGCTGCAGGAAATTGCTCTGACGAACCA |
| 23402_25 | 26 | [G/A] | CTCTGACTCTTGGCACGATTCTGCAGAAACTTTTGC |
| 23404_16 | 17 | [G/A] | TTCTGTCTACATGCACGCATATGCTCACACACATAC |
| 23406_7 | 8 | [A/G] | GTGGAGGGGGTTGCAGACATATGCTGATGTATCACA |
| 23408_24 | 25 | [C/T] | GCTCACAAATGCGCACCAGGCTGCCCCAAACAGAGA |
| 23411_1 | 2 | [C/T] | TCCTGTGAATGAGCACCTGACTGCATTTGTGATGAC |
| 23413_1 | 2 | [T/G] | ATGCATTGCGCAGCAACATGTTGCTCAACACACACA |
| 23414_10 | 11 | [G/T] | TGGACACACAGTGCAGACGGCTGCATTAGGAGGAGA |
| 23425_20 | 21 | [T/C] | CAGTATTTGGTCGCATCACATTGCATCATCCAATAT |
| 23428_11 | 12 | [T/C] | GTATGCAGAAGTGCACACACATGCACAACAGCAGCT |
| 23432_2 | 3 | [C/T] | TTCCCCACTGTTGCAGGCCACTGCTGTGTACCAGGC |
| 23435_4 | 5 | [T/C] | GTGCTGCAGACGGCAGGGGGCTGCTCCTTTAATACT |
| 2344_28 | 29 | [C/T] | GTATTTGCGTAGGCATTGACATGCACCATGTAGTCG |
| 23443_2 | 3 | [G/C] | AGGAAGTCAGTAGCAGACAGGTGCTGAAGCAACGTG |
| 23445_31 | 32 | [C/A] | GCTGCTGGCTCAGCAGTGACATGCTACAAAACAAAA |
| 23446_17 | 18 | [A/G] | GGATACAATGAAGCACGAAAGTGCCACGGCATCACT |
| 23447_28 | 29 | [A/C] | GTGTATCTGTATGCATGTCTGTGCGTGAACATGCCT |
| 23452_5 | 6 | [A/T] | GATAAAGCGTGTGCAGACAGCTGCTGGTTCTGGATA |
| 23454_34 | 35 | [T/A] | TCCAGGAATGCAGCACAGGCATGCGTTGGCTGTATT |
| 23456_16 | 17 | [G/A] | AGCCACACACAGGCATGAGTGTGCCACCAGGATGAA |
| 23460_11 | 12 | [G/T] | GGATCCCATCCGGCATCATAATGCACAGGACAGGAA |
| 23461_24 | 25 | [G/A] | GAAAAACTTGCAGCACTTGTTTGCGAACCCAAAGAT |
| 23462_10 | 11 | [A/C] | AATCGCTCTAAAGCACATCTCTGCCCCGTGATTAGC |
| 23465_19 | 20 | [G/A] | TCAAACCTCTGTGCAATGCGATGCTGCACTACAGTA |
| 23473_4 | 5 | [C/T] | AACCCTCGGAATGCAGTGACTTGCTTTTACTGGATA |
| 23479_2 | 3 | [T/C] | AATGCATGCCCTGCAGGTATTTGCACTACAACACCT |
| 23485_6 | 7 | [G/C] | TGGGTGGTGACCGCACGCCACTGCCATGTTACGTAA |
| 23487_27 | 28 | [G/A] | GTGCGACAAAAGGCAGATTCCTGCTCGGACCGTCTT |
| 23493_31 | 32 | [C/T] | GGTCATCTAACTGCACCAAAATGCTGCAGCTCGACT |
| 23496_10 | 11 | [G/A] | AGCGCAGGGCGAGCAGCTGGCTGCGCAGACGCAGGT |
| 23499_20 | 21 | [C/T] | GTATGCGTCTGTGCATCATTCTGCACAGAAGTCACT |
| 2350_24 | 25 | [T/A] | TTGTGCATATCTGCAATGTCATGCTCAAACAGGTTC |
| 23505_34 | 35 | [A/T] | TAAAGACTGATGGCACCAGACTGCAATGACCAACAT |
| 23508_5 | 6 | [C/T] | TCACTCGTGCGTGCACAGCTCTGCTCCGCCTCGGCC |
| 23513_33 | 34 | [T/C] | AAAGACTGTCGAGCACACCCTTGCCTCTTTGGGTAT |
| 23514_8 | 9 | [C/T] | ACAACGCTCCCTGCACGCCTATGCATTCATCTATCT |
| 23516_34 | 35 | [T/C] | ATGTGCCTGTATGCATGTGTTTGCTTGGTGTGATTA |
| 23517_10 | 11 | [G/A] | GTGTTTGTCCGTGCAGTGCGGTGCGTTGCGGTGTAG |
| 23522_25 | 26 | [C/T] | GAATGCGATCTGGCAGCAGGTTGCACTGTTACACCC |
| 23523_16 | 17 | [G/A] | ATGGGAATACATGCACGAGCCTGCTAAAGGCACCAA |
| 23527_5 | 6 | [A/T] | GATGTTAGTATTGCATGCAGCTGCTCAGACATGGAA |
| 23528_29 | 30 | [A/G] | CGATGTGTCAGAGCAGACATGTGCTCTGCATCTATT |
| 23530_1 | 2 | [C/T] | TCGCAAGGTGTGGCATGGTGGTGCCACAGGTAGTGC |
| 23532_32 | 33 | [C/T] | CACATCGTGAGCGCATCAGCATGCCTCATTCTTTCA |
| 23540_25 | 26 | [T/C] | GAAAGTAGACAGGCAAAAAGATGCCTCTCCCTGACA |
| 23544_10 | 11 | [C/G] | TCCCAGTGCCCTGCAGGCTGATGCCTCTCCCATGAC |
| 23549_26 | 27 | [G/A] | CATAAAAGTTGTGCAGCTGCATGCACGGATTACATC |
| 23550_5 | 6 | [C/A] | ACCTCCAGCTCGGCAGCCAGCTGCTGCGCTTTCATT |
| 23551_33 | 34 | [G/A] | ATTGGGTGCAGGGCAGGAATGTGCCCTGGGCAGGGT |
| 23553_6 | 7 | [C/T] | ATGAAACAAATGGCAGTGTTATGCTCGGTTCTCTCT |
| 23559_7 | 8 | [G/A] | TGCTATAGAAGAGCAGGATCCTGCTGAGTCACACCA |
| 23561_27 | 28 | [A/T] | ATTATCAGCTGGGCAACACATTGCCTGAGTTCATGT |
| 23562_5 | 6 | [G/T] | GCACGGGCAATAGCAATCCTTTGCTTCTGCCCACCA |
| 23568_25 | 26 | [T/C] | ACACACACACAAGCAGTTAGCTGCATGTGACAGGTA |
| 23570_7 | 8 | [G/A] | TCCATTCGGAGTGCATGGTGATGCCCGTCTCCATGG |
| 23572_4 | 5 | [C/T] | CATTCAAGTACAGCAGCAGGTTGCGTGGTGAAGTGA |
| 23574_6 | 7 | [C/T] | ATAAGACCCACTGCAGTGCTGTGCTGGCTGTGCTGC |
| 23583_2 | 3 | [A/G] | ACAATGGATGTTGCATGTGAATGCCTGAAAAAGCAA |
| 2359_8 | 9 | [G/A] | AAAAGGATAAAGGCAATGAGGTGCCGCTGAGGTTGA |
| 23592_7 | 8 | [G/A] | CATGTCAGAATTGCATACGGATGCCATCTTCTTCCC |
| 23593_5 | 6 | [G/A] | GCACTGCCTGTGGCACCACTGTGCCACCCCGAAAAT |
| 23595_20 | 21 | [C/T] | ATGCTAGAGGCAGCACATCCCTGCACAAGTGTGTTC |
| 23597_16 | 17 | [G/A] | CGCTCTCAGACGGCACGAGGTTGCTCAGGTTCAGGA |
| 23598_34 | 35 | [T/G] | GGCCACGGCGGAGCATCGGATTGCACGAACACCGTG |
| 23599_15 | 16 | [C/T] | ATTGACAGTGTGGCACGTGTCTGCCTTGACAGAGGA |
| 23601_16 | 17 | [G/A] | GAGCGTTAAAAAGCACGCTAATGCCCACACGGGGAA |
| 23605_10 | 11 | [G/A] | CTACAAAACCGAGCAGGGAAATGCGTTACGCTCCGG |
| 23607_5 | 6 | [G/A] | TCCTCGAACAAGGCACCCAACTGCTCCTCGGGCACT |
| 23609_5 | 6 | [C/A] | TAACCCATCCGTGCAGTTTGGTGCAGAGAAGTTTGG |
| 23612_2 | 3 | [A/G] | AGAGCAGCTGAGGCACCGCTCTGCCAGGGGTCACCG |
| 23616_5 | 6 | [C/T] | TCTATCAGCACAGCACATCTTTGCCAGTTCTTCCAT |
| 23621_5 | 6 | [C/A] | TATGGCAGTGAAGCAGGCAGATGCAAGTTCACCAGA |
| 23623_3 | 4 | [A/G] | TTTAGGAGCCAAGCAGAGGTCTGCCAAAGTTAACAC |
| 23627_34 | 35 | [T/G] | GTCCAACATTTTGCACATCGGTGCCCCTCGGTGCTG |
| 23629_26 | 27 | [G/A] | TTGCATCTTACAGCACAGAGCTGCTGGAATCTCACA |
| 23631_6 | 7 | [G/A] | TCTGCCAATGCTGCATCTCGCTGCACACTGCACAAG |
| 23644_17 | 18 | [G/A] | CAGGCTCAGAGGGCAGAGCGCTGCACTGATGCTGCC |
| 23649_24 | 25 | [G/A] | ATTTAGTTCGTTGCAATGGGTTGCGTCACTGCAACT |
| 23661_5 | 6 | [T/C] | GCATCTCTGAATGCAGCACTCTGCAATGCTCTGATG |
| 23664_19 | 20 | [C/T] | ACCACGTCATGTGCATGCACCTGCATAGTGCATGTT |
| 23665_28 | 29 | [T/C] | GCCTCTCGTTCCGCAGCCAGATGCTCGCCATGAACA |
| 23667_2 | 3 | [T/A] | GCTGGATAGCGGGCAAGACACTGCTCATGACTATAC |
| 23677_29 | 30 | [G/A] | TTAGCTGCGACTGCAGCTGCCTGCACACCGCCCTGA |
| 23681_16 | 17 | [C/T] | TTTTATGTGTTGGCAGCGGTGTGCATTTCTGGGGCA |
| 23684_10 | 11 | [C/T] | TCTCTTTCATCGGCACGCGCGTGCACGAGCGAGGTG |
| 23693_9 | 10 | [G/A] | CATTGCACAGAAGCAGCATGATGCAAGTTTGAGTAA |
| 23695_10 | 11 | [C/T] | TTACAAATTTCCGCAGTCATCTGCCAACTGTAATTA |
| 23701_7 | 8 | [G/A] | CAACAGGACAGAGCAAAATTTTGCCTGCAGATCCCT |
| 2371_19 | 20 | [C/G] | GCTAACAGGACAGCAGGTCCATGCTGGCTGACAGGC |
| 23714_5 | 6 | [A/T] | TACAGACGTGATGCAGGAACTTGCTGACCCTTTGAC |
| 23727_33 | 34 | [A/G] | AAGTGAGACTAAGCACAGAGCTGCCTGAAAGCAAAC |
| 23730_6 | 7 | [T/G] | TGTGGTTCTGATGCATGATGCTGCATGAAGCAGTCT |
| 23736_26 | 27 | [C/G] | ATAGATAATTGGGCAAAAACCTGCAGCACAGATTCA |
| 23737_15 | 16 | [T/C] | TTCCTGTTGACAGCATGACCGTGCGGGACAGCGGGA |
| 23739_33 | 34 | [C/T] | CCAGGTGCTGATGCATTGAGCTGCTCAACACTTCCA |
| 2374_5 | 6 | [T/C] | CCCGGTAACCGAGCACCGAGCTGCTGCTGCCGGACC |
| 23741_24 | 25 | [G/A] | ACAAACATAATCGCACACGCGTGCGCTCAGCACACA |
| 23742_34 | 35 | [T/C] | GTGCACTAGCAGGCAAACAGATGCCGAGCAAGCCCA |
| 23746_30 | 31 | [G/C] | CCACTGGCTGCTGCAGCAGACTGCGCTGCTGTACTC |
| 23749_18 | 19 | [C/G] | TGACAGGCATGTGCAAGCCGATGCATTCTAGGATGC |
| 23751_9 | 10 | [C/T] | CAATGGGAACGCGCAGCATGCTGCACCCCGATTAGC |
| 23752_9 | 10 | [C/T] | GAGCGAGCCCGAGCATGAAGCTGCCATGCTGGACCA |
| 23754_34 | 35 | [C/T] | CCGCAGCACCTGGCAGACTGATGCTGTTTAAAGACT |
| 23758_33 | 34 | [G/A] | AGTCCAGAGCTAGCACTCGCTTGCTTGCTGCATGAG |
| 23764_6 | 7 | [G/A] | CCCTTAGATGGTGCACATTTGTGCTGTGTCCTGCCA |
| 23767_30 | 31 | [G/T] | GAATATGCTGATGCACATGGGTGCCTAAAGGCAGTC |
| 23768_30 | 31 | [C/T] | AAGCTGCTTAATGCATTCCACTGCGGTTTTCAGTCA |
| 23778_6 | 7 | [T/C] | GTGATTTGTGGTGCAGGAAGATGCCCAGGAGGAGTC |
| 23780_24 | 25 | [T/A] | GCTAATGTTATGGCAGATCATTGCTTGGCCCCCTAA |
| 23781_26 | 27 | [G/T] | CTTGTGACGCAAGCACGCAAATGCTAGCCCTTCCAG |
| 23782_11 | 12 | [G/T] | AACTATTCTGCGGCAAAACCTTGCGCCATCTGTGGA |
| 23783_29 | 30 | [C/T] | TTACACAGTCACGCAAAGCAGTGCACTAGCCCGTTA |
| 23784_11 | 12 | [G/A] | GTTCAACCACCGGCACACACATGCTTACCTGCAGTT |
| 23785_7 | 8 | [T/G] | ACTAACCTCACTGCAGACAAATGCGTGCATTGACAA |
| 23789_15 | 16 | [C/T] | GTCCGTGGGGGCGCACGCTTGTGCCGGAAGAGACCG |
| 23795_20 | 21 | [A/C] | AACACGGTGGTGGCAGTATCATGCTACAGATCTGAT |
| 23806_29 | 30 | [C/T] | AACACAATCACAGCAGGTTGTTGCCACCTCTCTACA |
| 23810_19 | 20 | [C/T] | AAACCTGCATTAGCATCTCCATGCAGTCAGTGCGGG |
| 23820_29 | 30 | [A/G] | ATGCACCGAGTTGCAGGATAGTGCACACGAGAAATG |
| 23826_10 | 11 | [A/G] | TTTGTTGTACAAGCATGTTGGTGCCACACCCATTCT |
| 23828_1 | 2 | [T/C] | ATGGCCCCCACAGCATGTGCATGCACTCGTCCTGCG |
| 23829_5 | 6 | [G/A] | CATGTGTTCACAGCAGGAAGATGCTTTGTGATGTGC |
| 23830_9 | 10 | [G/T] | TGTACCACTGTGGCACCACACTGCTCCGTAATTAAT |
| 23837_24 | 25 | [A/G] | TATGGGTCAGCCGCATACATGTGCAAGCTCTTGCTT |
| 23838_32 | 33 | [G/A] | TTGCTGTGTCTGGCACTGGACTGCTTGACTGTGAAC |
| 23840_8 | 9 | [A/G] | CCTATTGTACGTGCACAACGGTGCGATAAATCCCCT |
| 23842_3 | 4 | [C/T] | CTTCAGAGAGCAGCAGTTGTTTGCCTCTCTGCTTCC |
| 23843_6 | 7 | [C/G] | GTGTGTCTCTTCGCAGCGAACTGCCTCACTCACCCC |
| 23851_20 | 21 | [C/T] | GTCATCTTCTGAGCACCAGCCTGCCTGAAGGTCCTG |
| 23856_26 | 27 | [C/T] | TTGTTTGGAGAGGCAGTTTTGTGCTTCGTGCCCGTT |
| 2386_27 | 28 | [G/A] | CATTGTCTCACTGCAGGGTTATGCAATGTGGTGAAA |
| 23864_32 | 33 | [G/A] | AAGGAAGAAAAAGCACAGTCTTGCATGACAGCGTTG |
| 23865_9 | 10 | [G/A] | GTCCTGTTGAAAGCAGGCTTCTGCACATGCTCAATA |
| 23878_10 | 11 | [C/A] | GCCTTTCCCGCGGCACTGTGCTGCACAGAGCTCGGA |
| 2388_20 | 21 | [C/T] | ATTCTGTTTGATGCATCTTTCTGCGTGGTGGAGGTC |
| 23880_2 | 3 | [G/C] | CCGGAGAGGTGGGCACCCCTGTGCTAGCTTAACTTG |
| 23881_19 | 20 | [G/A] | AGATCGTCCCTTGCAGCTGGATGCAGAGTGCACACT |
| 23882_5 | 6 | [T/C] | ATATCTTTGATTGCATCCGCTTGCTCACGATAATGA |
| 23884_2 | 3 | [T/A] | ATAAGAAACTGAGCACCAAGCTGCTCGCTTTTAGAA |
| 23892_11 | 12 | [A/G] | AGACGGTTGTTAGCATCCTGCTGCATGCGTCTCACC |
| 23896_19 | 20 | [A/G] | GATTCAGAGTGGGCAGCACGGTGCACATGGTAATGG |
| 23897_19 | 20 | [G/A] | CGCCAGCCAAAAGCAACACGGTGCACTAACAGAGCT |
| 2390_29 | 30 | [G/A] | GGACTACCTGTGGCACCACCATGCTGCCTGAGAATC |
| 23903_29 | 30 | [C/A] | ACATATCACGGTGCATCTGCATGCTTTAGCTTTCTC |
| 23904_16 | 17 | [A/C] | CCTCTGTGTCAAGCAGAACCATGCCAATTAGGCAAT |
| 23907_27 | 28 | [C/T] | GCACATTCAGCTGCACAGTAATGCACTCGCACCCCC |
| 23908_33 | 34 | [C/A] | TAAGATCCAAGAGCAGGGATGTGCGAGTGCTTTCAC |
| 23909_28 | 29 | [G/T] | ATGTGGCTCTGAGCACAACCTTGCCATCGTAGGTGA |
| 23911_2 | 3 | [C/G] | CGGCGGGTCGGGGCAGAATCGTGCGCCCGCCGCTGC |
| 23915_8 | 9 | [A/G] | GCGTGGCCATGAGCACGATGATGCAGACCTCCAGCA |
| 23916_32 | 33 | [G/A] | CAGCGTCCACTGGCACAGGGGTGCTGGTTCGGGGGA |
| 23923_3 | 4 | [T/C] | CCGTTTCCACCAGCACGAGAATGCCGAGGAGGAGCT |
| 23926_7 | 8 | [A/C] | ATGACAGAGAGTGCATGCTAGTGCTAGCTCTGATTT |
| 23934_6 | 7 | [G/A] | TTTGTTGAGTGTGCATTAGTCTGCAAGTGCTGTACT |
| 23942_3 | 4 | [T/C] | CCCTAGGCTTCTGCAGGTCTTTGCTTGGCCTCTGAA |
| 23951_17 | 18 | [G/T] | CTGAATATTCATGCACAGTCCTGCCTCATGAGCTCT |
| 23956_20 | 21 | [T/A] | TGTGCCCGCTGTGCAGCAAATTGCTGACAATTGTGC |
| 23960_33 | 34 | [G/A] | GTAAATGTATTGGCACTCTGCTGCAGCATTAACGCG |
| 23965_27 | 28 | [T/C] | TGCATGAAGCCAGCAAGTTATTGCACTTACACAGCC |
| 23968_17 | 18 | [A/C] | CAGCCGTATTCTGCAGCAGCTTGCAGCTTGGCAATC |
| 2397_5 | 6 | [T/C] | ACAGCTTTCTCTGCAGCTGTGTGCGGTCACTCGGGT |
| 23971_10 | 11 | [C/G] | ACAAGACTGACAGCAAATGCTTGCCTGTGTGAGTGT |
| 23976_1 | 2 | [C/T] | TCCATTCGTCCGGCACCGTTGTGCCCCGACAGCCAC |
| 23978_33 | 34 | [A/G] | CCTAAACACGCTGCAAACCAATGCCAGTGCCCTAGA |
| 23979_32 | 33 | [T/A] | CTTTCCTCCCTCGCAGCTGGATGCACATCTGTTACA |
| 23981_16 | 17 | [G/A] | TCAGCCTTGTGAGCAGACAGTTGCAGGGTCAGGTTT |
| 23997_33 | 34 | [T/G] | ACTTCTCACCCGGCAATCTTGTGCAGCTGACTGTAG |
| 23999_18 | 19 | [T/C] | ATTGACTTCACAGCATTTCTTTGCATGGGTCTCAGA |
| 24006_30 | 31 | [C/G] | CCTGGTACACTGGCATTCACCTGCTCCCACCGTCCA |
| 24007_26 | 27 | [A/T] | AAACTAGTTTTGGCAAGCAGATGCCATGCTGCACGA |
| 24009_27 | 28 | [G/A] | TCCATCCAACTCGCAAACGGCTGCCTGGTGCCTCCC |
| 2401_9 | 10 | [C/T] | AGCTTCAAACGGGCAGGGCGCTGCTCAGCGTGGAAC |
| 24016_27 | 28 | [C/A] | GCTCCACAACCTGCACACCACTGCAATCCACTACTG |
| 2402_27 | 28 | [C/T] | CCTGCTACTGGTGCATGCCAGTGCTTTTCTACCCAC |
| 24023_25 | 26 | [G/A] | CACATGCTTGTGGCAACATTGTGCCGTAAACATCAG |
| 24027_18 | 19 | [T/G] | TCTGACGCCACTGCATCGTGCTGCAGCTTCCAGGAA |
| 24030_8 | 9 | [C/T] | CAACTCTCCACTGCACATTGCTGCCATATGGTAGTG |
| 24033_26 | 27 | [G/A] | ACACTCAGCATCGCATATACTTGCACGAGCGGTCGA |
| 24035_15 | 16 | [C/T] | TCTTCCGATAATGCACCCTAATGCTGTCTCTTCCCC |
| 24043_8 | 9 | [G/A] | CAAGGCTGGGAGGCATTTCTGTGCCCGGTTTTGTAG |
| 24046_19 | 20 | [G/A] | TCGAGTCATTAGGCACAGGGTTGCTTGATACAAAGT |
| 24049_33 | 34 | [C/T] | CCAAAATATGGTGCAAGCCTTTGCGTCTGCATTCCG |
| 24054_16 | 17 | [G/A] | TCATCCTGGAGTGCAGGAGCGTGCGGATCGGAACGC |
| 24056_20 | 21 | [G/A] | AATCAGGTTGCAGCAGCCCAGTGCAAACATACAGCA |
| 24060_30 | 31 | [G/A] | TAATCTCGCACTGCAGCTGTCTGCTCCCCCGGCTAT |
| 24065_20 | 21 | [C/T] | TTAACACCACCCGCACTAACCTGCAAAACAGCTCTG |
| 24066_17 | 18 | [G/C] | TTTTAGCCCCGTGCAACGAGATGCTGTAAAGTAATA |
| 24067_9 | 10 | [G/A] | GGGACCATCGATGCAGTCCCATGCATGAGGTCATAC |
| 24069_7 | 8 | [G/A] | GGATTTGGAAATGCAGTGAAATGCAGTGGGCTTGTC |
| 24074_20 | 21 | [T/C] | AAATGCAGCTAAGCAATGCTTTGCCTGACCCTAATC |
| 24079_31 | 32 | [G/T] | AAACTTACAAGGGCATTTAGGTGCAGCCTTAGGCTT |
| 24085_25 | 26 | [C/T] | GCAGATTTTCCCGCAGATGGCTGCACGAAGTTCATG |
| 24087_30 | 31 | [G/A] | TAGTCATAAGTTGCAGCCCGGTGCTCGCCTGGGACT |
| 2410_9 | 10 | [A/G] | AATGATAATAACGCAAACTGCTGCTTACCTCGGAGC |
| 24103_2 | 3 | [C/A] | ATCATCATTTCTGCACCTTTCTGCATCCAACCTCTA |
| 24107_11 | 12 | [A/G] | AAGCAGCACACAGCATTTACTTGCGCAAGGTCCTGT |
| 24108_3 | 4 | [A/T] | GTCTAGCCTACTGCACCTGCCTGCTAGTCCAAAAAG |
| 24109_27 | 28 | [A/G] | CAAAAAGAAAGAGCATCGAGATGCTAAGGCTGTGCG |
| 24110_28 | 29 | [A/G] | GCAGAAATCAGCGCAAGGACTTGCTTTCATTCCTCC |
| 24112_5 | 6 | [G/T] | GACGTGAAGACGGCACGCTGCTGCTTCCTTCACACG |
| 24114_15 | 16 | [C/T] | GTTAAGTTAAAAGCACTGCGCTGCTGCTCCGCTGCA |
| 24117_34 | 35 | [A/G] | GGAGCAACACTGGCAACGCTTTGCAAAATGCTTCAC |
| 24120_1 | 2 | [C/A] | CCCGAGTTCAGCGCAAACGCTTGCGTCACACCAACG |
| 24122_24 | 25 | [G/A] | CGAGAGCCTCTCGCACAAGCATGCGGAATATAGACA |
| 24127_16 | 17 | [G/T] | AACAGCATGGCTGCATGGGTCTGCAGCACGACTCAC |
| 24134_24 | 25 | [A/T] | TTTGTGGGACAAGCATATGCCTGCAAGACCACAAAT |
| 24151_32 | 33 | [G/A] | TTTTCAACTACCGCACTGCACTGCATGTATTTGCTC |
| 24153_25 | 26 | [C/T] | TTGACCCAAGAAGCAAAGGACTGCACTATATCGCCA |
| 24154_34 | 35 | [T/C] | ACAAGTAACAAAGCAGGGAGTTGCTGCTATTGGGTG |
| 24157_8 | 9 | [A/G] | CCCCGGGTAGAGGCATAGCTATGCCTTAATGCCTCT |
| 24160_24 | 25 | [G/A] | GAAGCCTGAGCTGCATCTTAATGCGTTTGGGTCACA |
| 24162_28 | 29 | [A/T] | CAACCGGCATCGGCACACTCCTGCACACACACACAC |
| 24169_29 | 30 | [C/T] | CAGAAAGGGAGCGCATGGAGCTGCTTCAGCGAGATT |
| 2417_6 | 7 | [G/A] | GGCCACGTTGTAGCAGATCCGTGCTTCCAGTACCAC |
| 24174_8 | 9 | [G/C] | ATTTAACAGAGGGCATGAACCTGCCTCTCATACTCA |
| 2418_6 | 7 | [A/G] | CTTGGCATTGCAGCAGTGATGTGCAATAGTGGAACA |
| 24180_2 | 3 | [T/A] | TTTTTGAAGGCTGCAGGAAGCTGCGGTAATTTCCTC |
| 24181_26 | 27 | [C/T] | TTTGGCCATGGTGCAGATTAGTGCCTCCCATATACA |
| 24190_3 | 4 | [C/T] | GTCCGGTCAGTGGCATCCACATGCATCTGCCACCAT |
| 24196_9 | 10 | [T/G] | TTACAAAACTTTGCAGTGGTCTGCTGTGTGTATGTG |
| 24200_34 | 35 | [T/C] | AAAACCCTAACAGCAGCATCGTGCCCTACTGTTGTT |
| 24202_31 | 32 | [G/T] | ATGTTGTACGAAGCATGGGTATGCCAGCTGTGCCGC |
| 24204_11 | 12 | [G/A] | ACATATGCCGTGGCAGATTGTTGCCATGGAGGCATC |
| 24205_33 | 34 | [T/C] | TACGGTTGTGCAGCAACACAATGCCCAGTCGACTTA |
| 2421_30 | 31 | [T/C] | ATCTTGAAGGAAGCACATGTTTGCCATCACTGACCT |
| 24210_2 | 3 | [T/A] | ACTCTGTGGCGAGCATGTAGTTGCCTAAAGCACCAA |
| 24220_8 | 9 | [A/C] | GAGTGCCTAAGGGCAGACTGTTGCATCATGTGTTTT |
| 24221_1 | 2 | [G/A] | GGAACTCTGAAAGCAGCCCGTTGCTGGGCCGCTTCT |
| 24222_34 | 35 | [G/A] | AGTCAGGACCTGGCAATAGCGTGCGTGTGCGCACGC |
| 24226_24 | 25 | [C/T] | CCAGGATTCTGGGCAGGATTCTGCCATTTGTGGGCC |
| 24228_8 | 9 | [A/G] | ACACGTGTACATGCAAATGTGTGCACATGCCCTCTA |
| 24235_26 | 27 | [G/A] | GGTGTTACCATGGCAACCGCATGCTTGATTGCCTCA |
| 24237_8 | 9 | [G/C] | TACACGTCGACGGCATGCTGTTGCTTAGCAACAGGT |
| 24238_2 | 3 | [G/T] | ACGGCGCGTCAGGCAAACTGCTGCGGCTTTTGTGAA |
| 24239_25 | 26 | [A/T] | TTCTGCTGAATTGCAGACGCTTGCCAACAATTTCCC |
| 24245_18 | 19 | [C/T] | GAGTATTAGCCAGCATTTCACTGCTGCAGACTACAT |
| 24249_20 | 21 | [G/A] | GCACCAGCTGTGGCACCACCGTGCTGCCCGTACTCT |
| 24253_32 | 33 | [A/C] | GGGTCTGTCTGTGCACATGCCTGCTCACAGAAAATG |
| 24255_34 | 35 | [A/C] | CCCTCAGCGTCTGCAGTGATCTGCTATTCTTTAACG |
| 24263_9 | 10 | [T/C] | CAGGGAGCCTGAGCAGAGACGTGCAGGGAGGCCAAT |
| 24268_10 | 11 | [C/T] | GCTCCGGCGGCGGCAAATGGCTGCGTATCTCTCTTC |
| 24273_6 | 7 | [C/A] | CAGGACCGAGATGCAATGTTTTGCTCAGCCAGTGAG |
| 24282_24 | 25 | [C/T] | CGCAAGGAGCGAGCAGGCATCTGCCGAATCTGGAGT |
| 24287_31 | 32 | [A/G] | AGACGATAAGGTGCACCCCTCTGCTGACTCCAAAGC |
| 24294_7 | 8 | [C/T] | ATGTCCACATAGGCAGTCCAGTGCCAGTGTTTAAAC |
| 24295_31 | 32 | [T/C] | GAGCTTTTAAGGGCACTTCACTGCAGTCACCTAAAT |
| 24299_20 | 21 | [A/G] | GATGTAGAGGTGGCACGGTGATGCTGCCGGAAGCTT |
| 2430_10 | 11 | [G/A] | GCCGCTTTTAGAGCATCGGCATGCTTTCATTCAGAG |
| 24302_4 | 5 | [A/G] | TGCTAGCTGGGTGCATCGGTCTGCTGCCTCTGTTAG |
| 24304_24 | 25 | [G/A] | CAGGTTCTTGACGCACGATCGTGCGGATGTGTAGAA |
| 24306_15 | 16 | [A/T] | CATTCACTTCCAGCAACGTACTGCTTTTACAGCTCG |
| 24307_18 | 19 | [G/A] | GAAGGAGAGAGAGCAGCGGTCTGCTCACACTGCTCT |
| 24308_24 | 25 | [C/T] | CTCTGCAAAAAAGCAGGTTTGTGCCGAAGAGGCGAA |
| 24309_25 | 26 | [C/T] | TCGGAGTGGGCAGCAGCCGGATGCTCTACGATATCG |
| 24317_2 | 3 | [G/A] | GTGAGAGTGTGTGCAGGCACATGCACGTGTGCAATT |
| 24326_18 | 19 | [T/C] | TGTTCCTGTGGAGCAGCTTCCTGCCATGAACCAGGT |
| 24333_26 | 27 | [G/A] | TTACTGTGGAAGGCAAACTTCTGCTGGAATCCCTTC |
| 24334_17 | 18 | [G/A] | ATGTTGGCTTGAGCAGAGCACTGCATCCTGTCAGGG |
| 24335_3 | 4 | [G/A] | TTGGACATTCTAGCACGGCCTTGCCACTCGCTTGTT |
| 24338_28 | 29 | [G/A] | AGGATTCTACCAGCACAGAGCTGCGATCGCACTTAT |
| 24343_1 | 2 | [C/T] | TTGACTCGTTATGCACGTCAATGCTCATCTCGTAGA |
| 24348_15 | 16 | [G/C] | ATACCCTGCACAGCAGTAGCATGCTGGAGGTCATTT |
| 24349_34 | 35 | [C/T] | CCCTGACTCCGAGCAGATAGCTGCCAAGTTACTGCG |
| 24351_31 | 32 | [T/A] | TGCCTCCCGGTGGCACAATAGTGCTTTCCTATGGGC |
| 24354_24 | 25 | [G/A] | CGGTCTCATCACGCAACAGCATGCGCCGCTCAATGG |
| 24356_30 | 31 | [T/C] | ACCGGGCACAGGGCACGCGCATGCTGAGCTTCAGCG |
| 24359_27 | 28 | [T/A] | ATAGCCTGTGGGGCACCAGTGTGCCGTTAAGAGGCT |
| 24366_6 | 7 | [G/A] | AAAAAAACTGCCGCATCTCTGTGCCTTCACACTTTA |
| 24368_33 | 34 | [G/A] | CTAAATTAGCGGGCAAATCTCTGCGCCCATCAGGCG |
| 24369_26 | 27 | [A/G] | TATTATCACCCGGCATCTTCGTGCCCAGAGCAGCAA |
| 24370_28 | 29 | [G/A] | GTTCCCTGTGCTGCAGTTGCCTGCACATGGGTGTTG |
| 24375_18 | 19 | [T/C] | TGTGTTGACGGTGCAGATTAGTGCATCTGGGTTAAT |
| 24378_5 | 6 | [C/T] | CCACTCCAATCAGCAAACTCATGCCCCCCATGGCCT |
| 24380_29 | 30 | [G/A] | ACATCTGTAATGGCACCGATCTGCTCCATGGTGGCG |
| 24396_27 | 28 | [T/C] | GGTTTAACAGTGGCAGATCGGTGCTGCTGTGGTTCG |
| 24399_28 | 29 | [C/T] | TGTTAACATCTGGCAGTGTTATGCTGTGCGGCAATT |
| 24403_19 | 20 | [G/A] | GACCTGAGAGACGCAACGCGCTGCGGGTTTTGCGCT |
| 24408_2 | 3 | [C/A] | ATCATGGTGGTGGCAGTGTTATGCTTTGTGTCTGTT |
| 24409_16 | 17 | [C/T] | GTTCCTGCGGCAGCAGCAGAATGCCACCGTCACCGC |
| 24412_9 | 10 | [G/A] | CGCTAATAGAAGGCAGGCCTGTGCAACGGCGTGGGA |
| 24417_7 | 8 | [C/T] | CTGTGATCAACAGCACGTTCGTGCACAATAAGACGG |
| 24423_26 | 27 | [T/C] | CAGCTTGGATTTGCACTACGCTGCCATGTCGTCAGT |
| 24424_34 | 35 | [G/A] | CTTGTTGTTCTTGCAACCTTTTGCATGAGAGCCTGA |
| 24427_19 | 20 | [C/T] | TGAGTTTGTACAGCAGGTTCGTGCATTTCCAGAGAG |
| 24430_28 | 29 | [C/G] | AATGGGACCACGGCAACACGGTGCCGAACGTGGCCG |
| 24441_16 | 17 | [G/T] | CCACATCATCAGGCAGGAGCTTGCTCAGTTCTCTCA |
| 24442_5 | 6 | [T/G] | AAAGGTGTGGTTGCATAAGTGTGCACAACCCGAAGC |
| 24447_15 | 16 | [G/A] | ACAACCAAAAAGGCAGTCTCATGCCAGGTCACGATG |
| 24450_1 | 2 | [G/A] | TGAGGTGCCCTTGCATGCACTTGCGCAGAGTGATAC |
| 24455_3 | 4 | [G/A] | CAAGAGACCACAGCAGCATTCTGCAACCCAAAAGCA |
| 24470_24 | 25 | [T/G] | ATTGGTGATGATGCACAGCTGTGCTTGTGCCCCCTT |
| 24472_31 | 32 | [T/A] | GCAAGGGCCAGTGCATGAGCCTGCCTCTGCCTCAGA |
| 24481_10 | 11 | [G/A] | TGGTCACAAGGAGCAAACTGTTGCTCTCTGCCTTCA |
| 24483_24 | 25 | [A/G] | TGGCACGTCTGAGCAGCTGCATGCAAATCACCTACA |
| 24485_34 | 35 | [C/T] | CTGCGTGATCCTGCAGCAATCTGCTAGAGCATCACT |
| 24487_2 | 3 | [G/A] | ATGGCAGAACAAGCAGATGGCTGCTGGCCTGCTCTG |
| 24488_15 | 16 | [C/A] | CTGTGTTAACCAGCACACAGCTGCAGTTGTGTGTTG |
| 24490_20 | 21 | [G/A] | AAGCTTTGTTGCGCATCAGCGTGCTTTTTAGCTTTT |
| 24493_10 | 11 | [G/T] | AAGTGCCCTTGGGCAAGGAGTTGCTCCCTGGGTGCT |
| 24496_1 | 2 | [A/G] | GAGCTTGAACCTGCAATCTCTTGCATCAGAGTCAAT |
| 24498_25 | 26 | [C/A] | AAGTTAAATCTTGCAGCCAAGTGCCCAATGCTTTAT |
| 2450_17 | 18 | [G/T] | GTGTGCGCGCGTGCAGTGCAATGCAGTCGAAGGTGC |
| 24500_32 | 33 | [C/T] | GCCTTGTGTCTGGCATCTTTGTGCCATTCTGGCACC |
| 24501_6 | 7 | [A/G] | GACCGAAGCTAAGCAGCTAATTGCCTTAACATCATT |
| 24507_3 | 4 | [G/A] | AAGGAGCTCAACGCACCATCGTGCCCTTAATGGTTC |
| 24508_32 | 33 | [C/T] | TATGGCTTGTTAGCACAGCTCTGCTGCTTGATCTCA |
| 24510_26 | 27 | [C/T] | TTTGATAGGACAGCAGGCTGCTGCGCCGGTGTCGGG |
| 24513_33 | 34 | [C/T] | ACTGCAGAAGCTGCACTCACCTGCTCTATCTATCTA |
| 24514_4 | 5 | [C/T] | CTCGCGCGTCCGGCAGCGCTTTGCCCTTGGGACAGT |
| 24519_11 | 12 | [G/A] | TGTGGCAGTCAGGCAGAACATTGCATTACTGTTGTT |
| 24520_31 | 32 | [C/A] | ACGAGAGACTCGGCATTGAGCTGCTCTACAGCGAAC |
| 24521_26 | 27 | [T/A] | CACTTAAGAGTGGCAGGCTTCTGCTGTGGGTGTGCA |
| 24525_8 | 9 | [T/G] | GTGAAACTTTAAGCACTGACCTGCTGTGTGTTTTAA |
| 24528_15 | 16 | [C/T] | GTGTATATAAATGCACGGTAATGCGGTGTGACTGAC |
| 24533_6 | 7 | [C/T] | GCTTATCACACAGCACTGAGCTGCTGCACATAATCA |
| 24547_24 | 25 | [C/A] | GTACACTCAGTGGCACGTTTCTGCCTGAGTGTTTGG |
| 2455_26 | 27 | [T/A] | AGCTATGGAAATGCATTCTGATGCACTCGCAGAGAC |
| 24551_20 | 21 | [C/A] | CAGCTGGATGGGGCAAGATTCTGCCCCTATCTATCT |
| 24553_34 | 35 | [A/G] | GTGATCCTACTAGCAAGAGAGTGCAAATACCCACAA |
| 24555_25 | 26 | [G/A] | CCAGCAGCAGCAGCATCATCATGCCGGCTCCTTTAC |
| 24560_25 | 26 | [G/A] | GTCCAGCCCAGTGCACGAAGATGCTGATTGACCCCG |
| 24562_15 | 16 | [C/T] | CAGTCTGAGTCTGCATGTGTGTGCGCGTGTGTACGC |
| 24567_16 | 17 | [T/G] | TCACAGCTGTGTGCATGGGTTTGCAGTCACACATCG |
| 24572_33 | 34 | [G/T] | GTATAGTTACACGCAATAGGGTGCACGTTGAGAGCT |
| 24575_19 | 20 | [A/G] | TTCACCATGCCAGCAGATCAATGCCTGGCTGAGCCC |
| 24578_17 | 18 | [G/A] | TAATGATGATGAGCATGGAGTTGCGCAGGCTTGTCC |
| 24579_11 | 12 | [C/T] | GGCCCATCATACGCATTCCTATGCAGCGCTGCAGAT |
| 24580_29 | 30 | [G/A] | CCAGCGACTCCAGCAGCCCCCTGCGTAACGGCGTGG |
| 24581_20 | 21 | [G/A] | TGATTTTACTCTGCAGCTGCGTGCGTACTCAGGAGT |
| 24582_32 | 33 | [C/A] | CTTCAAGATGCAGCATAAGAATGCAGCAAAGCCGAG |
| 24587_11 | 12 | [T/C] | AAATGCCCTTTTGCAAGTCTTTGCATGAGATGGCTC |
| 24593_24 | 25 | [A/G] | TCTCCCGTATTTGCAGACGTTTGCAATTGCGCTTTG |
| 24598_19 | 20 | [C/T] | GAGCCTACAGGTGCATGGCCGTGCTGGAACAGGTTT |
| 24603_28 | 29 | [C/T] | ATTGAAAAACATGCACAGTTCTGCTCGGCTGTAGCT |
| 24604_32 | 33 | [C/T] | CACAGGTAATATGCAGTGGGGTGCCATGCCATCCAT |
| 24605_26 | 27 | [C/T] | GCCTGAGCAAGAGCATCCTCGTGCTTCAAACACACA |
| 24606_6 | 7 | [C/T] | TTGGGTCTAAATGCAAACACCTGCGTTTATTCTGCT |
| 24608_18 | 19 | [A/G] | AACTCAGGTTTAGCAGACAGCTGCTAAAAGGGTCAA |
| 24610_32 | 33 | [T/C] | TGTGTACTAAAAGCAGTCCTGTGCCAGACACATGGG |
| 24611_9 | 10 | [C/T] | TTCTCCGCTCCAGCAGAGTGTTGCTATCTGGAAATC |
| 24612_25 | 26 | [A/G] | TAGTTACTGGTAGCACTCCAGTGCTAGGTTGTGTTT |
| 24614_5 | 6 | [G/A] | AGACTGTCAGTGGCAGTGCCATGCAGACCATGCTTA |
| 24617_28 | 29 | [C/T] | GATAAGGGACGAGCAGATTTTTGCGTAACCTTTTTT |
| 24619_34 | 35 | [A/G] | ACACCATGATGAGCAGGAAGGTGCAGGCGAATACAG |
| 24625_4 | 5 | [G/A] | GTGTGAGTTATAGCAGGCTAATGCAGCTCACATTTA |
| 24628_31 | 32 | [G/A] | GGGGACTAGTGCGCAGCTGCCTGCCTGTCTGGAAGT |
| 24629_3 | 4 | [T/C] | AACTTGTTGCTTGCATGACAATGCCCCTGTGTACAC |
| 24630_30 | 31 | [G/A] | GAATAGCAAAACGCAAGGCTTTGCCATCTTGAAGGC |
| 24633_34 | 35 | [C/T] | GGCACTAATGAGGCAGTTGCGTGCGGCAAGGTCTCT |
| 24636_11 | 12 | [A/G] | AAAAACAGCACAGCATCTTAATGCCTGGTTTGTTCC |
| 24638_8 | 9 | [T/C] | GATAAATCCTGGGCAAGCTCCTGCAGGTAAGACTGA |
| 24641_17 | 18 | [G/A] | GTGTCACTCTCCGCACCGGCCTGCTGCTGAGATAGA |
| 24643_26 | 27 | [A/G] | CCAGCTCAGCAGGCACATGAGTGCTGAATCTGTAGA |
| 24644_9 | 10 | [G/A] | TGGAAACAGGGGGCAATTAAGTGCCTGTGAAGCTGA |
| 24646_34 | 35 | [G/C] | AACTGCGTGTGTGCATTTGCGTGCGTGTGTGTGTGT |
| 24648_25 | 26 | [C/T] | ACCTGCCGAACGGCATCTTATTGCTCAAACAGCTGT |
| 24649_26 | 27 | [C/T] | GAGTGGTCACTCGCAGTGAGTTGCATCTTAACACAT |
| 2465_19 | 20 | [C/T] | ACACACGACACCGCAAGTCCGTGCAATCCGAATTAG |
| 24654_33 | 34 | [C/T] | GCAGCAGTGTGCGCACAAGCCTGCCATAGCTCTCTT |
| 24656_8 | 9 | [G/A] | CCACGGGCGGAGGCAGAGGGCTGCTGGTGTTGTTGT |
| 24659_28 | 29 | [A/C] | GGATTAAACCAGGCACCCTGATGCTGCAAGTTTTGA |
| 24664_5 | 6 | [A/T] | AATTAAGTGGGAGCAGTGCTGTGCATACCTCCATAG |
| 24668_32 | 33 | [T/A] | TGGGAGGCGTAAGCAAAAATTTGCTCCCAAGATTAC |
| 24670_18 | 19 | [G/A] | AATAAACCGAATGCAGCGGGCTGCTGGAAACAGAAG |
| 24671_27 | 28 | [T/C] | GAGTCACAGTCAGCACAAACGTGCATATGTGCTGTA |
| 24674_11 | 12 | [A/G] | TTGTGTTCAGGAGCATTGTCATGCTTGGGTCTTGTC |
| 24676_8 | 9 | [C/T] | TGCATGTTCGTTGCATGTGGGTGCCAAAACTTTCAC |
| 24677_31 | 32 | [C/G] | CCTGAGGAGGGCGCAGTGCTTTGCAGGTTCTCAAAC |
| 24678_1 | 2 | [A/T] | TAGTCCCACTGAGCAGCCTTGTGCTGTCACCTGGGA |
| 24679_17 | 18 | [A/G] | ACCAGAGCAGCGGCAGAAACGTGCGTGGAGAACACA |
| 24688_4 | 5 | [G/A] | GAATGGAAAAGTGCAGATTCCTGCTGAGAGCCAACA |
| 24690_28 | 29 | [C/T] | GTGTTTCTGTCGGCACGTTTCTGCTCACCGGATCGT |
| 24693_26 | 27 | [A/T] | CAAATACACTATGCACTGAGGTGCCAAGGTGCCACT |
| 24697_15 | 16 | [G/A] | AAGCGCAGTTTTGCAGCCGAGTGCCGAGTCTGCAGC |
| 24703_32 | 33 | [A/G] | GCCAGCAATGTGGCACATTGATGCACTGGGACAAAA |
| 24708_24 | 25 | [G/A] | TAATGCGCTCTGGCACATGTGTGCGTTTTCTTGCAA |
| 24710_9 | 10 | [T/C] | AACAACCAGTTAGCATTGTTGTGCTAGCAGCTAACA |
| 24713_28 | 29 | [A/G] | AGCATGGTGGTGGCAGCATCATGCCGTGAGCACGGA |
| 24724_9 | 10 | [C/T] | CCCCGTGATCTGGCAGCGTGCTGCTGTTGTAAACGA |
| 24725_28 | 29 | [G/A] | TGTGTGACGAGGGCATCACTCTGCAATAGACTGGCA |
| 24730_7 | 8 | [A/G] | GCGAGTCAAGTGGCATATGTTTGCCTTGCAGATCAA |
| 24733_24 | 25 | [G/A] | CACTGCCAGGAAGCACTGCACTGCGATGGTCTGAGA |
| 24736_31 | 32 | [G/A] | TAAAGTGTGTGTGCATGTAAGTGCGTGTGCGGGTGT |
| 24741_7 | 8 | [C/G] | AGCTGGCCGTCTGCAGCGCCATGCCAGAGCAGATCG |
| 24744_4 | 5 | [T/G] | GAGATTTTCTGTGCAGGCGAATGCGATTGGTAGATC |
| 24745_20 | 21 | [G/T] | GAGGCTGAGGCAGCAGCCCCGTGCCACCATTCACCA |
| 24746_34 | 35 | [G/T] | ACAATAACTGTGGCATCATTGTGCGATCCATAGTGA |
| 24755_30 | 31 | [C/T] | AAACCAGGAACTGCACACTTCTGCGCTGCTCACACA |
| 2476_8 | 9 | [T/C] | CAAAGACCTCCAGCATTGCTCTGCTGTTCTATCTTC |
| 24762_26 | 27 | [C/T] | ATATCCTTTAGCGCATTCATTTGCACCGTGCCTGAC |
| 24763_27 | 28 | [G/A] | GACAGGAAGTCAGCACGCAATTGCCATGAGGCATTA |
| 2477_2 | 3 | [T/G] | GATGCAGCAGGAGCAGCAATCTGCAGAGATACTGTT |
| 24770_16 | 17 | [C/T] | TTTTGTGGGATGGCATCGGTTTGCTTTTGCAGTCCG |
| 24771_2 | 3 | [C/G] | CTCTACCACATAGCAGCAGACTGCAAATGGAACAAA |
| 24775_3 | 4 | [G/A] | AAAGAACACCAGGCAGAGAGGTGCCCTGTCTGTCCC |
| 24778_30 | 31 | [C/T] | TCAATTACGGTTGCATTAGGTTGCTTAGTCCGGCTC |
| 24785_34 | 35 | [A/G] | CCATTATTTCCAGCACACTTGTGCTTCACGGGGAAA |
| 24787_34 | 35 | [G/A] | TCTGAGAATGATGCAGAGGTTTGCAGATGGCAATGT |
| 24792_9 | 10 | [C/T] | GGCCCGCTTCAAGCATTGACCTGCAGACTAATTGCA |
| 24798_10 | 11 | [C/T] | GAGTAACGCTCAGCATGATGCTGCCACCACCATGCT |
| 2480_18 | 19 | [T/C] | TTAGAAGGTGACGCAGCCTGATGCTAAGATTAGATA |
| 24800_3 | 4 | [T/C] | ATCTGTCTCCCGGCACAGTGCTGCTCTGAGCCCTAG |
| 24807_18 | 19 | [C/T] | ATGCTACATTATGCAGTCCTGTGCTCTGATGCAATA |
| 2481_10 | 11 | [T/A] | TCTGGCAACATAGCATGACACTGCTGCTGATACATT |
| 24810_19 | 20 | [C/T] | GCAGTACAACCTGCAGCCTCCTGCCTCTCCACACCT |
| 24811_16 | 17 | [G/A] | GCACAGAGAGATGCACGTACATGCACACGCATCTTT |
| 24825_3 | 4 | [G/A] | GAAGGTGATATGGCATGGACATGCGTGGCTGCTAAT |
| 24826_20 | 21 | [G/A] | TTTCCCTTGTTAGCATGCGCGTGCTAGCACACCACA |
| 24829_31 | 32 | [C/T] | ACACACACAAACGCACACATTTGCACATCCCCGTAC |
| 2483_32 | 33 | [G/A] | CCTCCGGTGTGTGCATGCACATGCACATTTGTGTGT |
| 24831_31 | 32 | [G/T] | TATCTCAGTTCTGCAGGCACATGCTACAGGTGTTGC |
| 24832_31 | 32 | [T/C] | TGATCCGAAGTGGCACGTGTTTGCGCCGCATTAGCG |
| 24833_20 | 21 | [G/T] | CAGCCTCGGGCAGCACTGTGGTGCCGCAGGTAGTGC |
| 2484_27 | 28 | [G/A] | GTAGCCTGCCAGGCAGGGGAGTGCATGGAACCTTGA |
| 24841_32 | 33 | [G/T] | AATGTGAGACTGGCATGCAGCTGCTCAGCCATGAAC |
| 24853_34 | 35 | [T/C] | GCATGGACCTGTGCAAAAGTGTGCAAAGTAACTTTT |
| 24856_2 | 3 | [G/A] | GAGGCATCTCCTGCAGAATGCTGCTTGCATGCTAAA |
| 24861_7 | 8 | [C/T] | TATATCTCCGCTGCATTATACTGCCCGAGTGCTGAT |
| 24867_5 | 6 | [C/G] | ACTCACAGACACGCAACACAATGCAGACACACAGAA |
| 24868_2 | 3 | [T/C] | TGTCCTGTGTAAGCACTGAGCTGCATGACTGAAATT |
| 24870_26 | 27 | [G/C] | AGAACTTGGACAGCATCTGTTTGCCTGTAAAGACAT |
| 24877_7 | 8 | [G/A] | AAATAGCGCCCTGCATGTTTTTGCATCAGTAAACGA |
| 24880_34 | 35 | [C/A] | GAGCCAGGTCTGGCAAGAGAGTGCCGATCATCTGCA |
| 24885_7 | 8 | [G/C] | GACTTCAGTATAGCAACACAATGCCAGGCTCACAAC |
| 24889_1 | 2 | [A/T] | CAGAAAGCAGAAGCACTCCCGTGCTCTTATTTCCAC |
| 24892_27 | 28 | [A/G] | TTAATCTGTCATGCAGTTCATTGCTACAAGACGCCT |
| 249_20 | 21 | [T/A] | GTTATGTAGTTGGCAACTGATTGCCACAGCTCCAAC |
| 24919_4 | 5 | [C/T] | CCCTCAGGCCAAGCAGGGCTGTGCAGATGTGTACGA |
| 24924_33 | 34 | [G/A] | AACACTGCTCCAGCAGACTCGTGCCTGTTAAAAAAA |
| 24929_6 | 7 | [A/T] | TAACGGAGACATGCAGCTGTCTGCATTCTAAATAAC |
| 24933_25 | 26 | [G/A] | CACCTAGCCGGGGCAAATCAGTGCCGGTCCGTGGCA |
| 24934_18 | 19 | [G/A] | CGTGACACAAATGCACGCGCCTGCTTCTGTTCCACC |
| 24936_19 | 20 | [C/T] | CTGAAAGGGGCAGCAGGCCCGTGCTCCAATGCTGCA |
| 24946_16 | 17 | [G/A] | CAAAGGCACACAGCACGCATTTGCAGATAACGCACA |
| 24948_8 | 9 | [C/T] | TCATTTTCCGTCGCATTCATTTGCATGGCGGAGGGA |
| 24951_5 | 6 | [G/A] | TGCTGGGATGAGGCATCTAAATGCAAAGGCTGGAGG |
| 24954_6 | 7 | [G/C] | GTGTGTGTGCACGCACTGCTTTGCTTTGCATCTCTC |
| 24955_32 | 33 | [C/T] | TTTCTGCCTTGTGCACAACGATGCTGAGTGGACTCA |
| 24957_2 | 3 | [C/T] | TTCCCCTATGATGCACCGAACTGCCAGCCAGGCGAT |
| 24964_31 | 32 | [C/T] | TCACCGAACTGTGCAGAACTGTGCAATTGTTCCCCG |
| 24965_10 | 11 | [C/G] | CCAGGCTGTCCGGCAGAACGCTGCAGGAGGGATCGT |
| 24967_2 | 3 | [A/G] | GAAAGACGCAGTGCATAGAGCTGCCTGGTTTGTACG |
| 24972_19 | 20 | [G/T] | GCTCTGTGACTCGCAGAGTGTTGCCAATGGATCTGC |
| 24973_6 | 7 | [A/G] | CATTGTACGCAGGCAGACTCGTGCCCTCATAAGGAC |
| 24974_20 | 21 | [G/C] | CAGACGATCTGTGCACTGCTGTGCTGTCGGCCCATC |
| 24977_25 | 26 | [G/A] | AAAGCACATAGGGCATGGTGCTGCCGTTAGCACACC |
| 24979_8 | 9 | [G/A] | CAGCGACTGAACGCAATTCCTTGCCAGTGACACACA |
| 24981_2 | 3 | [T/C] | TGCGCAATCTCAGCACCCAGCTGCAAATAGTGTCCA |
| 2499_5 | 6 | [A/G] | GGGTGACATTCTGCAAACTCCTGCAGGGTCTCCAGT |
| 24991_19 | 20 | [A/G] | CAGCATAAGTTTGCATGGCATTGCACTTCTGCCTCA |
| 24998_28 | 29 | [G/A] | GAAACTTGGCTGGCATGTGTGTGCCTCCGCCATTAG |
| 25004_1 | 2 | [C/T] | TCTGCTGAATCAGCAGTCCTGTGCAGCAGTACATGA |
| 25011_27 | 28 | [C/T] | TTTAGGCCAGGGGCAGACAAATGCAAACGGGACCTT |
| 25015_25 | 26 | [C/T] | TGGACGAGGCCAGCAAGAAGATGCACCGTCAAATCA |
| 25018_32 | 33 | [A/T] | TAACTTGGTCTGGCACATTGCTGCCCTTTTACACTG |
| 25028_27 | 28 | [T/C] | AATGGGTCAATGGCAGGCACGTGCTGTTGTGGTCGA |
| 25029_19 | 20 | [G/A] | AAGTAAGTATGCGCATTCCGGTGCAGCCCATGGGAA |
| 2503_11 | 12 | [G/C] | TGATCTTGGCTCGCATTTCTCTGCACTGCATTTCTT |
| 25034_28 | 29 | [C/T] | GATGTGAGACTTGCATGCAGCTGCTTGTCCATGAGA |
| 25037_3 | 4 | [C/A] | GACCGGTCCAGGGCAGCTCTATGCCCTTTCCACACG |
| 2504_15 | 16 | [T/C] | CTCTAAAGGTGAGCATTTTTGTGCCCAACAGACTTA |
| 25043_27 | 28 | [T/C] | CTTTGCACATCTGCATGACGTTGCTCCTAATGCTTC |
| 25046_26 | 27 | [T/C] | AAGGGTGATGGAGCAAACACATGCTTTTTTCAAGAC |
| 25049_19 | 20 | [C/T] | TCTCCAAATTACGCAGCTTCCTGCTGGGTGAGTAAC |
| 25060_3 | 4 | [C/T] | TACCGTGTGTGTGCATGCATATGCGTGTGTGTGTGT |
| 25061_10 | 11 | [G/A] | ACCAGAGCAAGAGCAAAAATGTGCATGTGTACGAGC |
| 25074_10 | 11 | [G/A] | CCGAAGGTACGGGCAGAGCTGTGCTTCAAACACTTT |
| 25075_30 | 31 | [G/A] | CTTTGACCTGTTGCAGGTGTCTGCGTAGGAGGTCAT |
| 25078_34 | 35 | [C/A] | GAATGGGTAACAGCACACTTTTGCTGCAAGGATTCA |
| 25079_16 | 17 | [G/T] | GACACTCATGTCGCATGAGATTGCCTGGCTCACATT |
| 25080_8 | 9 | [A/C] | GCACAACGACATGCAATTATCTGCCAGTAGTGGTAA |
| 25081_33 | 34 | [A/G] | TGAGGGTCTGGAGCAGATCAGTGCTTTCCTGTCATC |
| 25084_5 | 6 | [A/T] | GCCAAAGATGGGGCAACAACCTGCCAAAAGTCTGAA |
| 25088_34 | 35 | [G/A] | ACGTGTAATGACGCAAAGCTCTGCTCAAGTTGCCGA |
| 2509_30 | 31 | [T/C] | ACCTTAGTAAGGGCACGAAGCTGCCCCCTATATTCT |
| 25090_17 | 18 | [C/T] | TGCTTAGGAAGGGCAAGCAAATGCCCTGATCTTCCA |
| 25093_29 | 30 | [C/T] | ACATACCTGCAGGCAGCCTCCTGCACTCTCTTATTG |
| 25094_29 | 30 | [C/T] | GTTAGATGAGCAGCACTCTCATGCTCTCCCCTCTCT |
| 25098_27 | 28 | [T/C] | CCAGTTGTAACTGCACCCTTGTGCATCTCACTGCCC |
| 25102_6 | 7 | [G/A] | TTAAGCGGCGGCGCAGATTTATGCTCTTCTTCCTCG |
| 25106_19 | 20 | [C/T] | CTGCGGGCAGGGGCACATTCATGCATCAAAGTGACC |
| 25115_29 | 30 | [G/A] | CCCTGTATAATGGCAGATGCGTGCTTGTGGCCCAAA |
| 25119_1 | 2 | [C/T] | CTTTCCTGTCTGGCACCTCTGTGCTGGAATGAACTT |
| 25123_6 | 7 | [G/A] | TAGAGCGAACGTGCACTGTGGTGCGTTCACTTGCTG |
| 25125_17 | 18 | [A/C] | AGAAAAGGGGCTGCAAACTTTTGCACACTGCACGTT |
| 2513_15 | 16 | [G/A] | GAAGCCTGTGTTGCAGAAATATGCAGATAAGCTGAT |
| 25132_2 | 3 | [G/A] | AGGGTTGTAGTGGCAACTTGTTGCCAGTGGTTGGAT |
| 25133_8 | 9 | [C/T] | ACACTGTCCAGAGCATCACACTGCCTATGTTGGCTT |
| 25135_10 | 11 | [G/A] | ACTCGGAGCTGAGCACAGAGCTGCAGCAGCTGCGCA |
| 25137_26 | 27 | [G/A] | GGCTTCTCCTGTGCACAAAGGTGCTCGAGGTTTGGT |
| 25140_25 | 26 | [C/T] | TGTGTCTCACGAGCATCTGCATGCACACACACACAC |
| 25151_34 | 35 | [G/A] | AGGCTACCTGTGGCACCACCATGCCGCCCTAATGGA |
| 25157_8 | 9 | [T/C] | TTGAGCAATGATGCATCTGATTGCGTTAATGCTAAC |
| 25162_10 | 11 | [C/T] | GGTTTGAGTGCCGCATATTTATGCTGGACAGAACCA |
| 25163_2 | 3 | [G/A] | ACGTGAAAATCAGCATAACTGTGCAGAGGTGTGAGA |
| 25175_10 | 11 | [G/A] | CGTGGGGTACATGCAAAGCCCTGCAGTGAACAACTA |
| 25181_31 | 32 | [G/A] | GGCACGGACAGTGCAGTTTAGTGCTGCATGGGTACA |
| 25187_2 | 3 | [G/A] | GCGTTTGTGCGAGCAGACTGTTGCTTAAAGCATGTT |
| 25191_7 | 8 | [G/A] | GATGGATGAAGCGCAGCTACCTGCTGTGCGCACGGT |
| 2520_32 | 33 | [G/C] | TAAACTTAATGGGCAGTTTAATGCTGTGATGGGCTC |
| 25206_15 | 16 | [G/A] | TGTCAGTCCACTGCAGTTCCATGCCATGTACACACA |
| 25207_19 | 20 | [T/G] | AACAATGCAGCTGCAGACTTCTGCTCCAGGGGATTT |
| 2521_29 | 30 | [C/T] | CATTGTCAAAAGGCATAAAGCTGCTTCTCCCTCGGC |
| 25213_9 | 10 | [C/T] | CCTGCACCGCGTGCAGGGCAATGCTGTCCTGCCAAC |
| 25214_26 | 27 | [A/G] | CACCGCACAGCCGCAGATCGGTGCAGAACGAAAATA |
| 25221_9 | 10 | [T/G] | AAGTGTTATTCTGCACCACCATGCTTCACTGTGGGT |
| 25227_26 | 27 | [A/C] | ACAGGTGGAGCAGCAAGCAGGTGCAGAAGAATTACA |
| 25228_31 | 32 | [C/T] | ACCAGATGCGAAGCAAAACGATGCTAAATCTCCCAG |
| 25232_11 | 12 | [G/A] | AGAGTCCACCCGGCATTTTCGTGCACAAGGAAGGAA |
| 25233_19 | 20 | [T/C] | GCTCACACACCTGCACCTGTATGCAGCCATGACGTA |
| 25236_7 | 8 | [A/C] | GCATCTTAAAAAGCAGCTGCCTGCCAGTCAACTCTT |
| 25237_17 | 18 | [A/G] | CAGGAAGTAAAAGCAGAGAAGTGCTGACGGAAATCT |
| 25238_16 | 17 | [A/G] | CACAAGGACACTGCAAGAACGTGCAACATTCCACAC |
| 25245_15 | 16 | [C/T] | CCAAAAGTCTCTGCACGGCCCTGCTTAAAACTATAA |
| 25250_5 | 6 | [G/A] | GCCTCGGTCATGGCAGGCTGGTGCTAAGTAATGTCT |
| 25254_16 | 17 | [G/A] | GGATGTTTGCACGCAGGAAGTTGCCCTCATACTGGG |
| 25260_24 | 25 | [G/T] | CAAAAGAATTTCGCAGGGGGGTGCGGAAGCTCAGGG |
| 25262_4 | 5 | [G/T] | TCGTGTTGGGTGGCACGAGTCTGCTTTGGGCCCCGT |
| 25263_4 | 5 | [C/T] | CTATCAGGTCACGCATTGACATGCAAAGAAACTCAC |
| 25273_17 | 18 | [G/A] | CAGTGCTACCTGGCACCGGCATGCCATCCTGGCCAG |
| 25280_17 | 18 | [T/C] | AGGCAGGTTCCTGCATTTCGTTGCTTGTTAATGGCA |
| 25281_32 | 33 | [G/A] | TATCATCGTCCAGCATTCCACTGCCTCCCTCTGAGT |
| 25282_7 | 8 | [G/C] | AGCTCCAGTGTGGCATAACAGTGCTGTCAGAGAGGG |
| 25283_28 | 29 | [A/T] | CTCTCTCTCTGTGCATGCCTGTGCCTTTTATCGGAT |
| 25284_17 | 18 | [A/T] | TGCGGCAGCGACGCAGGATTCTGCAAGGGCGGCAAA |
| 25285_7 | 8 | [G/A] | CCAAAGCATTCCGCAGTGACGTGCCATGCTTTGGCA |
| 25298_16 | 17 | [T/C] | TTTTAAATGCAGGCAGTACGTTGCTCACTGTAGACC |
| 25303_28 | 29 | [A/G] | CCCATGTGAAGGGCAGGTGTGTGCGTGAAACTCTTG |
| 25306_20 | 21 | [A/T] | TGGAACAGTTTCGCACTACCTTGCTGTCTGACTCCA |
| 25309_34 | 35 | [C/T] | GCCTGTTCTCCAGCAGAACCTTGCACTCCTGCCTTA |
| 25320_16 | 17 | [G/A] | GGAGCATGGTGAGCACGGGGATGCAGCTTTATCGAC |
| 25322_11 | 12 | [A/G] | TCGCTGGACACGGCACGCCGGTGCTGAAGGTCTCGC |
| 25326_11 | 12 | [T/G] | GGAGACTTCAGTGCACATCAGTGCAATCAAGGGGCA |
| 25327_28 | 29 | [A/G] | AAAGAAAAGTCTGCAGGTTTTTGCGAAAAACTGAAA |
| 25329_27 | 28 | [G/T] | CTGGACAGGGTGGCAAACCACTGCAGAGTACCATAC |
| 25330_4 | 5 | [C/T] | TGTCCTGGTTCAGCAGGCCCATGCATGCTGAGCAGC |
| 25333_31 | 32 | [C/A] | TGCGTCACATCGGCATCGAGTTGCTGGCCTGCAGCT |
| 25335_32 | 33 | [C/A] | TGAGTCATACCGGCACTGCTGTGCTGCTGCTGCTGC |
| 25339_32 | 33 | [C/T] | GAGCACAGGTGAGCAGGGTAATGCACCAAGTCCAAA |
| 25340_16 | 17 | [G/A] | GCTCACTCAGGCGCATAAAGCTGCCCTCCTCCAGAC |
| 25341_9 | 10 | [G/A] | GAACGTGGCGAGGCAGTCGGGTGCATGAGTTCAACG |
| 25343_19 | 20 | [G/A] | ACCCAGAGATGGGCACTCAGCTGCCAGGTTATCCCC |
| 2535_8 | 9 | [C/T] | ATGGCTTTCAGAGCATAGTTTTGCACTGATGTCAGC |
| 25364_2 | 3 | [G/A] | AGGAGGAGATCAGCAGGGGTGTGCCTCGGGTCCCCG |
| 25366_28 | 29 | [C/A] | GGGAAGGTTTTGGCATCACTGTGCCTCACTGTAACT |
| 25373_1 | 2 | [T/A] | TTCTGATTCATAGCACCGGGCTGCAAAGGATCAAAG |
| 25383_15 | 16 | [G/A] | CACACGTTTAGTGCAGAACAGTGCTGTGAACTGCAT |
| 25384_10 | 11 | [G/T] | CTGCTGGGATGTGCAGGACAATGCAGCTGTCACTTG |
| 25385_25 | 26 | [C/T] | AGCTACTGCAATGCACAATGATGCACATCGGCAGCT |
| 25388_31 | 32 | [T/C] | CATAACAGATGTGCAGTACGCTGCAGTGAACTGGTA |
| 25391_3 | 4 | [T/A] | ATGTGGCCCAATGCAATGCTCTGCTTCCAGAAGATG |
| 25395_4 | 5 | [T/G] | TCACTATCTGCTGCAGGGGTTTGCAGTCTAAAACAG |
| 25396_31 | 32 | [C/T] | TCTCTCTCCATTGCAGTCCCATGCCTGTCTTCTATA |
| 254_26 | 27 | [G/A] | TGTTGGCTTGAGGCACATTTTTGCTTGGGCGTGAAC |
| 25405_33 | 34 | [T/G] | AATCTGAGCTGTGCATGGAGTTGCATCAAGTCCTTA |
| 25421_6 | 7 | [T/G] | ATTAACGCGTATGCATTACGCTGCTCGATTGAAACG |
| 25422_31 | 32 | [T/A] | GGCTTTGTGGTGGCACAGTGGTGCAGTGGGTTACAC |
| 25429_30 | 31 | [C/T] | GATTGTAACATGGCAAATTGCTGCTGGTCTCGCACA |
| 2543_20 | 21 | [C/G] | ATTTTTGGATCGGCACTCGACTGCTCATGAAATACC |
| 25431_5 | 6 | [G/C] | GGGAGGACCAATGCAGCTGTGTGCAAACATAGTGGC |
| 25434_24 | 25 | [C/A] | ATGTTACGGATGGCACGGCGGTGCCGCAGAATCCAC |
| 2544_8 | 9 | [T/G] | ATTTCCCATATGGCAGAGAGGTGCATGTGCATTTTT |
| 25444_1 | 2 | [A/T] | TAAATGGCCACAGCAAAACAATGCAATAGGTAGCAA |
| 25446_32 | 33 | [G/A] | GGAAAACAATTTGCACAGAGTTGCCAGGTCACGGCT |
| 25447_7 | 8 | [C/T] | GGCGTGTCCTTGGCATTGTTTTGCGCGTTGACGTTG |
| 25453_16 | 17 | [A/C] | GAGGACAGAAAGGCAGACAATTGCTTTGGCTTCTAA |
| 25464_28 | 29 | [A/G] | GAACACAGCTCAGCATCATTCTGCATTCATGCGAGT |
| 25466_31 | 32 | [G/A] | CAGACTGCAAATGCAGATCAATGCGCTTTACGTAAA |
| 25467_33 | 34 | [G/A] | CTAAAGTGGTGGGCAGCCCTGTGCACAGCACCCGGG |
| 25472_31 | 32 | [G/A] | TTACGCTGTCAGGCAGTGCGATGCAGCAGCTGAGCA |
| 25473_9 | 10 | [C/A] | ATGTGTGAGCGTGCAGTTGTCTGCAGTGATCAAGCA |
| 2548_11 | 12 | [G/A] | TGCTGTGGTGGGGCATGCTTATGCTGCACAGATCCA |
| 25480_16 | 17 | [A/T] | GACGCCTCCGACGCAGATGTTTGCGCGGGCGCTAAT |
| 25483_28 | 29 | [G/A] | GGAACACAACCGGCATCTTCATGCGCAAGGCGGGAA |
| 25484_25 | 26 | [C/G] | TTGTCTGTCGGTGCAGGCGTTTGCACAGGACTAACC |
| 25485_2 | 3 | [G/A] | ACGTTGATAGGTGCAAGGCCATGCAAGGCTTTAAAG |
| 25489_1 | 2 | [T/A] | TTATCCCAGCTGGCAATTCAATGCATCCGATAGCAA |
| 25490_10 | 11 | [A/G] | TGTTTTGCAGAGGCAAACAAATGCCTGCATTCAGTC |
| 25492_24 | 25 | [G/A] | GTGTTAAACCGTGCAGCAAAATGCGCCGATTAACAT |
| 25499_31 | 32 | [G/A] | ACGTACACGGCAGCATTCGTCTGCAGACAGCGAGTA |
| 25500_32 | 33 | [A/T] | TGATTTGATGTGGCATTGGGTTGCTGACCTGGAGCT |
| 25501_16 | 17 | [A/T] | GCTGCTGAAAAGGCATAAGGCTGCGTGTGACAGTGA |
| 25504_26 | 27 | [G/C] | GAATGGACCGTGGCAGCATGTTGCAGGCTGCCCTGC |
| 25510_34 | 35 | [A/T] | TTAAGGCACTGTGCATATTAGTGCACGGACTGAAAC |
| 2552_3 | 4 | [G/A] | ACAGCACATGATGCACTGTGATGCATTGGGGTCGCA |
| 25522_3 | 4 | [T/C] | CATTTGTGTTGTGCAGAGCTCTGCCCTCGAAGGCAG |
| 25523_19 | 20 | [C/T] | ATTACACGTGATGCATCCTCGTGCTGAAAATGTGAC |
| 25525_7 | 8 | [G/C] | TGGATCTGTGGAGCAAAACAGTGCCGAAGAGCAGAA |
| 25533_31 | 32 | [A/G] | TGCAAACAAACTGCAAGGGGTTGCTAAACGCGAAGC |
| 25534_24 | 25 | [G/A] | CAAATCACCCAAGCATTAGTCTGCGTGGAGCTGGCA |
| 25535_19 | 20 | [G/A] | CTGAACTGACAGGCACTGTGATGCCAATTCCAGCCA |
| 25536_16 | 17 | [C/A] | AAGCTTCAGAAAGCAGCAGGATGCTGAACAGAAAAG |
| 25537_27 | 28 | [C/T] | TACTGCGCTGGAGCAGTAGAGTGCGGCCGCCACTGC |
| 25538_18 | 19 | [A/G] | TGGTGGAGGTCAGCACTCGCCTGCAGTTGACCTTGA |
| 25539_30 | 31 | [A/T] | TATAAAGTAGCTGCATCGATCTGCATTCACAAGCAT |
| 25544_5 | 6 | [T/C] | TCTTATGCCCATGCAAACAAATGCAGCAGTGAAGTG |
| 25552_20 | 21 | [A/G] | TCAGATTCTTGTGCACTGTGATGCGATCAGATTTAG |
| 25553_1 | 2 | [A/T] | GATCAAAAAAACGCAGGGTCATGCAGGTCTCAAACG |
| 25554_32 | 33 | [T/G] | CCACTGCTCGGGGCAAGTGTGTGCTGTGACTGTATG |
| 25559_1 | 2 | [T/A] | ATGCATATCTCAGCAAGCCTGTGCAAGAGCCGCTTA |
| 25560_9 | 10 | [A/C] | TGTAGTTAAACAGCAGTTAACTGCCTCAGTCAGGTG |
| 25562_6 | 7 | [A/T] | GCTTACAATAAAGCAGCGATTTGCTGGCTTGGACAG |
| 25568_10 | 11 | [G/A] | GGATTCAAACGTGCAATCACTTGCACCACTCAGGAG |
| 2557_34 | 35 | [T/C] | TGAAACAGATGTGCAAACGCTTGCACAGACAGCTCG |
| 25571_2 | 3 | [C/A] | GGCTTTATTGAGGCAGAGCAGTGCTGAGGAGGCTGG |
| 25575_33 | 34 | [G/T] | CATGGGTCAGCAGCAGGACCATGCGTGGGTCACGTG |
| 25576_16 | 17 | [A/G] | AGGTGTCGCATCGCAAAGGGCTGCCGCATGTCATCT |
| 25577_17 | 18 | [A/G] | ATCATTCGCACAGCATCAGGTTGCATGAGTGCTCTG |
| 25580_1 | 2 | [C/G] | ACATGGCATCTTGCATACCTTTGCAGCTGCGTGAAG |
| 25587_20 | 21 | [C/T] | AATGCACCAGCAGCATATTTCTGCCCTTTTATCCTG |
| 25590_25 | 26 | [G/A] | ACTTTGTGCACAGCATTGTCATGCTGAAAGAGGTTC |
| 25593_30 | 31 | [C/T] | GAGGACAGCCCGGCACAAGGATGCTCTTTCCGTCTT |
| 25594_19 | 20 | [T/G] | ATTAGCTGAGCTGCAAGTTTTTGCTGGTGTTTATTA |
| 25606_7 | 8 | [A/G] | GTGCTGTAGACAGCATTCCTTTGCCTGATAGTGTTA |
| 25615_9 | 10 | [T/A] | GCAGAAACGTACGCACTGCAATGCCAGCTCAAATTA |
| 25625_26 | 27 | [C/T] | TTGTAAAGCAATGCATCAAACTGCTTCCGCCGCCCG |
| 25628_3 | 4 | [T/G] | GAGTTATAGCCTGCAGCAACATGCAGAAAGCTTGCT |
| 2563_15 | 16 | [G/A] | GAGAGGCCAAAGGCAGACAAATGCCTGACAACTCCA |
| 25630_30 | 31 | [C/T] | CGAGAACACTAGGCACGTCAATGCCAAGAACGAATG |
| 25634_4 | 5 | [C/A] | CCTGCCGTCTGTGCAGATGAATGCAGCCTGAGCGCT |
| 25638_4 | 5 | [G/A] | GATCGTTAAAGGGCAGGTTTGTGCCTTTTCGCTAAA |
| 2564_34 | 35 | [C/T] | GGCCGTCGATCAGCACGTGCTTGCTGTTAGTCCTCC |
| 25642_27 | 28 | [C/T] | GAGGATGTCGCTGCACAAACCTGCCCTCAGACGCAG |
| 25644_24 | 25 | [G/A] | AATCACCAACCAGCATGTTGTTGCGAGGTGAGAAGA |
| 25650_20 | 21 | [T/G] | ACCAACTGTCAGGCAGGGTGTTGCATGGGTAAGGAT |
| 25653_33 | 34 | [C/A] | CAATACCTGGGTGCAGATGTCTGCTGTGCATCACTA |
| 25673_7 | 8 | [C/T] | CAATTACCGCAGGCATTCGAATGCAGATTTCTGCTC |
| 25677_26 | 27 | [C/T] | CGAGTGGCGAGTGCACGCGTGTGCGTTTGTGTGTGT |
| 2568_28 | 29 | [C/T] | ATACAGACAGCAGCAGCAGTCTGCCTTTCAAAAACA |
| 25685_32 | 33 | [C/T] | ATGTGTCATGTCGCACAGGCTTGCACATGTGGCTTT |
| 25686_16 | 17 | [T/C] | ACTTAATAGCTGGCACTCAAATGCCAAACTCGGCAT |
| 25688_1 | 2 | [G/A] | CGATTCAGAGCTGCAAAAACCTGCTCACGCCCTACG |
| 25693_34 | 35 | [G/A] | ACAGGATGTCCTGCAACTCCATGCCACATGCTCCGT |
| 25697_4 | 5 | [T/C] | ATTTTTGAATGGGCAGATTTGTGCCACTTTAGCCAC |
| 25699_28 | 29 | [A/G] | GGTCCTCTGGCAGCAGGGTGGTGCCACCAGACGGCA |
| 257_32 | 33 | [T/A] | GACCCAGCTTACGCAAACGTCTGCAGCGTGAAAATA |
| 2570_33 | 34 | [T/G] | TACACACAGGGAGCAGTGAATTGCAACACACATTCA |
| 25700_26 | 27 | [C/T] | ATGGATCCACCAGCAAGTACGTGCTGCAGGAGAGAG |
| 25703_2 | 3 | [C/T] | GCCGGTGAGAGTGCAGCATGGTGCCTGTTCTCACAC |
| 25704_34 | 35 | [C/T] | TTGGTGTTCAGGGCAACGCAATGCGCATGTTGGGCA |
| 25709_26 | 27 | [G/A] | TATTTAGTCCCGGCACTGTGATGCTCGCACAAGACT |
| 2571_10 | 11 | [T/A] | ACTGCTGTGATTGCATGTTTTTGCAGCGTGCTGAGT |
| 25714_5 | 6 | [G/A] | ATGCAGAATTTGGCAGGCATGTGCTAACATTTCACC |
| 25716_5 | 6 | [C/A] | TGGGACCAGGTGGCATGTTGGTGCCAAGTGCTAGTA |
| 25717_20 | 21 | [C/A] | AGAGAGCTGAGTGCAGATTTCTGCCACCATGCCCTG |
| 25718_31 | 32 | [C/T] | GGCAAAACGTGTGCAACGTAGTGCCAATGTTCAAAT |
| 25719_3 | 4 | [A/G] | GCGAGTGTGTGCGCACACGCATGCACACGCGTGTCA |
| 25724_30 | 31 | [T/C] | AACAACTTTCACGCACACACATGCCTACGGTCTGTA |
| 25730_20 | 21 | [C/T] | ACCGTAGCCATGGCACTCTTCTGCTGAAACGCCTTA |
| 25732_18 | 19 | [G/C] | GAGTAAATGCTTGCATGCGTCTGCGTGTGTGCGCAT |
| 25733_27 | 28 | [A/G] | GCTGTCAGACTGGCACCCAGCTGCTCTAGAATAACG |
| 25736_30 | 31 | [A/C] | CATAACACTGCAGCAGGTAGCTGCTTCACTACACCT |
| 25737_3 | 4 | [A/G] | CAAATAAAGAATGCAAGCGCTTGCTGCAAACATGCG |
| 25738_2 | 3 | [C/T] | ATCAAGGTCAGAGCAACAATTTGCCCATCTGCCCCT |
| 25740_8 | 9 | [C/T] | TACCACGCCCACGCATTGTCCTGCTGCAAAACATAA |
| 25741_32 | 33 | [G/A] | ATTACCACATGGGCAGGGCAGTGCTTTCAGCCGTAC |
| 25743_28 | 29 | [A/C] | CATGAGCAGATGGCAGTAAACTGCACACAGGGTTTG |
| 25748_15 | 16 | [G/A] | GACACACATCCAGCAGAGCAGTGCTACAGGCCGACG |
| 25757_27 | 28 | [C/G] | ATGTAGCCCAGAGCAGTTGGCTGCTCTCTATTAGTA |
| 25758_27 | 28 | [C/T] | GTGCTGGAGAGTGCACAGGAGTGCTTTCTGGATTTA |
| 2576_26 | 27 | [C/T] | ACTAGTGTTTGGGCATGGAATTGCATCCACCTGTAA |
| 25762_10 | 11 | [C/T] | TTTTGTGCTTCTGCAGGCAAGTGCCTGGGTTAAAGC |
| 25766_16 | 17 | [C/T] | TATCAGGTTTAGGCACCGTCTTGCGGATGCGAATCT |
| 25770_24 | 25 | [C/T] | ACAGAGATTAAGGCAGTACGTTGCCGCTTCCTGTAT |
| 25771_31 | 32 | [T/C] | ACATGCCTAAATGCACTGAGGTGCGGCCATGCAACT |
| 25773_31 | 32 | [C/T] | TTCACACCAGACGCAATGCTCTGCGGGTGGCCGAAG |
| 25775_6 | 7 | [C/T] | GATGCACTAGTGGCACCATTGTGCAAGCTCAGAACA |
| 25777_26 | 27 | [A/T] | TGCCAAATCCAAGCAGCTTGCTGCTGATGTACAATA |
| 25779_33 | 34 | [C/T] | ATTGCCAATACCGCATCTCGTTGCATACACAACCGT |
| 25780_24 | 25 | [G/A] | CAAAAGGTCAGAGCAGATCTGTGCGCTGAACATCTG |
| 25788_11 | 12 | [G/A] | TAAAGTCCGAAGGCAGGTTTATGCTCACAGGAGAAG |
| 25792_29 | 30 | [A/G] | CAATACCGGACAGCAAAGTGGTGCTGCGTATAATTG |
| 25799_15 | 16 | [A/G] | CACAACAAGTGAGCAATGCGATGCTGAGTTTAATTT |
| 25800_8 | 9 | [G/A] | TGGTGGACGGCAGCAGTGTTTTGCTGTAGCTCCAAG |
| 25805_30 | 31 | [T/C] | AAAGCTTTTCATGCAGCTGTTTGCAAATTGTCTTCC |
| 25808_2 | 3 | [G/A] | GAGCGTCAGTCAGCAGAAAAGTGCAGTCTTCTACAG |
| 2581_4 | 5 | [C/T] | CAAGCCAGATAAGCAGCTCCATGCATGTATGTACAT |
| 25816_11 | 12 | [C/T] | CCTTCCATCTCCGCACATTAATGCGATGCCAGTTAC |
| 25819_29 | 30 | [G/C] | GCATGAGGTGGAGCAGAGACATGCGCTAGGACGCAA |
| 25824_18 | 19 | [A/C] | TCATTATACCAGGCAGTAAAGTGCGAGGACCGTAAA |
| 25826_28 | 29 | [G/A] | TCGTCGTCGACCGCAAGATCCTGCGCCTGGTCAAAC |
| 25830_29 | 30 | [A/G] | ACCACGGGAGTTGCAACAGCCTGCAAGACAGGACGG |
| 25831_31 | 32 | [A/T] | GGCCGGGTAACAGCAGAGGGTTGCCAGATTGAGACA |
| 25837_27 | 28 | [G/A] | AGTGTCGATGTAGCAGGACATTGCCGAGGTTAATGG |
| 25847_27 | 28 | [G/A] | ACTTGGCATTAGGCAAGACTCTGCATGGGTGCCCTA |
| 25865_34 | 35 | [G/A] | CCAGTCCCTCCTGCAACATAATGCTGCCACCCCCGT |
| 25876_33 | 34 | [T/A] | GGTGCTACTTTGGCACTCAGATGCACTGAGACATAA |
| 25878_1 | 2 | [C/T] | ACACTCAACACTGCACACAGCTGCTCAAATTGCTTA |
| 25879_28 | 29 | [C/A] | TCTTCTAAAGCAGCATTCTCGTGCCACTCGTTCTGC |
| 25882_25 | 26 | [A/T] | TGTGGTTCTGGGGCAGCCTCGTGCCATCATCCCAGA |
| 25883_33 | 34 | [T/G] | ATTTGACTTTATGCACTGTGCTGCTGCCACATGTTT |
| 2589_1 | 2 | [G/A] | AGAAAAAAAGGGGCAACATGATGCCAGAATCAGTCA |
| 25895_18 | 19 | [C/A] | TGAGGATCGTTAGCAGCCCCATGCTGCATGCCTATA |
| 25896_2 | 3 | [C/T] | TCCGACATTCTCGCATTGTTTTGCAACCAGGTTAGT |
| 25897_34 | 35 | [C/T] | CAGACACGGTCCGCAAGCGCATGCCCATCCATCTCG |
| 25899_2 | 3 | [C/T] | CACTACCCACTTGCAGACATATGCCAACTTGCCAGT |
| 2590_18 | 19 | [A/G] | TTTAGGGCTGTAGCACTGGTCTGCCGTAACATTAAA |
| 25900_3 | 4 | [A/T] | TGTATTCCCTGTGCATGCCAGTGCTTTCCAGGTGTA |
| 25904_27 | 28 | [A/G] | TCTGTCCAGCTCGCATGACTTTGCTATACAATGGAC |
| 25906_19 | 20 | [C/T] | GGGGACACGACTGCAAGCACGTGCAGGTAAACAAAC |
| 25907_4 | 5 | [G/A] | ATTTGGAACGGTGCAACATGTTGCCCTAATGGCTCT |
| 25911_31 | 32 | [C/T] | GACCTCCGTGGTGCATTCTAGTGCCATTGTTCATCA |
| 25919_11 | 12 | [T/C] | CAAAGCCAGCTTGCAATTGTTTGCCAGTCGCGGGTG |
| 25922_2 | 3 | [G/A] | GTGTACAGTCCTGCATGTCTCTGCTAGGATCTAAGA |
| 25926_4 | 5 | [C/T] | GCGACGAGCGCTGCAGGTTTTTGCTGCTCGTACTTC |
| 25927_6 | 7 | [T/C] | AAGACGTTGAGGGCATGGGGGTGCCTGAAATTAAAT |
| 25928_32 | 33 | [A/G] | ACACTACCTGCAGCATCACCGTGCCACCCATGAAAC |
| 25932_25 | 26 | [T/C] | GGCTCCGGGCAGGCACGGGTATGCGTGAGTGCCTAC |
| 25935_31 | 32 | [G/C] | GCTCAACCTCCCGCAGACAGCTGCGCAGATCCCCCA |
| 25946_25 | 26 | [C/T] | TGTGTGGTGGAGGCAGTGAAATGCACGCTCTTCTTT |
| 25953_7 | 8 | [A/G] | AGAAATGAGGCAGCACAGTGGTGCAACAGGGTAGTG |
| 25954_16 | 17 | [A/G] | GTGACACGACTGGCATATCGGTGCTTGCTCATGTAA |
| 25958_32 | 33 | [C/A] | TTTTGTATTTCAGCAGTCATGTGCTCCCGTACCAAA |
| 25960_3 | 4 | [C/T] | TGTCGTATTTCTGCAGTCTTGTGCTCCACTGGGTGT |
| 25962_26 | 27 | [C/A] | AACAACCCCACAGCAAGATGCTGCCTCCAGCATGCT |
| 25964_19 | 20 | [T/C] | TCACTTTCGCTCGCACTTTTTTGCCAACGTCCCGAA |
| 25969_32 | 33 | [G/A] | CACTGCAGGTCAGCAGAAGACTGCAAACACACGACC |
| 25970_32 | 33 | [G/A] | AAGATGTCTTCTGCACCGAGCTGCCACTGCTGGGCC |
| 25978_2 | 3 | [G/C] | AAGATGCTTTGTGCACTTGCCTGCATGATGATCCTC |
| 25979_1 | 2 | [C/G] | ACGATTCAGCCTGCAGCCTTGTGCTAAGCTGTAATT |
| 2598_15 | 16 | [C/T] | CTGGTAAGCAATGCACGGCAATGCACTCACTCAACG |
| 25980_4 | 5 | [C/A] | AAAACAAATGCTGCACCGTGGTGCATTGTGGGATTG |
| 25995_34 | 35 | [A/G] | TTGAGCAAATGAGCAGTGATGTGCGGCGGCGGCGAC |
| 25999_24 | 25 | [A/G] | TTGGCACAATAGGCATAATCATGCAACAAGACTCTG |
| 26_6 | 7 | [T/C] | AAAAAATGCACTGCAGCATGTTGCCACATACTGTGC |
| 26003_6 | 7 | [C/T] | CCTACTCAACATGCATACACATGCACACACCTTCAC |
| 26009_19 | 20 | [C/T] | ACGTACATCCTGGCACAGTCTTGCCTATCTCTCGCA |
| 26014_33 | 34 | [G/A] | TGACAAGAAGCAGCATGCACCTGCACACCCTTGGAG |
| 26015_8 | 9 | [G/A] | CAGTGTGAGTGAGCAGCCCTGTGCATAGTTAGAAAT |
| 26016_10 | 11 | [G/A] | GTGTGGCAGGAAGCAGTGTCCTGCTGAAACAAGTGA |
| 26024_10 | 11 | [T/C] | CAACAGATCATGGCAAAGACCTGCCCAGTGTTCAGC |
| 26028_24 | 25 | [A/G] | GAGGTCGTCTGGGCAATGCAGTGCACACGTGGAACT |
| 26029_6 | 7 | [G/A] | TGTGCAGCTGGCGCAGGCCCATGCTTACCCTGCACC |
| 26030_7 | 8 | [C/T] | ATGATCCCTGTTGCACGGAGCTGCTCCCACAGGAGA |
| 26031_4 | 5 | [T/A] | ACACTACCTGTGGCACCATGCTGCCAAGCATTTTAA |
| 26042_27 | 28 | [C/T] | ATCCCCAGTAATGCAGGAGGTTGCGGACGCTTGTCC |
| 26043_29 | 30 | [C/T] | TATTACCTGTTGGCAAGGAGCTGCGACTTTGTCCCT |
| 26044_17 | 18 | [G/A] | ACTGTAACGGTGGCATGGAGGTGCTAGGGAACGACA |
| 26045_27 | 28 | [C/T] | AAGTGTAAGCCAGCACCTAATTGCACACGAAAATAT |
| 26053_7 | 8 | [G/A] | GAAAATCGATCAGCAGCAGCATGCACAGAGGATCGT |
| 26057_29 | 30 | [G/A] | TGAACGAGTGAAGCAGCTGTTTGCTTTCGGCGTTGT |
| 26058_25 | 26 | [G/A] | CCCTGGAGATCAGCAGAGCGTTGCGGGTTCTGCGCT |
| 26059_30 | 31 | [C/T] | TGTGTGTGTGTTGCATCACAGTGCCGCTTGCTTCTC |
| 26060_24 | 25 | [A/C] | ATAAGCAGCCGAGCACTAGTGTGCACCATCTGGTCT |
| 26063_6 | 7 | [A/G] | CATGTCAGGGCTGCAGGTCAGTGCAGAGCACTCTGC |
| 26064_20 | 21 | [C/G] | TGTGTTGTGCTGGCATGCTGCTGCTGATGGCCATGA |
| 26068_8 | 9 | [G/A] | TGGAGCTCGCCTGCATTGCTGTGCTGCAGCATTTCA |
| 2607_10 | 11 | [A/C] | AAAGTGTCTGATGCACCTGGATGCACAGGTTTCACA |
| 26070_24 | 25 | [G/A] | GATGGAGTACGTGCACATGTGTGCGTGTGGGGTGGG |
| 26074_33 | 34 | [C/T] | GGGAGACAGAGAGCAGTGCATTGCAAAGCCGTTCAA |
| 26085_19 | 20 | [G/A] | ACTTTTATGCAGGCAGAGGGATGCTCAGGTTCTATT |
| 26088_33 | 34 | [G/C] | CACCGGGTCGAGGCAGGGCCATGCCAGGTAATAGGG |
| 2609_6 | 7 | [T/A] | CGATACTGTTTGGCAGTAAAATGCTGCAGATGCATG |
| 26091_17 | 18 | [C/T] | GCTCTGAGGCATGCAGTCGTCTGCCGCATTCAGCCA |
| 26092_4 | 5 | [T/C] | CTTTTCCAACTTGCATGGTCATGCCTGTCTGATTTA |
| 26103_34 | 35 | [C/T] | CCTGTTCCGACAGCAGGTAGCTGCTCATCCCTTTCT |
| 26107_4 | 5 | [C/A] | AGTCCAGGAAGGGCATTCGGATGCTGCGCTCGGCAC |
| 26108_8 | 9 | [G/T] | TTGCAGTGGACCGCACACACATGCAGACTGATGTTT |
| 26110_1 | 2 | [A/C] | CATGCAGACCTGGCATGTCACTGCAGCTAATGATCT |
| 26116_26 | 27 | [C/T] | ATTTATCTCTCTGCAGGTAGCTGCACCGTCAGATCG |
| 26120_33 | 34 | [A/G] | ACTGTGTGTAGAGCACAGACCTGCCGGCAGCGAAGG |
| 26121_26 | 27 | [C/G] | CATTTAACAACAGCAAATGTCTGCATCGCTGCATCA |
| 26126_10 | 11 | [A/G] | CGATCAGGTCAGGCACGAAGCTGCCAGTCAGCCTGA |
| 2613_17 | 18 | [G/A] | ACTTCAGCTGTTGCATGGCCTTGCAGGACTGGGACT |
| 26132_25 | 26 | [G/A] | GGGGAGGACACTGCAATGAAGTGCCGTCCTGAATTA |
| 26133_30 | 31 | [A/G] | TAAATTTCCCGAGCAGTTGCATGCAGCGGTACCATA |
| 26136_2 | 3 | [C/T] | AGCAACACTGAGGCACTGCCATGCTTCACTGTAAGC |
| 26139_18 | 19 | [G/T] | CAGTGTCTCCCAGCACTCGACTGCTCTCATGGAGGT |
| 26142_19 | 20 | [A/T] | GTTGGTCTAAGAGCATCGAATTGCCACCACTGCTCT |
| 26144_30 | 31 | [T/C] | ATGCCGCACAAAGCAAATGACTGCGAAGCCTGAGGC |
| 26150_1 | 2 | [G/T] | GGCGCTGCGGTGGCACCTTTTTGCACTAGGTCACAC |
| 26151_29 | 30 | [G/A] | AACCCAGGAAGAGCACATTACTGCAGCACGTGACAC |
| 26153_19 | 20 | [C/A] | AGCATGGTGGTGGCAACATCGTGCTTCTGTAGCGTT |
| 26154_6 | 7 | [C/T] | ATACTGCAGCTAGCATGACCGTGCTCCGTTTGACCT |
| 26157_7 | 8 | [C/G] | GTTTCGGGAGCTGCAGGTTAATGCTGGAGGTACGAT |
| 26164_2 | 3 | [G/A] | CAGAAAAAGACAGCATGGAGCTGCTTCAGCCATTCA |
| 26165_30 | 31 | [T/C] | GAACTACATGATGCATGCCGCTGCATCTTTTCAGAT |
| 26166_26 | 27 | [G/T] | GTACTGCTCATTGCATATGACTGCAGGTGCACCCTG |
| 2617_34 | 35 | [G/A] | GCAAGACTAAAAGCACACTGCTGCTGACTGACATGA |
| 26171_1 | 2 | [A/C] | CACACACACACAGCAGAAGCCTGCAGATCGGACATC |
| 26174_3 | 4 | [C/G] | ACTCTGAATCAAGCAATAGTCTGCAGCACCTCTACA |
| 2618_28 | 29 | [G/A] | TTGGGTTTTCTTGCACCAGTGTGCTTCAGTTCTTAT |
| 26185_17 | 18 | [G/A] | CAGCTTGGCGTAGCAGTGAGATGCACCATCTGTTGT |
| 26186_17 | 18 | [T/C] | ATGGTACACTTTGCACCTGCGTGCCAGGCTGAATGA |
| 26187_4 | 5 | [A/T] | ACACTACCTGTTGCACCATCGTGCTTCACATGTGTA |
| 26194_1 | 2 | [T/C] | TTATAGGTGTCAGCATATGTCTGCAGAGGAAACCAC |
| 26198_3 | 4 | [T/A] | TGGTTAAAGAACGCACCACGATGCTGAGAGCTCAGG |
| 2620_15 | 16 | [A/G] | ATGTTCTGATCTGCAAACCTCTGCAAAACCTTGATA |
| 26206_15 | 16 | [G/A] | TCACAAAAACCTGCAGTTACGTGCAGGAGTGTGTAG |
| 26207_27 | 28 | [G/A] | GGAGCTCTTTCTGCAGCACACTGCTCTGATGCTGTC |
| 2621_17 | 18 | [A/G] | TGCTGCCAGTTGGCAATACAGTGCGCCTGTAAGGCA |
| 26213_15 | 16 | [C/A] | AAACTGCAGCTGGCACAACCGTGCCGGCTGTGATAT |
| 2622_34 | 35 | [G/A] | TAGGTCTTAGCAGCACTGGCCTGCCACTGTGTGAGA |
| 26223_24 | 25 | [A/G] | GGATGCCCTCTTGCACCAGTGTGCAGTTTCCCAGAG |
| 26225_17 | 18 | [G/T] | GGGTCTTCCATGGCAAAGACTTGCAGGCCACAATTT |
| 26235_32 | 33 | [T/A] | TGTGATGGATTTGCAGCCTATTGCAGAAACACTGGG |
| 26238_26 | 27 | [C/T] | CCAAAAGGGTGTGCAAACTTCTGCACCCAACTAAAA |
| 26248_24 | 25 | [A/T] | AATGATGATCATGCACTTGAGTGCATCCCAATGGTA |
| 2625_16 | 17 | [A/G] | AAGGCAGAAGCTGCAGAAGAATGCCTTGCTTGTGTA |
| 26253_16 | 17 | [A/G] | ATTCTAAGCCCAGCATAACCATGCAAGTTTCTCAGA |
| 2626_4 | 5 | [A/G] | AATGAACTGGATGCATTAAATTGCCCCTGGGTCTCT |
| 26262_28 | 29 | [G/A] | TTATTACAGAAGGCATTCGACTGCCAGGGAGATTGA |
| 26266_2 | 3 | [G/A] | GAGGCTGTGGAAGCAAACAACTGCAGTCACTGGGAA |
| 26269_2 | 3 | [G/C] | GACGAGAGGACGGCAAATTGATGCGGCGTGCTGCTA |
| 26271_7 | 8 | [C/T] | TTCACAACGGATGCAGTGAGGTGCTTTAACCCTCTA |
| 26272_3 | 4 | [T/C] | CCCCAAGTTACTGCACCCTAGTGCCAGAGAGGGCAA |
| 26274_17 | 18 | [A/G] | GATGACATCTGGGCATCATTGTGCAGCGGCGATTAT |
| 26279_4 | 5 | [G/A] | CCGTATGCGAGAGCACCAAAGTGCCAGAATAATCTA |
| 26281_10 | 11 | [C/T] | TACACAGTATCAGCACATGAATGCGCCTCAATTCAT |
| 26286_26 | 27 | [G/A] | TGGACACCTGGAGCAAGTCTGTGCCTGAAAGAGATG |
| 26298_8 | 9 | [C/T] | GAGCTGCACGCTGCAGTACCGTGCCAGTGCGATACC |
| 26303_15 | 16 | [A/G] | AAGAAAGACACAGCAACTGACTGCTGGGAAGAGGGA |
| 26305_3 | 4 | [C/A] | TGCCTGCTCACAGCAATTTGCTGCCACCACCATGCT |
| 26306_8 | 9 | [A/G] | GAATTTCAAAAGGCAAAGCACTGCGCACCTGGTCAA |
| 26308_26 | 27 | [G/A] | ACTACCCAGGAGGCAAAGCCATGCTGGAACTGCACA |
| 26313_32 | 33 | [C/T] | GGGCACATCTGAGCACATCAATGCAATTTCTTCCTC |
| 26317_33 | 34 | [C/A] | TATGAGAAGTTGGCACAGAGCTGCCTACCTCAGCAC |
| 26321_3 | 4 | [C/T] | ATTCACGTTCGAGCACCGCTCTGCCAGCAATTAAAC |
| 26324_28 | 29 | [G/A] | TCAGAAAATGATGCACTGCAATGCTTGGGAGTGTTG |
| 26327_20 | 21 | [G/A] | ATGGCTTCAACGGCAGACGCGTGCACGCGAACGCCT |
| 26328_8 | 9 | [G/A] | AATTAGCGGGTGGCACAGTTTTGCCGCAGGTAGTGC |
| 26332_1 | 2 | [C/A] | GCTTTTGCTGGTGCACCTGGCTGCAGGTGTGTAGGT |
| 26337_32 | 33 | [C/T] | TCATCCCTTCCTGCAAGACGCTGCTGAACACGCGAC |
| 2634_19 | 20 | [A/G] | GACCCCAGTACGGCAAAACACTGCCTGGGATGTATC |
| 26341_11 | 12 | [T/G] | TTGTTAAGTTGGGCAGATTTTTGCAGCCACTAAGTT |
| 26343_9 | 10 | [A/G] | GAACTACCCATGGCACCACTGTGCCAGCCTAAACCA |
| 26344_3 | 4 | [C/T] | CTTCACAAGTCAGCATCTCGGTGCTCCTGCTCATTA |
| 26349_27 | 28 | [T/G] | CAGGATACTGGTGCATCGGAATGCAGGTCCATAACG |
| 26351_1 | 2 | [G/C] | CGCATGAAGCATGCAAGTCGGTGCGCGAGATAACGT |
| 26364_1 | 2 | [T/G] | CTCAGCAGCTCTGCACAGCACTGCTGTTTCTTGCCA |
| 26368_2 | 3 | [A/G] | AAAAATTCAATCGCAGGTGCCTGCAGCTTCGGTAAA |
| 26370_18 | 19 | [G/A] | AACTCAGGAGAAGCATCCGCATGCCTCCCCTGACGT |
| 26373_31 | 32 | [C/T] | ATGAAGAGGAACGCAAACAACTGCGCTCAGCCGAGG |
| 2638_7 | 8 | [T/C] | CTGGAAGTGCCTGCAGAACAATGCCAGTCACAAAAT |
| 26398_30 | 31 | [T/C] | GGGCAGTTTGATGCAGTGAAGTGCACCCTATACTGC |
| 26400_24 | 25 | [G/A] | TGGAATATTCCAGCATGATGATGCGCTCCATTGTGA |
| 26402_20 | 21 | [C/T] | TGAGATGAGATTGCAGTGGCCTGCACTCTTGGCATT |
| 26403_15 | 16 | [T/C] | TTTTGAACGTGGGCATTGTAATGCTTGACCATGTTT |
| 26404_8 | 9 | [C/T] | GGCTACAACGCTGCACAGCTTTGCCCTCTGTAACAA |
| 26411_19 | 20 | [C/T] | AGCAAAACAAAAGCAGGTGCGTGCGCGACACACACA |
| 2642_25 | 26 | [G/A] | TAAGCAGCAAAAGCAACAATTTGCCGTTTCTTCATG |
| 26420_20 | 21 | [G/A] | ATCTCTAGAGGGGCACCAACGTGCCATAGGGCCGAC |
| 26424_30 | 31 | [G/A] | TGGAAGCTTTGTGCATGTAGCTGCTCTCACGATGCT |
| 26427_33 | 34 | [G/A] | TAATTGTATACAGCATTTCACTGCGAGCTGACAGAC |
| 26430_4 | 5 | [G/A] | ACTTGGCTTTATGCATCCGATTGCAAAAATCTTCTC |
| 26432_5 | 6 | [A/C] | CTACCACAACAAGCACCAGCCTGCTTTAGCACACAG |
| 26444_7 | 8 | [C/T] | AAAGGGCCTCGGGCAGGGGAATGCCTCGTGTCTCCG |
| 26446_5 | 6 | [T/C] | GCAGCTGAAAAAGCACTTGCCTGCACTTCTGATGAA |
| 26449_6 | 7 | [T/C] | GTGTGGTACCCTGCAATGACTTGCTTCCTGTCCCAT |
| 26450_17 | 18 | [A/C] | CTCCAGGGTCCAGCAGCAGCATGCTTTACATCACTT |
| 26460_33 | 34 | [G/A] | AGGTGGGAGTTAGCAAGGATTTGCAAAAGGGGTGAT |
| 26461_34 | 35 | [G/C] | TGGCTAAACCCTGCAGGCTGATGCCCAAATAAAAGT |
| 26463_5 | 6 | [C/T] | GATGGCCACATCGCAGGCGAGTGCGTTATCTTTCGA |
| 26465_18 | 19 | [C/A] | ATGCGAAACCGGGCACAAAGGTGCCGTGTCGAGATC |
| 26468_19 | 20 | [C/T] | TGATAATTCCTGGCATGATCCTGCGCAGCTGCAGCA |
| 2647_28 | 29 | [G/A] | ATCCCAACACATGCATGTACATGCAGTGGCCTGCAT |
| 26484_16 | 17 | [C/T] | AGTAGATCTCCTGCAGCGCCATGCTCCACATTTCCC |
| 26492_28 | 29 | [C/A] | TTACAAGTGACTGCAAATTGCTGCAATCCTCACCAT |
| 26501_7 | 8 | [C/T] | AGCAGCACATTAGCATGGTCCTGCATTTGGAACTGA |
| 26507_11 | 12 | [T/G] | TAGAGTCAGTCTGCATCTCTGTGCTTGTGTACATGT |
| 26509_29 | 30 | [G/A] | TTGGTGTCTCCAGCAGTGCTGTGCTGTGCGTTTACT |
| 26512_15 | 16 | [T/C] | GGGGCACAGTTGGCATCTTAGTGCATCCCAAAAGTG |
| 26515_31 | 32 | [C/T] | TGTTCTGAATGTGCAAATCCCTGCAGCCATTCTTTA |
| 26519_28 | 29 | [G/A] | AATGCGTACCAAGCACCCTGATGCAGATGAATGTAA |
| 26525_2 | 3 | [A/T] | AAAAGTCGCGGCGCACCGGAGTGCGACATGATCAGA |
| 26528_26 | 27 | [A/T] | GCACAACTCCATGCATTCGGCTGCTGAAACATCTCC |
| 2653_31 | 32 | [T/A] | AAGTGCTTTGTGGCATTGCATTGCGTAACTCTCCAG |
| 26535_29 | 30 | [A/C] | ACAGCAGATGCAGCAGTAAACTGCCCTTCACCCATC |
| 26540_24 | 25 | [G/A] | TGAGCTAACTCAGCAGAGAATTGCGAAGTGGACGTT |
| 26541_18 | 19 | [G/A] | TTAGGACGCGGTGCACACGACTGCTTCTGGGCCAAA |
| 26553_4 | 5 | [C/T] | AACTCCAGGTTCGCATAGCGATGCTCGCAAACCCCC |
| 26556_6 | 7 | [C/A] | CAACTTCCCCTGGCAACTAACTGCCACGACCACGCG |
| 26557_9 | 10 | [A/G] | GTTGTTTTAAAGGCACGGACATGCACGGGTGCGGAG |
| 2657_20 | 21 | [T/G] | TTCAGTTATCATGCAGGCTATTGCAGCGCTGGATCT |
| 26576_33 | 34 | [G/T] | TAGGCTCTGGGTGCAAATACCTGCAGAGTAAAAGAA |
| 26577_9 | 10 | [C/T] | AATGCGTTTCAGGCATGAGTGTGCGCGTTTCAGCAT |
| 26579_11 | 12 | [C/T] | TGCACACGTGGCGCAGATATCTGCCGCCTGTATTCT |
| 2658_16 | 17 | [A/C] | ATTGGAGTGGTGGCACAGAGATGCCATTTTCTCTTT |
| 26580_27 | 28 | [G/A] | ATGGGGTTTCCAGCAGAAGACTGCTGCGTAAGCATA |
| 26581_5 | 6 | [G/C] | TCGGCGGCGGAGGCACGGGCATGCAACTCGCAGAAC |
| 26582_16 | 17 | [G/T] | AGTTGTCTACCTGCAGGGGTGTGCTTTCAGCAGACA |
| 26583_3 | 4 | [C/A] | TTACGAGGCGCAGCAAAAAATTGCGACGGAAAACAG |
| 26587_30 | 31 | [A/G] | TTTACAAGCTTAGCACAAATATGCAGCCTCGCCCAC |
| 26589_15 | 16 | [C/T] | AATTTGTAAGTGGCACGGTGGTGCCAAAGTTTTTGT |
| 26594_11 | 12 | [A/G] | GCACAACGTAGGGCAAGATTCTGCACCTGGTCACTA |
| 26597_17 | 18 | [G/A] | TGGCTCTAACAAGCAAAGGCATGCCATGTCCCCTCT |
| 266_4 | 5 | [A/G] | TACAAAGCAAAGGCAAGTGAGTGCACAGCTTATTAT |
| 2660_18 | 19 | [G/A] | GAGGGTGACAAGGCAACCGCATGCTGGAGAACTACT |
| 26602_16 | 17 | [G/A] | CGTCTATCCAGGGCACGACGTTGCCTGGCATGTAGG |
| 26605_18 | 19 | [A/T] | GAATGTATGCATGCACAGAGCTGCTTGCGTAAGCGC |
| 26609_31 | 32 | [G/A] | GAGTAAATGAAAGCAAACAGATGCCGCTTCTGAGAT |
| 26614_20 | 21 | [T/C] | ATCACGGCCTTGGCAATCTGTTGCAACTGATTGCAA |
| 26619_6 | 7 | [G/A] | TAACATGACAAGGCAGTGTGCTGCAGCCTATTGTAA |
| 26621_28 | 29 | [A/C] | TTGACATTAACGGCAGGAATTTGCACCCAGAGTTTG |
| 26622_3 | 4 | [A/G] | TTCATGCTTTCTGCAGAAAAGTGCATGATGCAGTGA |
| 26623_20 | 21 | [G/A] | AAACCTGCTCCAGCATGATGGTGCTCCTGTGCACAA |
| 26630_4 | 5 | [G/A] | TATCGTCATATGGCATCGTCCTGCTGGACTAGAAGA |
| 26632_19 | 20 | [G/A] | ATTACCTTCCCGGCACTGCGGTGCTTCTGGTAAACA |
| 26636_33 | 34 | [G/A] | GCCGGGCATATAGCATTGCAATGCACAATGCACGGA |
| 26637_15 | 16 | [A/C] | CAGAGAGCTTCAGCAAGCAGCTGCTGCTGAGCCTCT |
| 26638_18 | 19 | [T/C] | ACCCATGAATTTGCAAACTTCTGCCTTGTGTAGGTC |
| 2664_3 | 4 | [A/G] | AACATGTTCTACGCAATGACATGCTAATAACCGCCT |
| 26642_2 | 3 | [T/A] | CATAGATGATTAGCAGCTCTTTGCCTGCAGCAGGAA |
| 26644_5 | 6 | [T/A] | GTTGCTGCTGGGGCAGGGCCATGCATTACGTTTAAT |
| 26654_31 | 32 | [T/G] | ATTTAATGCTAAGCACAAAAGTGCAGCAGGTTCCCT |
| 26661_31 | 32 | [A/G] | ACCCAAGCTAATGCACGAGTGTGCCAACGCTAAGAG |
| 26662_32 | 33 | [A/G] | TTTCTCTTCCCTGCATGAAGGTGCACACATACAGGA |
| 26665_16 | 17 | [G/A] | ATTCTTGGTCCAGCAAGCAGTTGCCAGGGGCCTTTT |
| 26666_1 | 2 | [T/C] | GTCTGTGATTGGGCACCCTGTTGCTCCAGGGGCCTC |
| 2667_5 | 6 | [T/C] | AAACCTCAACAGGCATGCTTCTGCCTTTTAAGGAAC |
| 26675_6 | 7 | [G/C] | GATGAGGAGGGGGCAGATTCTTGCTCGAGTCACCAT |
| 26684_3 | 4 | [T/C] | CTGTGCGTGGATGCAGCTCGCTGCTGGTGAACACAC |
| 26685_10 | 11 | [A/G] | ATTGCCATGGAGGCAGAGTGTTGCACTGGCAGGATA |
| 26687_19 | 20 | [C/T] | TTTGACTTCCCAGCAGCTGCGTGCGGTGCAGGGAGT |
| 26688_31 | 32 | [C/T] | TGTACAGGCAGTGCAATACTTTGCATGTCAGCGTGA |
| 26690_3 | 4 | [C/T] | TTTCAGATCCCGGCACTAAGCTGCCACTTTTGGCCC |
| 26698_1 | 2 | [T/C] | CTAATGGATCTAGCAGAGCTCTGCAGGCCACACTAG |
| 26699_30 | 31 | [G/A] | CACTCCCCAATAGCAGGGAAGTGCTGTGATGGCCAA |
| 26702_1 | 2 | [A/G] | AAGAGAATAAAAGCAAGCAGCTGCTCATGCCTCAGT |
| 26705_20 | 21 | [C/A] | TCGCACTCCTCTGCAATCCCATGCTCTTTCTCTCAG |
| 26707_15 | 16 | [C/G] | TAAATGCCCTGTGCACAGGGTTGCCAGGTCCAACAA |
| 26709_24 | 25 | [C/T] | TGAGAAGCAACAGCAATAAATTGCCCCTGCAGGCTT |
| 2671_15 | 16 | [C/T] | AATTAGACACGTGCACAGAGCTGCTGCTAAAACCCT |
| 26712_9 | 10 | [A/G] | AATTTGAATACAGCAGGAACGTGCTTGCAAAGAGTG |
| 26714_34 | 35 | [C/T] | TCTTCATAACTGGCAACTGGCTGCTCTGTTCCTTTA |
| 26719_18 | 19 | [C/T] | ACTATACCATTAGCATGTCACTGCTCCCTGCGGTTT |
| 26729_7 | 8 | [G/A] | GAGGCCCGTCATGCACACTATTGCTGTGCTGCGAAC |
| 26736_18 | 19 | [C/T] | ACTTTACCTGTGGCACCACCATGCAACTCAGTGCTG |
| 26741_8 | 9 | [A/G] | TGGGATTGAGCAGCACAGTGGTGCCGCAGGTAGTGC |
| 26748_7 | 8 | [G/A] | GCTACGGGAACGGCAACCACGTGCACGGCTCGAACC |
| 2675_3 | 4 | [G/C] | GCCGGGCAAAGGGCAAAAGTCTGCTGCACATCCCAT |
| 26750_4 | 5 | [G/C] | GGTTGATCTACTGCACTGCATTGCATGTTGGCGGTA |
| 26756_8 | 9 | [C/T] | TTGCTCAGCGTAGCAAATCTGTGCAACGGAAGACAA |
| 26766_16 | 17 | [C/G] | GCAGGGAAAACAGCAGCGCAATGCCAGATCCCTTAT |
| 26770_29 | 30 | [A/G] | AGTGCACGGTTTGCACCTGTGTGCAAGTCAAAGAAA |
| 26775_25 | 26 | [G/A] | TGGCACACTGGGGCACAGTCATGCCGGTACAGAAAA |
| 26777_18 | 19 | [C/T] | ACCTCAGTGAAAGCAGAGCGCTGCCAAACAGCCCCA |
| 2678_5 | 6 | [C/T] | CCAATCCTGTCTGCAGCCTCCTGCAGTAATTAGTTT |
| 26784_18 | 19 | [G/A] | ATTCGTGCACATGCAAGCGAATGCAGAAATGGCAAG |
| 26785_27 | 28 | [T/A] | ACCAATGTTCGAGCATGACAATGCACCTGTACACAA |
| 26787_29 | 30 | [C/G] | TAATAGCAAAGCGCAACCTGATGCAACCGCAACGCT |
| 26790_16 | 17 | [G/A] | TGACACTCCTGGGCATGGGGTTGCGAGGGTACGGGA |
| 26793_4 | 5 | [C/A] | TGCACCACGTTTGCATATGCATGCTGAGGAGTGCAC |
| 26794_26 | 27 | [C/T] | CTGCATGAACTCGCAGCCCTCTGCACTGAGACTCCA |
| 26795_15 | 16 | [C/T] | TCCGGCAACGCGGCACGATGTTGCGCCAAACAAATG |
| 26796_20 | 21 | [G/T] | CCAGGCCCCAGGGCAAAATTTTGCAGTTTTATATGG |
| 26801_29 | 30 | [T/A] | CGTCTTTGTTCAGCAGACACGTGCTGGTCAGGAATA |
| 26804_26 | 27 | [G/A] | TCAGACAGAGCTGCAGCAAACTGCCTGAGAGAGACA |
| 26805_4 | 5 | [G/A] | GAGCGAGCAGATGCAGCTCCATGCCAGAGGGAGGCT |
| 26807_8 | 9 | [C/T] | TAAGATGCCTGTGCAGTCGAATGCAGGAAAAGAAAA |
| 2681_6 | 7 | [C/A] | GCACAACAAAATGCAAAATGGTGCAACAGGCAGAAG |
| 26810_31 | 32 | [T/C] | CAATCCTCAGAAGCAAATAGATGCGCCAACCTGCTG |
| 26811_30 | 31 | [C/T] | AATACAGCATCAGCAACACAATGCTGACCCCGGGGG |
| 26813_4 | 5 | [C/T] | TGTGCGCTCGGTGCAGCAGGATGCGGTAGACGCCGG |
| 26816_24 | 25 | [C/A] | ATGGTGAAGAGAGCAGGATACTGCCTGTCTAGGGTT |
| 26820_25 | 26 | [C/T] | GATAAATGCAGTGCATTGATTTGCTCAGCCCCTCCT |
| 26823_30 | 31 | [T/C] | ACATGTGACTGAGCACAATGTTGCCTTTCCTATGCG |
| 26830_19 | 20 | [G/A] | TAAAACTGCCTGGCATGCCGTTGCTCAGGTCTGTCT |
| 26832_32 | 33 | [A/C] | ATTCCCACACTTGCAGCCTATTGCACCAGCTCATCT |
| 26839_28 | 29 | [G/A] | TACGTCGTAAGGGCAGCGACGTGCATTCGAAGATTT |
| 26842_10 | 11 | [T/C] | GGAAGTTCATTGGCACGGTGTTGCTTTTTGTGCTGT |
| 26846_24 | 25 | [A/G] | AACGACTCGTACGCATGTGCATGCAATCGAAGCTGT |
| 26851_32 | 33 | [A/T] | AAGGTTTTCTGTGCATTCGTTTGCTGTACGGTATGC |
| 26852_20 | 21 | [C/A] | CTGTTTATATGTGCATGGCGCTGCAGTGCGCCCTCT |
| 26859_11 | 12 | [A/G] | CAAATCTTCTCAGCAGCAGGATGCAGCACAAAGTCT |
| 2686_6 | 7 | [C/T] | GTCTGTCAGTCTGCAAGGTTGTGCTTATCTTACCAC |
| 26860_11 | 12 | [A/T] | AATCAGGCGAAAGCATTGCTTTGCAGTCTAGTGTGT |
| 26867_9 | 10 | [G/A] | CACCTTAATGCAGCACCGCCATGCCACCCATCAAAT |
| 26869_28 | 29 | [G/A] | GAGGCCATATCAGCAACTACCTGCTAGAGAAGGTAG |
| 26870_2 | 3 | [G/A] | GGGAACCGTGGGGCAGACTGCTGCGCCATCCTGCTG |
| 26877_1 | 2 | [G/A] | AGATTAGCTGCAGCACCACCTTGCTGCTCTACTGTA |
| 26878_17 | 18 | [G/A] | TAAGGCTGATATGCAATGATCTGCAGAAGAGACCAG |
| 26884_26 | 27 | [G/A] | GGACGGGGAGAAGCAGCAGGCTGCAGGACAGATAGC |
| 26891_24 | 25 | [A/G] | AGTACAGGCATTGCAGGGCACTGCAGGGCAGAACAG |
| 26899_29 | 30 | [G/C] | CATTGTGTACCAGCACCAGGATGCATCCTGAACTAG |
| 26900_11 | 12 | [C/T] | CATTTTGGTTTCGCACACAGTTGCTGAGTTTTGCTG |
| 26903_2 | 3 | [C/T] | CTTACGACCGAAGCAGGGAGCTGCATGTAATTGATT |
| 26905_11 | 12 | [C/T] | GAGATAATGACCGCATCCATGTGCTCTTGGCTCTGA |
| 26906_31 | 32 | [T/A] | CCTCCACAGACTGCACTGGCCTGCGTGGATCTTTAT |
| 26911_24 | 25 | [C/G] | GCGTGAGCGGAAGCAGGTCGGTGCCGGCAGATCCAG |
| 26917_20 | 21 | [A/G] | CTTACACACACTGCAGAAACATGCAGGATGAGCACA |
| 26923_19 | 20 | [G/T] | GGTCACGTTCATGCAGGAGGATGCGCTGCTGAAAAG |
| 26925_30 | 31 | [C/T] | AATTCGGCTGGTGCAAATGTCTGCAGAATACTAGGC |
| 26929_2 | 3 | [C/T] | GCCGGATGATGAGCAATTTACTGCAGTCAGGAGACG |
| 26930_2 | 3 | [G/C] | TTGTAGCGAATGGCACACACGTGCTCATGGGGAAAA |
| 26931_28 | 29 | [G/A] | ATAAGGCAAAGGGCAGGAATATGCTCCTGGGCAGGG |
| 26941_10 | 11 | [T/C] | AGCTGGAAGATGGCAGGGTGTTGCTCGACACGTGGT |
| 26942_5 | 6 | [G/A] | ATGCTGAAAGCTGCAGAGCTGTGCTATGCTGTGTGT |
| 26944_17 | 18 | [C/T] | TCAATAGCACAGGCATCTTGCTGCACCTCTGTAACA |
| 26945_30 | 31 | [C/T] | CACACACTGCGGGCATGGTTATGCCGGGGTCAGAGT |
| 26947_4 | 5 | [C/T] | CAGCTGTCTGTAGCACAATCATGCATTCTTTCTGAC |
| 26949_1 | 2 | [G/A] | TGGCTGATAAGGGCAACTATGTGCTGCCACATGTCT |
| 26951_2 | 3 | [C/T] | CACGGTTGTTGTGCAAACAGCTGCACTGCGCCTTGT |
| 26961_29 | 30 | [T/C] | AGTGGAGTTAATGCACACACATGCCTGCCTACACAC |
| 26963_19 | 20 | [C/T] | ACATTCCCTACAGCACCACCGTGCCACCCTAATTTT |
| 26970_31 | 32 | [T/G] | GCCCTTTCCACTGCATTGGGTTGCGGCTCTCTACTT |
| 26972_15 | 16 | [T/G] | GGCACCCATGGGGCAGGGACATGCTTGGGTTATTAA |
| 26974_26 | 27 | [G/C] | TCCAGAGTTCCTGCAGCGTGCTGCCCGGGGTTCTCT |
| 26978_31 | 32 | [T/C] | GAGCAATGAGAGGCACAGCGATGCGGTGTCCTGCCC |
| 26984_3 | 4 | [C/T] | ACACTCTTTATCGCACAACGATGCAAAAAAAGCACA |
| 26989_2 | 3 | [T/C] | AGTTAGTACCTGGCATTTCGATGCAGACTCCATCGT |
| 26996_1 | 2 | [C/T] | GCGGAGTCAGGGGCAGGTGCATGCGGCTTGAGGCAT |
| 27003_17 | 18 | [G/A] | AAAACTATATTGGCAACGCTGTGCAAAAGTCGAGGC |
| 27006_6 | 7 | [C/T] | CCCCACCGCCATGCACATTGCTGCAGCTAAGGAAGT |
| 27007_3 | 4 | [G/A] | GTGGAAGAACTTGCAGTGTCCTGCACAGAGCCCTGA |
| 27019_3 | 4 | [C/T] | GTCCTCAGTGCGGCATATGGCTGCAGCGGCGCAGCG |
| 27020_30 | 31 | [C/A] | ACACCTTGTAGAGCACTACACTGCTCCCAGCACTCC |
| 27021_27 | 28 | [C/T] | AGTAAGGTCAGAGCAGCACTTTGCTCTCTCAGTGAC |
| 27024_4 | 5 | [T/C] | GACCTTCAAAATGCAAAGTGTTGCCCTCGAGGACAC |
| 27026_20 | 21 | [C/T] | ATTCAGCATCAGGCAAATGACTGCACTGCTTTGTGT |
| 27029_24 | 25 | [G/A] | TTCAGTAAGACAGCAAAGTGCTGCGAAGTGGAAGCG |
| 27030_25 | 26 | [T/G] | AAGCTTGTGTGGGCATCTGTATGCTTGTGGGTGCAT |
| 27031_10 | 11 | [G/A] | ACCCACCTACGTGCACACAACTGCATTTCTGAAGCT |
| 27044_9 | 10 | [G/C] | GAGTGAAGAGAAGCAGCAGGATGCAAGGACTTGTCA |
| 27052_3 | 4 | [T/C] | CTTTATCTGCCAGCACAATTTTGCGTCTGTCTCCTC |
| 27053_19 | 20 | [C/T] | AAAGCACAAAGCGCACACTTATGCACACACTCACAC |
| 27054_8 | 9 | [T/G] | CCTGATCATGTTGCAGTAAAGTGCTGGGTGACCAGA |
| 27055_6 | 7 | [G/A] | TGTAAGGAGCGTGCAAGGAGGTGCCAAAACTTACGC |
| 27056_11 | 12 | [C/T] | AGAAAAGTTCACGCAGCCATATGCTGTTGTGACACT |
| 27058_9 | 10 | [C/A] | CCACTGCTCCAGGCATGTTTGTGCATGTGATTTCTG |
| 27060_10 | 11 | [G/A] | GTGTATTCCTGAGCACGAAGATGCCCGTTGGGTTCC |
| 27071_16 | 17 | [C/T] | AGATGGCGGCGTGCAGCGGCGTGCGGCCCATGTTGT |
| 27073_28 | 29 | [G/A] | TGCTGAAAGGAAGCACGCACATGCGCGTGCACACAC |
| 27077_26 | 27 | [C/T] | AAGGGAAATGAAGCACAGCTTTGCACCGTCATGGCA |
| 2708_27 | 28 | [G/C] | GGCGTGTCAGCAGCAGGAGTGTGCGTCGTGGCAGTT |
| 27080_33 | 34 | [G/T] | GCTTCTTATACAGCAACGGCTTGCATGTCTCGGGTG |
| 27081_18 | 19 | [A/G] | GGCTGAGCTAATGCAGAAATCTGCTGACCTGAGACA |
| 27084_7 | 8 | [T/C] | AGGTCAACCATGGCACTCAGGTGCACCACCAAGATA |
| 27089_26 | 27 | [A/C] | AGATCCCTGTTGGCACAAAACTGCACACAATAGGAA |
| 2709_25 | 26 | [G/A] | TGGCTGTTGGTCGCATTGTGTTGCAGGGCATAAGCC |
| 27090_19 | 20 | [G/A] | AGCAGGAGTAACGCAGAGCGCTGCTTATTTCTGTAG |
| 27095_26 | 27 | [G/T] | TGCAATGTTTACGCATCCGCATGCTTGAGGAGCGCG |
| 27101_18 | 19 | [C/G] | ACCTGTTCCTTAGCACCGCCATGCCGCTCCTCACCG |
| 27103_20 | 21 | [T/C] | GAGAAGATTGTTGCAGAAGTTTGCAGTTTGTCCTGT |
| 27104_1 | 2 | [C/A] | ACACTGGCGGTGGCAGTGTCATGCTGTGGGGTGGTT |
| 27106_26 | 27 | [T/C] | GCTACTAGATTAGCATCGGTATGCTCTGGTACCGAC |
| 27111_18 | 19 | [C/A] | GGCCTACCAGCAGCAACTCTATGCCCTATCCAGTCA |
| 27112_26 | 27 | [G/T] | AAGGTTCCCATTGCAGCACAATGCGAGAGTTTCTGA |
| 27115_9 | 10 | [A/G] | CACCAGAGGACTGCACAAGGTTGCCCCCCAGTGAAT |
| 27116_5 | 6 | [G/T] | TGTGTGTCGTAAGCATTATGGTGCGGATTGCATTCC |
| 27122_31 | 32 | [G/T] | ATCTGTATGTAAGCAACACACTGCCTGATCTGCAGC |
| 27128_6 | 7 | [C/G] | GAAAGGCTGCGTGCATCTTAGTGCACGCCTGGCTCT |
| 2713_4 | 5 | [C/T] | GATGCTGGATGGGCAAACATGTGCATGAAAACACCC |
| 27133_9 | 10 | [G/T] | CTACCGCCTGGTGCAGTCTTTTGCAGTCGTCCTGTT |
| 27134_9 | 10 | [G/A] | AGCAGACTCGTGGCATCAGCCTGCAAGTCACTCACA |
| 27137_7 | 8 | [T/C] | ACTTGACTTGTAGCATGTTGCTGCTGTGGAACCTCC |
| 27146_18 | 19 | [C/A] | GACATCTCGGTGGCAGGTCGCTGCTACACCTGGAGC |
| 27147_25 | 26 | [G/A] | ACGCATTAGAATGCAGAGATATGCAGGCCTGCAGGC |
| 27152_26 | 27 | [C/T] | TGTTATAACAAGGCACTGCGGTGCTCCGTCGAACCC |
| 27155_26 | 27 | [C/T] | AGTGGCAGCTTGGCAGTGCTTTGCCTCGAACCCCCA |
| 27158_4 | 5 | [G/A] | TAACGGGCTTTGGCATCAGCATGCATACCAAGTTGT |
| 27164_20 | 21 | [C/T] | TGGACCCTCATAGCATGACACTGCCACTATCATCTG |
| 27167_25 | 26 | [C/G] | AGTAGCCACTCTGCAGTATTCTGCTCAGAGTTATGT |
| 2717_26 | 27 | [C/T] | TGCCCAATACATGCACATGGCTGCCCCCACACTCCC |
| 27176_20 | 21 | [A/T] | CGATCAATTCTGGCAGGGGTATGCGTCAGGAAATAA |
| 27179_25 | 26 | [C/T] | ACTTCTCCTCCTGCAGGTAGCTGCTCAGAACGTCCT |
| 2718_19 | 20 | [G/A] | ACTCGTGTGTAGGCATTAAGCTGCGCGAAGCCAACC |
| 27180_24 | 25 | [A/G] | CTCCTCGGCTCAGCATCTGCGTGCACACACCCGGAC |
| 27182_9 | 10 | [C/T] | TTGTTCCTCCGTGCACTGTGGTGCAGATGGGACTTG |
| 27188_32 | 33 | [G/C] | GTACTCCGCCTTGCAATGTGCTGCTCCAGGAAGGCC |
| 27190_30 | 31 | [T/A] | CATTCGGTTTTAGCATCGTGTTGCACTGGGTCAAAC |
| 27197_24 | 25 | [A/G] | CATCATCCGGACGCACAAGTGTGCAAATGCTTAAGC |
| 27198_15 | 16 | [C/T] | ACATTGTCCTACGCACCTTTGTGCCGTTCATTCAAG |
| 27204_3 | 4 | [C/A] | GCTCTCGGTGGAGCAAAGGCCTGCTTTCCATCCACC |
| 27212_34 | 35 | [G/A] | ATATCCTGTGATGCATTGGCATGCTGTCCTGAGTGG |
| 27214_19 | 20 | [G/A] | CTGGCTATAACAGCACAGCGGTGCTCTTAGTAGCTG |
| 27216_7 | 8 | [C/T] | TTGGGCTCAGGGGCATTATCATGCTGATACAGGTCT |
| 27218_28 | 29 | [A/T] | AAGCTAATTCAAGCACAAACCTGCCAAAAAAGCTTA |
| 2722_20 | 21 | [G/A] | GGTTGTGGTGCTGCAGCACCGTGCGCACATGTGCAG |
| 27220_26 | 27 | [C/T] | CGTAACACACTGGCAGCATCCTGCGACGAGCTCTGA |
| 27221_17 | 18 | [G/A] | GCTCAGTACAAGGCACTGAAATGCACTCCACCTTTT |
| 27230_19 | 20 | [C/T] | TTCCTAAAACAAGCATTGTCGTGCTCATGTTCCAGA |
| 27231_3 | 4 | [C/T] | TGACTCTCATCTGCAACCCAGTGCTGAACTCTTAAT |
| 27237_34 | 35 | [C/T] | GCGGTGTCCTCGGCAGCCAGCTGCTGAATCCCTTCA |
| 27239_19 | 20 | [T/C] | GTCTGTGACCTTGCATACATGTGCAGAAGTACCAGT |
| 27240_30 | 31 | [A/T] | TCTCCAGTTTGTGCAGCAGCGTGCTTTCTTAACCGA |
| 27247_32 | 33 | [A/C] | AATACACACCATGCACAACACTGCAGGCACACCACG |
| 27250_34 | 35 | [G/A] | GCGAGCTCGGCGGCACGCGGTTGCAGGCTGCTTGGA |
| 27256_31 | 32 | [A/C] | ATGGAGAGGAGGGCACTAAAATGCCTAACAAAGCCG |
| 27257_3 | 4 | [G/A] | ACTGAAACACCTGCACACCGGTGCGTTCGTGCAGTT |
| 27267_3 | 4 | [G/A] | ACAGCTAGCAAGGCATGCTATTGCTATCCCCCAGGA |
| 27270_4 | 5 | [C/T] | TCCACATAGATGGCAGTGGTGTGCAGTGCTCATTCT |
| 27271_27 | 28 | [A/T] | AGCACAGAGGACGCAGAGATCTGCTGCAAACTGTGT |
| 27275_7 | 8 | [G/A] | AGGAGATGAAAAGCAGTGCTGTGCTGCTCTGTCTCT |
| 27281_8 | 9 | [T/C] | AGACAGACTCGAGCACGACGATGCTTTCATTATCAA |
| 27284_19 | 20 | [G/A] | CGCTATTTTGCGGCACAACGATGCGGACCCGCACCG |
| 27285_16 | 17 | [G/A] | ATTAGATTTTCTGCAGGGACATGCTGATGGCAGGCT |
| 27288_4 | 5 | [T/A] | ACTGTCAGGTCAGCAATCCAGTGCTCCAGAATGTTC |
| 27290_20 | 21 | [C/T] | GCAGGGGTCGCCGCAGAGCTCTGCTGCTGGGCGGGG |
| 27293_10 | 11 | [C/A] | GAACCCAAAACCGCAGGTTAGTGCTTACACATGACC |
| 27301_18 | 19 | [G/A] | TAAAGATTTGCCGCATGAGAGTGCCTCCGACATGAA |
| 2731_27 | 28 | [G/C] | CTCATTTTCTTGGCACACGTCTGCGTCGATTAATAT |
| 27313_28 | 29 | [C/G] | CACGAAGACGTCGCAGACGGCTGCCGCGCCGACGTC |
| 27315_7 | 8 | [C/T] | CTCAAAACGATGGCATAATGCTGCCACCTGCTGGCT |
| 27317_17 | 18 | [A/G] | TGCCAGAAAATGGCAGCACGATGCCGAGATGCCGCG |
| 27322_16 | 17 | [G/A] | AAAAACCTATACGCACGTTTCTGCCAGGCCTGTTAC |
| 27325_10 | 11 | [C/T] | TGCCACACTTCAGCACCCCCTTGCGTGACCGTAAAA |
| 27329_16 | 17 | [C/T] | AAAAGTGTACCAGCAGCGTGTTGCGTATGACAGAAT |
| 27332_8 | 9 | [C/T] | ATACTGTACGGTGCATACGAATGCAAATTCCACCGA |
| 27333_11 | 12 | [G/T] | GTGATGTCCTAGGCAGTCCTATGCATGAGAGCTTGT |
| 27335_3 | 4 | [A/G] | CCCAGGGTGTTGGCAGGAATATGCAGTGCTGTGTGA |
| 27336_25 | 26 | [C/T] | ACATCTTGGCATGCAAAAGCCTGCTCCTTCCAGAAA |
| 27346_25 | 26 | [A/G] | TGGACTCAGGTAGCATCAGGTTGCTAGGCAACTTCT |
| 27357_17 | 18 | [C/T] | ACAGCTTGGCTTGCAACCTCCTGCATCATGTCCTGC |
| 27361_1 | 2 | [T/C] | GTGTAATATGTCGCAACGCTCTGCTACGAATTAAAT |
| 27362_29 | 30 | [C/T] | ACACAGGGATACGCATCGACATGCACAAACGCACAC |
| 27363_34 | 35 | [G/A] | GAAGTATTCTACGCAATCCAGTGCCCGTGGAGCAGT |
| 2737_16 | 17 | [T/C] | AAGGTCATAACAGCATTAGGTTGCTCTGTGTTTGTC |
| 27372_5 | 6 | [C/T] | AACACCCACACAGCATCATTCTGCCACCACAAACCT |
| 27376_31 | 32 | [G/A] | TTTTAGGCTGGAGCAACATAATGCCTGCCCAATGTA |
| 27379_25 | 26 | [T/G] | TGGCTCCGTTGCGCACTGTGCTGCTTCTTTTTCTCT |
| 27382_1 | 2 | [C/G] | ACAGGAGGAGGTGCACACAGCTGCCAATCACATATT |
| 27387_6 | 7 | [A/G] | TGTCTCAGTGTCGCACGCCCCTGCAGCTTTCTTTAG |
| 27388_34 | 35 | [C/T] | GCACTGCTGACAGCATAGACGTGCACCACTGGCACA |
| 2739_28 | 29 | [A/T] | GTCTTGCATTCTGCATGTGTATGCTCGAATGGTTTT |
| 27393_32 | 33 | [A/G] | GCTGGTCAAGCTGCATGCTCTTGCCAAACCCAAAGT |
| 2740_28 | 29 | [T/A] | CACGTACACACAGCAGTATGTTGCTATCATTGCAGG |
| 27400_26 | 27 | [C/T] | GGAATGGTGTTAGCAAAGTCCTGCAGCGGAGAAGAG |
| 27403_1 | 2 | [A/T] | AAAGAAGAGCATGCAGCAATGTGCACTTGTTTCCCA |
| 27405_5 | 6 | [G/T] | GTCATGTTTACAGCATTTCTTTGCACACGCCTGATA |
| 27413_6 | 7 | [T/C] | TGCGTGTCCCAGGCACTCATCTGCTGAAATCCGCAA |
| 27415_5 | 6 | [T/C] | CTTTTTACTTCAGCACAGACTTGCATACGGTACCTA |
| 2742_11 | 12 | [T/A] | TACTAGGGTGTTGCATCAGGTTGCATCTGGTGTAAA |
| 27422_18 | 19 | [A/G] | CATTAGACACACGCAAACACGTGCGTGCACACACAC |
| 27423_7 | 8 | [T/G] | CCTTCTCTGTTAGCATGTTGCTGCTCGCCTCTAGAC |
| 27430_34 | 35 | [G/A] | ACGCTAGAAGAGGCAGACTGTTGCATTGTGGAAAGA |
| 27432_15 | 16 | [G/T] | GTGTGTGTGTTGGCATTTGCTTGCTTTGGCACACTT |
| 27434_19 | 20 | [T/G] | ACCTGAAGCCGTGCAACGTTTTGCTGAGCAGCCGAG |
| 27435_8 | 9 | [T/C] | GGCGGCATTGGTGCATCATCGTGCCAGGGTCTGGAA |
| 27440_3 | 4 | [C/T] | CTTCAGATTGGTGCAGTTACCTGCCCACTGTATCCG |
| 27443_8 | 9 | [G/A] | CACACTACGCACGCATGCAGCTGCCAGTAACAGGTG |
| 27444_8 | 9 | [C/T] | TGGCAGTTCTTAGCAAAATTGTGCAAGCTCTGTTAG |
| 27448_24 | 25 | [T/A] | TAAAATCCTACCGCATGTGTGTGCTCAGGACAGAGA |
| 27449_19 | 20 | [C/T] | GACACCGGCCGAGCAAGATTATGCGCTTTGTAGACA |
| 27455_16 | 17 | [G/A] | AAACTGGTACAGGCAAGCTTATGCAGCCTTTACATA |
| 27458_19 | 20 | [T/C] | GCTGGTCCACTTGCATGTCTGTGCCTGTAAATCTCT |
| 27471_18 | 19 | [A/T] | GATGCAAAGCCAGCATGGTCCTGCATTGCATGCACA |
| 27473_3 | 4 | [C/T] | CCTCACTGCCCCGCACGCCTCTGCCAGGCAGCCGGG |
| 27475_24 | 25 | [C/A] | AAGCACATTTGAGCAGCACAGTGCCGTAAAAAAGTA |
| 27477_6 | 7 | [C/G] | CTCAGACCCACAGCAACACAATGCTCTGATCCAAAG |
| 27478_4 | 5 | [T/C] | CAACTTGGTGAGGCATTTAGATGCCTGCGAGACCAT |
| 27480_27 | 28 | [C/T] | TCGATGGGGTATGCAAACTTTTGCTCTCTGTCTGTA |
| 27483_31 | 32 | [G/A] | ACAGTGTCTGAAGCACTGCCCTGCAGAGAACGAGAG |
| 27485_10 | 11 | [A/T] | CTTGGAGGTGATGCAATTTCATGCTTCAGGCTTTAT |
| 27487_2 | 3 | [G/A] | ACGCACCCCACAGCACTCCAATGCCATAAGGTTTAT |
| 27488_7 | 8 | [A/G] | GCATGGTGCCTGGCAGACCTCTGCATTGCTAAGCAG |
| 27494_34 | 35 | [T/C] | TCGTGGGAGAAGGCAATGAGCTGCAGTGTTGTCTTA |
| 27496_26 | 27 | [C/A] | TAGCAGATTTCTGCAACTCGGTGCTACAGGAGAATA |
| 27499_24 | 25 | [G/A] | CACGAGGCCTGAGCACGGAAATGCGTGCAAGTTCTC |
| 2750_29 | 30 | [G/A] | TGTGCTTTGTTTGCACATTTTTGCTGAGCGCACACT |
| 27501_20 | 21 | [C/T] | TACACAAGCCAAGCACGTCTCTGCCGTTGACTTTCT |
| 27503_11 | 12 | [C/A] | AAGCTTGTGATCGCAGAGTAGTGCGCATCCTGTGGG |
| 27507_3 | 4 | [G/A] | TTCGGGTGCAAGGCAAGAATATGCACACAAGTATTT |
| 2751_18 | 19 | [T/C] | ACGAATTTCCTTGCAGGATGATGCCAATAATGACCC |
| 27510_11 | 12 | [T/C] | ATGAGATGCAATGCAGCCGTGTGCTCCAAGTAGTCG |
| 27513_4 | 5 | [C/T] | CCCTCATTGATTGCAGGCTGTTGCTGAATAAGCGAA |
| 27515_25 | 26 | [C/T] | AACCGTGTTCCTGCACATGCTTGCTCAAGTGCACTT |
| 27517_24 | 25 | [G/A] | ATTCCTCAGTCTGCACAACTTTGCGTGACTACACTC |
| 27519_29 | 30 | [A/G] | AATTTTGCGGTGGCACGGTGGTGCTGCAGATAGTGC |
| 2753_29 | 30 | [G/A] | ATGGTTTCCATAGCAACGACTTGCACGATGGGCGTC |
| 27530_33 | 34 | [C/G] | TCAGGTAAAGGCGCACAAGGTTGCCAGATTGACCTC |
| 27531_19 | 20 | [A/T] | TTTGGAGTGCCAGCATTGCATTGCTATGTTCCTAAG |
| 27533_34 | 35 | [G/A] | TGGATATGGCAGGCACACACATGCACATACACACGC |
| 27535_4 | 5 | [A/T] | GCTCACTCTGGAGCAGAGTAATGCTCTGGATCCCCT |
| 27538_9 | 10 | [C/T] | TGGAGAACTCACGCAGGTCGATGCTCCAGGATGAGA |
| 27541_26 | 27 | [A/G] | CTTTATGCTCGAGCAGCCTTTTGCAGAAGGAAACTT |
| 27547_30 | 31 | [C/G] | TGTGGTCAGAATGCACAACATTGCAGCCACCTAGGA |
| 27554_9 | 10 | [G/C] | AAAGATCTCGGAGCACGAAGCTGCTTTGAATCAGCT |
| 27555_3 | 4 | [T/G] | TCATAGAGCAAGGCAGAACCCTGCTGACCAATCAGA |
| 27556_3 | 4 | [G/A] | TAGGAAGCAGCAGCATTGCAGTGCATTACAGATCAC |
| 27558_16 | 17 | [A/C] | CTAAACAGGTGAGCACACTGCTGCAGTGACCTTAAG |
| 27565_9 | 10 | [T/G] | TCCCTCAGATGAGCATCGCGATGCCATCTATTATTA |
| 27579_33 | 34 | [G/A] | TAACAGAAGACAGCATTGCCTTGCACAAACCAGGAA |
| 2758_3 | 4 | [G/A] | CTTGAAAAACATGCAGTCGAGTGCTTGTAGTTGCTC |
| 27583_15 | 16 | [C/T] | ATTGTGACTTGGGCACCAACTTGCCCCCTGTTCCCT |
| 27584_31 | 32 | [G/T] | ACTTCCAGAATGGCATTTACCTGCGAGTGGAGCTGA |
| 27586_5 | 6 | [C/A] | TGAAACAGTACAGCAAGGTCATGCAGCAGATGCAGT |
| 27595_9 | 10 | [G/A] | AGCAGTTAGGGAGCAGAATCATGCTGACGCTGCACA |
| 27596_11 | 12 | [A/G] | TGACTGATATCAGCATTAGGATGCCACATGACTGAC |
| 27600_9 | 10 | [T/A] | TAGTCATGGTTTGCACACTCCTGCCCTGGCACAATT |
| 27607_24 | 25 | [G/A] | TTTCGTGTCCCAGCAACATGTTGCGTTCTGTCAGTT |
| 27608_6 | 7 | [G/A] | TACCGAGAGGCTGCATTCGCATGCTTTCCGTCCACT |
| 27610_30 | 31 | [C/T] | TACTGCCTTTTGGCAAATCCATGCCACAGACTCTTT |
| 27617_33 | 34 | [A/T] | CAATGGAGTCACGCACAATTTTGCAAAGGGTTCAAT |
| 27626_9 | 10 | [A/G] | CAGAGAGCTAAAGCAGGGTATTGCACTCTTTCTGAT |
| 27631_27 | 28 | [G/T] | GTCCAAACGCACGCAGACTGTTGCACTGCTATTGTC |
| 27632_19 | 20 | [T/G] | GACGAGTCAGAAGCAGGAATCTGCCTCAGTGTATGT |
| 27640_31 | 32 | [A/G] | AAGGGGGAGAATGCAAATTTTTGCACTGAGCAATAG |
| 27644_2 | 3 | [T/C] | TCTCCAAACACAGCAAACCACTGCCTAGCACTAATA |
| 27649_18 | 19 | [G/A] | AAGCACCAATTAGCATAGGAATGCATTTGGCTCCTG |
| 27651_17 | 18 | [G/A] | AATGCTGATATGGCACAGGATTGCAACAGGATGTTT |
| 27653_33 | 34 | [G/A] | GTGTGGCAGAGGGCACACCTGTGCATTCCACACGTC |
| 27655_15 | 16 | [A/T] | GCCTGTTCACTTGCAACGACCTGCCTCGGATATACC |
| 27656_19 | 20 | [C/T] | GGATGTCCTCTGGCACAGACTTGCTAGGCCAGACAC |
| 27662_32 | 33 | [G/A] | TGTCAGAGTTCAGCAAACAGATGCAGCGCTGTGAGA |
| 27664_1 | 2 | [T/A] | CTCACCCTCACAGCAGGACGCTGCCACCACCATGAT |
| 27670_1 | 2 | [G/A] | AACGAGATCCTCGCAACCTTCTGCTGGCTTTCCAGA |
| 27672_3 | 4 | [G/A] | GCCGGCCGGCGTGCATTCGTTTGCAGCGGTGGAGGG |
| 27676_16 | 17 | [G/C] | ACAGCTATTTCAGCACGGACATGCTAGAACTGCCAC |
| 27681_31 | 32 | [C/G] | GGCATCTCATGAGCAGGTGAGTGCTGGCAAACAAAA |
| 27683_5 | 6 | [G/A] | GAGGTGAAGTGTGCAGCTGACTGCAGCCCACATTAC |
| 27685_7 | 8 | [C/T] | CCCTGCTCCACAGCAGCTCCGTGCACAAGCGTACAC |
| 27686_33 | 34 | [G/A] | CTTAGTCTGTAGGCAGGCTTGTGCCTTATTTCCGGA |
| 27688_19 | 20 | [A/G] | AACTCCAGTCCAGCATCCAGGTGCATCCTGGAACAA |
| 27691_34 | 35 | [A/G] | TCTGAAAGGGGGGCACAGGTGTGCAGGTGTGCATAA |
| 27705_1 | 2 | [C/T] | TCTGTCAGCACTGCAAGAAGCTGCTCAAGGGGCTGT |
| 27708_20 | 21 | [C/T] | AAGGCTACAGATGCAACACTCTGCCCAAAAGGGATT |
| 27712_32 | 33 | [G/A] | GTGCTGCACTATGCAGGAGCTTGCCCAATACCGTCC |
| 27719_7 | 8 | [A/C] | ACACCTTAGACAGCATGTTCATGCCCATGGCGTCGC |
| 27728_17 | 18 | [C/T] | GAGCTTATCTCTGCACACGCATGCACATAGATCTAC |
| 27729_17 | 18 | [C/T] | GTTCCCTTCCCTGCAATCGTGTGCTTTTTATGAGAG |
| 27731_9 | 10 | [G/A] | GGTGGTCCCGCGGCAAATGACTGCCCGAGCAAGGCT |
| 27736_1 | 2 | [C/T] | CCCTCTTGTGCTGCAGGTAATTGCTGTGGGACTCGA |
| 27742_10 | 11 | [C/T] | ACTTTTGTTTCAGCATGTCAATGCAGTCGTGCTGTG |
| 27745_11 | 12 | [C/T] | GCCATATCAAACGCATCGGTCTGCCTTATCGCTTTT |
| 27747_1 | 2 | [C/A] | CCGTCACGTCCAGCACCATCATGCTGCCGTCCGAGA |
| 27749_30 | 31 | [A/G] | ACACCTGCAGGCGCACAATATTGCCCTACTAGAGAC |
| 27753_32 | 33 | [T/C] | GATTGGCCACGAGCAAATGTGTGCAAACTAAATCAA |
| 27758_34 | 35 | [C/G] | TGCTGGCAGAAGGCAGAACCTTGCCACGATACGCCA |
| 27762_26 | 27 | [C/A] | GTTCTCCACACAGCAGATTAGTGCAGCTCATTCTTT |
| 27769_28 | 29 | [T/C] | TGTTCACTCAGAGCAGCAACATGCACAGTCATATTC |
| 27773_18 | 19 | [A/G] | ACACTACCTATGGCACCAACGTGCTGTCAAGATAAT |
| 27774_32 | 33 | [A/G] | CATGAAGATTCAGCAGATTCATGCCCTTGGGCGAGG |
| 27775_6 | 7 | [C/T] | ATGCTGCCCCATGCACCACCCTGCTGTCCTTCTGTC |
| 27779_3 | 4 | [T/A] | CATTACTGGAGTGCAGATGTTTGCATCCAGTTCAGA |
| 27781_17 | 18 | [G/A] | GTGCGACGCCTGGCACTGAAGTGCGAGGAGCGCTTT |
| 27782_32 | 33 | [G/A] | CCACAATCGCTTGCAACTACCTGCTTACGGTAGAGC |
| 27786_6 | 7 | [A/G] | TGGAGCAGCCGAGCAATCCAGTGCTTGGTAATCCAG |
| 27787_7 | 8 | [T/C] | ACACGCCTGCGGGCACACACATGCACACAGACACAC |
| 27792_10 | 11 | [G/T] | TTAAGGTTTGGAGCATGCCTGTGCAATGAACTTGTT |
| 27797_32 | 33 | [G/T] | GTGAGGCTTCAAGCATCAGCGTGCAAGCATTAGTCA |
| 27798_31 | 32 | [G/C] | GCGGCTTTCAGGGCACCAACCTGCTGCACCGCGTCA |
| 278_7 | 8 | [T/A] | CTCTGTATCACAGCAGCAGGTTGCCGACGAGAACAT |
| 2780_33 | 34 | [T/C] | CCCCTCAGGATGGCATGTCTATGCAGTATCGTTCAT |
| 27807_31 | 32 | [A/T] | CCACCCACAGTGGCAGTTACTTGCCCCTTTTACTTG |
| 27808_15 | 16 | [A/G] | TTCGGTAGCCAGGCAATTCTCTGCGCTCCATTCAAC |
| 27810_8 | 9 | [T/C] | CAGATGGCTACAGCACAACGCTGCCCTCTTGTGACT |
| 27830_32 | 33 | [C/T] | CGCAGATCCATCGCAGGGTACTGCATTCAGTGCAGT |
| 27839_4 | 5 | [C/T] | TGTACGAACCAGGCACGCATGTGCGTTTGAAGTGTG |
| 27841_9 | 10 | [T/C] | CGAAATCCACGTGCATTTTGCTGCCTGCAATACAAA |
| 27844_18 | 19 | [C/A] | AGGGAACGAGTGGCATGCCGGTGCTGATGGGTAATG |
| 27850_17 | 18 | [G/A] | GGACAAAGAGATGCAGTGGGTTGCGATGTTGCCTCA |
| 27868_25 | 26 | [T/C] | GAGGGAAAACGCGCACTAGGGTGCACAAGAGCAAAC |
| 27875_25 | 26 | [C/T] | GACCTGACGTGGGCACGGCCATGCCCGAGCTCATGC |
| 27880_30 | 31 | [G/A] | ATGACGAGTTTGGCACACGTCTGCCACGCCGTGAGC |
| 27882_18 | 19 | [A/G] | TATGGATTTGGTGCATTTAGGTGCTCAGATGACTTT |
| 27886_6 | 7 | [C/T] | ATCAACCGTGCTGCATCTAAATGCAGATTTCTGGAA |
| 27891_34 | 35 | [A/C] | ACCCACACAAGTGCACACACATGCATACATGCACAT |
| 27893_20 | 21 | [G/T] | GAGCTCAGCTGGGCAATAAGGTGCTGGTACTCCTGG |
| 279_19 | 20 | [C/A] | ATTAGAGCGATCGCAATCACATGCCGTGGGAGGCTA |
| 27900_15 | 16 | [C/T] | CGTGTCGGGCTGGCACGTGACTGCGGCACGTCTCTC |
| 27902_1 | 2 | [G/T] | GGATGTTTGCTGGCAGGCAGCTGCACGCACACAAGA |
| 27904_34 | 35 | [G/A] | AATTAAAACCCAGCATCATGATGCACACACAGGTGA |
| 27905_2 | 3 | [A/G] | AAAGGCAATGTTGCAAATGGCTGCCCTGCCCAGAAG |
| 27907_3 | 4 | [A/T] | TGAAGGATTTTGGCACAAGCTTGCAGCGTTGTTTTG |
| 27909_18 | 19 | [G/T] | CCAGATTTCCCAGCACTCGGCTGCCTCGGAGTCTGC |
| 2791_32 | 33 | [G/A] | GCCACTTGGGTGGCATGGTAGTGCCGAATAGTGACT |
| 27910_26 | 27 | [G/A] | GCACAGGAATCTGCACAGGTGTGCACGTGAACCACA |
| 27912_27 | 28 | [C/T] | TCCTCAAATGATGCAGCATCCTGCTTTCACAATGCA |
| 27923_8 | 9 | [G/T] | AGTGAGCAGTGAGCACTTGTCTGCAGCACTGTCCAG |
| 27930_27 | 28 | [A/G] | TACTCCACAAAGGCAAAACCCTGCCAAAAACCGAGG |
| 27931_31 | 32 | [T/C] | CTAAACCCAAATGCAAAACAGTGCAGGCACTTCTGG |
| 27932_7 | 8 | [G/A] | ACACACTGAGCTGCAGTTTCATGCAGCCAATCCTCC |
| 27935_28 | 29 | [G/A] | ATGTTCTTGGCTGCAGTAAAATGCAAATGGAGCAAT |
| 27938_29 | 30 | [G/A] | ATGGAGAAACCGGCAGCGTCGTGCGAACGGATAACA |
| 27941_19 | 20 | [G/A] | CAGCCTCTCTGTGCAGATTGCTGCTGATTGAGCAGG |
| 27946_33 | 34 | [A/G] | CCCTTGGGTAAGGCACATAACTGCTTTACCCGGAGC |
| 27950_26 | 27 | [C/A] | ACACTACCTGCGGCACCACCATGCCACCTCACCCAT |
| 27953_33 | 34 | [A/C] | ACAGACAGACTGGCAGCAAAGTGCACAAGGCCTATC |
| 27955_1 | 2 | [C/T] | GCGAACACTCACGCATCTTCGTGCCAGCTCCGTGTG |
| 27956_32 | 33 | [A/C] | CAAAGGTATTAGGCAGCAGTCTGCAACACTCGAGTG |
| 27960_34 | 35 | [G/A] | TTCTCTAGTATGGCAAAAGCATGCACGAGCAAATGG |
| 27967_7 | 8 | [T/C] | CTGTGTACGTGTGCAAATCTCTGCTTCTCCTGAGTC |
| 27971_10 | 11 | [T/G] | AATGCCCAGGTAGCAATTGTGTGCATTCTGCCATCT |
| 27977_18 | 19 | [C/T] | ACAAGCTTTTTTGCAGGACTTTGCACCTGTCCACAC |
| 27979_34 | 35 | [C/T] | GAGGGGGTTTCTGCACAGCACTGCACCGTCACCGCG |
| 27985_10 | 11 | [G/A] | CTGGCAGAGGGAGCAGGGCAGTGCAAATGTAAAACA |
| 27997_27 | 28 | [C/T] | CAGGCTCTAGTGGCAAACTAATGCACTCCTGTGACT |
| 27999_25 | 26 | [C/T] | GTCGAAAGATCCGCATACCTCTGCACGCTCCAGTCA |
| 28002_7 | 8 | [T/C] | TTCTGCATGCTCGCACTATAATGCCACGCTTACACC |
| 28010_17 | 18 | [C/T] | GGCAGGGTTTCAGCAAACGGATGCTAGCATGTGTCC |
| 28014_19 | 20 | [G/A] | TGATAATCACATGCATTCTGATGCGGTCTGTTCGCA |
| 28016_9 | 10 | [C/T] | GTGTGCGTTCGTGCATGCGTGTGCGAAAGAGTGTGT |
| 28017_6 | 7 | [G/A] | AAAGAGGATAGAGCAGATCAGTGCACAAAGGCCCTG |
| 28019_1 | 2 | [A/C] | AACAGAAAAACAGCAGAACCTTGCTAAAGCGAACTC |
| 28036_9 | 10 | [A/T] | CCCTGTGGGAGGGCATAAGACTGCTACAGCACTCAG |
| 28044_28 | 29 | [T/C] | TTAGCCTGATATGCAGCCAACTGCTATGTACTGATA |
| 28051_7 | 8 | [C/T] | GTGTGTGCGCGTGCATGTGCGTGCTGGTGTGCATGT |
| 28054_5 | 6 | [G/C] | GCTCAGACTGTTGCATTTTCATGCGTCATCATCTGC |
| 28057_24 | 25 | [T/C] | TGCTTCTTAGCTGCAGGCGTGTGCTTGTCGTCCTCA |
| 28059_2 | 3 | [C/T] | AACGCAGGAAGTGCAGATAGATGCAGATCTGTGAGC |
| 28063_11 | 12 | [G/A] | GGATAACCTCTGGCATTGCCCTGCATCTGTCCCAGG |
| 28064_32 | 33 | [C/G] | TAGCAGCACAAAGCAATGAGCTGCTTGCAGATCGGA |
| 28070_19 | 20 | [C/A] | TGACTCTCCCGAGCACGTTCCTGCAGCTGTTTAATC |
| 28074_30 | 31 | [G/A] | CAGACATAACGAGCAGAGAATTGCAGTGCAGATCGA |
| 28077_10 | 11 | [G/T] | ACACGGAATGGTGCAGATCTGTGCTAGTTTCTAGTT |
| 28080_32 | 33 | [C/T] | ATCTTTGCACCAGCACTCACCTGCATGATGGTCCAT |
| 28097_7 | 8 | [G/C] | TTAAAATGTAGCGCATGGTTTTGCCATTTGGATCAG |
| 28098_17 | 18 | [G/A] | TGTAAAGCAATTGCACCGGCATGCTTTTTGCTCATT |
| 28099_25 | 26 | [T/A] | CCTTGCTGTGAGGCAGAAGCGTGCCTCTGTCTGAAA |
| 2810_29 | 30 | [T/C] | AAATCTTTAGGAGCATTCTCATGCTGCCTTGGTGGC |
| 28102_27 | 28 | [A/T] | AACTTTCGCTTGGCACCGACGTGCCGGATAATAAAC |
| 28107_8 | 9 | [T/C] | ATCAGAATTCCAGCATGACACTGCCACCAACACACG |
| 28112_32 | 33 | [T/C] | CTGTTGAGAGGGGCATTGCAATGCATTAGCATTCAG |
| 28115_34 | 35 | [T/C] | AATCAGATCGAGGCAGAATTGTGCGATGACTTGTCA |
| 28118_27 | 28 | [T/G] | TACAGCATCACGGCATCTTAATGCATCTCAATGGAA |
| 2812_28 | 29 | [C/T] | GCGTCCGTCCTCGCACCTGTTTGCTGAGCCGTTTCT |
| 28127_34 | 35 | [A/G] | GTAGGAGGGAAAGCAAAATGATGCTAAGCACACAAG |
| 28136_4 | 5 | [T/G] | GTCATGTTGGCAGCAGCACAATGCATGAAGTCATAT |
| 28137_33 | 34 | [C/T] | AAAGATGTTAAAGCAGTCTGGTGCCTGGTTCTTCAA |
| 2814_2 | 3 | [C/T] | ACCCAGACACACGCATAGACATGCATGCGGGTCTGT |
| 28141_5 | 6 | [G/A] | CTAACGGCATTGGCAACAGTGTGCTGCGTCACTTTG |
| 28159_16 | 17 | [C/G] | CTGATGCGAGCTGCAACAGGATGCCAATGAAGGGAG |
| 28164_30 | 31 | [A/G] | GGGCTGGAAGGTGCACTTGTGTGCTGCACAACTTAA |
| 28165_18 | 19 | [A/T] | CAAATCACACATGCACCCACATGCCACACTATACCA |
| 28171_1 | 2 | [C/T] | GCCAGTGGCTCTGCATGTTCCTGCACAGGACTTGAA |
| 28175_3 | 4 | [G/A] | TCTAGGGTTAGTGCAAATGTTTGCCAAGCCTTTTGC |
| 28176_20 | 21 | [A/G] | TAAATGCGTCATGCAGCACCATGCAGACACTGTATA |
| 28186_34 | 35 | [G/A] | AACACACCAAGGGCAATTTCCTGCCCAAAATCATGA |
| 28189_3 | 4 | [A/T] | TTTAAGGCAAATGCAATACGGTGCAATCTGTCCGTA |
| 28190_16 | 17 | [G/A] | TGGAGGCGGCGCGCACGGAGCTGCACTCGGCCCTGC |
| 28191_2 | 3 | [T/G] | TATTTGACTCCTGCACCTTACTGCAATTTCAGCACC |
| 28192_7 | 8 | [G/A] | TGAGGTAACCTTGCACGGTCATGCACTAATCGATAT |
| 28196_4 | 5 | [T/C] | TGCCTGCAAACAGCATCAGAGTGCCGTCAGCGAAGG |
| 28197_27 | 28 | [A/G] | CTCAAGCTTTTGGCAAGAAATTGCACCAGTGTTGTG |
| 28201_31 | 32 | [G/A] | GATGCTCAGCTGGCACTGGTTTGCTTAGTTCGTCTC |
| 28202_28 | 29 | [G/C] | ACAAAGCTAAAGGCAGAAAGATGCTCAACCATGCAG |
| 28210_33 | 34 | [T/C] | CTAAAGACTAATGCAATCGGGTGCTGTCCAGAATAC |
| 28211_9 | 10 | [A/G] | CTTTGGTCCATTGCACTTCCCTGCAGCTCTAGCTTG |
| 28214_31 | 32 | [G/A] | GCAGATTGATTGGCAGCCAGATGCGGAGTGAGCAGA |
| 28215_26 | 27 | [C/A] | ATTGATTCAGAGGCAGTAATCTGCACCGCTAATGTT |
| 28222_11 | 12 | [A/G] | AGAATGCATGGAGCACACAGGTGCACACACACACAC |
| 28223_28 | 29 | [G/A] | GTGTCTTCAGCTGCAGGTTTTTGCCTACGATTCTTT |
| 28225_18 | 19 | [T/C] | AGCGGTGGGAAAGCACCGTGTTGCTATATTTGTGTG |
| 28229_10 | 11 | [C/G] | CCTAGTCACACGGCACTCGCGTGCGAATGTGTGAAA |
| 28230_20 | 21 | [A/T] | AGAAAGCGTATGGCACACACATGCTGAGCTTGAAAT |
| 28237_9 | 10 | [T/A] | CATGCACTTTATGCACTGTGGTGCAGGTGGGTAATT |
| 28243_15 | 16 | [G/A] | GACTGAGGAAAAGCAGTAACCTGCGACAGGGCAGCC |
| 28249_6 | 7 | [A/G] | ATTGGGAGATCTGCACTTCAGTGCCGAGTAGAAAGA |
| 28251_34 | 35 | [G/A] | CCATGGCCTCCAGCAAGCTACTGCTAGCTGCCAAGT |
| 28252_32 | 33 | [A/G] | AAACAGACACGGGCAAATTTATGCCACTGGGGAAAA |
| 28260_29 | 30 | [C/A] | CGGGTGGCTTCTGCATGTCGGTGCTCTTTCACTCTC |
| 28264_34 | 35 | [T/A] | TCAGATACATCAGCATGCACGTGCTTTTTGGCCTTA |
| 28277_1 | 2 | [G/A] | TGAGTGCAGCATGCACTGACCTGCCTGTGAGGCCAA |
| 28279_10 | 11 | [G/A] | TGACGCACTCGGGCATTGGGATGCATCAGCAGGTCT |
| 28281_32 | 33 | [G/A] | CACCGGGACACTGCAAACTGCTGCCTACGACAGCAC |
| 28288_1 | 2 | [T/C] | TTCTGGTTTGAGGCATAACAGTGCTGTTTTGTTCTG |
| 2829_33 | 34 | [G/T] | GGTTGTGTTAAAGCAAAAATGTGCCAGGACCAGTGT |
| 28290_27 | 28 | [G/A] | GTATGCAGAGGTGCATGTATCTGCTGGGCTCAATGT |
| 28296_25 | 26 | [G/A] | CCATACCTGTCTGCAGATGCATGCTGAGGGTGTGTG |
| 28298_7 | 8 | [T/C] | TCTGTCCTGCTTGCAATTAAGTGCCCAGCGCGACAG |
| 283_11 | 12 | [C/A] | TGACATGCAAGCGCACGCTGTTGCACAGAAGCATTA |
| 28302_11 | 12 | [T/A] | GATATATTGGCTGCATATGTTTGCAAACAGTGGCAT |
| 28304_30 | 31 | [T/C] | TGGCAACAAATTGCAGTTGGCTGCACAGCCTTTGGC |
| 28306_24 | 25 | [A/G] | CAAAGCAAGCAAGCACGTGGGTGCACACTTCTGTAA |
| 28311_5 | 6 | [C/G] | ATTAGCGTTGGGGCAGGGTTGTGCTATCTTCCAGGC |
| 28312_6 | 7 | [C/T] | ATGACTCACCATGCATTTTGATGCCCAGTTTGTAGG |
| 28320_29 | 30 | [C/T] | TGAATTTCGGGAGCAGTTTCGTGCAGAGCCCATCTT |
| 28321_32 | 33 | [C/T] | CCAGGTGACCCGGCAGCACCCTGCCTGTTTTTCTTT |
| 28338_4 | 5 | [T/C] | CTGGTCGTTCCAGCAAAGGTATGCGAGCGCTGTGAC |
| 28345_19 | 20 | [A/G] | TACAGGATAGGCGCAGGATAGTGCTCGCTTCCAGGG |
| 28346_11 | 12 | [G/A] | GACTGTCGAACGGCATGACAGTGCAGCAGGTAGCAT |
| 28347_1 | 2 | [G/A] | CGCATTGACAGCGCACGGACGTGCCGTCATTAAGCT |
| 28349_5 | 6 | [A/T] | ATGTTAGCAGGTGCATTACTGTGCCACCCATTATCA |
| 28350_3 | 4 | [A/G] | GGAAAGAGCGACGCAGCATGCTGCAGCTCTCTGTTC |
| 28353_4 | 5 | [G/A] | AGACGAGCCTATGCAGTGACATGCCATCCATGCACA |
| 28354_16 | 17 | [G/A] | TTGGTCCAGAAAGCACGCTTCTGCACATTCCTAATA |
| 28359_6 | 7 | [T/C] | CCAGCTTACAGGGCATAAGGTTGCCGTAAAGATCCT |
| 28363_28 | 29 | [C/G] | ATTAAACACAATGCACGGGCTTGCGTTCCCCGGCTG |
| 28366_2 | 3 | [A/G] | TCAGCTAATATAGCAATGCGGTGCGTAAAAACGTGC |
| 28370_6 | 7 | [G/T] | AACCATGGTGAGGCAACCAAGTGCTCATCTAAAAAT |
| 28376_7 | 8 | [T/C] | AAACGTCTCGAGGCATCACCTTGCTGCAGTAGTACT |
| 28379_24 | 25 | [A/G] | GCACTGGAACCTGCAACCTGATGCAACTGGTTGTGT |
| 28381_1 | 2 | [T/A] | GTACACAGGTGTGCACACAGCTGCTAACAAACGTCA |
| 28386_25 | 26 | [G/C] | ATAGACAGCAAAGCACATGAATGCGGCCCCTGCTGC |
| 28389_2 | 3 | [T/G] | GCTTATGGTTGGGCACAGATATGCACACATACACAC |
| 2839_11 | 12 | [T/C] | TGTAAAGTAGCTGCAGCGAGCTGCTGATGTTAGATT |
| 28392_34 | 35 | [C/A] | CGGTCAGCTAGGGCACTAGATTGCGTGCAGAAGCCA |
| 28394_26 | 27 | [C/T] | ATGGCATCCCCAGCATACCACTGCACCGTCACATTC |
| 28395_27 | 28 | [T/G] | ACTGCTCCTCCTGCACACATCTGCCCCTGCCTTGCC |
| 28398_9 | 10 | [C/A] | TTGTTGGACCATGCATGTCCATGCTTTGAAAACGAT |
| 28399_28 | 29 | [T/C] | TGTCTGGATGATGCATGACTGTGCTAGCTCATGCCT |
| 28407_30 | 31 | [A/T] | TCCTTGGGCAAGGCACCTAACTGCAATTCCATCAGA |
| 28409_26 | 27 | [G/A] | GTGAATAAATCTGCATGGCGGTGCTTGTGCACCCAC |
| 28411_26 | 27 | [G/T] | CTCTTTGGATGTGCAACAGCATGCTCGGTGTAGCTT |
| 28418_4 | 5 | [C/T] | GCTGCATGATTGGCAACCTTGTGCATTGTCAGAGCA |
| 2842_25 | 26 | [G/A] | GTCTGGCCGCTGGCAAGGTTTTGCCGGAACAGAAAC |
| 28422_10 | 11 | [G/A] | TGTGTGTTAGGGGCAGAGGCATGCAAGTGTATACAG |
| 28427_33 | 34 | [A/C] | GAGAGTCACTCGGCATATTTCTGCTGAACAGTGAGG |
| 28428_15 | 16 | [T/A] | AGGCCCTACTGCGCATGCGCGTGCCGGAAACACGTG |
| 28438_27 | 28 | [G/A] | TTTATGCTGCGGGCAAACATTTGCACTGAATCGTAT |
| 2844_2 | 3 | [C/T] | ATCAGTGACTTTGCATGCCCGTGCTGCTGGATCACA |
| 28450_1 | 2 | [A/T] | GAGGTGGGCGGAGCAGGGACTTGCCCTGAAAGTCGC |
| 28451_31 | 32 | [G/A] | ATATATCACCCGGCACCGGCTTGCTCATTCTGACTA |
| 28453_26 | 27 | [T/C] | AAAAATGAGGTGGCACAGTGTTGCTGTAGGTAGTGC |
| 28457_16 | 17 | [T/A] | TTGGCTTTTGAAGCACTACACTGCAATGGCAGGGCC |
| 28459_27 | 28 | [G/A] | GCCTCCGAAATAGCAACAGTGTGCGCTGGGCTATTT |
| 28460_17 | 18 | [G/A] | TACTGTGTGAAGGCAAGGGGTTGCCAAACAGGATTT |
| 28464_6 | 7 | [C/T] | TGCTCTTTTCCAGCATCATGATGCAATCACTGGCAC |
| 28466_18 | 19 | [G/A] | GTGAACAGTGGAGCAGGTGAATGCCGGGTGTCCCAG |
| 28469_27 | 28 | [C/G] | GTAACGCGTACAGCACACACGTGCGCTCGTGTAAAT |
| 2847_25 | 26 | [G/C] | GATGTTTGCTGTGCAAGTTGTTGCCGTGTTTATGAA |
| 28474_3 | 4 | [T/A] | ATGTGTGACGGAGCAGTTTAATGCCTTGGTTGTCGT |
| 28486_29 | 30 | [A/G] | TGTAAGACCTGCGCAGACAGGTGCTAAACACAGACG |
| 28490_27 | 28 | [C/A] | TTCCAAAAAAAGGCACGTTCTTGCGTTCGTAGATAT |
| 28497_3 | 4 | [T/C] | TTTTACTGCACAGCAGGGCAATGCTTAACTTTCACT |
| 28498_4 | 5 | [G/C] | TGCTGGCTCTGCGCAGACGTCTGCCTCCTGTCCTGT |
| 28505_6 | 7 | [G/A] | TGTCCAGGACCTGCAGAAGCCTGCTGACCTTCTGTT |
| 28506_10 | 11 | [A/G] | GTTTACGGCGAAGCATGGTTGTGCTTGTGGAGGTTG |
| 28508_26 | 27 | [C/T] | TAGACAACATATGCACTGTGCTGCTGCCCATTATTC |
| 28517_27 | 28 | [C/T] | TGTGGTTTTAATGCACTCCACTGCTGCCATTTGACT |
| 28519_3 | 4 | [A/G] | GCAACATGTTGAGCACATGGTTGCAGATTGGTCCAA |
| 28521_33 | 34 | [G/A] | TGCAATCCCTGTGCAACAGAATGCAAGGAATGTGAA |
| 28525_4 | 5 | [G/A] | TCTTGAAGGTTGGCATGGTGGTGCCAGAGGTAGTGA |
| 28526_10 | 11 | [C/T] | CTTGATGCAACGGCAAAAACCTGCACTAATGAGGCG |
| 28529_18 | 19 | [C/A] | ACACTACCTGTGGCACAACTTTGCTGCCTGCGGTAT |
| 28533_18 | 19 | [C/T] | TGCTGCTGATCTGCACAGCGCTGCCACTTCTCCCAC |
| 28539_4 | 5 | [C/G] | TTCTCTATAATTGCAGGAAGTTGCTGGTGAGCCTCT |
| 28541_4 | 5 | [C/T] | TAAACGAACCAGGCACCCCAATGCCACTGGTGTTTA |
| 28549_33 | 34 | [T/G] | CTGCTGCTTTCTGCAGTTTTCTGCTGGTGGTGTTAA |
| 2855_8 | 9 | [A/G] | AGCAAGGGACGTGCAAAATTTTGCACACCCTTCAGA |
| 28550_11 | 12 | [T/C] | ACACACACACATGCAGGCAAGTGCACACAGCCTCTG |
| 28554_24 | 25 | [G/C] | TATTTCCCAGTGGCAAATCCCTGCGTGATCAACCGC |
| 28555_10 | 11 | [G/A] | CAGCTGTGTGGAGCAGAATTCTGCAGAGAAAAAGAT |
| 28560_3 | 4 | [C/T] | GCTCTGTCTCTGGCATGTTCCTGCTGTGCACTTGAA |
| 28565_1 | 2 | [C/T] | CCAGATGTTTGCGCATACACGTGCCTGTTCACCTCC |
| 28568_32 | 33 | [C/G] | AAGTACGGGTGTGCAAACTTTTGCATTCATCTCTAT |
| 28574_24 | 25 | [G/A] | GACTCACAGGTAGCACTTTGCTGCGCCTACAGGTGT |
| 28578_28 | 29 | [A/T] | TCCATCACAGGAGCAAGCCGGTGCAACAAATAGAGC |
| 28586_2 | 3 | [C/T] | TTCCGAAACTTTGCAAAAGTATGCCACGCAATATCA |
| 28591_11 | 12 | [A/G] | ACACTACTTGTAGCACCACCGTGCCACCCTATTTAT |
| 28596_33 | 34 | [G/A] | AACTTGTGGCTGGCACTGAGGTGCAACAGATACGAT |
| 28601_33 | 34 | [T/A] | AATAACGTGGCAGCAAAGCAATGCAACGTGTTATAT |
| 28603_1 | 2 | [C/A] | ACAAGACACACAGCAACACAGTGCGAGTGAGAAGCG |
| 28604_33 | 34 | [C/T] | TGATCTGTATGAGCACTTTGATGCCCACTAGCTCCA |
| 28605_34 | 35 | [C/T] | ATTGATTTGATGGCACAGCCTTGCTGGCACAGCTCC |
| 28606_10 | 11 | [G/A] | GAATTTAGCAGAGCAGCTACTTGCCTATGTGCCACT |
| 2861_3 | 4 | [C/T] | AATCGCACCACTGCATTCTAGTGCAGTGCCAGAGTT |
| 28611_34 | 35 | [G/A] | GACCCCTGGCCAGCAACAAAATGCCCCACCAACAGA |
| 28612_26 | 27 | [T/C] | TAACTTCAACATGCATGCACATGCACCAGAGCTCCT |
| 28619_6 | 7 | [C/A] | ACTCTCCCTAATGCACAGCAGTGCTCAGGGGTCAAG |
| 2862_33 | 34 | [T/A] | GCTGATATGTCTGCAGGCACTTGCGAGATGTTTTAC |
| 28621_29 | 30 | [A/T] | GCTCCCAAGATAGCAGGCTGTTGCTACACAACAACT |
| 28623_17 | 18 | [T/A] | ATCAGACCATTGGCAGTTAGCTGCTATTTGCGAGAG |
| 28625_5 | 6 | [T/A] | TTGGGTAAACAGGCAGCTTATTGCTACATTCCTGAC |
| 28626_11 | 12 | [G/A] | GCCATTGACACGGCATCGTCGTGCAGGGGCCCCGGT |
| 28633_7 | 8 | [A/C] | TGAAGAAAGCGGGCAGATAGCTGCTGTTGCCAGGAG |
| 28636_7 | 8 | [T/C] | AATCCATTAGGAGCACAATGGTGCTAAACAAGGCTA |
| 28640_30 | 31 | [A/G] | ATCGGCCATCAGGCATGACATTGCTTAGACACAGAC |
| 28644_20 | 21 | [C/T] | AAGTGGGTTAAAGCAGCCATCTGCAACTTGTCTTTG |
| 28646_25 | 26 | [G/A] | TTACTGCGGTGAGCAATTTCCTGCCGTTTCCTGCTT |
| 28647_31 | 32 | [G/A] | GAAATGGATGACGCATTTTATTGCCGCCTATGAAGG |
| 28651_17 | 18 | [C/T] | TGCTTAGACCCTGCACCCATGTGCTGAAGTAGTTAA |
| 28653_34 | 35 | [G/A] | AGTGTGTTCAGTGCACACCATTGCCAAGCTGTCTGA |
| 28656_2 | 3 | [G/A] | TGGAAGTGATGAGCAAGTGGCTGCCCCGAGGCTAAC |
| 28660_7 | 8 | [A/G] | TCTGCAGAAGGGGCAGGCAGATGCTGACCTCATGTA |
| 28662_17 | 18 | [C/A] | TTTATTAGAAAAGCAGCCTTGTGCGCCATCTAACGG |
| 28664_1 | 2 | [G/C] | CGGAGTGTAAATGCAGTGTTTTGCTGTGGAGTTTAA |
| 28673_15 | 16 | [A/C] | GTCATGTGCAAAGCAACCCGGTGCCAAAATCCTCAG |
| 28678_6 | 7 | [C/T] | CCAACTCGCTGGGCATTGCCTTGCAGATCCTCCTTC |
| 28680_30 | 31 | [G/A] | ACATGCAAAACGGCACTCAAGTGCGGAGAGGAAAGA |
| 28686_5 | 6 | [T/C] | GTATATAACTATGCACCGATCTGCCGTGGCATTAGA |
| 28688_19 | 20 | [C/T] | CAATGAATACTGGCAGCACCTTGCAAAGCATGCTAT |
| 28691_33 | 34 | [G/A] | TGATGGTGGCTTGCATGCAGCTGCTCAGCCATGGAA |
| 28692_4 | 5 | [T/C] | TTCCTACAGTCTGCAGCAGGCTGCACGAGATGCAAA |
| 28694_34 | 35 | [G/A] | GGCCATATTAATGCAGTCACATGCAGGGCGGATCGC |
| 28696_18 | 19 | [G/A] | AGCACTTTGGGGGCAGTTGTGTGCTGGTGGTTAGGG |
| 28697_1 | 2 | [G/A] | GGCGGGGCTGCAGCAGTAGAGTGCCTGTCTGGGCCT |
| 287_18 | 19 | [C/T] | AAGCACTAGCTAGCAAAACCGTGCACCTTCTAATTT |
| 28701_31 | 32 | [T/C] | TGATGAGCTGTTGCAGCATTTTGCAGTCTCTTAAGC |
| 28706_32 | 33 | [G/A] | TCAACATCCTTTGCATCGGCATGCATCCAAATGTAT |
| 28709_10 | 11 | [T/C] | CGATGTCCGCTGGCAGGGCCGTGCGACAGTCTGAGA |
| 28721_4 | 5 | [A/G] | GGTTATGTGACTGCAGGAGCTTGCAGTGATCAAGAA |
| 28722_10 | 11 | [G/A] | GGGTGGTGTGGTGCAACCACGTGCAAAGTGGACTCA |
| 28729_20 | 21 | [C/T] | TAGTTGGAATCTGCAACCTTCTGCAGCTCGACTCCG |
| 28731_17 | 18 | [G/A] | AACTGGCCAACTGCAGAGAAATGCACAGAAATTACA |
| 28732_2 | 3 | [C/T] | CTCGAGTATGTAGCAGGTTACTGCCAGTGGCACTGA |
| 28736_30 | 31 | [C/G] | ATGGCTGTGTTCGCAAAGGAATGCCCACTACACACA |
| 28739_18 | 19 | [A/C] | TACAGCCATCCAGCATAGAGATGCTGCAGGAGAGAT |
| 28743_3 | 4 | [A/T] | CACAAGGTGGGAGCAATTGGGTGCATATGTGGCACC |
| 28749_34 | 35 | [T/C] | TTGTGTACAGGGGCATCGTCATGCAGGAACAGGTTG |
| 2875_17 | 18 | [A/G] | AGTGGAATGTCAGCAGCAACATGCTCAGGCTCCAAC |
| 28757_28 | 29 | [G/A] | TTGGGACGATCAGCACCATGGTGCGGACGGAGGGCC |
| 28765_15 | 16 | [T/C] | ACGGAGTGACGTGCATGTGCATGCACATTTCTGTTT |
| 28766_9 | 10 | [G/A] | CCGCATGTCAGAGCACATTCATGCCCACACACAGAC |
| 28774_29 | 30 | [A/G] | AAAAATCACAGTGCATTGATGTGCAGGTCAGTTCTG |
| 28779_31 | 32 | [G/A] | AATAGGTCCCAAGCAGCCGCCTGCTGGAGCAGAGCA |
| 28790_7 | 8 | [G/A] | GGTAGTAGGGCTGCAGGAAGTTGCAAGGCGTGACAG |
| 28792_31 | 32 | [C/T] | CCGGGTAGATGTGCAGTGTTGTGCCCACCACCAACA |
| 28794_5 | 6 | [C/A] | TCCCACACCGACGCAAGATTTTGCACTCGTAGTCCT |
| 28797_16 | 17 | [G/A] | ACACAAGCTGTTGCAGGTAACTGCTTCTGCTCTAAA |
| 28800_8 | 9 | [C/A] | TATACACACCAAGCAGACTCATGCTTAAGGTGGTCA |
| 28802_25 | 26 | [G/A] | TGTAGGGAAAATGCAGCCCTATGCGGAAGAACCTAT |
| 28804_17 | 18 | [C/G] | GATGTTGTGTTAGCAGGCTGCTGCAGGTTTACGCGG |
| 28806_18 | 19 | [A/G] | TTCAAAGCTTACGCAACCATCTGCAAGTGTAGCAGA |
| 28807_16 | 17 | [C/A] | GAGTGTGTCGCAGCACCGATTTGCTCTCAGCCCCTC |
| 28809_3 | 4 | [T/C] | ACATTACATGTGGCACCACCATGCTGCCTATTAAGT |
| 28812_3 | 4 | [T/A] | CTTTGGCAGAAAGCATTTTCATGCAGGCCCAATCAA |
| 28814_8 | 9 | [A/G] | ACTCCTGTAGAGGCAACAGATTGCACTCATTCCTCA |
| 28815_1 | 2 | [G/A] | CGTGTCCCCACAGCATGACACTGCCACCACCATGCT |
| 28816_4 | 5 | [G/A] | AGCGGAAACGAAGCAAGGTAATGCAGACAGAATATG |
| 28820_2 | 3 | [T/C] | TCTGTGATGCTCGCATGTGCGTGCGAATGTGAAATT |
| 28829_10 | 11 | [A/T] | TTCGTAATACATGCAAAATTGTGCGATGGATGCAGA |
| 28831_28 | 29 | [G/A] | TAGGCTCAGCAAGCATGATCATGCTCATGTCTAAAT |
| 28839_32 | 33 | [G/A] | GTGCTGCGGGGGGCATCTGCCTGCTCGGGGGCGAAA |
| 28848_19 | 20 | [C/T] | GCTTTAATTCACGCAGCAACCTGCGCTCACACATGC |
| 28851_25 | 26 | [C/T] | CTGTAGCAGTGGGCAGAAAGCTGCTCCTCTTATCAC |
| 28853_31 | 32 | [G/A] | AGGGCAGTCGCAGCAGCAGCTTGCCGAAGCGGCTCG |
| 28855_34 | 35 | [T/C] | TAGTTCAAACCAGCACATTACTGCCTTTATGGCCTG |
| 28857_9 | 10 | [G/A] | GGTGTTAATGCCGCACAATGCTGCTTAAACAGATCT |
| 28858_32 | 33 | [G/A] | TTACATGTGAGTGCACGAACATGCACGTGCACGTCT |
| 2886_31 | 32 | [C/T] | CCCACACTCACTGCACACATTTGCATCTTTTCTAAG |
| 28875_34 | 35 | [T/A] | AACAGCACTGCAGCAGATCGGTGCATCATTATAATG |
| 28876_4 | 5 | [C/T] | TCGGCGAGCACAGCAAGGCACTGCACACAGAGCAGA |
| 28879_4 | 5 | [G/T] | AGAGGTATGACAGCAGAAGAATGCGTTCGCGTGCAT |
| 28886_3 | 4 | [C/T] | CATCACTGCAGTGCATGGCTGTGCTGCCCTTTTGTC |
| 28889_28 | 29 | [A/C] | AGCAGAACTACAGCACCAAGTTGCTGACAGATTTTT |
| 28898_16 | 17 | [C/T] | CCTCCTTCCCCAGCATCCCTCTGCCGGGTCAGGCAA |
| 2890_9 | 10 | [C/T] | GAGGTCGTACGGGCAAAGAGCTGCATAAAAGTCATG |
| 28910_1 | 2 | [G/T] | CGATGTCCTGTCGCATTGGGCTGCAGAGAATCAGTG |
| 28911_25 | 26 | [G/T] | CCTGGTTTGGCTGCATGTTGGTGCTGCAGGTAGTGT |
| 28915_8 | 9 | [T/C] | TGTTAGCGTGGCGCAGCGGGATGCTAATCATCACTG |
| 28918_30 | 31 | [A/G] | TCCCACATGATGGCAGTATGATGCAAGCCCAACTTC |
| 28919_34 | 35 | [C/T] | TAGATGCAGAATGCACATCGGTGCTGTATCAGCTCG |
| 28923_18 | 19 | [A/G] | AAGGAACCACTGGCACACATCTGCATCCAGCTGTAG |
| 2893_27 | 28 | [T/C] | TAAATAAGGACGGCATAGTGGTGCTGTTGGTACATG |
| 28931_31 | 32 | [C/T] | CTCTGCGCCCTGGCACGCTCCTGCGCACACACGCGC |
| 28939_7 | 8 | [T/C] | ACATTTTCAGCCGCAGTCCCCTGCCCAACTCATAAG |
| 28953_19 | 20 | [G/A] | TGATGCTTTTGTGCACAGCGATGCTTTTTCAGAAAA |
| 28957_31 | 32 | [A/T] | TACCTGCCTAAAGCAGTCGAGTGCAGTTAATAAGAG |
| 28964_28 | 29 | [G/T] | GCCACTGGCAATGCAATTATTTGCTGTGGCCCAGTC |
| 28968_19 | 20 | [C/A] | GAACGTAAGTGTGCACACACATGCTGCTGTCTATTT |
| 28969_29 | 30 | [A/G] | TAAAGCAGCCGAGCAGTACACTGCTGCAAACCCATA |
| 28972_1 | 2 | [C/T] | GCTTGAAGGGCAGCAAGAAGCTGCTTTTCATCGACT |
| 28981_32 | 33 | [C/T] | GTCCCTGCTTTGGCAGCACACTGCCGCTTGTTCGGG |
| 28988_25 | 26 | [A/G] | ACAAGTTGTTGGGCAGCCCTGTGCCATAGAGAAATT |
| 28992_18 | 19 | [G/T] | GTATTCAGAGAAGCACTCGCTTGCTCTGTACCACTC |
| 28993_1 | 2 | [A/G] | AACAACAAGTGAGCAGTCACCTGCTTTCTCTGTGAA |
| 28996_6 | 7 | [G/A] | AGAAGTGAACTGGCACAACACTGCGTTCAGGCTTAC |
| 28997_33 | 34 | [C/A] | CTGAGGGTATTTGCATGATCATGCAGCTGCACACAG |
| 28998_4 | 5 | [T/C] | AATGTGAGTCACGCAGGAGGCTGCAGGATAAAGGAA |
| 29007_2 | 3 | [A/C] | TGAGATGAACATGCATGAGCCTGCTTTGGCTCTTAT |
| 29010_15 | 16 | [G/A] | GAGGGGATAACAGCAGCCTCCTGCAGATCTATCACT |
| 29011_11 | 12 | [T/C] | GAGCCTCATATTGCACCAGAGTGCACTCAGTCCCAA |
| 29014_29 | 30 | [G/T] | CACAGCCAGGTGGCACGGCTATGCTGCAGGCATGGG |
| 29017_19 | 20 | [A/C] | GAGGTCAAGGTCGCATTATACTGCAGAGGTGATTGT |
| 29020_34 | 35 | [T/A] | CTGCATGCGTTGGCAATATCATGCAGCATTTCTTTT |
| 29021_33 | 34 | [C/T] | GTTTCTAGCTTTGCAGTGGTTTGCCTCATCTTTCAT |
| 29023_24 | 25 | [G/T] | GAGAGCAGCATGGCAGAGCCGTGCGTCAGCATCTAG |
| 29027_1 | 2 | [G/A] | TGAGTCTGCAGGGCAAACACATGCCAATGTCATATT |
| 29028_11 | 12 | [A/G] | AGCGGGATCTCAGCATTAGGCTGCCGCGATGACGTT |
| 2904_10 | 11 | [G/A] | TGGTTACAGGGTGCACATTGTTGCTACCCGTTTCAG |
| 29047_34 | 35 | [A/T] | TTATTAAACAGAGCAAGCATCTGCCAGGGAAGTTAG |
| 29048_6 | 7 | [G/T] | GAAGAAGACGGTGCACACTTGTGCTGCTTTTCATTT |
| 29052_11 | 12 | [C/G] | CTTTTCCTTTTCGCACCCTTTTGCACCAGGAACAAG |
| 29053_4 | 5 | [A/G] | CAGTATGTATGCGCAGAATTTTGCAGCAAACACTGC |
| 29058_20 | 21 | [C/T] | CAGAAATACACAGCACCGTTCTGCTCCGAACCCCCT |
| 2906_9 | 10 | [G/A] | ATAACTCTGGAGGCATGTGTTTGCTGCACATCTCCA |
| 29062_4 | 5 | [C/T] | ATATCTTTCACTGCAGGGCATTGCTCAGGAGATCCT |
| 29064_8 | 9 | [C/T] | CGACTCTTCACCGCACAACCATGCAAGTGGAGAAAC |
| 29066_8 | 9 | [G/A] | GAATGACTGGAGGCAAAGAGCTGCTCTTCTCATTGT |
| 29068_2 | 3 | [C/G] | GCCTTTCAGGACGCACAAGTCTGCCTGTTCGTCTTC |
| 29069_34 | 35 | [G/T] | TAGATGTTGGTGGCATGGTGGTGCTGCAGGTAGTTT |
| 29070_33 | 34 | [T/G] | GCAGACTATGCGGCATTGTCATGCTCTCTAGTCTGT |
| 29072_11 | 12 | [C/T] | TAACTGGCTTACGCACACTGGTGCTCCATTACACTA |
| 29074_31 | 32 | [T/C] | GCCGGCACTGGTGCACTTTGTTGCCCCTGTGTGTTT |
| 29077_30 | 31 | [T/A] | ATACGTACGCCTGCAGCCCGGTGCTTTTATTATAAA |
| 29080_28 | 29 | [C/T] | GCAGTAACTGGGGCACCACTGTGCCACCCATGTCTT |
| 29087_25 | 26 | [C/T] | GAGTCCTCCAGAGCAGAGGGCTGCTCGTATTAATGA |
| 29091_2 | 3 | [C/A] | CCCGGCAGCCTGGCATCAGTGTGCCTGGAGCTCTAC |
| 29092_27 | 28 | [C/T] | AATACTTTTCCTGCACTTTGCTGCACTCGTAGCGTT |
| 29093_16 | 17 | [C/T] | ACACTGCCTGTGGCACCACCATGCCACCCCAACATG |
| 29097_33 | 34 | [C/T] | CTGACTGTTTTGGCAGCTGAGTGCCGAGCTCTCCGT |
| 29098_26 | 27 | [A/G] | AAGTCTGCAATTGCAGGGAAATGCAGAAAAGGCTTT |
| 29100_18 | 19 | [G/T] | ACTGCTGGTTCTGCATGAGCCTGCATTCATTCCATA |
| 29101_9 | 10 | [G/A] | CAGGATAAGGTGGCACGTCGGTGCCAAAGGTAGTGT |
| 29103_9 | 10 | [C/T] | AAACAAACGCGTGCAACCACATGCTTTGTTCATATA |
| 29107_24 | 25 | [G/A] | GTATGCAAAACTGCAGAGAGTTGCGATTTGGTGGAA |
| 29117_20 | 21 | [C/T] | GCCAGATCTAATGCAGGGTGCTGCTGGAGCAGACAG |
| 29121_27 | 28 | [T/C] | TGTAATATCCACGCAGACGTCTGCATCTTTACTGTA |
| 29127_2 | 3 | [A/C] | TGAAGCTACAAGGCAAACTGCTGCTTTCAGGGTAAA |
| 29131_5 | 6 | [A/G] | CTCACACAGGGCGCAGTGCTGTGCAGAAGCATGAGA |
| 29132_11 | 12 | [G/C] | ATTGATCAGATGGCATGGTGGTGCCACAGGCAGTGT |
| 29137_7 | 8 | [T/C] | AACCAATTCCTCGCATGACAATGCCAAAGACATCAC |
| 29141_7 | 8 | [C/T] | TGACAATCCTGGGCATCTACATGCTTATGCGTACAC |
| 29142_26 | 27 | [A/G] | GTCAAACATCACGCAGGTAAGTGCACAGCGAATTCG |
| 29143_28 | 29 | [T/A] | CTTCGTCCTGCAGCAGCAGTATGCACGTTCTTTAAA |
| 29149_20 | 21 | [A/G] | CAGTGGTGTATAGCAGGATGATGCCCAGGCGATGCT |
| 2915_26 | 27 | [T/C] | AACATGGTGCTTGCAATTAGTTGCAGTTGACTGTAC |
| 29163_2 | 3 | [C/A] | TACCGCACAACTGCAAAGTTCTGCTCCGATACCGTA |
| 29166_20 | 21 | [C/A] | CAGGTCTCCGTAGCAGTTACCTGCATTGCACAAACT |
| 29169_19 | 20 | [G/C] | GGTGGCGTTAAAGCACTCCGATGCTTTTCAGCATTC |
| 29170_18 | 19 | [C/T] | ACACTTCCGTACGCATTCCTTTGCTGGTAGTGCTTT |
| 29172_2 | 3 | [C/A] | GCCAGTTTCTGAGCAACAGAGTGCTGCTGCCTGGAT |
| 29173_9 | 10 | [C/T] | TACGTGGAGCGTGCAGTCACCTGCCAGACTTCCACT |
| 29183_28 | 29 | [A/G] | AATCACATCGCAGCAGCACAGTGCAAAAAGGATAAT |
| 29187_27 | 28 | [C/T] | CGAAATCACACCGCAGCATGATGCTTTCAAGACACT |
| 2919_24 | 25 | [T/C] | CAGCAGATGGGGGCACACTATTGCTTTTACACAGAT |
| 29191_8 | 9 | [G/A] | GTGGTGGGGCAGGCACAGGGCTGCACAGGCGAGGCT |
| 29193_2 | 3 | [A/G] | GTAGAGCTGGCAGCAGCCGTGTGCCGAGAGCTGGCA |
| 29194_33 | 34 | [G/A] | AGAAACAGTAAAGCAGGTACTTGCAGGCAGATGGTA |
| 29198_4 | 5 | [C/G] | ACAACACTGACTGCATTCACCTGCCTTTGTACTGCA |
| 29200_8 | 9 | [G/A] | CATACGACGGTAGCACACAGGTGCTAATGCGGTAAT |
| 29204_32 | 33 | [C/T] | TACGCTAGACTGGCACTTAAGTGCTGGGTGCACAGC |
| 29212_34 | 35 | [C/T] | GCTGAAGACAAGGCAGCCCTCTGCACAACAAACTCA |
| 29213_11 | 12 | [T/C] | GGGCCGAAGTTCGCACACAGGTGCGGGAGGACGAGT |
| 29216_29 | 30 | [G/A] | GTCCATCACACAGCAGCCCCCTGCCAAAGGTCACAC |
| 29217_4 | 5 | [C/T] | GCATCATGAACGGCACGGTGCTGCTCGACATGCGGG |
| 29218_9 | 10 | [G/T] | CACTAGTGTGTAGCACTAGCCTGCCAGCTGGGCTGC |
| 29222_30 | 31 | [A/C] | ACTGTATGAACTGCACTGCTATGCCCCAACCCACAC |
| 29226_3 | 4 | [G/T] | CCCGCCAGCTGTGCAGTGCGGTGCCATTCATGAACT |
| 29229_17 | 18 | [T/C] | GAGTGACACTGAGCAGGTCCATGCTGCCACTAGTGT |
| 2923_25 | 26 | [G/C] | ATAAACTGTGTTGCACAAGTCTGCTGTTACAAGCCG |
| 29230_19 | 20 | [C/T] | CTCCACCAACGCGCACCAGCCTGCCACTCAGGGGCT |
| 29250_6 | 7 | [T/G] | GGTCTCTCTATGGCAACAAGATGCACCTGACCGTGC |
| 29251_7 | 8 | [T/G] | ATAATATGGACTGCAGCATTGTGCTTCGTTCACCAC |
| 29252_3 | 4 | [C/T] | CTCCCCTGGCTTGCAGCAGCGTGCCCCTGCACTTCT |
| 29260_30 | 31 | [C/T] | TGCCATGATGTTGCACCATTGTGCTACCTTCTCATA |
| 29263_2 | 3 | [A/T] | AGACACCGATCGGCAGACAGGTGCAGGAACTTCAGT |
| 29266_25 | 26 | [C/G] | CACGAAACAGCTGCATGCTCATGCGCGGGTTTTAAA |
| 29267_27 | 28 | [A/C] | ACCCCCAGGGTGGCAATCCAATGCAGGCTACCAACA |
| 29270_17 | 18 | [T/G] | CCTGGCTGACCAGCATTTCCCTGCAATCAAAAGATT |
| 29275_34 | 35 | [A/G] | TTGCAGCCTGCTGCAATTTCATGCGACCACACGTAA |
| 29276_25 | 26 | [T/A] | CTGCCAGGCTGAGCAGCGAGCTGCCTTTAGCAGCTT |
| 29280_25 | 26 | [C/T] | TGGCACTGCAGGGCATAAGTATGCTCCTCAACAATG |
| 29282_7 | 8 | [A/T] | TAAAGAAATAAAGCAGACCGCTGCATCTGGATTTTG |
| 29288_33 | 34 | [T/G] | TAAGGAATTGCAGCATCAGCGTGCTAATGTGTGGGT |
| 29289_28 | 29 | [C/T] | CAGATACTGACTGCAGCCTCCTGCTGCTCCAGGTCA |
| 29291_33 | 34 | [G/A] | TGACGAGGGGGCGCAGGGTCGTGCGGGAGACACGAC |
| 29294_30 | 31 | [G/A] | AAAAAACCTTTTGCATATAAGTGCCCTGGCGACCAC |
| 29297_5 | 6 | [C/T] | CGAGACCCGACAGCATTTCAATGCACTTTCACCAAA |
| 29299_10 | 11 | [C/T] | AATGGAATTCCAGCATCTTAGTGCCATCTTGTGGTC |
| 29301_10 | 11 | [G/A] | TGACCCTCAGGAGCATCTGTTTGCACTCCTGCAACT |
| 29302_17 | 18 | [G/A] | ATCCTGTCTACTGCAAAGAGCTGCCGTGCTGACTCG |
| 29303_30 | 31 | [A/G] | ACCAACAGAGATGCAGCAACATGCACGAAAGAAGTC |
| 29313_27 | 28 | [G/A] | TGATGTGTGAAAGCAAGGATATGCAGCGAGGAGTCT |
| 29320_26 | 27 | [C/T] | TGGGTGCTGACAGCAGCACATTGCAACGTAGGGTAA |
| 29322_5 | 6 | [C/A] | TAGAACATTTTGGCAGCCTCCTGCATTGAGCCAAGC |
| 29325_2 | 3 | [G/A] | CCACATCTGAGTGCATGTGAGTGCAGCTAGAGATAT |
| 2933_32 | 33 | [A/T] | AGAGTTGCACCGGCACTGAACTGCAACTTCACACAC |
| 29331_18 | 19 | [C/T] | CAACGAGGACGTGCATATCATTGCCCGTTCTGATGA |
| 29336_33 | 34 | [G/A] | CAAGGAGGCAAAGCAGCCAGATGCCATTCTCACGGC |
| 29338_1 | 2 | [T/C] | GTGTGTGTGTGTGCACATGTTTGCCTGTTTACTTCT |
| 29345_24 | 25 | [T/C] | GCGTCTGGCCGGGCATGGCACTGCTGCCGGGCTGCG |
| 2935_26 | 27 | [C/T] | AGCTGAAGCAAAGCATAATGCTGCCACCACCACACT |
| 29350_19 | 20 | [G/A] | TGGCATCATGCGGCAGGAAGCTGCAGTTTCAGTTCG |
| 29353_19 | 20 | [G/A] | GACCACAGGGTGGCAGGGTGATGCTACAGGTAGTGT |
| 29354_11 | 12 | [C/T] | TGTTGGACAATCGCACTTGTGTGCAGGAGATTACAG |
| 29356_10 | 11 | [C/A] | GCCTACACTCCTGCATCCATCTGCTGGGAGCTGCTG |
| 29366_10 | 11 | [C/T] | CATTTTTCTCCGGCACTCAGATGCGAGCCGGGCGGA |
| 29368_20 | 21 | [G/T] | TTCTCAAGTCTGGCAGCCTAGTGCTGGTGGAAAGTC |
| 29371_3 | 4 | [G/C] | GCAGTGCAAAATGCAACTTGATGCGGCTGCAAGGCG |
| 29376_8 | 9 | [G/A] | ACGAACTCGTGGGCAAACTGTTGCCGTGCGTGACTC |
| 29379_17 | 18 | [G/A] | CAGCCACACCCAGCACTGAATTGCAAATATGCAGTC |
| 29384_29 | 30 | [A/C] | TCCTAGCAGATAGCAGACACATGCAAAACCCACAAA |
| 29399_5 | 6 | [G/T] | TGCATGTGTCGTGCAGCTCTGTGCAGAGTGATGTGG |
| 29404_7 | 8 | [C/T] | TGTATCTCTAAAGCAGTGGTCTGCAGGCACATCTGT |
| 29407_18 | 19 | [C/T] | CAGGTTGGCACGGCACTTCGCTGCATACGGGCACAG |
| 29408_9 | 10 | [C/T] | GAGGAGACCCGAGCATTTCAATGCCCTGGCGTGACA |
| 29409_30 | 31 | [C/T] | TAGGTGGCACTGGCAATAGTCTGCCTACCTCTCTCT |
| 2941_25 | 26 | [A/G] | AATCAAGATATGGCACTGTACTGCCAACTGCTTCCT |
| 29418_19 | 20 | [G/T] | TGTCCATCAAGGGCACTTGGTTGCTCCATGGTTAAG |
| 29420_32 | 33 | [G/A] | GTGAATTTTATGGCACTCTTTTGCTAGCTGACGCGC |
| 29421_34 | 35 | [A/G] | AACCTAACAAACGCAACATCCTGCTGCTACAAATAA |
| 29448_32 | 33 | [C/A] | AATGGTAGCTAGGCATAAACCTGCTGACAACACTGT |
| 29450_19 | 20 | [C/T] | ATGCTTCGTGCTGCATCCTCGTGCATCTTGCGATTT |
| 29457_2 | 3 | [G/T] | GTGCTGCAAACGGCACAAGCGTGCTTATCAGCCTCA |
| 29458_25 | 26 | [T/C] | GACGATGAGGCAGCAGCAGGATGCCTCTGCCCGGGG |
| 2946_10 | 11 | [G/A] | AAACCCCAGAGGGCAGGGTGGTGCCTGAATCTCAGT |
| 29469_34 | 35 | [C/T] | AAGTCTGGATCAGCAGTTATGTGCAGACTCAGATCG |
| 29470_10 | 11 | [G/A] | CAGCAGCGTCGAGCACCAGCGTGCGGGCATAACACC |
| 29474_16 | 17 | [T/A] | TGCATCTTGCTTGCACTAGCTTGCTTCTTAGCATTA |
| 29475_26 | 27 | [C/T] | TGTGGACTAACAGCATTTCTCTGCTTCACTGAGCCA |
| 29483_18 | 19 | [T/G] | ACTGGCGAATGAGCAAATTGCTGCCAAATTGTGCCT |
| 29485_17 | 18 | [T/C] | AAAAAACGACATGCATCTCTTTGCACTCACCAGGCC |
| 29486_17 | 18 | [G/A] | GAGTGATCGGTAGCATTGAGCTGCTTCTGTGCTGTT |
| 29490_30 | 31 | [A/G] | ACATGAGGACTAGCAGTGTCATGCATCAGAAGGAAC |
| 29492_11 | 12 | [G/A] | AGCAGCTCTATGGCATGACGCTGCCACCACCATGCT |
| 29494_33 | 34 | [A/G] | AAGTCACCAGTGGCACGTCCCTGCATACCTATTAAT |
| 29498_27 | 28 | [A/G] | TAAAACACAAATGCAGCTTCTTGCTAAACAGCTGAG |
| 295_15 | 16 | [C/T] | TAACACTAGGTGGCACGGTGGTGCTGCAGGTAGTGT |
| 29506_10 | 11 | [T/C] | GCTGGATGCTTAGCACTGTTGTGCACCGTATCTTTG |
| 29508_6 | 7 | [G/T] | AGACCTGCTGATGCAGGCTATTGCCCTGGCAACCAG |
| 29516_27 | 28 | [G/A] | GCTAGGTGTGAAGCATGGGTTTGCCACGTGTTACCT |
| 2952_34 | 35 | [C/T] | GAGAACAGGTCTGCACCACAGTGCCTTTAAACTTCT |
| 29526_2 | 3 | [A/G] | TGACACCACAGGGCAGAGAGATGCTTCTGGGTCTCT |
| 29529_17 | 18 | [T/A] | CCAAGTGGACTCGCAATTTTTTGCCATTCTGGCAGC |
| 29531_17 | 18 | [A/G] | GTGACCTGTCTTGCACAATGTTGCATGAATTGTGCA |
| 29534_9 | 10 | [A/G] | GAAAGACAAACAGCAGGTCCGTGCGAAAGCGGTGCA |
| 29539_6 | 7 | [G/A] | CCCTGTGAGACAGCACCATCATGCTGCAGCATTTGG |
| 29541_4 | 5 | [T/C] | TTCCTAGCCAACGCATGGTGTTGCCTTGTACACCAA |
| 29556_9 | 10 | [A/G] | ACTATGTGAAAGGCAAAATAATGCTGCCCAGTTCAG |
| 29561_1 | 2 | [A/G] | CATGTTGAAAAAGCACTCCATTGCCGGTGATGTAAA |
| 29563_24 | 25 | [T/A] | TTCTAGCCAAGCGCAAATGTTTGCTTATTCATCCAG |
| 29565_7 | 8 | [G/A] | TGGATAGGAAGGGCATTAAACTGCCAATGGGTCTGT |
| 29573_18 | 19 | [G/A] | CCAAAATACACAGCAGTGGCCTGCGTGGTAATGCTG |
| 29577_26 | 27 | [C/T] | GCTGCAGGTAATGCAGATCTCTGCCTCTGGTCTCTT |
| 29582_8 | 9 | [G/A] | GGGAAATGGTAAGCACCCTGTTGCTGGCTGAATCCT |
| 29583_19 | 20 | [C/T] | CCCTTTTCACCTGCATCCCCATGCTTCACAGTCGGA |
| 29589_1 | 2 | [T/G] | GTGACGTGGCATGCAGTCTCTTGCCACTCTGTAGCG |
| 29590_9 | 10 | [T/A] | GGATGATTTTATGCACTGCACTGCTGCGGCACGATT |
| 29592_10 | 11 | [G/T] | TGGAGTTGGTGCGCACTAAGATGCAGTCGAGGCGTG |
| 29600_10 | 11 | [C/T] | CAGGCCGCTGCAGCATTACATTGCTCAGACCCTCAA |
| 29605_3 | 4 | [T/G] | TGTTTCTAACCTGCACCTGGATGCTCGTACTGTTCT |
| 29608_9 | 10 | [T/A] | GTACACCAGTCTGCATAGAAGTGCAGAACTGCAATA |
| 29611_6 | 7 | [G/A] | GAATTGGAAATTGCAGAATCTTGCCGAATCTGCACA |
| 29614_26 | 27 | [C/T] | CAGCCAAACCCTGCACAGCCCTGCCTCGGATGTACA |
| 29616_5 | 6 | [G/A] | TGCCAGTGGGTGGCATGGTGGTGCAGTGATCTATAG |
| 29617_33 | 34 | [C/T] | AAGCTCAGCACAGCAGGAGCTTGCCAGGACCCTCAG |
| 29619_32 | 33 | [A/T] | ATATTTGGAGGGGCACCTGTGTGCCCATAGATAGGC |
| 2962_10 | 11 | [G/A] | CTGGATACCAGTGCAGGTCCATGCTGAACTACTCAC |
| 29622_15 | 16 | [G/A] | TAGTATCAACAAGCAGTAGCATGCCAAACTGGACTT |
| 29624_2 | 3 | [T/C] | TCTGATTCTGGTGCAGTGTGATGCTGTTTTGCCGCA |
| 29626_1 | 2 | [A/T] | TAAGAACAGTCTGCAGGCAGTTGCACTGCATTGACA |
| 29629_17 | 18 | [G/C] | CACTGCAACATCGCAGAGCGGTGCCACGTCCTCCAT |
| 29630_4 | 5 | [T/C] | GCTCTTCATCACGCAATCGTTTGCGTTCCAGTTCTC |
| 29638_9 | 10 | [G/T] | GCACTACCTGCAGCACCACTATGCCACCTTAGATAA |
| 29641_8 | 9 | [A/G] | TGTAGCCGAAGAGCACTCTGTTGCGCAAAGGTCTTT |
| 29642_34 | 35 | [A/G] | TTTTTTTGTGCAGCATTTCTGTGCAGACACACACAC |
| 29648_33 | 34 | [C/T] | GTGCCACATATGGCAACAATCTGCCTTGCTAATCCA |
| 29649_31 | 32 | [C/T] | TTCTTACCTCATGCACAAAGATGCCGAATCCCGACC |
| 2965_8 | 9 | [C/T] | CACCGCCACTTAGCATAATGCTGCTGCAATTAGTGA |
| 29654_28 | 29 | [C/T] | CTGGAGGAGACAGCAGTACTGTGCACCACAACCTAT |
| 29659_8 | 9 | [A/G] | CAATCAGAAACAGCAAAAGCTTGCACAGGTTGGAAA |
| 29661_27 | 28 | [G/A] | AGCAGGGACTAAGCAGCAATGTGCTTGGAGAGCGAA |
| 29662_32 | 33 | [C/T] | GGAGAAAAATGAGCAGTTTCTTGCATCACCAACGAC |
| 29663_3 | 4 | [C/T] | ATTCACACTCTAGCAGCCAGATGCAAAATGCATCAA |
| 29677_6 | 7 | [G/A] | ATGAGCGTTTAGGCACAGCTTTGCACTTTCCCTGCA |
| 29678_4 | 5 | [G/C] | GACTGTATACTGGCAAGCAGATGCAGACTCAGAAGG |
| 29680_1 | 2 | [T/A] | AATAGCGCCCGAGCATAAGTATGCCTGCAGTCTATT |
| 29685_33 | 34 | [G/A] | CCATGGAGGACAGCACTGTTATGCCACAAGAGCGTA |
| 29686_3 | 4 | [A/G] | CAGAAAAAGGCTGCAGAGGCATGCACAGGTCTTCAC |
| 29690_11 | 12 | [T/C] | TTGTAGAGAAGTGCACCAGGATGCAGCTCACGCATG |
| 29700_8 | 9 | [G/A] | CCAGATAGGATGGCATGTCCCTGCGTAATGCTGTGG |
| 29701_2 | 3 | [C/A] | ATCGACTAGCCAGCAGTCATGTGCTATGTGTGCAAC |
| 29706_34 | 35 | [T/C] | GAGATAAATCCTGCAGCACCCTGCTCACTTCTCATT |
| 29707_31 | 32 | [C/T] | CAGTGTCCGGCAGCAACCTTCTGCAGTCCTTTAAAG |
| 29709_26 | 27 | [C/T] | GAGCTGACAGAGGCAGATGAATGCTTCTTCCTACAG |
| 2971_6 | 7 | [C/G] | GCAGTACATAATGCAGTGGACTGCAGTGCTATTCCA |
| 29710_16 | 17 | [C/T] | CCACCTTGCTCTGCAGCGTATTGCTGGGCGCAGGTT |
| 29716_9 | 10 | [G/A] | TCAAAGCGCGGGGCAAAACACTGCACATGCATCATA |
| 29729_4 | 5 | [G/A] | CAGTGAACATGGGCAGCAGGGTGCTTGAGACTCAGA |
| 29730_11 | 12 | [T/C] | GGTCACTTCACTGCAATGCCATGCACGAATGCTTTA |
| 29731_33 | 34 | [C/T] | GCGCACTCAGACGCATCACGTTGCTCCGCGTACCGG |
| 29736_32 | 33 | [C/A] | GCCTGTTGTTAGGCAGCACACTGCGTCATGATCATA |
| 29738_24 | 25 | [T/G] | GGCCAGGATGTTGCAGCGCAGTGCTCCGTAGACGAT |
| 29741_8 | 9 | [T/G] | CTAACAGTTGGAGCACAATGCTGCCAGAGTGGAGAA |
| 29745_2 | 3 | [G/A] | TTGCGACTCACCGCACTCGTCTGCGCTGACGTTGAG |
| 29746_18 | 19 | [T/C] | GCATGACGATATGCATTTTGCTGCTAATCCACTGAT |
| 29750_29 | 30 | [C/T] | GGAGCGGCTGCTGCATGCTTCTGCACATGCTGAACG |
| 29751_27 | 28 | [C/T] | GCGAGCAACTCGGCATGAGGATGCGTGCAAAAAGCA |
| 29754_29 | 30 | [C/T] | CATTATTTCCCAGCATCGTCCTGCAAACTCTTTCAC |
| 2976_7 | 8 | [G/T] | ACACCCGGTGCTGCAGGGCAATGCCTACGCTGACTC |
| 29760_34 | 35 | [C/T] | CATCGCCCCCGTGCAGACGCTTGCGCTTGGTCAGCC |
| 29763_6 | 7 | [C/T] | GGCTCGCAATCAGCACTACTTTGCTCCAGAGTATGG |
| 29764_27 | 28 | [C/A] | GATAATACGAAGGCACCTGTCTGCCACCTGCACTCT |
| 29769_20 | 21 | [T/G] | GTCCTTCGACACGCAGCTGTTTGCCGGAGATTATTT |
| 29770_19 | 20 | [A/G] | TTTGGCTTGGAGGCAGAGCAATGCTTTATGTGTAAC |
| 29773_1 | 2 | [C/T] | CCTCGTGATGGAGCAGGTTTTTGCCATGTGGCGAAT |
| 29778_1 | 2 | [A/G] | GAGGCAGCATGAGCAGCTTGATGCAGGCGCTGATGT |
| 29780_15 | 16 | [C/T] | AGTCAACGGGTGGCACGATGGTGCCACAGGTAGCGG |
| 29785_26 | 27 | [T/C] | GAGTCTAAAGCCGCAACAGATTGCCTCAGCTTTGGC |
| 29792_6 | 7 | [C/T] | GTCTTGCGCCAAGCAGAAACCTGCAACATTGTGGTA |
| 29795_15 | 16 | [G/A] | TATTCCCCTGGTGCAGGTCGATGCCTGGCTTCATTT |
| 29796_27 | 28 | [G/T] | GCCTATAATCCAGCAGCAGCCTGCTGAGGAGAGCAG |
| 29799_28 | 29 | [C/T] | GGTTAATCGCGTGCATTCGGCTGCGTGTCATCGGTA |
| 29803_33 | 34 | [G/A] | ACACAGGGTTTAGCAGTTTAGTGCCTCTGAGACGTA |
| 29804_29 | 30 | [C/T] | GATACGTAGCCAGCACCATTTTGCACGCTCCATCAT |
| 29806_34 | 35 | [C/A] | GTGTGTCGCTGCGCATTCATTTGCATAACATAAGCT |
| 29807_11 | 12 | [C/T] | GCACAAACAGGCGCACACACATGCACACACACACAC |
| 29811_29 | 30 | [T/C] | GTGAATCATAACGCACCCGAATGCCTGAATGCCTCT |
| 29819_33 | 34 | [C/T] | GCTAAGCTAACTGCAGAACTTTGCAGTGACAGACTG |
| 29821_19 | 20 | [A/T] | TTTGGGAACACAGCAGGAATCTGCTGGCAATAAACG |
| 29822_33 | 34 | [T/A] | GACCTGTTTGACGCAGCGCAGTGCATAACGGAGTGT |
| 29823_29 | 30 | [G/C] | TCGAGTCGGTGTGCATCCTTTTGCTAGCAGCACATT |
| 29827_34 | 35 | [G/A] | AGGAATCAGAGAGCACGTGCTTGCTACACCTCATGA |
| 29828_28 | 29 | [G/A] | GGGTGCGCTGATGCATAGTGTTGCAAAAGCAATCAT |
| 29831_8 | 9 | [C/T] | ACAAGAAGTCTGGCAGGGTGCTGCTAGCGGCATCAT |
| 29832_7 | 8 | [G/A] | CCTGAGTGAGTAGCACAGTGGTGCCGTCTGTCACTC |
| 29833_2 | 3 | [G/A] | GTGATGGTGTGGGCAATGTTCTGCTAGGAAACCTTG |
| 29834_11 | 12 | [G/A] | CTGTGACAGGCGGCACCCGCATGCTATGGAAAGCTG |
| 29838_2 | 3 | [C/T] | CACACACACACAGCATTTCTTTGCAGTGGCCTAAAG |
| 29841_20 | 21 | [G/T] | GCAGACACCCTAGCAGTAGCGTGCTAGCATTAGACT |
| 29846_32 | 33 | [G/A] | ATGATGGTAGTGGCATTGTGGTGCTGCAGGTAATGT |
| 2985_2 | 3 | [A/T] | ACACACGTGCACGCATGCACATGCAACAACAACCAC |
| 29850_15 | 16 | [C/T] | AATTAACTACCGGCACAATCGTGCTGTTAACACGTT |
| 29854_15 | 16 | [A/C] | TCCTTCAGTTGTGCAAAAGCATGCACATGTGCCTTT |
| 29855_9 | 10 | [A/G] | AGGATAATGAATGCACGCATCTGCCATCAGTCCGAA |
| 29857_1 | 2 | [G/T] | TGGTCTCTTCCAGCAGGACGATGCCCAACCACACAC |
| 29858_4 | 5 | [A/T] | TCCTAATCAAGAGCACGAGATTGCTCCTCAAAGTGA |
| 29865_31 | 32 | [G/A] | CAGTTTATACGTGCATGTCGCTGCAGTCACTGTATA |
| 29868_24 | 25 | [G/T] | GTACTATGTTGCGCATACGCATGCGAAACACAACCG |
| 29874_25 | 26 | [A/G] | ACACCACCTGTTGCACCACCATGCTACCCATTCTTT |
| 29875_7 | 8 | [G/A] | TACCGGAACCAGGCACTGACCTGCTGCAGAGTAATG |
| 29877_2 | 3 | [C/G] | TCCGATGCCTCAGCAGTATTGTGCTCATGCAGATGA |
| 29888_8 | 9 | [C/T] | GTCTCTCTCGCCGCACCTTTCTGCTTTATTTTACGT |
| 29889_26 | 27 | [T/G] | GCATACAAAGTGGCACAATGGTGCCATGAGTGTGCA |
| 29890_6 | 7 | [C/T] | GGAACACAGTCAGCAAAAATGTGCACGAGCGCAGCT |
| 29891_2 | 3 | [C/T] | TCCGTTACATTAGCACTGCGCTGCGCTCTCCGGTTC |
| 29893_3 | 4 | [C/T] | GATCGCTACAGAGCAGTGCCCTGCCCTGGCTCTCGG |
| 29894_7 | 8 | [A/G] | GACAGCAACACTGCATCAGGATGCGGACCACAAAAG |
| 29895_7 | 8 | [G/A] | ATGCGTTGATTGGCAGAAACATGCTACAAGGATTTA |
| 29896_34 | 35 | [C/T] | GAAGTCAGAGCTGCAGGCATGTGCCTGGGAGGCCCG |
| 2991_4 | 5 | [C/T] | TCCTCATGCTGTGCAACATCGTGCTGGACTGCGCGA |
| 29919_26 | 27 | [C/T] | ACAAAAGTCTCAGCAACACTTTGCCTCACGAAAGTG |
| 29926_25 | 26 | [C/T] | AATGCAATAACCGCAATCTAGTGCACGTCAACTTTT |
| 29934_16 | 17 | [C/T] | CACACACTACCTGCATCACCCTGCTGCTCAAGCCCA |
| 29936_7 | 8 | [A/C] | CCGGCATACCCTGCATCCCCTTGCTCTCTCCTCCTC |
| 29941_7 | 8 | [C/T] | AAAAATACGTGAGCAGATTTTTGCTAGCACGCTGCT |
| 29943_16 | 17 | [A/G] | CTAATCTCTGAGGCACACCGATGCTGTCATTACGCA |
| 29946_2 | 3 | [C/G] | AACTGCCCAAAAGCACAATACTGCCACCACCATGCG |
| 29952_29 | 30 | [T/C] | CACAGGGGAAAAGCAATATCGTGCCAAAATGCGCAG |
| 29955_24 | 25 | [T/C] | AAGGTGTTCACTGCACCATGTTGCTTCCCGAATACC |
| 29956_1 | 2 | [A/G] | AATAGGATGCTGGCACAAAACTGCAAGTCAGAGGAT |
| 29957_24 | 25 | [A/C] | AATCATGTGGCAGCATCACAATGCAGAAAATGATGC |
| 2996_31 | 32 | [G/A] | CCACCTTTAACAGCAGTCAGCTGCAGGCACTGGAGA |
| 29960_18 | 19 | [A/G] | GCAGTCGCCACGGCACTTATATGCTGAAGTGCCCTT |
| 29963_9 | 10 | [T/C] | ACAGGAACTTAAGCACACTCGTGCACTAGGCTTTTT |
| 29964_5 | 6 | [C/T] | ACCCTTACGTAGGCATGGCCATGCACACACATACAC |
| 29965_11 | 12 | [T/A] | CACACACACAGAGCAGTGCAGTGCGAGATCCCGAAA |
| 29966_8 | 9 | [T/C] | CATGTTGGTGCTGCAGGTAGGTGCATGTCATCACCT |
| 29970_19 | 20 | [T/C] | AGCCACCCCAGGGCAGCTTTATGCCCACCCAGTCTT |
| 29973_26 | 27 | [T/C] | CGAATTGTTTTGGCAACAGAATGCCATCTTCCCTTA |
| 29983_11 | 12 | [A/G] | GCAGGAGGTCTAGCATCAGTGTGCGTTTTTATAACA |
| 29985_10 | 11 | [A/G] | GTATTTGTCAAAGCAGAAGGCTGCCAACTGTGTGTG |
| 29995_5 | 6 | [C/A] | GACACCGAGCGTGCAGTACTTTGCCAAACTGTTTAC |
| 29999_18 | 19 | [A/G] | TAGTGCAAAGCAGCAAACAACTGCCATGGTCATTAG |
| 3_7 | 8 | [G/A] | TTTAAAGGAATTGCAGTGTGTTGCTTTGCATTCGTG |
| 30_19 | 20 | [T/C] | TTCAGTGTCTGTGCAGGTTTCTGCCAGCCAGGTGGT |
| 3000_32 | 33 | [A/G] | TTTTCAGCAATTGCACGTTGCTGCACAAGGTCAGAC |
| 30006_8 | 9 | [G/A] | AGTGTAATGAGTGCATCATCTTGCACGTGATGCAAG |
| 30009_7 | 8 | [G/T] | CCGAGCAGTACCGCATGCAGCTGCGCAGGCACCGTG |
| 30011_7 | 8 | [C/T] | GCAGGGGCTGTAGCAGAAATGTGCAGGGTGGTGGGT |
| 30012_11 | 12 | [A/G] | GTCAGCATTAAAGCAGACGCATGCCACACTTGACTG |
| 30013_31 | 32 | [T/C] | CTGGAGTTCGCTGCAGAGTCCTGCCCCACTATATCA |
| 30016_29 | 30 | [T/G] | AACAAGTGACGGGCACATGGGTGCCAAATTGCTTTG |
| 30023_10 | 11 | [G/C] | ACCACTGAAACAGCACGTGAGTGCACACAGTACTGT |
| 30024_32 | 33 | [C/T] | CCTGGGCGTTGTGCACAGGGTTGCCCAACACTCTAG |
| 30028_8 | 9 | [G/A] | GTTTGTCTGTGTGCATGCTTTTGCAAAGGGGGTCCC |
| 30029_30 | 31 | [C/T] | GGGTTTGCTTTGGCAAGCCACTGCCTCTTTCATGCC |
| 3003_5 | 6 | [C/T] | GTGCATCGGAGCGCAGGCTCGTGCAGTCCAGATCAT |
| 30030_29 | 30 | [T/A] | GTGAAGCCAGGTGCATAGACGTGCCTTTTTGTCGTG |
| 30032_25 | 26 | [T/C] | CCTCTCTCTCCTGCAGTAACCTGCGTAATGTTCTCT |
| 30040_15 | 16 | [G/C] | CTTTGTAGACACGCAGGATGCTGCGCTGATTATGTT |
| 30047_16 | 17 | [C/A] | GTGTAAACGGAAGCAGCTTGTTGCGAGGACGTACGA |
| 30052_7 | 8 | [G/C] | GTGGCGCGGTTAGCACAGCGTTGCTCTGAATGACTG |
| 30055_15 | 16 | [T/A] | ATTCCTCTCGCAGCATTGCAGTGCTGCGTTGCTATA |
| 30061_2 | 3 | [C/T] | GTCAAAGCTCCCGCAAAGAAGTGCACGAAGATGAAG |
| 30062_17 | 18 | [A/C] | TTCAAGCAGGGAGCAACAGTGTGCATATTTTGCAGA |
| 30070_3 | 4 | [C/T] | CTGCTATTTTGTGCATTGCACTGCTGCCACCTGCAT |
| 30073_31 | 32 | [G/C] | AATACCAGGGCAGCAAGTCATTGCAGGGAATGAACT |
| 30074_17 | 18 | [C/A] | AAAACCCAAAAAGCAGACAGTTGCCTGACAAAGTCC |
| 30076_15 | 16 | [G/A] | GGGTGGTGAACAGCAAAAATCTGCCCTACGACCTCC |
| 30078_5 | 6 | [G/T] | GCGTGGACGTCGGCACCGTGCTGCAGCAGGTACTCT |
| 3008_31 | 32 | [G/C] | TCCTTCATACGAGCAGACACCTGCACATCTGGATCA |
| 30083_20 | 21 | [C/G] | CACGCAGGGTCAGCAGCTGCCTGCAGAATAAAGTCG |
| 30086_19 | 20 | [C/T] | TGTTGTGAAAGTGCATCTCCCTGCGTGTGCCTGTCG |
| 30099_33 | 34 | [G/A] | CTTAATGACGGCGCAGTGGCTTGCTCTTGCCCCGAG |
| 30100_1 | 2 | [T/C] | GTTGGAAAGTTTGCAACTCTCTGCTTTGCAGAACTC |
| 30101_7 | 8 | [C/T] | TGTATTTCTGTAGCAACCGACTGCTTCACGACTTGA |
| 30108_7 | 8 | [T/A] | GGCTTCCTCCAGGCACTCGAGTGCGTGTCTGTGTCC |
| 30109_34 | 35 | [G/A] | CGAGTGCAGGAGGCATCAGGTTGCTCGTTTCGCCGC |
| 30116_29 | 30 | [C/T] | ATTACCCACAATGCACCACTTTGCCGGCGCTCACTG |
| 30117_1 | 2 | [C/T] | GTGATACAGAGTGCACCTGGATGCCCAGCTCTCTGG |
| 30118_28 | 29 | [G/A] | TCTGGCGAACCTGCAGTAGCATGCACAGGAATATGA |
| 30125_2 | 3 | [C/A] | GTCAAGCAGCCAGCAGCTTAATGCTGCTGAGAAAAG |
| 30131_8 | 9 | [C/T] | CATCCTTCCACTGCACAGAAGTGCTGATTCAGCATT |
| 30136_16 | 17 | [C/G] | TGTCCGGTTCGTGCAGCTCCGTGCGGCGTGTGATGA |
| 30137_1 | 2 | [C/T] | TCTGAGCTTTCTGCACTCACTTGCGATGTGGTGGGG |
| 30143_34 | 35 | [C/T] | CTGTCATTTAATGCATCCACCTGCCTTGCTTTCTCT |
| 30149_28 | 29 | [C/T] | GAAAATCTCAAGGCATCAGGTTGCTCCTCATGACAA |
| 3015_19 | 20 | [C/T] | CACTGATTGTGTGCAGCACCGTGCACTAGGCAAAAG |
| 30150_5 | 6 | [G/C] | ATCTGCAGAGCAGCACGAAACTGCTCGTGAGAAACG |
| 30155_4 | 5 | [C/A] | GAGACGACTGCAGCACCTGGATGCTTTGACCTTGCT |
| 30159_27 | 28 | [T/A] | AGATGTAGGCTGGCACAGACTTGCCCATTTTTTGAT |
| 30161_4 | 5 | [C/G] | AACACTAAACCTGCAGGCCTCTGCTAAAAAGCTGAA |
| 30169_34 | 35 | [G/A] | CCATGATGCCTGGCACACAATTGCAGCCTGTGGTGA |
| 30174_30 | 31 | [C/T] | CCCTGTTTTACTGCACCGAGCTGCTCCAGTCTCTTA |
| 30185_19 | 20 | [G/T] | CAGTAGAACATAGCACACTGGTGCAGCAAGCTTACC |
| 30187_25 | 26 | [G/A] | TTCCTGCCCTGAGCACGAAGATGCCGTGTGTGTGCT |
| 30189_17 | 18 | [G/T] | AAATAAGAAACAGCAGCGACATGCAGCAAGTAAACA |
| 30190_8 | 9 | [G/C] | CCACAGCAGTGCGCAGTGCAGTGCAGGTAGTTAGCA |
| 30199_3 | 4 | [G/A] | GCCGAGCCGGCTGCATTCTCCTGCCGCCCATGGTGA |
| 30201_27 | 28 | [T/C] | TTGTAAAAGCCGGCAGTATTGTGCCTGTGCTCCTCG |
| 30204_31 | 32 | [T/C] | TGGTGCTTAAAAGCAATTTCGTGCGCTGTGCTATGT |
| 30207_8 | 9 | [G/C] | TGTTAGCTGGTCGCATGGGTGTGCTGCAGTCTTAGA |
| 30210_24 | 25 | [C/G] | CCTGTCCTTGCTGCATAACGCTGCGAACACCGTGCT |
| 30213_18 | 19 | [C/T] | ATGTTCACCAGTGCAATGCCATGCTGGTTGTGCCCA |
| 30217_27 | 28 | [C/A] | AGTCGCACAACAGCAGCAGCTTGCTCTCGTCTGCGT |
| 30222_3 | 4 | [G/A] | GGGGCTGAAACGGCAGCCTGATGCTGCGGGATTTGA |
| 30223_1 | 2 | [T/C] | TTACTGCAGCTGGCACATCTGTGCATGTCTGGATGC |
| 30228_3 | 4 | [G/A] | CTCGCTTATCTGGCAGCTGTTTGCTAACTTACCTTA |
| 3023_27 | 28 | [A/G] | GCCAAAGTTGGGGCAGTGGGATGCTTTAAATGCTAC |
| 30232_30 | 31 | [C/T] | CTGGAGCAGTGGGCAGATCTGTGCCTTACCCACGGG |
| 30233_3 | 4 | [C/T] | CTACGGCATCGAGCACGCTCTTGCTCTGGATGTGCT |
| 30236_19 | 20 | [T/A] | GGCAGACGGTGCGCAGAAATATGCCTTTCCAGGCTG |
| 30237_18 | 19 | [T/C] | TGCTGGCTCATGGCACCCTATTGCTATGTTGGCTGG |
| 30245_31 | 32 | [A/C] | GCGATTGTGTACGCATGTGTGTGCTTGAAGGAATTG |
| 30247_15 | 16 | [T/C] | GAGACTGCCACAGCATTGCCGTGCAAGCCGCAGCCT |
| 30257_6 | 7 | [C/T] | TGTGCTTGGCGTGCATGCTGGTGCATTTCATTTAGC |
| 30263_11 | 12 | [C/G] | TGTGCAGAAACCGCATGTGGTTGCAGAGGAAAACGG |
| 30264_26 | 27 | [G/T] | GAAACATTGTCGGCATTTGGATGCTCGGTATCTGAT |
| 30265_10 | 11 | [G/T] | GGAAGCTGGATAGCATGGAGCTGCTAGCAGGCACTT |
| 30267_1 | 2 | [T/C] | GTACTGCACACTGCACCGTCCTGCACTGTCCAGTGG |
| 30279_15 | 16 | [C/T] | TGCACTGTGCCAGCACCAACCTGCCAACACTATCAT |
| 3028_7 | 8 | [C/G] | AACATGTCTGCTGCAGGTACCTGCTAACAGAGGGGT |
| 30284_3 | 4 | [T/C] | GAATTTGTTCCAGCATCCTGGTGCTAGGACAGAGAT |
| 30286_24 | 25 | [G/A] | GCAGCGTTTTATGCAAAGCCATGCGAGGCAAATTCA |
| 30290_17 | 18 | [A/G] | AATGTAGTGCGTGCAGCACCATGCCAGACTACAAGA |
| 30291_34 | 35 | [C/T] | TTGTCGAACCTGGCAACCGTCTGCTGATGTGCTTCC |
| 30292_3 | 4 | [G/T] | GAAGTCCCACGGGCACACCTCTGCCATCCTGCCACT |
| 30299_25 | 26 | [A/C] | AGGATACAGCAGGCAGAATTGTGCCACACACACACA |
| 30304_2 | 3 | [A/G] | TCAGGACACAGTGCATCCTGGTGCTCCAGCAGCAGC |
| 30311_15 | 16 | [G/A] | GGGTCGATGGTGGCAGTGTGTTGCTGCTTCTGCTGC |
| 30315_27 | 28 | [G/C] | ACAGATAGTAGTGCAGTGTTCTGCCCAGCCCACCCC |
| 30316_4 | 5 | [C/T] | TCACCGCTCCGGGCACATGTGTGCCCATTTTACGTG |
| 30319_7 | 8 | [C/T] | ACATCAGCGCCAGCATCAGGCTGCTCTCTCGGACTC |
| 3032_25 | 26 | [C/T] | CCTTACTCAGGCGCAGACTCATGCTCATGACCCCCA |
| 30321_34 | 35 | [C/T] | GGTACTGCATGTGCAAAAAGTTGCCCTCACCCTTCT |
| 30322_33 | 34 | [G/A] | TATATCATGTTTGCAGGTCGGTGCATTTCCAAGGAA |
| 30328_28 | 29 | [T/A] | GTGAAGGAAAGTGCACTGCACTGCTAGCTTTTTTTT |
| 30331_33 | 34 | [C/G] | AGATCATGGCAAGCACACACTTGCAATTATCTCGCT |
| 30336_5 | 6 | [A/G] | AGAAGATCTACCGCAAGGCTGTGCTGGTAGTCCATC |
| 30337_11 | 12 | [G/A] | AAGGGGCAAAAGGCAAAAGCATGCCAACAGAAAAAC |
| 30339_29 | 30 | [G/A] | GTCCAAGCGAAAGCAAGAACTTGCTGACCGCCTGAA |
| 30340_19 | 20 | [C/T] | CCCGTACTGTTAGCAGCGTCGTGCTATGCATCATAC |
| 30343_15 | 16 | [A/G] | TCTCAGCCAATAGCAAGACGGTGCGTCCTCACTGCT |
| 30353_9 | 10 | [G/A] | ATATAAACAGAAGCAGCCCTCTGCATGAGGGAGGAT |
| 30358_25 | 26 | [T/A] | AAGCCATTAATGGCACATGCCTGCTTGTCATGAAAT |
| 30364_4 | 5 | [A/G] | ACTCACTCCTCCGCACCCGGTTGCCTTGACAGTAGA |
| 30371_2 | 3 | [T/C] | CATGTTCAGTGTGCAGCAGGCTGCACTGGCCCCAAA |
| 30374_17 | 18 | [T/A] | GACGCTAAAGCAGCACTAAGTTGCTCCTTCTGGGGC |
| 30375_16 | 17 | [G/A] | CTTACATCAGCAGCATGCCAATGCTAAACTTGAGTA |
| 30381_15 | 16 | [G/A] | TGTTCCTGGAAAGCAGTGCGCTGCCTCAGTACGGAG |
| 30382_20 | 21 | [G/A] | ACATTTGGGATGGCACAATGGTGCCACAGGTAGTGT |
| 30383_1 | 2 | [C/T] | CCTTGTAATGGGGCACGTCGGTGCACTTGAGCATGC |
| 30384_9 | 10 | [A/G] | GGTGTGTGTAGAGCAGCGCTGTGCCGAGCTGAAGAG |
| 30385_25 | 26 | [G/A] | TGTGTGCTGCATGCAGAGAGCTGCTAAGATGAAGGT |
| 30388_9 | 10 | [G/A] | TGTGATGTTGAAGCAGAGACATGCTGGACCAGTAGT |
| 30392_10 | 11 | [G/A] | ACGCATTTCCGAGCAAGCACATGCTAGCCTTCACCT |
| 30393_5 | 6 | [C/G] | TGGTACAGCTGTGCATGACGCTGCTGAAGTGCCTTG |
| 30397_9 | 10 | [G/T] | TGGCCAAAAGCAGCAGGAATATGCAGGAATGGGCAA |
| 30404_15 | 16 | [G/C] | GTTGCTGGTAGAGCAGGCCGATGCTACGACTGCAGA |
| 30405_24 | 25 | [A/G] | TGCAGCTAGCACGCAAAAAGCTGCAGATCTTATGGA |
| 30408_1 | 2 | [T/C] | CTCGACTGAAGCGCATGACCATGCATTGCAAAGCCG |
| 30418_2 | 3 | [G/A] | TCGAGGTGAAGCGCAACGATCTGCTCACACAAGAAA |
| 30424_19 | 20 | [A/G] | GTTAGAGGACCAGCACAAGAGTGCAAAGAGCCCTAT |
| 30433_1 | 2 | [T/A] | TTACCATAGAGGGCAGGTCGTTGCTGTCAGCCTCAT |
| 30438_6 | 7 | [T/C] | TTTCCTTGTTCAGCACCGCTCTGCTGAGGTTCACGA |
| 30440_1 | 2 | [T/C] | CCGTGTTTGACAGCACAGTAATGCCATTCCTCCCAC |
| 30445_29 | 30 | [C/T] | TAGCCTTATCTGGCACATCCTTGCCTCTGCCTCCTG |
| 3045_31 | 32 | [C/T] | ACTGCTGTCTATGCACACAACTGCCTTCTGTCAGCA |
| 30452_33 | 34 | [T/C] | CTAGCTCCATTTGCAGACCAATGCATTTTCATCCGA |
| 30454_1 | 2 | [A/G] | AATGTGTGTTATGCACCCCACTGCCCAGTGATTGTT |
| 30456_25 | 26 | [G/A] | ACATTTCCATCTGCAGGTAGTTGCTGAACGACTGTT |
| 30458_31 | 32 | [G/A] | ATCTAACCTCGAGCAGGAGGTTGCTAAATACGAGAC |
| 30460_25 | 26 | [A/T] | CACCGACGGCATGCACCAGCATGCTAAACATGCACA |
| 30464_33 | 34 | [G/T] | GCGTCCCAAACCGCACAGCAGTGCACTTAATAGGCT |
| 30465_25 | 26 | [C/G] | ACGCCTGAAAACGCATTCACGTGCCCTGCCATGGGA |
| 30466_1 | 2 | [C/T] | CCCTCTCTCTCCGCAGGAAGCTGCACAGCGGAATGA |
| 30468_24 | 25 | [A/C] | TCTATAATGGGTGCAGTGTGGTGCCGTGTGTGTATG |
| 30474_9 | 10 | [C/T] | CTCAGGTGCCGAGCAGTCACTTGCTGTTTCTGCCTT |
| 30476_10 | 11 | [C/T] | AGTGCAGACACAGCAGGTTACTGCCATAGCTGTGTA |
| 30478_32 | 33 | [T/C] | CAGGGGAATGTGGCATGTCTCTGCTTCTCATCTACT |
| 30479_17 | 18 | [C/T] | ACTGTAAGTCAGGCACTCTTGTGCAGCATCAGTGTC |
| 30481_6 | 7 | [C/T] | TGACATCATCAAGCATCCATGTGCCAATTAGTGCAA |
| 30482_2 | 3 | [G/T] | AAGCAACATTCTGCACAGAAATGCTCTGCTGTACAT |
| 30484_4 | 5 | [G/A] | CTTCGGGTTCCCGCAGTCGTATGCACAGCGCACATT |
| 30486_10 | 11 | [G/C] | GAAGAGCGCGGAGCAATCGCGTGCAGACAGGAATTC |
| 30488_27 | 28 | [T/A] | AATTGCAACCTGGCACAACATTGCAAGTGGGTACAT |
| 30490_29 | 30 | [T/C] | GAGGAAACCGCCGCACACTCATGCACTGCTAGACAC |
| 30492_26 | 27 | [A/G] | ATAAACAGTTCAGCACAGTGCTGCCTAATCTCAATT |
| 30496_18 | 19 | [G/A] | TAAAATGCTGGAGCAAATGAATGCGGTGGAATTCTT |
| 305_15 | 16 | [T/C] | TTGAAGTACAATGCATGACTGTGCCTTCGTGTTTAA |
| 30505_3 | 4 | [C/T] | ACGTCTCACTCTGCAGAAGCTTGCTTTCCTGAAACC |
| 30509_28 | 29 | [C/T] | CAAAAGCCAGGGGCAAGTTAATGCCATTCAGCGCCA |
| 30510_19 | 20 | [A/T] | ATGTTTTACGACGCACACTAATGCATGGTGAAGAAC |
| 30512_19 | 20 | [C/T] | CGTTCAATCACAGCACATCCGTGCTCCACGCTGCTG |
| 30517_7 | 8 | [C/T] | TGCTGCACGTGAGCACAGTGGTGCAGACTCATACAA |
| 30522_27 | 28 | [G/A] | TGTCTTGTGGTGGCAGCATCATGCTGTGAGGGTGTT |
| 30525_24 | 25 | [A/G] | TGCCCAATCTCGGCATGGCACTGCATGATGCCCGGT |
| 30526_2 | 3 | [G/A] | CCGTCTGTGAAAGCACGCCCATGCTGAGTCTCCCAC |
| 30535_33 | 34 | [G/A] | CGTTGCGTAGTCGCAAAAATCTGCTTTTTATCCGTG |
| 30540_26 | 27 | [T/C] | AAATCCTCCATTGCATGGCCCTGCCTCGTTCCCCTA |
| 30541_4 | 5 | [G/T] | CCCCGGTGTGCAGCAAGGGACTGCAGATTCACACTA |
| 30551_8 | 9 | [A/G] | TGTACCAAAGGAGCACAATTCTGCTGTGTGGAAGAA |
| 30553_15 | 16 | [G/A] | GCTGCTGCTGCCGCAGCTGTATGCGCTCCTGACATG |
| 30554_25 | 26 | [G/A] | GCTGGGTGTGAAGCAGGACTATGCCGATGCATATTC |
| 3056_9 | 10 | [C/T] | CAGGATCAGCGAGCATTGTCATGCTTATCACCTATG |
| 30560_25 | 26 | [C/A] | TCTCCTACAAGTGCATTTGGATGCCCTCTGATGTTG |
| 30562_26 | 27 | [T/G] | GCACCTTTCATGGCAGAACCTTGCCATTGGCCTCTG |
| 30570_3 | 4 | [A/G] | AATAGTTTAATTGCACGGCTTTGCCTCCGCAATGCA |
| 30572_15 | 16 | [C/T] | CAGCACTCTGGAGCACGACTCTGCTGACCCTAATGA |
| 30575_25 | 26 | [C/G] | AACAGACCCCAAGCAGTACAGTGCAGAGGATTACTG |
| 30582_7 | 8 | [T/A] | GTGGAACTGAAGGCAGGCTGGTGCGTAACAGGTAGA |
| 30583_11 | 12 | [C/T] | ATCGCAGGCATCGCACAAGCATGCGTCCCTTCGGAC |
| 30586_2 | 3 | [G/A] | TCGACGAGCTCAGCACCCGTGTGCCGACCCGCTTGG |
| 30588_18 | 19 | [C/T] | TTAAATGTAGCAGCATGGCCGTGCGATTGGTCATGT |
| 30590_16 | 17 | [G/A] | GTTGTATGGTTGGCACGAGGCTGCAAACTAATACCA |
| 30596_15 | 16 | [C/T] | TGCCCTTCATCCGCACGGGCATGCTTTACTTCTAGT |
| 306_11 | 12 | [C/A] | AAGAATGTCAACGCAAAGGTTTGCAATTAGCCTTCC |
| 3060_34 | 35 | [T/C] | TAAGCCTTATGCGCAGTAAAATGCACGTAATCTTTA |
| 30601_26 | 27 | [G/T] | GACTGCCCCATTGCATACAACTGCACGCCATTGTAA |
| 30610_15 | 16 | [T/G] | ACACACACACACGCATGCACGTGCATACACTCAAGA |
| 30618_18 | 19 | [A/C] | CAGTGACCGGGAGCAGGAAGCTGCCAGCTCACCTGA |
| 30619_24 | 25 | [G/C] | TTGGCACCCTGTGCACCTGGGTGCGCTCTGGTCTAA |
| 30626_6 | 7 | [G/A] | GTGTGTGTGTTGGCATTCCTGTGCTCTGGTGCTCGG |
| 30628_20 | 21 | [G/A] | TATGTCAGTGCAGCAGAGGCGTGCAAATGTCCTCAG |
| 30634_34 | 35 | [C/T] | GAAATCATTACGGCAAAAAGCTGCAACCCAATCTCA |
| 30636_31 | 32 | [G/A] | TGTGTTATTTGTGCATATGCGTGCACCTATCGGCCA |
| 30642_1 | 2 | [C/G] | CCTCCCCGGTCAGCATGGCCATGCCGCCGCAGGGCT |
| 30646_33 | 34 | [T/C] | CTGAGCTCATCGGCATGGACGTGCTCGTGGATCTGG |
| 30648_34 | 35 | [A/T] | TAACGCGAGCATGCATGGGAGTGCCTTCATCTGGAA |
| 30653_18 | 19 | [G/A] | GAACAAGCCTAAGCAGACGTCTGCATGCTGAGAAAT |
| 30658_15 | 16 | [A/C] | TGGTCTGGGGTGGCAAAGTCATGCAGCTGGTAGTGT |
| 30661_4 | 5 | [T/C] | ATGTTTTACGCAGCATGTTTGTGCTGGAGGGCCCCA |
| 30662_8 | 9 | [A/G] | TCGTGTTAAGAGGCAGAAATCTGCACTCGCATTCAA |
| 30666_34 | 35 | [G/T] | AGCTGAAGTTGTGCAGGACACTGCGATGACCATAGT |
| 30669_33 | 34 | [T/C] | AAATTCTCAAGCGCATCATCGTGCAATACAAAGTCT |
| 3067_19 | 20 | [A/G] | TTGGTTCCTGCAGCAACTCGCTGCCCAAACAACAAG |
| 30672_18 | 19 | [G/A] | AAATAATACGCCGCATCCGCATGCCAAATGGAGAGT |
| 30674_34 | 35 | [A/G] | TCCTGCGTGCGTGCATGCCTGTGCGTGTGTGTGCAT |
| 30675_4 | 5 | [G/T] | AATGGCTTTAAAGCAGAGGGATGCGGATACGTAGTA |
| 30680_20 | 21 | [C/A] | ATTTGACAACATGCAGTCCACTGCGGCAAAAAAGGT |
| 30681_25 | 26 | [T/C] | ACACACATGGTTGCATCGACCTGCTTTAGTTCACAG |
| 30692_31 | 32 | [G/A] | CCTGAACTTTAAGCACCTCTGTGCTGAGCCTGAACA |
| 30696_17 | 18 | [C/T] | GAAAAACACCCGGCACTCTGATGCGCGCACGCTCTA |
| 30697_34 | 35 | [G/A] | TGAGCTCCAGCAGCAGCTTCCTGCAAATTCCAGCGG |
| 307_1 | 2 | [G/A] | TGAACAAGACTGGCAGGCTGCTGCAGTATAAAGCTG |
| 30700_27 | 28 | [T/A] | ATAGGCTTTAGTGCAGTGAAGTGCTCGTCTTATTTA |
| 30702_1 | 2 | [G/A] | AGAGCATTTTCAGCAAGCACATGCTGTTTTACCATC |
| 30703_28 | 29 | [G/A] | CTGCGGACCTCAGCAGCGTGTTGCTCCTGATAGAAA |
| 30705_9 | 10 | [C/T] | CCATCTTTCCCCGCATCCGGATGCACGAGATGCGTC |
| 30707_11 | 12 | [C/T] | CATCCTTTAGACGCATCAGTCTGCACTGAAAAACAA |
| 30709_5 | 6 | [T/A] | TACTTTAACTGTGCAGCCACGTGCTCAAAAAGCAAG |
| 30710_5 | 6 | [T/C] | TGTCTTTCTGCTGCAACTTCCTGCCAGCTGCTCCCC |
| 30711_27 | 28 | [A/G] | AGTGATGGAAAGGCAACAGGATGCCGGAGACAGAAA |
| 30713_30 | 31 | [G/A] | CCTGTCTGGGGAGCAATAGATTGCCTTGCTGGGACC |
| 30716_27 | 28 | [G/A] | GGTGAAGAGGTGGCACAAAGCTGCTACGATCATCCA |
| 30718_26 | 27 | [C/T] | TAGACTTACAAAGCAGACCTGTGCTGCTGCTTCTGT |
| 30720_10 | 11 | [T/G] | TTTACAAACATGGCATGGTGGTGCAGCAGGTAATGT |
| 30739_31 | 32 | [T/A] | TGTTTAGAAAGAGCAAACCTCTGCAACCCAGTGTAA |
| 30740_20 | 21 | [A/C] | GGGGTGTTAAGCGCAAAACGATGCAGCTGCTGCAAT |
| 30741_7 | 8 | [T/C] | GCCATGCTTGTTGCACTACCTTGCTGGTCAGATAGA |
| 30745_25 | 26 | [C/G] | CATGGTCCTACAGCACCTATCTGCTCTTCTTCTGCA |
| 30753_19 | 20 | [T/C] | ACGGGCCTCTCCGCATCCTCCTGCGGGTCACTTTTA |
| 30765_11 | 12 | [C/T] | GCGCACTCGCCCGCAGCCTTCTGCGTCATGGAGCAG |
| 30768_3 | 4 | [A/G] | CCGATAAGCTCAGCATCACCGTGCACCACGAGTCCG |
| 3077_28 | 29 | [G/T] | CAAAATGGTGTGGCATAATGGTGCCACAGGTAGTGT |
| 30778_19 | 20 | [C/T] | GCGCTACCTACAGCACCACCGTGCCCTCTATTGTGA |
| 30788_27 | 28 | [C/A] | TGTAGCAGCACAGCATGTTCCTGCGATCAGGTTACT |
| 30798_30 | 31 | [C/A] | TCTCTTCGTAGAGCATTCTGGTGCCATGTACTGTAA |
| 30799_28 | 29 | [A/G] | AGGTACATTCACGCAGAACCATGCTGGGGTCCGAAT |
| 30801_30 | 31 | [C/T] | TGAAGTGTAAGTGCATGTTCATGCATCTGCCCTGTA |
| 30806_3 | 4 | [C/T] | AAACGTGTGCATGCATGCACGTGCTCTTGATGCAAT |
| 30811_5 | 6 | [C/G] | CATCCCCCTGCTGCATGCCCGTGCACTAAAGCTGTC |
| 30818_4 | 5 | [G/A] | AAGTAAACCAGTGCAAACATGTGCAAGAACAGGTCA |
| 30823_28 | 29 | [C/T] | AGATTTTCTCCTGCAGGTGTTTGCATGCCTGGACAA |
| 30835_17 | 18 | [G/A] | CTAGGAACACAGGCATGGTGTTGCTGCAGGTAGTCT |
| 30836_32 | 33 | [A/G] | GATAAAATGCACGCACACACATGCACATGTGCACAC |
| 30839_32 | 33 | [G/A] | AAAATGAACTGTGCACACTTTTGCCCTTTGACGTTG |
| 30841_31 | 32 | [G/A] | TAAATGTTGGTAGCACCAGTGTGCCAGATCAGATCT |
| 30842_15 | 16 | [T/G] | GGTGGGTTTGTGGCAGTAAGCTGCCTGATGAGAGCT |
| 30846_7 | 8 | [C/A] | TGTAAAACACTGGCACTGGTGTGCTGTGGCAGGAGA |
| 30850_19 | 20 | [C/T] | TTCTCCCAGAATGCATCGCCGTGCTGCGTTTCTCTC |
| 30851_1 | 2 | [C/T] | ACAGCGGAATCGGCAACTTCCTGCCCGAAGAAAAAA |
| 30853_17 | 18 | [C/T] | AGGCTTTAAAAAGCAGTCCCGTGCAGACCTCTACTC |
| 30855_33 | 34 | [C/T] | ACAGTGAGACGTGCATTCAAATGCACCACTTTGCCT |
| 30870_3 | 4 | [C/T] | GGACGTGTCCCTGCACCGGAATGCTGTTTCACTCCG |
| 30884_19 | 20 | [A/G] | AATGTATTTTGTGCAGTCCAGTGCAAAGGCGGAAAG |
| 30885_28 | 29 | [T/A] | AGGCTTGCTGTGGCATGTTCCTGCACCAAATTAAAG |
| 30888_34 | 35 | [G/T] | AATCATGTTCATGCATGACAGTGCTCCATCTCGTGC |
| 30889_20 | 21 | [G/A] | GACCAGGAGCTGGCAGTCACGTGCCACCCTAAGACA |
| 3089_29 | 30 | [G/C] | CAGTACTTATGGGCACTTATGTGCATGCTGCAGGAG |
| 30890_11 | 12 | [C/T] | ACTACGGCTCACGCAGAGCGCTGCGGGCTTAATTTA |
| 30892_11 | 12 | [C/T] | TAAATGGGTTATGCAGGTCTGTGCCCAGTGTTGCAG |
| 30895_17 | 18 | [T/A] | TGTTACATCACAGCAGCATCGTGCTTTAACTAACAC |
| 30896_5 | 6 | [G/A] | GGACCGATATAAGCAGTCACCTGCTCTGAGAGTGTA |
| 30897_1 | 2 | [A/T] | GATTAGACTAAAGCAGCATCCTGCAGGCTGTACAGT |
| 30898_27 | 28 | [C/G] | GGTCTTGCACACGCACTTTTATGCAAACGCTGACGA |
| 30903_33 | 34 | [A/G] | CATGCAAGACAAGCAACACGTTGCCAGAGTGGTAAA |
| 30906_19 | 20 | [T/C] | GAGAGATCCCGTGCAGACGTATGCGCAGCGGCAGCT |
| 30909_3 | 4 | [C/A] | AATCGACTAGTTGCACATTAGTGCCTCCCAACAGCA |
| 3091_2 | 3 | [C/T] | CTCAATCTGGGAGCACCTTTTTGCTTTCAACTGCCC |
| 30910_30 | 31 | [G/T] | TTATGGCATTTGGCAGAAATGTGCAGAAATGTGCAG |
| 30913_34 | 35 | [G/A] | TACACAATCTGGGCACCAACATGCATTTTAAACCGA |
| 30921_7 | 8 | [G/A] | TTTGGACGGAGTGCACCAGGTTGCGGATGCGCCCAG |
| 30933_4 | 5 | [G/A] | GCTCGTATGTACGCACCCACATGCACCCACACACAC |
| 30935_27 | 28 | [C/T] | AGACGAGTCTCTGCAGCTACATGCTTTCATTTCTGA |
| 30937_32 | 33 | [A/G] | GAGCCCTGGCGTGCAATCTGCTGCTGTTCCTCAGGA |
| 30943_32 | 33 | [C/T] | AATTCTGTACCTGCATTTGTATGCGCCAGCTTCATT |
| 30953_27 | 28 | [C/T] | GCTAGATCGGGAGCATGATGATGCCTTCCTCCATCT |
| 3096_28 | 29 | [C/A] | CACAAACCACATGCACTGTTGTGCCAAGCACTTTTG |
| 30961_19 | 20 | [C/T] | TGATCTCACCGGGCAGCAGCGTGCACTCACTCCCTG |
| 30967_7 | 8 | [A/G] | CTGTGACAGATTGCATGCCTTTGCATTGTGTCTTAA |
| 30969_10 | 11 | [C/T] | AGCTGAGGGACGGCATCGACGTGCGGCTCCTCCACT |
| 30977_27 | 28 | [C/T] | CAGCAGGACCAAGCAAGGCAGTGCCCTCATCTCCAC |
| 30981_17 | 18 | [G/A] | ACAGTCCGTGTTGCACCGAACTGCTTCGTAATGCCA |
| 30983_26 | 27 | [G/A] | TTGTGCTACACTGCACAGTCATGCACGCTTCTCTCT |
| 30985_16 | 17 | [A/G] | GAACCATGTCCTGCATAAAGATGCCCACGGGCTCGG |
| 30991_32 | 33 | [C/T] | GAAGAACAAACTGCACCCAGTTGCCTTTTTCTCCTG |
| 31014_10 | 11 | [A/C] | AATCTTCCAAATGCAGCCTGTTGCTGAGGGAGAAAT |
| 31015_33 | 34 | [T/G] | GCACTGGTGGTGGCAGCATTATGCTGTGGAGGTTTT |
| 31018_20 | 21 | [T/C] | ACAGCACCAGGAGCATCAATTTGCTGTCTGCAGACG |
| 31024_33 | 34 | [C/T] | TCCTGATTCCGTGCAGGCTTCTGCCCTCTGTGCCTT |
| 31026_8 | 9 | [T/C] | CCCTGTGGTGCTGCATTGGCCTGCTTCAGCGTTTCT |
| 31028_11 | 12 | [C/T] | ACTCTCAGACTCGCAGTACTGTGCCCTCATTCACTA |
| 31035_16 | 17 | [G/A] | CTCGAGTTCAGCGCAGGCTTATGCATACAGAGGAAG |
| 31036_15 | 16 | [C/T] | ATGCAAAGCCCCGCACGCTCTTGCCCTTTATACTCT |
| 31041_18 | 19 | [T/C] | AGAAGTTCCACAGCACAATGCTGCCAAGCTGCATAC |
| 31046_10 | 11 | [C/T] | ACAATGCCTGCGGCACCACTGTGCTGCCCTACTGAA |
| 31051_31 | 32 | [G/T] | CTCACCACAGCTGCATTGGGCTGCTGTTTCTGCCAG |
| 31054_33 | 34 | [C/T] | GGGGCTAGGATGGCAGTGTTCTGCAGCGTTAAACCC |
| 31056_16 | 17 | [C/T] | AAGCTCACAAAAGCAGCGGCCTGCCGGGACGACTTA |
| 3106_4 | 5 | [G/A] | TAGGGTGGGTGTGCATGCGCATGCAGGGTGGATGAC |
| 31060_29 | 30 | [C/T] | AAGGATCCTTTGGCATGACATTGCAGCTTCAGTGAT |
| 31061_10 | 11 | [A/G] | CACGCCCACGAAGCATAGAAATGCAAACACACACAC |
| 31067_29 | 30 | [A/T] | GCTCCCTGGTTGGCATAAGCTTGCCCACAACTCCAG |
| 31068_26 | 27 | [G/A] | CAAGAAGTCCTGGCAAACATTTGCCAGGCAGCCACT |
| 31073_11 | 12 | [T/C] | GACAGAGCTGCTGCAATGTGCTGCCACTAGGACAAG |
| 31075_7 | 8 | [A/G] | CCACATTAGCAAGCAATGGTCTGCAGCCAAGACTCA |
| 31078_28 | 29 | [A/G] | AGGAGAAGAAGCGCACAGGCTTGCGGCGAAAATAGC |
| 31080_31 | 32 | [G/A] | AAGTGCACTTGTGCAAACATGTGCAACACATGCATG |
| 31082_33 | 34 | [C/A] | AAGAGCATGGCTGCAGGAGTGTGCAGGTGCAGACCA |
| 31086_5 | 6 | [C/T] | AAGTTCCCTGACGCAACAGCGTGCTCTTCAAAAAGT |
| 31088_31 | 32 | [C/T] | TCCATCAGAAGGGCACATTCCTGCACATTATCACAC |
| 3109_7 | 8 | [T/C] | ACATGTTTGCATGCATGTTACTGCATGTACTCCCAA |
| 31090_32 | 33 | [G/C] | AATATTGAGGCAGCATGGCAGTGCCTCAGGTAGTGT |
| 31097_31 | 32 | [C/T] | TCTACTGTCCATGCATCAAAGTGCGTCTCAGCTGGG |
| 31100_7 | 8 | [C/T] | AAATGCTCTGAAGCAGACGCATGCAGTCGCATTTAT |
| 31101_4 | 5 | [T/C] | AAACTTGCCACAGCACCCCGTTGCTATTTAAAGGAT |
| 31105_28 | 29 | [G/C] | ATCAGCGGAGCTGCAGATTTCTGCTAACGTAGTATT |
| 31108_24 | 25 | [A/G] | GTTGAGCCTGCTGCAGCCGGTTGCACCCGTTCTCCT |
| 31110_29 | 30 | [A/C] | TCTGCCCGCTCTGCAAATGACTGCAACTCAGTCTAA |
| 31113_30 | 31 | [G/A] | GGTGATGATGGCGCAAATGTTTGCCGAGCCGCTTTA |
| 31114_4 | 5 | [C/T] | TCACCTCCTGCCGCAACTCCCTGCACAGATTCACAC |
| 31119_7 | 8 | [A/T] | AATCGTGAAGCAGCATCACAATGCACAACGAGATTC |
| 31120_5 | 6 | [G/A] | TCAGAGATAAATGCATATTCATGCGGCGTCGCTGGG |
| 31121_24 | 25 | [G/A] | TTTGTCGAAGACGCACAATAATGCGAACCCGCAGGC |
| 31123_25 | 26 | [A/G] | GCAGCACACAGAGCAGCTACATGCCAGATAAAGTCT |
| 31124_15 | 16 | [C/T] | AATTAATTCCCCGCATGACATTGCGTGTGCAATCAA |
| 31126_34 | 35 | [G/A] | CGGGCTTTTCATGCACTTTGGTGCAGCAGCTAGAGT |
| 31129_5 | 6 | [T/A] | ACTTTTGCAAGGGCAGCTCGTTGCTTACGGCACCCC |
| 3113_32 | 33 | [C/T] | AGCAATCTTACAGCAGAAGTCTGCAGAGAGCTCCCA |
| 31134_20 | 21 | [A/C] | TGGAATCAGCTGGCAGAACGCTGCCCAAAGTCAGAG |
| 3114_31 | 32 | [T/C] | CGCTCCTGTGCCGCATGCTAATGCGCAATCACGTAG |
| 31142_31 | 32 | [C/T] | TTCGCACGTCCAGCATCAGAATGCAGGTGCACACGT |
| 31143_27 | 28 | [C/T] | CTCTCTGGTGGTGCACAATGATGCACACATGGTGGA |
| 31144_19 | 20 | [G/A] | CGCCGAAAGGACGCATCACGGTGCAGTTTTACGAGA |
| 31145_11 | 12 | [T/C] | CACCACAAGCGTGCACAAATATGCAAAACCGTCACG |
| 31150_20 | 21 | [G/A] | GGGATGTAGGTCGCATTGTTGTGCGTGATAAAAGAG |
| 31153_34 | 35 | [G/A] | GGGCAGATACTGGCACTGGTGTGCGGCAGTCGTAGA |
| 31157_2 | 3 | [G/A] | TTGAAGCATAAAGCACAGTGATGCTAAAGCCTCTGT |
| 31163_8 | 9 | [G/A] | GCGGAGACGAGTGCAGAAGCGTGCGGAGATAACGCT |
| 31167_28 | 29 | [C/G] | CTGTGACATCAGGCAGCATCTTGCCATCCCAGTGTA |
| 31168_26 | 27 | [T/A] | CAGTGCAGCATAGCAAACAGTTGCTCTCGGGTGTAA |
| 31171_30 | 31 | [T/C] | GGCGTTTCTAGGGCACTCGTATGCTTCTGATGAATA |
| 31175_18 | 19 | [A/C] | CCACATACACAGGCATTCACTTGCACAATCACCCCT |
| 31176_11 | 12 | [G/A] | TCGAAGCACCTGGCAGAAACCTGCACAAACACAGGG |
| 31187_30 | 31 | [C/T] | GCTCACGTCTGTGCAATTCCCTGCCTGCCTTCACCT |
| 31193_1 | 2 | [G/T] | TGGCTCTAAACAGCAGTGTCTTGCTTGATTACGGCA |
| 31196_33 | 34 | [T/G] | TTTTAAATGCTAGCACATGTCTGCGTAGGCCATTTG |
| 31200_19 | 20 | [A/G] | GATCTCTCGCTGGCACTGCAGTGCATTGTCATTTAT |
| 31209_33 | 34 | [G/A] | TCCAGCAGAGCTGCACAACGTTGCTTCGGTAGAGAC |
| 31210_4 | 5 | [C/G] | GCCCCCGGACCGGCAAGTCTTTGCCTCCAGCCCCAG |
| 31211_31 | 32 | [C/T] | AAACATGATTAGGCACGTTAGTGCGTGAGCTCTCCA |
| 31214_18 | 19 | [A/G] | GGAGAGAAGGAAGCAGTCAGCTGCAAATGTGCCATT |
| 31217_1 | 2 | [C/G] | TCGCAGCTTGTGGCAGTGATCTGCTGGGGATTTGAT |
| 31219_27 | 28 | [A/G] | GTAGCGTAGTCAGCAGAAACCTGCGCCAGTCAAGTC |
| 31220_3 | 4 | [A/G] | TGAAGAGAATACGCACAGGCCTGCCAAATATCACAG |
| 31228_28 | 29 | [C/T] | AATGGCTTTTCTGCATTGGTGTGCGTTGCTGTCCCC |
| 31231_8 | 9 | [C/T] | ATGCTTTTCAATGCATTACGCTGCATGCAGATCACA |
| 31232_3 | 4 | [C/T] | GTCCGGCAGTGTGCACATTTGTGCTGCTTCTAAGAT |
| 31233_1 | 2 | [A/G] | TAAACCCTCTGTGCAGCCTCGTGCCCCATCCCCAGA |
| 31234_33 | 34 | [C/T] | CAGACGAGCCGGGCAGATCTCTGCGCTGTCAGACGT |
| 31242_28 | 29 | [G/T] | GCTAACTCGCGTGCACGTGCGTGCCGCCGGTCTCCT |
| 31243_25 | 26 | [G/T] | ACGGTTATGGTGGCAGCCTGGTGCAGAAGTTTGTGT |
| 3125_19 | 20 | [C/T] | GGAACATTGTCAGCAGAAACGTGCATGCTTGCAGCT |
| 31251_33 | 34 | [G/A] | TAGTTATTTGATGCAGCATGGTGCAGTCTTCCAGGG |
| 31252_1 | 2 | [T/C] | ATACAGTCACAAGCAGCGATCTGCAGGTTCAAGGAC |
| 31257_29 | 30 | [A/T] | GTCTGCACGTTCGCAGTCAGGTGCATTGGAGAACGG |
| 31262_28 | 29 | [C/T] | TCCTGGTTCCCAGCATGCACCTGCACCACGCCGTGG |
| 31266_32 | 33 | [C/T] | CATGTTTTCTTCGCACGTTGATGCGGTTCGGTCACT |
| 31269_31 | 32 | [C/T] | CAGCTGTGAAGGGCATCATGCTGCACCGAGTCATCT |
| 31273_32 | 33 | [A/T] | AGGACGTGCTCAGCACCTCTGTGCGACCATCCAATA |
| 31279_3 | 4 | [C/T] | TCCCAAGACCACGCACTAACTTGCCTGGATAAACAC |
| 31281_27 | 28 | [A/G] | AAACTACATGTAGCACCACTGTGCCACGTCCACTTT |
| 31283_20 | 21 | [T/C] | TTCCTGAGCCTGGCATCAGTTTGCTGACGGCTTATG |
| 31293_11 | 12 | [A/G] | ATGCCGAAACAAGCAACGCAATGCAGATTTGACATT |
| 3130_34 | 35 | [A/G] | GAGAAACCCAATGCAACCACATGCAAAAACTCAGAA |
| 31303_31 | 32 | [T/C] | GTATGCTGCGCTGCACTGTCGTGCGTCAAGCTCCCG |
| 31304_34 | 35 | [C/A] | ATGAGAGGGTTTGCAAATTTCTGCAACCTGGTTTCT |
| 31310_16 | 17 | [C/T] | ATTTCCCCGAGCGCAACGTGCTGCTTCTTTCATAAC |
| 31316_19 | 20 | [A/C] | CCACTAGCAGCAGCATCGGAGTGCCGGTCCTTTTAA |
| 31322_8 | 9 | [G/A] | ATGAGCAAGGGTGCAAATTGCTGCTATGGGCTTATC |
| 31323_19 | 20 | [T/C] | CTCCTCCAAGCAGCATTTTTCTGCCAAACAGTTCAA |
| 31324_30 | 31 | [G/T] | AACCCGGCTCTGGCACCCTGCTGCATAATGGATGAC |
| 31325_19 | 20 | [T/C] | TAAACACCTACAGCACAAGTGTGCTGACATTCGCAG |
| 31326_31 | 32 | [A/T] | TGGACTATGTGGGCATGAGTTTGCAGCACTTAAGAA |
| 31337_26 | 27 | [C/T] | ACACACCACCTGGCATCCGCTTGCCTCGTATGCGTA |
| 31344_5 | 6 | [G/A] | GATCCGGATGTAGCACCTGACTGCATTCCAAAAGTG |
| 31345_11 | 12 | [C/A] | GGTCTTTTCTTCGCAGCGCTTTGCGTTTTTTGGCAG |
| 31359_32 | 33 | [A/T] | ATCAAAGACTCAGCAAAATGCTGCGTGTTTCTAATA |
| 3136_32 | 33 | [G/A] | GCTCCATGTACCGCATCCATATGCAAGTGTCCACGT |
| 31363_32 | 33 | [A/G] | GAGATCTTCATGGCATTCGTTTGCGTGATCTTAAAG |
| 31365_10 | 11 | [G/A] | AAGTAAATTGGAGCAGGTGCGTGCCCTGCTGTCAAC |
| 31367_1 | 2 | [G/A] | TGATGTGATGGGGCAAAGTATTGCCCATCTACCCAC |
| 31370_9 | 10 | [G/A] | GTTTACTTCGGTGCAAGAGGTTGCATGGTACTCATA |
| 31372_34 | 35 | [C/T] | TGGTGTTGGAACGCAGTAATATGCGGGGTGAATCCA |
| 31379_11 | 12 | [C/T] | CGCTTGGCTAACGCAGACACATGCACTGTGAATCAA |
| 31383_28 | 29 | [T/C] | GTCCCTGCTAAAGCACTGATCTGCTGGATTCTATCA |
| 31385_29 | 30 | [G/C] | CAGGAGTTCTGAGCAGTACAATGCTCTGTGTTTGGA |
| 31388_27 | 28 | [G/A] | ATGGAAACCAAAGCAAAGGCGTGCTGAGATATCCAT |
| 31389_28 | 29 | [C/T] | GATCTTCGTGTTGCACCTTTTTGCTCACCAGGTGCA |
| 31392_9 | 10 | [T/C] | GTGTGACAGTCTGCAGACATTTGCAGTGCCTGCTTA |
| 31394_34 | 35 | [G/A] | GAGCTGACGGATGCAGTCATGTGCCGGGGTCTCCGG |
| 31397_9 | 10 | [C/T] | TATTGAACTCCTGCACTTGTGTGCTAGCCTGTTTTG |
| 31398_29 | 30 | [G/A] | GTTAGGAAGGTCGCAGATCTCTGCTGAATGATATGA |
| 31401_28 | 29 | [A/G] | TGAAATAGAAGAGCACCGTCCTGCTCCCACTAAGGT |
| 31403_1 | 2 | [G/A] | GGATCTCCAATCGCACCGCAGTGCACCAGCCCTAGC |
| 31409_15 | 16 | [G/A] | ACAGGATGAACTGCAGCGTGCTGCAGACGAGCAGCG |
| 31413_9 | 10 | [A/G] | TGGGTGTTTAGTGCACTCTTCTGCATCATTTCCTCT |
| 31416_27 | 28 | [A/C] | GAGCGTCTGACAGCAGTTAATTGCGACAGACATAAA |
| 3142_2 | 3 | [C/T] | TTCGGTTACCTTGCACATTTCTGCAGATAGACCCTC |
| 31421_11 | 12 | [C/T] | ACACATGCATACGCAGTTCAGTGCTTTCTGCACACC |
| 31425_18 | 19 | [T/C] | TCGTCTGTTATTGCATGGTTGTGCATCATCAGTGTT |
| 31427_18 | 19 | [A/G] | CCCTCCAGGTGTGCATGGACCTGCAAACAAAGAGTC |
| 31432_9 | 10 | [T/A] | GTGTGTGTGTTCGCACTCCAGTGCAATCAGACGTGC |
| 31437_7 | 8 | [A/C] | GCTGCAGAGCTTGCATTACAGTGCCAGAGGTCACAG |
| 31445_30 | 31 | [A/G] | GGAGGGGAAGTAGCAGGGGCATGCTGCATCACACAC |
| 31465_26 | 27 | [C/G] | CATACCCCAGGGGCAGATTACTGCAGCCTGGGGGCC |
| 31469_19 | 20 | [G/T] | TGGAGCATCTGGGCAACCTTCTGCAGGCCAGCGCTC |
| 31485_33 | 34 | [T/C] | CTCGTTTTGCATGCAGTTTGTTGCGTGTGGGCGTGC |
| 31486_10 | 11 | [G/A] | TAATCCCACTGAGCATTCCGATGCCGTAAGTGGAAA |
| 31488_7 | 8 | [G/A] | GTCACTGGTTAAGCAAACTTGTGCCAATGGTGTCCA |
| 31489_10 | 11 | [C/T] | GGTGCATAAACGGCAGTAAGGTGCTGAACTGTGGTA |
| 31490_34 | 35 | [G/A] | GGAGGTGTTAAAGCAGAAACATGCAGGGCGGTGCGC |
| 31495_29 | 30 | [A/G] | GGGCGAGAAACTGCATCAATCTGCCATGGAAGCACA |
| 31496_17 | 18 | [G/A] | GGAGTGGACATGGCATTGAATTGCGCACACGAGCAG |
| 31497_6 | 7 | [C/T] | GAACAGCCAGTGGCACAACAATGCAGCAGGTAACAT |
| 31505_10 | 11 | [C/A] | GGTGCAGCCACTGCACTCCTGTGCTCGTGTGTCATA |
| 3151_4 | 5 | [T/A] | TGTTTTATGTGTGCAGGATGCTGCTAATCCTGCAGA |
| 31514_24 | 25 | [A/C] | GATTGCACTAATGCATCCATGTGCATTCATGAGCCT |
| 31517_7 | 8 | [C/G] | TTCCACACAGTTGCAGTAGAGTGCAGAGGTCTGGTT |
| 31522_20 | 21 | [G/A] | GTGCTGTTGGCAGCAGCAGTGTGCACCAAGGCTGGA |
| 31525_32 | 33 | [C/T] | TCGTTGGCATCAGCAAAGTATTGCTGGGCCTGCAAG |
| 31529_30 | 31 | [C/T] | AAAAAAGTAAACGCAGCATGATGCCTTGTCCCTGTT |
| 31530_34 | 35 | [A/G] | TTCCAAAACAATGCAGGCCTGTGCCTGAAACCTGAG |
| 31531_2 | 3 | [C/G] | GGCGAGTGTGCAGCATCACAATGCCCTTTAGAAATT |
| 31537_20 | 21 | [C/A] | GAACACAAACACGCAGGCGGATGCAGGAACCGTGAT |
| 31550_34 | 35 | [A/C] | ACACCGGTTACAGCAGCTGTGTGCAAATGGTTTTAC |
| 31554_1 | 2 | [T/G] | TTCACAGACATGGCATTTATTTGCTCAGGTTGCTCT |
| 31559_6 | 7 | [T/C] | CCTTAATCTCCAGCACACAGCTGCCTCACACCATAC |
| 31560_5 | 6 | [A/C] | GTACCAGCAATGGCAATCCAATGCTGATCATTTGAT |
| 31562_34 | 35 | [C/T] | GCTCGAGCCTGTGCACGTGCGTGCTCTGATTGGGCG |
| 31565_26 | 27 | [A/G] | ATTGAGTGCCTGGCACCACAGTGCCAAAGAAAGACT |
| 31569_17 | 18 | [G/A] | AGCGATGTATTTGCAGTGAAGTGCAACTCACACATA |
| 31579_29 | 30 | [G/A] | GAGCAGTGCTAAGCATCAGGTTGCCAGATGCTCTAT |
| 3158_26 | 27 | [T/G] | CAGGCAGGCCAGGCAGGCACATGCACTCTACGTCTA |
| 31581_18 | 19 | [T/G] | TGCAAGAAACTGGCATGATGGTGCACGAGAGCATCT |
| 31588_4 | 5 | [T/G] | AAGTTCCTCGTGGCAAATATCTGCGAGCTGATCAGC |
| 31589_11 | 12 | [C/T] | CAGCTTTCCGCCGCACTAACTTGCCTCATAAACACT |
| 31598_6 | 7 | [C/T] | CCTGTACGCCCAGCAGCTCGCTGCTCAGCACGCCAT |
| 31616_4 | 5 | [G/A] | ACCTGAAGAGCCGCACTGAATTGCAGCTGCTGCACG |
| 31622_29 | 30 | [C/T] | TATCTAAGATTGGCAGGTTGCTGCAAGCGCGCAATT |
| 31623_28 | 29 | [C/T] | TATCCTTTATTTGCAGACTGCTGCCATTCACACCAC |
| 31624_8 | 9 | [G/A] | CGTTATCAGGCAGCACGCTGGTGCATATATTACCTA |
| 31625_18 | 19 | [C/A] | GTTAGACTGGAGGCAACTCTGTGCTGGGATTTATGA |
| 31627_31 | 32 | [A/G] | GACAGATACAAGGCATGGTGGTGCAGTACACGCCTA |
| 31629_24 | 25 | [T/C] | GATCTCCTTCTCGCAATTCAATGCTGTAGACGTGTT |
| 31632_29 | 30 | [G/A] | CACTGGTATCGTGCACTGGAGTGCTGTGTGAGGTGA |
| 31634_17 | 18 | [C/T] | ACACCTGTTTTGGCAGTCTTGTGCCTAGTAAGTGTA |
| 31641_24 | 25 | [C/T] | TCCCACACAGTTGCACACAACTGCCAAGCTGCCTTG |
| 31643_19 | 20 | [C/G] | TCAGAGACACTGGCACTAGCGTGCCTCTTCAGTGTA |
| 31648_27 | 28 | [C/A] | ACACACACACACGCACTGTCATGCCAGCTTTCTTCT |
| 31653_16 | 17 | [G/A] | GAATTGGAATCGGCACGTTACTGCCTGCACAGCTTC |
| 31654_15 | 16 | [G/A] | CACAGCATCCAGGCAGCCGTTTGCTCAAGGGCACTT |
| 31659_26 | 27 | [G/A] | ATTAAACCTACAGCACACCAGTGCACGAGTCGAGGA |
| 31662_2 | 3 | [G/T] | GTGTACAGGCCGGCACCTACATGCCACATTCGTACA |
| 31664_26 | 27 | [T/G] | CTCTGACAGCCTGCAACAGTCTGCAATTCTTTGCAT |
| 31665_16 | 17 | [C/T] | ACACTTCCTGCAGCACCAACATGCCACTGTTATTGT |
| 31670_31 | 32 | [T/C] | TATTTGACAGTGGCAACCTTATGCCCCTGTCTGATG |
| 31672_4 | 5 | [G/T] | GCCAGTGAACCGGCAGAGGAATGCCCCCGGAGCAGG |
| 31681_18 | 19 | [G/T] | TCCTCACTGTCCGCACACGGTTGCTCTCTGTGGATA |
| 31682_4 | 5 | [T/C] | GTCTTTCTACCCGCACAAATCTGCCCATCAGGCTTT |
| 31683_18 | 19 | [G/A] | AAGCCACAGTAAGCAGATGAGTGCTTATTCCACACT |
| 31687_34 | 35 | [G/A] | CACATACCTGTGGCACCACTGTGCTGCCTTGGCTGA |
| 31690_10 | 11 | [T/A] | GAATATTTTGAAGCAGCGGTGTGCCTAATGTGCATG |
| 31693_10 | 11 | [C/T] | TTTGGATTTTCTGCACCACACTGCACACTTTGCACA |
| 31697_3 | 4 | [C/T] | CAGCTCGCTTCAGCAAAATCCTGCCACTGTCTTTAA |
| 31701_33 | 34 | [C/T] | TCGATTTGTTGTGCAGACACATGCACTGACGCACAC |
| 31702_10 | 11 | [C/T] | TGATCTACGACGGCAACCCAGTGCCGGGGAGCCCGT |
| 31704_20 | 21 | [G/C] | AATCACTACTACGCACCACAGTGCAACGTGGCCAGT |
| 31706_29 | 30 | [A/G] | CCAGAGCGACACGCACTGCAGTGCATCACATTGTGT |
| 31716_30 | 31 | [A/C] | TAATGTGCTTGTGCACTATAATGCTGGGCTAGATGC |
| 31718_18 | 19 | [G/T] | CACCTCTGCGAAGCAAAAGCATGCCAGGACATTGCC |
| 3172_24 | 25 | [T/C] | TCTGGGGAAGACGCAGACGGCTGCCGTGCAGAGGGA |
| 31721_25 | 26 | [C/T] | CCAAGTCCATCAGCAAACACTTGCTCCGTGATGGAC |
| 31722_32 | 33 | [G/A] | GTGGCATATGAGGCACTAAAATGCAGTTGAGGGTTT |
| 31728_32 | 33 | [A/C] | CTTTGTACTCTTGCATCCTCTTGCAACTCTGAACCC |
| 31733_33 | 34 | [C/T] | TTTTCCAGTCTTGCATCCTGGTGCCATGTCTTCCCT |
| 31740_30 | 31 | [T/G] | GTGTACGTGTGTGCATGTGTTTGCGTGTGCTCATGT |
| 31741_3 | 4 | [C/T] | TAGCGACAACAAGCACAACACTGCCAAAATTGGTCG |
| 31744_2 | 3 | [A/T] | GTACGAGGAGCTGCAGAGCTCTGCTGGAAACTATGG |
| 31747_11 | 12 | [C/T] | TGTCCTCAGTACGCAGAGGTTTGCGGTGCTCTGGGC |
| 31752_17 | 18 | [T/C] | AAGGATCAGGTTGCAAATACATGCCAAGCAAATCAG |
| 31762_15 | 16 | [C/G] | GTGCACAAGGCAGCACCCCAATGCCAACACAGGTCA |
| 31764_19 | 20 | [G/A] | AAGTGATGGATGGCAGCCCGCTGCTGCAGGAGAGTG |
| 31766_5 | 6 | [A/G] | TCACAATGGGCAGCACAATGGTGCCACAGGTAGGGT |
| 31769_31 | 32 | [C/T] | AAAAAAATCTGTGCACGCTACTGCTGAACTTCCCAT |
| 3177_31 | 32 | [C/A] | ACTAAGGGGATGGCATGATGGTGCCAAGCCAAGGGT |
| 3178_2 | 3 | [A/G] | TCATACCAGGAGGCAATTTAGTGCATCCAGTTCACC |
| 31785_10 | 11 | [G/A] | GCTGCAGTGCGTGCAGGGCGTTGCCTGCGAGCCCAA |
| 31787_33 | 34 | [G/A] | AAAGGAGTTGATGCATGGTGCTGCCTTTAAGCCGCT |
| 31788_2 | 3 | [G/T] | GCGTTGGCCGTGGCAAGAACCTGCTGGGAGGAAACA |
| 31797_1 | 2 | [T/C] | ATCTCTGTTTGAGCAGTAGCCTGCAACAGCTCTGAC |
| 31800_2 | 3 | [G/T] | AGGTAACCAGGTGCAAAATGTTGCTGAGGTTTACAC |
| 31803_30 | 31 | [G/A] | CTGTGGCCGACAGCAGGTCCGTGCAACTCTGAAACC |
| 31805_7 | 8 | [G/A] | GGACTACGGTGAGCAGCGCACTGCTGACCCTGACAA |
| 31807_34 | 35 | [C/T] | ACCAGTCAGAGTGCAGAACAGTGCCAACCACCTCCG |
| 31813_3 | 4 | [C/T] | CATCAGCGGCGTGCAGTTTAGTGCCTTTTCTCTCCT |
| 31814_27 | 28 | [T/C] | GGAAAGAGCATGGCACAAAAATGCCTCTGACGACAG |
| 31818_9 | 10 | [A/G] | TAAGACCCGACTGCAACTGGATGCCAGGTATGTAGT |
| 31819_18 | 19 | [A/C] | GCACTACCTGTGGCACCAATGTGCAGCCCAAACTGA |
| 3182_9 | 10 | [C/T] | TTCTGACATCCAGCAGCTTACTGCCGTTTCTCTTCT |
| 31822_32 | 33 | [G/A] | GCCATGGTAGGGGCAAGGGGTTGCGTTGCGACGGGG |
| 31824_34 | 35 | [G/A] | TGGCCCAGTGTGGCAAAAAGGTGCTGTGCCATGCGT |
| 31826_18 | 19 | [C/T] | GTGTGCTGTTGTGCACGGCTGTGCAGAGGTAATGCT |
| 31827_16 | 17 | [C/T] | CATGGAGACCACGCACCGGAGTGCGTTGGCCGAGAG |
| 3183_2 | 3 | [G/A] | ATGGATGTTCTAGCATGACAATGCCCATGTTTACAG |
| 31832_30 | 31 | [A/T] | ACACATGGCTTTGCAATGCCCTGCTGGCACAATTAA |
| 31835_33 | 34 | [A/C] | CTATCTGCCCTTGCAGAAGAATGCAGCATAACCATG |
| 31839_25 | 26 | [G/A] | GGGTTGTGGGTGGCACATTGGTGCCGCAGGTAGTGT |
| 31842_3 | 4 | [T/C] | GCTTTGGCTGCAGCAGCATCTTGCTCAGCTTTAGCT |
| 31843_29 | 30 | [A/G] | GACAAGACCAGGGCATGGTGATGCCGGCTAGAGTGA |
| 31856_9 | 10 | [C/T] | CGGTTTGAGCGAGCATTACTCTGCTCTGAGTATGAC |
| 31857_27 | 28 | [A/G] | TCTTTCACACCAGCAGCTCAGTGCACGAAAGCATGT |
| 31858_2 | 3 | [T/G] | TTTGGAGTTTGAGCACATTTTTGCAGTTCCCAATGC |
| 31860_16 | 17 | [G/A] | TCAGGCCCAAATGCAGGATTTTGCCAGAGAGGAGTA |
| 31861_27 | 28 | [T/C] | CCGGTTCTTCCTGCAATAGGATGCCAGTGTTGCAAC |
| 31862_34 | 35 | [A/G] | GCATTTGGGAAGGCAATTAGTTGCACCTGCATCGAT |
| 31864_6 | 7 | [G/A] | TTGCTTGATGAGGCAGAAAAGTGCAGTGTGTCTGTG |
| 31867_11 | 12 | [C/T] | GCGTGCCAAGGCGCAATGTCATGCGTAGGAGTGGCA |
| 31869_27 | 28 | [C/T] | ATTGCAAAAAAAGCAGTTTGTTGCTCTCGATCGTGG |
| 31870_30 | 31 | [C/T] | TCACTATCTGTTGCAGCGACTTGCAGTGATCTGACA |
[truncated: 728,854 more chars]
