# Supplementary material for: Population Genomics in Rhamdia quelen (Heptapteridae, Siluriformes) Reveals Deep Divergence and Adaptation in the Neotropical Region
Source: Genes (Basel). 2020 Jan 17;11(1):109. doi: 10.3390/genes11010109 (PMC7017130; doi:10.3390/genes11010109)
Supplement: Supplementary file 1 [file genes-11-00109-s001.zip › Supplementary File SVIII.docx]

**Table SVIII 1.** Annotation of the *Rhamdia quelen* Outlier dataset that results from comparing northern and southern basins.

| SNP code | Homologous sequence and E-value | Gene | Biological process/Function (GO terms) | | Chromosome *I. punctatus* and position |
| --- | --- | --- | --- | --- | --- |
| 33517_8 | XM_026914278.1 | spaca6 | No data in Blast2go | | Chr 1: 6,713,266-6,713,453 |
|  | 2 e^-4^ |  |  |  |  |
| 6113_20 | Genome *I. punctatus* | ascc3 | No data in Blast2go | | Chr 1: 18,860,777-18,860,806 |
|  | 3 e^-6^ |  |  |  |  |
| 8232_7 | Genome *I. punctatus* | Unanotated |  | | Chr 2: 16,840,611-16,840,634 |
|  | 5 e^-5^ | sequence |  |  |  |
| 66673_9 | Genome *I. punctatus* | CSMD1 | Homeostatic process; Nervous system process | | Chr 3: 28,942,449-28,942,484 |
|  | 2 e^-7^ |  |  |  |  |
| 64979_10 | Genome *I. punctatus* | Unanotated |  |  | Chr 5: 13,660,451-13,660,486 |
|  | 9 e^-10^ | sequence |  |  |  |
| 15866_28 | Genome *I. punctatus* | ralgps1 | Small GTPase mediated signal transduction; regulation of Ral protein signal transduction | | Chr 5: 13,807,353-13,807,388 |
|  | 5 e^-5^ |  |  |  |  |
| 66502_32 | Genome *I. punctatus* | hoxd3a | Anterior/posterior pattern specification; positive regulation of transcription by RNA polymerase II; embryonic skeletal system morphogenesis | | Chr 6: 9,429,548-9,429,581 |
|  | 1 e^-8^ |  |  |  |  |
| 16532_28 | Genome *I. punctatus* | Unanotated |  | | Chr 6: 14,560,728-14,560,755 |
|  | 2 e^-7^ | sequence |  |  |  |
| 68330_15 | Genome *I. punctatus* | Unanotated |  | | Chr 7: 13,834,719-13,834,754 |
|  | 2 e^-7^ | sequence |  |  |  |
| 49857_34 | Genome *I. punctatus* | kdrl | No data in Blast2go | | Chr 8: 7,712,981-7,713,013 |
|  | 2 e^-10^ |  |  |  |  |
| 9230_26 | XM_027135514.1 | chic1 | No data in Blast2go | | Chr 8*: 7,757,023-7,757,984* |
|  | 7 e^-5^ |  |  |  |  |
| 449_34 | XM_027165565.1 | fbxw2 | No data in Blast2go | | Chr 8: 8,029,587-8,031,148 |
|  | 4 e^-7^ |  |  |  |  |
| 71932_15 | Genome *I. punctatus* | vti1a | No data in Blast2go | | Chr 13: 13,766,786-13,766,822 |
|  | 2 e^-7^ |  |  |  |  |
| 47398_20 | Genome *I. punctatus* | Unanotated |  | | Chr 14: 19,002,180-19,002,206 |
|  | 2 e^-4^ | sequence |  |  |  |
| 27081_18 | Genome *I. punctatus* | ubr4 | Ion binding | | Chr 15: 15,739,029-15,739,064 |
|  | 4 e^-12^ |  |  |  |  |
| 29877_2 | Genome *I. punctatus* | camta1a | Biosynthetic process; cellular nitrogen compound metabolic process | | Chr 15: 18,120,420-18,120,452 |
|  | 5 e^-8^ |  |  |  |  |
| 67564_7 | XM_026921172.1 | scn8a |  | | [Chr 15: 19,737,353-19,737,386](https://www.ensembl.org/Ictalurus_punctatus/Location/View?r=15:19737351-19737387;tl=M2XPvxIM58aEzu03-5640152-748629204) |
|  | 6 e-6 |  |  |  |  |
| 53712_15 | Genome *I. punctatus* | polm | No data in Blast2go | | Chr 16: 20,362,022-20,362,044 |
|  | 2 e^-4^ |  |  |  |  |
| 18670_31 | Genome *I. punctatus* | Unanotated |  | | Chr 18: 1,036,051-1,036,086 |
|  | 2 e^-7^ | sequence |  |  |  |
| 48262_4 | Genome *I. punctatus* | Unanotated |  | | Chr 20: 9,580,304-9,580,339 |
|  | 9 e^-10^ | sequence |  |  |  |
| 66950_20 | Genome *I. punctatus* | gpc5c | Signal transduction | | Chr 20: 12,911,278-12,911,305 |
|  | 5 e^-5^ |  |  |  |  |

SNP: SNP locus code; Homologous sequence and E-value: Genome of *I*. *punctatus* or GenBank accession number of homologous sequences and E-value: value result of blast with sequence homologue; Gene: gene name to which RAD-tag is homologous; Biological process/Function: biological process or function of Gene Ontology terms (GO) to which “Gene” is classified; “Chromosome *I. punctatus*” and “Chromosome position”: Chromosome of *I. punctatus* and position where the RAD-tag is anchored.
