# Supplementary material for: Population Genomics in Rhamdia quelen (Heptapteridae, Siluriformes) Reveals Deep Divergence and Adaptation in the Neotropical Region
Source: Genes (Basel). 2020 Jan 17;11(1):109. doi: 10.3390/genes11010109 (PMC7017130; doi:10.3390/genes11010109)
Supplement: Supplementary file 1 [file genes-11-00109-s001.zip › Supplementary file SX.docx]

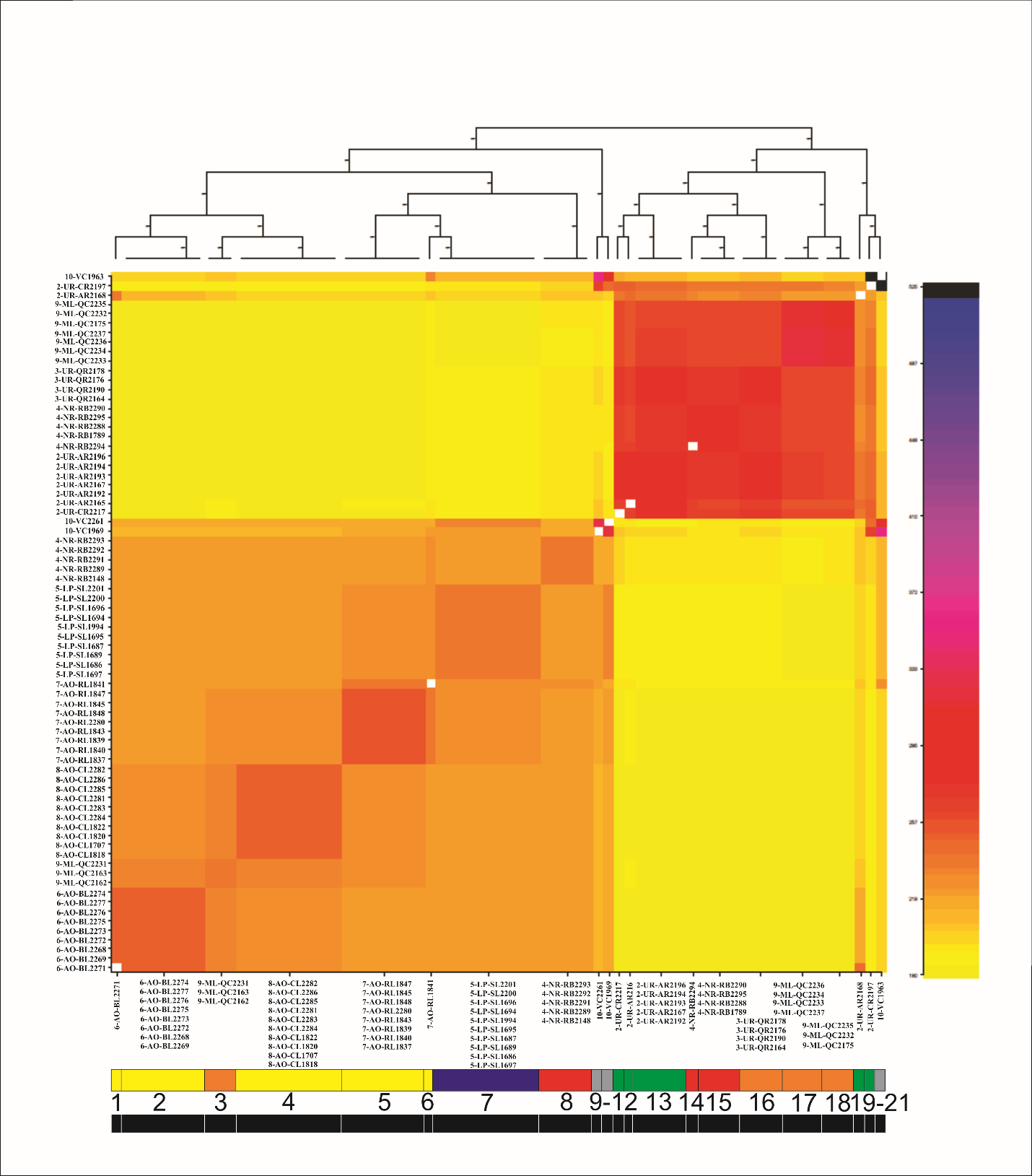


Figure S 1. *Rhamdia quelen* fineRADstructure plot based on Neutral *loci*. Horizontal axis shows clusters of individuals and vertical axis shows each individual. Pairwise coefficients of co-ancestry are color coded from low (yellow) to high (black). The dendrogram shows a clustering of individual samples based on the pairwise matrix of co-ancestry coefficients. Legends on the left and below the co-ancestry matrix show the name of each individual, and their corresponding basins are colored according to the code used in Figure 1 (Uruguay River basin: green; Negro River basin: red; La Plata River basin: blue; coastal SW Atlantic Ocean basin: yellow; Merin lagoon: orange). Numbers along the bottom line indicate the different minor clusters detected.


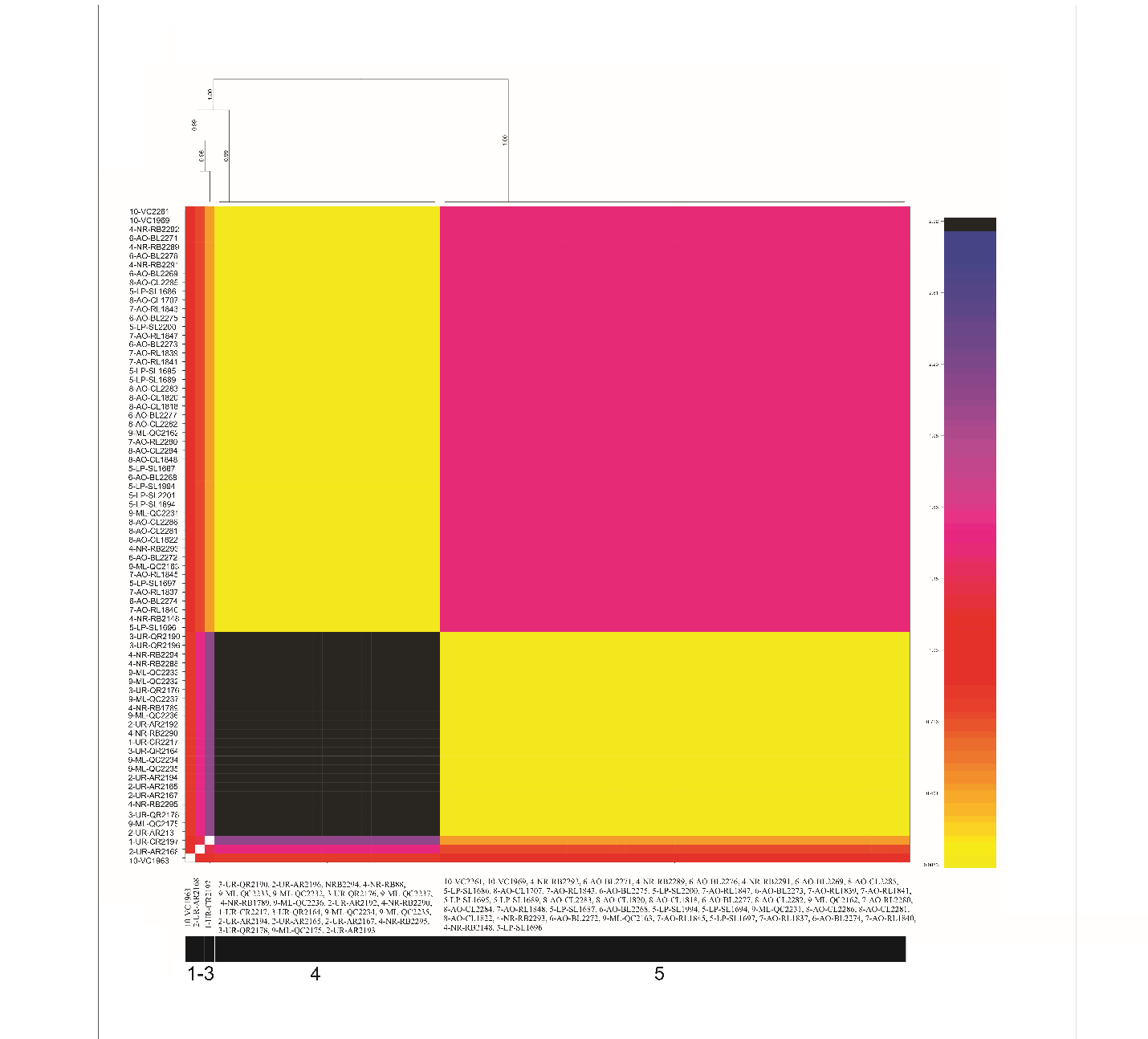


Figure S 2. *Rhamdia quelen* fineRADstructure plot based on Outlier *loci*. Horizontal axis shows clusters of individuals and vertical axis shows each individual. Pairwise coefficients of co-ancestry are color coded from low (yellow) to high (black). The dendrogram shows a clustering of individual samples based on the pairwise matrix of co-ancestry coefficients. Legends on the left and below the co-ancestry matrix show the name of each individual, and their corresponding basins are colored according to the code used in Figure 1 (Uruguay River basin: green; Negro River basin: red; La Plata River basin: blue; coastal SW Atlantic Ocean basin: yellow; Merin lagoon: orange).


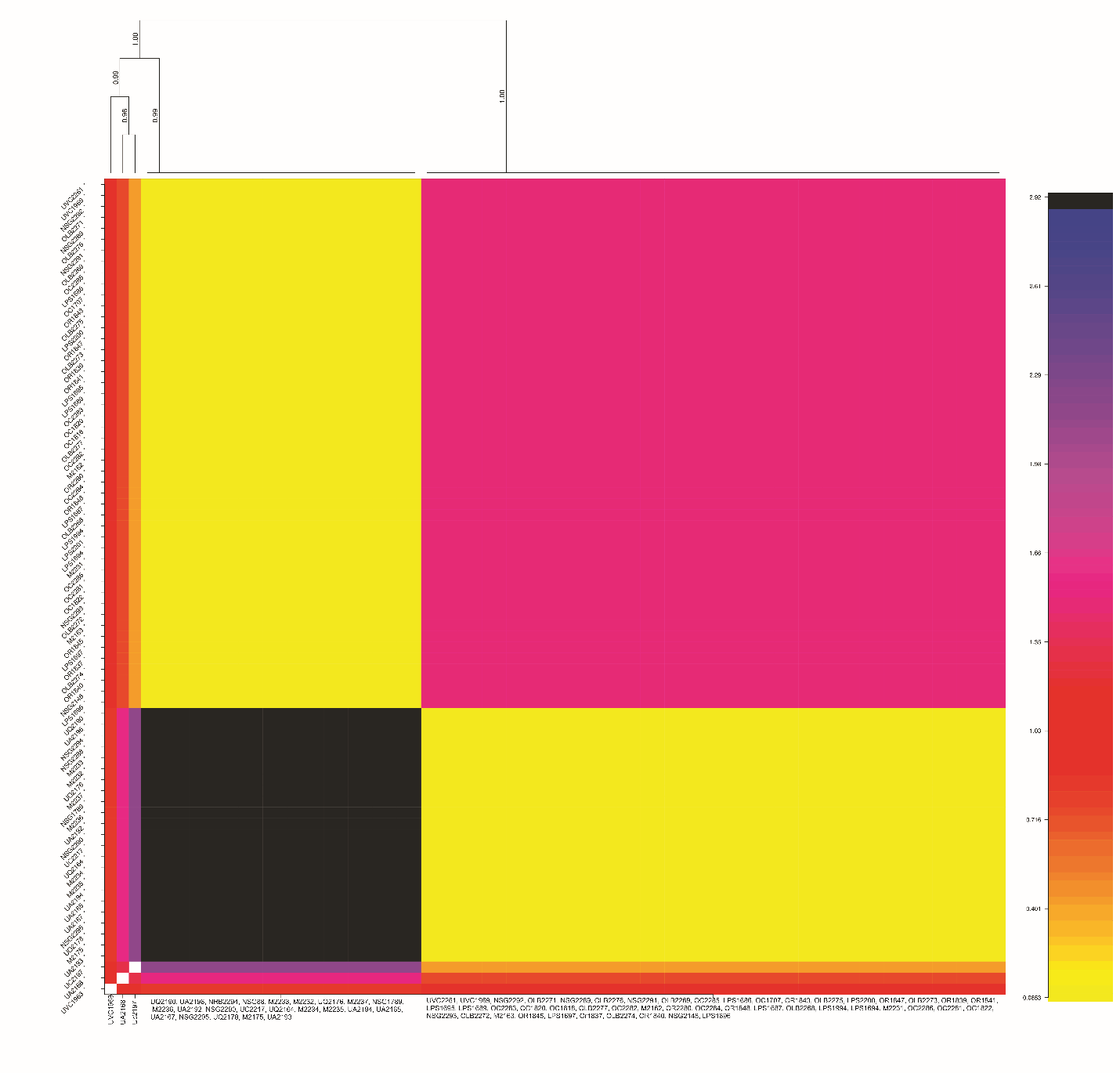


Figure S 3. *Rhamdia quelen* fineRADstructure plot based on Outlier *loci*. Horizontal axis shows cluster of individuals and vertical axis shows each individual.
